# Integrated Assessment and Prediction of Transcription Factor Binding

**DOI:** 10.1371/journal.pcbi.0020070

**Published:** 2006-06-16

**Authors:** Andreas Beyer, Christopher Workman, Jens Hollunder, Dörte Radke, Ulrich Möller, Thomas Wilhelm, Trey Ideker

**Affiliations:** 1 Department of Bioengineering, University of California San Diego, La Jolla, California, United States of America; 2 Leibniz Institute for Age Research, Fritz Lipmann Institute, Jena, Germany; 3 Leibniz Institute for Natural Product Research and Infection Biology, Hans Knöll Institute, Jena, Germany; EMBL Heidelberg, Germany

## Abstract

Systematic chromatin immunoprecipitation (chIP-chip) experiments have become a central technique for mapping transcriptional interactions in model organisms and humans. However, measurement of chromatin binding does not necessarily imply regulation, and binding may be difficult to detect if it is condition or cofactor dependent. To address these challenges, we present an approach for reliably assigning transcription factors (TFs) to target genes that integrates many lines of direct and indirect evidence into a single probabilistic model. Using this approach, we analyze publicly available chIP-chip binding profiles measured for yeast TFs in standard conditions, showing that our model interprets these data with significantly higher accuracy than previous methods. Pooling the high-confidence interactions reveals a large network containing 363 significant sets of factors (TF modules) that cooperate to regulate common target genes. In addition, the method predicts 980 novel binding interactions with high confidence that are likely to occur in so-far untested conditions. Indeed, using new chIP-chip experiments we show that predicted interactions for the factors Rpn4p and Pdr1p are observed only after treatment of cells with methyl-methanesulfonate, a DNA-damaging agent. We outline the first approach for consistently integrating all available evidences for TF–target interactions and we comprehensively identify the resulting TF module hierarchy. Prioritizing experimental conditions for each factor will be especially important as increasing numbers of chIP-chip assays are performed in complex organisms such as humans, for which “standard conditions” are ill defined.

## Synopsis

Transcription factors (TFs) bind close to their target genes for regulating transcript levels depending on cellular conditions. Each gene may be regulated differently from others through the binding of specific groups of TFs (TF modules). Recently, a wide variety of large-scale measurements about transcriptional networks has become available. Here the authors present a framework for consistently integrating all of this evidence to systematically determine the precise set of genes directly regulated by each TF (i.e., TF–target interactions). The framework is applied to the yeast Saccharomyces cerevisiae using seven distinct sources of evidences to score all possible TF–target interactions in this organism. Subsequently, the authors employ another newly developed algorithm to reveal TF modules based on the top 5,000 TF–target interactions, yielding more than 300 TF modules. The new scoring scheme for TF–target interactions allows predicting the binding of TFs under so-far untested conditions, which is demonstrated by experimentally verifying interactions for two TFs (Pdr1p, Rpn4p). Importantly, the new methods (scoring of TF–target interactions and TF module identification) are scalable to much larger datasets, making them applicable to future studies in humans, which are thought to have substantially larger numbers of TF–target interactions.

## Introduction

Combinatorial transcriptional regulation is an important means of achieving highly specific expression of individual genes using small groups of transcription factors (TFs) [1–7]. These groups, called TF modules [3–6], integrate signals from different pathways to fine-tune the cellular response at the transcriptional level. The complexity of transcriptional regulation in higher species suggests that combinatorial regulation is of particular importance for metazoans [5,8]. However, detecting biologically significant TF modules is only possible if the gene targets regulated by each TF are known with high accuracy.

Recently, measurement of TF–target binding relationships has become much more systematic through the technique of chromatin immunoprecipitation coupled with microarray chips (chIP-chip) [9–11]. By this approach, a TF of interest is immunoprecipitated along with all of the gene promoters and other genome fragments it binds in vivo; these fragments are identified by hybridization to a DNA microarray, thus elucidating all of the promoters bound directly by that TF. However, observed DNA binding in an upstream region alone is not always sufficient to indicate true interaction between a TF and a potential target gene [11,12]. Even if binding occurs, the event may not be biologically relevant, or the observed binding may relate to some cellular function other than gene expression. Moreover, unlike genome sequencing, which has a well-defined endpoint, interaction mapping projects are difficult to “complete” because a cell's pattern of interactions is strongly dependent on variables such as the cell type, genetic background, stage of development, time after stimulus, or specific environmental or biological condition. Accordingly, many true binding events may be missed by chIP-chip because the relevant conditions have not yet been examined.

Therefore, to correctly interpret measurements of TF–target binding, there is a need for computational methods that (1) identify which binding interactions have a regulatory function; (2) provide insight into new TF–target relationships that are likely to be condition-specific; and (3) perform an efficient yet exhaustive identification of TF modules, including quantification of their statistical significance. Existing bioinformatic approaches for assigning TFs to target genes rely on stepwise integration of one or a few lines of evidence, such as combining chIP-chip data [11] with TF binding motifs or coexpression [3,4,13–18]. Other approaches combine TF binding locations with diverse biological data to infer regulatory networks [19,20], but require the prior assignment of interactions and interaction probabilities.

Here, we implement a Bayesian approach that integrates all available types of genome-scale evidence to construct accurate transcriptional regulatory networks. In addition to measurement of direct promoter binding and detection of DNA binding motifs, we find that evidence of gene fusion and shared phylogenetic profiles (i.e., co-occurrence in a significant number of species) is surprisingly informative for predicting true regulatory interactions. High-confidence interactions are used to identify TF modules (i.e., sets of TFs that cooperate to regulate a significant number of genes in common). Application of this procedure to integrate genome-scale data for yeast reveals a large hierarchical network of regulatory relationships and predicts many new condition-specific transcriptional interactions. We validate several of these interactions through new chIP-chip experiments for Rpn4p and Pdr1p, two transcription factors predicted to bind many new targets in response to chemical stress. Incorporation of these new binding data into modules reveals cross-talk between TFs involved in the response to stress, histone regulation, and regulation of the cell cycle.

## Results/Discussion

### Overview of the Approach

**Figure 1:**
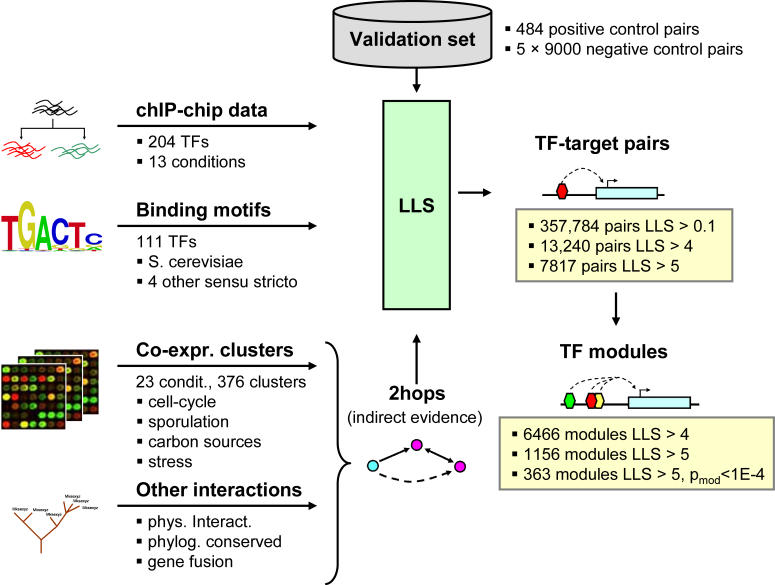
Identifying High-Confidence TF–Target Interactions and TF Modules Different lines of evidence indicative of TF–target interactions are combined to yield an integrated probabilistic measure of interaction propensity. Using a positive and a negative validation set, the input evidences are independently converted into LLSs. Individual LLSs are integrated into one value per TF–target pair. TF modules are identified as subsets of TFs that regulate common genes.

To permit the construction of accurate transcriptional networks, we developed an integrative framework to quantify the likelihood of direct regulatory interaction between a TF and each of its possible target genes (Figure 1). The TF–target assignment relies on a Bayesian classifier to consistently integrate many different lines of experimental evidence, as has been successfully applied for associating genes with similar function [21–24] or for predicting genetic interactions [25]. Based on control sets of known true and false transcriptional interactions, a log-likelihood score (LLS) is calculated for each type of evidence, which quantifies the likelihood that a given TF–target relationship is correct. Individual LLSs are then added together to compute an overall integrated LLS for the interaction (the so-called “naive” Bayesian approach); this combination of evidence types into a common probabilistic measure eliminates the need for discretization (applying a *p*-value threshold) and accounts for the relative predictive power among the input data types.

Given a method for assessing interaction reliability, we also sought to organize high-confidence interactions into TF modules (i.e., sets of TFs that cooperatively regulate sets of genes). For this purpose, we applied an algorithm that identified all TF combinations regulating common targets and assigned *p*-values of significance to these overlaps [26]. This method of module identification is scalable to much larger datasets, which will be particularly necessary in view of the complex transcriptional regulation observed in higher eukaryotes [8]. Given a set of TF modules, the integrated LLSs could be subsequently refined in a process that examined the overlap between modules and gene expression clusters. Further details on the Bayesian integration and the module identification procedures are provided in Materials and Methods.

### Diverse Evidence Types Are Informative of True TF Interactions

**Figure 2:**
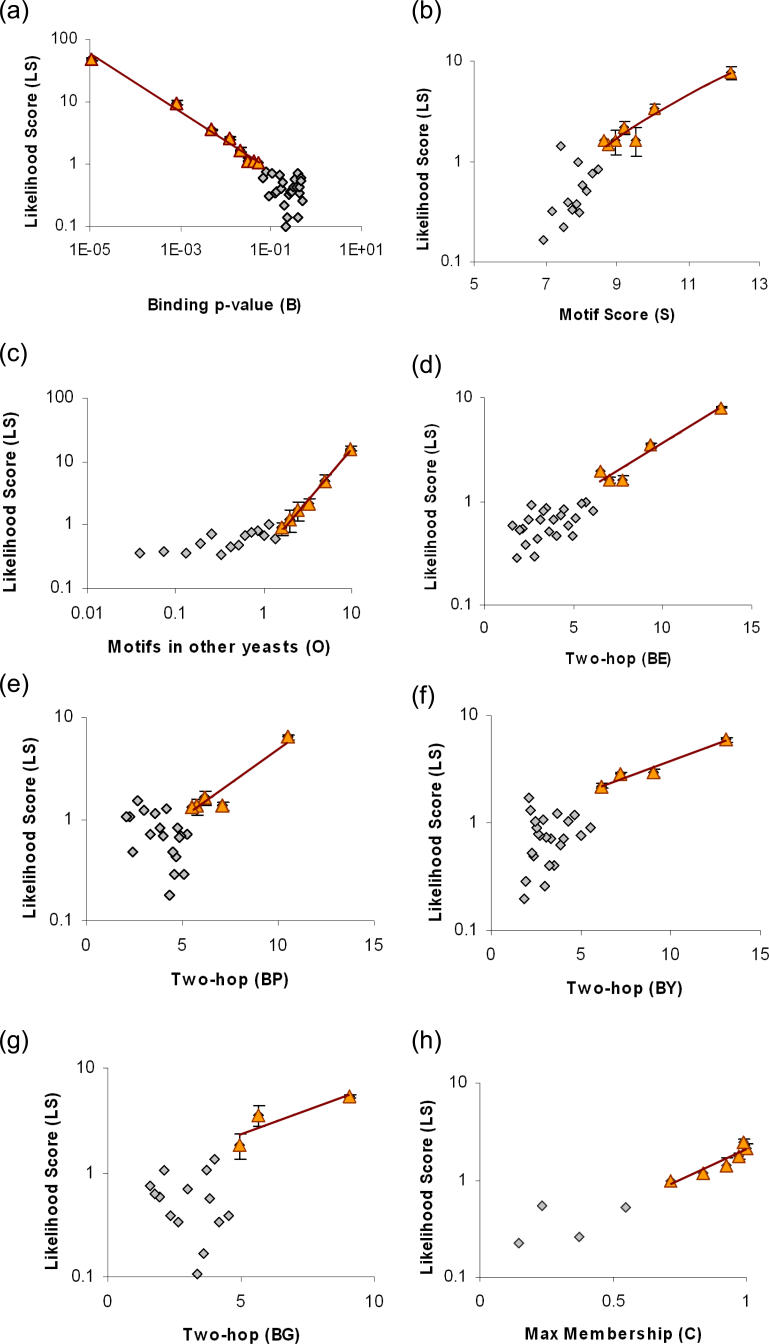
Regression Lines Used for Scaling the Different Evidence Types Needed for Predicting TF–Target Interactions TF–target pairs were binned according to the value of the respective evidence type, and the LS for each bin was calculated using the validation sets (Equation 1). Each point is the average of five runs with different negative validation sets (the positive set was always the same). Error bars represent standard deviations over the validation sets. Gray diamonds lie in parameter ranges that were excluded from the LS prediction because the LSs were not significant. Abbreviations used in the *x*-axis labels are explained in the main text.

We applied our integrative Bayesian approach to assign confidence scores to every potential TF–target pair in yeast. Seven distinct lines of evidence were made available to the model (Figure 1): DNA binding intensities measured with chIP-chip technology [11] (*B*), TF binding motifs in *S. cerevisiae (S)* and four other sensu stricto species *(O),* coexpression information *(E),* physical protein–protein interactions [21] *(P),* gene pairs with shared phylogentic profiles [21] *(Y),* and pairs of genes that were fused together in other species [21] (*G*). Initially, we found that only *B, S,* and *O* were directly predictive of known transcriptional interactions (Figure 2A–2C). However, further analysis showed that the remaining types of evidence were able to predict TF–target interactions indirectly when combined transitively with a second line of evidence in what has been called a “2hop” [25] relationship. For example, if transcription factor *X* regulates gene *Y* and gene *Y* is coexpressed with gene *Z,* then a 2hop relationship exists between *X* and *Z.* The 2hop relationship has been applied for predicting synthetic-lethal interactions [25] but, as shown in Figure 2, it can also be surprisingly predictive of TF binding.

Four types of 2hops were examined, in which the first hop *(X* → *Y)* was always measured by TF binding (evidence *B*), and the second hop *(Y* ↔ *Z)* was supported by evidences *E, P, Y,* or *G,* giving 2hops *BE, BP, BY,* and *BG,* respectively (Figure 2D–2G). While it is well established that coexpressed genes *(BE)* or those that encode interacting proteins *(BP)* tend to be coregulated [7,15,26,27], the findings that gene fusion *(BG)* or shared phylogenetic profiles *(BY)* can indicate coregulation are potentially less intuitive. In this case, phylogenetic information *(G* and *Y)* is employed for the identification of functionally related genes in S. cerevisiae [28,29]. Such gene pair then has an increased likelihood of being regulated by the same TF(s). The last evidence type that we used employs a coexpression criterion: after an initial assignment of TFs to coexpressed clusters, we use cluster membership as additional evidence for a TF–target interaction (evidence *C,* see Materials and Methods for details).

**Figure 3:**
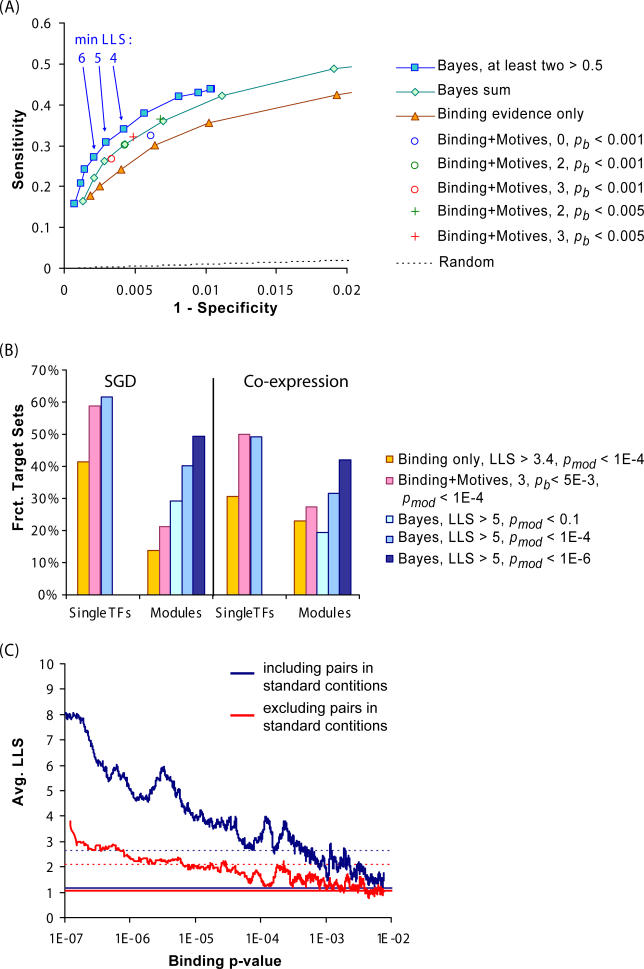
Quality Assessment of the Predicted TF–Target Gene Interactions (A) ROC curves are average of two cross-validations (see Materials and Methods). Lines show specificity and sensitivity accounting for binding evidence only and for integrating all evidences based on the Bayesian approach (with and without [“Bayes sum”] additional filtering). Additional filtering requires that at least two evidences have LLS > 0.5 (see Materials and Methods). Single points refer to previous selections [11] based on binding evidence (chIP-chip, *p_b_* < 0.001, *p_b_* < 0.005) and motif presence in zero, two, or three yeast species, respectively. Blue arrows indicate the respective LLS thresholds. (B) Target gene sets were validated against Gene Ontology categories taken from SGD [31] and clusters of coexpressed genes (see Materials and Methods). In the latter case, all evidences based on expression data were excluded when assigning TFs to targets. The vertical bars indicate the fractions of TFs or TF modules for which the target genes significantly overlap with at least one category or cluster (*p* < 10^−4^, hypergeometric distribution). The filtering criteria for the three sets of predicted interactions were chosen such that all selections have the same specificity (0.995). Yellow indicates using binding *p*-values as the sole selection criterion; green, selections by Harbison et al. [11] based on binding motifs conserved in at least three species and with binding *p*-values *(p_b_)* < 0.005; and blue, combining all possible lines of evidence; at least two predicted LLSs must be > 0.5; the sum of all evidences must yield a LLS > 5. All modules are significant with *p*_mod_ < 10^−4^, except for the light and dark blue bars (*p*_mod_ < 0.1 and < 10^−6^, respectively). The *p*_mod_ does not apply to the single TFs. (C) LLSs were determined based on all evidences, but excluding binding under nonstandard conditions. The average LLS (sliding window) is plotted versus binding *p*-values under nonstandard conditions. Blue line indicates all TF–target pairs; red line, subset excluding pairs binding under standard conditions (i.e., LLS is exclusively based on evidences other than binding). Horizontal lines indicate global average LLS (solid lines) and average plus one standard deviation (dashed lines).

Combining all lines of evidence (*B, S, O, C,* and 2hops) yielded a total of 7,817 high-confidence interactions with integrated LLS > 5 (Table S1). We found that the distinction of known true and false interactions could be further improved by requiring that one of the evidences for DNA binding *(B, S)* and one evidence for functional interaction *(O, BE, BP, BY, BG, C)* have an LLS > 0.5 (Figure 3A). Binding in upstream regions does not always imply functional regulation of downstream genes. The additional filtering ensures that there is significant evidence for both upstream binding and functional interaction (see Materials and Methods for details). Applying this additional filtering criterion reduced the number of interactions to 5,245 (involving 117 different TFs).

2hops were informative for scoring a substantial number of putative transcriptional interactions (Table S1). For instance, for 359 high-confidence predictions (LLS > 5), the underlying evidence was based exclusively on 2hops and membership in a coexpression cluster, without observed chIP-chip binding and without significant binding motifs. By the same criterion, another 419 (8%) interactions with significant observed binding were supported only by 2hops or cluster membership but not by DNA binding motifs. Given the absence of observed motifs, it is possible that these TFs do not directly bind DNA but serve as cofactors together with DNA-binding TFs. Two well-known examples of cooperative regulation “at a distance” are the histone regulators Hir1p and Hir2p [30]. Based largely on 2hops, our model obtained very consistent evidence for interactions connecting these two factors to eight histone-related genes (Table 1).

**Table 1:**
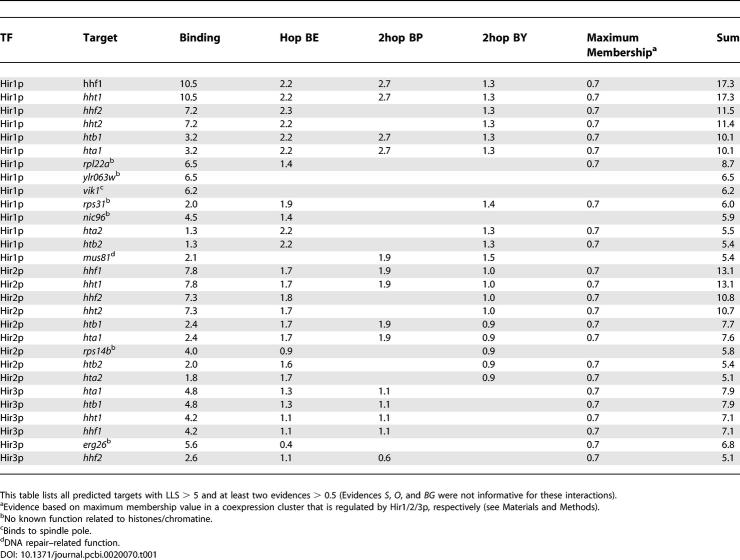
Evidences Supporting Hir1p and Hir2p Binding

### TF Module Hierarchy

**Figure 4:**
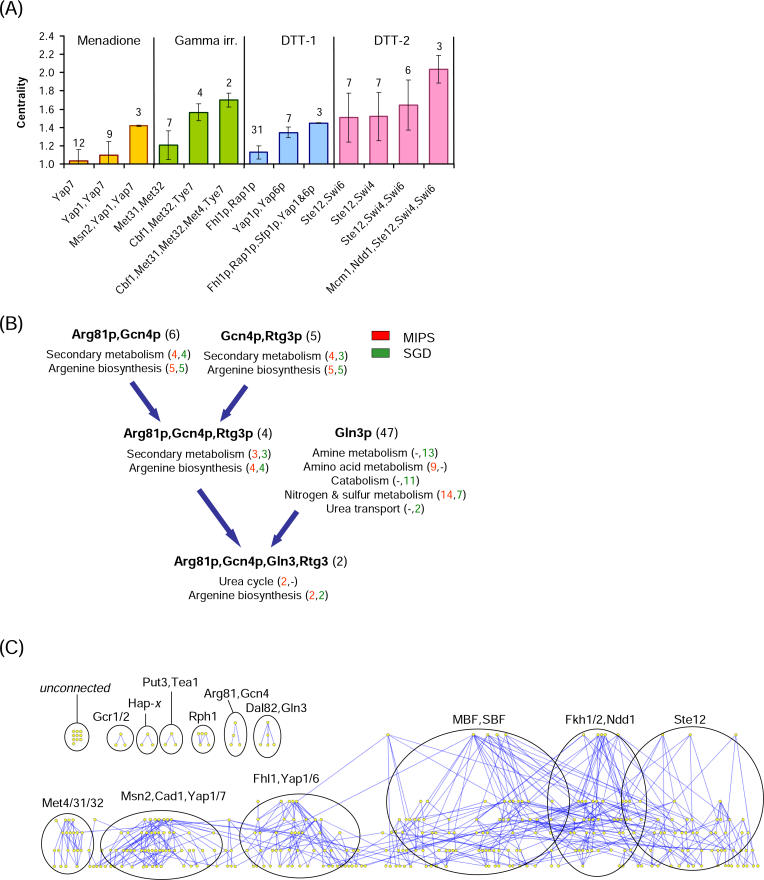
Combinatorial Regulation by TF Modules (A) Bars show average centrality (see Materials and Methods) of target genes overlapping with stress-related clusters (± standard error). Values above bars are numbers of overlapping target genes. Generic TFs such as Yap1p or Swi6p are reused in several modules. Combination with other TFs yields specificity (i.e., a smaller number of target genes and an increased centrality). (B) Hierarchy of TF modules. Arrows represent a subset relationship (i.e., all TFs of the source module are contained in the target module). Downstream TF modules always share their targets with upstream TF modules. Annotations are based on significant (*p* < 10^−4^, hypergeometric distribution) overlaps between the target gene sets and the respective functional category. Values in parentheses are numbers of target genes (black) and numbers of overlapping genes (red, green). (C) Complete hierarchy of the 363 significant TF modules (*p*_mod_ < 10^−4^). Highlighted regions contain TF modules that are enriched with the respective TFs or TF complexes.

Pooling high-confidence TF–target interactions revealed a total of 363 significant TF modules (*p*_mod_ < 10^−4^), each of which contained two to 13 distinct TFs. Examples of identified modules are shown in Figure 4, while lists of TF modules at different confidence levels can be found in Tables S2 and S3. Table S4 lists 1,122 significant (*p* < 10^−4^) overlaps between target gene sets and coexpression clusters.

Figures 4A and 4B illustrate how small TF modules (two to three TFs) that may regulate a broad spectrum of diverse functions can combine into larger highly specific regulatory units. The TF module hierarchy arising from this “specification by combination” gives rise to a complex network; key modules such as SBF (Swi4p, Swi6p) or MBF (Mbp1p, Swi6p) appear as network hubs (Figure 4C and Table 2), which typically share large numbers of target genes, whereas TF combinations at the leaves of the network are large (>3 TFs) and have only few target genes.

**Table 2:**
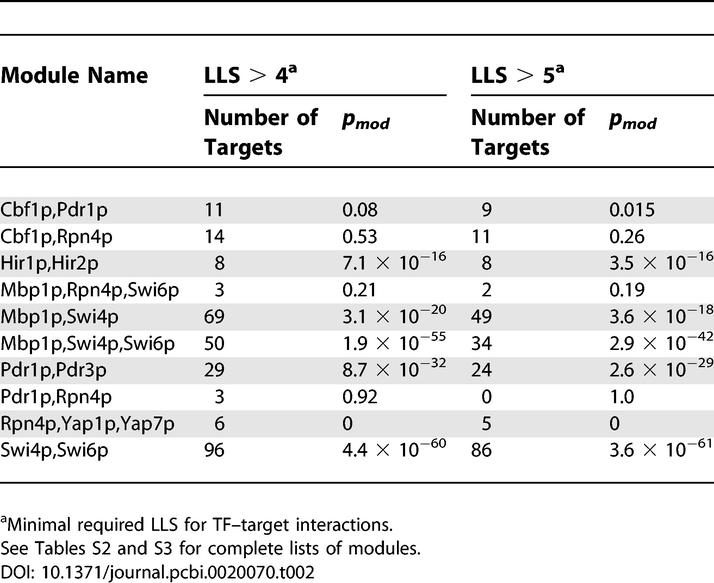
Selected TF Modules Discussed in the Text

### Benchmarking and Comparison to Previous Approaches

We next compared the integrative approach (“Bayes”) to two previous methods, one based on a chIP-chip binding measurement alone (“binding only”) [10,11], and the other requiring the presence of a conserved TF binding motif in addition to observed binding (“binding + motif”) [11]. In a two-fold cross-validation we randomly split the reference interactions into two datasets (A and B) of equal size. Subsequently, we used A to train the statistical model and tested it on B and vice versa. Figure 3A shows that binding alone is already a good predictor of true positive and true negative interactions. However, accounting for additional evidences improves the sensitivity significantly. The remaining analysis is based on a Bayesian model trained on the full reference dataset. Since the dataset is large (>480 positive control interactions, >9,000 negative control interactions) compared to the number of trained parameters (three parameters per line of evidence, eight evidences), the final model is unlikely to suffer from overfitting.

While the receiver operator characteristic (ROC) curves imply better coverage of our approach, we also wanted to assess the quality of these predictions. If several target genes are regulated by the same TF, one might expect these genes to be coexpressed and to have similar cellular functions. This notion provided a means to benchmark the integrative Bayes classifier versus the other methods for TF–target assignment. Figure 3B shows the fraction of TFs for which the target genes are functionally homogeneous according to the *Saccharomyces* Genome Database (SGD) [31] (similar results were obtained for other thresholds and using annotations from the Munich Information Center for Protein Sequences (MIPS) [32]; Figure S1). Gene annotations were not used in the classifier and thus provided an independent assessment of accuracy. Similarly, target gene sets were overlaid with clusters of coexpressed genes. For both databases and the coexpression analysis, the “binding + motif” method outperformed “binding only,” with the Bayesian classifier outperforming both methods in all but one case. Figure 3B also demonstrates that TF modules with lower *p*-values are functionally more homogeneous and more likely to be coexpressed than modules with higher *p*-values. Hence, the statistical significance expressed by the module *p*-value (*p*_mod_) can be interpreted as a biological significance.

### Prediction of New Transcriptional Interactions

Beyond assigning confidences to raw interaction measurements, we investigated whether an integrative approach could predict interactions that had not yet been observed experimentally. Overall, our high-confidence set of 5,124 TF–target pairs included 980 interactions that were based on multiple lines of evidence but were not supported by direct chIP binding (LLS for binding < 0.05). We hypothesized that for many of these TF–target pairs, direct binding might indeed occur but in conditions that had not been previously measured. Although the available chIP binding data included profiles for most TFs in nominal conditions (YPD media), few of these factors had been examined in more than one to two other conditions [11].

To test our hypothesis, we applied a cross-validation procedure in which LLS values were recalculated using only chIP data from nominal conditions, and the resulting TF–target pairs with high LLSs were compared with the available binding measurements from other growth conditions. As shown in Figure 3C, we found that TF–target interactions from other conditions tended to have large LLS values, many of which were due to the presence of binding motifs and/or informative 2hops. Thus, it is likely that many TF–target pairs predicted with high confidence may simply have not yet been observed in the appropriate condition(s).

### Discovery and Validation of Rpn4p and Pdr1p Transcriptional Reprogramming

**Figure 5:**
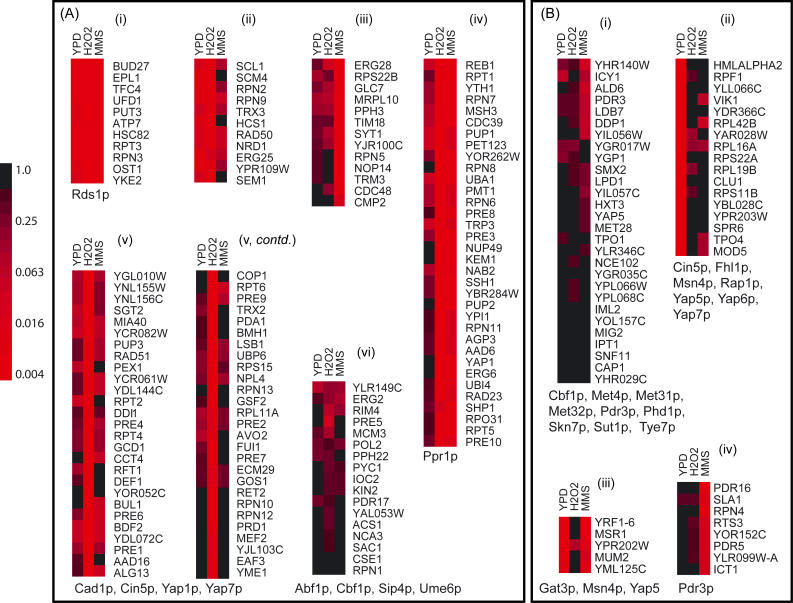
Rpn4p and Pdr1p Binding under Normal and Stress Conditions (H_2_O_2_ and MMS) Binding *p*-values for MMS (this study) and other conditions (taken from [11]) are shown for groups of (A) Rpn4p and (B) Pdr1p targets (LLS > 5) with coherent binding patterns (red, strong binding; black, no binding). Additional transcription factors coregulating a significant (*p* < 0.001) number of genes either as individual TFs or as members of TF modules are listed below each cluster.

Encouraged by the above cross-validation results, we sought to experimentally verify several of the interactions predicted to operate under new conditions. Rpn4p and Pdr1p exhibit significant transcriptional reprogramming under oxidative stress (Figure 5). This implies a role of these regulators in detoxification and/or DNA repair [33,34]. Accordingly, we performed genome-wide chIP-chip analysis for these two factors in cells grown in the presence of 0.03% methyl-methanesulfonate (MMS), an alkylating agent that causes damage to DNA and other cellular components [35].

Of the 104 predicted interactions (LLS > 4) for Rpn4p and Pdr1p that did not have prior chIP-chip binding evidence, 19 had significant *p*-values of binding under MMS (Table 3; overlap is significant at *p* = 1.7 × 10^−7^, hypergeometric distribution). Accordingly, Figure S2 shows that TF–target pairs observed under MMS tend to also have high LLS values according to the Bayes classifier. Thus, the LLS can predict novel DNA binding interactions, even if no such binding has been observed previously.

**Table 3:**
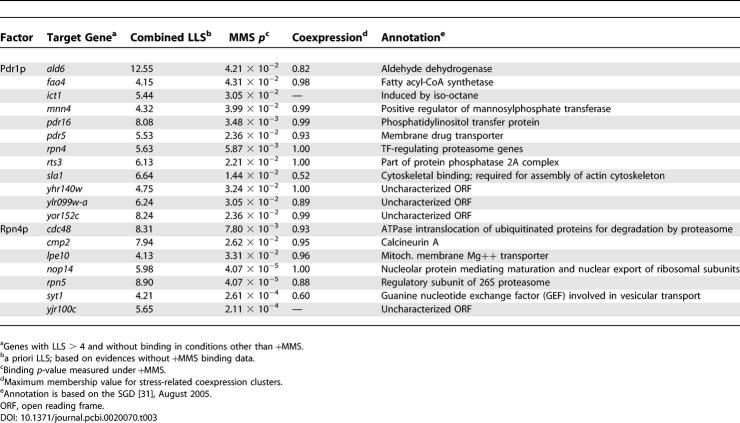
Experimental Validation (chIP-chip) for Pdr1p and Rpn4p under MMS

Figure 5 shows genes with coherent binding patterns of Rpn4 (Figure 5A) or Pdr1p (Figure 5B) across nominal conditions, oxidative stress, and stress due to MMS. The following three groups of genes can be distinguished: genes that are targeted under all conditions (cluster a-i); genes that are targeted under stress conditions in general (cluster a-iv); and genes that are targeted under one stress specifically (e.g., clusters a-v or b-iii). Clusters a-vi and b-i contain genes without observed binding of the two TFs under any of the tested conditions. The large LLSs of these interactions imply that most of these genes are in fact regulated by Rpn4p and Pdr1p, but under other untested conditions.

Relating the new MMS binding data to TF modules suggested that, although both TFs respond to DNA damage, they regulate distinct sets of genes in a nonredundant manner. First, Pdr1p and Rpn4p were never present in the same module (they had no common targets at LLS > 5). Instead, Pdr1p formed a TF module with Pdr3p (Table 2), reflecting an earlier observation that Pdr1p and Pdr3p can bind as homo- or heterodimers to the same binding sites [33]. On the other hand, Rpn4p shared targets predominantly with other stress-related TFs such as Yap1p or Yap7p. Either Pdr1p or Rpn4p could form a module with the cell-cycle regulator Cbf1p (Figure 5 and Table 2); the co-occurrence of stress- and cell-cycle–related TFs in the same module is not surprising since stress signals may induce cell-cycle arrest [1].

Rpn4p and Pdr1p exhibit distinct stress response schemes. While Rpn4p primarily binds under stress conditions but not under nominal conditions, Pdr1p binds a large fraction of its targets under nominal conditions. These binding sites are released by Pdr1p under stress (clusters b-ii and b-iii). A second group of Pdr1p targets comprises genes that are unbound under any of the tested conditions (cluster b-i) or just weakly bound under MMS stress (cluster b-iv). Binding of Pdr1p was not observed for any of its significant (LLS > 5) targets under oxidative stress. The distinct regulatory patterns are at least partially explained by cofactors that act in concert with Rpn4p or Pdr1 in a modular fashion. For instance, clusters b-i and b-iv are regulated by Pdr1 and Pdr3p together, whereas clusters b-ii and b-iii contain no targets of Pdr3p. A consistent pattern emerged indicating that genes regulated by Pdr1p but not by Pdr3p are bound under nominal conditions, whereas those regulated by the Pdr1p/Pdr3p complex are not. In support of previous speculations our findings suggest that dimer composition affects binding site specificity of Pdr1p and Pdr3p [33].

### Conclusions

In summary, we have developed an approach for assigning likelihood scores to transcriptional interactions based on integration across eight types of direct and indirect evidence. The integration of different lines of evidence serves two major purposes: first, if binding was already observed in chIP-chip experiments, additional evidence helps reduce the number of false positive predictions by verifying that the interaction between a TF and its target gene is functional. Secondly, if no binding has been observed, other evidences may reduce false negative predictions and suggest that interactions may occur under so-far untested conditions. Based on the latter, we were able to experimentally confirm 19 new transcriptional interactions that are active during damage-related stress. We have also explored how high-confidence TF–target interactions can be used to infer the TF module hierarchy underlying transcriptional gene regulation. In this regard, our analysis of modules involving Pdr1p, Rpn4p, Hir1p, and Hir2p suggested how cells achieve a high degree of specificity by combining generic factors with other more specific factors into complex regulatory units. Although we have focused on yeast, the framework is general and may be especially relevant as large-scale transcriptional mapping projects get under way in humans.

## Materials and Methods

### Control sets.

A set of 484 high-confidence TF–target interactions was created as a positive control by extracting regulatory interactions from the Incyte YPD Database (http://www.incyte.com), which is based on a curated, literature-derived dataset. In order to obtain negative control data, five sets of random TF–gene associations were generated, where each set contained > 9,000 interactions. Cocitation criteria [21] were applied to further enhance the stringency of the control sets: interactions in the positive control set were required to have a significantly enriched number of cocitations (*p* < 0.1), whereas interactions in the negative control set were required not to have a cocitation link.

### Likelihood ratios.

LLSs were calculated as described in Lee et al. [21]:

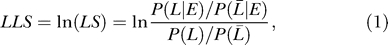
where *P*(*L*) is the *prior* probability of observing a true TF–target interaction, *P*(*L̄*) is the *prior* probability of not observing a true TF–target interaction, *P*(*L*)/*P*(*L̄*) is the *prior* likelihood ratio for observing a true interaction, and *P*(*L*|*E*)/*P*(*L̄*|*E*) is the *posterior* likelihood ratio after observing the evidence *E.* Input evidences were binned ensuring that bins for one evidence type always have the same size and LLSs were calculated for each bin based on the positive and negative training sets. The best regression (maximizing R^2^) through the resulting LLSs was used for predicting LLSs of unknown TF–gene pairs (Figure 2). In the case of chIP-chip measurements, the likelihood ratio of each bin was plotted versus the minimal binding *p*-value of all available conditions (Figure 2A). For the cross-validation (Figure 3C), binding *p*-values were restricted to YPD only for training the scoring scheme, and the remaining conditions were used for testing.

Adding LLSs of different evidences is appropriate if the input data are statistically independent. Lee et al. [21] propose a weighing factor *D* accounting for dependence between evidences. We used ROC curves and Mathew's correlation coefficient (MCC) [36] to judge the quality of different weighing factors; the best performance was obtained by simple addition of LLSs (i.e., *D* = 1). Filtering positive from negative interactions was improved by additionally requiring that at least one of the two evidences *(B* or *S)* and one of the remaining evidence types have LLS > 0.5. If written as pseudocode, this rule reads: SELECT IF( (*B* > 0.5 OR *S* > 0.5) AND (*O* > 0.5 OR *TE* > 0.5 OR…OR *C* > 0.5) ). The rationale for this grouping of evidences is that *B* and *S* provide evidence for (possible) upstream binding, but they do not imply a true regulatory interaction. The remaining evidences, on the other hand, functionally link the TF to its target gene. Note that evidence *O* implies that the binding site is conserved upstream of the respective gene in a significant number of species, which also suggests a functional interaction. Different LLS thresholds were tested in steps of ΔLLS = 0.05 to maximize the MCC. The threshold at 0.5 was found to maximize the MCC. The final predictions were based on the sum of all evidences (Figure 3A) and the LLS threshold that maximized the MCC (i.e., LLS > 5; Figure S3). The maximum MCC is the same (0.4) when using the split reference data (cross-validation) and when using the full dataset for training.

### Coexpression evidence.

The score for TF–target interactions was computed over two steps. In the first step, an initial set of target genes (LLS > 6) and TF modules was identified with high confidence. In the second step, these high-confidence target sets were used to search for additional targets that were coexpressed with the existing ones, in a manner similar to Bar-Joseph et al. [4] For the second step, expression data covering a broad range of cellular functions (Figure 1) were clustered as described below, yielding a membership value for every gene in every cluster. Significant overlap (*p* < 10^−4^, hypergeometric distribution) between a target gene set and a coexpression cluster was taken as evidence that every gene in this cluster was regulated by the corresponding TF (or TF module). The cluster membership values of significant clusters were used as additional evidence for TF–target interaction and were translated into LLSs accordingly. If a gene was the member of several clusters regulated by the same TF, the maximum membership value was selected for computing the LLS. This new line of evidence, *C,* was added to the LLSs determined in the first step.

### Binding motifs.

TF binding site motifs were defined as position-specific weight matrices (PWMs) [37]. PWMs were compiled for 111 different individual TFs from Harbison et al. [11] and from public databases [38,39]. When more than one matrix was defined for the same TF, the PWM with the highest information content per position (relative entropy) was selected. Using the PWM scoring functionality of ANN-Spec [37], the score distribution for each motif was determined over possible subsequences of all intergenic regions such that a score threshold could be selected to ensure that the fraction of predicted binding sites was < 10^−4^.

The genome sequences of promoter regions in S. cerevisiae (2,000 bp upstream of each gene) and of the promoters of homologous genes in four other sensu stricto species (1,000 bp upstream of each homolog) were obtained from SGD [31] (download from June 2005; Washington University, St. Louis, Missouri, United States and Massachusetts Institute of Technology, Cambridge, Massachusetts, United States). Log-likelihood ratios were calculated separately for every species and the LLSs of the four related species were added into one LLS for the evidence *O* (“binding motif in other yeast species”).

### 2hops.

A 2hop relationship exists between a transcription factor, *A,* and a gene, *C,* via an intermediate gene, *B,* if there is evidence that *A* regulates *B,* and *B* is functionally linked to *C.* The two evidence types are then transformed into respective likelihood scores *LS_AB_* and *LS_BC_.* The product of the two LSs is proportional to the product of the *posterior* likelihood ratios:


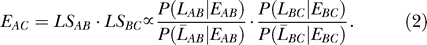


Note that the denominator in Equation 1 is essentially the fraction of true interactions among all possible interactions [21]; hence, it is a constant for all interaction pairs *AB* and *BC.* Therefore, *E_AC_* from Equation 2 is proportional to the probability that the network path from *A* via *B* to *C* actually exists given the evidences *E_AB_* and *E_BC_.* Thus, *E_AC_* served as evidence for a direct link from *A* to *C* and the likelihood ratio *LS_AC_* was calculated from *E_AC_* based on the training data in the same way as for all other input evidences. In this study *LS_AB_* was always based on chIP-binding *p*-values, and *LS_BC_* was taken from Lee et al. [21].

### Transcription factor modules and *p*-value estimation.

TF modules were determined using our previously described method [26], yielding closed sets of TFs associated with distinct sets of target genes. The *p*-values quantify the likelihood of observing the given TF module in a randomized regulatory network of the same size and same number of TFs. Briefly, a TF module *M* is defined as a set of *n* distinct transcription factors *(m_1_*,… , *m_i_*, …, *m_n_).* Let *F* be the total number of all TF–target interactions and *f_i_* be the number of interactions from *m_i_* to its target genes in the entire network. We then compute the relative frequency of *m_i_* as *φ_i_* = *f_i_*/*F*. A random set of *n* TFs has *n*! different permutations and thus, the probability of finding *M* in a random set of *n* transcription factors is *n*! × Π*φ_i_*. Note that this implicitly assumes that the probability of drawing a TF *i* is independent of the other TFs in the module. This assumption may be violated for small numbers of TFs, because the probability of drawing one TF would then depend on the TFs that have already been withdrawn from the “pool.” In our case we have >100 different TFs. We compared the direct estimation of the *p*-values with random permutations of the TF–target interactions to verify that the pool size does not affect the *p*-values. We observed no significant deviations between the two schemes (data not shown). Next, it is possible to calculate the probability, *p_M_,* of finding *M* in the set of *k* ≥ *n* TFs that regulate a given gene. This *p_M_* can be computed as 1 minus the probability of not finding *M* in 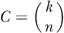 random trials:


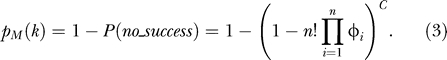


Note that a TF occurs at most once in every set. The *p_M_* are calculated for all set sizes *k* appearing in the original (observed) data and a weighed sum *P_M_* is calculated as

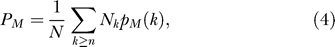
where *N_k_* is the number of sets of size *k* and *N* is the total number of sets *≥ n* (i.e., the number of target genes). Equation 4 gives the average probability of finding *M* in one random set. A binomial distribution is assumed for estimating the probability *p*_mod_ of module occurrence (i.e., number of genes regulated by *M*).

Apart from being scalable, our approach has a number of advantages in comparison to existing algorithms for finding TF modules [4,15,18,40–42]: (1) all available evidences can be integrated into one common score; (2) the variable predictive power of different evidences is taken into account; (3) there is no size threshold on the number of TFs in each module; (4) all modules at all hierarchical levels are identified, without the need to restrict the search to a specific hierarchical level (“slicing”; see also [18]); (5) target genes and TFs can be members of several modules; (6) the algorithm is not restricted to TFs with known binding matrices; and (7) we assign a *p*-value to every TF module based on the number of target genes.

The importance of some of these aspects has been discussed previously [18,42]. Existing approaches cover some of these features (e.g., genetic regulatory modules (GRAM) [4] fulfills 5 and 6 or the extended signature algorithm [42] agrees with 3, 4, and 5). Features 1, 2, and 7 are unique to our method.

### Coexpression clustering and centrality.

Microarray mRNA expression data were taken from the literature [1,43–45] (23 different conditions, 310 profiles). Genes were clustered separately for each study or group of conditions (i.e., cell cycle, stress-related, metabolism) using only genes that changed significantly (standard deviation of log_2_-fold change > 0.45) [46]. Gene clusters were obtained using a multistep procedure that determines the total number of clusters *(k)* and the cluster membership of each gene. Within each step, clustering is performed using the fuzzy c-means algorithm [46], which estimates the probability of membership of every gene to each cluster. The initial *k* was set to the largest value allowed for the given dataset (3 times the number of profiles); all other parameters were set to default values. Genes with membership values less than 0.2 were removed from the respective clusters. We define “centrality” as the average membership value of a subset *S_C_* of a cluster *C* normalized by the average of all memberships for cluster *C.*

### Genome-wide chIP-chip analysis.

Haploid W303-derived strains harboring either *rpn4* or *pdr1* tagged with the cMyc epitope were obtained from the laboratory of Dr. Richard A. Young at the Whitehead Institute for Biomedical Research (Cambridge, Massachusetts, United States). Cells were grown to log-phase in YPD media at 30 °C, then treated with 0.03% MMS for 1 h. Protein-DNA binding locations were assayed using a chIP-chip protocol previously described [10] with corresponding IP-enriched and unenriched samples cohybridized to a single cDNA microarray containing all yeast intergenic sequences derived from PCR amplification. Microarray data were analyzed using the VERA error-modeling package [47] to generate *p*-values of TF binding for each promoter region. Unlike for gene expression analysis, in which both increases and decreases in fluorescent intensity are of interest, DNA binding is indicated for increases only, representing increased promoter binding in the IP-enriched versus IP-unenriched sample. We therefore modified the VERA likelihood ratio test to use a one-sided statistic by forcing *μ_x_* > *μ_y_* in the denominator of Equation 5 of Ideker et al. [47]. To derive *p*-values from the log-likelihood ratio statistic, we indexed values on the cumulative distribution for a negative control experiment: IP-unenriched versus IP-unenriched over three replicate microarrays.

## Supporting Information

Supplementary data files can also be downloaded from the accompanying Web site at http://www.fli-leibniz.de/tsb/tfb.

Figure S1 Functional Homogeneity and Coexpression of Target Sets Using Annotations Based on Munich Information Center for Protein Sequences and SGD (A) Using selections at specificity = 0.995 (i.e., LLS > 5). (B) Using selections at specificity = 0.997 (i.e., LLS > 6). See Figure 3B for more details.
(571 KB PNG)

Figure S2 Average LLS versus Binding *p*-Value under MMS Stress Sliding window average of LLS is shown. Horizontal lines are average LLSs over all genes. LLSs were determined without MMS binding data, but using all data from Harbison et al. [11]. LLSs are significantly increasing with decreasing binding *p*-values (Pdr1p: *p* = 2 × 10^−8^; Rpn4p: *p*=3× 10^−25^; two-sided *t* test for difference of the correlation coefficient from zero).
(218 KB PNG)

Figure S3 MCC [36] for the Scenarios Shown in Figure 3A (240 KB PNG)

Table S1 All TF–Target Interactions with LLS > 4 First column contains TF name, second column contains target gene, and columns 3 to 11 contain the respective evidences expressed as LLS. The last column contains the sum of the individual LLS. An extended table including TF–target pairs at lower thresholds can be downloaded from http://www.fli-leibniz.de/tsb/tfb.
(539 KB PDF)

Table S2 TF Modules for LLS Threshold 4 Module names are followed by module size, number of target genes, and *p*_mod_ *.* An extended table including the target genes can be downloaded from http://www.fli-leibniz.de/tsb/tfb.
(553 KB EPS)

Table S3 TF Modules for LLS Threshold 5 Module names are followed by module size, number of target genes and *p*_mod_ *.* An extended table including the target genes can be downloaded from http://www.fli-leibniz.de/tsb/tfb.
(272 KB EPS)

Table S4 Significant Overlaps (*p* < 10^−4^) between Target Gene Sets and Coexpression Clusters Target gene sets are targets of either individual TFs or TF modules. All TF–target interactions have an LLS > 5. Clusters were determined with fuzzy c-means (see Materials and Methods). Genes with membership values < 0.2 were excluded from the clusters. Significance of overlaps was determined assuming a hypergeometric distribution.
(46 KB PDF)

Table S5 Positive Control Set of TF–Target Interactions The negative control sets can be downloaded from http://www.fli-leibniz.de/tsb/tfb.
(7 KB PDF)

## Abbreviations

ChIP-chip: chromatin immunoprecipitation coupled with microarray chips
LLS: log-likelihood score
LS: likelihood score
MCC: Mathew's correlation coefficient
MMS: methyl-methanesulfonate
*p_mod_*: module *p*-value
PWM: position-specific weight matrix
ROC: receiver operator characteristic
SGD: *Saccharomyces* Genome Database
TF: transcription factor
