# Supplementary material for: Integrated Assessment and Prediction of Transcription Factor Binding
Source: PLoS Comput Biol. 2006 Jun 16;2(6):e70. doi: 10.1371/journal.pcbi.0020070 (PMC1479087; doi:10.1371/journal.pcbi.0020070)
Supplement: Supplementary file 1 [file pcbi.0020070.st001.pdf]

TF	Target	Binding_0-2000	Motif_Score_SC	Other_Yeasts	Two-Hop_BE	Two-Hop_BP	Two-Hop_BY	Two-Hop_BG	Max_Membership	Sum
ABF1	YAL016W	1.47884	1.26151	3.06892						5.80927
ABF1	YAL038W		1.09089	2.74841		0.761451				4.60075
ABF1	YAL053W	3.10342		2.81541						5.91883
ABF1	YAL054C	3.10342		2.14887	0.327443					5.57973
ABF1	YBL005W	3.00893		1.50856						4.51749
ABF1	YBL007C	3.00893		0.750906		0.445585				4.20542
ABF1	YBL038W	2.38831	0.582795	1.00721		0.480364				4.45868
ABF1	YBL039C	2.38831	0.582795	1.01716	0.805235	0.430609				5.22411
ABF1	YBL050W	0.230929	1.06449	1.97943	0.464631	0.621141				4.36062
ABF1	YBL066C	0.37531	0.319257	3.05419	0.565826					4.31458
ABF1	YBL079W	2.17705	1.20081	3.40042						6.77828
ABF1	YBL080C	2.17705		2.30366						4.48071
ABF1	YBR011C	2.60484	0.809938	0.0520559	0.566628	0.442028				4.47549
ABF1	YBR077C	2.4073		1.85049						4.25779
ABF1	YBR101C	2.96683		1.66006						4.62689
ABF1	YBR109C	2.24758	1.27292	1.50349	0.590196	0.555637				6.16982
ABF1	YBR110W	2.24758	1.27292	1.51758	0.599719					5.6378
ABF1	YBR198C	1.62339		2.03898	0.952702	0.326636				4.94171
ABF1	YBR218C		1.15277	4.1895						5.34227
ABF1	YBR283C	2.78005	1.13723	0.524487						4.44177
ABF1	YBR284W	2.78005	1.13723	0.505799	0.957031					5.38011
ABF1	YCL045C	1.32725	0.883093	2.14788						4.35822
ABF1	YCR053W	2.01694	1.36552	2.85431						6.23677
ABF1	YDL046W		1.11774	13.9249						15.0426
ABF1	YDL047W	0.33983	0.680054	7.85635		0.430609				9.30684
ABF1	YDL051W	1.47852		1.5787	0.758511	0.356874				4.1726
ABF1	YDL070W	1.50368	1.4477	2.84077						5.79215
ABF1	YDL072C	1.50368	1.4477	3.11455	0.355153					6.42108
ABF1	YDL195W	1.13234	1.68681	4.75339		0.364994				7.93753
ABF1	YDL197C	1.13234	1.68681	6.90537						9.72452
ABF1	YDR041W			4.7979						4.7979
ABF1	YDR130C	2.80677		4.72503						7.5318
ABF1	YDR194C	2.30543	1.49278	2.69038				0.994761		7.48335
ABF1	YDR195W	2.30543	1.49278	2.66365		0.492133				6.95399
ABF1	YDR222W	0.699698	1.21673	2.50366						4.42009
ABF1	YDR245W	2.95621	1.14334	0.222712		0.57076				4.89302
ABF1	YDR280W	2.83731	1.40862	3.16015						7.40608
ABF1	YDR283C	3.17191	1.13723			0.357385		1.0341		5.70063
ABF1	YDR328C	2.70753	1.22878			0.636117				4.57243
ABF1	YDR346C		1.32049	3.34482						4.66531
ABF1	YDR408C	1.36281	0.85745	3.72963						5.94989
ABF1	YDR462W	1.06836		3.38336						4.45172
ABF1	YDR463W	2.00685	1.14445	2.2685						5.4198
ABF1	YEL017W	2.07928		7.04889						9.12817
ABF1	YEL037C	2.75961	1.74254	2.43715		0.47464				7.41394
ABF1	YER007C-A	2.72917	0.445024	2.66629						5.84048
ABF1	YER119C		1.38897	6.51548						7.90445
ABF1	YER120W		1.38897	6.59807		0.344211				8.33125
ABF1	YFL037W		1.61214	6.24976						7.8619
ABF1	YFL038C		1.61214	6.63906						8.2512
ABF1	YFL047W	2.85158	1.74254							4.59412
ABF1	YFL048C	2.85158	1.74254							4.59412
ABF1	YFR031C		1.36819	2.87459		0.642521				4.8853
ABF1	YGL011C	1.17152	1.04813	1.47838	0.589947	0.344211				4.63219
ABF1	YGL022W		1.51779	3.26218						4.77997
ABF1	YGL023C		1.51779	1.59637				1.00037		4.11453
ABF1	YGL105W	2.32556	0.0219202	3.07751						5.42499
ABF1	YGL106W	2.32556	0.0219202	5.89077				1.07558		9.31383
ABF1	YGL107C	2.32556	0.0219202	3.14231						5.48979
ABF1	YGL172W	1.42312	0.981091	1.14589		0.660613				4.21071
ABF1	YGR012W			6.91096						6.91096
ABF1	YGR103W	0.682364	1.20081	1.50392	0.871145	0.470081				4.72832
ABF1	YGR185C	2.48955	0.744176	2.31071	0.35819					5.90263
ABF1	YGR202C	1.81478		2.86143						4.67621
ABF1	YGR204W	1.81478		2.80065						4.61543
ABF1	YGR231C	2.27188	1.54373	4.16348						7.97909
ABF1	YGR232W	2.27188	1.54373	4.86386		0.534743				9.21421
ABF1	YGR268C	2.52056	0.929997	2.34948						5.80004
ABF1	YGR270W	2.52056	0.929997	2.31118						5.76174
ABF1	YHL002W	0.914681	0.651051	3.00727						4.573
ABF1	YHL003C	0.914681	0.651051	2.96873						4.53446
ABF1	YHR019C	1.06078	1.52455	0.858574	0.596806					4.04071
ABF1	YHR041C	1.34102	1.0113	4.04516						6.39748
ABF1	YHR042W	1.34102	1.0113	1.73463						4.08695
ABF1	YHR064C	2.32038	0.568062	2.20227						5.09071
ABF1	YHR071W		1.11941	3.1611						4.28051
ABF1	YHR072W-A		0.964493	8.10384		0.541902				9.61023
ABF1	YHR115C	2.29435		3.33144						5.62579
ABF1	YHR116W	2.29435	1.04813	2.87979						6.22227
ABF1	YHR117W	2.29435	1.04813	3.19074				0.984143		7.51736
ABF1	YHR199C	2.59102	0.58586	3.64903						6.82591
ABF1	YHR200W	2.59102		3.63256		0.44859				6.67217
ABF1	YIL035C	1.65624	0.287697	1.18949				1.0341		4.16753
ABF1	YIL128W		1.50999	6.19992						7.70991
ABF1	YIL129C		1.50999	3.36545						4.87544
ABF1	YIL134W	1.98738	1.68681	6.65115						10.3253
ABF1	YIL135C	1.98738	1.68681	6.49294						10.1671
ABF1	YJL003W	1.39956		4.05246						5.45202
ABF1	YJL053W	0.932671		5.17096						6.10363
ABF1	YJL055W	0.257466	1.11941	4.54278						5.91966
ABF1	YJL062W	2.05689		2.78994						4.84683
ABF1	YJL062W-A			5.43709						5.43709
ABF1	YJL063C	2.05689		5.41519						7.47208
ABF1	YJL201W	2.96654	1.11104	2.67987		0.457027				7.21448
ABF1	YJL204C	2.96654	1.11104			0.566194				4.64377
ABF1	YJR047C	0.112472		4.80525	0.609887					5.52761
ABF1	YJR048W	0.112472		4.82596						4.93843
ABF1	YJR062C	2.03106	1.13723	2.81154						5.97983
ABF1	YJR064W	2.03106	1.13723	1.80901						4.9773
ABF1	YJR119C	0.251531	0.422364	4.18104				1.08921		5.94415
ABF1	YJR121W	0.251531		4.19946	0.351739	0.390318				5.19305
ABF1	YKL017C	2.05602		5.38997						7.44599
ABF1	YKL028W	2.39178	1.61214	2.9688		0.381506				7.35423
ABF1	YKL029C	2.39178	1.61214	2.96416						6.96808
ABF1	YKL048C	1.04949	0.719093	1.40583				1.0341		4.20851
ABF1	YKL172W	2.86623	1.53233	3.65602	0.725048	0.470081				9.24971
ABF1	YKL175W		1.45138	4.1941		0.442028				6.08751
ABF1	YKL176C		1.45138	4.24267						5.69405
ABF1	YKL182W	1.30418	1.49278	0.685156		0.359848		0.909249		4.75121
ABF1	YKL190W	2.63604	0.808171	0.172948				1.13067		4.74783

TF	Target	Binding_0-2000	Motif_Score_SC	Other_Yeasts	Two-Hop_BE	Two-Hop_BP	Two-Hop_BY	Two-Hop_BG	Max_Membership	Sum
ABF1	YKR029C	2.94916	0.764401	1.51753		0.542174				5.77327
ABF1	YKR030W	2.94916	0.764401	3.35239						7.06595
ABF1	YKR059W	2.16062	1.36819	1.96854	0.568899	0.445661		0.994413		7.50632
ABF1	YKR060W	2.16062	1.36819		0.693017					4.22183
ABF1	YKR072C		1.65087	8.97949						10.6304
ABF1	YKR081C	3.05728			0.79861	0.485143				4.34103
ABF1	YLL033W	3.11308	1.57521	0.0318724						4.72016
ABF1	YLL034C	3.11308	1.57521	0.0296683	0.727329	0.470081				5.91537
ABF1	YLR095C	2.83365	1.41973	3.8781						8.13148
ABF1	YLR096W	2.83365	1.41973	1.50211				1.0341		6.78959
ABF1	YLR175W	2.30169	0.476972		0.746067	0.761451				4.28618
ABF1	YLR178C	1.91156	1.61214		0.584038	0.526335				4.63407
ABF1	YLR179C	1.91156	1.61214	1.64555						5.16925
ABF1	YLR180W	1.91156	1.61214	1.69522						5.21892
ABF1	YLR257W	0.611339	0.695101	2.41294	0.497011					4.21639
ABF1	YLR396C	2.35449	0.991939	1.44355						4.78998
ABF1	YML013W		1.19641	3.44178						4.63819
ABF1	YMR197C	2.21615	1.22987	1.08458		0.621141				5.15174
ABF1	YMR198W	2.21615	1.22987	1.07873				0.972727		5.49748
ABF1	YMR216C	1.81395	1.90751	3.96731						7.68877
ABF1	YMR217W	1.81395	1.90751	2.92791	0.723512					7.37288
ABF1	YMR235C	1.82013	1.58241	5.78074	0.408539					9.59182
ABF1	YMR237W	1.82013	1.58241	5.54288						8.94542
ABF1	YNL036W	2.71416	1.04022	2.1238						5.87818
ABF1	YNL037C	2.71416	1.04022	1.74427						5.49865
ABF1	YNL117W	3.20908	1.16053	1.5434						5.91301
ABF1	YNL118C	3.20908	1.16053	1.52078						5.89039
ABF1	YNL119W	2.94002		3.97382	0.729135					7.64298
ABF1	YNL121C	2.94002		4.03046						6.97048
ABF1	YNL189W	2.39077	1.04813	1.56685		0.526335				5.53209
ABF1	YNL243W	2.64817	0.582795	0.603641		0.340251				4.17486
ABF1	YNL244C	2.64817	0.582795	3.30332		0.320356				6.85464
ABF1	YNL245C	2.64817	0.582795	2.14883						5.37979
ABF1	YNL250W	1.67466	0.90051	1.26683				1.06213		4.90413
ABF1	YNL251C	1.67466	0.90051	2.6334						5.20857
ABF1	YNL321W	2.45163	1.68681	5.99854						10.137
ABF1	YNL322C	2.45163	1.68681	7.37207						11.5105
ABF1	YNR035C	2.66843	1.50218			0.442028				4.61264
ABF1	YNR036C	2.66843	1.50218	9.20693						13.3775
ABF1	YNR037C	2.66843	1.50218	10.559						14.7296
ABF1	YNR038W	2.66843	1.50218	10.788				0.995771		15.9544
ABF1	YNR047W	2.57834	0.460707	0.037887				1.0341		4.11103
ABF1	YOL058W	0.843898	0.99935	2.84904						4.69229
ABF1	YOL068C	2.61855	1.40862	4.07518		0.631425				8.73377
ABF1	YOL076W	2.69118	1.78039	4.11735						8.58892
ABF1	YOL077C	2.69118	1.78039	4.26124	0.783518	0.470081				9.98641
ABF1	YOL115W	2.99027	1.36819							4.35846
ABF1	YOL116W	2.99027	1.36819	4.72321						9.08167
ABF1	YOR056C	2.36169	1.36819		0.802229					4.53211
ABF1	YOR057W	2.36169	1.36819					0.972011		4.70189
ABF1	YOR098C	1.60896		2.06786		0.480685				4.15751
ABF1	YOR099W	1.60896		2.793						4.40196
ABF1	YOR117W	2.36838	0.439368	1.36404		0.580257				4.75205
ABF1	YOR167C	1.34864	1.05265	1.45185	0.803004					4.65614
ABF1	YOR168W	1.34864	1.05265	1.46774	0.396347					4.26538
ABF1	YOR231W	1.01471	1.74254	1.72335				1.0341		5.5147
ABF1	YOR327C		1.36819	3.65382						5.02201
ABF1	YOR337W		1.54373	7.42489						8.96862
ABF1	YPL050C		1.08136	3.54698						4.62834
ABF1	YPL159C	2.4452	1.51779	0.322637	0.475028					4.76065
ABF1	YPL243W	0.77967	0.839899	3.14504						4.76461
ABF1	YPL244C	0.77967	0.839899	3.34682						4.96639
ABF1	YPR128C	2.13445	1.07518	0.875721						4.08535
ABF1	YPR129W	2.13445	1.07518	1.07937						4.289
ABF1	YPR176C	2.63982		1.84266						4.48248
ABF1	YPR178W	2.63982		1.88909		0.501024				5.02993
ACE2	TM(CAU)J3	7.21147	0.508741							7.72021
ACE2	YAL062W	6.15838								6.15838
ACE2	YBL014C			4.12881						4.12881
ACE2	YBL023C	3.83358			2.45703		2.11755			8.40816
ACE2	YBL026W					4.11544			0.48603	4.60147
ACE2	YBL027W				1.79826		2.10348		0.731102	4.63284
ACE2	YBL038W		0.508741	0.325568		3.9249	2.11647			6.87568
ACE2	YBL040C		0.508741	2.25962	1.33514		1.09479			5.19829
ACE2	YBL072C				2.3079		2.03851		0.72958	5.07599
ACE2	YBL092W			0.51042	0.813549		2.06621		0.732408	4.12259
ACE2	YBL111C				1.17497			2.89316		4.06813
ACE2	YBR031W			1.4912	2.29191		2.02237		0.731372	6.53685
ACE2	YBR046C			4.68437						4.68437
ACE2	YBR048W				2.26963		2.11158		0.732681	5.11389
ACE2	YBR057C	4.86124						0.992088		5.85333
ACE2	YBR109C				2.43801	2.09326	1.09479			5.62606
ACE2	YBR126C				2.97017	2.13984				5.11001
ACE2	YBR143C		0.508741		0.864542		2.1033		0.725707	4.20229
ACE2	YBR154C			1.11173	1.04687	3.33393	2.02463		0.711746	8.22891
ACE2	YBR157C	4.35513	0.508741							4.86387
ACE2	YBR158W	15.8551	0.508741	1.67658	3.23891		1.59973		0.722592	23.6017
ACE2	YBR162C	1.1911	0.508741	1.2819	2.51916			1.37537	0.727658	7.60393
ACE2	YBR162W-A	1.1911	0.508741	1.72809					0.728066	4.156
ACE2	YBR181C				2.48334		2.02264		0.730251	5.23623
ACE2	YBR189W				2.28946		2.0775		0.732326	5.09929
ACE2	YBR191W				2.29071		2.08785		0.725883	5.10444
ACE2	YBR196C		0.508741	3.49041		1.64897				5.64812
ACE2	YBR230C	3.4941			2.80548					6.29958
ACE2	YBR247C		0.508741	2.9767	1.0707				0.728441	5.28458
ACE2	YCL059C		0.508741	0.00168023	1.22449	1.42428	0.947681		0.730058	4.83693
ACE2	YCR031C				1.56081		2.12126		0.730243	4.41231
ACE2	YCR032W						1.06587	3.4065		4.47237
ACE2	YCR077C			0.726053		4.11544				4.84149
ACE2	YDL010W				2.55031		1.73886			4.28917
ACE2	YDL038C						1.53361	2.41169	0.726845	4.67215
ACE2	YDL042C	0.951521				3.33196			0.627584	4.91106
ACE2	YDL047W		0.508741	0.311302		3.9249	1.9931			6.73804
ACE2	YDL061C				2.36468		2.00915		0.731842	5.10567
ACE2	YDL083C				1.45521		2.10797		0.727734	4.29091
ACE2	YDL127W	3.4046	0.508741	0.197057					0.661532	4.77193
ACE2	YDL140C				2.44987	4.11544				6.56531
ACE2	YDL153C			1.42407	1.41269	2.14215	1.99744		0.732155	7.7085
ACE2	YDL179W	0.579533			3.30242				0.690764	4.57272
ACE2	YDL199C			2.32662	2.91346					5.24008

TF	Target	Binding_0-2000	Motif_Score_SC	Other_Yeasts	Two-Hop_BE	Two-Hop_BP	Two-Hop_BY	Two-Hop_BG	Max_Membership	Sum
ACE2	YDL201W		0.508741		1.00815		2.06499		0.711889	4.29377
ACE2	YDR012W				1.67593		2.02237		0.732434	4.43073
ACE2	YDR025W		0.508741		1.72662		2.11158		0.732106	5.07905
ACE2	YDR064W		0.508741		1.2102		2.06457		0.712068	4.49558
ACE2	YDR074W		0.508741		2.72707	2.13984				5.37565
ACE2	YDR116C				2.43415		2.1086			4.54275
ACE2	YDR153C	5.91957	0.508741	0.301529						6.72984
ACE2	YDR155C	5.91957	0.508741	0.550722						6.97903
ACE2	YDR156W	5.91957	0.508741	0.581778						7.01009
ACE2	YDR158W	5.91957	0.508741	0.600341						7.02865
ACE2	YDR237W				2.91058		2.11019			5.02077
ACE2	YDR358W				2.71655		1.71089	2.98756		7.415
ACE2	YDR378C					4.11544				4.11544
ACE2	YDR382W				1.78002		1.54264		0.727919	4.05058
ACE2	YDR404C			2.06041			2.10354			4.16395
ACE2	YDR418W				1.45687		2.06898		0.71499	4.24084
ACE2	YDR447C				1.80403		2.0851		0.723762	4.61289
ACE2	YDR450W				1.53062		2.02481		0.72879	4.28422
ACE2	YDR473C		0.508741			4.11544				4.62418
ACE2	YDR482C			0.807511		3.67116				4.47867
ACE2	YDR500C		0.508741		2.38689		2.0238		0.716947	5.63638
ACE2	YDR524C	7.54128	0.508741	0.295487						8.34551
ACE2	YDR524C-B	7.54128								7.54128
ACE2	YDR525W-A	7.54128	0.508741	1.80871						9.85873
ACE2	YDR527W	7.54128	0.508741	0.85744					0.598939	9.5064
ACE2	YEL007W	6.04844		0.934414						6.98285
ACE2	YEL011W		0.508741	2.92571	1.40695					4.8414
ACE2	YEL026W		0.508741	3.35047	1.02744		2.10587		0.732578	7.7251
ACE2	YEL037C			4.9997						4.9997
ACE2	YEL061C	0.351873	0.508741	5.76882						6.62943
ACE2	YEL075C				1.16827			2.83947		4.00774
ACE2	YEL076C				1.20698			2.89295		4.09993
ACE2	YER029C				0.436691	4.11544			0.515337	5.06747
ACE2	YER072W			1.00045	2.54368				0.720905	4.26504
ACE2	YER078C	15.8551								15.8551
ACE2	YER079W	15.8551	0.508741	1.0154	1.22969					18.6089
ACE2	YER080W	15.8551								15.8551
ACE2	YER102W				1.94883		2.03851		0.732364	4.7197
ACE2	YER109C			2.96358			1.76589			4.72947
ACE2	YER110C				2.51501	1.11759			0.66972	4.30232
ACE2	YER112W					4.11544	1.95789			6.07333
ACE2	YER124C	15.8551	0.508741	2.32507	3.09598				0.699157	22.484
ACE2	YER125W	15.8551	0.508741	2.14071						18.5046
ACE2	YER126C	6.59195			0.78953				0.732654	8.11413
ACE2	YER127W	6.59195	0.508741		0.820824				0.732634	8.65415
ACE2	YER128W	6.59195								6.59195
ACE2	YER146W	0.610242	0.508741			4.11544	2.11859			7.35301
ACE2	YER165W				2.32896		1.94048		0.732326	5.00177
ACE2	YER172C				2.51184	4.11544	1.93329			8.56057
ACE2	YER177W	3.60621				3.33393				6.94014
ACE2	YER178W	3.60621			0.565807					4.17202
ACE2	YER188C-A	5.82711								5.82711
ACE2	YER189W	5.82711			0.947031			2.83947		9.61361
ACE2	YER190W	5.82711			0.947031		0.835638	2.68225		10.292
ACE2	YFL021W	4.10444	0.508741							4.61318
ACE2	YFL022C	4.10444	0.508741						0.711015	5.3242
ACE2	YFL039C				2.45984	4.11544	1.94048			8.51576
ACE2	YFL051C		0.508741		2.91019			1.6336		5.05253
ACE2	YFL055W	2.92238	0.508741		0.95991					4.39103
ACE2	YFL056C	2.92238	0.508741					1.0857		4.51682
ACE2	YFL057C	2.92238	0.508741		0.95991			1.0857		5.47673
ACE2	YFL064C				1.23965			2.83241		4.07206
ACE2	YFL065C				1.19382			2.89293		4.08675
ACE2	YFL066C				0.586489		1.55188	2.89316		5.03153
ACE2	YFR017C	15.8551		0.803399						16.6585
ACE2	YFR019W	15.8551					2.11997			17.9751
ACE2	YFR022W					4.11544				4.11544
ACE2	YFR031C-A	0.518977		2.47483	1.77822				0.730116	5.50214
ACE2	YGL006W-A	6.85249								6.85249
ACE2	YGL007C-A	6.85249								6.85249
ACE2	YGL028C	15.8551	0.508741	0.32012	3.33418				0.710135	20.7283
ACE2	YGL030W		0.508741		1.65346		2.05533		0.73004	4.94757
ACE2	YGL068W				2.46954		2.09809			4.56763
ACE2	YGL070C		0.508741	1.59108			2.11837		0.605167	4.82336
ACE2	YGL076C			0.299012	2.3358		2.11023		0.732293	5.47734
ACE2	YGL087C					3.33393	1.88582			5.21975
ACE2	YGL103W				2.32668		2.02169		0.723774	5.07214
ACE2	YGL135W				1.70333		1.99485		0.731883	4.43006
ACE2	YGL147C	1.17303			2.69136		2.105		0.732425	6.70182
ACE2	YGL173C			0.157853		4.11544				4.27329
ACE2	YGL227W	7.23975			1.63464					8.87439
ACE2	YGL228W	8.36427								8.36427
ACE2	YGL229C	8.36427			1.40493					9.7692
ACE2	YGL241W				2.41647		1.87141			4.28788
ACE2	YGR034W				1.47795		2.079		0.732211	4.28916
ACE2	YGR041W	12.6511			2.52896					15.1801
ACE2	YGR056W		0.508741	0.0332642	2.35763		1.98443			4.88407
ACE2	YGR085C		0.508741		1.23882		2.05161		0.730832	4.53
ACE2	YGR091W		0.508741			4.11544	2.10944			6.73362
ACE2	YGR118W				1.78703		2.12221		0.726006	4.63525
ACE2	YGR136W					3.33393	1.89541			5.22934
ACE2	YGR214W		0.508741		1.65219		2.1082		0.729189	4.99832
ACE2	YGR220C			1.28876		3.9249	2.07279			7.28645
ACE2	YGR283C	2.32112	0.508741	0.501239	0.970565		0.803736			5.1054
ACE2	YGR285C	0.199245		0.624953	1.01961	3.9249	1.00004		0.687871	7.45662
ACE2	YGR296W		0.508741		0.594216		0.842794	2.66456		4.61031
ACE2	YHL013C	2.7807			1.29309			1.36699	0.732422	6.1732
ACE2	YHL029C	2.74649	0.508741	1.60564						4.86087
ACE2	YHL033C				2.34723		2.07296		0.730565	5.15076
ACE2	YHL049C		0.508741		1.34455			2.89271		4.746
ACE2	YHR019C		0.508741	2.91912					0.732469	4.16033
ACE2	YHR064C				0.714004	3.9249			0.707454	5.34636
ACE2	YHR091C	3.07516	0.508741	0.613847						4.19775
ACE2	YHR104W		0.508741	0.629788	3.01747					4.156
ACE2	YHR106W	0.814702			3.30155					4.11625
ACE2	YHR135C		0.508741	1.06289		3.67116				5.24279
ACE2	YHR137W		0.508741	1.14431	2.49374					4.14679
ACE2	YHR141C				1.41859		2.09639		0.732431	4.24741
ACE2	YHR143W	15.8551	0.508741	3.73714	3.43618				0.710475	24.2476
ACE2	YHR147C			1.20949	2.4983		2.09391			5.8017

TF	Target	Binding_0-2000	Motif_Score_SC	Other_Yeasts	Two-Hop_BE	Two-Hop_BP	Two-Hop_BY	Two-Hop_BG	Max_Membership	Sum
ACE2	YHR148W				1.28224		2.04984		0.72882	4.0609
ACE2	YHR165C					4.11544				4.11544
ACE2	YHR170W			2.23911	1.30904				0.729747	4.2779
ACE2	YHR193C	3.70728	0.508741	4.76188					0.719576	9.69748
ACE2	YHR195W	3.95142	0.508741							4.46016
ACE2	YHR196W	3.95142			1.3602				0.732628	6.04425
ACE2	YHR200W			4.66482						4.66482
ACE2	YHR203C		0.508741	4.42987	1.88554		2.02237		0.729712	9.57623
ACE2	YHR218W				1.20609			2.89316		4.09925
ACE2	YIL005W				2.51143		1.88582			4.39725
ACE2	YIL015W	0.124702	0.508741	0.405172	0.953247		1.66836	1.59361	0.713218	5.96705
ACE2	YIL021W			0.0782318		3.33393	2.09649		0.708648	6.2173
ACE2	YIL131C			1.91233		2.55045				4.46278
ACE2	YIL133C		0.508741		2.41599		2.0664		0.731481	5.72261
ACE2	YIL148W				2.31489		1.94048	2.96926	0.72265	7.94728
ACE2	YJL063C	0.194822			3.03276		2.09755			5.32513
ACE2	YJL076W	12.9529			0.63927					13.5922
ACE2	YJL078C	12.9529		0.810258	2.97716		1.94048	1.55544	0.573929	20.8102
ACE2	YJL080C	3.47398			2.16286		1.55444		0.686557	7.87784
ACE2	YJL124C				1.23634	4.11544	2.04733		0.515411	7.91452
ACE2	YJL138C				1.12946			2.89316	0.634677	4.6573
ACE2	YJL159W	6.13416								6.13416
ACE2	YJL160C	6.13416								6.13416
ACE2	YJL177W				2.35897		2.07314		0.732323	5.16443
ACE2	YJL189W		0.508741		2.56188		1.0042		0.72745	4.80227
ACE2	YJL190C				2.24968		2.11864		0.731944	5.10026
ACE2	YJL191W				2.78981		2.12126		0.697259	5.60833
ACE2	YJR017C			3.34365			0.863941			4.20759
ACE2	YJR022W		0.508741	0.123311		4.11544				4.74749
ACE2	YJR044C	7.21147	0.508741	0.0175117						7.73772
ACE2	YJR065C				2.39616		1.94048			4.33664
ACE2	YJR084W				2.53023		1.87141			4.40164
ACE2	YJR091C			0.0748903		2.92563	1.97063			4.97115
ACE2	YJR123W				2.71425		2.09054		0.730495	5.53529
ACE2	YJR145C	7.96901	0.508741	0.0551988	0.604799		2.02237		0.7321	11.8922
ACE2	YJR147W	7.96901	0.508741							8.47775
ACE2	YJR149W	4.4273			2.54281					6.97011
ACE2	YKL009W				1.64479	1.42428	2.07316		0.732742	5.87497
ACE2	YKL020C					4.11544				4.11544
ACE2	YKL081W				2.64719		1.73886		0.716437	5.10249
ACE2	YKL084W	7.13631	0.508741	0.0915408						7.73659
ACE2	YKL113C	0.0711991		0.875538	0.767928		2.10067		0.732334	4.54767
ACE2	YKL121W				2.44615		2.00261			4.44876
ACE2	YKL142W	2.45879			3.12294					5.58173
ACE2	YKL150W	3.47886		0.654863	2.71734		1.68733			8.53839
ACE2	YKL151C	3.47886		0.33312	0.665679					4.47766
ACE2	YKL170W		0.508741		2.27318		2.11843			4.90035
ACE2	YKL172W				1.94244		1.53004		0.727602	4.20008
ACE2	YKL173W					4.11544				4.11544
ACE2	YKL180W				1.37029		2.07161		0.731451	4.17335
ACE2	YKL181W		0.508741		2.47895		0.798454		0.732745	4.51889
ACE2	YKL193C				2.66853	2.55045	1.71867			6.93765
ACE2	YKR026C			3.71175		0.583833			0.668069	4.96365
ACE2	YKR042W	5.1038								5.1038
ACE2	YKR056W			2.68498	1.22081				0.731595	4.63739
ACE2	YKR059W				0.634807			2.89316	0.731665	4.25963
ACE2	YKR081C		0.508741	2.06427	1.0888	1.42428			0.724924	5.81101
ACE2	YKR094C		0.508741		1.11996		1.94048	2.96926	0.732378	7.27082
ACE2	YLL026W				3.08126		2.00917			5.09043
ACE2	YLL039C		0.508741		0.74495		1.94048	2.97038		6.16455
ACE2	YLL045C				1.45334		2.07296		0.732387	4.25869
ACE2	YLR009W				1.44611		2.03977		0.732537	4.21842
ACE2	YLR029C				1.60959		2.06457		0.729166	4.40333
ACE2	YLR048W			0.579118	1.35439		2.10192		0.728315	4.76374
ACE2	YLR075W				2.51626		2.09649		0.726646	5.3394
ACE2	YLR120C			0.0333626	2.88784		1.67372			4.59492
ACE2	YLR167W			0.877617	1.29374		1.94048	2.97373	0.732273	7.81784
ACE2	YLR173W		0.508741	3.87215						4.38089
ACE2	YLR185W				2.885		2.0238		0.727582	5.63638
ACE2	YLR197W		0.508741	0.80783	1.35307		2.06457		0.732763	5.46697
ACE2	YLR219W		0.508741	1.34856	2.87828					4.73558
ACE2	YLR254C	0.934594	0.508741				1.96805	2.99097		6.40236
ACE2	YLR259C			0.0896658	0.930917			3.40802		4.4286
ACE2	YLR286C	15.8551	0.508741	0.605081	3.43618				0.71213	21.1172
ACE2	YLR287C	5.66783	0.508741		0.492027				0.732437	7.40104
ACE2	YLR287C-A	5.66783	0.508741		1.07487				0.566434	7.81787
ACE2	YLR289W	5.66783	0.508741							6.17657
ACE2	YLR314C			0.525221		0.651792		2.98038		4.15739
ACE2	YLR340W		0.508741		1.55648	0.583833	2.03005		0.730078	5.40918
ACE2	YLR344W				2.62903		2.079		0.687598	5.39563
ACE2	YLR367W		0.508741		0.757292		2.11864		0.729524	4.1142
ACE2	YLR388W				1.53257		2.00915		0.7297	4.27142
ACE2	YLR397C	6.8818							0.511177	7.39298
ACE2	YLR438C-A	15.8551					2.11997			17.9751
ACE2	YLR439W	15.8551								15.8551
ACE2	YLR441C				1.80756		2.10358		0.73224	4.64338
ACE2	YLR462W				1.24932			2.89288		4.1422
ACE2	YLR466W				0.531108		0.842901	2.76536		4.13937
ACE2	YML004C	2.36018			1.66597					4.02615
ACE2	YML007W	4.75009			0.759486					5.50958
ACE2	YML008C	4.75009			0.422419				0.678844	5.85135
ACE2	YML026C				1.71296		2.02481		0.724718	4.46249
ACE2	YML063W				1.73108		2.10358		0.728705	4.56337
ACE2	YML065W				2.20708		2.07063			4.27771
ACE2	YML098W		0.508741	3.57564						4.08438
ACE2	YML100W	8.53683	0.508741		2.72462					11.7702
ACE2	YML101C	8.53683	0.508741	5.2219						14.2675
ACE2	YML111W					4.11544				4.11544
ACE2	YML120C		0.508741	2.49938	2.84256					5.85068
ACE2	YML123C					3.33393			0.706758	4.04069
ACE2	YMR030W			1.77612	2.71958					4.4957
ACE2	YMR121C	0.958399	0.508741		1.72035		2.06457		0.723287	5.97535
ACE2	YMR143W				1.54349		2.10797		0.730342	4.3818
ACE2	YMR251W-A	1.84475			2.89811					4.74286
ACE2	YMR262W	3.22364			2.60913					5.83277
ACE2	YMR268C					4.11544				4.11544
ACE2	YMR275C			0.281293		4.11544				4.39673
ACE2	YMR304W		0.508741		2.74976		0.964368	1.46125		5.68412
ACE2	YMR316W				1.38486	3.44746				4.83232
ACE2	YNL002C		0.508741		1.6627	1.42428			0.732211	4.32793

TF	Target	Binding_0-2000	Motif_Score_SC	Other_Yeasts	Two-Hop_BE	Two-Hop_BP	Two-Hop_BY	Two-Hop_BG	Max_Membership	Sum
ACE2	YNL005C				2.33184		2.10657			4.43841
ACE2	YNL067W				1.1986		2.1082		0.731789	4.03859
ACE2	YNL069C				1.70395		2.09589		0.731454	4.53129
ACE2	YNL078W	2.36651	0.508741		2.77538				0.721175	6.37181
ACE2	YNL098C	2.2129					0.909706	1.75877		4.88138
ACE2	YNL147W					4.11544	2.07136			6.1868
ACE2	YNL154C	1.39756		0.191376		3.67116				5.2601
ACE2	YNL162W				1.19801		2.09639		0.7309	4.0253
ACE2	YNL178W		0.508741	1.38346	1.68477		2.09269		0.691676	6.36134
ACE2	YNL185C				2.3964	3.9249				6.3213
ACE2	YNL207W		0.508741	1.69888			2.10795		0.450312	4.76588
ACE2	YNL241C	5.72617								5.72617
ACE2	YNL243W					4.11544	1.87482			5.99026
ACE2	YNL247W			2.16667	2.48349				0.699072	5.34923
ACE2	YNL252C				2.23496		1.89161			4.12657
ACE2	YNL284C					3.9249	2.09602			6.02092
ACE2	YNL302C		0.508741		1.77056		2.02237		0.730777	5.03245
ACE2	YNL327W	5.96293	0.508741	0.499478	3.24347		0.819692		0.711625	11.7459
ACE2	YNL328C	5.96293	0.508741	0.745807						7.21748
ACE2	YNL329C	5.96293	0.508741							6.47167
ACE2	YNL339C		0.508741		0.38202		0.842794	2.66456		4.39811
ACE2	YNR019W	3.33492			0.7115					4.04642
ACE2	YNR067C			0.0930768	3.23661		0.804438		0.619425	4.75355
ACE2	YOL040C		0.508741	1.04672	1.56743		2.12126		0.728934	5.97309
ACE2	YOL060C		0.508741	1.71543	2.56274					4.78691
ACE2	YOL069W		0.508741	4.72241			0.846398	1.36699		7.44454
ACE2	YOL073C		0.508741	3.88857						4.39731
ACE2	YOL094C		0.508741	2.84726		0.83608			0.544545	4.73663
ACE2	YOL121C		0.508741		1.18848		2.02237		0.722269	4.44186
ACE2	YOL135C		0.508741			3.9249				4.43364
ACE2	YOL149W					4.11544			0.727843	4.84328
ACE2	YOR018W					4.11544				4.11544
ACE2	YOR101W		0.508741				0.964368	1.91747	0.732502	4.12308
ACE2	YOR138C	8.6521	0.508741	0.999205						10.16
ACE2	YOR140W	8.6521								8.6521
ACE2	YOR145C				1.36071		2.06348		0.732496	4.15669
ACE2	YOR150W		0.508741	2.01926	2.58863		2.11499			7.23162
ACE2	YOR188W			2.43242	0.357297		1.6634		0.732414	5.18553
ACE2	YOR198C						1.89059	2.92463		4.81522
ACE2	YOR206W		0.508741		1.36925	1.42428			0.732745	4.03502
ACE2	YOR215C			1.25594	2.91739					4.17333
ACE2	YOR264W	0.386048	0.508741	0.369348	3.13838				0.654427	5.05694
ACE2	YOR270C			0.182949	2.65825		2.03953			4.88073
ACE2	YOR315W	1.75411			2.6244					4.37851
ACE2	YOR369C				1.5701		2.08612		0.732475	4.3887
ACE2	YOR373W	0.816967	0.508741	1.78483			1.73443			4.84497
ACE2	YPL024W	6.62599			1.93169					8.55768
ACE2	YPL026C	6.62599								6.62599
ACE2	YPL079W				1.40995		2.08785		0.732411	4.23021
ACE2	YPL081W	0.646416			1.09292		2.0775		0.700794	4.51763
ACE2	YPL090C		0.508741		1.1567		2.02264		0.732003	4.42008
ACE2	YPL093W				1.06115	1.42428	2.11033		0.7308	5.32656
ACE2	YPL097W		0.508741	1.4057			2.0928			4.00724
ACE2	YPL126W		0.508741	4.18827	0.55275	3.33196			0.718564	9.30029
ACE2	YPL131W				1.42601		2.03426		0.730645	4.19092
ACE2	YPL160W				2.52308		2.10332		0.732455	5.35886
ACE2	YPL198W				1.48573		2.11023		0.725393	4.32135
ACE2	YPL220W				1.72143		1.99485		0.730958	4.44724
ACE2	YPL230W	1.00723	0.508741	0.987837	2.62577					5.12958
ACE2	YPL237W		0.508741		0.677598		2.0966		0.731698	4.01464
ACE2	YPR010C		0.508741	0.423753		3.33196			0.7171	4.98155
ACE2	YPR016C				0.63492	1.42428	2.02252		0.732129	4.81385
ACE2	YPR043W				1.33405		2.07895		0.727283	4.14028
ACE2	YPR102C				1.84626		2.05161		0.730832	4.6287
ACE2	YPR106W	1.31002			2.51624		0.961661		0.680713	5.46863
ACE2	YPR119W			0.177622	2.2412	0.441847	1.94048		0.613261	5.41441
ACE2	YPR132W				1.75557		2.12221		0.732361	4.61014
ACE2	YPR178W		0.508741			4.11544				4.62418
ACE2	YPR184W				2.34356	3.7382				6.08176
ACE2	YPR187W		0.508741		1.1358	3.33393	2.10391		0.730944	7.81332
ACE2	YPR204W				0.890477		0.846429	2.75636		4.49327
ADR1	YBR126C			5.29042						5.29042
ADR1	YBR128C			6.77423						6.77423
ADR1	YBR255C-A	4.72022								4.72022
ADR1	YBR256C	4.72022								4.72022
ADR1	YBR257W	4.72022								4.72022
ADR1	YBR297W			5.23975						5.23975
ADR1	YDL240W	4.33732								4.33732
ADR1	YDR379W	0.514044		3.66918						4.18322
ADR1	YDR380W			4.88019						4.88019
ADR1	YFR008W	3.99063		0.326112						4.31674
ADR1	YGL222C	0.940644		3.38516						4.3258
ADR1	YIL034C			5.48641						5.48641
ADR1	YIL157C			4.62683						4.62683
ADR1	YJL023C	4.25084								4.25084
ADR1	YJR052W	2.57712		3.67761						6.25473
ADR1	YLR064W	3.02326		1.09653						4.11979
ADR1	YLR129W	0.405873		3.276	0.58082					4.26269
ADR1	YNL071W			4.13821						4.13821
ADR1	YPL024W			4.28834						4.28834
ADR1	YPL026C			6.67657						6.67657
AFT2	YAL035W			4.35526	1.14169					5.49695
AFT2	YBR077C	4.12405								4.12405
AFT2	YBR078W	4.12405								4.12405
AFT2	YBR103W	3.80552					1.31043			5.11595
AFT2	YBR191W	1.40464			1.59219		1.05198			4.04881
AFT2	YBR195C			2.26697	0.877642		1.31856			4.46317
AFT2	YBR204C	5.71077			1.05819					6.76896
AFT2	YBR207W	5.71077		0.939484						6.65025
AFT2	YBR247C			0.742359	1.867	2.12613				4.73549
AFT2	YCR057C				0.915902	2.12613	1.30629			4.34832
AFT2	YCR105W	4.58348		0.411018						4.9945
AFT2	YCR107W	5.75252			0.535826					6.28835
AFT2	YDL014W				1.35578	2.31667	1.02152			4.69397
AFT2	YDL022W	1.63458		2.76486	1.28455					5.68399
AFT2	YDL051W			2.50715	1.65548					4.16263
AFT2	YDR151C	4.9574								4.9574
AFT2	YDR152W	4.9574			0.551449					5.50885
AFT2	YDR324C				2.1053	1.29761	1.25874			4.66165
AFT2	YDR422C	10.8768								10.8768

TF	Target	Binding_0-2000	Motif_Score_SC	Other_Yeasts	Two-Hop_BE	Two-Hop_BP	Two-Hop_BY	Two-Hop_BG	Max_Membership	Sum
AFT2	YDR441C	2.00023		2.22689						4.22712
AFT2	YDR534C	4.87423		0.740882	0.356774					5.97189
AFT2	YEL058W	4.10288		2.47429						6.57717
AFT2	YEL060C	4.10288		1.05195						5.15483
AFT2	YER027C			2.63332	1.80026					4.43358
AFT2	YER033C			4.66565	0.69761					5.36326
AFT2	YER035W			4.42709	0.557451					4.98454
AFT2	YER048W-A	5.88215								5.88215
AFT2	YER049W	5.88215			1.01798					6.90013
AFT2	YER133W	3.92019				0.908069	0.992845			5.8211
AFT2	YER145C	3.14823		2.35872						5.50695
AFT2	YER146W	3.14823		1.31146		1.80112	1.11205			7.37286
AFT2	YFL041W	1.97228		1.87782	1.23362	1.1864				6.27012
AFT2	YFR017C	5.20862								5.20862
AFT2	YFR019W	5.20862					1.11342			6.32204
AFT2	YFR024C-A	3.01309			2.43837			1.03794		6.4894
AFT2	YGL261C	3.63969			0.744775					4.38446
AFT2	YGR220C	0.263939		2.52142		1.61058	1.06624			5.46218
AFT2	YHL013C	1.54566			1.41499		1.08913			4.04978
AFT2	YHL046C	2.69544			1.38724					4.08268
AFT2	YHR007C-A	5.59246								5.59246
AFT2	YHR008C	5.59246								5.59246
AFT2	YHR033W	4.78341								4.78341
AFT2	YHR115C	2.71026		0.328063			1.24119			4.27951
AFT2	YHR117W	2.71026		0.314302				0.976036		4.0006
AFT2	YHR148W	1.39634		0.160439	1.46774		1.0082			4.03272
AFT2	YHR195W	4.25164								4.25164
AFT2	YHR196W	4.25164			2.18216					6.4338
AFT2	YIL004C	5.52585			0.582127					6.10798
AFT2	YIR034C			2.74134	1.77353					4.51487
AFT2	YIR041W	3.97435			0.544995					4.51934
AFT2	YJL068C	6.12275			0.675276					6.79803
AFT2	YJL069C	6.12275		0.0919955	1.31295		1.20882			8.73652
AFT2	YJR063W			0.857241	1.20549	1.49904	1.10087			4.66264
AFT2	YJR143C	5.76074								5.76074
AFT2	YKL009W	1.14874			1.88677		1.09291			4.12842
AFT2	YKL029C			3.70025	0.907011					4.60726
AFT2	YKL054C	3.81335		0.276349						4.0897
AFT2	YKL085W	0.305793		3.4698		0.345899				4.12149
AFT2	YKL101W	7.16909								7.16909
AFT2	YKL103C	7.16909			0.76029					7.92938
AFT2	YKL144C			1.0135	1.25132	1.49904	1.08417			4.84803
AFT2	YKR052C	11.7005		0.548636	0.663749					12.9129
AFT2	YLL008W	2.98529			1.74559					4.73088
AFT2	YLL011W	9.19183			1.17302		0.848485			11.2133
AFT2	YLR020C	0.528963		2.82585			1.09382			4.44863
AFT2	YLR034C	4.97498		2.47289	0.767657					8.21553
AFT2	YLR051C	2.86994			1.60851					4.47845
AFT2	YLR120C	4.60867			1.04804					5.65671
AFT2	YLR129W				2.15769	1.29761	1.284			4.7393
AFT2	YLR136C	7.44268		1.61562						9.0583
AFT2	YLR137W	7.44268		0.188448						7.63113
AFT2	YLR138W	7.44268								7.44268
AFT2	YLR206W	3.50737			0.602631					4.11
AFT2	YLR222C	0.673645			2.05556	1.29761	1.30629			5.3331
AFT2	YLR357W			3.01452			1.04573	0.912341		4.97259
AFT2	YLR377C			4.14479	1.07573					5.22052
AFT2	YLR397C	5.78756			0.365333					6.15289
AFT2	YLR409C				1.76117	1.29761	1.3376			4.39638
AFT2	YLR438C-A	7.28209				0.685163	0.869981			8.83723
AFT2	YLR439W	7.28209								7.28209
AFT2	YLR460C	3.40668			0.903505					4.31019
AFT2	YLR461W	3.40668			1.05937					4.46605
AFT2	YML092C	1.35075			1.65869		1.10681			4.11625
AFT2	YMR034C	3.74149		3.276			0.926811			7.9443
AFT2	YMR035W	3.74149		1.00439						4.74588
AFT2	YMR041C	3.82454		1.70398	0.575601		0.937074			7.0412
AFT2	YMR042W	3.82454		1.33131		0.867734	0.996745			7.02033
AFT2	YMR043W	3.82454				0.867734	1.04573			5.738
AFT2	YMR055C	4.42127								4.42127
AFT2	YMR058W	4.42127		0.820317						5.24159
AFT2	YMR146C			2.17013	1.03967		1.31856			4.52836
AFT2	YMR325W	3.54519			1.09657					4.64176
AFT2	YNL208W			4.14622	1.3482					5.49442
AFT2	YNL241C	8.23955		1.31345						9.553
AFT2	YNL259C	2.01565		2.33042		0.5709				4.91697
AFT2	YNR026C	5.29702								5.29702
AFT2	YNR028W	5.29702					0.815813			6.11283
AFT2	YOR224C	6.8689								6.8689
AFT2	YOR226C	6.8689		2.03207						8.90097
AFT2	YOR227W	6.8689		0.975739						7.84464
AFT2	YOR383C	4.11788			0.511278					4.62916
AFT2	YOR384W	4.11788								4.11788
AFT2	YPL034W	4.36958		1.5138						5.88338
AFT2	YPL135W	2.93246		1.7916						4.72406
AFT2	YPL137C	2.93246		1.96866						4.90112
AFT2	YPL253C	4.11966		0.0682031						4.18786
AFT2	YPL263C			2.25118	1.15593			1.23308		4.64019
AFT2	YPR027C			4.34166						4.34166
AFT2	YPR040W	1.42384		5.27968						6.70352
AFT2	YPR133C			3.23001			1.22444			4.45445
AFT2	YPR141C	0.16039		1.42933		0.947405	1.24544	0.92949		4.71206
AFT2	YPR181C	1.54603			1.14861	1.32701				4.02165
ARG80	SNR37	5.22879								5.22879
ARG80	TK(CUU)C	4.2786								4.2786
ARG80	YCR019W	4.2786								4.2786
ARG80	YER069W	5.87653								5.87653
ARG80	YFL023W	8.28635								8.28635
ARG80	YFL024C	8.28635								8.28635
ARG80	YOL140W	6.1079								6.1079
ARG81	YBR247C				2.36156		1.72901			4.09057
ARG81	YER069W	10.4445			0.722554		0.967391		0.515261	12.6497
ARG81	YFL023W	14.3482								14.3482
ARG81	YFL024C	14.3482								14.3482
ARG81	YFR057W	4.61974								4.61974
ARG81	YHR179W	4.83946								4.83946
ARG81	YHR196W	3.44549			2.37631					5.8218
ARG81	YJL088W	8.15371			1.57699		1.23635	1.40152		12.3686
ARG81	YJR109C				1.3032	1.57938	1.24145	1.62584		5.74987
ARG81	YKL106W	7.42292								7.42292

TF	Target	Binding_0-2000	Motif_Score_SC	Other_Yeasts	Two-Hop_BE	Two-Hop_BP	Two-Hop_BY	Two-Hop_BG	Max_Membership	Sum
ARG81	YKL107W	7.42292						0.878023		8.30094
ARG81	YLR243W				2.36841	2.51882				4.88723
ARG81	YML093W				2.3655		1.79389			4.15939
ARG81	YML098W	6.72425								6.72425
ARG81	YML099C	6.72425								6.72425
ARG81	YOL058W	5.21266			0.682767		1.26646			7.16189
ARG81	YOL140W	12.4222			0.705051					13.1273
ARG81	YOR302W	6.46071			0.833919					7.29463
ARG81	YOR303W	6.46071			0.833919		0.988003	1.7066		9.98923
ARG81	YPL265W	8.32897								8.32897
ARO80	SNR32	4.70133								4.70133
ARO80	YBL078C	0.335593			2.02494	1.85106				4.21159
ARO80	YDR373W	0.817014						3.24428		4.06129
ARO80	YDR378C	4.67295								4.67295
ARO80	YDR379W	4.67295								4.67295
ARO80	YDR380W	14.6508			2.78359					17.4344
ARO80	YDR381W	5.84401								5.84401
ARO80	YDR382W	5.07072								5.07072
ARO80	YDR421W	9.13502								9.13502
ARO80	YDR469W	1.18373			2.83888					4.02261
ARO80	YEL034W	0.605849			0.722595	3.07647				4.40491
ARO80	YER079W				2.29892	3.4137				5.71262
ARO80	YGL202W	2.20579			2.21858					4.42437
ARO80	YGR119C				2.76507		1.8914			4.65647
ARO80	YGR154C				2.21383	3.07647				5.2903
ARO80	YHR089C	0.446144			0.463774	3.40367				4.31359
ARO80	YHR135C	14.9015								14.9015
ARO80	YHR136C	14.9015								14.9015
ARO80	YHR137W	14.9015			2.7324					17.6339
ARO80	YHR138C	4.36105			2.43448					6.79553
ARO80	YHR139C	5.57453								5.57453
ARO80	YHR140W	5.57453								5.57453
ARO80	YIL004C	7.46716								7.46716
ARO80	YJL138C				1.02287	3.07647				4.09934
ARO80	YKL027W	0.206455			2.00321		1.86451			4.07418
ARO80	YNL124W	13.2185								13.2185
ARO80	YNL125C	13.2185			0.625145					13.8436
ARO80	YNL139C	1.87493				3.07647				4.9514
ARO80	YOR177C					3.07647	0.803925	1.41111		5.2915
ARO80	YPL019C	1.74746			2.35412					4.10158
ARO80	YPL253C	4.72962								4.72962
ARO80	YPR048W				2.31311		1.7415			4.05461
ARR1	YDR101C	1.17578		2.08934	1.27103					4.53615
ARR1	YGL001C	0.279536		3.63474	0.33763					4.25191
ARR1	YGL189C			5.05069	0.498284					5.54897
ARR1	YGR109C			4.09924						4.09924
ARR1	YGR110W			4.69671						4.69671
ARR1	YGR117C	2.2684		2.08966						4.35806
ARR1	YHR170W			3.65692	0.546781					4.2037
ARR1	YJR138W			4.12547						4.12547
ARR1	YJR143C	6.11913								6.11913
ARR1	YKL001C	3.39668		0.803342						4.20002
ARR1	YKL069W	0.0764326		3.99664						4.07307
ARR1	YKL071W			4.24357						4.24357
ARR1	YKL107W	0.634429		4.34166						4.97609
ARR1	YNL331C	0.0895513		4.07633						4.16588
ARR1	YOL048C			4.85554						4.85554
ARR1	YOR001W	5.01094			0.657388					5.66833
ASH1	YDR077W	3.90955			0.424256					4.33381
ASH1	YER092W	1.84037		2.19469						4.03506
ASH1	YGR189C	2.88193		2.71761	0.44569					6.04523
ASH1	YGR191W			4.34166						4.34166
ASH1	YIL122W	4.66816								4.66816
ASH1	YIL140W	3.95375			0.462237					4.41599
ASH1	YKL060C			4.26368						4.26368
ASH1	YKL103C	0.816503		3.2317	0.343662					4.39187
ASH1	YNL300W	3.867			0.446009					4.31301
ASH1	YNL301C	3.867			0.464009					4.33101
ASK10	SNR37	4.42612								4.42612
ASK10	TW(CCA)J	4.36158								4.36158
ASK10	YCR096C	3.35718				0.741572				4.09875
ASK10	YCR097W	3.35718				0.741572				4.09875
ASK10	YDR084C	4.15474								4.15474
ASK10	YGL001C	5.22397			0.452168					5.67614
ASK10	YGR071C	5.9848								5.9848
ASK10	YGR072W	5.9848								5.9848
ASK10	YGR074W	5.9848								5.9848
ASK10	YJR010C-A	4.23584								4.23584
ASK10	YJR011C	4.23584								4.23584
ASK10	YJR013W	4.23584								4.23584
ASK10	YOR001W	5.67188			0.544469					6.21635
AZF1	SNR13	2.3783	2.43011							4.80841
AZF1	SNR18	2.69783	1.65442							4.35225
AZF1	TH(GUG)E1	1.69603	2.41549							4.11152
AZF1	YAL002W		1.65442	3.2313						4.88572
AZF1	YAL003W	2.69783		1.54398						4.24181
AZF1	YAL005C	2.69783	1.65442	3.25869						7.61094
AZF1	YAL036C		2.01948	2.68282	0.447411					5.14971
AZF1	YAL038W		1.44296	5.81537						7.25833
AZF1	YAL039C	2.43604	1.44296	1.31565						5.19465
AZF1	YBL029W	2.20243	1.45874	0.652297						4.31347
AZF1	YBR072W		1.39588	3.08769						4.48357
AZF1	YBR077C		2.71747	4.6327						7.35017
AZF1	YBR114W	0.0558303	1.57675	6.05689						7.68947
AZF1	YCL001W		2.68879	2.70764						5.39643
AZF1	YCL063W		1.48756	5.00082						6.48838
AZF1	YCL064C		1.48756	5.24736						6.73492
AZF1	YCR008W		1.37246	3.4286						4.80106
AZF1	YCR024C	0.229177	1.36873	3.4376						5.03551
AZF1	YDL035C		2.16046	1.85216						4.01262
AZF1	YDL037C	3.97632	2.45396							6.43028
AZF1	YDL047W		1.86121	2.77829						4.6395
AZF1	YDL048C		1.86121	2.37701	0.596165					4.83439
AZF1	YDL107W			4.88155						4.88155
AZF1	YDL170W	0.300413	2.37541	9.85248						12.5283
AZF1	YDL171C	0.300413	2.37541	2.7326						5.40842
AZF1	YDL229W		2.16324	3.63437						5.79761
AZF1	YDR041W	0.0505899		6.98939						7.03998
AZF1	YDR042C	2.22077	2.83612							5.05689
AZF1	YDR051C		1.41603	2.71407						4.1301

TF	Target	Binding_0-2000	Motif_Score_SC	Other_Yeasts	Two-Hop_BE	Two-Hop_BP	Two-Hop_BY	Two-Hop_BG	Max_Membership	Sum
AZF1	YDR441C	1.74014	1.56697	1.57979						4.8869
AZF1	YDR470C	2.3783	2.43011							4.80841
AZF1	YDR472W	2.3783	2.43011							4.80841
AZF1	YDR524C	1.73019	2.16046	1.99605						5.8867
AZF1	YEL021W		1.59371	2.43176						4.02547
AZF1	YEL047C	0.950043		11.5061						12.4561
AZF1	YEL056W		1.64121	2.49681						4.13802
AZF1	YEL057C		1.64121	2.49029						4.1315
AZF1	YER037W		1.72052	4.52133						6.24185
AZF1	YFL031W	0.404654		4.39107						4.79572
AZF1	YFL033C	0.404654	1.24898	4.07935						5.73298
AZF1	YGL009C	0.432348		4.58712						5.01947
AZF1	YGL056C		2.00041	4.46287						6.46328
AZF1	YGL126W		2.3584	6.50681						8.86521
AZF1	YGL127C		2.3584	5.85902						8.21742
AZF1	YGL128C		2.3584	6.00952						8.36792
AZF1	YGL223C			6.53558						6.53558
AZF1	YGL247W		2.56482	1.97523						4.54005
AZF1	YGR043C			5.33108						5.33108
AZF1	YGR055W	0.53799	3.05355	3.46182						7.05336
AZF1	YGR152C		1.29999	2.49593				0.870441		4.66636
AZF1	YGR178C		2.17622	3.19758						5.3738
AZF1	YGR260W		1.89001	2.97615						4.86616
AZF1	YHL032C		1.48129	4.43039						5.91168
AZF1	YHR069C		2.38211	1.71725		0.563576				4.66294
AZF1	YHR161C		2.12694	7.48032						9.60726
AZF1	YHR162W		2.12694	6.21629						8.34323
AZF1	YHR163W		2.12694	6.23435						8.36129
AZF1	YHR207C		2.85996	2.74338						5.60334
AZF1	YHR208W		2.85996	2.6396						5.49956
AZF1	YIR019C	3.42077	2.18455							5.60532
AZF1	YJL128C		1.69135	3.60741						5.29876
AZF1	YKL110C		1.84357	3.525						5.36857
AZF1	YKR097W	0.611942	2.27715	2.72163						5.61072
AZF1	YLL019C	3.18511	2.52448							5.70959
AZF1	YLR016C		2.52448	2.31719						4.84167
AZF1	YLR017W		2.52448	2.39605						4.92053
AZF1	YLR023C	2.80791	2.46761	2.40262						7.67814
AZF1	YLR249W		2.52448	1.24618	0.390599					4.16126
AZF1	YLR287C	0.0703692	2.40261	1.61042						4.0834
AZF1	YML007W	2.50076	1.51831							4.01907
AZF1	YML008C	2.50076	1.51831		0.344802					4.36387
AZF1	YMR220W		2.63696	7.23303	0.480096					10.3501
AZF1	YMR243C		2.85996	8.98347						11.8434
AZF1	YMR244W		2.85996	4.76911						7.62907
AZF1	YMR265C		2.15071	2.66511						4.81582
AZF1	YMR266W		2.15071	2.58965						4.74036
AZF1	YNL184C		2.61602	1.77744						4.39346
AZF1	YNL185C		2.61602	2.99622						5.61224
AZF1	YNL245C		2.54056	4.19917						6.73973
AZF1	YNL287W		1.30755	2.83408						4.14163
AZF1	YNL327W		2.26486	1.01044				1.00704		4.28234
AZF1	YNR050C	2.03206	2.2644							4.29646
AZF1	YNR051C			5.38831						5.38831
AZF1	YOR065W	1.96647	2.12601							4.09248
AZF1	YOR152C	0.0799864		4.60399						4.68398
AZF1	YOR204W		2.26486	2.44055						4.70541
AZF1	YOR291W		1.80021	2.94322						4.74343
AZF1	YOR355W	0.478222		4.097						4.57522
AZF1	YPL014W		2.11994	3.32949						5.44943
AZF1	YPL015C		2.11994	4.24912						6.36906
AZF1	YPL106C		1.66659	3.71006						5.37665
AZF1	YPL117C		1.56594	2.65718						4.22312
AZF1	YPR156C		2.10074	4.04367						6.14441
AZF1	YPR157W		2.10074	2.18921						4.28995
BAS1	YAL044C	15.4841					1.9414		0.483671	17.9092
BAS1	YAL046C	15.4841								15.4841
BAS1	YAL047C	15.4841								15.4841
BAS1	YAL060W	2.3766		0.00964807	2.63268					5.01893
BAS1	YAR015W	0.627837			2.36634		1.39215		0.713653	5.09998
BAS1	YBL027W				2.38202		2.05459			4.43661
BAS1	YBL038W				2.33205		2.06268			4.39473
BAS1	YBL063W			0.0658525			2.0727	2.84444		4.98299
BAS1	YBR013C	1.93191			2.50178					4.43369
BAS1	YBR031W				2.08965		1.97313			4.06278
BAS1	YBR084C-A				2.08247		2.05459			4.13706
BAS1	YBR087W	2.19213					2.0429			4.23503
BAS1	YBR143C	0.673363			2.53842		2.05079			5.26257
BAS1	YCL030C	3.49037		2.29487					0.699847	6.48509
BAS1	YCL051W			5.34835						5.34835
BAS1	YCL057C-A			4.63375						4.63375
BAS1	YCR031C	0.0824369			2.45647		2.07163			4.61054
BAS1	YDL061C				2.03229		2.00069			4.03298
BAS1	YDL083C	2.18158			1.04043		2.02753			5.24954
BAS1	YDL167C			2.93684	1.25477					4.19161
BAS1	YDL197C						1.97535	2.91997		4.89532
BAS1	YDR018C	14.1271								14.1271
BAS1	YDR019C	14.1271					2.03406		0.542524	16.7037
BAS1	YDR021W	14.1271								14.1271
BAS1	YDR025W				1.98893		2.06865			4.05758
BAS1	YDR064W	1.21859			2.43268		2.01694			5.66821
BAS1	YDR120C				2.1903		1.88855			4.07885
BAS1	YDR150W						2.07021	2.88079		4.951
BAS1	YDR158W			2.3501	2.13512					4.48522
BAS1	YDR237W	0.379587			2.92928		2.0478			5.35667
BAS1	YDR337W			2.08175	2.96318		2.06436			7.10929
BAS1	YDR393W						2.02405	2.93791		4.96196
BAS1	YDR407C	5.47334								5.47334
BAS1	YDR408C	5.47334					0.842164	0.977717		7.29322
BAS1	YDR409W	5.47334								5.47334
BAS1	YDR418W				2.57484		2.03651			4.61135
BAS1	YDR494W			1.42497	2.58279					4.00776
BAS1	YDR495C				2.34353		1.97794			4.32147
BAS1	YDR500C				2.10019		1.9034			4.00359
BAS1	YDR532C			0.00986273			1.97887	2.92897		4.9177
BAS1	YEL026W				2.02794		2.04257			4.07051
BAS1	YEL061C						2.06512	2.83303		4.89815
BAS1	YER050C			1.15458	2.7523		1.86898			5.77586
BAS1	YER055C	0.395974		3.93464						4.33061
BAS1	YER061C	3.98942		1.97213					0.632615	6.59417

TF	Target	Binding_0-2000	Motif_Score_SC	Other_Yeasts	Two-Hop_BE	Two-Hop_BP	Two-Hop_BY	Two-Hop_BG	Max_Membership	Sum
BAS1	YER074W	0.427871			2.21294		1.95128			4.59209
BAS1	YER091C	10.3488								10.3488
BAS1	YER092W	10.3488		0.166969						10.5158
BAS1	YGL076C				2.16252		2.02124			4.18376
BAS1	YGL135W	0.704081			2.02297		2.03146			4.75851
BAS1	YGL137W			0.0117152	2.41402	2.15332				4.57906
BAS1	YGL186C	4.00234								4.00234
BAS1	YGL234W	2.32312		0.266675	0.636665		0.864875	1.54538		5.63671
BAS1	YGR061C	3.68241					0.994152			4.67656
BAS1	YGR095C				2.03044		2.03935			4.06979
BAS1	YGR118W				2.65893		2.07334			4.73227
BAS1	YGR185C				2.41309		2.05844			4.47153
BAS1	YGR202C	2.06246		1.27102			2.01413			5.34761
BAS1	YGR218W	0.114112				4.0153				4.12941
BAS1	YGR285C				2.22331		1.78121			4.00452
BAS1	YHL004W					3.82476	1.9553			5.78006
BAS1	YHL047C				2.25743		1.88112			4.13855
BAS1	YHR038W				2.54308		1.9769			4.51998
BAS1	YHR172W					4.0153				4.0153
BAS1	YIL004C	8.58675								8.58675
BAS1	YIL133C				2.2292		2.03912			4.26832
BAS1	YIL138C						1.97091	2.95039		4.9213
BAS1	YJL177W				2.36941		1.99978			4.36919
BAS1	YJL208C				2.08975	2.44432				4.53407
BAS1	YJR007W				2.42	2.86744	1.991			7.27844
BAS1	YJR063W			0.356628	1.59413		2.07786			4.02862
BAS1	YJR094W-A				2.14843		2.01534			4.16377
BAS1	YJR123W				2.03896		2.04957			4.08853
BAS1	YJR145C				2.09541		1.97313			4.06854
BAS1	YKL009W				2.07027		2.02963			4.0999
BAS1	YKL170W				2.42435		2.07247			4.49682
BAS1	YKL179C	2.32281		0.0229999			2.02139	0.899294		5.26649
BAS1	YKL180W				2.04153		1.97891			4.02044
BAS1	YKR079C	10.3705		0.856182			1.96094			13.1876
BAS1	YKR080W	10.3705		0.54628	2.07123				0.721761	13.7098
BAS1	YLL026W	1.30563		3.08512	0.514706		2.04505			6.95051
BAS1	YLR028C	0.252817		1.32792	1.78199		0.884312			4.24704
BAS1	YLR045C					4.0153	1.98961	2.92421		8.92912
BAS1	YLR058C	14.6136			1.20448				0.475996	16.2941
BAS1	YLR060W	14.6136			0.426563		1.43634			16.4765
BAS1	YLR061W	5.28515			2.20739					7.49254
BAS1	YLR063W	5.28515								5.28515
BAS1	YLR172C			0.0658715	2.06072		2.03219			4.15878
BAS1	YLR197W				2.30303		1.9423			4.24533
BAS1	YLR243W				2.46459		1.97681			4.4414
BAS1	YLR359W	3.66437		0.124299	2.21498		0.917058		0.719203	7.63991
BAS1	YLR367W	0.815715			1.98588		2.0751			4.87669
BAS1	YLR378C				2.98621		2.02506			5.01127
BAS1	YLR388W				2.072		2.00069			4.07269
BAS1	YLR423C					2.25175	2.07435	2.94276		7.26886
BAS1	YLR432W			1.77496	2.37136					4.14632
BAS1	YML092C			2.01926			2.05572			4.07498
BAS1	YML093W			1.69695	2.47751			2.52444		6.6989
BAS1	YMR120C	3.50628		0.206847	0.461721		0.884312		0.706943	5.7661
BAS1	YMR121C				2.13916		2.01694			4.1561
BAS1	YMR122W-A	3.50628		0.506576						4.01286
BAS1	YMR187C	15.4841								15.4841
BAS1	YMR188C	15.4841		1.11998	0.376003		1.36055			18.3406
BAS1	YMR189W	15.4841		1.13203			1.9414		0.722465	19.28
BAS1	YMR193W	1.68768			3.19425					4.88193
BAS1	YMR260C				2.20499		1.97198			4.17697
BAS1	YMR294W						1.99605	2.96271		4.95876
BAS1	YMR300C	6.669		3.1138			0.927945		0.689719	11.4005
BAS1	YMR314W			2.62882			2.05906			4.68788
BAS1	YNL003C			7.91363						7.91363
BAS1	YNL005C			2.65407			2.02024			4.67431
BAS1	YNL009W	1.48603		3.2448	0.586799					5.31763
BAS1	YNL030W				2.3401		1.98189			4.32199
BAS1	YNL067W			1.45732	0.478451		2.06495			4.00072
BAS1	YNL069C			1.49892	2.04093		2.06284			5.60269
BAS1	YNL126W					4.0153				4.0153
BAS1	YNL188W	1.4978				4.0153				5.5131
BAS1	YNL209W	0.873684			3.23325					4.10693
BAS1	YNL220W	1.19098		2.33844			0.915133		0.601635	5.04619
BAS1	YNL225C			0.558554	2.1995		1.9424	3.29621		7.99666
BAS1	YNL288W	0.75377		3.34588						4.09965
BAS1	YNR037C				2.58404		1.82897			4.41301
BAS1	YOL039W			0.273725	2.31042		1.96718			4.55133
BAS1	YOL097C				2.46776		2.04002			4.50778
BAS1	YOL121C	1.12721			1.06931		1.97313			4.16965
BAS1	YOL124C				2.16442		1.97948			4.1439
BAS1	YOR119C				2.33707		1.95411			4.29118
BAS1	YOR128C	5.94329		0.332607			1.01315		0.683465	7.97251
BAS1	YOR191W						1.90042	2.83659		4.73701
BAS1	YOR198C	0.608947			0.451757		1.93265	2.91277		5.90612
BAS1	YOR310C				2.53311		1.9423			4.47541
BAS1	YOR369C				1.98093		2.04258			4.02351
BAS1	YPL018W	4.83444								4.83444
BAS1	YPL019C	4.83444								4.83444
BAS1	YPL022W						2.00655	2.97479		4.98134
BAS1	YPL081W	0.658819			2.20651		2.05444			4.91977
BAS1	YPL089C			4.22483						4.22483
BAS1	YPL090C				2.86654		2.0369			4.90344
BAS1	YPL160W				2.29438		1.92309			4.21747
BAS1	YPL186C				2.10686		2.06239			4.16925
BAS1	YPL218W				1.34039	2.15332	1.95141			5.44512
BAS1	YPL253C	4.15608					2.04583			6.20191
BAS1	YPL260W						1.92833	2.93228		4.86061
BAS1	YPR102C	0.595619			2.00826		1.93983			4.54371
BAS1	YPR132W				2.02302		2.07334			4.09636
BAS1	YPR141C					0.957238	2.07557	2.8781		5.91091
BAS1	YPR182W				2.55299		1.84473			4.39772
CAD1	YBR008C		0.654685	5.83238			0.806948		0.698782	7.99279
CAD1	YBR184W			4.78348						4.78348
CAD1	YCL026C-A	4.55787		0.388256					0.503069	5.4492
CAD1	YCL026C-B	4.55787	0.654685							5.21256
CAD1	YCR102C		0.445652	2.63228	0.532683		0.851343		0.731563	5.19352
CAD1	YDL110C			2.47596	0.858938				0.728867	4.06377
CAD1	YDL237W	4.57662	1.28051							5.85713
CAD1	YDL239C	4.57662	1.28051	0.474297		1.28281				7.61424

TF	Target	Binding_0-2000	Motif_Score_SC	Other_Yeasts	Two-Hop_BE	Two-Hop_BP	Two-Hop_BY	Two-Hop_BG	Max_Membership	Sum
CAD1	YDR132C	2.78471	0.884255	8.18271	0.712282					12.564
CAD1	YDR135C	5.28313	0.654685	1.83876						7.77657
CAD1	YDR137W	5.28313	0.654685	2.94774						8.88556
CAD1	YDR453C		0.0952242	3.26846					0.684814	4.0485
CAD1	YDR513W				1.78163		0.944792	1.50085	0.723167	4.95044
CAD1	YDR533C	8.85039	0.872616	1.09843	0.880372				0.729324	12.4311
CAD1	YEL043W	6.76576			0.579053					7.34481
CAD1	YEL044W	6.76576		0.358265						7.12403
CAD1	YEL046C	6.76576		0.373086						7.13885
CAD1	YER147C		0.78337	4.75448						5.53785
CAD1	YER148W		0.78337	4.53387	0.446907					5.76415
CAD1	YFL055W	1.05809	1.28051		1.74499					4.08359
CAD1	YFL056C	1.05809	1.28051	2.08175	0.505539				0.73237	5.65826
CAD1	YFL057C	1.05809	1.28051		1.74494		1.11694		0.716806	5.91729
CAD1	YGL114W	1.47973	0.0972284	3.23549						4.81245
CAD1	YGL156W	1.57313			0.897827	0.878551			0.658801	4.00831
CAD1	YGL197W			5.73285						5.73285
CAD1	YGR209C		0.210312	3.27611	1.37221				0.721574	5.58021
CAD1	YGR233C	4.27301	0.78337	2.71697						7.77335
CAD1	YGR234W	4.27301	0.78337	8.49789						13.5543
CAD1	YHL039W	2.58713	0.872616	9.7447						13.2044
CAD1	YHL040C	2.58713	0.872616	2.71959			0.847976	1.11091	0.726446	8.86467
CAD1	YHR008C	2.06056	0.78337	8.13842						10.9824
CAD1	YHR043C	0.387007		2.08029	0.886729		1.11226			4.46629
CAD1	YHR129C			5.30616						5.30616
CAD1	YHR134W	1.33342	0.445652	3.21032						4.98939
CAD1	YHR139C			5.36302						5.36302
CAD1	YHR140W			5.55159						5.55159
CAD1	YJL100W	6.16017	0.872616	9.06018						16.093
CAD1	YJL101C	6.16017	0.872616	8.69346	0.749546					16.4758
CAD1	YJL163C			3.8502	0.349167				0.723167	4.92253
CAD1	YJR084W		0.03274	5.8097						5.84244
CAD1	YJR104C	0.195161	1.28051	11.7906	1.43461	1.34965			0.715542	16.7661
CAD1	YJR105W	0.195161	1.28051	11.6694			0.877095			14.0222
CAD1	YJR122W	0.238872	0.872616	6.69647	0.568148				0.706178	9.08228
CAD1	YJR137C		0.0972284	4.85554						4.95277
CAD1	YJR138W		0.0972284	6.86017						6.9574
CAD1	YKL040C	3.61548					0.913324			4.5288
CAD1	YKL071W	2.8498	0.0972284	3.20774	0.698986					6.85375
CAD1	YKL085W	4.10774	1.28051						0.583097	5.97135
CAD1	YKL086W	4.10774	1.28051	4.96549						10.3537
CAD1	YKL087C	4.10774	1.28051	6.52838	0.715766					12.6324
CAD1	YKL101W	6.75956	0.872616	3.3805						11.0127
CAD1	YKL103C	6.75956	0.872616	5.4492	1.30748	0.693517			0.731067	15.8134
CAD1	YKL155C	3.53771	0.654685							4.1924
CAD1	YKL187C		0.445652	4.56042					0.560153	5.56622
CAD1	YKR052C	4.12066	1.28051	7.94691	0.833031					14.1811
CAD1	YKR071C	8.09366	0.78337	7.2252						16.1022
CAD1	YKR076W		0.872616	3.10328	0.74714				0.732569	5.4556
CAD1	YLL058W	6.31214	1.28051				0.897639			8.49029
CAD1	YLL060C	6.31214	1.28051	5.77323	0.803789				0.73202	14.9017
CAD1	YLR074C		0.445652	4.88235						5.328
CAD1	YLR075W		0.445652	4.18734						4.63299
CAD1	YLR108C	5.24955	0.78337	6.67495	0.482526					13.1904
CAD1	YLR109W	5.24955	0.78337	8.12091					0.732701	14.8865
CAD1	YLR295C		0.210312	3.72255		0.766417				4.69928
CAD1	YML116W	6.04488	0.78337	1.75636	0.773097		0.929017		0.587553	10.8743
CAD1	YML129C			3.81237	0.692914					4.50528
CAD1	YMR038C	5.60972	0.884255	10.5385						17.0325
CAD1	YMR318C			3.863			0.95423			4.81723
CAD1	YNL134C	1.13204		1.95594	1.91899		1.06115		0.730175	6.7983
CAD1	YNL331C			9.44805					0.71832	10.1664
CAD1	YOL078W	1.11032	1.28051	2.10547						4.4963
CAD1	YOR173W	3.89916		3.33218						7.23134
CAD1	YPL075W			4.32553						4.32553
CAD1	YPL091W		0.884255	11.7921	0.431843					13.1082
CAD1	YPL170W	1.5809	0.884255	4.02773						6.49289
CAD1	YPL171C	1.5809	0.884255	2.92797						5.39313
CAD1	YPL252C			4.12906						4.12906
CAD1	YPR048W	4.14574	0.654685	2.21538	0.928665					7.94447
CBF1	TP(UGG)O3	3.79964	0.592597							4.39224
CBF1	YAL012W	3.396	0.592597	0.200132	0.67475				0.714342	5.57782
CBF1	YAL017W	2.76135	0.592597	3.76908						7.12303
CBF1	YAL018C	2.76135	0.592597	1.20558						4.55953
CBF1	YAL026C	4.22479	0.592597	0.504601		0.7498				6.07179
CBF1	YAL053W	4.09346	0.592597	5.04941		0.785252				10.5207
CBF1	YAL054C	4.09346	0.592597	1.5305						6.21656
CBF1	YBR039W	3.52591	0.592597	1.75488		0.544678				6.41807
CBF1	YBR049C	3.87478	0.592597	0.805136	0.569065					5.84158
CBF1	YBR088C	3.83689	0.592597		0.435113	0.839989				5.70459
CBF1	YBR089C-A	3.83689	0.592597	6.07733		0.948744		1.24115		12.6967
CBF1	YBR126C			2.65937		0.974375			0.467858	4.1016
CBF1	YBR133C	1.79989		3.82724		0.930331		0.950446		7.50791
CBF1	YBR143C	2.82144			1.13376	0.594748				4.54995
CBF1	YBR222C	4.57729	0.592597	1.0151						6.18499
CBF1	YBR249C	4.25195	0.592597	1.7786	0.35552					6.97867
CBF1	YBR250W	4.25195	0.592597	0.876714						5.72126
CBF1	YBR287W	2.03033	0.0334155			0.58132		1.19021	0.559698	4.39497
CBF1	YCL035C	1.36674	0.592597	2.5778	0.680241					5.21738
CBF1	YCR087C-A	4.18286	0.592597	4.85854						9.634
CBF1	YCR088W	4.18286	0.592597	4.21006		1.01673				10.0022
CBF1	YCR092C	3.72176	0.592597	3.56818	0.626663	0.871069				9.38027
CBF1	YCR093W	3.72176	0.592597	2.68459		0.58132				7.58027
CBF1	YCR106W	3.46627	0.592597							4.05887
CBF1	YDL006W	2.32339	0.592597	2.28118						5.19717
CBF1	YDL020C	4.09772	0.592597	1.95981						6.65013
CBF1	YDL051W	2.52392			0.869356	0.834757				4.22803
CBF1	YDL058W	3.51909	0.592597	2.26858		0.406414				6.78668
CBF1	YDL059C	3.51909	0.592597	5.27838					0.675426	10.0655
CBF1	YDL073W	4.55648	0.592597	1.58899						6.73807
CBF1	YDL074C	4.55648	0.592597	2.53035						7.67943
CBF1	YDR054C	3.59453	0.592597							4.18713
CBF1	YDR055W	3.59453	0.592597							4.18713
CBF1	YDR141C	3.98841	0.592597							4.58101
CBF1	YDR160W	4.43061								4.43061
CBF1	YDR190C	3.05388	0.592597	0.906905	0.80577	0.394605				5.75376
CBF1	YDR191W	3.05388	0.592597	0.958985						4.60546
CBF1	YDR398W	3.24818	0.592597	1.67981	0.432052					5.95264
CBF1	YDR438W	0.193327	0.592597	3.23001						4.01593
CBF1	YDR439W	4.86335	0.592597							5.45595

TF	Target	Binding_0-2000	Motif_Score_SC	Other_Yeasts	Two-Hop_BE	Two-Hop_BP	Two-Hop_BY	Two-Hop_BG	Max_Membership	Sum
CBF1	YDR502C	1.49179	0.592597	3.44407						5.52846
CBF1	YDR503C	1.49179	0.592597	3.364						5.44839
CBF1	YDR508C	3.87643	0.592597	2.32025						6.78928
CBF1	YDR510W	3.87643	0.592597	3.72299						8.19202
CBF1	YDR511W	3.87643	0.592597	3.58869						8.05772
CBF1	YDR529C	3.70812	0.592597		0.331496					4.63221
CBF1	YDR530C	3.70812	0.592597	1.94552						6.24624
CBF1	YDR531W	3.70812	0.592597	1.88454						6.18526
CBF1	YEL001C	0.952318	0.592597	1.60726	0.874544					4.02672
CBF1	YEL024W	2.15547	0.592597	1.78075	0.887255					5.41607
CBF1	YEL043W	3.56492	0.592597	1.69563	0.658699					6.51185
CBF1	YEL044W	3.17019	0.592597	2.93903						6.70182
CBF1	YEL046C	3.56492	0.592597	2.66175						6.81927
CBF1	YER017C	4.73946	0.592597	0.379554						5.71161
CBF1	YER019W	4.73946	0.592597	0.752259						6.08432
CBF1	YER043C	3.98639	0.592597	3.63094	0.586799					8.79673
CBF1	YER053C	1.79934	0.592597	2.31772	0.618252				0.710607	6.03852
CBF1	YER057C	1.0349	0.592597	3.51032	1.14247					6.28029
CBF1	YER058W	1.0349	0.592597	4.47528						6.10278
CBF1	YER060W-A	4.37665	0.592597							4.96925
CBF1	YER063W	1.92108	0.592597	3.91569						6.42937
CBF1	YER071C	3.45686	0.0334155			0.880099				4.37037
CBF1	YER072W	3.45686	0.0334155		0.885145			1.13427		5.50969
CBF1	YER073W	3.45686	0.0334155	1.64194				1.03152		6.16374
CBF1	YER091C	4.62167	0.592597	1.22629					0.709264	7.14982
CBF1	YER092W	4.62167	0.592597	1.63835	0.735625					7.58824
CBF1	YER094C	3.50695		0.602125	0.559464	0.411824				5.08036
CBF1	YER095W	3.50695		0.349351		0.675371				4.53167
CBF1	YER099C	4.16324	0.592597	3.27038						8.02622
CBF1	YER100W	4.16324	0.592597	3.82023						8.57607
CBF1	YER111C	3.90779	0.592597					0.860789		5.36118
CBF1	YER112W	3.90779	0.592597	1.34167		0.855718				6.69778
CBF1	YER114C	3.97883	0.592597	1.26103						5.83246
CBF1	YER124C	4.36363	0.592597	1.13665						6.09288
CBF1	YER125W	4.36363	0.592597	1.1436						6.09983
CBF1	YER177W	2.25175	0.592597	1.24727	0.510586					4.6022
CBF1	YFL017W-A	4.48582	0.592597	0.898555		0.699679				6.67665
CBF1	YFL018C	4.48582	0.592597	0.811712		0.333217				6.22335
CBF1	YFL023W	3.93702	0.592597	0.305395						4.83501
CBF1	YFL024C	3.93702	0.592597	0.552391		0.647648				5.72966
CBF1	YFL031W	4.21956	0.592597	0.436635						5.24879
CBF1	YFL033C	4.21956	0.592597	0.213129						5.02529
CBF1	YFR001W		0.592597	2.55455	1.14343					4.29058
CBF1	YFR017C	3.21036	0.592597		0.353088					4.15604
CBF1	YFR030W	2.93052	0.592597	0.785964	0.808865			1.40459	0.567915	7.09045
CBF1	YFR033C	2.38488	0.592597	1.24416	0.336752					4.55839
CBF1	YFR049W	4.03317	0.592597	1.71048						6.33625
CBF1	YGL001C		0.592597	2.56315	0.623164	0.703277				4.48219
CBF1	YGL125W	3.8205	0.592597	2.92473					0.708114	8.04594
CBF1	YGL127C	3.8205	0.592597	1.96714						6.38024
CBF1	YGR016W	1.38803	0.592597	2.60447						4.5851
CBF1	YGR032W	3.3686	0.592597	2.59642						6.55762
CBF1	YGR052W	4.10883	0.592597							4.70143
CBF1	YGR060W	3.21533	0.592597	1.26163						5.06956
CBF1	YGR110W	3.05618	0.592597		0.580117					4.22889
CBF1	YGR128C	4.24789	0.592597		1.26211					6.1026
CBF1	YGR154C	3.18032	0.592597	0.629401					0.510846	4.91316
CBF1	YGR155W	3.18032	0.592597	0.514143						4.28706
CBF1	YGR200C	3.26578	0.592597	0.014447	0.49788					4.3707
CBF1	YGR202C	4.24338	0.592597	0.146901						4.98288
CBF1	YGR204W	4.24338	0.592597	0.183286						5.01926
CBF1	YGR223C	4.57284	0.592597	0.0842575						5.24969
CBF1	YGR224W	4.57284	0.592597	0.298819						5.46426
CBF1	YGR229C	3.01071	0.592597		0.457899					4.06121
CBF1	YGR238C		0.592597	3.54757						4.14017
CBF1	YHL004W	1.18872	0.592597	2.39074		0.406414				4.57847
CBF1	YHL007C	1.18872	0.592597	2.64682		0.443155				4.87129
CBF1	YHR001W-A	3.82751	0.592597	1.24708	0.499638					6.16683
CBF1	YHR002W	3.82751	0.592597							4.42011
CBF1	YHR083W	3.24262	0.592597	1.42407						5.25929
CBF1	YHR089C	4.40816	0.592597	1.45397	0.809638	0.799045				8.06341
CBF1	YHR098C	3.71506	0.592597	1.30434						5.612
CBF1	YHR099W	3.71506	0.592597	0.0919955		0.707567				5.10722
CBF1	YHR112C	2.57603	0.592597	1.64441					0.651625	5.46466
CBF1	YHR113W	2.57603	0.592597	1.65548					0.656366	5.48047
CBF1	YHR135C	3.90384	0.592597							4.49644
CBF1	YHR136C	3.90384	0.592597	5.27582						9.77226
CBF1	YHR137W	3.90384	0.592597	3.1145						7.61094
CBF1	YHR207C			5.19843						5.19843
CBF1	YIL031W	3.49024	0.592597	2.17943						6.26227
CBF1	YIL033C	3.49024	0.592597	2.15878				0.922397		7.16401
CBF1	YIL074C	3.85186	0.592597	1.21797	0.65025				0.691515	7.00419
CBF1	YIL088C	3.66194	0.592597	1.15374					0.646752	6.05503
CBF1	YIL118W	2.33517	0.592597	2.28767	0.532913					5.74835
CBF1	YIL126W	4.61887	0.592597	2.4304		0.680536		1.10024		9.42264
CBF1	YIL127C	4.61887	0.592597	3.3244	1.18633					9.7222
CBF1	YIR017C	3.82255	0.592597	0.0604999	0.433848	0.712044			0.697679	6.31922
CBF1	YIR018W	3.82255	0.592597	1.74432					0.730521	6.88999
CBF1	YIR042C	1.41107	0.592597	1.56294					0.708759	4.27537
CBF1	YJL012C	3.1919	0.592597	2.46987				1.18201		7.43638
CBF1	YJL054W	0.792623	0.592597	3.03209						4.41731
CBF1	YJL060W	2.84004	0.592597	2.1767					0.704851	6.31419
CBF1	YJL100W	3.58698	0.592597	0.893335						5.07291
CBF1	YJL101C	3.58698	0.592597	0.834746	0.327882				0.722351	6.06456
CBF1	YJL133W	3.60547	0.592597	2.80477						7.00284
CBF1	YJL149W	2.99099	0.592597	0.425435						4.00902
CBF1	YJL151C	2.99099	0.592597	0.840338	0.493263					4.91719
CBF1	YJL153C	2.99099	0.592597	0.70786	0.603712					4.89516
CBF1	YJL166W	4.44161	0.592597	3.6959						8.73011
CBF1	YJL167W	4.44161	0.592597	3.91942	0.493811					9.44744
CBF1	YJL168C	4.44161	0.592597	1.41612				1.00554		7.45587
CBF1	YJL186W	4.05641	0.592597	3.27108						7.92009
CBF1	YJL187C	4.05641	0.592597	2.27236		0.321175				7.24254
CBF1	YJL209W	3.7967	0.592597	1.64835						6.03765
CBF1	YJL212C	3.7967	0.592597							4.3893
CBF1	YJR009C	4.89357	0.592597	0.29994	0.642569					6.42868
CBF1	YJR010W	4.89357	0.592597	0.945695	0.342223				0.639468	7.41355
CBF1	YJR060W	3.2478	0.0334155			0.867198				4.14841
CBF1	YJR095W	3.66615	0.592597	1.638						5.89675
CBF1	YJR119C	2.62782	0.592597	0.511582				1.39032		5.12232

TF	Target	Binding_0-2000	Motif_Score_SC	Other_Yeasts	Two-Hop_BE	Two-Hop_BP	Two-Hop_BY	Two-Hop_BG	Max_Membership	Sum
CBF1	YJR121W	2.62782	0.592597	0.482316		0.787121				4.48985
CBF1	YJR137C	4.69204	0.592597	1.20667	0.449032			1.08717	0.699732	8.72724
CBF1	YJR138W	4.69204	0.592597	0.844984						6.12962
CBF1	YJR139C	3.89155	0.592597	0.903355						5.3875
CBF1	YJR145C	3.22855	0.0334155		0.78971					4.05168
CBF1	YKL007W	4.57613	0.592597	0.6086	0.744926	0.577947				7.1002
CBF1	YKL008C	4.57613	0.592597	0.344356						5.51308
CBF1	YKL016C	3.11689	0.592597	0.708423		0.544678				4.96259
CBF1	YKL191W	4.53848	0.592597	1.49092						6.622
CBF1	YKL192C	4.53848	0.592597	1.7821						6.91318
CBF1	YKL193C	4.53848	0.592597	2.77895	0.426784					8.33681
CBF1	YKR057W	4.16584	0.592597		0.823105					5.58154
CBF1	YKR058W	4.16584	0.592597				0.806061		0.553176	6.11767
CBF1	YKR068C	1.91509	0.592597	3.79668						6.30437
CBF1	YKR069W	1.91509	0.592597	2.18283					0.72696	5.41748
CBF1	YLL008W	2.39134	0.592597	1.20126	0.808851					4.99405
CBF1	YLL010C	2.39134	0.592597	1.31676						4.3007
CBF1	YLL055W	4.41692	0.592597	2.3501					0.598217	7.95783
CBF1	YLL056C	4.41692	0.592597							5.00952
CBF1	YLR032W	3.30289						1.03182		4.33471
CBF1	YLR033W	4.83042				0.680536				5.51096
CBF1	YLR058C		0.592597	4.34166						4.93426
CBF1	YLR081W	4.01174	0.592597							4.60434
CBF1	YLR092W	4.69979	0.592597	1.17243	0.381806			0.953316	0.643613	8.44355
CBF1	YLR142W	2.62169	0.592597	0.867632	0.367026			1.18201		5.63096
CBF1	YLR149C	4.26425	0.592597	2.31901						7.17586
CBF1	YLR150W	4.26425	0.592597	0.90462	0.348516					6.10998
CBF1	YLR174W	3.57008	0.592597	2.14575	0.338151					6.64658
CBF1	YLR175W	3.57008	0.592597		0.834885	1.02529				6.02285
CBF1	YLR278C	2.48786	0.592597	1.93156						5.01202
CBF1	YLR283W	2.48786	0.592597	4.33302						7.41348
CBF1	YLR303W	2.53206	0.592597	3.18387	0.512241				0.713406	7.53417
CBF1	YML018C	2.02182	0.592597	1.65402	0.702666					4.9711
CBF1	YML119W	3.66818	0.592597	2.99469						7.25547
CBF1	YML120C	3.66818	0.592597	5.50532			0.778073			10.5442
CBF1	YML121W	3.04155	0.592597	4.80386						8.43801
CBF1	YML123C	3.04155	0.592597	3.95723						7.59138
CBF1	YMR037C	2.97297	0.592597	0.0452576				0.956944		4.56777
CBF1	YMR114C			3.09277	0.612394				0.710103	4.41527
CBF1	YMR195W	2.09498	0.592597	2.33378						5.02136
CBF1	YMR196W	2.26285	0.592597	2.83301					0.482929	6.17139
CBF1	YMR198W	2.78828		0.776288			0.79294	1.00979		5.3673
CBF1	YMR217W	2.08965	0.592597	0.519764	0.830368					4.03238
CBF1	YMR290C	0.620235		2.28292	1.07013	0.608512				4.5818
CBF1	YMR301C	3.35098	0.592597	0.478066						4.42164
CBF1	YMR308C	3.64536	0.592597		0.419394	1.15155				5.8089
CBF1	YNL002C	2.72002	0.592597		1.23105					4.54367
CBF1	YNL036W	4.80579	0.592597	1.3776				0.934216	0.711135	8.42134
CBF1	YNL037C	4.80579	0.592597	1.26306	0.6605					7.32195
CBF1	YNL063W	3.87836	0.592597	1.72662						6.19758
CBF1	YNL064C	3.87836	0.592597	1.08988						5.56084
CBF1	YNL094W	4.37646	0.592597	1.44457		0.964466				7.37809
CBF1	YNL095C	4.37646	0.592597	0.730967	0.363235					6.06326
CBF1	YNL103W	2.64421	0.592597	0.465531		0.867198				4.56954
CBF1	YNL104C	2.64421	0.592597	0.682029	0.414815					4.33365
CBF1	YNL151C	0.31259	0.592597	3.276	0.634805					4.81599
CBF1	YNL175C	3.3942		0.687039	0.983283					5.06452
CBF1	YNL178W	0.223142	0.592597	2.363	1.13495					4.31369
CBF1	YNL220W	3.20298	0.592597	3.22071						7.01629
CBF1	YNL221C	3.20298	0.592597	2.42719						6.22277
CBF1	YNL231C	1.89407	0.592597	2.59755	0.348185					5.4324
CBF1	YNL232W	3.12463	0.592597	4.90869						8.62592
CBF1	YNL256W	0.887169	0.592597	7.85235						9.33212
CBF1	YNL257C	0.887169	0.592597	3.41504						4.89481
CBF1	YNL259C	3.28516	0.592597	0.842849						4.72061
CBF1	YNL260C	3.28516	0.592597	1.25201						5.12977
CBF1	YNL277W	2.20254	0.592597	1.19591					0.474908	4.46596
CBF1	YNL281W	3.5515	0.592597							4.1441
CBF1	YNL282W	3.5515	0.592597	1.92157						6.06567
CBF1	YNL283C	3.5515	0.592597	0.0543381						4.19844
CBF1	YNR032C-A	3.24891	0.592597	0.4821						4.32361
CBF1	YNR051C	4.15387	0.592597	0.850105						5.59657
CBF1	YOL064C	4.03812	0.592597	0.395808					0.722266	5.74879
CBF1	YOL065C	4.03812	0.592597							4.63072
CBF1	YOL100W	3.06398	0.592597	0.425249						4.08183
CBF1	YOR038C	4.51113								4.51113
CBF1	YOR039W	4.51113	0.592597	0.231823		0.58132				5.91687
CBF1	YOR040W	4.51113	0.592597							5.10373
CBF1	YOR064C	3.75323	0.592597	0.436084						4.78191
CBF1	YOR065W	3.75323	0.592597	0.356798	0.560875					5.2635
CBF1	YOR098C	4.87589	0.592597	1.20477		0.819365				7.49262
CBF1	YOR099W	4.87589	0.592597	1.36867						6.83716
CBF1	YOR136W	1.89531	0.592597	0.832309	0.863195	0.755381				4.93879
CBF1	YOR162C	2.62402	0.592597	0.874693						4.09131
CBF1	YOR299W	2.12601	0.592597	1.95724						4.67585
CBF1	YOR303W	3.0469	0.592597	0.716307		0.608788				4.96459
CBF1	YOR327C	3.45017		0.932128						4.3823
CBF1	YOR328W	3.45017		0.60518						4.05535
CBF1	YPL066W	2.49736	0.592597	1.94918						5.03914
CBF1	YPL068C	2.49736	0.592597	1.23502						4.32498
CBF1	YPL170W	3.29592	0.592597					0.884142		4.77266
CBF1	YPL171C	3.29592	0.592597					0.945399		4.83392
CBF1	YPL204W	4.50941	0.592597	0.41686		0.404755				5.92362
CBF1	YPL206C	4.50941	0.592597	0.104892						5.2069
CBF1	YPL235W	3.46935	0.592597	2.81623		0.862552				7.74073
CBF1	YPL236C	3.46935	0.592597	1.62655				0.944748		6.63324
CBF1	YPL250C	2.9901	0.592597	1.82831					0.710205	6.12121
CBF1	YPL254W	3.36929	0.592597	4.70173						8.66362
CBF1	YPR102C	2.13569	0.592597	1.50491	1.08854					5.32174
CBF1	YPR103W	2.13569	0.592597	1.49218	0.484912	0.782002				5.48738
CBF1	YPR167C		0.592597	3.19924	0.466254		0.791626		0.630379	5.6801
CBF1	YPR168W		0.592597	3.69157		0.489398				4.77356
CIN5	TD(GUC)K	4.38817								4.38817
CIN5	TP(UGG)O3	5.35507								5.35507
CIN5	YAL037W	8.61675								8.61675
CIN5	YAL063C	4.06235								4.06235
CIN5	YBL008W	1.89713			1.04719		1.1148			4.05912
CIN5	YBL025W	4.06235								4.06235
CIN5	YBL026W	4.06235			1.18309					5.24544
CIN5	YBL027W	1.56156			1.76506		1.16947		0.621053	5.11714

TF	Target	Binding_0-2000	Motif_Score_SC	Other_Yeasts	Two-Hop_BE	Two-Hop_BP	Two-Hop_BY	Two-Hop_BG	Max_Membership	Sum
CIN5	YBL029C-A	8.11187								8.11187
CIN5	YBL029W	8.11187								8.11187
CIN5	YBL063W	1.07876		2.94186			1.33227			5.35289
CIN5	YBL064C	1.07876		3.90896	0.963931				0.727385	6.67904
CIN5	YBR008C	4.65981			0.35547				0.527861	5.54314
CIN5	YBR025C	1.41287		4.22073	0.854297					6.4879
CIN5	YBR048W	0.96651		0.908191	1.90283		1.197		0.732299	5.70683
CIN5	YBR069C	2.73733		1.59118						4.32851
CIN5	YBR071W	2.73733			1.49286					4.23019
CIN5	YBR072W	2.59515			0.678865		0.799514		0.716522	4.79005
CIN5	YBR078W	5.3223								5.3223
CIN5	YBR169C			3.41521	1.13119					4.5464
CIN5	YBR182C	1.75995		4.92933						6.68928
CIN5	YBR183W	1.75995		5.98473						7.74468
CIN5	YBR291C			4.36301						4.36301
CIN5	YCL059C			2.56718	0.65922	0.389985	1.0358			4.65219
CIN5	YCR102C	2.28408		3.21928	0.840767		0.980093		0.731563	8.05578
CIN5	YDL001W	6.54654								6.54654
CIN5	YDL037C	5.65801		0.233312						5.89132
CIN5	YDL129W	5.77268		0.186967						5.95965
CIN5	YDR077W	4.49946			1.08103					5.58049
CIN5	YDR161W			3.40164	0.733673					4.13531
CIN5	YDR343C	2.50756			1.50529					4.01285
CIN5	YDR368W			3.33217	0.593442		0.84059			4.7662
CIN5	YDR441C	8.84999		1.4336						10.2836
CIN5	YDR508C	3.3449		0.353335			0.934645			4.63288
CIN5	YDR510W	3.3449		0.886894		1.03148				5.26327
CIN5	YDR511W	3.3449		0.833611						4.17851
CIN5	YDR533C	2.49764		1.81679	1.21907					5.5335
CIN5	YEL006W	1.9108		2.4792						4.39
CIN5	YEL011W	3.21147					1.28298			4.49445
CIN5	YEL043W	6.92684			0.509079		1.27473			8.71065
CIN5	YEL044W	6.92684								6.92684
CIN5	YEL046C	6.92684					0.971455			7.8983
CIN5	YEL053C	2.23012		1.87207						4.10219
CIN5	YER028C	6.50791								6.50791
CIN5	YER030W	6.50791			1.39473					7.90264
CIN5	YER045C	7.84882		0.0643655						7.91319
CIN5	YER046W	7.84882		2.68051						10.5293
CIN5	YER049W			3.18058	0.87169					4.05227
CIN5	YER071C	6.94418								6.94418
CIN5	YER073W	6.94418								6.94418
CIN5	YER074W	0.533771			1.94497		0.995673		0.731944	4.20636
CIN5	YER133W					2.00721	1.12375	2.11193		5.24289
CIN5	YFL004W	7.45176								7.45176
CIN5	YFL017C			3.08921	1.2015					4.29071
CIN5	YGL253W	3.85806			0.67002					4.52808
CIN5	YGR123C				1.07934		0.983407	2.02215		4.0849
CIN5	YGR138C	1.21833			1.00553		1.13518		0.726473	4.08551
CIN5	YGR162W			3.32818	1.80307	0.638342				5.76959
CIN5	YGR180C	6.70759			0.590881	0.553953			0.723134	8.57556
CIN5	YGR181W	6.70759								6.70759
CIN5	YGR209C	2.67868			1.13005				0.721574	4.5303
CIN5	YGR238C	9.17984					1.0859			10.2657
CIN5	YGR249W	5.16022								5.16022
CIN5	YHR049W	4.29847								4.29847
CIN5	YHR050W	4.29847					0.895987			5.19446
CIN5	YHR128W	3.11437			1.63893					4.7533
CIN5	YHR133C	2.52206		2.24125						4.76331
CIN5	YHR170W			4.89671	0.749459					5.64617
CIN5	YHR179W	2.83915		3.09391	0.59442		0.99052		0.725971	8.24397
CIN5	YHR203C			2.65407	1.81212		1.15276		0.729283	6.34823
CIN5	YIL045W	2.45738		0.167967	1.23946	1.11356				4.97837
CIN5	YIL052C	0.695131		1.35187	1.97193				0.703285	4.72222
CIN5	YIL070C			3.39962	0.685891					4.08551
CIN5	YIL077C			3.56212	0.900963					4.46308
CIN5	YIL079C			2.63772	0.514438		0.935168			4.08733
CIN5	YIL115C	1.34977				1.73525		1.18238		4.2674
CIN5	YIL118W	7.99822		0.0875695						8.08579
CIN5	YIL119C	7.99822		4.4902					0.479649	12.9681
CIN5	YIR019C	7.27649								7.27649
CIN5	YJL026W	2.66218			1.41725	1.64602				5.72545
CIN5	YJL078C	3.02754					1.25029	1.47865		5.75648
CIN5	YJL171C	2.58848		3.30974	0.906223					6.80444
CIN5	YJL177W			0.418468	1.96626		1.13964		0.717273	4.24164
CIN5	YJL213W			6.56143	0.799158					7.36059
CIN5	YJR010C-A	8.92062								8.92062
CIN5	YJR011C	8.92062		0.762424						9.68304
CIN5	YJR013W	8.92062		0.534502						9.45512
CIN5	YJR094C	6.29559		0.26375						6.55934
CIN5	YJR102C	5.24799								5.24799
CIN5	YJR103W	5.24799								5.24799
CIN5	YJR109C	0.387173			0.492939	1.73918	1.08786	1.46103		5.16818
CIN5	YJR132W	7.03812								7.03812
CIN5	YJR145C	5.55305		0.129416	1.68731		1.15276		0.703229	9.22577
CIN5	YJR147W	5.55305		0.139773	0.561689					6.25451
CIN5	YKL043W	5.82854		8.0726		0.361009				14.2621
CIN5	YKL101W	5.46364								5.46364
CIN5	YKL103C	5.46364			1.05812	0.845558			0.731067	8.09838
CIN5	YKL110C	4.13538		0.359976						4.49536
CIN5	YKL129C	0.645306			0.872319		1.15675	1.75301		4.42739
CIN5	YKR038C	4.38817		2.11478						6.50295
CIN5	YKR039W	4.38817		2.14696						6.53513
CIN5	YKR067W	2.99402			1.74957					4.74359
CIN5	YLL011W	2.76058			1.48313		1.09201			5.33572
CIN5	YLL052C	5.15353						1.30476		6.45829
CIN5	YLL053C	5.15353						1.30476		6.45829
CIN5	YLL054C	3.64556		4.54098						8.18654
CIN5	YLR029C	2.76147			1.69272		1.18133		0.719649	6.35517
CIN5	YLR034C	3.22269					0.899223			4.12191
CIN5	YLR075W	2.43768			1.50714		1.12225			5.06707
CIN5	YLR081W	7.38339			0.509297					7.89269
CIN5	YLR105C	0.895642		3.69168						4.58732
CIN5	YLR121C	8.64581			0.408092					9.0539
CIN5	YLR258W				1.64484	2.00721			0.458441	4.11049
CIN5	YLR312W-A	0.183011		5.44646	1.03562					6.66509
CIN5	YLR330W			1.09653	1.12136		1.09539	1.21832		4.5316
CIN5	YLR344W	0.381879			1.86367		1.11819		0.687598	4.05134
CIN5	YLR413W	6.28727			0.411708					6.69898
CIN5	YLR460C	2.25983			0.567874		0.97316		0.731604	4.53247

TF	Target	Binding_0-2000	Motif_Score_SC	Other_Yeasts	Two-Hop_BE	Two-Hop_BP	Two-Hop_BY	Two-Hop_BG	Max_Membership	Sum
CIN5	YML005W	1.141		1.4912				1.84299		4.47519
CIN5	YML006C	1.141		3.84573						4.98673
CIN5	YML116W	3.94382							0.587553	4.53137
CIN5	YML131W	6.46607		2.28369	1.14378		1.02555		0.731528	11.6506
CIN5	YMR041C	1.4471			1.82595		0.966264			4.23931
CIN5	YMR090W	2.145		5.86889	1.37919				0.72016	10.1132
CIN5	YMR097C			6.35427						6.35427
CIN5	YMR144W			4.12725						4.12725
CIN5	YMR173W	2.71121			0.987646				0.728887	4.42774
CIN5	YMR177W	7.49575		2.81864						10.3144
CIN5	YMR308C			1.62966	1.17565	1.50981				4.31512
CIN5	YNL087W	8.91763			0.902487					9.82012
CIN5	YNL134C	1.262		0.196745	1.22682		0.953254		0.727761	4.36658
CIN5	YNL178W	7.98146			1.19126		0.867641		0.691676	10.732
CIN5	YNL180C	7.98146		4.53906			1.22659			13.7471
CIN5	YOL082W			3.23452	1.46801	0.932971				5.6355
CIN5	YOL096C			5.7378			1.2466			6.9844
CIN5	YOL121C	0.61553			1.8253		1.15276		0.719485	4.31307
CIN5	YOL125W			4.91717	0.502577					5.41975
CIN5	YOL126C			2.84805			1.18667			4.03472
CIN5	YOL151W	5.61187		2.33189	1.31828		0.889469		0.725065	10.8766
CIN5	YOL156W	8.74215		0.137168	0.575387					9.45471
CIN5	YOL157C	8.74215		0.121756						8.86391
CIN5	YOR019W	4.02108			1.26045					5.28153
CIN5	YOR047C	4.13498				1.36195				5.49693
CIN5	YOR049C	7.93831		0.0682031						8.00651
CIN5	YOR167C	0.468064		0.253142	1.76129	0.487637	1.0482			4.01833
CIN5	YOR178C	8.0455		0.101414						8.14691
CIN5	YOR185C			3.97181	1.0303		1.14218			6.14429
CIN5	YOR186W			3.85435	1.01616					4.87051
CIN5	YOR187W			4.99069	0.675535					5.66622
CIN5	YOR302W	7.05272			0.954853	0.574295				8.58187
CIN5	YOR303W	7.05272			0.954853		0.858746			8.86632
CIN5	YOR310C	2.79946			0.936567		1.12721			4.86324
CIN5	YOR311C	2.79946		2.61402						5.41348
CIN5	YOR313C	6.64038			0.968239				0.560188	8.16881
CIN5	YOR315W	6.64038			0.968239					7.60862
CIN5	YOR316C	2.01695		2.39732						4.41427
CIN5	YOR369C	1.03511			1.9405		1.07062		0.647075	4.6933
CIN5	YOR381W	4.39265								4.39265
CIN5	YPL061W	5.16773		0.182095						5.34983
CIN5	YPL088W			3.33571			0.813233		0.554133	4.70308
CIN5	YPL089C			3.06227	1.09213		1.01802			5.17242
CIN5	YPL160W	1.60634			1.33562		1.18759			4.12955
CIN5	YPL165C	6.22278								6.22278
CIN5	YPL179W			4.69246	1.06469		1.14402	2.10952		9.01069
CIN5	YPR063C	5.88024								5.88024
CIN5	YPR065W	5.88024			0.86302					6.74326
CIN5	YPR066W	5.88024					0.996406			6.87665
CIN5	YPR086W			4.42062			1.18133			5.60195
CIN5	YPR124W	3.39682		0.187874	1.05069					4.63538
CSRE	YAL053W			17.435						17.435
CSRE	YAL054C			13.918						13.918
CSRE	YBR083W			4.09708						4.09708
CSRE	YCR005C			6.32656						6.32656
CSRE	YCR010C			4.42786						4.42786
CSRE	YCR034W			6.14041						6.14041
CSRE	YDR407C			4.96229						4.96229
CSRE	YDR437W			4.17782						4.17782
CSRE	YER065C			19.6169						19.6169
CSRE	YER145C			5.2857						5.2857
CSRE	YGL179C			4.42044						4.42044
CSRE	YGR162W			4.04305						4.04305
CSRE	YJR095W			6.36215						6.36215
CSRE	YJR100C			4.63515						4.63515
CSRE	YJR101W			6.1506						6.1506
CSRE	YJR152W			4.38925						4.38925
CSRE	YKR079C			4.02404						4.02404
CSRE	YKR097W			13.4191						13.4191
CSRE	YLR153C			6.097						6.097
CSRE	YLR154C			7.95966						7.95966
CSRE	YLR227C			5.86774						5.86774
CSRE	YLR271W			4.36918						4.36918
CSRE	YLR303W			7.13289						7.13289
CSRE	YLR377C			6.92512						6.92512
CSRE	YLR381W			4.03606						4.03606
CSRE	YML042W			4.07235						4.07235
CSRE	YML043C			4.07087						4.07087
CSRE	YML060W			7.35378						7.35378
CSRE	YNL023C			8.08454						8.08454
CSRE	YOR095C			5.47204						5.47204
CSRE	YOR189W			5.35093						5.35093
CSRE	YOR337W			4.18017						4.18017
CSRE	YPL119C			5.44227						5.44227
CSRE	YPR166C			9.25701						9.25701
CSRE	YPR168W			9.58473						9.58473
CST6	YDR373W	3.28382						0.955322		4.23914
CST6	YMR065W	5.16351								5.16351
CUP2	YBR061C			4.49805						4.49805
CUP2	YCL035C			4.20561						4.20561
CUP2	YCR005C			6.87802						6.87802
CUP2	YDL040C			4.17693						4.17693
CUP2	YDL189W			4.48502						4.48502
CUP2	YDR373W			4.23181						4.23181
CUP2	YER165W			5.55612						5.55612
CUP2	YER180C			4.83442						4.83442
CUP2	YGR072W			4.61774						4.61774
CUP2	YHR103W			4.00298						4.00298
CUP2	YHR117W			5.77934						5.77934
CUP2	YHR133C			4.23381						4.23381
CUP2	YIL125W			6.38269						6.38269
CUP2	YJR076C			4.79893						4.79893
CUP2	YJR104C			4.73924						4.73924
CUP2	YKL028W			4.03091						4.03091
CUP2	YKL029C			4.19658						4.19658
CUP2	YML032C			7.16423						7.16423
CUP2	YML038C			6.46551						6.46551
CUP2	YNR051C			6.36112						6.36112
CUP2	YOL003C			7.03916						7.03916
CUP2	YOR049C			6.88567						6.88567

TF	Target	Binding_0-2000	Motif_Score_SC	Other_Yeasts	Two-Hop_BE	Two-Hop_BP	Two-Hop_BY	Two-Hop_BG	Max_Membership	Sum
CUP2	YOR278W			7.86412						7.86412
CUP2	YPL048W			4.64816						4.64816
CUP2	YPL211W			4.33403						4.33403
CUP9	YFR005C	6.49625								6.49625
CUP9	YFR006W	6.49625								6.49625
CUP9	YIR019C	4.72979								4.72979
CUP9	YKR092C	4.42649								4.42649
CUP9	YKR093W	4.42649								4.42649
CUP9	YOR178C	4.73075								4.73075
DAL80	YBL058W	4.22025								4.22025
DAL80	YBL059W	4.22025								4.22025
DAL80	YDR123C	4.0884								4.0884
DAL80	YDR126W	4.0884								4.0884
DAL80	YER189W	2.93062			0.355355			1.08719		4.37317
DAL80	YER190W	2.93062			0.355355			1.08719		4.37317
DAL80	YNL336W	5.1607								5.1607
DAL80	YNL339C	5.1607								5.1607
DAL81	YAL035W	0.353393		3.24563	0.638005					4.23703
DAL81	YAR008W		2.39014	8.94995						11.3401
DAL81	YBR026C			4.29623						4.29623
DAL81	YBR043C	4.62654								4.62654
DAL81	YBR056W	0.541065		5.08074	0.447178					6.06898
DAL81	YBR068C	3.30041		0.703666						4.00408
DAL81	YBR154C			5.85132		0.344703				6.19602
DAL81	YBR155W			5.71844						5.71844
DAL81	YBR195C	4.1885								4.1885
DAL81	YBR199W			4.01679						4.01679
DAL81	YBR297W		2.63483	8.51541						11.1502
DAL81	YCL024W	6.45861		2.06337						8.52198
DAL81	YCL025C	6.45861		2.08173	0.422402					8.96274
DAL81	YCR023C			5.02127						5.02127
DAL81	YDL210W	2.12812	2.39014	8.2029			0.927989			13.6491
DAL81	YDL236W	4.74306								4.74306
DAL81	YDL237W	4.74306			0.700616					5.44368
DAL81	YDL238C	4.74306								4.74306
DAL81	YDL239C	4.74306								4.74306
DAL81	YDR242W	0.30551	2.63483	4.79363						7.73397
DAL81	YDR476C			7.44659						7.44659
DAL81	YDR508C	5.20059								5.20059
DAL81	YDR510W	5.20059		0.546146						5.74674
DAL81	YDR511W	5.20059		0.551283	0.607155					6.35903
DAL81	YEL007W	2.16657		6.03071						8.19728
DAL81	YEL017W	0.774433		4.96797						5.7424
DAL81	YEL062W	4.25561								4.25561
DAL81	YEL063C	4.25561								4.25561
DAL81	YEL071W	3.74548		1.35786						5.10334
DAL81	YER053C		0.452558	2.99576	0.708064					4.15638
DAL81	YGL209W	0.28626		4.56172						4.84798
DAL81	YGR018C		6.55133	2.84726						9.39859
DAL81	YGR019W	2.25404	6.55133	14.3408						23.1462
DAL81	YGR055W	4.29869					0.943349			5.24204
DAL81	YHL004W	0.552981		4.30976						4.86274
DAL81	YHL010C			4.32389						4.32389
DAL81	YHR124W			4.05592						4.05592
DAL81	YIL057C	1.33493	1.52195	2.82585						5.68273
DAL81	YIL119C	1.08221		3.5397						4.62191
DAL81	YIL155C			5.38529						5.38529
DAL81	YIR012W			4.54812	0.446744					4.99486
DAL81	YIR027C	3.43927		2.72702						6.16629
DAL81	YIR028W	3.43927		2.77577						6.21504
DAL81	YIR029W	4.15602								4.15602
DAL81	YJL088W	3.58846			0.583911					4.17237
DAL81	YJL133W	2.35109		11.0092						13.3603
DAL81	YJR077C			6.8719						6.8719
DAL81	YJR078W			7.64728						7.64728
DAL81	YJR109C	3.76785					0.916838			4.68469
DAL81	YJR148W	5.17923								5.17923
DAL81	YJR149W	5.17923								5.17923
DAL81	YKL103C	2.74222		0.650226	0.539816	0.738447				4.67071
DAL81	YKR035W-A	1.62672		2.85576						4.48248
DAL81	YKR039W	3.87917			0.811254					4.69042
DAL81	YKR092C	5.67466		0.416284						6.09094
DAL81	YKR093W	5.67466			0.549418					6.22408
DAL81	YLL019C			5.64802						5.64802
DAL81	YLR304C	3.33411			1.02753					4.36164
DAL81	YML031W	1.0384	1.31833	7.44115						9.79788
DAL81	YML032C		1.31833	9.24991		0.857149				11.4254
DAL81	YML102W			4.31987		0.965989				5.28586
DAL81	YNL142W	4.77697			0.765916					5.54289
DAL81	YNL204C			9.3198						9.3198
DAL81	YOL058W	3.76483		0.168228	0.805417					4.73848
DAL81	YOL117W	3.31811	0.794012							4.11212
DAL81	YOR001W	0.846769	1.95198		1.2196					4.01835
DAL81	YOR152C	0.413729		7.32083						7.73456
DAL81	YOR185C		0.452558	6.16892						6.62148
DAL81	YOR186W		0.452558	6.22555						6.67811
DAL81	YPL004C			4.60399						4.60399
DAL81	YPL265W	6.47395								6.47395
DAL81	YPR035W	3.85894		0.416018						4.27496
DAL81	YPR040W	4.27613		2.90884						7.18497
DAL81	YPR144C	5.99595			0.537434					6.53338
DAL81	YPR145W	5.99595			0.349657					6.34561
DAL81	YPR151C	0.581659		3.66204						4.2437
DAL81	YPR181C			6.705						6.705
DAL81	YPR182W			7.61766						7.61766
DAL81	YPR194C	4.35231								4.35231
DAL82	TC(GCA)B	7.37613								7.37613
DAL82	YBL058W	4.13623				0.952713				5.08894
DAL82	YBL059W	4.13623								4.13623
DAL82	YBR166C	3.1889			1.38544					4.57434
DAL82	YBR208C	15.058								15.058
DAL82	YBR217W	0.459101		0.572638		3.33393				4.36567
DAL82	YBR247C				2.15433	2.49474				4.64907
DAL82	YCL059C				0.641926	2.49474	0.868923			4.00559
DAL82	YCR034W			0.768612	2.17777		1.2494			4.19578
DAL82	YCR077C			3.14263		1.22837				4.371
DAL82	YCR096C	1.33225		4.34166	0.408256					6.08217
DAL82	YDL123W	4.84512		0.308919						5.15404
DAL82	YDL124W	4.84512			0.364754					5.20987
DAL82	YDL125C	4.84512								4.84512

TF	Target	Binding_0-2000	Motif_Score_SC	Other_Yeasts	Two-Hop_BE	Two-Hop_BP	Two-Hop_BY	Two-Hop_BG	Max_Membership	Sum
DAL82	YDL126C	4.84512				0.761346				5.60647
DAL82	YDL131W				1.83704			3.26898		5.10602
DAL82	YDL170W	5.82135		0.0895763						5.91093
DAL82	YDL171C	5.82135		1.09653			2.02874			8.94662
DAL82	YDL182W			1.2301	1.98521			3.26898		6.48429
DAL82	YDL237W	3.17297		2.15433						5.3273
DAL82	YDL238C	3.17297		1.45302						4.62599
DAL82	YDR144C	4.80467								4.80467
DAL82	YDR145W	4.80467								4.80467
DAL82	YDR388W					4.11544				4.11544
DAL82	YDR496C	1.17212			1.79075		1.24963			4.2125
DAL82	YEL062W	0.700203		3.65855						4.35875
DAL82	YEL063C	0.700203		1.27102	2.29321					4.26443
DAL82	YER045C	4.05361		0.555598						4.60921
DAL82	YER046W	4.05361		0.0294305						4.08304
DAL82	YGR019W	0.521851		1.41498	0.525934		1.88042			4.34319
DAL82	YGR075C	2.64383		1.39224						4.03607
DAL82	YGR128C			1.77273	2.23712					4.00985
DAL82	YGR233C	0.928436			2.49134		1.46787	0.998389		5.88603
DAL82	YGR262C	0.440273				3.33393	0.807722			4.58193
DAL82	YHL016C	6.12356		0.049948	2.48231					8.65582
DAL82	YHR071W	7.64311								7.64311
DAL82	YHR135C			1.88722		3.33393				5.22115
DAL82	YHR141C	3.57646			0.411007		0.840753			4.82822
DAL82	YHR169W				1.7428	3.33393				5.07673
DAL82	YIR005W					3.33393	0.880655			4.21458
DAL82	YIR025W	4.12106		1.06168						5.18274
DAL82	YIR026C	7.19841								7.19841
DAL82	YIR027C	15.8551		2.50878	1.48616		0.850691			20.7007
DAL82	YIR028W	15.8551		1.16849						17.0236
DAL82	YIR029W	7.72135		2.22856	2.56963					12.5195
DAL82	YIR030C	5.60207		2.51801						8.12008
DAL82	YIR031C	11.2083		2.4645	2.43539					16.1082
DAL82	YIR032C	9.18441		1.43766					0.699891	11.322
DAL82	YIR033W	11.2083		0.301529						11.5098
DAL82	YJL008C	3.5741					0.812704			4.3868
DAL82	YKL081W				1.80326	2.67049	1.41064			5.88439
DAL82	YKL101W	3.94209		0.060854						4.00294
DAL82	YKL103C	3.94209		0.608788	0.549218	0.512336				5.61243
DAL82	YLL034C	0.784188		1.77015	1.83613					4.39047
DAL82	YLR001C			4.34788						4.34788
DAL82	YLR003C			5.20576	1.56441					6.77017
DAL82	YLR008C	1.45023		4.6785						6.12873
DAL82	YLR011W	0.990274		4.1782						5.16847
DAL82	YLR186W			0.911671	2.27249	3.33393				6.51809
DAL82	YLR295C	5.81835								5.81835
DAL82	YLR297W	5.81835								5.81835
DAL82	YLR436C	15.8551			0.478639					16.3337
DAL82	YLR437C	15.8551								15.8551
DAL82	YLR438C-A	5.16046					0.828555			5.98901
DAL82	YLR438W	15.8551								15.8551
DAL82	YLR439W	5.3821			2.61799					8.00009
DAL82	YMR108W			3.33871	1.43242					4.77113
DAL82	YMR121C	1.6086		2.25286	0.410121					4.27158
DAL82	YMR206W			4.43735						4.43735
DAL82	YMR207C			1.22948				3.27249		4.50197
DAL82	YMR251W-A	3.58966		0.79184	0.493739					4.87524
DAL82	YMR264W	1.99317			2.48896					4.48213
DAL82	YMR271C	2.59824					2.08262			4.68086
DAL82	YMR272C	3.61317			0.483717					4.09689
DAL82	YMR276W			4.77768						4.77768
DAL82	YNL004W	5.42773		0.243447						5.67118
DAL82	YNL005C	5.42773		0.182772			0.797175			6.40768
DAL82	YOL020W	4.0962								4.0962
DAL82	YOL021C	4.0962				0.364261				4.46046
DAL82	YOL077C	2.11736			1.85639		1.29398			5.26773
DAL82	YOR001W	2.66529		0.469973	2.15136	0.750539				6.03716
DAL82	YOR027W			1.76341	2.49681		1.51754			5.77776
DAL82	YOR143C	8.66909								8.66909
DAL82	YOR190W	0.0613898		5.5791						5.64049
DAL82	YOR303W			0.930372			1.91689	2.89083		5.73809
DAL82	YPL054W	5.85202								5.85202
DAL82	YPL056C	5.85202								5.85202
DAL82	YPL057C	5.85202								5.85202
DAL82	YPL211W			2.58536	1.59711					4.18247
DAL82	YPL263C			0.722722	1.76227		1.49181	1.02811		5.00491
DAL82	YPR093C			3.62341	0.432155					4.05556
DAL82	YPR144C	10.5573		0.968253	0.539238					12.0648
DAL82	YPR145W	10.5573		0.998262						11.5556
DAT1	YDR545W	4.59257								4.59257
DAT1	YER188C-A	4.31775								4.31775
DAT1	YER189W	4.31775			0.638709			1.33714		6.2936
DAT1	YER190W	4.31775			0.638709			1.33714		6.2936
DAT1	YGL001C	5.11279								5.11279
DAT1	YNL336W	5.17651								5.17651
DAT1	YNL339C	5.17651								5.17651
DIG1	SNR40	9.41338								9.41338
DIG1	TA(AGC)H	13.7085								13.7085
DIG1	TD(GUC)J1	4.31524								4.31524
DIG1	TD(GUC)L2	14.2094								14.2094
DIG1	TD(GUC)O	5.05856								5.05856
DIG1	TF(GAA)D	15.8551								15.8551
DIG1	TG(GCC)B	15.8551								15.8551
DIG1	TI(AAU)P2	9.81011								9.81011
DIG1	TK(CUU)E1	5.05415								5.05415
DIG1	TK(UUU)P	4.18976								4.18976
DIG1	TL(CAA)D	4.2498								4.2498
DIG1	TL(UAA)N	7.18649								7.18649
DIG1	TP(UGG)N1	12.3833								12.3833
DIG1	TQ(UUG)D2	9.86413								9.86413
DIG1	TT(AGU)N1	7.64332								7.64332
DIG1	TV(AAC)K2	8.46551								8.46551
DIG1	TW(CCA)G1	5.582								5.582
DIG1	TX(XXX)D	5.17988								5.17988
DIG1	YAL029C	0.97092			0.665273		1.87498	3.4051	0.697086	7.61336
DIG1	YAR007C				1.99126	2.30763				4.29889
DIG1	YAR014C	9.48593								9.48593
DIG1	YAR015W	9.48593								9.48593
DIG1	YBL004W	4.42026		1.12407						5.54433
DIG1	YBL016W	6.36523			1.69757	1.7019	1.60613			11.3708

TF	Target	Binding_0-2000	Motif_Score_SC	Other_Yeasts	Two-Hop_BE	Two-Hop_BP	Two-Hop_BY	Two-Hop_BG	Max_Membership	Sum
DIG1	YBL017C	6.36523		0.326112		3.73262				10.424
DIG1	YBL034C	0.233866		1.79731		0.343085	1.66416			4.03842
DIG1	YBL112C				0.944056			3.40826		4.35232
DIG1	YBR009C	0.414926			2.82275	2.02796			0.636584	5.90222
DIG1	YBR011C	3.70108		0.271753	2.85751		1.64822			8.47856
DIG1	YBR040W	5.16248			3.19301				0.440728	8.79622
DIG1	YBR041W	5.16248			0.479246					5.64173
DIG1	YBR081C	2.12325					1.61459	0.882026		4.61987
DIG1	YBR082C	11.0174								11.0174
DIG1	YBR083W	11.0174				3.33393				14.3513
DIG1	YBR088C	0.528806			2.12511	2.49816			0.726537	5.87861
DIG1	YBR096W	0.613493			2.7494				0.705027	4.06792
DIG1	YBR109C				2.20048	0.834408	1.63907			4.67396
DIG1	YBR155W			0.0823729	2.16838	0.369544	1.627			4.2473
DIG1	YBR212W	0.53821		0.127002			1.67726	2.38458		4.72705
DIG1	YCL027W	15.8551			1.96264	0.343085			0.725414	18.8862
DIG1	YCL029C	15.8551					1.88697			17.7421
DIG1	YCL035C				2.78983		1.31894			4.10877
DIG1	YCL043C				2.76788		1.85759		0.577297	5.20277
DIG1	YCL054W	1.04869			2.41607		1.29636			4.76112
DIG1	YCL055W	12.2298			2.49835				0.729409	15.4576
DIG1	YCL056C	12.2298		0.919904						13.1497
DIG1	YCL059C				2.83948	0.838022	1.8862			5.5637
DIG1	YCL066W			0.98479	2.49083	4.11544				7.59106
DIG1	YCR009C				2.29227	2.84294				5.13521
DIG1	YCR023C	3.60078		0.0658525			1.29516			4.96179
DIG1	YCR026C	3.25637			0.868614					4.12498
DIG1	YCR067C				2.85125			1.24925		4.1005
DIG1	YCR089W	15.8551								15.8551
DIG1	YDL010W				2.67931		1.67578		0.709014	5.0641
DIG1	YDL065C					2.81099	1.7333			4.54429
DIG1	YDL070W				3.03235		1.52909	0.881283		5.44272
DIG1	YDL101C			0.692588	1.59166	1.63495			0.656947	4.57614
DIG1	YDL117W	0.0664531		2.08175	1.88691					4.03511
DIG1	YDL127W	11.9001		1.49934	0.566933					13.9664
DIG1	YDL153C				2.10153	3.44746	0.952907			6.5019
DIG1	YDL156W				1.08009	2.30763	0.86818		0.610166	4.86607
DIG1	YDL197C				1.74688		1.96739	2.72249	0.610551	7.04731
DIG1	YDR075W				1.11782	3.33393	2.04554			6.49729
DIG1	YDR085C	15.8551			1.199					17.0541
DIG1	YDR097C	9.86413					0.943881		0.687202	11.4952
DIG1	YDR098C	9.86413								9.86413
DIG1	YDR146C	0.396628			1.53776	2.60348	1.66464	0.984893	0.693765	7.88117
DIG1	YDR240C	3.05951				1.27475				4.33426
DIG1	YDR251W	0.627446		0.735747	2.95785		1.03553			5.35657
DIG1	YDR281C	7.25494								7.25494
DIG1	YDR295C						2.08792	3.41613		5.50405
DIG1	YDR309C	9.24728			1.00349					10.2508
DIG1	YDR312W	2.84547			2.25621					5.10168
DIG1	YDR323C						1.93125	3.28268		5.21393
DIG1	YDR339C	5.17988		0.00791489	0.476089					5.66388
DIG1	YDR369C	4.0637			0.372202					4.4359
DIG1	YDR370C	4.0637		0.0247715						4.08847
DIG1	YDR371W	4.0637		0.0304865	1.83382					5.92801
DIG1	YDR373W	0.35881			0.388824		1.655	1.80087		4.2035
DIG1	YDR393W						1.98353	2.74111	0.712752	5.43739
DIG1	YDR432W			2.06721		1.36307			0.578063	4.00834
DIG1	YDR468C			0.45512		3.73262				4.18774
DIG1	YDR480W					4.11544				4.11544
DIG1	YDR503C			2.69382	1.39807					4.09189
DIG1	YDR505C			2.72305	2.0915					4.81455
DIG1	YDR506C	4.2498								4.2498
DIG1	YDR518W			1.65395			2.015		0.70249	4.37144
DIG1	YDR532C			3.71339			2.09099	2.73429		8.53867
DIG1	YEL048C			2.5957	2.02542					4.62112
DIG1	YEL061C						1.95824	2.60935	0.712379	5.27997
DIG1	YER016W			0.879215		3.7382			0.472508	5.08992
DIG1	YER048W-A	7.72458								7.72458
DIG1	YER049W	7.72458			1.76772					9.4923
DIG1	YER075C	1.48013		0.575509		1.55361			0.625155	4.2344
DIG1	YER082C				2.07575	0.838022	1.60831			4.52208
DIG1	YER094C	1.99751		0.0270834	0.45519		1.87122			4.351
DIG1	YER095W	1.99751		0.0109229		1.07611	1.8488		0.45128	5.38462
DIG1	YER118C	2.92313				1.20097			0.723639	4.84774
DIG1	YER133W	0.187037				1.95174	2.03499			4.17377
DIG1	YER155C	1.73556		1.31623		2.20552			0.544443	5.80175
DIG1	YFL005W				1.99409	2.37499	1.99452			6.3636
DIG1	YFL026W	3.8829		0.890121	0.385574					5.1586
DIG1	YFL027C	3.8829		0.526864						4.40976
DIG1	YFL037W	1.88106					1.52909		0.694369	4.10452
DIG1	YFL039C	1.88106				4.11544				5.9965
DIG1	YFL065C				0.884746			3.40485		4.2896
DIG1	YFL066C				0.815276			3.39949		4.21477
DIG1	YFR016C	0.115641			0.627731		1.9984	2.65099		5.39276
DIG1	YFR019W						1.85921	2.965		4.82421
DIG1	YFR023W	2.62066		0.725912			1.62177			4.96834
DIG1	YFR025C	2.82087		0.505799	2.44548					5.77215
DIG1	YGL032C	4.00223		0.222325	2.67749				0.569655	7.4717
DIG1	YGL044C					1.60452	1.5623	2.37692		5.54374
DIG1	YGL075C						1.93741	3.28268		5.22009
DIG1	YGL089C			2.58236	2.50349					5.08585
DIG1	YGL095C	1.38616		0.965738		3.54208	1.67244			7.56642
DIG1	YGL097W	6.58558					0.92094		0.630283	8.1368
DIG1	YGL106W			1.0812			1.63907	1.80255		4.52282
DIG1	YGL116W	5.70517		0.546425		0.966328	1.6099			8.82782
DIG1	YGL117W	5.582		0.310914	0.375686					6.2686
DIG1	YGL120C				2.26547	2.22592				4.49139
DIG1	YGL157W			1.0729	2.94861				0.586386	4.6079
DIG1	YGL229C			0.558317	2.87288				0.586958	4.01816
DIG1	YGR013W	5.33224				1.46351				6.79575
DIG1	YGR014W	5.98206		0.757628					0.705133	7.44482
DIG1	YGR040W			1.19051		4.11544				5.30595
DIG1	YGR088W			1.5138	2.58947					4.10327
DIG1	YGR130C						1.87687	2.89939		4.77626
DIG1	YGR152C	0.181069		1.81761	0.411845		1.6054		0.661664	4.67759
DIG1	YGR165W	2.94969		1.69652						4.64621
DIG1	YGR172C	3.31521				1.20097			0.716924	5.2331
DIG1	YGR185C				1.99635		1.87283		0.67325	4.54243
DIG1	YGR189C	11.1542		1.81093	1.04366				0.555406	14.5642
DIG1	YGR212W			2.4645	1.70405					4.16855

TF	Target	Binding_0-2000	Motif_Score_SC	Other_Yeasts	Two-Hop_BE	Two-Hop_BP	Two-Hop_BY	Two-Hop_BG	Max_Membership	Sum
DIG1	YGR214W				2.7796		1.89265			4.67225
DIG1	YGR233C	4.87403								4.87403
DIG1	YGR234W	4.87403								4.87403
DIG1	YGR238C				1.3488		2.04936	2.72249	0.716349	6.837
DIG1	YGR285C			2.19401	1.63629	2.20552			0.60133	6.63715
DIG1	YGR294W	1.70358			2.63398					4.33756
DIG1	YHL010C						2.11723	2.73583		4.85306
DIG1	YHL013C				0.469772		1.23615	3.1312		4.83712
DIG1	YHL033C			2.58536	0.446782		1.93207			4.96421
DIG1	YHR005C	6.91456		0.193736		1.55361	1.68657			10.3485
DIG1	YHR079C	0.322465		1.0414	3.09492		1.63042			6.08921
DIG1	YHR084W	15.8551		1.57759		2.02796			0.542415	20.0031
DIG1	YHR086W	6.03148		1.16838		1.27475	1.64496	2.36891		12.4885
DIG1	YHR107C					4.11544				4.11544
DIG1	YHR158C					1.20097	2.04939	3.16005		6.41041
DIG1	YHR205W	6.90053					0.938916			7.83945
DIG1	YIL015W	2.8358		0.539662	3.11728		1.68131			8.17405
DIG1	YIL036W	15.8065		1.16621						16.9727
DIG1	YIL037C	15.8065		0.936638	2.48269					19.2258
DIG1	YIL083C	11.1374		0.00301196						11.1404
DIG1	YIL084C	11.1374								11.1374
DIG1	YIL123W	7.61717		0.0968201	0.612024				0.679809	9.00582
DIG1	YIL138C					0.343085	1.98978	2.74719	0.519134	5.59919
DIG1	YIL144W					1.00587	1.89071	3.25364		6.15022
DIG1	YJL036W	0.0720593					2.10292	2.99806		5.17304
DIG1	YJL078C	2.33184		0.421261			1.64136			4.39446
DIG1	YJL112W	4.31524								4.31524
DIG1	YJL138C				0.410462	1.63495		3.01037		5.05578
DIG1	YJL148W	1.93403			2.5529					4.48693
DIG1	YJL170C	5.38928			2.81857					8.20785
DIG1	YJL171C	5.38928			1.48779					6.87707
DIG1	YJL200C	2.42692		0.706381			1.24673			4.38003
DIG1	YJR062C				2.65412		1.88162			4.53574
DIG1	YJR154W	11.2116		0.352662						11.5643
DIG1	YKL010C					2.81099	1.31783			4.12882
DIG1	YKL094W	9.56153								9.56153
DIG1	YKL095W	9.56153								9.56153
DIG1	YKL096W	2.97483			0.829787				0.64856	4.45318
DIG1	YKL103C	0.234691			1.74559	3.33393				5.31421
DIG1	YKL104C	5.30224		0.604601					0.700354	6.60719
DIG1	YKL113C				1.88348		1.89084		0.721773	4.49609
DIG1	YKL129C			0.492117	3.15116			3.40567		7.04895
DIG1	YKL144C			0.241703	2.05716		1.84473			4.14359
DIG1	YKL150W				2.51047		1.90686			4.41733
DIG1	YKL179C	0.905768					1.97091	3.36085		6.23753
DIG1	YKL189W	13.918		1.7538	3.00683					18.6786
DIG1	YKL193C				2.85756		1.65805			4.51561
DIG1	YKR010C			1.33131	1.65963		1.70653			4.69747
DIG1	YKR011C	2.14358			2.94966				0.603888	5.69713
DIG1	YKR013W	2.14358		0.543733	2.85463			2.94117	0.698386	9.1815
DIG1	YKR014C			1.81748	2.25393		1.6727			5.74411
DIG1	YKR042W	1.07217			2.52437				0.730131	4.32667
DIG1	YLL057C	2.66876		0.0459366	1.36109					4.07579
DIG1	YLR019W	0.298513					1.53303	2.41678		4.24832
DIG1	YLR026C			0.120415		3.73262	0.908159			4.76119
DIG1	YLR045C					2.92563	1.96332	2.72831		7.61726
DIG1	YLR084C	3.85176							0.681804	4.53356
DIG1	YLR093C			0.158308	1.95944	3.73262				5.85037
DIG1	YLR178C				3.07502		1.89037			4.96539
DIG1	YLR186W	0.301647		3.34588	1.86333	0.369544				5.8804
DIG1	YLR199C	0.501726		3.90288	1.14024					5.54485
DIG1	YLR210W	0.858153		2.2546			1.66958			4.78233
DIG1	YLR224W			4.59949						4.59949
DIG1	YLR229C			1.67282		2.33161	1.52909			5.53352
DIG1	YLR275W	2.32203		0.0330061		1.27475	1.65258			5.28237
DIG1	YLR276C			2.43242	2.1249					4.55732
DIG1	YLR293C			0.530527		1.61309	1.61281		0.446128	4.20256
DIG1	YLR309C						2.07209	2.81386		4.88595
DIG1	YLR314C						2.07543	3.00653		5.08196
DIG1	YLR332W	3.56518			0.711516				0.56656	4.84326
DIG1	YLR353W	4.19136								4.19136
DIG1	YLR423C						2.01363	2.74358		4.75721
DIG1	YLR431C						1.96442	3.41601		5.38043
DIG1	YLR452C	5.05897		0.343776	2.8761	1.7019			0.710249	10.691
DIG1	YLR453C	3.23058		0.407735		2.15412				5.79243
DIG1	YLR457C				2.82025	1.20097				4.02122
DIG1	YML046W	5.07015		0.511435		1.27475				6.85633
DIG1	YML047C	5.07015		0.0709255	2.93665					8.07773
DIG1	YML064C				0.684212	2.74391	1.66045			5.08857
DIG1	YML085C					4.11544			0.691535	4.80698
DIG1	YML093W				2.2872		1.84611			4.13331
DIG1	YML124C	3.3787		0.802614			1.52909		0.628672	6.33908
DIG1	YMR001C			2.99161	0.751347				0.692418	4.43538
DIG1	YMR012W	0.754511				2.95111	2.02057		0.713345	6.43954
DIG1	YMR014W	1.69058		0.358962	2.34204					4.39158
DIG1	YMR031C	0.429428					1.92602	3.04455		5.4
DIG1	YMR043W	0.739146				4.11544	0.908789			5.76337
DIG1	YMR065W	6.98819			3.01029		1.83046			11.8289
DIG1	YMR116C				2.47206	2.20552	0.807433			5.48501
DIG1	YMR119W	2.39363			2.85274					5.24637
DIG1	YMR128W			1.26683	1.82535	2.65132				5.7435
DIG1	YMR164C	7.03259		0.0475158						7.08011
DIG1	YMR197C	14.4371				1.55361				15.9907
DIG1	YMR198W	14.4371					2.05347			16.4906
DIG1	YMR232W	11.1414		1.36998	0.630015					13.1414
DIG1	YMR239C				2.21854		1.85489			4.07343
DIG1	YMR246W	0.441115		0.474344	1.73046		0.825559	1.47379	0.503324	5.44859
DIG1	YMR277W						1.58815	2.48396		4.07211
DIG1	YMR290C			3.76218	1.88727					5.64945
DIG1	YMR294W			2.14714			2.00307			4.15021
DIG1	YMR305C	4.52721		1.93939	1.97519				0.714726	9.15652
DIG1	YMR306W	4.52721		2.47851						7.00572
DIG1	YNL042W-B	12.3833								12.3833
DIG1	YNL079C					0.343085	2.09021	2.74358		5.17687
DIG1	YNL082W			0.924917	1.72093		1.41649			4.06234
DIG1	YNL087W	1.40246			2.2795		0.952907			4.63487
DIG1	YNL098C				2.58406		1.67887			4.26293
DIG1	YNL102W				1.31225	3.23601				4.54826
DIG1	YNL110C				2.12427	0.838022	1.64353	1.11911		5.72493
DIG1	YNL175C	0.315028			2.45527		1.69725	1.36042		5.82797

TF	Target	Binding_0-2000	Motif_Score_SC	Other_Yeasts	Two-Hop_BE	Two-Hop_BP	Two-Hop_BY	Two-Hop_BG	Max_Membership	Sum
DIG1	YNL192W	8.44166			2.8258					11.2675
DIG1	YNL208W	1.59935			2.88176					4.48111
DIG1	YNL272C	9.40787		0.0161638			2.09425			11.5183
DIG1	YNL279W	15.8551			2.61112					18.4662
DIG1	YNL280C	15.8551								15.8551
DIG1	YNL284C	2.31252		0.101414		0.838022	1.89717			5.14913
DIG1	YNL289W	9.41338								9.41338
DIG1	YNL291C	9.41338								9.41338
DIG1	YNL295W			4.34166						4.34166
DIG1	YNL298W	4.32882		0.931365						5.26018
DIG1	YNL313C	1.19069		0.661276	0.813432	1.07611	0.916821			4.65833
DIG1	YNR006W						1.9172	2.99645		4.91365
DIG1	YNR026C	4.10266								4.10266
DIG1	YNR028W	4.10266								4.10266
DIG1	YNR044W			0.518487	2.93363				0.709255	4.16137
DIG1	YNR054C	11.8255			2.17155					13.997
DIG1	YOL006C						2.03887	3.00497		5.04384
DIG1	YOL034W	0.245053					1.84788	2.61634		4.70927
DIG1	YOL090W				1.87666	2.49816	0.943881		0.551439	5.87014
DIG1	YOL091W	0.404796		0.671304			2.0916	3.12798		6.29568
DIG1	YOR036W				2.16996	3.73262	2.05395			7.95653
DIG1	YOR129C	6.85187								6.85187
DIG1	YOR130C	5.05856			0.482612					5.54117
DIG1	YOR165W						1.73422	2.76462	0.575974	5.07481
DIG1	YOR177C						2.1128	3.15278		5.26558
DIG1	YOR191W				2.97814		1.82592	0.941112		5.74517
DIG1	YOR193W	1.15558			2.64097				0.474151	4.2707
DIG1	YOR198C				0.926725		2.09502	3.14628		6.16803
DIG1	YOR216C						1.90629	3.41601		5.3223
DIG1	YOR236W	1.53637		0.200711	1.44188		1.84418			5.02314
DIG1	YOR246C	1.10421		2.11478			0.838611			4.0576
DIG1	YOR288C	3.9895					1.86323			5.85273
DIG1	YOR326W						2.07816	3.39578	0.709707	6.18365
DIG1	YOR341W			1.30973	2.27618	0.464775				4.05069
DIG1	YOR342C	13.7862			1.77101					15.5572
DIG1	YOR344C	2.78946		1.96999	1.54525					6.3047
DIG1	YPL043W				2.36845	0.838022	1.63533	1.06579		5.90759
DIG1	YPL049C	1.38182		0.333537		4.11544	1.13499			6.96579
DIG1	YPL076W			4.4337						4.4337
DIG1	YPL077C			4.59771						4.59771
DIG1	YPL093W				2.35374		1.87482			4.22856
DIG1	YPL152W			2.22104	2.13039					4.35143
DIG1	YPL154C	0.631216		0.71486	2.95067		1.42063			5.71738
DIG1	YPL156C	13.6037			2.82001					16.4237
DIG1	YPL160W				1.68568		1.86553		0.538487	4.0897
DIG1	YPL163C	5.83882			2.4977				0.5241	8.86062
DIG1	YPL164C	5.83882					1.41649			7.25531
DIG1	YPL174C						2.05107	3.06137		5.11244
DIG1	YPL177C	2.7326		0.624458			1.64836			5.00542
DIG1	YPL178W	0.22438				1.27298	1.62393	2.41358		5.53487
DIG1	YPL186C				2.51451		2.088			4.60251
DIG1	YPL192C	12.4203		0.448608	2.91555					15.7845
DIG1	YPL217C				2.90124		1.64688			4.54812
DIG1	YPL235W					2.30763	2.01965		0.642631	4.96991
DIG1	YPL242C				0.854105	4.11544			0.61232	5.58187
DIG1	YPL253C						1.98396	3.41607		5.40003
DIG1	YPL255W			0.973975			1.88157	2.78753		5.64307
DIG1	YPL256C			2.11919	1.68419				0.676277	4.47966
DIG1	YPR049C						2.09624	3.4069		5.50314
DIG1	YPR060C	5.27453								5.27453
DIG1	YPR062W	5.27453								5.27453
DIG1	YPR072W			0.987837			1.99132	2.78753		5.76669
DIG1	YPR093C				2.45655		1.64325			4.0998
DIG1	YPR119W	4.18976			0.535511	1.37542	0.891211		0.660814	7.65272
DIG1	YPR133C	12.5975		1.53714			2.05909	1.18576		17.3795
DIG1	YPR135W	12.5975							0.711185	13.3087
DIG1	YPR141C	0.740043		0.612984	2.06629	3.73262	2.0932	2.94903		12.1942
DIG1	YPR198W	1.96265			2.17844		1.30538			5.44647
DIG1	YPR202W				0.777203			3.40826		4.18546
ECB	YBR142W			4.1951						4.1951
ECB	YBR282W			5.59321						5.59321
ECB	YBR286W			4.06787						4.06787
ECB	YLR028C			4.4747						4.4747
ECB	YML114C			4.02252						4.02252
ECB	YNL032W			4.34166						4.34166
ECB	YNL241C			4.37872						4.37872
FHL1	SNR11	5.79715	1.95632							7.75347
FHL1	SNR13	7.00698	0.649233							7.65621
FHL1	SNR17A	6.30111	0.0448876							6.346
FHL1	SNR24	3.89917	1.73754							5.63671
FHL1	SNR34	4.07577								4.07577
FHL1	SNR55	4.5552								4.5552
FHL1	SNR57	4.5552								4.5552
FHL1	SNR59	5.79675	0.977661							6.77441
FHL1	SNR63	5.12705								5.12705
FHL1	SNR72	4.30053	1.58189							5.88242
FHL1	SNR73	4.30053	1.58189							5.88242
FHL1	SNR74	4.30053	1.58189							5.88242
FHL1	SNR75	4.30053	1.58189							5.88242
FHL1	SNR76	4.30053	1.58189							5.88242
FHL1	TE(UUC)L	4.5552								4.5552
FHL1	TP(UGG)L	7.22117								7.22117
FHL1	YAL003W		0.0691049		1.45372	0.830391	0.931853		0.731639	4.01671
FHL1	YAR028W	6.65235								6.65235
FHL1	YAR029W	6.65235								6.65235
FHL1	YBL027W	6.96994			1.73548		1.10496		0.731575	10.542
FHL1	YBL028C	6.96994		0.615927	1.36059				0.732035	9.67849
FHL1	YBL069W	5.88599	1.09369	1.78103	0.726225					9.48693
FHL1	YBL071W-A	0.964196	1.09369	2.88713						4.94502
FHL1	YBL072C	5.88599	1.09369	2.01439	1.76825		1.06109		0.72958	12.553
FHL1	YBL087C	6.36116		1.8539	1.81149		0.853225		0.730586	11.6104
FHL1	YBL088C	6.36116								6.36116
FHL1	YBL092W	5.20781	1.73754	1.82222	1.69123		1.11339		0.732408	12.3046
FHL1	YBL093C	5.20781	1.73754	3.7844					0.665995	11.3957
FHL1	YBR009C				1.76801	1.36198	0.93257		0.570602	4.63316
FHL1	YBR046C	7.04154	0.93115							7.97269
FHL1	YBR048W	7.04154	0.93115		1.81253		1.11328		0.732681	11.6312
FHL1	YBR084C-A	6.517	0.902249		1.77834		1.10496		0.731401	11.034
FHL1	YBR084W	0.0584976			1.45951	1.45133	0.811897		0.732249	4.51348
FHL1	YBR085W	6.517	0.902249	5.29217	1.03192					13.7433

TF	Target	Binding_0-2000	Motif_Score_SC	Other_Yeasts	Two-Hop_BE	Two-Hop_BP	Two-Hop_BY	Two-Hop_BG	Max_Membership	Sum
FHL1	YBR117C	3.17114	0.785737	1.60183	0.435022					5.99373
FHL1	YBR118W	3.17114	0.785737	1.42528	1.41521	0.318948			0.673751	7.79007
FHL1	YBR126C	5.11649								5.11649
FHL1	YBR155W				1.1667		0.894302	1.68998	0.729339	4.48032
FHL1	YBR181C	5.57159	1.34787	4.99419	1.79161		1.09492		0.731982	15.5322
FHL1	YBR188C	6.98245	0.977661	5.57045						13.5306
FHL1	YBR189W	6.98245	0.977661		1.77733		1.11364		0.732326	11.5834
FHL1	YBR191W	6.28296	0.977661	4.82827	1.79257		1.01857		0.728157	15.6282
FHL1	YBR267W				1.19354	1.29418	0.924101		0.728902	4.14072
FHL1	YCL036W	3.48331	0.811116	2.71317					0.502124	7.50972
FHL1	YCL037C	3.48331	0.811116	3.94225	1.0026				0.728682	9.96796
FHL1	YCL059C				1.76984	1.50442	0.995344		0.730058	4.99966
FHL1	YCR031C	0.529159	0.133183	0.521321	1.7034		1.11775		0.730243	4.73506
FHL1	YDL043C	0.350418	1.34787	0.969117				1.42355		4.09095
FHL1	YDL055C	0.21157	1.58189		1.76955		0.975358		0.731108	5.26948
FHL1	YDL060W	5.55474	1.73754	2.68821	0.395197	0.858694	0.806293		0.72994	12.7706
FHL1	YDL061C	5.55474	1.73754	6.54009	1.64246		1.02473		0.732364	17.2319
FHL1	YDL063C	5.55474	1.73754		0.65122	0.954296			0.725003	9.6228
FHL1	YDL075W	6.74503	0.504391	1.28276	1.64448		0.98354		0.72997	11.8902
FHL1	YDL076C	6.74503	0.504391	3.89135		0.534959				11.6757
FHL1	YDL081C	1.18701			1.68815	1.73499			0.731102	5.34125
FHL1	YDL082W	7.19813	1.58189		1.69577		0.904006		0.731111	12.1109
FHL1	YDL083C	7.19813	1.58189	1.26675	1.76515		1.10728	1.35038	0.729148	14.9987
FHL1	YDL129W	6.12438	1.73754		0.603822				0.716712	9.18245
FHL1	YDL130W	6.12438	1.73754	1.42407	1.43996	1.73499	0.988348			13.4493
FHL1	YDL133C-A	7.19261	1.72904							8.92165
FHL1	YDL133W	7.19261	1.72904	4.50966	0.454742					13.8861
FHL1	YDL134C	7.19261	1.72904			1.50043	0.973462			11.3955
FHL1	YDL136W	5.56668	1.22714		1.59291		0.94		0.732158	10.0589
FHL1	YDL182W	1.58327			1.45175			0.878688	0.732343	4.64605
FHL1	YDL184C	6.38499	0.832277		1.34721				0.713761	9.27824
FHL1	YDL191W	6.3374	0.599937		1.65807		0.94		0.729949	10.2654
FHL1	YDR025W	6.42487			1.81236		1.11328		0.732106	10.0826
FHL1	YDR037W		1.33822	1.43921	1.20056				0.73173	4.70972
FHL1	YDR046C	0.750184		6.62188					0.593958	7.96602
FHL1	YDR047W	0.750184		7.13149						7.88167
FHL1	YDR064W	6.22775			1.70693		1.11339		0.732202	9.78027
FHL1	YDR065W	6.22775								6.22775
FHL1	YDR192C	1.79164	1.10545			1.06498	1.01426			4.97633
FHL1	YDR300C		0.649233	1.46472	1.03905		0.856548		0.732631	4.74218
FHL1	YDR324C	0.062553		2.0517	0.794178		0.908679		0.732666	4.54978
FHL1	YDR382W				1.71466	1.73499	0.83585		0.727919	5.01342
FHL1	YDR384C	3.90933			0.45392				0.711197	5.07445
FHL1	YDR385W	3.90933			1.40995	1.06498			0.731707	7.11597
FHL1	YDR387C		0.816414	2.48227			0.933549			4.23223
FHL1	YDR418W	6.76743	0.811116	13.109	1.79858		1.11439		0.731789	24.3323
FHL1	YDR447C	6.46467	1.58189	3.69978	1.70093		1.09492		0.723762	15.266
FHL1	YDR448W	6.46467	1.58189	5.31188		1.22991	0.973514			15.5619
FHL1	YDR449C	5.99611	1.73754	5.50935	1.54361				0.732725	15.5193
FHL1	YDR450W	5.99611	1.73754	2.59343	1.73543		1.05682		0.72879	13.8481
FHL1	YDR470C	7.00698	0.649233	1.50595						9.16216
FHL1	YDR471W	7.00698	0.649233		1.77356				0.729612	10.1594
FHL1	YDR500C	4.87072	0.421733		1.76722		1.03794		0.728007	8.82562
FHL1	YDR501W	5.40406	0.421733	3.84444						9.67023
FHL1	YEL007W	3.19061	0.569905	1.07422						4.83474
FHL1	YEL009C	5.16616	0.569905	2.40788	1.0155	1.58045			0.682819	11.4227
FHL1	YEL034W	0.456029	0.278	0.384754	1.0324	0.442809	1.08846		0.456537	4.13899
FHL1	YEL054C	7.03713	0.649233	3.696	1.78859	1.64022	1.11439		0.729125	16.6547
FHL1	YEL056W		1.50113	7.36876			0.955591			9.82548
FHL1	YER012W	0.40984			1.00507	1.7764	1.09177			4.28308
FHL1	YER031C		0.822294	2.95066	0.819862		0.850752			5.44357
FHL1	YER056C-A	6.79232	1.58189	0.214416	1.77877		0.930578		0.729495	12.0275
FHL1	YER058W	6.79232	1.58189							8.37421
FHL1	YER074W	7.04288	0.649233	0.600341	1.79903		1.02176		0.731944	11.8452
FHL1	YER074W-A	7.04288	0.649233							7.69211
FHL1	YER082C				1.43509	1.26343	0.954454		0.73215	4.38512
FHL1	YER088C	3.29762				1.4656				4.76322
FHL1	YER094C				1.23736	1.7764	1.06403			4.07779
FHL1	YER101C	6.25023	0.0448876	1.70854						8.00366
FHL1	YER102W	6.25023	0.0448876	0.590982	1.83101		1.06109		0.732364	10.5106
FHL1	YER115C	7.10697	1.50113							8.6081
FHL1	YER116C	7.10697	1.50113	6.81774			1.00082			16.4267
FHL1	YER117W	7.10697	1.50113	4.08877	1.7519		0.853225		0.732551	16.0345
FHL1	YER126C				1.62393	1.18359	1.05493		0.732654	4.5951
FHL1	YER130C	3.76713	0.504391	0.55902						4.83054
FHL1	YER131W	3.76713	0.504391	0.271435	1.70878		0.827169		0.732701	7.81161
FHL1	YER146W	1.87069			1.29317		1.08425		0.557424	4.80553
FHL1	YER148W				1.76964	1.58045	1.06504			4.41513
FHL1	YER165W			0.584817	0.872948	1.06498	0.912943		0.732326	4.16801
FHL1	YFL034C-A	6.08988	1.95632		1.12465					9.17085
FHL1	YFL034C-B	6.08988	1.95632							8.0462
FHL1	YFL034W	6.08988	1.95632	1.61968						9.66588
FHL1	YFR031C	6.69218	1.95632							8.6485
FHL1	YFR031C-A	6.69218	1.95632		1.79788	1.29418			0.730116	12.4707
FHL1	YFR032C	4.32803	1.73754		0.852866					6.91844
FHL1	YFR032C-A	4.32803	1.73754	5.86454	1.3925					13.3226
FHL1	YGL008C		1.58189	1.91315	0.975482				0.721277	5.1918
FHL1	YGL014W	2.23516			0.723884		0.983222		0.719972	4.66224
FHL1	YGL029W		1.34787	0.382858	1.69185				0.732208	4.15479
FHL1	YGL030W	5.35828	1.34787		1.76071	1.18359	1.03545		0.73004	11.4159
FHL1	YGL031C	5.35828	1.34787	5.96972	1.72842		0.980965		0.730011	16.1153
FHL1	YGL074C		1.34787	3.65189						4.99976
FHL1	YGL076C	1.011	0.649233	0.312321	1.79185		1.06826		0.732293	5.56496
FHL1	YGL083W		1.95632	3.0249			0.965656			5.94688
FHL1	YGL084C		1.95632	2.09996						4.05628
FHL1	YGL099W	6.11004			1.44945	1.45133	1.07954		0.732637	10.823
FHL1	YGL100W	6.11004								6.11004
FHL1	YGL101W	6.61771			0.601179				0.728626	7.94752
FHL1	YGL103W	6.61771		1.08532	1.60205		1.0446		0.723774	11.0735
FHL1	YGL104C	6.61771		1.07467						7.69238
FHL1	YGL123W	5.78509	0.308971	8.21246	1.81838		1.03058		0.732364	17.8878
FHL1	YGL124C	5.78509	0.308971	6.28247	0.742261			1.39545		14.5142
FHL1	YGL134W	6.61118	1.32318		0.709051					8.64341
FHL1	YGL135W	6.61118	1.32318	9.22192	1.76739		1.08785		0.731883	20.7434
FHL1	YGL136C	6.61118	1.32318	10.6807						18.6151
FHL1	YGL145W	6.90772								6.90772
FHL1	YGL147C	6.90772			1.82549		1.10039		0.732443	10.566
FHL1	YGL188C-A	3.51923	1.58189							5.10112
FHL1	YGL189C	6.16824	1.58189	2.96633	1.71245		0.827169		0.728682	13.9848
FHL1	YGL190C	6.16824	1.58189		0.647563	1.77697				10.1747

TF	Target	Binding_0-2000	Motif_Score_SC	Other_Yeasts	Two-Hop_BE	Two-Hop_BP	Two-Hop_BY	Two-Hop_BG	Max_Membership	Sum
FHL1	YGR010W			3.73676	0.595226					4.33199
FHL1	YGR020C	1.36175			0.365984	1.30948	0.878189		0.469639	4.38504
FHL1	YGR027C	4.83544	1.07799	5.58155	1.76122				0.732085	13.9883
FHL1	YGR033C	5.77477	1.32318	0.968445	0.698345					8.76474
FHL1	YGR034W	5.77477	1.32318		1.80326		1.09648		0.732578	10.7303
FHL1	YGR081C	1.73632			1.00406	1.14428			0.705412	4.59007
FHL1	YGR084C	6.16752	1.10545		0.930934					8.2039
FHL1	YGR085C	6.16752	1.10545	2.43024	1.78199		1.06396		0.730832	13.28
FHL1	YGR090W	1.23852			0.999612	1.50442			0.720397	4.46295
FHL1	YGR094W		0.934609	2.9943	1.46661				0.729665	6.12518
FHL1	YGR103W		1.10545		1.54505	1.45133			0.731419	4.83325
FHL1	YGR117C	6.62603	0.278	2.93154	0.803621					10.6392
FHL1	YGR118W	6.62603	0.278		1.75526	0.36962	1.10642		0.732026	10.8674
FHL1	YGR147C	5.44936	1.07799							6.52735
FHL1	YGR148C	5.44936	1.07799	4.91639	1.72544		0.980965		0.73119	14.8813
FHL1	YGR149W	5.44936	1.07799	6.68706						13.2144
FHL1	YGR159C		0.599937	1.56615	0.823926		0.987713	1.53423	0.677433	6.18939
FHL1	YGR213C	5.27608		1.4983						6.77438
FHL1	YGR214W	5.27608			1.78515		1.11263		0.729189	8.90305
FHL1	YGR240C	0.969426	0.234486		0.516596	1.3099	0.978243			4.00865
FHL1	YGR260W		1.09369	2.40149	1.2941		0.817643			5.60692
FHL1	YHL001W	4.78234	0.784553		1.79517		0.980965		0.729365	9.07239
FHL1	YHL015W	4.0334	0.421733	5.67054	1.62755		1.10906		0.732108	13.5944
FHL1	YHL016C	4.08497	0.421733	0.331944						4.83865
FHL1	YHL033C	5.1076	0.632241	0.318263	1.78588		1.11651		0.730565	9.69106
FHL1	YHL036W		1.33822	2.31907			0.903073			4.56036
FHL1	YHL038C		1.33822	3.25473						4.59295
FHL1	YHR009C		1.50113	4.32516						5.82629
FHL1	YHR010W		1.50113	0.0543381	1.83046				0.731542	4.11747
FHL1	YHR019C	2.28829			1.43684				0.732469	4.4576
FHL1	YHR020W	2.28829			1.68239		1.06241		0.732205	5.7653
FHL1	YHR021C	6.75056	0.627377	1.6802	1.4166		1.09492		0.729462	12.2991
FHL1	YHR021W-A	6.75056	0.627377	0.290647						7.66858
FHL1	YHR038W			5.59032		1.1676	0.951328			7.70925
FHL1	YHR141C	6.81264	1.58189		1.7404		1.07203		0.732431	11.9394
FHL1	YHR142W	6.81264	1.58189	2.4699					0.578254	11.4427
FHL1	YHR203C	6.64039	1.34787	1.85829	1.83344		1.09492		0.73173	13.5066
FHL1	YHR204W	6.64039	1.34787	1.5584	0.76036	0.837479			0.63933	11.7838
FHL1	YIL018W	6.75661	0.798736		1.769				0.730612	10.055
FHL1	YIL020C	6.75661	0.798736		0.982976				0.71142	9.24974
FHL1	YIL053W	4.97878	1.35269	1.44363		1.0229			0.728125	9.52613
FHL1	YIL055C	4.97878	1.35269	1.36588						7.69735
FHL1	YIL069C	6.92465	0.133183		1.41262		1.02176		0.646292	10.1385
FHL1	YIL070C	6.92465	0.133183		1.09955	0.326677				8.48406
FHL1	YIL133C	6.92239	0.00633055	0.338933	1.76295		1.1135	1.40007	0.732757	12.2769
FHL1	YIL148W	7.02428	1.73754	0.709413	1.6919		0.913516		0.72265	12.7993
FHL1	YIL149C	7.02428	1.73754	3.0505			0.885089	1.17215		13.8696
FHL1	YJL001W				1.14659	1.7764	1.09599			4.01898
FHL1	YJL134W	6.47138	1.72904	5.83481	0.728211					14.7634
FHL1	YJL136C	6.47138	1.72904		1.7853				0.728163	10.7139
FHL1	YJL137C	6.47138	1.72904		0.496108		0.846343			9.54287
FHL1	YJL138C	0.918372	0.133183		1.57219	1.02597		0.887594	0.638221	5.17553
FHL1	YJL148W	1.388	0.67161		1.52342				0.729885	4.31292
FHL1	YJL177W	6.57764	1.58189	0.250565	1.82047		1.09629		0.732323	12.0592
FHL1	YJL189W	6.85581	0.44942		1.49223		1.04677		0.72745	10.5717
FHL1	YJL190C	6.85581	0.44942	5.23691	1.73601		1.11352		0.731944	16.1236
FHL1	YJL191W	6.89898			1.49182		1.11775		0.7041	10.2127
FHL1	YJL192C	6.89898			0.672013					7.57099
FHL1	YJL198W	3.72861			0.8932				0.73261	5.35442
FHL1	YJL200C	3.72861		0.925616	0.731459				0.732601	6.11829
FHL1	YJR047C			1.50846	0.767136		1.08846		0.723454	4.08751
FHL1	YJR094W-A	0.964935	1.34787	1.14038	1.7714		1.0424		0.732642	6.99963
FHL1	YJR104C	2.37469			1.22312		0.913919			4.51173
FHL1	YJR105W	2.37469			1.00909		0.825012			4.20879
FHL1	YJR123W	7.29995	0.798736	5.77395	1.80626		1.1004		0.731921	17.5112
FHL1	YJR138W		0.70591	1.39954		1.3099	0.890957			4.30631
FHL1	YJR139C		1.72904	3.44067					0.651441	5.82115
FHL1	YJR145C	5.66916	0.902249	3.08374	1.68724		1.09492		0.7321	13.1694
FHL1	YJR147W	5.66916	0.902249	9.32741						15.8988
FHL1	YKL006C-A	5.15795	1.33822							6.49617
FHL1	YKL006W	5.15795	1.33822		1.76081		0.980965		0.731492	9.96944
FHL1	YKL009W				1.65965	0.917651	1.05989		0.732742	4.36993
FHL1	YKL021C				1.26175		0.836735	1.1862	0.732414	4.0171
FHL1	YKL040C		1.06786	3.67929						4.74715
FHL1	YKL060C			3.06628	0.915546		0.96321			4.94504
FHL1	YKL081W		0.166932	0.40093	1.59636	1.3099	0.914947		0.716437	5.10551
FHL1	YKL156W	5.69443	0.205071		1.36368		1.09492		0.730947	9.08905
FHL1	YKL172W				1.52407	1.69447	0.818045		0.727602	4.76419
FHL1	YKL180W	6.38683	1.73754	4.4949	1.76352	0.434194	1.08952		0.731451	16.638
FHL1	YKR057W	3.06348	0.977661		1.77985				0.731806	6.5528
FHL1	YKR058W	3.06348	0.977661				1.06801			5.10915
FHL1	YKR059W				1.51214	1.02597		0.887594	0.731665	4.15737
FHL1	YKR092C	1.82003	0.822294	1.34396	1.11495				0.706814	5.80805
FHL1	YKR094C	3.77105			1.80712		0.913516		0.732378	7.22406
FHL1	YKR095W	3.77105		1.73181			0.902975	1.17209		7.57792
FHL1	YLL006W	4.46977								4.46977
FHL1	YLL007C	4.46977								4.46977
FHL1	YLL045C	3.17109	0.784553	3.78987	1.72062		1.11651		0.732701	11.3153
FHL1	YLR003C		0.632241	1.32882	1.76379				0.732566	4.45742
FHL1	YLR009W		0.374226		1.67471	0.326677	1.09492		0.732537	4.20307
FHL1	YLR028C	5.48609	0.977661							6.46375
FHL1	YLR029C	5.48609	0.977661	1.10905	1.64671		1.11339		0.729166	11.0621
FHL1	YLR030W	5.48609	0.977661	1.20639						7.67014
FHL1	YLR031W	5.48609	0.977661	1.31908						7.78283
FHL1	YLR047C	6.78086	1.73754	9.79903						18.3174
FHL1	YLR048W	6.78086	1.73754		1.7817		1.11467		0.729935	12.1447
FHL1	YLR060W	1.13893			1.3288		1.07435		0.684477	4.22656
FHL1	YLR061W	7.08075	1.34787		1.78415				0.726892	10.9397
FHL1	YLR063W	7.08075	1.34787		0.7552				0.726197	9.91002
FHL1	YLR073C	4.71523	0.785737						0.702997	6.20396
FHL1	YLR074C	4.71523	0.785737	6.38062	1.07554	0.923115			0.600685	14.4809
FHL1	YLR075W	4.71523	0.785737	5.01544	1.5396		1.08092		0.726646	13.8636
FHL1	YLR077W	4.71523	0.785737							5.50097
FHL1	YLR166C	5.33302		3.77196						9.10498
FHL1	YLR167W	5.33302		4.85985	1.62902		0.928987		0.732273	13.4831
FHL1	YLR183C	6.88881	1.50113	7.27097	0.894379				0.601867	17.1572
FHL1	YLR185W	6.88881	1.50113		1.74081		1.03794		0.727582	11.8963
FHL1	YLR186W	6.88881	1.50113		1.29036		1.04862		0.725587	11.4545
FHL1	YLR249W		0.902249	1.57118	1.48546	1.3099	0.828788		0.732763	6.83034
FHL1	YLR264W	2.18595			1.70753		1.03123		0.726769	5.65148

TF	Target	Binding_0-2000	Motif_Score_SC	Other_Yeasts	Two-Hop_BE	Two-Hop_BP	Two-Hop_BY	Two-Hop_BG	Max_Membership	Sum
FHL1	YLR287C	5.49889	1.73754		1.07131	1.11134			0.732437	10.1515
FHL1	YLR287C-A	5.49889	1.73754	3.69168	1.34985				0.566434	12.8444
FHL1	YLR289W	5.49889	1.73754							7.23643
FHL1	YLR325C	5.71769	1.07799	3.42919	1.82021				0.728591	12.7737
FHL1	YLR326W	5.71769	1.07799	4.35618						11.1519
FHL1	YLR333C	4.5552		0.727112	1.44964				0.718784	7.45074
FHL1	YLR335W	4.5552			1.02056	0.890624			0.662597	7.12898
FHL1	YLR337C	5.14542	0.816414	0.832692						6.79453
FHL1	YLR340W	5.14542	0.816414	1.42527	1.51682	1.11134	1.0686		0.730078	11.8139
FHL1	YLR344W	4.84482	1.73754	0.469973	1.74297		1.09648		0.730782	10.6226
FHL1	YLR345W	4.84482	1.73754		0.526985					7.10935
FHL1	YLR367W	3.22273			1.69699		1.11352		0.729524	6.76276
FHL1	YLR372W		0.917296	1.29265	1.55512				0.732009	4.49707
FHL1	YLR387C	4.07577		0.90117	0.978966		0.924101			6.88001
FHL1	YLR388W	4.07577			1.75757		1.02473		0.7297	7.58777
FHL1	YLR399C	1.81674	1.95632	2.6123			0.852934			7.23829
FHL1	YLR406C	5.49039	1.73754	0.630546	1.12661		1.04063		0.702238	10.728
FHL1	YLR407W	5.49039	1.73754	3.92522					0.610551	11.7637
FHL1	YLR440C	5.2036								5.2036
FHL1	YLR441C	5.2036		0.00497938	1.82741		1.10751		0.73224	8.87574
FHL1	YLR447C	6.16671	0.627377	2.93337	0.431668				0.688311	10.8474
FHL1	YLR448W	6.16671	0.627377	1.41005	1.758		0.975354		0.728588	11.6661
FHL1	YML022W	1.15893			1.65248		0.78969		0.732607	4.33371
FHL1	YML024W	6.7541	1.73754	2.47698	1.76153		1.09492		0.730225	14.5553
FHL1	YML025C	6.7541	1.73754	6.64982	1.13439		0.99149			17.2673
FHL1	YML026C	6.56926	1.73754	0.916845	1.74756		1.05682		0.730894	12.7589
FHL1	YML062C	4.30705								4.30705
FHL1	YML063W	6.58117	1.09369	1.8793	1.80004		1.10751		0.730694	13.1924
FHL1	YML064C	6.58117	1.09369	2.84925		0.389506				10.9136
FHL1	YML073C	6.77033	0.977661		1.78271		0.975354		0.728154	11.2342
FHL1	YMR013C	4.30053	1.58189	1.25201					0.471388	7.60582
FHL1	YMR013W-A	4.30053	1.58189							5.88242
FHL1	YMR014W	0.312933	1.58189	3.71225	1.4335				0.731704	7.77228
FHL1	YMR092C		1.73754	3.38876	0.499664		0.972283			6.59825
FHL1	YMR093W		1.73754	5.94326	1.1799		0.92563	1.20961	0.732654	11.7286
FHL1	YMR116C	3.89917	1.73754	3.34427	1.5572				0.730888	11.2691
FHL1	YMR121C	0.543321		1.22663	1.52059		1.11339		0.729028	5.13296
FHL1	YMR142C	6.50599	1.58189	1.04455	1.80825		0.904006		0.731648	12.5763
FHL1	YMR143W	0.228274	1.58189		1.8046		1.10728	1.35038	0.730342	6.80277
FHL1	YMR194W	5.79715	1.95632	3.56493	1.57754				0.731663	13.6276
FHL1	YMR205C		1.95632	5.05739			0.956824			7.97053
FHL1	YMR206W		1.95632	4.25272						6.20904
FHL1	YMR229C	5.26851	0.832277	0.627036	1.10771		1.03472		0.727693	9.59795
FHL1	YMR230W	5.26851	0.832277		1.75171				0.731707	8.5842
FHL1	YMR242C		0.601159	2.50072	1.81181				0.728265	5.64195
FHL1	YNL002C				1.77896	0.917651	0.798087		0.732211	4.22691
FHL1	YNL016W			1.13984	0.474298		1.03347	1.65954		4.30715
FHL1	YNL030W	0.77651			1.52858	1.3099	0.93257		0.631177	5.17874
FHL1	YNL067W	3.89277			1.72861		1.10419		0.731789	7.45736
FHL1	YNL069C	6.55573	0.70591		1.77848		1.11801	1.37865	0.731454	12.2682
FHL1	YNL096C	6.63049	0.977661		1.77205		0.848915		0.732584	10.9617
FHL1	YNL101W		0.643777	4.23696			0.929702			5.81044
FHL1	YNL110C				1.73769	0.917651	1.026	1.30518	0.732745	5.71927
FHL1	YNL162W	6.27106	0.270231		1.7391		1.07203		0.7309	10.0833
FHL1	YNL163C	6.27106	0.270231		0.563281				0.731601	7.83617
FHL1	YNL175C				1.36472	0.476036	0.876067	1.27247	0.731372	4.72067
FHL1	YNL178W	4.24945	0.288988	3.35818	1.81944		1.10752		0.732428	11.556
FHL1	YNL180C	4.24945	0.288988		0.424078		0.983222			5.94574
FHL1	YNL302C	6.78839	0.816414	0.380626	1.56606		1.09492		0.730777	11.3772
FHL1	YOL038W	6.686	0.70591		0.445764		1.09146			8.92913
FHL1	YOL039W	6.686	0.70591	6.22793	1.68508	1.73499	0.964285		0.732698	18.7369
FHL1	YOL040C	6.686	0.70591	6.31426	1.74143		1.11284		0.732507	17.2929
FHL1	YOL041C	6.686	0.70591		1.41857	0.553302	0.821144		0.731812	10.9167
FHL1	YOL086C	1.69787	0.798736	1.16133	0.514935		1.00223			5.1751
FHL1	YOL109W	2.59683	0.805814	7.33806	1.36532		0.871015			12.977
FHL1	YOL111C	2.59683	0.805814	6.164						9.56664
FHL1	YOL120C	5.50216	0.902249	1.59118	1.75022		0.907645		0.732047	11.3855
FHL1	YOL121C	6.40342	1.50113	5.39509	1.76499		1.09492		0.722269	16.8818
FHL1	YOL122C	6.40342	1.50113							7.90455
FHL1	YOL127W	6.02483	1.95632	1.54801	1.7506		1.11909		0.717543	13.1164
FHL1	YOL128C	6.02483	1.95632	11.5325						19.5136
FHL1	YOR007C	0.679733		0.342166	1.15429		0.99434	1.54275		4.71328
FHL1	YOR056C		0.029361		1.23778	1.14428	0.919538		0.731352	4.06231
FHL1	YOR095C	4.85537	0.632241	0.93083	1.41283				0.730202	8.56147
FHL1	YOR096W	4.85537	0.632241	0.50121	1.80439		0.848915		0.730703	9.37283
FHL1	YOR145C		0.816414		1.56554	1.14428	1.09492		0.732496	5.35365
FHL1	YOR161C	3.59942	1.72904	1.5351	0.390231					7.25379
FHL1	YOR167C	0.613201			1.7199		1.03123		0.728582	4.09291
FHL1	YOR182C	6.40372	1.73754		1.64647		0.868992		0.711405	11.3681
FHL1	YOR184W	6.40372	1.73754	7.739			0.880769			16.761
FHL1	YOR234C	6.30111	0.0448876	1.17243	1.73192		0.887588		0.728391	10.8663
FHL1	YOR236W	6.30111	0.0448876	1.99028			0.95867		0.627343	9.92229
FHL1	YOR272W		0.365951		1.35658	0.830391	0.935236	1.36786	0.720089	5.57611
FHL1	YOR292C	6.88704	0.93115	2.53947						10.3577
FHL1	YOR293W	6.88704	0.93115		1.78501				0.732032	10.3352
FHL1	YOR294W	6.88704	0.93115		1.36162	1.7764			0.731164	11.6874
FHL1	YOR311C	6.78749	1.95632							8.74381
FHL1	YOR312C	6.78749	1.95632	6.36097	1.79008				0.730976	17.6258
FHL1	YOR326W				0.700741	1.51409	1.02618	1.34939	0.468049	5.05845
FHL1	YOR340C	0.917127	1.33822	2.58965	1.24518				0.698254	6.78843
FHL1	YOR341W	0.917127	1.33822	1.16218	0.74028				0.560824	4.71863
FHL1	YOR344C	3.88018							0.610278	4.49046
FHL1	YOR354C	5.78874		0.381231						6.16997
FHL1	YOR355W	5.78874			0.583257				0.530816	6.90281
FHL1	YOR369C	5.97186	1.73754	6.54064	1.78035		1.04016		0.732475	17.803
FHL1	YPL043W				1.47615	1.44967		1.70167	0.730847	5.35834
FHL1	YPL079W	6.28648	0.902249	1.0294	1.80131		1.01857		0.732411	11.7704
FHL1	YPL081W	2.35562	0.902249		1.4984		1.11364		0.715401	6.58531
FHL1	YPL082C	2.35562	0.902249	1.43748						4.69535
FHL1	YPL090C	7.0856	1.73754		1.78748		1.09492		0.732003	12.4375
FHL1	YPL093W				1.52184	1.45133	1.10549		0.7308	4.80946
FHL1	YPL130W	5.79101	0.178138							5.96915
FHL1	YPL131W	5.79101	0.178138	0.886004	1.6803	0.890513	1.09492		0.732601	11.2535
FHL1	YPL133C	5.79101	0.178138		0.50037					6.46952
FHL1	YPL143W	5.2505	0.258558		1.7106		0.887588		0.731055	8.8383
FHL1	YPL144W	0.138282	0.811116	3.22318					0.607124	4.7797
FHL1	YPL145C	0.138282	0.811116	3.2296	0.652915					4.83191
FHL1	YPL198W	5.79675	0.977661		1.78993		1.06826		0.725393	10.358
FHL1	YPL199C	5.79675	0.977661	3.38241	0.962654					11.1195
FHL1	YPL201C	5.79675	0.977661		0.52408	0.802081			0.610504	8.71108

TF	Target	Binding_0-2000	Motif_Score_SC	Other_Yeasts	Two-Hop_BE	Two-Hop_BP	Two-Hop_BY	Two-Hop_BG	Max_Membership	Sum
FHL1	YPL220W	0.962175		1.74814	1.76309		1.08785		0.730958	6.29221
FHL1	YPL249C-A	6.55024	1.95632	10.8648	1.32853					20.6999
FHL1	YPL250C	6.55024	1.95632							8.50656
FHL1	YPL263C				1.01955		0.983532	1.8208	0.724865	4.54875
FHL1	YPR016C		0.93115		0.676555	1.29418	1.10007		0.732129	4.73408
FHL1	YPR041W				1.08522	1.29418	1.01416		0.732458	4.12602
FHL1	YPR042C	6.59217	0.977661	3.99122			1.06126			12.6223
FHL1	YPR043W	6.59217	0.977661		1.77459		1.0424		0.732569	11.1194
FHL1	YPR080W	4.5964		0.144735	1.30018				0.70474	6.74606
FHL1	YPR102C	7.19051	0.569905	2.54943	1.82861	1.69447	1.06396		0.730832	15.6277
FHL1	YPR103W	7.19051	0.569905	1.82578			1.11325			10.6994
FHL1	YPR110C		0.518051	1.38754	1.47819	0.995453	1.10467		0.732402	6.21631
FHL1	YPR131C	6.53975	1.58189	1.69485			1.00243			10.8189
FHL1	YPR132W	6.53975	1.58189		1.74988		1.10642		0.732361	11.7103
FHL1	YPR161C	0.767065	1.33822	4.46901			1.03499			7.60928
FKH1	YBL081W	5.69405		0.638248						6.3323
FKH1	YBL082C	5.69405		0.990605	0.577096					7.26175
FKH1	YBR010W	1.38461		2.04209	1.01921					4.44591
FKH1	YBR029C	5.61735		0.318444			0.814954			6.75075
FKH1	YBR030W	5.61735		0.316435	0.589793					6.52358
FKH1	YBR104W	3.96661		0.568087						4.5347
FKH1	YBR109C	2.45307			0.500697	1.25697	0.993487			5.20422
FKH1	YBR138C	3.78594		2.67254						6.45848
FKH1	YBR139W	3.78594		3.3255						7.11144
FKH1	YBR158W	1.90773		1.23939	0.752084				0.685207	4.58441
FKH1	YBR162C	4.96705			1.45053					6.41758
FKH1	YBR163W	4.96705								4.96705
FKH1	YBR267W			2.54405	0.979478		0.927005			4.45053
FKH1	YCL027W	1.31481		2.43242	0.373746	0.381094				4.50207
FKH1	YCL059C	0.145509			1.35009	1.55325	1.04335		0.440085	4.53228
FKH1	YCL064C	2.70846		1.92858						4.63704
FKH1	YCR075C	6.68817		0.797504						7.48567
FKH1	YCR084C	0.648545		5.24999						5.89854
FKH1	YCR086W	0.648545		4.90327						5.55182
FKH1	YCR092C	4.00942			0.397355					4.40678
FKH1	YCR093W	4.00942								4.00942
FKH1	YDL101C	0.245505		3.36941		0.430386				4.0453
FKH1	YDR263C	4.86615		0.644702			1.06654			6.57739
FKH1	YDR324C	7.06971		0.294267	1.2824					8.64638
FKH1	YDR325W	7.06971		2.58565						9.65536
FKH1	YDR365C			3.25373	1.03919					4.29292
FKH1	YDR524C			4.09687				0.922342		5.01921
FKH1	YDR525W-A			6.46758						6.46758
FKH1	YEL050C			4.21569						4.21569
FKH1	YER031C	2.40579		2.45249						4.85828
FKH1	YER032W	2.40579		1.85469						4.26048
FKH1	YER062C	3.24845		0.933478						4.18193
FKH1	YER064C	1.21541		4.34166						5.55707
FKH1	YER065C			4.26045						4.26045
FKH1	YER070W	6.15912		3.38206	0.482096					10.0233
FKH1	YER094C	1.67046		0.670659	1.0524	0.517114	1.02837			4.939
FKH1	YER124C	5.09941		2.29346	1.15866				0.620921	9.17245
FKH1	YER125W	5.09941		2.03643		0.381094				7.51693
FKH1	YFR003C	5.26638								5.26638
FKH1	YFR004W	5.26638			0.590753	0.538145				6.39528
FKH1	YFR023W	4.54443								4.54443
FKH1	YFR052W	3.30942			0.990508	1.06643				5.36636
FKH1	YGL011C			1.30926	1.2035	0.517114	0.992733			4.02261
FKH1	YGL028C	2.74995		3.84281	1.37237				0.710135	8.67526
FKH1	YGL092W	5.55995								5.55995
FKH1	YGL116W	3.89096				0.724114	1.0454			5.66047
FKH1	YGL256W	4.79219		0.206043						4.99823
FKH1	YGL257C	4.79219		0.329254						5.12144
FKH1	YGR052W	4.21978			0.486754					4.70653
FKH1	YGR092W	3.97311		1.93491						5.90802
FKH1	YGR098C	5.44479		2.8116						8.25639
FKH1	YGR099W	5.44479		3.8488						9.29359
FKH1	YGR246C			3.39724			0.826577			4.22382
FKH1	YHR118C	2.80368		3.07461						5.87829
FKH1	YHR143W	6.37121		1.79766	0.898864				0.697071	9.7648
FKH1	YHR158C			1.78151			1.09607	1.86646		4.74404
FKH1	YHR200W	3.23151		2.39365		1.06643				6.69159
FKH1	YIL015W	2.42441					0.921806	1.19977		4.54599
FKH1	YIL076W	3.68289				0.538145				4.22103
FKH1	YJL078C	1.83058			1.03605		0.939457	1.62433		5.43042
FKH1	YJL189W	4.92199			0.624557					5.54655
FKH1	YJL190C	4.92199			1.27862		1.00605			7.20666
FKH1	YJR088C	0.496458		4.01938						4.51584
FKH1	YJR091C	4.34384		0.413753		0.901041				5.65863
FKH1	YKL069W	4.64466		0.114186						4.75885
FKH1	YKL159C	5.30791								5.30791
FKH1	YKR054C	7.09875		1.05205						8.1508
FKH1	YKR055W	7.09875		3.44844						10.5472
FKH1	YKR056W	7.09875		1.09356	1.35449					9.5468
FKH1	YKR059W	5.9115			1.2576			0.888962		8.05806
FKH1	YKR060W	5.9115			1.59701					7.50851
FKH1	YLL004W			3.70995		0.592152				4.3021
FKH1	YLL032C			5.24317						5.24317
FKH1	YLR131C	3.35543		2.5265	0.496144					6.37807
FKH1	YLR167W			2.53042	1.07791			1.0356		4.64393
FKH1	YLR300W	3.88952		1.15358						5.0431
FKH1	YLR353W	4.03378								4.03378
FKH1	YLR399C	3.91674		0.729055						4.64579
FKH1	YLR453C	0.17828		4.43612						4.6144
FKH1	YLR454W	0.17828		3.96966			0.980736			5.12868
FKH1	YML026C			2.27749	1.21514		0.829773		0.59918	4.92158
FKH1	YML080W			3.97505	1.54253					5.51758
FKH1	YMR078C	2.5401			0.929506		1.02786			4.49747
FKH1	YMR086W	4.03995								4.03995
FKH1	YMR117C	2.78213		0.594141	0.780428					4.1567
FKH1	YMR183C	6.15824		1.4912			1.00734			8.65678
FKH1	YMR184W	6.15824		2.60014						8.75838
FKH1	YMR185W	6.15824			0.685302					6.84354
FKH1	YMR195W			4.31996						4.31996
FKH1	YMR197C	7.25826		4.20446						11.4627
FKH1	YMR198W	7.25826		4.30125			0.878191			12.4377
FKH1	YMR199W	0.945436		3.50944						4.45488
FKH1	YNL091W	3.95465					0.87026			4.82491
FKH1	YNL093W	3.9719		0.0408538			0.859971			4.87272
FKH1	YNL161W			6.58365						6.58365

TF	Target	Binding_0-2000	Motif_Score_SC	Other_Yeasts	Two-Hop_BE	Two-Hop_BP	Two-Hop_BY	Two-Hop_BG	Max_Membership	Sum
FKH1	YNL176C	2.21497		3.18257						5.39754
FKH1	YNL218W	1.65776		1.5434			0.799335			4.00049
FKH1	YOL023W	4.60213								4.60213
FKH1	YOL031C	3.79313			0.334607					4.12774
FKH1	YOR198C			1.06872	1.01809		1.09525	1.85268		5.03474
FKH1	YOR311C	2.40123		3.79201						6.19324
FKH1	YOR312C	2.40123		1.49431	1.23395					5.12949
FKH1	YOR313C	4.93518			0.619911					5.55509
FKH1	YOR315W	4.93518		3.31163	0.619911				0.508411	9.37513
FKH1	YPL116W	2.61386		1.46252	0.358178					4.43456
FKH1	YPL117C	2.61386		1.95738	0.330238					4.90148
FKH1	YPL141C	4.23919		0.969117	0.724249				0.706048	6.6386
FKH1	YPL177C	4.2506					0.993487			5.24409
FKH1	YPL212C			2.93748	0.665189		0.98493			4.5876
FKH1	YPL226W	1.971		2.43728	0.464516					4.8728
FKH1	YPL227C	1.971		5.63264			0.820567			8.42421
FKH1	YPL242C			4.31312	0.641923	0.766023			0.61232	6.33339
FKH1	YPR093C			4.7637			0.993487			5.75719
FKH1	YPR119W	3.29252		4.64792	0.91763	0.885668	0.913272		0.660814	11.3178
FKH1	YPR141C			0.856182	0.599863	1.79468	1.08607			4.3368
FKH1	YPR143W			3.4263	1.17721					4.60351
FKH2	YAL028W	10.328	0.0435391	1.29545						11.667
FKH2	YAL029C	10.328	0.0435391	0.326825			1.51665	1.78475	0.697086	14.6969
FKH2	YAR002C-A					2.43737	1.73222			4.16959
FKH2	YAR018C	7.91473				0.992169			0.440601	9.3475
FKH2	YAR019C				0.786718	1.43647	1.93879			4.16198
FKH2	YBL031W	6.9922								6.9922
FKH2	YBL032W	6.9922		0.726045	0.492997		1.51578		0.592485	10.3195
FKH2	YBL033C	6.9922								6.9922
FKH2	YBL034C	6.9922		0.0311115						7.02331
FKH2	YBL038W					3.62268	1.00771			4.63039
FKH2	YBL063W			1.09653			1.50678	2.07775		4.68106
FKH2	YBL089W				2.12317	2.43737	1.77208			6.33262
FKH2	YBL097W		0.953018	9.28925						10.2423
FKH2	YBR009C	2.55998	0.272822	1.15286	1.41874	0.526363			0.729277	6.66004
FKH2	YBR010W	2.55998	0.272822	1.64258	1.38707	0.526363	1.51621		0.72791	8.63293
FKH2	YBR036C	11.1845								11.1845
FKH2	YBR037C	11.1845		2.24277						13.4273
FKH2	YBR038W	11.1845		2.14187	2.51633					15.8427
FKH2	YBR054W	2.69482			0.688126	2.73354				6.11649
FKH2	YBR066C					2.73354	1.70364			4.43718
FKH2	YBR077C	6.49961								6.49961
FKH2	YBR078W	6.49961			0.362553					6.86216
FKH2	YBR081C					2.43286	1.25945	0.959513		4.65182
FKH2	YBR088C				2.41643	0.362462	1.02943		0.726537	4.53486
FKH2	YBR092C	2.14722		2.13256	0.428434				0.701164	5.40938
FKH2	YBR093C	2.53872		2.14092	0.348461				0.696367	5.72447
FKH2	YBR104W	2.07807					1.51578		0.496309	4.09016
FKH2	YBR109C	0.57841				2.62337	2.00462			5.2064
FKH2	YBR135W	2.63076		0.233944		2.69467				5.55937
FKH2	YBR138C	8.6403		1.78382						10.4241
FKH2	YBR139W	8.6403		1.75788	0.624094		1.51665		0.598631	13.1376
FKH2	YBR158W	2.02116	0.421733	1.94467	2.29455		1.51578		0.722592	8.92049
FKH2	YBR162C	1.16672		0.395782	1.03598			2.39761		4.99609
FKH2	YBR162W-A	1.16672		0.364577	2.46406				0.518151	4.51351
FKH2	YBR175W						2.0454	2.99961	0.63591	5.68092
FKH2	YBR195C						2.09607	2.86118		4.95725
FKH2	YBR202W	0.133513		1.70324	2.81365		0.980529		0.69011	6.32104
FKH2	YCL014W		0.615197	1.37393			1.60407		0.66652	4.25972
FKH2	YCL057W	3.90651			0.535305					4.44182
FKH2	YCL059C	3.90651			0.884545	1.22309	1.00811		0.566994	7.58925
FKH2	YCL061C	3.90651			0.432628				0.657264	4.9964
FKH2	YCL063W	1.19224	0.496303	4.48186						6.1704
FKH2	YCL064C	1.19224	0.496303	6.3302					0.488403	8.50715
FKH2	YCR024C-A	2.5947			0.574465	0.587399			0.678566	4.43513
FKH2	YCR027C	1.2555					2.00462	1.39069		4.65081
FKH2	YCR084C	0.685252	0.52115	2.1395	1.46657	0.526363		1.18142		6.52025
FKH2	YCR086W	0.685252	0.52115	2.16964		3.48398				6.86002
FKH2	YDL010W			0.156043	1.43838		2.08593		0.709014	4.38937
FKH2	YDL014W	0.483053		0.634408	0.511278	0.699333	1.00939		0.72745	4.06491
FKH2	YDL017W	2.8321		0.14258			1.88877			4.86345
FKH2	YDL018C	2.8321		0.140074			1.78412			4.75629
FKH2	YDL070W	0.686331			0.55028	0.763621	1.51578	0.95877		4.47478
FKH2	YDL101C	2.54731	0.971367	8.70361		0.363392			0.656947	13.2426
FKH2	YDL192W				2.5499		2.00563			4.55553
FKH2	YDL239C					1.95539	0.993227	2.42017		5.36879
FKH2	YDR077W	3.50279		0.0191051	1.48577					5.00767
FKH2	YDR101C				0.914526	2.43286			0.730741	4.07813
FKH2	YDR107C	1.4675	0.303177	0.340205	1.48981		1.51578			5.11647
FKH2	YDR113C	1.4629				4.10231				5.56521
FKH2	YDR128W						2.08491	2.85364		4.93855
FKH2	YDR130C	0.81887		3.56572		0.553797				4.93839
FKH2	YDR144C	1.45618					2.00462		0.633026	4.09383
FKH2	YDR146C	9.45968	1.14889	8.42635	2.836		2.00462		0.725461	24.601
FKH2	YDR147W	9.45968	1.14889	8.15087			2.00549			20.7649
FKH2	YDR150W	3.81456					1.07496	2.23099		7.12051
FKH2	YDR179C	0.59662	0.419841	4.08759						5.10405
FKH2	YDR212W					3.3208			0.692753	4.01355
FKH2	YDR253C			2.74002			1.93654	3.39415		8.07071
FKH2	YDR259C	2.27493	0.325034	2.69703		0.413563				5.71056
FKH2	YDR263C	6.61064	0.501282	2.73513			1.03129			10.8783
FKH2	YDR267C					3.92827	2.05397			5.98224
FKH2	YDR295C	0.396067			0.797033		1.05064	1.78389		4.02763
FKH2	YDR312W	2.46409			1.25322				0.544809	4.26212
FKH2	YDR313C	1.79918				2.43737	1.73222			5.96877
FKH2	YDR324C	5.8467	1.14889	8.10434	0.562239	0.699333	2.03791		0.623007	19.0224
FKH2	YDR325W	5.8467	1.14889	7.41696	0.707346					15.1199
FKH2	YDR353W			3.55289					0.451688	4.00458
FKH2	YDR368W		0.52115	2.25435			1.23579			4.01129
FKH2	YDR402C	1.00881	0.631025	2.60769						4.24752
FKH2	YDR403W	1.00881	0.631025	4.61971						6.25955
FKH2	YDR412W				0.869728	2.69467			0.612754	4.17715
FKH2	YDR451C	9.97347	0.0435391	1.18815	1.24611		1.20761			13.6589
FKH2	YDR452W	9.97347	0.0435391	0.468562						10.4856
FKH2	YDR492W				2.22283	2.48972			0.726329	5.43888
FKH2	YDR500C	5.55436			1.37843		0.803099		0.708827	8.44472
FKH2	YDR501W	5.55436		1.59682						7.15118
FKH2	YDR524C			3.15382			1.51578			4.6696
FKH2	YDR525W-A			4.729						4.729
FKH2	YEL013W					3.24994	1.73834	1.46766		6.45594

TF	Target	Binding_0-2000	Motif_Score_SC	Other_Yeasts	Two-Hop_BE	Two-Hop_BP	Two-Hop_BY	Two-Hop_BG	Max_Membership	Sum
FKH2	YEL027W			5.81931			1.51755			7.33686
FKH2	YEL029C			3.40039	2.59158					5.99197
FKH2	YEL034W				3.14196		1.02323		0.705969	4.87116
FKH2	YEL050C		0.122554	1.90338	2.98393					5.00986
FKH2	YEL075C	0.711306				2.73354		1.00592		4.45077
FKH2	YER029C	2.6616	0.615197		0.329275	0.786092			0.703549	5.09571
FKH2	YER031C	2.6616	0.615197	7.13231						10.4091
FKH2	YER032W	2.6616	0.615197	5.89482	2.43362	3.92827				15.5335
FKH2	YER053C	0.960298		0.68214	1.08849		1.51578			4.24671
FKH2	YER066W						2.06958	2.93312		5.0027
FKH2	YER070W	4.1616			0.461815	0.654959			0.729022	6.0074
FKH2	YER078C	4.33815								4.33815
FKH2	YER079W	4.33815								4.33815
FKH2	YER080W	4.33815								4.33815
FKH2	YER082C				2.82181	1.22309	2.09607		0.696916	6.83789
FKH2	YER094C	3.59893					1.00338			4.60231
FKH2	YER095W	3.59893				0.54311	1.02128		0.45128	5.6146
FKH2	YER110C				1.1382	2.43286			0.506786	4.07785
FKH2	YER111C					3.13895	1.00003			4.13898
FKH2	YER124C	4.85124	1.14889	9.18592	0.854374				0.699157	16.7396
FKH2	YER125W	4.85124	1.14889	9.41666		2.43737	1.75828			19.6124
FKH2	YER127W	0.1917		2.80168	0.55834	0.699333			0.724366	4.97542
FKH2	YFL037W	0.182453	0.51619	0.790648		1.72287	1.72812		0.694369	5.63465
FKH2	YFL039C	0.182453	0.51619		1.59478	2.43286	1.51621			6.24249
FKH2	YFR023W	3.07538		0.145029			2.06802			5.28843
FKH2	YFR028C		1.14889	7.82945	1.5354	1.43647			0.67053	12.6207
FKH2	YFR029W		1.14889	7.51155						8.66044
FKH2	YGL001C	2.195			0.381859		1.19163		0.724161	4.49265
FKH2	YGL003C					3.13895	1.92694			5.06589
FKH2	YGL008C	2.78608		0.0516927	2.55261	0.535736			0.597854	6.52397
FKH2	YGL014W				2.21678		2.00462		0.701927	4.92333
FKH2	YGL021W	2.46166	0.78692	3.08788	2.73994		1.87315		0.732698	11.6822
FKH2	YGL028C	1.67591		4.33578	1.30131				0.710135	8.02313
FKH2	YGL049C		0.0664192	3.52803		1.53233	2.06302			7.1898
FKH2	YGL086W	0.945976		0.687039		4.10231	1.04228			6.7776
FKH2	YGL097W	0.745897			0.596924		1.51578	0.881998	0.682007	4.42261
FKH2	YGL106W					2.43286	2.00462	1.9853		6.42278
FKH2	YGL116W	15.8065			2.3062	3.13895	1.88402			23.1357
FKH2	YGL163C	2.22438				0.806831		1.13507		4.16628
FKH2	YGL174W		0.78692	5.79223			1.25328			7.83243
FKH2	YGL175C		0.78692	5.83087						6.61779
FKH2	YGL181W	4.61658					1.51578			6.13236
FKH2	YGL184C	4.61658								4.61658
FKH2	YGL195W				1.10711	2.43286	1.51578		0.64966	5.70541
FKH2	YGL225W				0.530524	2.43737	1.77208		0.533984	5.27396
FKH2	YGL256W	3.29142	0.953018	2.19656						6.441
FKH2	YGL257C	3.29142	0.953018	2.07417					0.515194	6.8338
FKH2	YGR092W	3.74381	0.971367	9.202	2.67079				0.64445	17.2324
FKH2	YGR098C	1.55981	0.617026	3.54239			1.87315			7.59238
FKH2	YGR099W	1.55981	0.617026	5.96228						8.13912
FKH2	YGR108W	2.20181	0.415422	0.689278	1.91855		1.93476		0.662002	7.82182
FKH2	YGR152C			1.77772			2.012	1.39066	0.661664	5.84204
FKH2	YGR180C	0.583658	0.233182	1.39576	1.28767				0.723134	4.2234
FKH2	YGR184C	2.17105				1.3227			0.704508	4.19826
FKH2	YGR214W			0.782804	1.51665		1.01455		0.727092	4.0411
FKH2	YGR218W					4.11544				4.11544
FKH2	YGR229C	2.49334		2.96551						5.45885
FKH2	YGR230W	2.49334		4.86897						7.36231
FKH2	YHL010C			1.84696			1.22467	1.38815		4.45978
FKH2	YHL024W	4.84773								4.84773
FKH2	YHR023W	0.730718			2.5044	2.43286			0.73036	6.39834
FKH2	YHR026W					2.9592	1.67789		0.713312	5.3504
FKH2	YHR049W	0.509687		0.338607		2.73354	1.67047			5.2523
FKH2	YHR057C			0.0211015		3.1829	1.18226			4.38626
FKH2	YHR061C	4.67452		1.11225						5.78677
FKH2	YHR103W	1.67008			2.51739		1.55205			5.73952
FKH2	YHR128W				0.720799	3.92827			0.723885	5.37295
FKH2	YHR129C					2.43737	1.72812			4.16549
FKH2	YHR143W	4.53219	0.304465	3.13574	1.40328				0.710475	10.0862
FKH2	YHR148W				2.4292	0.699333	1.01115		0.691626	4.83131
FKH2	YHR151C	15.1617	0.00565177	1.96946						17.1368
FKH2	YHR152W	15.1617	0.00565177	1.92389	0.338391	0.553797				17.9834
FKH2	YHR158C	0.495505		0.515652		0.716601	1.28617	2.42017		5.4341
FKH2	YHR166C					3.13895	1.13383			4.27278
FKH2	YIL046W					1.72348	1.99341	1.193		4.90989
FKH2	YIL050W			1.41633		3.43598				4.85231
FKH2	YIL063C					2.43737	1.75828			4.19565
FKH2	YIL096C				0.717027	2.73354			0.668283	4.11885
FKH2	YIL106W			2.64326	2.56999	0.845942			0.718663	6.77786
FKH2	YIL107C			3.51835	1.70314					5.22149
FKH2	YIL123W	2.51618	0.218163		1.56736				0.679809	4.98151
FKH2	YIL124W			2.95257	1.53612					4.48869
FKH2	YIL133C				2.48296		1.29262		0.732469	4.50805
FKH2	YIL137C				2.07056	2.43737				4.50793
FKH2	YIL144W			0.605081		0.482195	0.971285	2.42017		4.47873
FKH2	YIL148W				1.48962		1.51755	0.986403	0.702745	4.69632
FKH2	YIL158W	15.8065	0.51619	0.1304	0.959124				0.592062	18.0043
FKH2	YIL169C	1.89454			2.13373					4.02827
FKH2	YIR012W				2.1587		2.00285		0.729823	4.89137
FKH2	YJL013C		0.419841	1.4962		4.10231				6.01835
FKH2	YJL014W					3.3208		0.883303	0.712104	4.91621
FKH2	YJL030W					4.10231				4.10231
FKH2	YJL036W	1.87477					1.07245	1.38509		4.33231
FKH2	YJL047C					2.43737	2.00462			4.44199
FKH2	YJL051W	12.8812	0.52115	2.01235	2.48125		0.820454			18.7164
FKH2	YJL052C-A	12.8812	0.52115							13.4024
FKH2	YJL069C				2.65261	1.22309	1.94197		0.686513	6.50418
FKH2	YJL078C	1.68836			0.660392		1.51947	1.00592	0.573929	5.44807
FKH2	YJL092W	1.54655			1.44056		0.946049		0.592617	4.52578
FKH2	YJL137C		0.308327	6.49254						6.80087
FKH2	YJL158C	3.46231		0.221464	1.48068				0.595078	5.75953
FKH2	YJL168C				1.52851	3.92827	1.51802			6.9748
FKH2	YJL186W	0.404149	0.455693	9.16212						10.022
FKH2	YJL187C	0.404149	0.455693	8.72648		2.35744	1.88129			13.8251
FKH2	YJL190C	0.48212			2.16127		1.03511		0.71737	4.39587
FKH2	YJR032W	1.51284	0.360852	1.12484			1.22544		0.693222	4.91719
FKH2	YJR050W					2.73354	2.01565			4.74919
FKH2	YJR063W				2.38821		1.03577		0.715953	4.13993
FKH2	YJR064W					3.3208			0.710842	4.03164
FKH2	YJR091C	14.0465	1.14889	8.05974		0.820005	1.51578			25.5909

TF	Target	Binding_0-2000	Motif_Score_SC	Other_Yeasts	Two-Hop_BE	Two-Hop_BP	Two-Hop_BY	Two-Hop_BG	Max_Membership	Sum
FKH2	YJR092W		1.14889	7.84319	2.4989		1.52631		0.722847	13.7401
FKH2	YJR094W-A		0.971367		1.52325		1.02418		0.710024	4.22882
FKH2	YJR119C					2.73354	1.72812	1.14194		5.6036
FKH2	YJR145C	1.0523		0.49213	1.31482		0.982323		0.703229	4.5448
FKH2	YJR148W	1.53645				3.92827				5.46472
FKH2	YKL002W					2.9592	1.75198			4.71118
FKH2	YKL067W	1.48215		1.54444	1.67387	1.05922				5.75968
FKH2	YKL081W			1.10781	1.23047	0.654959	2.02371		0.715152	5.7321
FKH2	YKL096W-A	3.12632	0.617026	1.74498						5.48833
FKH2	YKL099C	1.64077			2.27201				0.658845	4.57163
FKH2	YKL101W					4.10231				4.10231
FKH2	YKL113C				0.696957	2.9592	1.01275		0.731627	5.40053
FKH2	YKL121W				2.32946		2.06777			4.39723
FKH2	YKL144C				1.86026	2.73354	1.03457		0.691823	6.32019
FKH2	YKL146W					2.43737	1.73834			4.17571
FKH2	YKR036C			1.86091		0.845942	2.00063	2.74854		7.45602
FKH2	YKR042W	10.7767			1.50671				0.730131	13.0135
FKH2	YKR048C		0.134511			3.13895	1.93476			5.20822
FKH2	YKR051W	0.167882		2.59012			1.25172			4.00972
FKH2	YKR054C	1.16092	1.14889	3.2448		0.335831				5.89044
FKH2	YKR055W	1.16092	1.14889	17.8083			2.06777			22.1859
FKH2	YKR056W	1.16092	1.14889		1.02664				0.70733	4.04378
FKH2	YKR059W	2.47961			0.933575			0.863335	0.506947	4.78347
FKH2	YKR060W	2.47961			0.940526	0.699333			0.722319	4.84179
FKH2	YKR092C	2.17341			2.21402				0.730187	5.11762
FKH2	YKR099W				2.48507		1.91377		0.516804	4.91564
FKH2	YLL011W			0.281568	1.42198	0.699333	2.08491		0.637168	5.12496
FKH2	YLL039C					3.13895	1.51578	0.987515		5.64224
FKH2	YLR045C			0.503133			2.0339	1.96861	0.710854	5.2165
FKH2	YLR056W				2.36216		1.96573		0.715055	5.04294
FKH2	YLR084C	5.73054		5.6261	2.59957		1.65999		0.681804	16.298
FKH2	YLR086W	5.73054						2.42017		8.15071
FKH2	YLR131C	15.8551	0.415422	3.50497	2.31263		1.87831			23.9664
FKH2	YLR133W	15.8551	0.415422							16.2705
FKH2	YLR167W			1.64953	1.1555			0.990876	0.495617	4.29152
FKH2	YLR189C	14.7356	0.436223	0.646572						15.8184
FKH2	YLR190W	14.7356	0.436223	1.64775	2.54133				0.650114	20.011
FKH2	YLR196W		0.617026		1.24101		2.08554		0.687672	4.63125
FKH2	YLR210W	1.52592			2.57206		1.97586			6.07384
FKH2	YLR249W				2.83859	2.43286			0.72647	5.99792
FKH2	YLR254C	0.548639			2.39746		1.0394	1.11991		5.10541
FKH2	YLR257W	1.84083	0.316044	2.65987						4.81674
FKH2	YLR286C	5.90328			1.12319				0.71213	7.7386
FKH2	YLR300W	6.50915	0.325034						0.690512	7.5247
FKH2	YLR353W	1.81135	0.631025	3.88958						6.33196
FKH2	YLR392C	0.157864		0.824283		3.1829				4.16505
FKH2	YLR397C		0.527345	1.01021		2.73354			0.563168	4.83426
FKH2	YLR399C	4.14465					1.51578		0.72451	6.38494
FKH2	YLR409C				2.11688	0.699333	2.08491		0.714257	5.61538
FKH2	YLR429W						2.05397	2.78931	0.568056	5.41134
FKH2	YLR438C-A	3.5221				0.553797	1.04456			5.12046
FKH2	YML015C					2.9592	1.67857			4.63777
FKH2	YML034W	1.71885			2.68601				0.6732	5.07806
FKH2	YML051W	7.59432								7.59432
FKH2	YML052W	7.59432		1.57176	1.85774					11.0238
FKH2	YML053C	7.59432		1.55432						9.14864
FKH2	YML063W	5.93131	1.14889	11.1959	1.30143		1.0263		0.730694	21.3345
FKH2	YML064C	5.93131	1.14889	15.0463	0.709711	1.5276	2.03791			26.4017
FKH2	YML093W				1.59336		2.04213	2.41235	0.723703	6.77154
FKH2	YML126C	1.68743			2.3316		1.05603		0.727608	5.80267
FKH2	YMR001C	1.76964			2.8349		1.13795		0.692418	6.43491
FKH2	YMR031C	3.1444					1.04792	2.42017		6.61249
FKH2	YMR032W	3.1444			2.96152				0.706327	6.81225
FKH2	YMR049C				2.28824	0.81095	2.06994		0.725883	5.89501
FKH2	YMR076C	3.24982			1.09049				0.71218	5.05249
FKH2	YMR116C	2.39637	0.953018		1.23432	1.78138	1.99341		0.704494	9.06299
FKH2	YMR117C	2.39637	0.953018	2.12024	0.327503					5.79713
FKH2	YMR131C			0.527379	2.20576		2.08996		0.709364	5.53246
FKH2	YMR139W			0.568628		3.33393	1.3115			5.21406
FKH2	YMR144W	7.66637	0.0885384							7.75491
FKH2	YMR145C	1.07269			3.02902		1.27728		0.719494	6.09848
FKH2	YMR146C				0.754103		2.06301	2.82106	0.676004	6.31418
FKH2	YMR182C	0.29512			1.61211		1.87141	2.93651		6.71515
FKH2	YMR183C	2.71568					1.37599			4.09167
FKH2	YMR193W			3.88297			1.25054			5.13351
FKH2	YMR197C	6.95807	0.615197	5.61733		2.73354				15.9241
FKH2	YMR198W	6.95807	0.615197	5.51893			0.990503	2.392		16.4747
FKH2	YMR199W	5.10964		0.682066		2.73354			0.574583	9.09983
FKH2	YMR204C	0.902981	0.401512	4.25373						5.55822
FKH2	YMR215W	2.76714		0.125984	0.962752				0.607875	4.46375
FKH2	YMR290C	0.643457			2.11616	0.654959			0.730947	4.14552
FKH2	YMR299C	0.304567	1.14889	3.39192						4.84538
FKH2	YMR304W				0.397144	3.9249	1.87616	0.936899		7.1351
FKH2	YMR308C				0.539358	3.92827			0.615556	5.08318
FKH2	YNL030W	0.512863			2.22646	1.53233	0.856507		0.731422	5.85958
FKH2	YNL040W	3.91318							0.609911	4.52309
FKH2	YNL041C	3.91318					1.03199			4.94517
FKH2	YNL058C	2.28249		0.86683	2.4426				0.615162	6.20708
FKH2	YNL066W	0.813921	0.971367	7.84797	0.865495				0.593239	11.092
FKH2	YNL067W	0.9889	1.14889	3.34588	1.24983		1.03447		0.69605	8.46402
FKH2	YNL068C	0.9889	1.14889	8.75043						10.8882
FKH2	YNL110C				2.59441		2.00183		0.657625	5.25387
FKH2	YNL111C	0.178564			2.13409		1.51578		0.726464	4.5549
FKH2	YNL126W			4.23852					0.688546	4.92707
FKH2	YNL132W	0.90549			2.19239	0.699333			0.704872	4.50209
FKH2	YNL175C	2.58451			0.764586		2.0723		0.693507	6.1149
FKH2	YNL176C	2.52785				2.73354				5.26139
FKH2	YNL250W			0.736645			1.04947	2.42017		4.20629
FKH2	YNR001C	1.49817		3.58422						5.08239
FKH2	YOL030W	0.771737	0.51619	0.667164	1.74146				0.633032	4.32958
FKH2	YOL041C				2.28927		2.04341		0.631057	4.96374
FKH2	YOL077C				2.22735		2.02662		0.45045	4.70442
FKH2	YOL086C	1.52216			0.336948	2.43286	1.29469			5.58666
FKH2	YOR025W	1.16307			2.87425		0.90225		0.691494	5.63106
FKH2	YOR033C					2.73354	0.989858		0.691588	4.41499
FKH2	YOR058C	3.69403			1.69466			1.17035		6.55904
FKH2	YOR095C	1.6516			2.27938				0.681335	4.61231
FKH2	YOR101W		0.578502				2.03941	1.39056		4.00847
FKH2	YOR137C	0.426316		4.6592						5.08552
FKH2	YOR140W	1.72154			0.963381	0.501506	1.23356			4.41999

TF	Target	Binding_0-2000	Motif_Score_SC	Other_Yeasts	Two-Hop_BE	Two-Hop_BP	Two-Hop_BY	Two-Hop_BG	Max_Membership	Sum
FKH2	YOR188W	0.28719	0.325034	1.15051	2.63259	0.335831				4.73116
FKH2	YOR210W				2.50245		0.889418		0.671014	4.06288
FKH2	YOR230W	2.84942		0.142388			1.12054			4.11235
FKH2	YOR239W	2.06196	0.252062				2.10721		0.681531	5.10276
FKH2	YOR246C	8.93667		1.06575						10.0024
FKH2	YOR247W	8.93667		2.00168	1.88259				0.713318	13.5343
FKH2	YOR265W					3.24994	0.944705			4.19465
FKH2	YOR272W				2.20206	0.654959	2.05811	1.32273	0.695484	6.93334
FKH2	YOR313C	14.2404	0.134511		2.52073				0.510975	17.4066
FKH2	YOR315W	14.2404	0.134511	6.97454	2.52073				0.730125	24.6003
FKH2	YOR317W	1.42738				3.24994			0.481899	5.15922
FKH2	YOR369C	1.62499	0.1292		2.20699		1.03973		0.701818	5.70273
FKH2	YPL016W	1.59963	0.473223	1.39998	1.20822					4.68105
FKH2	YPL017C	1.59963	0.473223	2.10938						4.18223
FKH2	YPL116W	5.45674	0.953018	5.05067	0.413339					11.8738
FKH2	YPL117C	5.45674	0.953018	6.8517	2.23104				0.496069	15.9886
FKH2	YPL140C	1.74679		0.301529		3.3208				5.36912
FKH2	YPL141C	2.3842	1.14889	8.59181	2.86897				0.706048	15.6999
FKH2	YPL155C	5.85124								5.85124
FKH2	YPL163C	0.640637		2.30576	1.03284				0.580565	4.5598
FKH2	YPL177C	5.50988				0.421369	2.00462			7.93587
FKH2	YPL181W			2.04767			2.07977			4.12744
FKH2	YPL183C			0.159927	1.37249		2.00193	3.14183	0.690133	7.36631
FKH2	YPL184C				2.47796		2.00462			4.48258
FKH2	YPL192C	0.628658		3.47658		0.992169				5.09741
FKH2	YPL209C		0.615197	3.70806		0.526363				4.84962
FKH2	YPL219W				1.8642	2.73354				4.59774
FKH2	YPL226W	0.515542	0.308327	2.02655	0.499057				0.706095	4.05557
FKH2	YPL242C	3.1498		1.00384	2.54272	1.43647	1.89865		0.61232	10.6438
FKH2	YPL260W			0.861293			1.32164	1.98536		4.16829
FKH2	YPL276W				2.28307		2.00726			4.29033
FKH2	YPR028W						1.94716	2.97038		4.91754
FKH2	YPR033C	1.41414	0.615197	3.47087	0.98033				0.711452	7.19199
FKH2	YPR034W	1.41414	0.615197	4.74498			1.93476			8.70908
FKH2	YPR035W	5.73179	0.419841	1.26672					0.682552	8.1009
FKH2	YPR086W					4.11544	1.01908			5.13452
FKH2	YPR110C			1.38571	2.15006	0.600761	0.962786		0.727881	5.8272
FKH2	YPR112C				0.774744		1.22959	2.04863	0.659602	4.71257
FKH2	YPR119W	12.2379		0.172298	2.81173	4.10231	1.93608		0.725537	21.9859
FKH2	YPR120C				2.36135	2.73354	1.72812			6.82301
FKH2	YPR141C			1.55931	0.538544	1.71364	1.95344	0.896875		6.66181
FKH2	YPR149W	3.62275			0.873848					4.4966
FKH2	YPR152C	0.654117				3.92827				4.58239
FKH2	YPR188C				0.695939	2.43737	1.73222			4.86553
FZF1	YIL004C	4.58424								4.58424
FZF1	YJL148W	4.20938			0.476319					4.6857
FZF1	YOR138C	5.00878								5.00878
FZF1	YOR140W	5.00878								5.00878
FZF1	YPL034W	4.76077								4.76077
GAL4	TM(CAU)J3	4.74185								4.74185
GAL4	YAL049C	3.09358	2.83612							5.9297
GAL4	YAL053W	0.225625		6.34671						6.57233
GAL4	YBL041W		0.323751	2.85207			0.919701			4.09552
GAL4	YBR009C	0.750246			0.843754	1.70399	0.829852			4.12784
GAL4	YBR015C	4.66829								4.66829
GAL4	YBR017C	8.77663								8.77663
GAL4	YBR018C	12.4406	2.62115	10.3105	2.61592		1.62529		0.710273	30.3237
GAL4	YBR019C	12.596	3.97648	12.7179	2.47403				0.713914	32.4783
GAL4	YBR020W	12.596	3.97648	12.3456	2.58416	2.75622	1.60703		0.707087	36.5726
GAL4	YBR021W	9.68779			2.16951				0.647715	12.505
GAL4	YBR022W	4.1882								4.1882
GAL4	YBR033W	1.45612		6.10065						7.55677
GAL4	YCL067C	3.43009					0.947699			4.37779
GAL4	YCR107W	3.86921					0.967692			4.8369
GAL4	YDL079C	0.843504	1.31079	3.7839						5.93819
GAL4	YDR006C	5.14433	2.5782							7.72253
GAL4	YDR007W	5.14433	0.0448876	0.0538667						5.24308
GAL4	YDR009W	12.2901	2.5782	5.31357	1.56045	2.75622	1.50074			25.9993
GAL4	YDR058C		1.48704	3.32426						4.8113
GAL4	YDR277C	8.43316	1.00732	3.168						12.6085
GAL4	YDR299W	3.9782			0.897623					4.87582
GAL4	YDR377W			4.77771						4.77771
GAL4	YDR487C			4.9166						4.9166
GAL4	YEL056W	6.92235	3.68226							10.6046
GAL4	YEL057C	6.92235	3.68226						0.684424	11.289
GAL4	YER188C-A	5.82806								5.82806
GAL4	YER189W	5.82806			0.947223			1.60929		8.38457
GAL4	YER190W	5.82806			0.947223			1.60929		8.38457
GAL4	YFR017C		0.223392	4.43158	0.527134					5.18211
GAL4	YFR033C		1.16994	3.02355						4.19349
GAL4	YGL064C		2.21267	8.07176						10.2844
GAL4	YGL127C			4.04581						4.04581
GAL4	YGL134W	5.16066	0.362127	2.29908						7.82187
GAL4	YGL136C	5.16066	0.362127	0.69122						6.21401
GAL4	YGL200C			3.48271		0.470547	0.947699			4.90096
GAL4	YGL221C			6.91292						6.91292
GAL4	YGR067C			2.0256	2.13853					4.16413
GAL4	YGR095C			6.12142						6.12142
GAL4	YGR253C	0.393783		5.53671			0.840318			6.77081
GAL4	YGR254W	0.393783		5.16607						5.55985
GAL4	YHR091C	4.14376								4.14376
GAL4	YJL039C		1.48704	3.00439						4.49143
GAL4	YJR044C	4.74185								4.74185
GAL4	YKL127W			7.61766						7.61766
GAL4	YKR038C		1.48704	4.17585						5.66289
GAL4	YLL066C	3.63952			1.03796			1.60929		6.28677
GAL4	YLR081W	12.0104	3.12564	3.74688	1.99863				0.705611	21.5872
GAL4	YLR082C	4.30382								4.30382
GAL4	YLR152C			4.26669						4.26669
GAL4	YLR351C	0.797459	1.32049	2.86376						4.98171
GAL4	YLR357W	3.04509	2.86242							5.90751
GAL4	YLR437C			4.48955						4.48955
GAL4	YLR438W		0.147104	6.75451						6.90161
GAL4	YML050W	10.8201	3.94845							14.7685
GAL4	YML051W	10.8201	3.94845	11.803		3.23563			0.440425	30.2476
GAL4	YML053C	10.8201	3.94845							14.7685
GAL4	YML102W		1.0836	4.32743			1.04435			6.45538
GAL4	YMR105C		1.10265	1.82181	0.677288	1.42307				5.02482
GAL4	YMR315W	0.105489		2.03898	0.617594		1.42324			4.1853
GAL4	YMR318C		1.69084	1.49892			0.882757		0.604155	4.67667

TF	Target	Binding_0-2000	Motif_Score_SC	Other_Yeasts	Two-Hop_BE	Two-Hop_BP	Two-Hop_BY	Two-Hop_BG	Max_Membership	Sum
GAL4	YNL055C		1.48704	4.24275	0.692328					6.42212
GAL4	YNL073W	4.99837	2.56136	5.69114						13.2509
GAL4	YNL074C	4.99837	2.56136	5.6648						13.2245
GAL4	YNL339C	3.87823			0.382212					4.26044
GAL4	YOL098C		1.53648	3.22468						4.76116
GAL4	YOR119C	6.02486	3.16232	6.94735	0.479544					16.6141
GAL4	YOR120W	6.02486	3.16232	6.94704					0.515076	16.6493
GAL4	YOR138C	5.76438	2.09417	2.00234						9.86089
GAL4	YOR140W	5.76438	2.09417	1.81627						9.67482
GAL4	YPL016W		2.51882	1.54883						4.06765
GAL4	YPL066W	1.80687	1.74704						0.506824	4.06073
GAL4	YPL088W			2.78673	0.331347		0.968668			4.08674
GAL4	YPR194C	2.56713	1.88514						0.659893	5.11216
GAL80	YBR019C	2.80038	1.75253	1.90334	0.414061				0.713914	7.58422
GAL80	YBR020W	2.80038	1.75253	2.21755	0.414061				0.700524	7.88504
GAL80	YBR210W	0.217587	1.48495	2.44003						4.14257
GAL80	YCL057C-A		1.48495	5.5559						7.04085
GAL80	YCL057W		1.48495	4.93982						6.42477
GAL80	YCR049C		1.75253	2.67249						4.42502
GAL80	YCR051W		1.75253	3.50765						5.26018
GAL80	YCR057C		1.48495	3.55481						5.03976
GAL80	YCR060W		1.48495	4.40836						5.89331
GAL80	YDL173W			8.10384						8.10384
GAL80	YDR487C			6.02264						6.02264
GAL80	YER012W		1.48495	4.72657						6.21152
GAL80	YER035W		1.20356	4.52422						5.72778
GAL80	YER088C		0.259207	4.74742						5.00663
GAL80	YER098W	0.252507	1.48495	4.39686						6.13432
GAL80	YFR033C		0.265694	6.33749						6.60318
GAL80	YGL054C		1.48495	3.02261						4.50756
GAL80	YGR028W		1.48495	4.33768						5.82263
GAL80	YGR086C		1.48495	4.39543						5.88038
GAL80	YIR007W		1.48495	2.54086						4.02581
GAL80	YJR147W		1.48495	3.35651						4.84146
GAL80	YKR003W			4.79893						4.79893
GAL80	YLL012W		2.00852	2.24097						4.24949
GAL80	YLR437C		0.596268	6.31099						6.90726
GAL80	YLR438W			8.59263						8.59263
GAL80	YML051W		1.75253	5.25077		0.591261				7.59456
GAL80	YMR086W		2.00852	4.49126						6.49978
GAL80	YNL241C		1.48495	5.0262						6.51115
GAL80	YNL327W		1.75253	4.83727						6.5898
GAL80	YNL328C		1.75253	5.82423						7.57676
GAL80	YPL088W			4.08608						4.08608
GAL80	YPL230W		1.75253	2.9834						4.73593
GAL80	YPL248C		1.48495	3.69168						5.17663
GAT1	SNR53	4.3781								4.3781
GAT1	TG(GCC)G2	4.82433								4.82433
GAT1	TK(CUU)C	5.14778								5.14778
GAT1	TR(UCU)E	5.71552								5.71552
GAT1	TT(AGU)C	4.66894								4.66894
GAT1	TW(CCA)J	7.5429								7.5429
GAT1	TW(CCA)K	5.06425								5.06425
GAT1	YCL066W			2.93034		1.09569				4.02603
GAT1	YCR019W	5.14778		1.22909						6.37687
GAT1	YCR096C	4.66894		0.616015		1.09569				6.38065
GAT1	YCR097W	4.66894		0.842031		1.09569				6.60666
GAT1	YDR040C	4.21241								4.21241
GAT1	YDR041W	4.21241								4.21241
GAT1	YDR042C	3.24614		1.32063						4.56677
GAT1	YDR079W	2.57135		2.63332						5.20467
GAT1	YDR084C	6.20462								6.20462
GAT1	YDR139C			4.97956						4.97956
GAT1	YEL024W			3.69168	0.56148					4.25316
GAT1	YEL050C	4.3781								4.3781
GAT1	YEL058W	0.795863		4.34118						5.13704
GAT1	YEL062W	2.03527		2.1755						4.21077
GAT1	YGL001C	3.80843		1.89916	0.572974					6.28056
GAT1	YGR023W			4.00888						4.00888
GAT1	YGR071C	5.5294		0.1064						5.6358
GAT1	YGR072W	5.5294		1.23752						6.76692
GAT1	YGR074W	5.5294								5.5294
GAT1	YGR218W	4.82433								4.82433
GAT1	YHR015W	3.85985		2.1907						6.05055
GAT1	YHR156C	2.01561		2.43715						4.45276
GAT1	YIR005W	2.68906		2.09441						4.78347
GAT1	YIR029W	3.80458		3.30134						7.10592
GAT1	YJL110C	3.43654		0.905903						4.34244
GAT1	YJL128C			4.82584						4.82584
GAT1	YML098W	2.14273		2.2302		0.646506				5.01944
GAT1	YML099C	2.14273		3.23001						5.37274
GAT1	YMR019W	5.14049		1.03393						6.17442
GAT1	YMR119W	3.81436			0.484233					4.29859
GAT1	YNL142W	2.91414		1.14277						4.05691
GAT1	YOL159C	4.26552								4.26552
GAT1	YPL216W	2.81062		1.72183						4.53245
GAT1	YPL274W			4.66341						4.66341
GAT3	YEL075C	2.8655			0.411708			1.1423		4.41951
GAT3	YEL076C	2.8655			0.621208			1.1423		4.62901
GAT3	YEL076C-A	2.8655			0.621208			1.1423		4.62901
GAT3	YEL077C	2.8655			0.361988			1.1423		4.36979
GAT3	YNL339C	3.79107			0.53688					4.32795
GAT3	YPR202W	3.23647			0.650169			1.1423		5.02894
GAT3	YPR203W	3.23647			0.617933			1.1423		4.9967
GCN4	TD(GUC)O	4.67805								4.67805
GCN4	TT(AGU)J	4.23105								4.23105
GCN4	YBL039C				1.01904	3.43106				4.4501
GCN4	YBL043W	3.38286		0.353504	1.77521					5.51157
GCN4	YBL045C	3.38286			0.621054					4.00391
GCN4	YBL076C	3.54303			0.850064					4.39309
GCN4	YBR043C	4.22369		0.170128						4.39382
GCN4	YBR048W	2.88643			0.949031		0.856018		0.649936	5.34141
GCN4	YBR114W	3.75227						2.24973		6.002
GCN4	YBR115C	4.50693			1.61197		1.35628			7.47518
GCN4	YBR139W			3.36203					0.662157	4.02419
GCN4	YBR143C	3.75581			0.91546		0.923728			5.595
GCN4	YBR145W	3.75581		3.73895	1.92343			0.897174		10.3154
GCN4	YBR146W	3.75581					0.963712			4.71952
GCN4	YBR147W	2.6294		5.31436	0.649403				0.457661	9.05082
GCN4	YBR245C					2.17785		2.24962		4.42747

TF	Target	Binding_0-2000	Motif_Score_SC	Other_Yeasts	Two-Hop_BE	Two-Hop_BP	Two-Hop_BY	Two-Hop_BG	Max_Membership	Sum
GCN4	YBR248C	3.61354		0.435042	2.31846		1.76483			8.13187
GCN4	YBR249C	3.42379		0.987247						4.41104
GCN4	YBR250W	3.42379		2.03898						5.46277
GCN4	YCL028W	7.88316								7.88316
GCN4	YCL030C	7.88316			1.98482		1.55241		0.6744	12.0948
GCN4	YDL004W			3.23001	0.962579					4.19259
GCN4	YDL025C	1.71508		0.0953101	1.70287				0.709457	4.22272
GCN4	YDL069C	2.79611		1.45048						4.24659
GCN4	YDL110C				2.317	1.91357				4.23057
GCN4	YDL170W	6.39458		1.28812						7.6827
GCN4	YDL171C	6.39458							0.688957	7.08354
GCN4	YDL182W	3.94231			1.27409		1.29284			6.50924
GCN4	YDL198C	7.14525		0.546146					0.580378	8.27177
GCN4	YDL199C	7.14525								7.14525
GCN4	YDR035W	3.29705			2.45892				0.725478	6.48145
GCN4	YDR084C	3.82308				0.339658				4.16274
GCN4	YDR125C	13.6161								13.6161
GCN4	YDR127W	13.6161								13.6161
GCN4	YDR237W	4.09157			1.13585		0.969162			6.19658
GCN4	YDR341C	3.68303			0.360822					4.04385
GCN4	YDR354W	3.37531		0.878809			1.54904	1.10264		6.9058
GCN4	YDR425W			0.395943	0.690241	2.95362				4.0398
GCN4	YDR481C	5.3046							0.599449	5.90405
GCN4	YDR483W	5.3046		0.0729421						5.37754
GCN4	YDR511W	2.61321		1.54098						4.15419
GCN4	YEL062W	9.16173								9.16173
GCN4	YEL063C	9.16173					1.40098			10.5627
GCN4	YER039C	2.46309			1.12443		1.24433	0.958956		5.79081
GCN4	YER052C	10.5585		0.115605	2.42821				0.706791	13.8091
GCN4	YER055C	9.60633		0.189689	1.02452		1.52784		0.549553	12.8979
GCN4	YER061C	1.87614					0.789386	1.23588	0.632615	4.53402
GCN4	YER069W	8.96585		0.0811535	2.03476	0.792356	1.66192		0.515261	14.0513
GCN4	YER073W	3.79077		0.0538503	1.87889	0.337703				6.06121
GCN4	YER086W	3.31375		0.139367	1.02469		1.52461			6.00242
GCN4	YER090W	3.11503			0.896643	0.649571	1.66757	1.43817		7.76698
GCN4	YER095W					3.43106	1.24473			4.67579
GCN4	YER175C	2.72864			1.59259				0.688352	5.00958
GCN4	YFL028C	2.80209			1.47953					4.28162
GCN4	YFR024C-A	1.60742				2.72951				4.33693
GCN4	YFR049W	1.36826		2.81298						4.18124
GCN4	YGL009C	1.88561		0.803399	2.06679		1.6652			6.421
GCN4	YGL026C	1.81358		0.933478	1.94732		1.70418			6.39856
GCN4	YGL059W	2.96696		0.0658525	1.98326					5.01607
GCN4	YGL117W	2.74353		0.315439	2.27114				0.659039	5.98915
GCN4	YGL125W	3.26582		0.285286					0.708114	4.25922
GCN4	YGL181W	5.17146								5.17146
GCN4	YGL184C	5.17146		0.588631						5.76009
GCN4	YGR019W	1.00488			1.22064		1.55466		0.731616	4.5118
GCN4	YGR061C	0.90913		0.890403	1.07194		1.46969			4.34116
GCN4	YGR161C	2.72553		0.697437	1.19784					4.62081
GCN4	YGR202C	2.61293		1.77003						4.38296
GCN4	YGR204W	2.61293		1.86634						4.47927
GCN4	YGR234W			0.238085	0.865488	3.32048				4.42405
GCN4	YGR267C	3.81324		1.01008	1.7276					6.55092
GCN4	YHR018C	11.3548		1.0612	2.75201		1.90162			17.0696
GCN4	YHR019C	2.80397		0.440179	0.388229				0.470839	4.10322
GCN4	YHR020W	2.80397		0.436033	1.22537				0.517937	4.98331
GCN4	YHR071W	7.41023			2.27671					9.68694
GCN4	YHR161C	3.9574		1.29754						5.25494
GCN4	YHR162W	3.9574		3.69168						7.64908
GCN4	YHR163W	3.9574		3.2448						7.2022
GCN4	YHR194W			2.35106	2.10137					4.45243
GCN4	YHR208W	1.55962		2.42608	1.17151				0.454622	5.61183
GCN4	YIL018W	1.79366		2.10873	0.907426				0.564629	5.37444
GCN4	YIL056W	2.06033		3.42821						5.48854
GCN4	YIL057C	2.06033		3.53056	1.36704	0.337703				7.29563
GCN4	YIL064W			3.34588	0.696489					4.04237
GCN4	YIL065C			5.25851						5.25851
GCN4	YIL078W	1.57023		0.911671	0.613114			1.06436		4.15937
GCN4	YIL079C	1.57023		0.932508		0.688773	1.24433			4.43584
GCN4	YIL094C	0.726508		2.18918	0.388899		1.48062			4.78521
GCN4	YIL116W	1.98013		1.90324	1.89855		1.80804			7.58996
GCN4	YIL125W			1.10815	2.18725		1.27248			4.56788
GCN4	YIR034C	3.05428		0.0895763				1.1246	0.46156	4.73002
GCN4	YJL008C	0.705036				3.32048	0.886534			4.91205
GCN4	YJL071W	3.54531			0.907419	2.25565				6.70838
GCN4	YJL088W	10.468		0.142605	1.3785		1.1764	2.68396	0.689725	16.5392
GCN4	YJL089W	0.89665			1.64261			2.24293		4.78219
GCN4	YJL130C	2.31041		0.00590778	1.9521		1.39529	1.05873		6.72244
GCN4	YJL173C	1.46719		0.770323		2.72951				4.96702
GCN4	YJL198W	4.34326								4.34326
GCN4	YJL200C	4.34326		1.96024			1.58945			7.89295
GCN4	YJL212C	2.13762		2.62336						4.76098
GCN4	YJR016C	3.59306		0.442527	1.49463		1.36053			6.89075
GCN4	YJR024C	3.7738		0.473132						4.24693
GCN4	YJR025C	3.7738		0.4819	0.485731				0.515317	5.25675
GCN4	YJR109C	14.0258		0.411702	2.20641	1.16716	1.58803	2.04287	0.658616	22.1006
GCN4	YJR110W	14.0258		0.591969						14.6178
GCN4	YJR112W	2.89415			2.11157					5.00572
GCN4	YKL015W	9.34451		0.202787				1.11102		10.6583
GCN4	YKL016C	9.34451		2.1917	0.557418					12.0936
GCN4	YKL017C	9.34451								9.34451
GCN4	YKL103C	2.72729			0.699504				0.714251	4.14105
GCN4	YKL184W						1.8266	2.4617		4.2883
GCN4	YKL210W	3.01638					1.47694			4.49332
GCN4	YKL211C	3.01638			2.29253	0.676202	1.80267	1.16732		8.9551
GCN4	YKL216W				1.1574		1.80233	1.71138		4.67111
GCN4	YLL004W	4.2353								4.2353
GCN4	YLL005C	4.2353					1.24433			5.47963
GCN4	YLR047C	2.96533		1.11525						4.08058
GCN4	YLR048W	2.96533		0.106105	1.17193		0.911752			5.15512
GCN4	YLR152C	3.4957							0.567334	4.06303
GCN4	YLR293C			1.49859	0.418124	1.90408			0.44516	4.26595
GCN4	YLR355C	9.38347		1.02101			1.45332		0.721263	12.5791
GCN4	YLR356W	9.38347		0.891301						10.2748
GCN4	YLR357W	9.38347						1.23502		10.6185
GCN4	YML116W	2.4807			1.37972		1.5385		0.587553	5.98647
GCN4	YMR019W	3.06286			0.401511			2.24295	0.440044	6.14737
GCN4	YMR062C	9.57521			2.71878		1.07513		0.551357	13.9205
GCN4	YMR063W	9.57521								9.57521

TF	Target	Binding_0-2000	Motif_Score_SC	Other_Yeasts	Two-Hop_BE	Two-Hop_BP	Two-Hop_BY	Two-Hop_BG	Max_Membership	Sum
GCN4	YMR064W	9.57521								9.57521
GCN4	YMR095C	3.58954		0.611082		0.427048				4.62767
GCN4	YMR096W	3.58954		0.571122		0.427048				4.58771
GCN4	YMR108W	1.64681			0.957427		1.57398		0.615063	4.79328
GCN4	YMR135C	3.17792		0.891596						4.06952
GCN4	YMR186W				0.909544	3.32048				4.23002
GCN4	YMR188C	3.39958		1.62624			0.913356			5.93918
GCN4	YMR189W	3.39958		0.629788					0.451518	4.48089
GCN4	YMR195W	2.71637		1.32733					0.633771	4.67747
GCN4	YMR217W			2.14714	1.49353			1.13106		4.77173
GCN4	YMR251W-A	1.59902			2.2073				0.672912	4.47923
GCN4	YNL004W	6.05657								6.05657
GCN4	YNL005C	6.05657			0.734458					6.79103
GCN4	YNL065W	2.9632					1.46596			4.42916
GCN4	YNL104C	3.83758		0.563819			1.2777			5.6791
GCN4	YNL175C	1.93245		0.824239	0.927297		0.952246			4.63623
GCN4	YNR050C	3.13822						1.10947		4.24769
GCN4	YOL040C			3.19543	0.875975		0.951465			5.02287
GCN4	YOL058W	9.93213		0.250098	2.10735		1.76825			14.0578
GCN4	YOL064C	5.01798		0.229239	1.55958					6.8068
GCN4	YOL065C	5.01798		0.20912						5.2271
GCN4	YOL119C	3.5417		0.509429						4.05113
GCN4	YOL140W	10.5199		0.293348	1.1778		1.35619			13.3472
GCN4	YOR108W	8.80022			0.981622		1.2777			11.0595
GCN4	YOR130C	4.67805			1.69207		0.91787		0.584787	7.87278
GCN4	YOR136W					3.32048	1.48062			4.8011
GCN4	YOR201C	2.81486		2.71761						5.53247
GCN4	YOR202W	2.81486		2.3501	1.31677		1.52405		0.555165	8.56095
GCN4	YOR221C	2.71501		0.530527	1.57324					4.81878
GCN4	YOR222W	2.71501		0.503866			1.18065			4.39953
GCN4	YOR302W	4.9337			0.521994	3.6216				9.07729
GCN4	YOR303W	4.9337		0.0277546	1.91735	3.6216	1.90656	2.71753		15.1245
GCN4	YOR337W	9.05993		0.0389574						9.09889
GCN4	YPL111W	3.50937		0.364464	0.910619					4.78445
GCN4	YPL227C			2.88287			1.29368			4.17655
GCN4	YPL235W			1.5138		3.32048				4.83428
GCN4	YPL252C	3.46901		2.27243						5.74144
GCN4	YPL253C	3.46901						0.922166		4.39118
GCN4	YPL264C	3.16359					1.13685			4.30044
GCN4	YPL273W	3.59514			1.32347					4.91861
GCN4	YPR058W	2.76478		1.41823						4.18301
GCN4	YPR110C	3.30555			1.00744					4.31299
GCN4	YPR145W	2.89606		0.265538	1.60947					4.77107
GCR1	TF(GAA)D	2.26303	2.09792							4.36095
GCR1	YAL038W	4.07792	2.09792	7.70166						13.8775
GCR1	YBL066C			5.98447						5.98447
GCR1	YBL074C	1.48222	0.0381421	2.99897						4.51933
GCR1	YBL091C-A		0.524249	3.69168						4.21593
GCR1	YBR196C	2.69751	0.0381421	2.8624						5.59805
GCR1	YCL044C		2.09792	2.27493						4.37285
GCR1	YDR050C	0.952396	0.0381421	4.15751						5.14805
GCR1	YDR097C	1.43886	0.166273	5.09017						6.6953
GCR1	YDR107C			6.64532						6.64532
GCR1	YDR155C		2.09792	2.07458	0.745354					4.91785
GCR1	YDR156W	0.159113	2.09792	2.29333						4.55036
GCR1	YDR180W		2.09792	3.48408						5.582
GCR1	YDR257C		0.0381421	4.61434						4.65248
GCR1	YDR328C			4.23648						4.23648
GCR1	YDR399W		2.09792	5.45158						7.5495
GCR1	YDR487C		0.913827	4.11365						5.02748
GCR1	YER098W	1.98653		4.28317						6.2697
GCR1	YER177W	2.28604	2.09792	7.27117						11.6551
GCR1	YER178W	2.28604	2.09792							4.38396
GCR1	YGR014W		2.09792	2.9834						5.08132
GCR1	YGR071C		0.166273	4.97112						5.13739
GCR1	YGR080W		0.0381421	7.71629						7.75443
GCR1	YGR286C			4.77442						4.77442
GCR1	YHR137W	0.703955	0.404044	3.2448						4.3528
GCR1	YIR011C		0.0381421	4.14398						4.18212
GCR1	YJR009C	1.02902	2.09792	1.5013	0.922235					5.55047
GCR1	YJR072C		0.913827	4.15002						5.06385
GCR1	YJR074W		0.913827	4.11378						5.02761
GCR1	YKL060C	1.20092	0.524249	5.0447	0.44933					7.2192
GCR1	YKL073W			5.52815						5.52815
GCR1	YKL141W		2.09792	2.18615						4.28407
GCR1	YKL152C	1.69732	0.0381421	3.21799						4.95345
GCR1	YKR007W		2.09792	1.91447						4.01239
GCR1	YLR150W		2.09792	2.19401						4.29193
GCR1	YLR374C			4.0617						4.0617
GCR1	YML004C			5.61545						5.61545
GCR1	YML102W		0.404044	3.62945						4.03349
GCR1	YMR060C		2.09792	4.64403						6.74195
GCR1	YMR184W		2.09792	1.93467						4.03259
GCR1	YMR205C	0.814908	2.09792	1.27443						4.18726
GCR1	YMR206W	0.814908	2.09792	1.39956						4.31239
GCR1	YMR233W		2.09792	4.87197						6.96989
GCR1	YMR303C			4.15354	0.378073					4.53161
GCR1	YNR039C		0.0381421	5.34938						5.38752
GCR1	YOL028C		0.913827	6.42948						7.34331
GCR1	YOL029C		0.913827	4.29045						5.20428
GCR1	YOR197W		0.0381421	5.19062						5.22876
GCR1	YOR278W		0.913827	4.10165						5.01548
GCR1	YPL013C		2.09792	4.46218						6.5601
GCR1	YPL022W		0.913827	3.15944						4.07327
GCR1	YPL047W		2.09792	4.41156						6.50948
GCR1	YPL075W		2.09792	9.24471						11.3426
GCR1	YPR061C			4.54003						4.54003
GCR1	YPR068C		2.09792	2.60959						4.70751
GCR2	TP(UGG)O3	6.20379								6.20379
GCR2	YAL038W	8.74149			1.21074					9.95223
GCR2	YBR143C	5.95929			0.343213		0.893568			7.19607
GCR2	YBR145W	5.95929								5.95929
GCR2	YBR146W	5.95929					0.893568			6.85286
GCR2	YBR196C	8.17594								8.17594
GCR2	YBR221C				1.65841	1.82799	1.19945	2.01643		6.70228
GCR2	YDL027C	4.36415								4.36415
GCR2	YDL028C	4.36415								4.36415
GCR2	YDR042C	4.80414								4.80414
GCR2	YDR050C	1.26341			1.66016		1.15319			4.07676
GCR2	YER091C	3.57656							0.465259	4.04182

TF	Target	Binding_0-2000	Motif_Score_SC	Other_Yeasts	Two-Hop_BE	Two-Hop_BP	Two-Hop_BY	Two-Hop_BG	Max_Membership	Sum
GCR2	YER177W	8.0875			1.24865					9.33615
GCR2	YER178W	8.0875			1.1712					9.2587
GCR2	YGL100W	4.81256				0.440128				5.25269
GCR2	YGL101W	4.81256								4.81256
GCR2	YGL104C	4.81256								4.81256
GCR2	YGL136C	3.35804			1.11762					4.47566
GCR2	YGL255W	4.60173								4.60173
GCR2	YGR192C	8.31148			1.71894					10.0304
GCR2	YGR240C	2.80371			0.901899	1.52769				5.2333
GCR2	YHL034C	3.11228			1.39893					4.51121
GCR2	YHR174W	3.50254			1.78753					5.29007
GCR2	YIL004C	4.67177								4.67177
GCR2	YJL148W	3.93463			0.856177					4.79081
GCR2	YJL194W	5.25022					0.939189			6.18941
GCR2	YJL196C	5.25022								5.25022
GCR2	YJR009C	6.98923			1.98905					8.97828
GCR2	YJR010W	6.98923								6.98923
GCR2	YKL060C	8.96158			1.49773					10.4593
GCR2	YKL152C	8.2215			1.22128		0.83073			10.2735
GCR2	YLL015W	7.67434								7.67434
GCR2	YLL025W	7.82311								7.82311
GCR2	YLR264W	2.03082			1.1933		0.834976			4.0591
GCR2	YLR301W	3.24942			1.12062					4.37004
GCR2	YMR014W	4.61638			0.716912					5.33329
GCR2	YMR205C	6.97503				0.401626	0.977075			8.35373
GCR2	YMR206W	6.97503								6.97503
GCR2	YOL084W	6.4149								6.4149
GCR2	YOL086C	6.4149			2.01796					8.43286
GCR2	YOL121C	3.35364			0.556892		0.915336			4.82587
GCR2	YOR065W	4.44341								4.44341
GCR2	YOR303W	3.94101					0.890987			4.832
GLN3	TK(CUU)C	4.04464								4.04464
GLN3	TR(UCU)E	5.15526								5.15526
GLN3	YAL062W	1.07888	0.180771	6.31482						7.57447
GLN3	YBL028C	0.772112		1.09359	2.53134					4.39704
GLN3	YBL090W			4.45248						4.45248
GLN3	YBL095W			4.61371						4.61371
GLN3	YBR043C	1.75911	0.180771	8.3363						10.2762
GLN3	YBR208C	0.546182	0.180771	7.1973						7.92425
GLN3	YBR247C				3.23654	3.9249				7.16144
GLN3	YBR248C				3.22105		1.50793			4.72898
GLN3	YBR267W				2.2467		1.916			4.1627
GLN3	YBR271W				2.80587		1.87141			4.67728
GLN3	YCL037C				2.26843		1.96532			4.23375
GLN3	YCL059C				0.808041	3.9249				4.73294
GLN3	YCL063W	2.63628	0.180771	6.95833						9.77538
GLN3	YCL064C	2.63628	0.180771	8.45331						11.2704
GLN3	YCR019W	4.04464								4.04464
GLN3	YCR034W				3.25997		1.87141			5.13138
GLN3	YCR057C				2.29203	3.9249				6.21693
GLN3	YCR082W			4.73945						4.73945
GLN3	YDL098C		0.180771	6.72334						6.90411
GLN3	YDL170W	9.39736		0.917222						10.3146
GLN3	YDL171C	9.39736		0.23525						9.63261
GLN3	YDL215C	2.97355					1.36786			4.34141
GLN3	YDR030C	4.27654								4.27654
GLN3	YDR031W	4.27654		0.575509						4.85205
GLN3	YDR041W	3.0647		1.39282						4.45752
GLN3	YDR077W	3.72404			0.462468					4.18651
GLN3	YDR379C-A			5.95277						5.95277
GLN3	YDR380W			5.32356						5.32356
GLN3	YDR447C			3.29632	2.72534					6.02166
GLN3	YDR448W	0.0502479	0.180771	3.78937		1.17062	1.89659			7.0876
GLN3	YDR496C	0.254099			2.87298		1.87164			4.99872
GLN3	YDR510W	3.36118		1.19795						4.55913
GLN3	YDR511W	3.36118		1.18272						4.5439
GLN3	YEL009C	4.94653								4.94653
GLN3	YEL013W	0.427703		3.08649		0.374358	0.821713			4.71026
GLN3	YEL058W	0.969791		7.51315						8.48294
GLN3	YEL059C-A			8.77215						8.77215
GLN3	YEL060C	0.969791		5.8843						6.85409
GLN3	YEL062W	1.78782		3.93757						5.72539
GLN3	YEL063C	1.78782		2.85186						4.63968
GLN3	YER082C				2.48548		1.89659			4.38207
GLN3	YER175C	4.2007			2.68289				0.688352	7.57194
GLN3	YER176W	4.2007								4.2007
GLN3	YER188C-A	5.51171								5.51171
GLN3	YER189W	5.51171			0.882602			1.55229		7.9466
GLN3	YER190W	5.51171			0.882602			1.55229		7.9466
GLN3	YFL021W	4.47641	0.180771	2.42212						7.0793
GLN3	YFL022C	4.47641	0.180771	2.32388						6.98106
GLN3	YFR007W	1.21309		3.46277						4.67586
GLN3	YGL001C	3.74457		0.605381	0.462492		1.544			6.35644
GLN3	YGL008C	2.28811		4.90705						7.19516
GLN3	YGL255W	5.79714								5.79714
GLN3	YGR090W				0.615589	3.9249				4.54049
GLN3	YGR145W			3.31044	2.47808					5.78852
GLN3	YGR155W				2.58769		1.5985			4.18619
GLN3	YGR189C	1.05247		2.66358	0.697002					4.41305
GLN3	YHL015W	2.10579	0.180771	7.93867	0.377645					10.6029
GLN3	YHL016C	2.10579	0.180771	5.42579						7.71235
GLN3	YHR018C	2.27857		0.22476	0.722045		1.55199			4.77736
GLN3	YHR052W	2.71471		1.61114	2.58308					6.90893
GLN3	YHR088W	0.17171			2.61156		1.89541			4.67868
GLN3	YHR148W		0.180771	1.38543	2.70375					4.26995
GLN3	YIL079C				2.50543		1.93395			4.43938
GLN3	YIR027C	2.36484		1.30003			1.37444			5.03931
GLN3	YIR028W	2.36484		2.98792						5.35276
GLN3	YIR029W	4.73157	0.180771	9.49378						14.4061
GLN3	YIR030C	2.4083		7.59977						10.0081
GLN3	YIR031C	4.15439	0.180771	1.77728						6.11244
GLN3	YIR032C	4.15439	0.180771	4.62557					0.699891	9.66062
GLN3	YIR033W	4.15439	0.180771	3.7412						8.07636
GLN3	YJL033W			4.18454	2.53597					6.72051
GLN3	YJL088W	2.40226			0.344591		1.44112	0.89326	0.689725	5.77096
GLN3	YJL148W	2.05094			2.52853					4.57947
GLN3	YJL164C			11.4502						11.4502
GLN3	YJL170C			8.37713						8.37713
GLN3	YJL172W	3.41791	0.180771	5.66137						9.26005
GLN3	YJL173C	3.41791	0.180771	1.54801						5.14669

TF	Target	Binding_0-2000	Motif_Score_SC	Other_Yeasts	Two-Hop_BE	Two-Hop_BP	Two-Hop_BY	Two-Hop_BG	Max_Membership	Sum
GLN3	YJR024C		0.180771	5.37592						5.55669
GLN3	YJR109C	4.08827			0.35237					4.44064
GLN3	YJR110W	4.08827								4.08827
GLN3	YJR148W	4.00767							0.657945	4.66561
GLN3	YJR149W	4.00767								4.00767
GLN3	YJR152W	4.35071		0.10742						4.45813
GLN3	YKL082C				2.5146		1.88282			4.39742
GLN3	YKR038C	1.5174		3.6335						5.1509
GLN3	YKR042W	4.10083								4.10083
GLN3	YKR056W	1.05722		1.08614	3.39258					5.53594
GLN3	YKR092C	5.0041	0.180771	0.353335						5.53821
GLN3	YKR093W	5.0041	0.180771	1.96222						7.14709
GLN3	YLL011W				2.77975		1.87457			4.65432
GLN3	YLL034C	2.04633		1.34021	2.91835					6.30489
GLN3	YLL035W			3.0757	2.47626					5.55196
GLN3	YLR047C	2.82554	0.180771	4.79166						7.79797
GLN3	YLR048W	2.82554	0.180771	4.00407	0.64049					7.65087
GLN3	YLR089C	0.550384	0.180771	6.18645						6.91761
GLN3	YLR090W	0.550384	0.180771	7.40073						8.13189
GLN3	YLR130C	4.38648								4.38648
GLN3	YLR186W	1.03628		1.20949	3.35469					5.60046
GLN3	YLR257W	2.359		1.54883	0.32751				0.571022	4.80636
GLN3	YLR409C				3.42931		1.87457			5.30388
GLN3	YML086C		0.180771	4.13135						4.31212
GLN3	YML092C			4.13108						4.13108
GLN3	YML093W				2.35498		1.87199			4.22697
GLN3	YMR049C		0.180771		3.17978		1.89659			5.25714
GLN3	YMR088C	2.79626	0.180771	6.63354						9.61057
GLN3	YMR108W			2.00128	2.51465		1.51254			6.02847
GLN3	YMR131C				3.14907		1.87798			5.02705
GLN3	YMR210W			5.18625						5.18625
GLN3	YNL025C	0.602305		3.48112						4.08343
GLN3	YNL075W				3.41805		1.89541			5.31346
GLN3	YNL110C				2.88352	0.3776	1.88239			5.14351
GLN3	YNL141W	2.20318		1.04034	2.97043					6.21395
GLN3	YNL142W	4.31149	0.180771	6.09874					0.677427	11.2684
GLN3	YNL175C				2.38449		1.89541			4.2799
GLN3	YNL186W				2.41234		1.87457			4.28691
GLN3	YNL274C			4.13698	0.353695					4.49068
GLN3	YNL330C			4.16189						4.16189
GLN3	YNR003C	2.23259			2.24415					4.47674
GLN3	YNR029C			4.40696						4.40696
GLN3	YNR046W		0.180771	2.21788	2.49813					4.89678
GLN3	YOL041C				2.37477		1.92287			4.29764
GLN3	YOL058W	2.17418			2.81939		1.54598			6.53955
GLN3	YOL076W			5.91258						5.91258
GLN3	YOL077C				2.9386		1.916			4.8546
GLN3	YOL117W	4.10004								4.10004
GLN3	YOL119C	4.10004								4.10004
GLN3	YOR001W	3.03829			3.23358					6.27187
GLN3	YOR119C			2.36705	2.289					4.65605
GLN3	YOR221C	1.51463			2.53501					4.04964
GLN3	YOR303W	0.368883		0.431626		0.938933	1.54278	0.926845		4.20907
GLN3	YOR348C	0.782907		2.73302	0.637823					4.15375
GLN3	YPL043W				3.06016	0.3776	1.89541			5.33317
GLN3	YPL211W			1.62584	2.67934					4.30518
GLN3	YPL263C			0.0407649	2.8445		1.88282			4.76808
GLN3	YPL265W	4.55204							0.531402	5.08344
GLN3	YPR035W	11.5296	0.180771	3.41789						15.1283
GLN3	YPR040W	4.20253								4.20253
GLN3	YPR144C	15.8551		1.72138	0.615431					18.1919
GLN3	YPR145W	15.8551		2.38015						18.2353
GZF3	TR(UCU)E	6.14429								6.14429
GZF3	YCL067C	5.293								5.293
GZF3	YER189W	3.87339			0.547936			1.25707		5.6784
GZF3	YER190W	3.87339			0.547936			1.25707		5.6784
GZF3	YLL066C	3.02364			0.638677			1.25707		4.91939
GZF3	YNL336W	5.44959								5.44959
GZF3	YNL339C	5.44959								5.44959
GZF3	YOR001W	3.65316			0.682426					4.33559
HAC1	TA(AGC)F	4.94098								4.94098
HAC1	YAL013W			4.88155						4.88155
HAC1	YAL015C			4.7979						4.7979
HAC1	YBR033W		0.794603	5.62423						6.41883
HAC1	YBR040W		1.78486	7.17559						8.96045
HAC1	YBR050C			7.4828						7.4828
HAC1	YBR135W			7.28139						7.28139
HAC1	YCL032W			4.06331						4.06331
HAC1	YCL044C	0.859474	2.39593	9.59284	0.392311					13.2406
HAC1	YCR020C-A	1.94894		3.35402						5.30296
HAC1	YCR086W		2.0508	7.47507						9.52587
HAC1	YDL020C			4.39597						4.39597
HAC1	YDL028C			4.36936						4.36936
HAC1	YDL070W		2.14093	5.09931						7.24024
HAC1	YDL072C		2.14093	5.19103						7.33196
HAC1	YDL237W		0.855113	9.20255						10.0577
HAC1	YDR013W		0.794603	4.76864						5.56324
HAC1	YDR217C	3.33746		0.732037						4.0695
HAC1	YDR328C			5.7773						5.7773
HAC1	YDR487C		0.741792	6.22794						6.96973
HAC1	YDR518W	0.409509	0.794603	3.94942						5.15353
HAC1	YDR519W		0.794603	3.36968						4.16428
HAC1	YEL039C		0.461334	3.69316						4.15449
HAC1	YER019C-A		1.06618	4.27514						5.34132
HAC1	YER020W		0.741792	3.71272						4.45451
HAC1	YFR026C	4.94098								4.94098
HAC1	YFR027W	4.94098								4.94098
HAC1	YFR041C	3.5968			0.513016					4.10982
HAC1	YGL224C			4.29517	0.505347					4.80052
HAC1	YGR161C	0.868755	0.973656	3.0504						4.89281
HAC1	YGR285C			4.75064						4.75064
HAC1	YIL048W	5.73266								5.73266
HAC1	YIL154C		2.0508	2.64933						4.70013
HAC1	YIL155C		2.0508	3.43461						5.48541
HAC1	YJL034W	2.47795	3.56936		0.474725					6.52203
HAC1	YJL035C	2.47795	3.56936	18.6824						24.7297
HAC1	YJR017C		0.453812	9.47302						9.92683
HAC1	YJR020W		0.453812	4.45929						4.9131
HAC1	YKL165C	3.68158			0.475606					4.15719
HAC1	YLR021W			4.0608						4.0608

TF	Target	Binding_0-2000	Motif_Score_SC	Other_Yeasts	Two-Hop_BE	Two-Hop_BP	Two-Hop_BY	Two-Hop_BG	Max_Membership	Sum
HAC1	YLR399C			6.13571						6.13571
HAC1	YLR438W		0.862121	7.81507						8.67719
HAC1	YML050W		2.55227	1.47778						4.03005
HAC1	YMR034C		2.0508	2.35106						4.40186
HAC1	YMR114C			3.01985	1.0353					4.05515
HAC1	YMR207C		0.339773	3.67692						4.01669
HAC1	YMR296C		0.453812	4.05246						4.50627
HAC1	YMR297W			4.07935		0.504219				4.58357
HAC1	YNL073W	1.33672		3.22312						4.55984
HAC1	YNL304W		0.332088	7.2887						7.62079
HAC1	YNL328C		1.17325	4.1501						5.32335
HAC1	YNR021W			7.98401						7.98401
HAC1	YOL031C	3.70901			0.490094					4.1991
HAC1	YPL103C			5.48244						5.48244
HAC1	YPL131W			5.08095						5.08095
HAC1	YPR106W		0.405309	3.78928						4.19459
HAL9	SNR37	4.75562								4.75562
HAL9	SNR61	5.38406								5.38406
HAL9	YCR102C	6.16032			1.03599					7.19631
HAL9	YGL001C	6.72504			0.393314		0.793564			7.91192
HAL9	YGR071C	5.4793								5.4793
HAL9	YGR072W	5.4793								5.4793
HAL9	YGR074W	5.4793								5.4793
HAL9	YKL062W	5.05731								5.05731
HAL9	YKL063C	5.05731								5.05731
HAL9	YLR460C	4.5757			1.3597					5.9354
HAL9	YLR461W	4.5757								4.5757
HAL9	YNL132W	3.65918			0.929638					4.58882
HAL9	YOL084W	5.60818								5.60818
HAL9	YOL086C	5.60818					0.924693			6.53287
HAL9	YOR001W	6.11309			0.428369					6.54146
HAP1	TQ(UUG)L	5.88282	0.682464							6.56528
HAP1	TS(AGA)E	6.85144								6.85144
HAP1	TY(GUA)M1	5.1864	1.72252							6.90892
HAP1	YBL043W	2.72657	1.1909	3.34764	1.11646				0.727426	9.109
HAP1	YBL045C	2.72657	1.1909	3.05138	1.49281				0.716258	9.17792
HAP1	YBL098W		1.5613	11.3585						12.9198
HAP1	YBR015C	1.9494	0.599325	1.73897						4.2877
HAP1	YBR016W	1.9494	0.701108	2.72791	0.345825					5.72424
HAP1	YBR082C	5.03545				0.733754				5.7692
HAP1	YBR083W	5.03545			1.03116					6.06661
HAP1	YBR187W			4.08608	0.381112					4.46719
HAP1	YBR265W		0.365314	7.02917						7.39448
HAP1	YCL037C	1.5303	0.761431	5.68805		1.41331				9.39309
HAP1	YCR004C			4.09708					0.474383	4.57146
HAP1	YCR028C-A			6.24965						6.24965
HAP1	YCR079W		1.74604	3.72553		0.337103				5.80867
HAP1	YDL173W	3.84849		0.328063	0.402784					4.57934
HAP1	YDL174C	3.84849			1.51413		0.834758			6.19738
HAP1	YDL178W	0.411932		10.8458	0.608206					11.8659
HAP1	YDR233C	2.64313		0.786786	0.865262					4.29518
HAP1	YDR309C		1.21892	4.57942	0.764026					6.56237
HAP1	YDR492W	2.79706			0.730033				0.712068	4.23916
HAP1	YDR507C		1.17601	3.75747						4.93348
HAP1	YDR524C	2.58919	0.147104					1.65728		4.39357
HAP1	YDR529C	6.22319			1.43329	0.413119			0.706785	8.77638
HAP1	YDR531W	6.22319			0.701867					6.92506
HAP1	YEL024W	5.37505			1.60021		0.939016		0.725572	8.63985
HAP1	YEL025C	5.37505								5.37505
HAP1	YEL032W	6.85144			0.535029		0.973942			8.36041
HAP1	YEL034W	6.85144			1.45763		0.965018		0.720553	9.99464
HAP1	YEL035C	6.85144								6.85144
HAP1	YEL036C	6.85144		2.31628						9.16772
HAP1	YER019C-A		1.41232	10.925	0.471205					12.8085
HAP1	YER043C	4.86332			1.17598				0.730876	6.77018
HAP1	YER044C	4.86332	0.905146		1.59375				0.730991	8.09321
HAP1	YER048C	1.98309	1.23643	3.6606						6.88012
HAP1	YER141W	5.79438			1.22148		0.994406			8.01027
HAP1	YER145C	3.0201	1.40384	10.0436						14.4675
HAP1	YER146W	3.0201	1.40384	8.29945			1.02433			13.7477
HAP1	YER188C-A	4.51029								4.51029
HAP1	YER189W	4.51029			0.678039			1.37184		6.56017
HAP1	YER190W	4.51029			0.678039			1.37184		6.56017
HAP1	YFR032C-A	6.67787	0.336572							7.01444
HAP1	YFR032C-B	6.67787	0.336572							7.01444
HAP1	YFR033C	6.67787	0.336572	0.150299	0.850513				0.627097	8.64235
HAP1	YFR050C		0.712505	2.30935			1.04705			4.0689
HAP1	YGL001C	4.69741			1.56815	1.01442			0.724161	8.00414
HAP1	YGL055W	5.79746	0.599325	1.54218					0.725525	8.66449
HAP1	YGL056C	5.79746	0.599325	1.69305						8.08984
HAP1	YGL071W		1.63867	7.44377						9.08244
HAP1	YGL187C	3.10646	1.41232	10.8984	1.3341				0.708809	17.4601
HAP1	YGL188C-A	3.10646	1.41232							4.51878
HAP1	YGL191W	2.70647			1.43218				0.691166	4.82982
HAP1	YGR047C	3.17653	1.5613			0.742797				5.48063
HAP1	YGR049W	3.17653	1.5613	11.4622	1.25355				0.729189	18.1828
HAP1	YGR060W	4.36791			0.783364	1.10337				6.25464
HAP1	YGR087C	3.90636	1.35162							5.25798
HAP1	YGR088W	3.90636	1.35162	5.65961					0.704611	11.6222
HAP1	YGR110W	4.97094	1.72252							6.69346
HAP1	YGR175C	5.41695			1.54983				0.698268	7.66505
HAP1	YGR183C	3.90492			0.972834				0.523335	5.40109
HAP1	YGR233C	6.13058						1.01913		7.14971
HAP1	YGR234W	6.13058			0.720782					6.85136
HAP1	YGR246C	3.36172	0.784553			0.692806	1.04975			5.88883
HAP1	YGR248W	3.36172	0.0354417		0.409168				0.689036	4.49537
HAP1	YGR249W	1.00993	1.23643	5.93923						8.18559
HAP1	YGR264C	4.35774			0.364041					4.72178
HAP1	YGR266W	4.35774			0.654699					5.01244
HAP1	YGR267C			4.92664						4.92664
HAP1	YHR001W	6.22468								6.22468
HAP1	YHR001W-A	2.3343	0.470722	0.748837	1.21258					4.76644
HAP1	YHR007C	5.75867	1.23643	11.859	0.975987				0.687238	20.5173
HAP1	YHR051W	6.27104	1.17601	5.63776	1.42352				0.66554	15.1739
HAP1	YHR052W	6.27104	1.17601							7.44705
HAP1	YHR188C	4.0268	1.19641	5.41649						10.6397
HAP1	YHR190W	4.0268	1.19641	6.92242	1.28323					13.4289
HAP1	YIL117C			3.63297	0.71901					4.35198
HAP1	YIL153W	4.46868			0.337385					4.80607
HAP1	YIL155C	4.46868							0.703126	5.17181

TF	Target	Binding_0-2000	Motif_Score_SC	Other_Yeasts	Two-Hop_BE	Two-Hop_BP	Two-Hop_BY	Two-Hop_BG	Max_Membership	Sum
HAP1	YJL048C	4.39014			1.03821					5.42835
HAP1	YJL166W	0.625071	0.428668	1.54195	0.955412				0.632304	4.18341
HAP1	YJR047C	6.14974	0.715501	2.4098	1.60097		0.970674		0.670671	12.5174
HAP1	YJR048W	6.14974	0.715501	3.12717	1.59435				0.729424	12.3162
HAP1	YJR104C	5.43747							0.464573	5.90204
HAP1	YJR105W	5.43747		0.852613	0.486937					6.77702
HAP1	YJR106W	5.43747								5.43747
HAP1	YKL018W	2.78446	1.35162							4.13608
HAP1	YKL019W	2.78446	1.35162	7.67275	0.61708					12.4259
HAP1	YKL020C	2.78446	1.35162	7.7211						11.8572
HAP1	YKL051W	4.58107	0.765588	2.5869						7.93356
HAP1	YKL052C	4.58107	0.765588	1.88736						7.23402
HAP1	YKL053C-A	4.58107	0.765588							5.34666
HAP1	YKL096W-A	4.12303	0.503147	1.9235						6.54968
HAP1	YKL150W	0.52086	0.398979		0.934447		0.813368	1.60883	0.486616	4.7631
HAP1	YKL182W	3.03492	0.342972	0.479583	0.698616	0.471201	0.903678	0.983578		6.91455
HAP1	YKR046C	3.99047			1.09148				0.729031	5.81098
HAP1	YLL033W	5.16556	0.628593	0.195173						5.98933
HAP1	YLL034C	5.16556			0.600204	0.808982				6.57475
HAP1	YLR009W		0.215547	5.30136	1.40421		1.02008			7.9412
HAP1	YLR038C		1.03116	5.18653	1.2565				0.698931	8.17312
HAP1	YLR048W			3.69436	0.758948		1.06555			5.51886
HAP1	YLR108C	3.41555	0.405942	1.05574	0.41287					5.2901
HAP1	YLR109W	3.41555	0.405942	1.41647						5.23796
HAP1	YLR110C	3.09862		1.00106						4.09968
HAP1	YLR113W	3.09862	0.0104016	0.988842						4.09786
HAP1	YLR142W	2.09208	1.07518	0.952671	0.852705					4.97264
HAP1	YLR153C	5.72672	1.04248	3.05805	1.57219				0.729785	12.1292
HAP1	YLR154C	5.88282	0.682464	1.6127						8.17798
HAP1	YLR197W		0.848681	7.92575	1.38961		1.04866			11.2127
HAP1	YLR200W			4.26961			1.06153			5.33114
HAP1	YLR205C		1.19641	5.12977	1.44892				0.721251	8.49635
HAP1	YLR206W	6.65185	1.19641	5.14785						12.9961
HAP1	YLR213C	5.46045								5.46045
HAP1	YLR214W	5.46045			0.41566					5.87611
HAP1	YLR254C	5.84551								5.84551
HAP1	YLR256W	5.84551								5.84551
HAP1	YLR289W			4.15283						4.15283
HAP1	YLR382C			4.55826						4.55826
HAP1	YLR395C	4.75661			1.27768				0.664352	6.69864
HAP1	YLR401C		1.27563	4.5085						5.78413
HAP1	YLR403W		1.27563	3.42869						4.70432
HAP1	YLR414C		1.17601	6.48513	0.615592					8.27673
HAP1	YLR417W		1.17601	5.23586						6.41187
HAP1	YML027W	4.17905		1.42497						5.60402
HAP1	YML028W	4.17905	0.0878694	0.71424	0.521854					5.50301
HAP1	YML038C	1.42971	0.31283	3.96674						5.70928
HAP1	YML054C	5.1864	1.72252	5.38997	0.80285	0.348361			0.653793	14.1039
HAP1	YML075C	6.42603			1.40472		1.02389		0.624996	9.47964
HAP1	YML126C	5.25263	0.0192121	0.648932	1.45804		1.00586		0.731267	9.11594
HAP1	YMR015C	4.92236	0.365314	0.823157	1.49468				0.719538	8.32505
HAP1	YMR055C	3.55996	1.63867	3.2448						8.44343
HAP1	YMR056C		1.01925	2.41347	1.27984					4.71256
HAP1	YMR058W	3.55996	1.63867	1.54634					0.706213	7.45118
HAP1	YMR070W	6.44344	1.21892	1.38138						9.04374
HAP1	YMR134W	5.37188	1.43664	0.611086	1.19165					8.61126
HAP1	YMR145C	5.79948			1.51896				0.732463	8.0509
HAP1	YMR201C	5.37354			0.877241					6.25078
HAP1	YMR202W	5.37354			1.45639				0.72728	7.55721
HAP1	YMR203W	5.37354								5.37354
HAP1	YMR211W			4.09219	0.397127					4.48932
HAP1	YMR220W	4.35108	0.728662	0.800405	1.30071					7.18086
HAP1	YMR236W			6.06193						6.06193
HAP1	YMR251W-A	4.09427			0.858006					4.95228
HAP1	YMR256C	2.80427	0.572363		0.755952				0.728374	4.86096
HAP1	YMR282C	6.6691	1.07799	4.4191						12.1662
HAP1	YMR302C	1.74017	1.34894		1.28292				0.463267	4.8353
HAP1	YNL051W	4.73761								4.73761
HAP1	YNL052W	4.73761			1.58712				0.730557	7.05529
HAP1	YNL054W			7.0809						7.0809
HAP1	YNL111C	5.85437			1.25199				0.730662	7.83702
HAP1	YNL155W	4.58948								4.58948
HAP1	YNL156C	4.58948			1.08024					5.66972
HAP1	YNL263C			6.01393	0.412962					6.42689
HAP1	YNL314W		1.17601	4.52484						5.70085
HAP1	YNL315C		1.17601	5.10946						6.28547
HAP1	YNL336W	5.70259								5.70259
HAP1	YNL339C	5.70259								5.70259
HAP1	YNR019W	3.94476			1.35778					5.30254
HAP1	YNR041C	3.54754	0.55144	1.65156	0.497707		0.867816			7.11606
HAP1	YNR043W	3.54754	0.55144	0.964196	1.56868				0.731437	7.36329
HAP1	YNR060W	1.94667	1.10153	4.2817	1.45413					8.78403
HAP1	YOL113W	2.82832	1.15333	5.05908						9.04073
HAP1	YOL114C	2.82832	1.15333	6.03478						10.0164
HAP1	YOR008C		1.17601	6.11133						7.28734
HAP1	YOR018W	2.49428	0.905146	5.85254						9.25197
HAP1	YOR038C		1.37353	3.05082						4.42435
HAP1	YOR040W		1.37353	1.49965			0.826634		0.52738	4.22719
HAP1	YOR064C	7.24338	1.63867	17.4243						26.3063
HAP1	YOR065W	7.24338	1.63867	13.0875	1.37096		0.824282		0.729882	24.8947
HAP1	YOR099W		0.113236	5.9181						6.03134
HAP1	YOR316C	4.75386		0.862297						5.61616
HAP1	YOR316C-A	4.75386								4.75386
HAP1	YOR317W	4.75386		0.816745	1.37917				0.481899	7.43167
HAP1	YOR348C	3.16983	1.86415		0.634793				0.683086	6.35186
HAP1	YOR349W	3.16983	1.86415							5.03398
HAP1	YOR356W	3.89288			1.29866					5.19154
HAP1	YPL027W	4.37293								4.37293
HAP1	YPL028W	4.37293		0.384188	1.18975		0.804205			6.75107
HAP1	YPL212C			1.84195	1.27538		0.930992			4.04832
HAP1	YPL224C		0.983948	3.40281						4.38676
HAP1	YPL231W	2.35564	0.738214		0.371775	0.654575		1.10598		5.22618
HAP1	YPR010C	4.31269	0.675833		0.774423	1.03922				6.80217
HAP1	YPR063C	3.91776	0.493813	0.399056						4.81063
HAP1	YPR065W	3.91776	0.493813	1.36864	0.463563					6.24378
HAP1	YPR066W	3.91776					0.871041			4.7888
HAP1	YPR124W	0.515874		2.65375	1.04474					4.21436
HAP1	YPR144C		1.25062	2.9786	0.734753					4.96397
HAP1	YPR145W		1.25062	5.28151						6.53213
HAP1	YPR151C	7.53479	0.657711	1.29487						9.48737

TF	Target	Binding_0-2000	Motif_Score_SC	Other_Yeasts	Two-Hop_BE	Two-Hop_BP	Two-Hop_BY	Two-Hop_BG	Max_Membership	Sum
HAP2	TL(CAA)M	4.19752								4.19752
HAP2	YBL043W	3.03988			1.65231					4.69219
HAP2	YBR126C				2.27481	1.74324				4.01805
HAP2	YBR155W				0.893659	3.45241				4.34607
HAP2	YDL082W	5.06154			0.832584					5.89412
HAP2	YDL170W	6.66583								6.66583
HAP2	YDL171C	6.66583								6.66583
HAP2	YDR041W	2.41183			1.8945		1.74693			6.05326
HAP2	YDR210W	5.22791								5.22791
HAP2	YDR519W				2.49607		1.79525			4.29132
HAP2	YER174C	13.3686					1.77848			15.1471
HAP2	YER175W-A	13.3686								13.3686
HAP2	YFL021W	8.76277								8.76277
HAP2	YFL022C	8.76277								8.76277
HAP2	YGL187C	4.44453			1.32839					5.77292
HAP2	YGL188C-A	4.44453								4.44453
HAP2	YGL189C				2.6171	3.00813				5.62523
HAP2	YGL220W	0.611717				2.66268	1.60407			4.87847
HAP2	YGR121C	4.20693								4.20693
HAP2	YGR122W	4.20693								4.20693
HAP2	YGR183C	3.74861			0.541617					4.29023
HAP2	YGR233C	4.7258					1.0646			5.7904
HAP2	YGR234W	4.7258								4.7258
HAP2	YGR262C	0.188354				2.66268	1.73929			4.59032
HAP2	YHL016C	4.10206			0.942581					5.04464
HAP2	YHR001W-A	4.42909			0.731291					5.16038
HAP2	YHR002W	4.42909								4.42909
HAP2	YHR016C	4.46707								4.46707
HAP2	YHR051W	6.24314			0.960988					7.20413
HAP2	YHR052W	6.24314			0.658582					6.90172
HAP2	YHR196W				1.37816	2.6709				4.04906
HAP2	YIR029W	8.55755			0.525201					9.08275
HAP2	YIR031C	7.77356								7.77356
HAP2	YIR032C	7.77356								7.77356
HAP2	YIR033W	7.77356								7.77356
HAP2	YLL026W	13.3991								13.3991
HAP2	YLL027W	13.3991					1.77491			15.174
HAP2	YLR049C	9.73803								9.73803
HAP2	YLR052W	9.73803								9.73803
HAP2	YLR196W				1.80446	2.71333				4.51779
HAP2	YLR220W	11.4017								11.4017
HAP2	YMR117C	4.19752				0.853246				5.05077
HAP2	YMR118C	4.19752								4.19752
HAP2	YMR119W	4.19752			0.649986		1.16474			6.01225
HAP2	YNL030W	3.7909			1.09214					4.88304
HAP2	YOR027W				2.07767	3.45241	1.11426			6.64434
HAP2	YOR267C	4.14689								4.14689
HAP2	YOR268C	4.14689								4.14689
HAP2	YOR269W	4.14689								4.14689
HAP2	YOR375C	5.437								5.437
HAP2	YOR376W-A	5.437								5.437
HAP2	YPL207W	11.367								11.367
HAP2	YPL235W						1.59756	2.50338		4.10094
HAP2	YPR144C	3.18047			1.77253					4.953
HAP3	YDR519W				2.59422		2.08719			4.68141
HAP3	YER174C	15.8551					1.83489			17.69
HAP3	YER175W-A	15.8551								15.8551
HAP3	YGL187C	6.45962			0.685057					7.14468
HAP3	YGL188C-A	6.45962								6.45962
HAP3	YGL189C				3.12503	3.13784				6.26287
HAP3	YGL220W					3.33393	1.89601			5.22994
HAP3	YGR262C	0.218044				3.33393	1.7957			5.34767
HAP3	YHR001W-A	4.22649			0.909172					5.13566
HAP3	YHR002W	4.22649								4.22649
HAP3	YHR051W	3.09374			1.37262					4.46636
HAP3	YKL027W				2.46882		1.64133			4.11015
HAP3	YLL026W	13.8795								13.8795
HAP3	YLL027W	13.8795					2.06684			15.9463
HAP3	YLR196W				1.88021	2.81343				4.69364
HAP3	YLR220W	13.2555								13.2555
HAP3	YOR027W				2.17582	3.58212				5.75794
HAP3	YPL207W	11.7378								11.7378
HAP3	YPL235W						1.65398	2.58997		4.24395
HAP4	TP(UGG)H	4.53245								4.53245
HAP4	YAL062W		0.554522	3.33789	1.58277					5.47518
HAP4	YAR014C	4.30317								4.30317
HAP4	YAR015W	4.30317								4.30317
HAP4	YBL015W		0.554522	5.49254	1.19673				0.699562	7.94335
HAP4	YBL030C	2.06199		1.86722	2.21244				0.676386	6.81804
HAP4	YBL043W	5.17924			2.30608				0.727426	8.21275
HAP4	YBL044W	5.17924								5.17924
HAP4	YBL045C	5.17924			1.77404	0.722014	1.22823		0.716258	9.61978
HAP4	YBL099W	5.78449	0.554522	4.28237	0.53972	2.72802			0.709026	14.5981
HAP4	YBR039W	10.7157			0.462852	1.39683			0.592875	13.1683
HAP4	YBR142W	0.309341		0.135599	2.70267	2.32958				5.47719
HAP4	YBR143C		0.554522	3.98967	0.940212					5.4844
HAP4	YBR145W			4.8309						4.8309
HAP4	YBR230C			1.8969	1.97841				0.688062	4.56337
HAP4	YCL054W	1.46765			2.76241					4.23006
HAP4	YCL057C-A		0.303177	5.00325						5.30643
HAP4	YCL057W		0.303177	4.88387						5.18705
HAP4	YCR016W			1.98783	2.32543					4.31326
HAP4	YCR083W			4.88819						4.88819
HAP4	YDL004W	2.50417		11.7399	1.82772	1.39683	1.2683		0.72703	19.464
HAP4	YDL005C	2.50417		12.5196		0.471048				15.4948
HAP4	YDL066W	6.96366	0.554522	9.20951						16.7277
HAP4	YDL067C	6.96366	0.554522	5.13146	1.51615	2.95631				17.1221
HAP4	YDL069C	6.96366	0.554522	8.5931						16.1113
HAP4	YDL180W	6.33774	0.554522							6.89226
HAP4	YDL181W	6.33774	0.554522	7.00981	1.66717	1.39683			0.684421	17.6505
HAP4	YDL213C				1.67747	2.32958				4.00705
HAP4	YDR237W	2.90067			1.41696					4.31763
HAP4	YDR253C	1.88787					1.29449	1.04066		4.22302
HAP4	YDR275W			5.35387						5.35387
HAP4	YDR298C	4.53132			1.79776	1.39683	1.2886		0.686686	9.7012
HAP4	YDR299W	4.53132			2.33004					6.86136
HAP4	YDR377W	9.23346		2.56074		1.39683				13.191
HAP4	YDR379W	9.23346								9.23346
HAP4	YDR525W-A		0.554522	9.16009						9.71461
HAP4	YDR527W		0.554522	11.2171						11.7716

TF	Target	Binding_0-2000	Motif_Score_SC	Other_Yeasts	Two-Hop_BE	Two-Hop_BP	Two-Hop_BY	Two-Hop_BG	Max_Membership	Sum
HAP4	YDR529C	8.85293	0.303177	4.78526	2.63108				0.706785	17.2792
HAP4	YDR531W	8.85293	0.303177							9.15611
HAP4	YDR536W			5.60867	1.73377				0.702581	8.04502
HAP4	YDR545W	4.48527								4.48527
HAP4	YEL024W	9.25466	0.303177	4.36274	2.08377				0.725572	16.7299
HAP4	YEL025C	9.25466	0.303177	1.96706						11.5249
HAP4	YER049W	2.87451			1.82535					4.69986
HAP4	YER079W			3.64354	0.762821					4.40636
HAP4	YER100W	0.415216			1.15115	1.84808	1.14759			4.56204
HAP4	YFR033C	1.72365			1.91426				0.627097	4.26501
HAP4	YFR044C			5.59438	1.77317					7.36755
HAP4	YFR045W			5.30851			1.29449	2.09488		8.69788
HAP4	YFR047C			4.15792					0.663026	4.82095
HAP4	YGL001C	3.84833			2.09099				0.724161	6.66348
HAP4	YGL032C			4.36938						4.36938
HAP4	YGL040C		0.423625	7.70451	1.43257					9.56071
HAP4	YGL078C			1.07031	2.64891				0.708765	4.42798
HAP4	YGL187C	12.2672		0.262042	2.53189	1.52462			0.708809	17.2946
HAP4	YGL188C-A	12.2672	0.303177							12.5704
HAP4	YGL191W	2.68624			2.62997				0.691166	6.00738
HAP4	YGR113W			3.93565	0.596749					4.5324
HAP4	YGR175C		0.303177	3.62368	1.50789		0.961271		0.698268	7.09429
HAP4	YGR183C	10.715			1.78979				0.523335	13.0281
HAP4	YGR272C	2.16535			2.27849					4.44384
HAP4	YHR001W-A	10.353			2.09548					12.4485
HAP4	YHR002W	10.353								10.353
HAP4	YHR005C		0.554522	8.05065			1.20971			9.81488
HAP4	YHR051W	12.1347		2.54931	2.55895	1.52462			0.66554	19.4331
HAP4	YHR052W	12.1347			0.776862					12.9116
HAP4	YHR066W				2.66114	2.92055				5.58169
HAP4	YHR144C	4.53245								4.53245
HAP4	YHR146W	4.53245								4.53245
HAP4	YHR151C		0.303177	4.4997						4.80288
HAP4	YHR170W	0.796812			2.5752				0.683679	4.05569
HAP4	YHR191C	4.99681			1.12546					6.12227
HAP4	YHR193C	4.99681		0.529245						5.52605
HAP4	YHR194W	4.99681		0.505799						5.50261
HAP4	YHR197W				2.54111	1.94651				4.48762
HAP4	YIL035C	1.99553		0.176628	1.05673	2.32958				5.55847
HAP4	YJL058C	3.62884				1.16189				4.79073
HAP4	YJL167W			3.04456	2.04638				0.461926	5.55287
HAP4	YJR019C		0.423625	3.6188						4.04242
HAP4	YJR052W			4.43963			1.08168			5.52131
HAP4	YJR077C	9.10185		1.83104	1.95697				0.717983	13.6078
HAP4	YJR078W	9.10185		1.89199					0.441297	11.4351
HAP4	YJR079W	9.10185			1.09757				0.560305	10.7597
HAP4	YJR088C	1.07811		2.30433	1.03173					4.41417
HAP4	YJR097W	4.60504								4.60504
HAP4	YJR098C	0.17946		7.01296						7.19242
HAP4	YJR119C	5.17544	0.554522	10.8712						16.6012
HAP4	YJR121W	5.17544	0.554522	9.44349	0.576179	2.72802			0.693375	19.171
HAP4	YKL016C	3.09491		1.05131	2.04617	2.3279			0.639673	9.15996
HAP4	YKL017C	3.09491		0.986917						4.08183
HAP4	YKL141W	0.802334		2.69248	1.54539		1.16945		0.667605	6.87726
HAP4	YKL146W	1.56931	0.303177	2.81757			1.20971			5.89977
HAP4	YKL148C	1.56931	0.303177	3.75093	1.37802				0.718989	7.72043
HAP4	YKL172W	0.185564			2.50344	2.32958				5.01858
HAP4	YKL212W		0.554522	4.27083						4.82535
HAP4	YKL213C		0.554522	3.51634						4.07086
HAP4	YKR036C	0.510654			2.19995	1.94651				4.65711
HAP4	YKR048C		0.423625	4.63404						5.05767
HAP4	YKR049C		0.423625	3.75125	1.24198					5.41685
HAP4	YKR081C				2.70817	2.92055				5.62872
HAP4	YLL019C		0.423625	3.93637					0.594492	4.95449
HAP4	YLR010C			6.42195						6.42195
HAP4	YLR038C	3.58111	0.303177	2.97055	2.45429				0.698931	10.0081
HAP4	YLR074C	0.162987			1.84984	2.92055				4.93338
HAP4	YLR168C	1.73388	0.303177	3.94649	0.505273				0.45671	6.94553
HAP4	YLR173W		0.303177	5.15067						5.45385
HAP4	YLR193C			7.7179						7.7179
HAP4	YLR196W	0.787361			2.56466		0.904423		0.6569	4.91334
HAP4	YLR220W			5.5357						5.5357
HAP4	YLR289W	3.29253						0.907982		4.20051
HAP4	YLR293C	6.31164	0.423625							6.73527
HAP4	YLR295C	6.31164	0.423625		1.49473	1.39683				9.62683
HAP4	YLR297W	6.31164	0.423625						0.499278	7.23454
HAP4	YLR335W				2.33947	1.53914	1.2608			5.13941
HAP4	YLR395C	2.79451		0.0944071	2.57933				0.664352	6.1326
HAP4	YML042W				1.20803	2.25321			0.707445	4.16868
HAP4	YML087C		0.303177	4.48563					0.548371	5.33718
HAP4	YML119W	1.41415	0.303177	4.45244						6.16977
HAP4	YML120C	1.41415	0.303177	4.30193	1.20696				0.7193	7.94552
HAP4	YMR049C				2.25397	2.32958				4.58355
HAP4	YMR074C			7.11743						7.11743
HAP4	YMR229C				1.91423	2.32958				4.24381
HAP4	YMR241W			2.81407			1.20971			4.02378
HAP4	YMR256C	8.03367		1.56549	1.66806				0.728374	11.9956
HAP4	YMR280C		0.303177	3.21611				0.935889		4.45518
HAP4	YMR281W		0.303177	4.26884						4.57202
HAP4	YNL048W			2.95017			1.22195			4.17212
HAP4	YNL051W	8.85763	0.554522							9.41215
HAP4	YNL052W	8.85763	0.554522	10.7433	2.45338	1.52462			0.730557	24.864
HAP4	YNL055C	2.3222	0.423625	6.59363					0.661697	10.0012
HAP4	YNL061W				2.80222	2.92055				5.72277
HAP4	YNL068C			2.80773	1.2176		1.25599			5.28132
HAP4	YNL110C				2.5171	2.92055				5.43765
HAP4	YNL132W				2.13635	2.32958				4.46593
HAP4	YNL175C				2.18883	2.32958				4.51841
HAP4	YNL336W	6.8563			1.08913					7.94543
HAP4	YNL339C	6.8563								6.8563
HAP4	YOL076W	6.31119	0.303177							6.61437
HAP4	YOL077W-A		0.303177	9.94323						10.2464
HAP4	YOL096C			3.36203	0.409423		1.29699			5.06844
HAP4	YOR046C			3.85975	0.591447					4.4512
HAP4	YOR064C	5.6973								5.6973
HAP4	YOR065W	5.6973			2.01104		1.17516		0.729882	9.61338
HAP4	YOR187W	1.4058	0.303177	3.35823	1.5883			0.93066	0.640184	8.22635
HAP4	YOR206W				2.7468	2.32958				5.07638
HAP4	YOR252W	0.792711		4.49541	2.42478					7.7129
HAP4	YOR256C	3.47598			0.619621					4.0956

TF	Target	Binding_0-2000	Motif_Score_SC	Other_Yeasts	Two-Hop_BE	Two-Hop_BP	Two-Hop_BY	Two-Hop_BG	Max_Membership	Sum
HAP4	YOR258W	3.47598			0.619621					4.0956
HAP4	YOR272W				2.16826	2.32958	0.872508			5.37035
HAP4	YOR310C				2.45338	2.32958				4.78296
HAP4	YOR324C		0.554522	7.21154		0.6455				8.41156
HAP4	YOR375C			3.61034	1.02494					4.63528
HAP4	YPL024W			5.64638						5.64638
HAP4	YPL026C			5.47653						5.47653
HAP4	YPL043W			0.18942	2.79266	2.92055				5.90263
HAP4	YPL078C			1.95469	0.73544	2.3279			0.503333	5.52136
HAP4	YPL177C			5.27657						5.27657
HAP4	YPL262W	0.318079		6.54976	0.421632				0.56372	7.85319
HAP4	YPL263C	0.318079		4.86087	0.567946					5.7469
HAP4	YPL270W	4.75439								4.75439
HAP4	YPL271W	4.75439			1.07209	1.39683				7.22331
HAP4	YPL272C	4.75439								4.75439
HAP4	YPR020W	4.39112		5.04777	1.46126	1.39683			0.671639	12.9686
HAP4	YPR060C	4.55483			1.70424					6.25907
HAP4	YPR062W	4.55483								4.55483
HAP4	YPR186C	4.17687								4.17687
HAP4	YPR187W	4.17687			1.78643					5.9633
HAP4	YPR189W	4.17687								4.17687
HAP4	YPR190C	3.99053			0.387194					4.37772
HAP4	YPR191W	3.99053			1.81121	1.04291	0.974751		0.726091	8.54549
HAP5	YBR155W	0.478397				3.80125				4.27965
HAP5	YCL043C				2.45515		1.7747			4.22985
HAP5	YDR041W				2.15846		1.89865			4.05711
HAP5	YDR519W				2.76003		1.92069			4.68072
HAP5	YER131W				0.661167	3.35697				4.01814
HAP5	YER174C	14.4371					1.9302			16.3673
HAP5	YER175W-A	14.4371								14.4371
HAP5	YGL189C				2.83535	3.35697				6.19232
HAP5	YGL220W					2.95111	1.72952			4.68063
HAP5	YGR052W	2.91884			2.36824					5.28708
HAP5	YGR119C	0.723222				3.80125				4.52447
HAP5	YGR262C	1.68998				2.95111	1.891			6.53209
HAP5	YKL027W				2.63463		1.73664			4.37127
HAP5	YKL062W	2.4569			2.55163					5.00853
HAP5	YLL026W	14.6912								14.6912
HAP5	YLL027W	14.6912					1.90035			16.5916
HAP5	YLR049C	5.16781								5.16781
HAP5	YLR052W	5.16781								5.16781
HAP5	YLR196W	0.200118			1.58381	2.42173				4.20566
HAP5	YLR220W	7.6901								7.6901
HAP5	YOR027W				2.34163	3.80125				6.14288
HAP5	YPL207W	10.2868								10.2868
HAP5	YPL235W						1.74928	2.73624		4.48552
HAP5	YPR103W	0.182827			2.05952		1.8384			4.08075
HIR1	YBL002W	1.25099			2.18473		1.26144		0.730527	5.42769
HIR1	YBL003C	1.25099			2.23107		1.25214		0.732381	5.46658
HIR1	YBL063W				1.64938		1.27258	1.67373		4.59569
HIR1	YBR009C	10.4554			2.19479	2.65777	1.27913		0.729277	17.3164
HIR1	YBR010W	10.4554			2.19479	2.65777	1.27913		0.72791	17.315
HIR1	YCL059C				2.49736		1.24457		0.524423	4.26635
HIR1	YCL067C	4.02102								4.02102
HIR1	YDR224C	3.23905			2.24399	2.65777	1.26144		0.728946	10.1312
HIR1	YDR225W	3.23905			2.20182	2.65777	1.25214		0.731721	10.0825
HIR1	YDR227W	0.949584				2.65777	1.30643			4.91378
HIR1	YDR300C				0.671864	2.46723	1.24104		0.606901	4.98703
HIR1	YDR386W	2.08603				1.87626	1.45916			5.42145
HIR1	YEL001C	4.03722								4.03722
HIR1	YEL002C	4.03722								4.03722
HIR1	YEL056W				1.35158	2.65777	1.27835			5.2877
HIR1	YFR001W	4.54221			0.417012					4.95922
HIR1	YFR002W	4.54221			1.40479					5.947
HIR1	YJL081C					2.65777	1.30651		0.593101	4.55738
HIR1	YLR002C	2.89411			1.7791					4.67321
HIR1	YLR061W	6.53549			1.44935				0.711197	8.69604
HIR1	YLR063W	6.53549								6.53549
HIR1	YLR167W	1.97273			1.89471		1.36881		0.730777	5.96703
HIR1	YLR185W				2.49657		1.25344		0.72006	4.47007
HIR1	YLR340W				1.52498	1.87626	1.31043		0.701701	5.41337
HIR1	YMR308C				1.69392	2.46723				4.16115
HIR1	YNL030W	7.18504			2.31234		1.27913		0.731422	11.5079
HIR1	YNL031C	7.18504			2.21981		1.27913		0.731267	11.4152
HIR1	YPL253C	6.18568								6.18568
HIR1	YPR110C				2.132	2.46723			0.670477	5.26971
HIR2	YBL002W	2.03377			1.64225		0.949629		0.730527	5.35618
HIR2	YBL003C	1.77571			1.68859		0.940332		0.732381	5.13701
HIR2	YBR009C	7.79964			1.66441	1.94083	0.967325		0.729277	13.1015
HIR2	YBR010W	7.79964			1.65228	1.94083	0.967325		0.72791	13.088
HIR2	YCL067C	4.30575								4.30575
HIR2	YDR224C	2.35524			1.70148	1.94083	0.949629		0.728946	7.67612
HIR2	YDR225W	2.35524			1.65934	1.94083	0.940332		0.731721	7.62746
HIR2	YJL191W	4.03309			0.931583		0.866361			5.83103
HIR2	YLR340W				1.50457	1.15932	0.998618		0.701701	4.36421
HIR2	YNL030W	7.2836			1.76984		0.967325		0.731422	10.7522
HIR2	YNL031C	7.2836			1.6773		0.967325		0.731267	10.6595
HIR2	YPR110C				1.5895	1.75029			0.670477	4.01027
HIR3	SNR53	4.33104								4.33104
HIR3	TK(CUU)C	4.47621								4.47621
HIR3	TR(UCU)E	4.79753								4.79753
HIR3	YBR009C	4.1677			1.06977	1.13501			0.729277	7.10176
HIR3	YBR010W	4.1677			1.09171	1.13501			0.72791	7.12233
HIR3	YCR019W	4.47621								4.47621
HIR3	YCR096C	3.35926				0.742134				4.10139
HIR3	YCR097W	3.35926				0.742134				4.10139
HIR3	YDR224C	4.81459			1.26281	1.13501			0.728946	7.94136
HIR3	YDR225W	4.81459			1.26281	1.13501			0.731721	7.94413
HIR3	YDR226W	4.81459								4.81459
HIR3	YEL050C	4.33104								4.33104
HIR3	YGL001C	5.62458			0.407103				0.724161	6.75584
HIR3	YLR048W	3.59523			0.73587					4.3311
HIR3	YMR119W	3.21903			1.21124					4.43027
HIR3	YNL030W	2.62805			1.12447	0.616353			0.731422	5.1003
HIR3	YNL031C	2.62805			1.05839				0.728083	4.41452
HIR3	YOR001W	4.08418			0.538913					4.62309
HMS1	YBR133C	8.30947								8.30947
HMS1	YBR135W	8.30947								8.30947
HMS1	YBR136W	8.30947								8.30947
HMS2	TK(CUU)E1	4.97089								4.97089

TF	Target	Binding_0-2000	Motif_Score_SC	Other_Yeasts	Two-Hop_BE	Two-Hop_BP	Two-Hop_BY	Two-Hop_BG	Max_Membership	Sum
HMS2	TR(UCU)E	4.97089								4.97089
HMS2	YDR078C	4.81617								4.81617
HMS2	YDR080W	4.81617								4.81617
HMS2	YDR282C	4.62283								4.62283
HMS2	YER010C	11.2369								11.2369
HMS2	YER011W	11.2369				0.368643				11.6055
HMS2	YER043C	4.31591			0.87702					5.19293
HMS2	YER044C	4.31591								4.31591
HMS2	YGR249W	4.31348								4.31348
HMS2	YIL118W	5.67093			0.600225					6.27116
HMS2	YIL119C	5.67093								5.67093
HMS2	YJR088C	6.95953								6.95953
HMS2	YJR089W	6.95953								6.95953
HMS2	YKR042W	4.54699			0.611313					5.1583
HMS2	YMR016C	4.31131								4.31131
HMS2	YMR017W	4.31131								4.31131
HMS2	YOR365C	5.12241								5.12241
HMS2	YOR368W	5.12241								5.12241
HMS2	YPL178W	1.97573			0.645724	2.86874				5.49019
HSF1	SNR18	5.52588	0.63953							6.16541
HSF1	YAL003W	5.52588	0.63953	0.392739						6.55815
HSF1	YAL005C	5.52588	0.63953	11.5576	1.33199	1.5437	0.805255			21.404
HSF1	YAL026C			3.45108			0.877065			4.32815
HSF1	YBL075C	3.62202	0.228615	2.94462	1.45732		0.805255		0.732686	9.79052
HSF1	YBR037C	0.670865		3.99065						4.66151
HSF1	YBR060C	2.16157		1.84014						4.00171
HSF1	YBR070C	4.18058	0.839899							5.02048
HSF1	YBR072W	4.18058	0.839899	16.1287	0.402073				0.726942	22.2782
HSF1	YBR101C	6.25523	0.202449	5.68597	1.40544	1.035			0.726561	15.3107
HSF1	YBR117C	1.96683	0.145118	1.6282					0.578465	4.31861
HSF1	YBR118W	1.96683	0.145118	2.17203	0.353736	1.13649				5.7742
HSF1	YBR165W	0.87393	0.176821	3.4572						4.50795
HSF1	YBR169C	3.32643		5.39333	1.31247				0.728817	10.761
HSF1	YBR285W			5.26675					0.697532	5.96428
HSF1	YCL050C	3.65537	0.819355	18.4594	0.720804					23.6549
HSF1	YCL059C	2.96832			1.05583		0.924446			4.9486
HSF1	YCR011C		0.660735	12.3179					0.454956	13.4336
HSF1	YCR012W		0.660735	14.5595	0.377016	1.35316			0.469475	17.4199
HSF1	YDL020C	5.33704	0.326959	5.72081	0.567977				0.725223	12.678
HSF1	YDL037C	1.57478		2.26507				1.59582		5.43567
HSF1	YDR011W	4.30012								4.30012
HSF1	YDR070C	2.56787				0.919009			0.702584	4.18946
HSF1	YDR151C	5.91502								5.91502
HSF1	YDR152W	5.91502	0.402778		0.622543					6.94034
HSF1	YDR153C	4.498								4.498
HSF1	YDR155C	4.498		0.886969	0.925172	0.581799				6.89194
HSF1	YDR156W	4.498		0.894714	0.715125					6.10784
HSF1	YDR158W	4.498			0.557067					5.05507
HSF1	YDR171W	5.14993	0.496926	3.12863	0.917707	1.35316			0.727755	11.7741
HSF1	YDR214W	5.72146	0.0978963	5.30792	1.17988				0.732745	13.0399
HSF1	YDR258C	6.153	0.609708	2.48155	1.10641				0.721902	11.0726
HSF1	YDR313C	2.00881		0.87837		0.687206	0.789367			4.36375
HSF1	YER024W		0.465717	5.29137						5.75709
HSF1	YER033C	5.00076							0.705124	5.70588
HSF1	YER035W	5.00076			0.599515					5.60027
HSF1	YER103W	6.12579	0.230573	7.1878	1.39374		0.805255		0.730031	16.4732
HSF1	YFL014W	5.6603		0.518192	0.601363				0.681848	7.4617
HSF1	YFL016C	5.6603		0.526003	0.813453	1.49337	0.833519	1.17667	0.6778	11.1811
HSF1	YFL017C	5.6603					0.930715			6.59102
HSF1	YFL037W	3.62632			0.444809					4.07113
HSF1	YFL039C	3.62632		1.70842		0.366238	0.789367			6.49035
HSF1	YFL056C	1.91207		1.16607	0.798937			0.903649		4.78073
HSF1	YGL001C			3.62739	0.44713					4.07452
HSF1	YGL115W			5.83842	0.654476					6.4929
HSF1	YGL130W	0.40775		1.92657	1.25638	1.29844				4.88914
HSF1	YGR112W		0.3276	3.81631						4.14391
HSF1	YGR142W	6.25674	0.326959	6.42385	1.40512				0.729515	15.1422
HSF1	YGR146C	3.1604	0.202449	4.02652	0.735466					8.12484
HSF1	YGR161C	3.67243			0.768127				0.694915	5.13547
HSF1	YGR192C	3.9693	0.27347		1.03317		0.869537			6.14548
HSF1	YGR209C	6.2363	0.274117							6.51042
HSF1	YGR210C	6.2363	0.274117	4.30102						10.8114
HSF1	YGR211W	6.2363	0.274117	2.93228			0.840431			10.2831
HSF1	YGR214W			4.62669	0.968126		0.933134			6.52795
HSF1	YGR215W		0.274117	4.60884						4.88296
HSF1	YHR024C		0.660735	7.79908						8.45982
HSF1	YHR026W		0.660735	4.81464						5.47537
HSF1	YHR082C	3.5554		0.500755					0.7111	4.76726
HSF1	YHR104W	2.43364	0.381219	1.43931	1.06646				0.730158	6.05079
HSF1	YHR174W	2.17908	0.636494	6.49691	0.37875	1.35316				11.0444
HSF1	YHR175W	2.17908	0.636494	1.56685						4.38242
HSF1	YJL034W	5.44039	0.427407		0.992899		0.836502		0.72649	8.42369
HSF1	YJL035C	5.44039	0.427407	4.58131	0.418294					10.8674
HSF1	YJL073W	2.10941			0.950195			1.14078		4.20038
HSF1	YJL143W	4.53944				1.49337				6.03281
HSF1	YJL144W	4.53944		0.770422	0.379802				0.707178	6.39684
HSF1	YJR009C			5.50871	1.10205					6.61076
HSF1	YJR045C	6.14209		0.604078	1.09498	1.36331	0.808248		0.709971	10.7227
HSF1	YJR046W	6.14209	0.329524	9.20455	0.604396				0.647066	16.9276
HSF1	YKL009W	2.13058			1.13402		0.888774			4.15337
HSF1	YKL010C	2.13058					0.789367	1.20366		4.12361
HSF1	YKL035W	3.5686			0.391941				0.666162	4.6267
HSF1	YKR072C		0.0985641	5.05627						5.15483
HSF1	YLL024C	1.05308	0.609708	8.26318	1.42152	1.26629	0.805255		0.670155	14.0892
HSF1	YLL026W		0.0978963	2.32424	1.56753	1.17882			0.731132	5.89962
HSF1	YLL039C	6.05076	0.819355	5.91961	0.788812				0.731844	14.3104
HSF1	YLR042C	6.38244							0.45779	6.84023
HSF1	YLR108C	4.04289		0.914568						4.95746
HSF1	YLR109W	4.04289		0.904865	0.555195					5.50295
HSF1	YLR215C	5.9507	0.287697							6.2384
HSF1	YLR216C	5.9507	0.287697	4.64064	1.59447	1.52636	0.896619		0.732698	15.6292
HSF1	YLR219W	5.9507	0.287697	4.43513						10.6735
HSF1	YLR259C	5.61688		0.379036	1.39809				0.729187	8.12319
HSF1	YLR260W	5.61688		0.382858						5.99974
HSF1	YLR293C	2.46867		3.11492		0.511177				6.09477
HSF1	YLR327C	2.24077		1.88501	0.427044				0.719147	5.27197
HSF1	YLR449W		0.260505	4.40216						4.66267
HSF1	YML100W	2.10923	0.0185348	0.62267	0.950486		0.802565		0.730709	5.23419
HSF1	YML130C	2.35951	0.0219202	1.28027	0.869778					4.53148
HSF1	YMR186W	6.26431		1.25558	1.53041	1.4417			0.53193	11.0239

TF	Target	Binding_0-2000	Motif_Score_SC	Other_Yeasts	Two-Hop_BE	Two-Hop_BP	Two-Hop_BY	Two-Hop_BG	Max_Membership	Sum
HSF1	YMR258C	2.30752		1.18527					0.591056	4.08385
HSF1	YNL006W	6.32852	0.30704	5.93664	0.900132				0.710288	14.1826
HSF1	YNL007C	6.32852	0.30704	4.80167	1.39062	1.32702	0.78636		0.720858	15.6621
HSF1	YNL008C	6.32852	0.30704		0.509407					7.14497
HSF1	YNL064C	3.44425			1.31415	1.32702	0.784133	1.17067		8.04022
HSF1	YNL077W	5.83553						1.14509		6.98062
HSF1	YNL281W	4.65919			0.838061	0.625158			0.731865	6.85427
HSF1	YNL283C	4.65919		2.45685						7.11604
HSF1	YNR069C	4.50195	0.0623883	1.89984		0.550611				7.01479
HSF1	YOL032W	1.88615	0.63953	12.9434	0.977065				0.731328	17.1775
HSF1	YOL078W	0.660846	0.46196	6.12298			0.839741			8.08553
HSF1	YOL079W		0.248811	5.47226						5.72107
HSF1	YOR006C	4.08164								4.08164
HSF1	YOR007C	4.08164			0.955448					5.03709
HSF1	YOR020C	1.71339		1.60155	1.04289	1.35158			0.72938	6.43879
HSF1	YOR027W	4.9769		1.25296	1.41482	1.52636	0.868033		0.709425	10.7485
HSF1	YOR267C	4.57966								4.57966
HSF1	YOR269W	4.57966								4.57966
HSF1	YOR285W	0.622197		3.60137	0.672145				0.732364	5.62808
HSF1	YOR286W	0.622197		3.58945						4.21165
HSF1	YOR299W	5.96014	0.292216	2.25691						8.50927
HSF1	YOR344C	3.8728	0.496303	5.40472						9.77382
HSF1	YPL005W	2.98575		1.27678						4.26253
HSF1	YPL106C	4.09548	0.397079	10.5677	0.91746		0.805255			16.783
HSF1	YPL237W	5.30091	0.1292		0.806589		0.950253			7.18695
HSF1	YPL239W	5.30091	0.1292	4.30182	0.806589					10.5385
HSF1	YPL240C	5.30091	0.1292	2.0502	1.63692	1.52636			0.729377	11.373
HSF1	YPL250C	4.87768		0.588604						5.46628
HSF1	YPL252C	4.87768			0.637568					5.51525
HSF1	YPR122W	0.997634		3.12339						4.12102
HSF1	YPR158W	6.00125		4.23637	1.24658				0.658478	12.1427
IME1	YDL154W	4.9472								4.9472
IME4	SNR37	5.50057								5.50057
IME4	TK(CUU)C	5.1044								5.1044
IME4	TM(CAU)J3	6.6394								6.6394
IME4	TR(UCU)E	6.73676								6.73676
IME4	YAL017W	4.00068								4.00068
IME4	YAL018C	4.00068								4.00068
IME4	YAL022C	6.36798								6.36798
IME4	YBR057C	4.22283						1.12455		5.34738
IME4	YCR019W	5.1044								5.1044
IME4	YCR097W	3.16947				0.690899	0.778488			4.63886
IME4	YCR105W	5.13042					0.951306			6.08173
IME4	YCR107W	7.18821			0.611289					7.7995
IME4	YDR084C	4.69216								4.69216
IME4	YER044C	3.0361			1.47257					4.50867
IME4	YER048C	3.2197						1.48268		4.70238
IME4	YFL016C	7.56103								7.56103
IME4	YGL001C	6.81205			0.70125					7.5133
IME4	YGL227W	5.00354								5.00354
IME4	YHR191C	4.91842								4.91842
IME4	YHR193C	4.91842								4.91842
IME4	YHR194W	4.91842								4.91842
IME4	YIL004C	5.89527			0.875399					6.77067
IME4	YJR010C-A	5.01432								5.01432
IME4	YJR011C	5.01432								5.01432
IME4	YJR013W	5.01432								5.01432
IME4	YJR044C	6.6394								6.6394
IME4	YJR143C	8.33252								8.33252
IME4	YMR258C	5.575								5.575
IME4	YNR018W	4.02281								4.02281
IME4	YNR019W	4.02281			0.640089					4.6629
IME4	YOR001W	5.2404			0.453344					5.69374
IME4	YPL034W	7.98995								7.98995
IME4	YPL253C	5.45455						0.902598		6.35715
INO2	TE(CUC)D	4.6002								4.6002
INO2	YBL036C			3.00967			1.12579			4.13546
INO2	YBR029C	6.05352		0.568071			1.12852			7.75011
INO2	YBR030W	6.05352		3.22648	0.599904					9.8799
INO2	YBR164C		0.292216	4.09261						4.38483
INO2	YBR177C	0.321522	0.292216	4.27501						4.88875
INO2	YCR057C	2.90978	0.292216	2.12663						5.32863
INO2	YCR060W	2.90978	0.292216	1.26254						4.46454
INO2	YCR098C	3.65192	0.292216	0.336423						4.28056
INO2	YDL049C	3.49815		1.23816						4.73631
INO2	YDR146C	4.71237	0.292216	2.62717						7.63176
INO2	YDR147W	4.71237	0.292216	1.38665						6.39124
INO2	YDR231C		0.292216	4.30334						4.59556
INO2	YDR279W	4.6002		0.265482						4.86568
INO2	YDR312W	2.19685	0.292216	2.2072						4.69627
INO2	YDR497C	6.32444	0.292216	0.987451						7.60411
INO2	YER049W			3.777	0.79523					4.57223
INO2	YFR015C			5.99317						5.99317
INO2	YFR028C			4.3146						4.3146
INO2	YFR049W		0.292216	6.19608						6.4883
INO2	YGL077C	0.800635	0.292216	4.07044						5.16329
INO2	YGR052W	0.875393	0.292216	3.61504						4.78265
INO2	YGR117C			4.03035						4.03035
INO2	YGR156W			5.5624						5.5624
INO2	YGR238C	2.77052	0.292216	1.66097						4.72371
INO2	YHL020C	2.24494		1.91081						4.15575
INO2	YHR123W	0.666187	0.292216	5.48471						6.44311
INO2	YIL166C			3.32992			1.00753			4.33745
INO2	YJL140W	2.48805	0.292216	2.45363						5.2339
INO2	YJL148W		0.292216	4.83362	1.27688					6.40272
INO2	YJL153C	3.52151	0.292216	1.68843						5.50216
INO2	YJR073C	1.54675	0.292216	2.98818						4.82715
INO2	YJR074W	1.54675	0.292216	4.26265						6.10162
INO2	YJR075W	1.54675	0.292216	3.13632						4.97529
INO2	YJR104C	4.52454								4.52454
INO2	YJR105W	4.52454								4.52454
INO2	YJR106W	4.52454								4.52454
INO2	YKL099C	0.199392	0.292216	4.95325	0.643302	0.43026				6.51842
INO2	YKL182W	7.46333	0.292216	0.196745		0.822227	0.962992	1.21789		10.9554
INO2	YKL183C-A	7.46333	0.292216							7.75555
INO2	YKR056W			4.78048	0.613817					5.3943
INO2	YLR136C		0.292216	3.71575						4.00797
INO2	YML075C	2.92551	0.292216	0.663127	0.838232					4.71908
INO2	YMR070W	3.28375	0.292216	0.543733						4.1197
INO2	YNL039W			5.57614						5.57614

TF	Target	Binding_0-2000	Motif_Score_SC	Other_Yeasts	Two-Hop_BE	Two-Hop_BP	Two-Hop_BY	Two-Hop_BG	Max_Membership	Sum
INO2	YNL129W	3.33154	0.292216	1.34519						4.96895
INO2	YNL130C	3.33154	0.292216	2.91659						6.54035
INO2	YNL169C	2.70536	0.292216	2.01083						5.00841
INO2	YNL172W	4.10598	0.292216							4.3982
INO2	YNL175C	4.10598	0.292216	0.0452576	0.997735					5.44119
INO2	YNL176C	4.10598	0.292216							4.3982
INO2	YNR016C	5.42013	0.292216	0.938304	0.697461		0.897214			8.24533
INO2	YNR017W	5.42013	0.292216							5.71235
INO2	YOR230W	3.87011	0.292216							4.16233
INO2	YOR234C	3.23855			0.778428					4.01698
INO2	YOR246C			3.276			1.07618			4.35218
INO2	YOR316C	3.7818	0.292216							4.07402
INO2	YOR316C-A	3.7818	0.292216							4.07402
INO2	YOR317W	3.7818	0.292216							4.07402
INO2	YPL014W		0.292216	4.60578						4.898
INO2	YPL231W	3.65596	0.292216	2.01671	0.58762	1.85005	1.13206	1.90397		11.4386
INO2	YPR113W			3.72801			1.02299			4.751
INO4	YBR029C	2.8205	1.15832	12.9808						16.9596
INO4	YBR030W	2.8205	1.15832	13.7474						17.7262
INO4	YBR085C-A	1.54489	0.7161	3.28275						5.54374
INO4	YDL049C	1.76346	1.15832	1.6069						4.52868
INO4	YDR045C		1.15832	9.51722						10.6755
INO4	YDR079W		0.756677	3.56449						4.32117
INO4	YDR146C	2.58466	1.15832	6.71974						10.4627
INO4	YDR147W	2.58466	1.15832	6.67914						10.4221
INO4	YDR174W		1.15832	6.8006						7.95892
INO4	YDR276C			4.50962						4.50962
INO4	YDR497C	2.77226	1.15832	12.93						16.8606
INO4	YDR502C		0.756677	4.76424						5.52092
INO4	YER026C	2.48585	0.699307	3.7034						6.88856
INO4	YER098W			4.74426						4.74426
INO4	YER115C	0.23857		3.85232						4.09089
INO4	YGL077C	1.3728	1.15832	7.8165						10.3476
INO4	YGL100W			4.45122						4.45122
INO4	YGL144C		0.539714	3.63137						4.17108
INO4	YGL243W		1.15832	4.88008						6.0384
INO4	YGR037C	0.160809	0.7161	4.49926						5.37617
INO4	YGR157W	2.71544	0.756677	2.32495						5.79707
INO4	YHL032C		1.15832	11.4728						12.6311
INO4	YHR018C		1.15832	8.21414						9.37246
INO4	YHR067W	1.22236	1.15832	5.35546						7.73614
INO4	YHR170W			5.77754	0.764924					6.54246
INO4	YIL118W	1.2038	1.15832	3.49618						5.8583
INO4	YIL119C	1.2038	1.15832	3.6526						6.01472
INO4	YJL117W	1.55507	0.117897	7.1935						8.86647
INO4	YJL140W	1.92322	1.15832	10.3921						13.4736
INO4	YJL141C	1.92322	1.15832	7.0151						10.0966
INO4	YJL153C	1.42578	1.15832	7.26333						9.84743
INO4	YJL167W	2.02909	1.15832	13.5653						16.7527
INO4	YJL168C	2.02909	1.15832	6.87352						10.0609
INO4	YJR008W	1.14387	1.15832	1.69888						4.00107
INO4	YJR104C	2.38171	1.15832	8.60929						12.1493
INO4	YJR105W	2.38171	1.15832	6.70181						10.2418
INO4	YKL182W	2.57908	1.15832	7.39773		0.60262		1.0713		12.809
INO4	YLR016C			8.33523						8.33523
INO4	YLR017W			7.74822	0.33563					8.08385
INO4	YLR034C	1.73566	0.756677	3.95364						6.44598
INO4	YLR132C	1.24333	0.539714	4.56431						6.34735
INO4	YLR133W	1.24333	0.539714	4.68572						6.46876
INO4	YLR351C	1.7756	1.15832	1.16544						4.09936
INO4	YLR414C		0.579729	4.0595						4.63923
INO4	YMR200W			6.106						6.106
INO4	YNL080C	1.92719	0.103904	2.1422						4.17329
INO4	YNL129W	1.69621	1.15832	10.803						13.6575
INO4	YNL130C	1.69621	1.15832	8.86072						11.7153
INO4	YNL137C		0.457574	5.15349						5.61106
INO4	YNL169C	2.13759	0.457574	2.88845						5.48361
INO4	YNL229C		0.539714	3.70313						4.24284
INO4	YNR016C	2.32247	0.756677	3.66657						6.74572
INO4	YNR017W	2.32247	0.756677	7.58787						10.667
INO4	YOL108C		0.539714	3.83194						4.37165
INO4	YOR114W	0.14968	0.280588	6.85804						7.28831
INO4	YOR212W		0.262452	4.24038						4.50283
INO4	YOR245C		1.15832	6.95599						8.11431
INO4	YOR247W		1.15832	3.17161						4.32993
INO4	YOR316C	2.06478	0.539714	1.91233						4.51682
INO4	YOR317W	2.06478	0.539714	4.93267						7.53716
INO4	YOR355W	0.0542292		5.38525						5.43948
INO4	YPL027W			4.98264						4.98264
INO4	YPL231W	2.84246	1.15832	6.85658		0.531519		1.02384		12.4127
INO4	YPR013C	2.42857	1.15832	12.4562						16.0431
IXR1	YBL087C	5.07236			0.37528					5.44764
IXR1	YBL088C	5.07236								5.07236
KSS1	YBL039C	1.02139			2.45083				0.724513	4.19673
KSS1	YBR247C				3.23654	3.9249			0.618513	7.77995
KSS1	YBR248C				3.22105		0.983786			4.20484
KSS1	YBR267W				2.2467		1.916			4.1627
KSS1	YBR271W				2.80587		1.87141			4.67728
KSS1	YCL037C				2.26843		1.96532			4.23375
KSS1	YCL059C	0.681335			0.587466	3.9249			0.691926	5.88563
KSS1	YCR034W				3.25997		1.87141			5.13138
KSS1	YCR057C	1.12357			2.29203	3.9249				7.3405
KSS1	YDL170W	8.21991								8.21991
KSS1	YDL171C	8.21991								8.21991
KSS1	YDR496C				2.87298		1.87164		0.656184	5.4008
KSS1	YER082C				2.48548		1.89659		0.669659	5.05173
KSS1	YFL021W	5.5095								5.5095
KSS1	YFL022C	5.5095								5.5095
KSS1	YGR128C	0.568159			3.31935				0.674083	4.56159
KSS1	YHR088W				2.61156		1.89541			4.50697
KSS1	YIL079C				2.50543		1.93395			4.43938
KSS1	YKL082C				2.5146		1.88282			4.39742
KSS1	YKR056W				3.39258				0.674045	4.06663
KSS1	YLL011W				2.77975		1.87457			4.65432
KSS1	YLR409C	0.321206			3.42931		1.87457		0.722565	6.34765
KSS1	YML093W	0.531748			2.35498		1.87199			4.75872
KSS1	YMR049C	0.417281			3.17978		1.89659		0.623477	6.11713
KSS1	YMR131C	0.212074			3.14907		1.87798		0.731777	5.9709
KSS1	YNL062C				3.48806				0.723979	4.21204
KSS1	YNL075W				3.41805		1.89541		0.720306	6.03377

TF	Target	Binding_0-2000	Motif_Score_SC	Other_Yeasts	Two-Hop_BE	Two-Hop_BP	Two-Hop_BY	Two-Hop_BG	Max_Membership	Sum
KSS1	YNL110C	1.34732			2.88352		1.88239		0.621884	6.73511
KSS1	YNL175C				2.38449		1.89541			4.2799
KSS1	YNL182C				3.41207				0.699879	4.11195
KSS1	YNL186W				2.41234		1.87457			4.28691
KSS1	YNL248C				3.50372				0.533441	4.03716
KSS1	YOL041C				2.37477		1.92287			4.29764
KSS1	YOL077C				2.9386		1.916		0.729803	5.5844
KSS1	YOR001W	0.743454			3.23358				0.55173	4.52876
KSS1	YPL043W	0.293838			3.06016		1.89541			5.24941
KSS1	YPL263C				2.8445		1.88282			4.72732
KSS1	YPR035W	7.06534								7.06534
KSS1	YPR144C	15.8551								15.8551
KSS1	YPR145W	15.8551								15.8551
LEU3	SNR53	5.23033								5.23033
LEU3	SNR8	5.618	2.56136							8.17936
LEU3	TA(AGC)K1	4.36317								4.36317
LEU3	TR(UCU)E	4.13865								4.13865
LEU3	YAL036C			3.25312	0.769138					4.02226
LEU3	YBL071W-A	1.35485	1.48652	5.3431						8.18447
LEU3	YBL087C	0.31772	0.0918823	3.60825	0.759498					4.77735
LEU3	YBR033W	0.693316	1.40119	4.4009						6.49541
LEU3	YBR068C	5.46462	2.2685	7.58719						15.3203
LEU3	YBR247C	0.0819135	0.861537	2.21158	1.01386					4.16889
LEU3	YCL018W	1.58391	2.2685	5.09522			1.32758			10.2752
LEU3	YDL033C		0.457574	4.07393						4.5315
LEU3	YDL222C		1.29729	3.19878						4.49607
LEU3	YDR153C	0.366406	1.2337	3.99719						5.5973
LEU3	YDR156W	0.366406	1.2337	4.79246						6.39257
LEU3	YDR432W		2.2685	5.22958						7.49808
LEU3	YEL050C	5.23033								5.23033
LEU3	YER071C	3.13106	1.48652							4.61758
LEU3	YER073W	3.13106	1.48652	4.64228	1.15942					10.4193
LEU3	YER074W	0.893569	0.965639	0.629411	0.852333		0.814843			4.15579
LEU3	YFL021W	1.56575	1.29729	2.01546						4.8785
LEU3	YFL022C	1.56575	1.29729	2.02958						4.89262
LEU3	YFL038C		2.2685	6.93831				1.04813		10.2549
LEU3	YGL009C	9.20551	2.17992	6.30527			0.975351			18.6661
LEU3	YGL071W		2.2685	8.69922						10.9677
LEU3	YGL101W	1.59279	1.15	3.22361						5.9664
LEU3	YGL125W	1.18248	1.48652	5.20057	0.944291				0.708114	9.52197
LEU3	YGR159C		2.17992	1.88708						4.067
LEU3	YHR207C	6.79733	2.56136	10.9834						20.3421
LEU3	YHR208W	6.79733	2.56136	14.2798	1.91277					25.5513
LEU3	YJL093C		2.17992	1.86021						4.04013
LEU3	YJR016C	3.68042	1.40119	4.05321						9.13482
LEU3	YKL028W		1.51831	6.19315						7.71146
LEU3	YKL029C		1.51831	6.20405						7.72236
LEU3	YKL117W	4.36317			0.412582					4.77575
LEU3	YKL120W	11.1831	1.16441	2.31317	1.01686					15.6775
LEU3	YKR071C		2.56136	14.6384	2.02941					19.2292
LEU3	YLL011W		0.612149	0.489531	1.69637		1.33006			4.12811
LEU3	YLL054C		0.887742	4.74192						5.62966
LEU3	YLR355C	2.60965	2.56136	12.083	0.643234		1.13142		0.721263	19.7499
LEU3	YLR356W	2.60965	2.56136	11.0083						16.1793
LEU3	YLR357W	2.60965	2.56136					0.884106		6.05512
LEU3	YMR003W		1.62185	2.79473						4.41658
LEU3	YMR014W	2.19647			1.82177					4.01824
LEU3	YMR108W	6.33136	2.56136	15.5011			1.3128		0.615063	26.3217
LEU3	YMR290C	1.42564	1.17159	2.86733	1.16813					6.63269
LEU3	YMR291W	1.42564	1.17159	2.18221						4.77944
LEU3	YNL065W	1.60386	1.21673	1.76098						4.58157
LEU3	YNL103W	5.48155	1.29729	4.32873						11.1076
LEU3	YNL104C	5.48155	1.29729	4.33965	0.460055		0.797581			12.3761
LEU3	YOL011W		1.40119	4.28494						5.68613
LEU3	YOR008C		1.17159	2.9834						4.15499
LEU3	YOR108W	2.68492	1.51831	6.30182						10.5051
LEU3	YOR224C	2.10791	0.794603	0.300868	1.02152					4.2249
LEU3	YOR270C	5.618	2.56136		0.432182					8.61154
LEU3	YOR271C	5.618	2.56136	12.9671						21.1465
LEU3	YOR272W	5.618	2.56136	7.22599	1.73063					17.136
LEU3	YOR375C	2.2345	2.2685	10.7473						15.2503
LEU3	YOR376W-A	2.2345	2.2685							4.503
LEU3	YPL253C	0.540867	0.490698	3.04349						4.07505
LEU3	YPR138C		0.480094	5.0077						5.48779
MAC1	YAR002C-A	0.954323			2.23988		1.28017			4.47437
MAC1	YBL103C			4.03616						4.03616
MAC1	YBR036C	4.16942								4.16942
MAC1	YBR037C	4.16942	0.408471							4.57789
MAC1	YBR038W	4.16942								4.16942
MAC1	YBR088C			1.09218	2.71751		1.05736			4.86705
MAC1	YBR267W		0.408471	3.92986	1.15641					5.49474
MAC1	YDL081C		0.408471	1.72183	2.32096					4.45126
MAC1	YDL175C		0.408471	2.38058			1.27978			4.06883
MAC1	YDL197C	0.596068	0.408471	4.75262						5.75716
MAC1	YDL201W		0.408471	1.97087	2.47273		1.04998			5.90205
MAC1	YDR058C	2.73028	0.408471	2.68811						5.82686
MAC1	YER145C	6.94249	0.408471	2.49232						9.84328
MAC1	YER146W	6.94249	0.408471	1.9976	2.24456					11.5931
MAC1	YER155C		0.408471	4.47891						4.88738
MAC1	YFR044C	4.50397								4.50397
MAC1	YGL135W	2.20223			2.6372		0.960882			5.80031
MAC1	YGL137W			1.77975		3.33393				5.11368
MAC1	YGL180W			4.34166						4.34166
MAC1	YGR255C	3.09796		2.57789						5.67585
MAC1	YGR256W	3.09796		2.71761						5.81557
MAC1	YHR191C	3.50044		0.530527						4.03097
MAC1	YIL103W		0.408471	5.2481			0.994772			6.65134
MAC1	YIL116W	3.98053	0.408471							4.389
MAC1	YIL117C	3.98053	0.408471							4.389
MAC1	YJR049C	2.1272	0.408471	4.00556						6.54123
MAC1	YKR044W		0.408471	5.12254						5.53101
MAC1	YLR012C			4.33572						4.33572
MAC1	YLR085C		0.408471	5.18824						5.59671
MAC1	YLR110C	1.43468	0.408471	2.50544						4.34859
MAC1	YLR167W	0.132497	0.408471	2.03898	2.29081					4.87076
MAC1	YLR213C	15.8551	0.408471	0.867632						17.1312
MAC1	YLR214W	15.8551	0.408471	0.49124	2.95533					19.7101
MAC1	YLR243W			1.08442	2.36872		0.96268			4.41582
MAC1	YMR047C				2.81797		1.28538			4.10335
MAC1	YMR090W		0.408471	3.32425	0.680637					4.41336

TF	Target	Binding_0-2000	Motif_Score_SC	Other_Yeasts	Two-Hop_BE	Two-Hop_BP	Two-Hop_BY	Two-Hop_BG	Max_Membership	Sum
MAC1	YMR319C	6.2413	0.408471	0.266252	2.2691					9.18512
MAC1	YNL016W	3.19249				0.333949	1.26349			4.78993
MAC1	YOL012C			1.93953	2.57093					4.51046
MAC1	YOL152W	4.51165	0.408471		2.41484					7.33496
MAC1	YOR039W		0.408471	5.25102						5.65949
MAC1	YOR043W		0.408471	5.89395						6.30242
MAC1	YPL177C	9.49408	0.408471	0.925616						10.8282
MAC1	YPR102C	0.533505		0.0905091	2.55724		0.912925			4.09418
MAC1	YPR120C	1.85544		0.78993	2.50728					5.15265
MAC1	YPR124W	15.8551	0.408471	2.13074	2.95533					21.3496
MAC1	YPR125W	5.32124	0.408471							5.72971
MAL33	YCR105W	10.193								10.193
MAL33	YCR107W	12.294			0.393715					12.6877
MAL33	YFR019W	3.13638					1.13113			4.26751
MAL33	YJL068C	5.86181								5.86181
MAL33	YJL069C	5.86181								5.86181
MAL33	YJR143C	8.12872								8.12872
MAL33	YLR397C	5.80209								5.80209
MAL33	YLR438C-A	7.43293								7.43293
MAL33	YLR439W	7.43293								7.43293
MAL33	YNL241C	7.83781								7.83781
MAL63	YBL010C			5.34884						5.34884
MAL63	YBR053C			6.52601						6.52601
MAL63	YBR101C			4.0878						4.0878
MAL63	YBR276C			4.69224						4.69224
MAL63	YBR279W			4.69236						4.69236
MAL63	YBR299W			8.596						8.596
MAL63	YDL204W			6.08517						6.08517
MAL63	YDL205C			4.86064						4.86064
MAL63	YDR207C			5.41771						5.41771
MAL63	YDR208W			4.57721						4.57721
MAL63	YDR463W			4.28347						4.28347
MAL63	YDR493W			5.34205						5.34205
MAL63	YEL009C			6.15548						6.15548
MAL63	YGL008C			5.68191						5.68191
MAL63	YGL047W			4.74061						4.74061
MAL63	YGL048C			4.73939						4.73939
MAL63	YGR141W			6.08919						6.08919
MAL63	YGR206W			4.74084						4.74084
MAL63	YHR011W			4.23105						4.23105
MAL63	YHR091C			5.33576						5.33576
MAL63	YHR114W			5.35559						5.35559
MAL63	YIL031W			5.72743						5.72743
MAL63	YIL033C			5.15833						5.15833
MAL63	YJR070C			5.07121						5.07121
MAL63	YKL088W			4.97135						4.97135
MAL63	YKL117W			4.38682						4.38682
MAL63	YKR006C			7.45548						7.45548
MAL63	YKR008W			7.56039						7.56039
MAL63	YKR100C			6.06723						6.06723
MAL63	YKR101W			4.87054						4.87054
MAL63	YLL026W			4.56477						4.56477
MAL63	YLR097C			4.48302						4.48302
MAL63	YLR131C			4.64596						4.64596
MAL63	YLR134W			9.88756						9.88756
MAL63	YLR193C			4.33503						4.33503
MAL63	YLR237W			4.31322						4.31322
MAL63	YLR396C			4.29544						4.29544
MAL63	YLR423C			5.48244						5.48244
MAL63	YML037C			4.18175						4.18175
MAL63	YNL024C			4.54572						4.54572
MAL63	YNL234W			4.73883						4.73883
MAL63	YNL289W			4.14689						4.14689
MAL63	YNL291C			6.60736						6.60736
MAL63	YNL298W			4.01923						4.01923
MAL63	YNL299W			4.10984						4.10984
MAL63	YOL005C			4.42256						4.42256
MAL63	YOR145C			11.7045						11.7045
MAL63	YPL098C			9.58307						9.58307
MATA1	YAL048C		0.86037	7.66129						8.52166
MATA1	YBR199W		0.86037	17.3573						18.2177
MATA1	YCL021W-A			4.01918						4.01918
MATA1	YCR081W			6.50367						6.50367
MATA1	YDL130W-A			4.81149						4.81149
MATA1	YDL217C			6.98171						6.98171
MATA1	YDL227C		0.764995	11.4846						12.2496
MATA1	YDR282C			4.11838						4.11838
MATA1	YDR402C	0.0643989		4.16574						4.23014
MATA1	YER105C			6.8471						6.8471
MATA1	YER106W			6.34671						6.34671
MATA1	YGR112W		0.497548	4.8544						5.35195
MATA1	YGR263C			4.23648						4.23648
MATA1	YHR127W			4.31999						4.31999
MATA1	YIL108W			4.01894						4.01894
MATA1	YIL166C			4.21285						4.21285
MATA1	YIR001C			6.64505						6.64505
MATA1	YKL005C			4.63962						4.63962
MATA1	YKL054C		0.661944	4.82945						5.49139
MATA1	YKL190W		0.534152	3.50844						4.04259
MATA1	YLR438W			4.81568						4.81568
MATA1	YNL314W			6.54487						6.54487
MATA1	YNL316C			5.48244						5.48244
MATA1	YOL110W			4.82071						4.82071
MATA1	YOL153C			4.54425						4.54425
MATA1	YOR051C		0.86037	4.74219						5.60256
MATA1	YOR350C			6.16938						6.16938
MATA1	YPL033C			4.09708						4.09708
MATA1	YPL269W		0.86037	7.28085						8.14122
MATA2	YAL056W		0.939217	4.77204						5.71126
MATA2	YAR018C			4.13419						4.13419
MATA2	YBL014C		0.939217	6.35464						7.29386
MATA2	YBL020W		0.409736	5.27187						5.68161
MATA2	YCR009C		0.939217	5.6261						6.56532
MATA2	YDL144C			4.89238						4.89238
MATA2	YDL161W		0.939217	9.41979						10.359
MATA2	YDL235C			8.53872						8.53872
MATA2	YDR297W		0.626768	4.02981						4.65658
MATA2	YDR451C		0.939217	3.99026						4.92948
MATA2	YER022W		0.939217	6.28161						7.22083
MATA2	YER049W		0.939217	8.21867						9.15789

TF	Target	Binding_0-2000	Motif_Score_SC	Other_Yeasts	Two-Hop_BE	Two-Hop_BP	Two-Hop_BY	Two-Hop_BG	Max_Membership	Sum
MATA2	YFL026W		0.626768	6.86526						7.49203
MATA2	YFL027C		0.626768	7.64667						8.27344
MATA2	YGL005C		0.939217	4.447						5.38622
MATA2	YGL032C		0.939217	3.71776						4.65698
MATA2	YGL067W		0.626768	3.96653						4.5933
MATA2	YGL130W			4.07542						4.07542
MATA2	YGL157W			4.80967						4.80967
MATA2	YGL237C		0.626768	4.41656						5.04333
MATA2	YGR075C		0.626768	3.84684						4.47361
MATA2	YGR112W			8.58409						8.58409
MATA2	YGR116W			4.52223						4.52223
MATA2	YHR038W		0.626768	3.3802						4.00697
MATA2	YIL015W		0.939217	5.53835						6.47757
MATA2	YIR022W		0.626768	3.55784						4.18461
MATA2	YIR032C			4.20464						4.20464
MATA2	YJR100C		0.626768	3.69168						4.31845
MATA2	YJR152W			5.73265						5.73265
MATA2	YKL208W		0.939217	10.3209						11.2601
MATA2	YKL209C		0.939217	7.7733						8.71252
MATA2	YKR066C			4.97453						4.97453
MATA2	YLR218C		0.939217	3.76218						4.7014
MATA2	YLR222C		0.939217	3.47906						4.41828
MATA2	YLR397C		0.939217	4.73444						5.67366
MATA2	YML001W			9.40531						9.40531
MATA2	YML032C		0.939217	10.7011						11.6403
MATA2	YMR157C		0.626768	3.87896						4.50573
MATA2	YMR201C		0.939217	3.54072						4.47994
MATA2	YMR203W		0.939217	3.93794						4.87716
MATA2	YNR046W		0.626768	4.28446						4.91123
MATA2	YOL032W		0.626768	4.67993						5.3067
MATA2	YOR004W			4.05532						4.05532
MATA2	YPL266W			4.19222						4.19222
MATA2	YPR057W		0.626768	6.40055						7.02732
MBP1	SNR37	4.07143								4.07143
MBP1	SNR40	6.82139								6.82139
MBP1	TH(GUG)K	4.16643								4.16643
MBP1	TK(CUU)C	7.52573								7.52573
MBP1	TM(CAU)J3	4.45761								4.45761
MBP1	TR(UCU)E	7.50636								7.50636
MBP1	YAL062W	3.18631			0.61533				0.66603	4.46767
MBP1	YAR003W	0.733792		2.03898	0.546232		1.10984		0.72319	5.15203
MBP1	YAR007C	2.53811	0.386934	3.63741	1.94749	2.25946	1.07137			11.8408
MBP1	YAR008W	2.53811	0.386934	2.54885		0.737124			0.522581	6.7336
MBP1	YBL002W	3.69803	0.722683		0.894581	0.671538			0.679472	6.6663
MBP1	YBL003C	3.69803	0.722683	0.426443	0.894581				0.716941	6.45868
MBP1	YBL023C	3.68648				2.25768	1.78517			7.72933
MBP1	YBL035C	2.28441	0.46196	1.76665	1.22775	1.24902				6.98979
MBP1	YBR007C	2.50102	0.386934	3.61415					0.718511	7.22062
MBP1	YBR009C	0.281465			3.0816		0.971821			4.33489
MBP1	YBR010W	0.281465			1.61646	0.778547	1.54711		0.71475	4.93833
MBP1	YBR034C	0.0785365			2.03059		1.48206		0.674447	4.26563
MBP1	YBR048W				1.7931		1.80885		0.724578	4.32653
MBP1	YBR049C	1.23872		1.33131	0.700638	0.671538	1.21865			5.16086
MBP1	YBR069C	5.53234							0.715545	6.24788
MBP1	YBR070C	5.53234			1.96338				0.671745	8.16746
MBP1	YBR071W	5.53234			1.95906				0.707254	8.19865
MBP1	YBR073W	1.4976			2.21902			1.00136		4.71798
MBP1	YBR077C	5.92099		0.492513						6.4135
MBP1	YBR078W	5.92099								5.92099
MBP1	YBR081C	2.45695	1.8421	0.792374				1.24407		6.33549
MBP1	YBR085C-A	0.0952695		4.41568						4.51095
MBP1	YBR086C	1.12701		2.96491					0.497902	4.58982
MBP1	YBR087W	1.12701				1.41299	1.72132			4.26132
MBP1	YBR088C	1.05778	0.230573	0.601325	2.24216	1.55724	1.80567		0.729644	8.22439
MBP1	YBR098W	1.42597	1.15555	1.52917						4.11069
MBP1	YBR114W	1.08898			0.433651	2.33875		1.00142		4.8628
MBP1	YBR126C				1.89186	1.47619			0.730163	4.09821
MBP1	YBR189W			0.66839	1.41158		1.80177		0.661198	4.54294
MBP1	YBR191W	2.02484			1.94898		1.73051		0.711752	6.41608
MBP1	YBR204C			4.43696	1.37718					5.81414
MBP1	YBR267W				1.8658		1.54711		0.653444	4.06635
MBP1	YBR275C	2.35022		2.45657						4.80679
MBP1	YCL024W	1.87712	0.230573	0.155174	1.25383				0.678093	4.19479
MBP1	YCL059C				3.08402		1.72444		0.460093	5.26855
MBP1	YCL061C		0.782778	3.17898	0.824267				0.657264	5.44329
MBP1	YCR019W	7.52573								7.52573
MBP1	YCR020C	5.22678								5.22678
MBP1	YCR020C-A	5.22678								5.22678
MBP1	YCR065W	13.3331	0.386934	1.63892	1.9453				0.560804	17.8651
MBP1	YDL003W	5.77024	0.386934	5.31625	2.53239	0.627868			0.725901	15.3596
MBP1	YDL008W	1.01614	0.386934	1.43834			1.19619			4.0376
MBP1	YDL010W	1.01614	0.386934	0.250098	1.47199		1.18851		0.709014	5.02269
MBP1	YDL017W	8.97342	0.386934	1.24451		0.830467				11.4353
MBP1	YDL018C	8.97342	0.386934	2.39046	1.59127		1.54711			14.8892
MBP1	YDL055C	1.39486			3.08494		0.837388		0.640668	5.95786
MBP1	YDL061C				1.83826		1.70288		0.732364	4.2735
MBP1	YDL083C				1.93894		1.78618		0.664754	4.38987
MBP1	YDL099W	3.90757	1.15555				0.994607			6.05773
MBP1	YDL101C	3.90757	1.15555	1.53007	0.916355				0.665038	8.17458
MBP1	YDL106C	1.89255	0.892969	0.1064			1.22545			4.11737
MBP1	YDL127W	1.08341	1.22768		1.69384					4.00493
MBP1	YDL156W	1.80594		2.64925	2.17361		1.54711		0.610166	8.78608
MBP1	YDL157C	1.80594		2.2566					0.556462	4.619
MBP1	YDL164C	0.253792		0.422567	2.36963		1.26637		0.704649	5.01701
MBP1	YDL195W	1.6156	0.680054	0.94759		0.472727			0.485554	4.20152
MBP1	YDL197C	1.6156	0.680054	1.72557	1.95851		1.00332	1.15042	0.610551	8.74403
MBP1	YDL225W			3.32352	0.974885	0.501849				4.80025
MBP1	YDL226C			0.831364		2.00146		1.53269		4.36551
MBP1	YDR025W				1.80165		1.80885		0.698837	4.30934
MBP1	YDR041W	2.88562					1.77845			4.66407
MBP1	YDR052C	0.413992	0.722683	2.25631		2.25768	0.833385		0.520603	7.00465
MBP1	YDR058C	0.120795	1.8421	3.1829					0.469181	5.61498
MBP1	YDR064W				1.88119		1.78563		0.732202	4.39902
MBP1	YDR097C	2.11517	1.05039	0.568087	2.2207	0.746728			0.687202	7.38828
MBP1	YDR113C	3.3774	0.386934	1.8235						5.58783
MBP1	YDR115W	3.3774	0.386934	3.26694			1.79555			8.82682
MBP1	YDR120C		1.01016	3.71787	0.813489		1.72387		0.724707	7.9901
MBP1	YDR144C	2.22097		0.111682			1.18851		0.633026	4.15419
MBP1	YDR192C	2.85642					1.21865			4.07507
MBP1	YDR211W	2.07722			1.67951		1.27015		0.677803	5.70468

TF	Target	Binding_0-2000	Motif_Score_SC	Other_Yeasts	Two-Hop_BE	Two-Hop_BP	Two-Hop_BY	Two-Hop_BG	Max_Membership	Sum
MBP1	YDR238C			2.82137		0.942156	0.88741		0.690597	5.34153
MBP1	YDR263C	13.222	0.386934	2.17463			1.38232		0.727391	17.8933
MBP1	YDR264C	8.02424				1.16246	0.999903	1.51897		11.7056
MBP1	YDR279W	1.9718		1.27644	0.939323				0.551748	4.73931
MBP1	YDR295C	2.03986	0.230573				1.0374	0.894005	0.718883	4.92072
MBP1	YDR297W	2.03986	0.230573	3.18617					0.587621	6.04422
MBP1	YDR309C	1.98006	0.386934	1.76026						4.12725
MBP1	YDR373W				1.4937		1.10385	1.75847		4.35602
MBP1	YDR418W				1.95642		1.7965		0.711614	4.46453
MBP1	YDR450W				1.56527		1.76134		0.714811	4.04142
MBP1	YDR451C	5.20267			1.6039		1.54711			8.35368
MBP1	YDR452W	5.20267								5.20267
MBP1	YDR500C	8.05678			0.731783		1.30577		0.632902	10.7272
MBP1	YDR501W	8.05678			1.98633				0.732428	10.7755
MBP1	YDR503C	0.849622		3.11229	1.29343		1.08326			6.3386
MBP1	YDR507C	2.46917	1.28647	4.63302	2.11813				0.476489	10.9833
MBP1	YDR524C	7.91394						1.53885		9.45279
MBP1	YDR524C-B	7.91394								7.91394
MBP1	YDR525W-A	7.91394		0.0936571						8.0076
MBP1	YDR527W	7.91394		0.254105					0.601383	8.76943
MBP1	YEL047C	0.454945	2.06497	1.29882					0.727168	4.5459
MBP1	YER001W	2.09389			1.97419					4.06808
MBP1	YER012W				1.74196	2.44997	1.77125			5.96318
MBP1	YER016W		0.286405	3.35894					0.472508	4.11785
MBP1	YER019W			5.15107						5.15107
MBP1	YER029C			1.10795	1.16932	2.16065	0.865703		0.703549	6.00717
MBP1	YER070W	13.0176	0.386934	3.53459	2.00977				0.729022	19.6779
MBP1	YER073W	3.6859							0.701976	4.38788
MBP1	YER078C	10.5764								10.5764
MBP1	YER079W	10.5764			0.999555					11.576
MBP1	YER080W	10.5764								10.5764
MBP1	YER087C-B	4.54826								4.54826
MBP1	YER093C-A	9.68569	0.834037							10.5197
MBP1	YER094C	9.68569	0.834037	0.281293			1.77961			12.5806
MBP1	YER095W	9.68569	0.834037	0.873838	1.58582	0.329926	1.79751		0.643734	15.7506
MBP1	YER098W	0.691533			2.24363		1.09554	1.52926		5.55996
MBP1	YER102W				1.54774		1.7604		0.713993	4.02213
MBP1	YER111C	6.99517	0.46196	3.69515		0.32055				11.4728
MBP1	YER112W	6.99517	0.46196	3.95289		2.16065	1.31334			14.884
MBP1	YER118C	2.42334		1.6685	1.93176				0.723639	6.74724
MBP1	YER146W					2.16065	1.78497		0.564274	4.50989
MBP1	YER148W				3.08426	0.86207	1.71948			5.66581
MBP1	YER154W	0.39819		4.50666	0.769993		1.36481			7.03965
MBP1	YER171W		0.386934	2.79793		0.524136	1.29696			5.00596
MBP1	YFL008W	2.30049		0.201307		1.39299			0.467832	4.36262
MBP1	YFL037W	1.44098	1.15555	2.33533					0.694369	5.62623
MBP1	YFL039C	1.44098	1.15555	0.132622	0.511871	1.16246	0.970097			5.37358
MBP1	YFR017C	5.9013								5.9013
MBP1	YFR019W	5.9013					1.80913			7.71043
MBP1	YFR026C	1.86211		1.2902	1.39235				0.59781	5.14247
MBP1	YFR027W	1.86211		1.2124	0.753224	3.2118				7.03953
MBP1	YFR053C		0.515569	1.96123	1.54861					4.02541
MBP1	YGL001C	5.75398								5.75398
MBP1	YGL011C	0.101055			1.93733	2.25946	1.8042			6.10204
MBP1	YGL030W				1.97683		1.68065		0.713324	4.3708
MBP1	YGL037C	0.374662		0.942396	2.23981				0.731434	4.2883
MBP1	YGL076C				1.92936		1.78433		0.727793	4.44148
MBP1	YGL135W				1.92869		1.75474		0.715457	4.39889
MBP1	YGL145W	1.88504	0.386934			2.18128				4.45325
MBP1	YGL147C	1.88504	0.386934		1.44282		1.80144		0.653996	6.17023
MBP1	YGL163C	0.480497	1.03003	0.221195		2.44997		1.00142	0.450902	5.63401
MBP1	YGL225W		0.204416	3.20457			1.19619			4.60518
MBP1	YGR020C			4.91102					0.469639	5.38066
MBP1	YGR034W				1.91529		1.79068		0.732578	4.43855
MBP1	YGR060W	0.434755		3.39132		1.38859				5.21467
MBP1	YGR080W	2.41553			1.92177					4.3373
MBP1	YGR085C	1.00439	0.386934	0.431914	1.36937		1.68446		0.699682	5.57675
MBP1	YGR094W			5.44011					0.722468	6.16258
MBP1	YGR109C	12.5047		1.62814	2.0764		1.13251		0.715524	18.0573
MBP1	YGR110W	6.33217	0.46196				1.09554			7.88967
MBP1	YGR138C	1.78417	0.386934	2.84726						5.01836
MBP1	YGR152C	1.50963			1.36174		1.08231	1.98473	0.661664	6.60007
MBP1	YGR165W	2.14011	1.8421	3.35021						7.33242
MBP1	YGR180C	2.61532	0.386934	4.41273	0.912408	0.588015			0.718206	9.63361
MBP1	YGR189C	3.38065	0.386934	5.43528	0.670868					9.87373
MBP1	YGR214W				3.0821		1.78657		0.727092	5.59576
MBP1	YGR221C	1.99964			2.1168				0.681171	4.79761
MBP1	YGR234W	0.822743			1.43809	2.25946				4.52029
MBP1	YGR248W			3.57994	1.91339					5.49333
MBP1	YHL008C			2.16733	2.01296					4.18029
MBP1	YHL033C		0.386934	0.469973	1.48921		1.79493		0.510095	4.65114
MBP1	YHL034C		2.06497	4.34543						6.4104
MBP1	YHR020W				1.08255	1.13823	1.13993		0.725464	4.08617
MBP1	YHR033W			5.40932					0.657419	6.06674
MBP1	YHR110W	2.54495	0.386934	2.21917	1.11592		1.16267		0.623362	8.05301
MBP1	YHR111W	2.54495	0.386934	1.79771						4.72959
MBP1	YHR143W	3.43178			0.793888					4.22567
MBP1	YHR149C	7.5618		1.81299	0.936034				0.654063	10.9649
MBP1	YHR150W	7.5618	0.386934	0.430965						8.3797
MBP1	YHR153C	2.68736		2.89352					0.626651	6.20753
MBP1	YHR154W	2.68736		1.14038			1.19619		0.483404	5.50733
MBP1	YHR169W				1.82422	2.56791			0.694757	5.08689
MBP1	YHR179W	1.68961			1.7077				0.632993	4.0303
MBP1	YHR189W		1.09985	1.16972			1.77445			4.04402
MBP1	YHR191C	5.84434				0.524079				6.36842
MBP1	YHR193C	5.84434					1.68423		0.548553	8.07712
MBP1	YHR194W	5.84434								5.84434
MBP1	YHR196W	2.71084			1.51058				0.454862	4.67628
MBP1	YHR204W		0.386934	4.74201					0.529091	5.65803
MBP1	YIL026C	2.93599	0.745368	2.88641	1.52743	1.39299				9.48819
MBP1	YIL066C	0.556109	1.05039	1.49953	1.65454	3.34942			0.636831	8.74682
MBP1	YIL140W	2.55173	0.386934	3.09427	2.23359				0.721283	8.98781
MBP1	YIL143C	2.55173	0.386934	0.944713			1.18186			5.06524
MBP1	YIL159W	1.43891	0.386934	3.58885						5.41469
MBP1	YIL160C	1.43891	0.386934	4.37134	0.795712					6.9929
MBP1	YIR012W			2.07063	2.01952		1.07384		0.729823	5.89381
MBP1	YJL001W	0.825801			1.11086		1.79868		0.731974	4.46732
MBP1	YJL002C	2.24324	0.386934				1.09554		0.593562	4.31928
MBP1	YJL039C	0.626647	1.8421	4.66314						7.13189
MBP1	YJL045W	4.32123	1.8421	3.20812	0.573185					9.94463

TF	Target	Binding_0-2000	Motif_Score_SC	Other_Yeasts	Two-Hop_BE	Two-Hop_BP	Two-Hop_BY	Two-Hop_BG	Max_Membership	Sum
MBP1	YJL073W	2.75112	1.15555	2.19792	1.29295					7.39754
MBP1	YJL074C	2.75112	1.15555	0.578865	1.56393	1.39299			0.722861	8.16532
MBP1	YJL076W	1.36805	0.386934	2.30973		0.324783			0.722416	5.11191
MBP1	YJL080C	2.3551					1.09059		0.728858	4.17455
MBP1	YJL115W	3.49415		0.100666	1.71471	0.489553				5.79908
MBP1	YJL122W		0.386934	1.68651	1.58133				0.712265	4.36704
MBP1	YJL124C		0.386934		1.38409	2.16065	1.05538			4.98705
MBP1	YJL148W	3.17748			1.73848				0.7273	5.64326
MBP1	YJL186W	5.9934	0.680054							6.67345
MBP1	YJL187C	5.9934	0.680054		2.0278		1.04392	1.55408		11.2993
MBP1	YJL191W				2.50279		1.80998		0.7041	5.01687
MBP1	YJL194W	2.85287	1.71299	2.16423			1.79705			8.52714
MBP1	YJL196C	2.85287	1.71299	1.79255						6.35841
MBP1	YJR001W	2.24324	0.386934	1.65404			1.19619			5.4804
MBP1	YJR006W		0.386934	1.83063		0.39476	0.953278		0.67584	4.24144
MBP1	YJR007W		0.386934		0.882965	0.396039	1.77601		0.659009	4.10096
MBP1	YJR011C	3.44004	0.562527							4.00257
MBP1	YJR017C		0.204416	4.50125						4.70567
MBP1	YJR030C	4.44164	1.15555	1.96949	1.82115				0.483093	9.87092
MBP1	YJR044C	4.45761								4.45761
MBP1	YJR047C	0.327231	1.57675		1.09556		1.79853			4.79807
MBP1	YJR055W		1.58446	3.27786						4.86232
MBP1	YJR063W				2.15748		1.812		0.715953	4.68543
MBP1	YJR094W-A				1.85894		1.80041		0.695293	4.35464
MBP1	YJR145C	0.254732	0.386934	0.292173	1.66688		1.75855		0.697397	5.05667
MBP1	YKL007W	2.30957	0.457574		1.60321	0.639776				5.01013
MBP1	YKL009W				1.82225		1.77666		0.717977	4.31689
MBP1	YKL039W		1.09985	1.23391		1.8134	1.11329			5.26045
MBP1	YKL045W	1.69951	0.834037	3.49668	2.01988					8.05011
MBP1	YKL046C	1.69951	0.834037	6.73161						9.26516
MBP1	YKL067W	4.16643							0.617231	4.78366
MBP1	YKL096W-A	4.44773								4.44773
MBP1	YKL101W	2.38504		1.43957					0.590437	4.41505
MBP1	YKL103C	2.38504		0.97655	1.48647				0.71049	5.55855
MBP1	YKL112W	9.27366	0.834037	2.32482						12.4325
MBP1	YKL113C	9.27366	1.8421	4.34567	2.50598		1.38722		0.721773	20.0764
MBP1	YKL141W	2.71628			0.618538		1.10218			4.437
MBP1	YKL142W	2.71628			2.04465	0.423031				5.18396
MBP1	YKL150W	2.02164			1.38464		0.955765			4.36205
MBP1	YKL179C	1.63229					1.61814	2.43101		5.68144
MBP1	YKL180W				1.71766		1.71853		0.6534	4.08959
MBP1	YKR077W	2.74474	0.230573	3.3176	2.17577				0.634381	9.10306
MBP1	YLL008W	1.63782			1.71877				0.667549	4.02414
MBP1	YLL021W	0.73741	0.524868	4.53183	0.590583				0.609788	6.99448
MBP1	YLL022C	0.73741	0.524868	6.16331	1.8181					9.24369
MBP1	YLL026W	1.91022			2.00297		1.71835			5.63154
MBP1	YLL034C	1.54476			2.06019				0.703792	4.30874
MBP1	YLR032W		0.386934	2.17474				1.00141	0.671261	4.23435
MBP1	YLR048W	3.1346			1.73725		1.77593		0.715645	7.36343
MBP1	YLR049C	1.27862	0.524868	3.385	1.76643				0.478182	7.4331
MBP1	YLR055C	1.64186	0.515569	3.88373						6.04116
MBP1	YLR056W	1.64186	0.515569	6.874	0.703627				0.643118	10.3782
MBP1	YLR060W			1.91233	0.760816		1.34221		0.719857	4.73521
MBP1	YLR103C	5.3818	0.386934	2.48002	2.39525	0.830467				11.4745
MBP1	YLR104W	5.3818	0.386934	0.964303						6.73304
MBP1	YLR120C	1.85888	0.386934	0.17463	1.80952		1.19619			5.42615
MBP1	YLR153C	0.670205		2.49533	1.23735					4.40289
MBP1	YLR185W	1.1321			3.08522		1.30577		0.707952	6.23104
MBP1	YLR186W	1.1321			0.723401		1.68577		0.715518	4.25679
MBP1	YLR196W		0.286405		1.97543		1.4862		0.67719	4.42522
MBP1	YLR212C	1.99005		0.509696	2.05356				0.599543	5.15285
MBP1	YLR229C	0.66881				1.16246	1.00812	1.4533		4.29269
MBP1	YLR262C				1.6827		0.986369	1.81166		4.48073
MBP1	YLR264W				1.91186	1.27937	1.69692		0.687443	5.57559
MBP1	YLR274W	0.980017			0.706662	0.830467	1.7462			4.26335
MBP1	YLR295C	2.47565		2.24576						4.72141
MBP1	YLR297W	2.47565		2.30092						4.77657
MBP1	YLR300W	3.59402	0.260505	3.30308					0.690512	7.84812
MBP1	YLR313C	0.549842	0.386934	4.01792	2.30053				0.634176	7.8894
MBP1	YLR314C			1.40905		0.501849	1.18535	1.18674		4.28299
MBP1	YLR344W	0.182504			1.75008		1.79068		0.730782	4.45405
MBP1	YLR367W	3.32277			0.839739		1.81135		0.64435	6.61821
MBP1	YLR382C	1.72161	1.15555	1.39011						4.26727
MBP1	YLR383W	1.72161	1.15555	0.488321	2.02168					5.38716
MBP1	YLR388W				1.94782		1.70288		0.684527	4.33523
MBP1	YLR438C-A	8.61398				1.72367	1.80711			12.1448
MBP1	YLR439W	8.61398								8.61398
MBP1	YLR457C	0.668435	0.46196	1.96778	3.00385					6.10203
MBP1	YLR459W	0.668435	0.46196	3.17139						4.30179
MBP1	YML024W				1.88081		1.7727		0.729339	4.38285
MBP1	YML026C				1.82595		1.76134		0.730894	4.31818
MBP1	YML027W	5.60112	0.393911	0.160999	0.647388		1.19463		0.730894	8.72894
MBP1	YML063W			0.127673	1.8126		1.80253		0.730694	4.4735
MBP1	YML069W	0.591868		2.23209		1.24902	1.22545			5.29843
MBP1	YML071C		1.8421	3.28695						5.12905
MBP1	YML092C					2.25946	1.8042			4.06366
MBP1	YML100W	5.28437	0.00429395	1.96526	1.64633				0.71945	9.6197
MBP1	YML101C	5.28437	0.0925508							5.37692
MBP1	YML109W	1.2708	1.30593	3.43999						6.01672
MBP1	YML110C	1.2708	1.30593	3.22601						5.80274
MBP1	YML114C	0.404057	0.881348	3.04228						4.32768
MBP1	YMR006C	2.03223	1.35323	2.22125	1.06635				0.617289	7.29035
MBP1	YMR012W				0.366481	2.25946	1.18851		0.713345	4.5278
MBP1	YMR017W	1.36146		1.81305			1.20767			4.38218
MBP1	YMR041C	1.60712	0.500037		1.09492		1.08407			4.28615
MBP1	YMR047C	0.335547	0.386934	2.98444		0.35096	1.10466		0.538214	5.70076
MBP1	YMR048W	0.335547	0.386934	2.44186	0.96541	0.914177				5.04393
MBP1	YMR049C		0.0925508		1.92261	0.631485	1.49828		0.689869	4.83479
MBP1	YMR076C	12.5078	0.834037	0.425705		0.33797	0.827892			14.9334
MBP1	YMR078C	2.55152			2.33049	1.41299	1.80255			8.09755
MBP1	YMR079W	2.55152		0.832804	0.577303				0.454484	4.41611
MBP1	YMR119W	0.380361			3.08318		1.2012			4.66474
MBP1	YMR121C	1.40204	0.577888	1.25881	0.642749		1.78563		0.729028	6.39615
MBP1	YMR135C	1.62325	0.834037	1.05613	0.564494					4.07791
MBP1	YMR144W	7.53008								7.53008
MBP1	YMR165C	3.82303							0.656519	4.47955
MBP1	YMR179W	10.2957	1.15555	4.49006	2.52764					18.4689
MBP1	YMR194W	2.5839			1.24603				0.731663	4.56159
MBP1	YMR199W	5.25956	0.386934	0.0658471	1.12555				0.574583	7.41247
MBP1	YMR215W	3.16627	0.605435						0.607875	4.37958

TF	Target	Binding_0-2000	Motif_Score_SC	Other_Yeasts	Two-Hop_BE	Two-Hop_BP	Two-Hop_BY	Two-Hop_BG	Max_Membership	Sum
MBP1	YMR258C	5.5182			0.354257					5.87246
MBP1	YMR279C	2.9117		3.2448					0.659083	6.81558
MBP1	YMR304W				1.67147	0.942156	1.00812	1.52841		5.15016
MBP1	YMR307W	2.94027			0.501268				0.73134	4.17288
MBP1	YNL015W	0.64762		2.5957	1.69003					4.93335
MBP1	YNL030W	2.09512	0.0925508		1.90617	0.769498	0.971821			5.83516
MBP1	YNL067W				1.50532		1.80292		0.69605	4.00429
MBP1	YNL077W	2.55418	0.386934	1.15792						4.09903
MBP1	YNL079C	2.16109			0.41768		1.06825	0.883409		4.53043
MBP1	YNL082W	0.532652	0.46196	2.86038	2.21702				0.555972	6.62798
MBP1	YNL090W	0.521405	1.35323				1.62854		0.50193	4.00511
MBP1	YNL102W	5.23693	1.15555	1.25485	1.81226	0.451972				9.91156
MBP1	YNL154C	2.27788				2.24613			0.659103	5.18311
MBP1	YNL162W				1.49977		1.79078		0.723105	4.01365
MBP1	YNL165W	0.93326	0.386934	3.53979						4.85998
MBP1	YNL166C	0.93326	0.386934	3.54863		0.501849				5.37067
MBP1	YNL178W	0.838698			1.62177		1.78727		0.69735	4.94509
MBP1	YNL186W	0.155277			1.59785		1.55197	1.53849		4.84359
MBP1	YNL231C	2.36289	0.562527		0.912475		1.21865		0.5356	5.59214
MBP1	YNL233W	1.50117	1.53233	1.79425	1.92196				0.644649	7.39436
MBP1	YNL241C	0.990143	1.58446		1.94946					4.52406
MBP1	YNL262W	1.53547			0.978966	1.24902	1.75592	1.06876		6.58814
MBP1	YNL273W	5.39402	0.386934	1.46536	1.98989					9.2362
MBP1	YNL274C	5.39402	0.386934	2.63823	2.05817				0.730419	11.2078
MBP1	YNL289W	6.82139	1.20026							8.02165
MBP1	YNL291C	6.82139	1.20026							8.02165
MBP1	YNL298W	2.96923	0.577888	1.51252						5.05964
MBP1	YNL300W	0.999162		0.704344	1.95416				0.721879	4.37955
MBP1	YNL312W	4.08208	0.524868	0.913802	1.9235	0.329926			0.73043	8.50461
MBP1	YNL313C	4.08208	0.524868	0.605792	0.89788				0.658511	6.76913
MBP1	YNR009W	3.74003	0.680054						0.557542	4.97763
MBP1	YNR010W	3.74003	0.680054		0.436311					4.8564
MBP1	YNR019W	3.44975			0.423951				0.641275	4.51498
MBP1	YNR041C	4.04444			0.359014				0.705083	5.10854
MBP1	YNR044W	4.04444							0.709255	4.75369
MBP1	YOL017W	5.34894		1.8472	1.75005				0.632377	9.57857
MBP1	YOL018C	5.34894		1.4683			1.6684		0.659312	9.14495
MBP1	YOL039W			2.11414	1.26284		0.980327			4.35731
MBP1	YOL040C			3.431	1.396		1.81204			6.63904
MBP1	YOL041C				2.13268	0.38743	1.54711		0.631057	4.69828
MBP1	YOL082W	1.53448		0.0543381	1.73838				0.700381	4.02758
MBP1	YOR001W	3.59459			0.997461					4.59205
MBP1	YOR033C	1.54613	0.386934	3.81121	0.989435		1.39223		0.691588	8.81753
MBP1	YOR074C	8.69034	1.58446	3.14652	2.28975			0.869805		16.5809
MBP1	YOR075W	8.69034	1.58446	5.57028		0.34416				16.1892
MBP1	YOR101W					1.33348	1.11907	1.98462		4.43717
MBP1	YOR157C				1.63272	1.26016	1.80369			4.69657
MBP1	YOR167C				1.70393	1.27937	1.69692			4.68022
MBP1	YOR210W				0.702923	1.59443	1.29186		0.671014	4.26023
MBP1	YOR212W			1.93603		2.00146	1.01394			4.95143
MBP1	YOR234C	1.72426			1.64429				0.714797	4.08335
MBP1	YOR236W	1.72426					1.1978	2.12507	0.627343	5.67447
MBP1	YOR246C	3.6354					0.817958			4.45336
MBP1	YOR247W	3.6354			0.483357					4.11876
MBP1	YOR289W	1.07061		0.220529	0.748852		1.28405		0.715624	4.03967
MBP1	YOR312C	0.769734	0.834037		1.76587				0.72231	4.09195
MBP1	YOR326W	0.121857				0.615128	0.791662	2.46958	0.709707	4.70793
MBP1	YOR378W	0.0892987		2.54745			0.813636		0.643344	4.09373
MBP1	YPL014W	2.77042	1.28701	8.08703					0.640228	12.7847
MBP1	YPL015C	2.77042	1.8421	6.4249						11.0374
MBP1	YPL043W				2.06074		1.50249		0.68059	4.24382
MBP1	YPL079W			0.114186	1.50957		1.73051		0.729471	4.08374
MBP1	YPL081W	3.80355	0.386934		0.71324		1.80177		0.715401	7.42089
MBP1	YPL082C	3.80355	0.386934	2.40246		0.643054		1.00142		8.23742
MBP1	YPL090C				1.76691		1.76686		0.693962	4.22773
MBP1	YPL093W				2.24643	0.60639	1.79095			4.64377
MBP1	YPL126W	1.81326	0.457574	1.20126	0.563363				0.662706	4.69816
MBP1	YPL152W	2.42362	0.386934	2.20087						5.01142
MBP1	YPL153C	2.42362	0.386934	2.91432	1.73548	0.942156				8.40251
MBP1	YPL160W			0.776288	1.27633		1.78339		0.700307	4.53632
MBP1	YPL166W			4.24433						4.24433
MBP1	YPL167C			3.56225			1.29921			4.86146
MBP1	YPL179W	1.74497	0.386934	2.80128						4.93318
MBP1	YPL196W	2.75404			1.50347					4.25751
MBP1	YPL198W				1.92422		1.78433		0.660635	4.36918
MBP1	YPL218W	1.32472	1.15555	0.578264		1.47619	1.14266			5.67738
MBP1	YPL230W	1.75143	0.386934	1.11917	0.592475				0.640081	4.49009
MBP1	YPL237W				1.43823	0.396039	1.78865		0.732484	4.3554
MBP1	YPL241C	4.91629	0.515569	0.0565211	0.601419				0.701217	6.79102
MBP1	YPL242C	4.91629	0.515569			2.00146	1.06439			8.49771
MBP1	YPL256C	1.16068			2.26432	1.72367			0.676277	5.82495
MBP1	YPL266W	2.41243	0.386934		2.02929				0.694842	5.5235
MBP1	YPL267W	2.41243	0.386934	4.05537	2.46817				0.640037	9.96294
MBP1	YPR016C	1.5871	0.386934		1.78794		1.7457		0.648765	6.15644
MBP1	YPR017C	1.5871	0.386934	2.99689						4.97092
MBP1	YPR018W	1.5871	0.386934	2.43715	1.11589				0.519236	6.04631
MBP1	YPR043W				1.92506		1.80041		0.679636	4.40511
MBP1	YPR075C	9.21791	1.58395	4.18841					0.706799	15.6971
MBP1	YPR079W	9.21791	1.58395							10.8019
MBP1	YPR103W				1.74707	2.44997	1.80307			6.00011
MBP1	YPR119W	1.79758		2.00247	0.471056	1.72367	1.13251			7.12729
MBP1	YPR120C	3.7698		5.66597	2.31294					11.7487
MBP1	YPR135W	1.87236	0.834037	2.35527	0.96966	1.24902			0.711185	7.99153
MBP1	YPR165W	0.812393	1.09929	4.1041		0.487732	1.07849			7.58201
MBP1	YPR174C	2.49844	0.524868	2.20414	1.18817				0.712365	7.12798
MBP1	YPR175W	2.49844	0.524868	1.26367	1.61831	0.643054			0.726429	7.27477
MBP1	YPR184W	0.672174	0.386934	7.36943		1.05112			0.708463	10.1881
MCB	YCL010C			6.86473						6.86473
MCB	YDL106C			4.22601						4.22601
MCB	YDR058C			11.4292						11.4292
MCB	YDR310C			5.84055						5.84055
MCB	YDR353W			5.75025						5.75025
MCB	YEL047C			6.75845						6.75845
MCB	YER111C			5.08339						5.08339
MCB	YER112W			4.63635						4.63635
MCB	YER188W			4.47564						4.47564
MCB	YFL039C			6.61191						6.61191
MCB	YGL050W			4.90112						4.90112
MCB	YGR165W			11.2242						11.2242
MCB	YHL034C			18.045						18.045

TF	Target	Binding_0-2000	Motif_Score_SC	Other_Yeasts	Two-Hop_BE	Two-Hop_BP	Two-Hop_BY	Two-Hop_BG	Max_Membership	Sum
MCB	YJL039C			11.2558						11.2558
MCB	YJL045W			9.08095						9.08095
MCB	YJR048W			4.12934						4.12934
MCB	YJR055W			9.15074						9.15074
MCB	YKL045W			5.06404						5.06404
MCB	YKL046C			5.55036						5.55036
MCB	YKL112W			5.02475						5.02475
MCB	YKL113C			9.11043						9.11043
MCB	YKR044W			8.04255						8.04255
MCB	YLR348C			4.54967						4.54967
MCB	YLR350W			4.55489						4.55489
MCB	YML071C			14.5784						14.5784
MCB	YMR016C			4.27934						4.27934
MCB	YMR017W			4.61371						4.61371
MCB	YMR122W-A			5.51619						5.51619
MCB	YMR179W			6.83252						6.83252
MCB	YNL210W			5.81879						5.81879
MCB	YNL211C			4.97453						4.97453
MCB	YNL233W			4.92705						4.92705
MCB	YNL298W			9.21743						9.21743
MCB	YOL122C			5.22305						5.22305
MCB	YOL147C			6.36982						6.36982
MCB	YOR008C			4.97521						4.97521
MCB	YOR075W			4.02622						4.02622
MCB	YPL152W			5.16008						5.16008
MCB	YPL153C			4.17869						4.17869
MCB	YPR075C			7.29692						7.29692
MCB	YPR174C			4.68898						4.68898
MCM1	SNR40	8.97703								8.97703
MCM1	YAL021C					0.869761	1.45437	1.89986		4.22399
MCM1	YAL038W	7.03109								7.03109
MCM1	YAL040C	8.37823	0.460081			1.66744	1.32301		0.536372	12.3651
MCM1	YAR002C-A	1.98777				1.89425	1.49601			5.37803
MCM1	YAR018C	4.83319	0.713704	5.47017					0.440601	11.4577
MCM1	YAR050W	1.75863	0.519291	0.0658471				1.73182		4.07559
MCM1	YBL089W				0.592434	1.89425	1.53586			4.02254
MCM1	YBR038W	3.42075	0.201138		1.7997					5.42159
MCM1	YBR060C	0.897997				2.14863	1.47315			4.51978
MCM1	YBR067C	0.633689	1.24517	3.46584						5.3447
MCM1	YBR139W	3.13448					0.97704		0.598631	4.71015
MCM1	YBR155W				0.962868	1.61966	1.47315			4.05568
MCM1	YBR160W	0.707618	0.519291	1.47778		2.14863	1.14309			5.99641
MCM1	YBR175W						1.44387	2.07639	0.63591	4.15617
MCM1	YBR202W	0.0839978	0.0925508	1.08512	1.76708	1.13618	1.21428		0.69011	6.06932
MCM1	YCL039W		0.576047		0.527336	1.47837	1.53244			4.11419
MCM1	YCL059C		1.06843	1.6201	0.525607	0.375506			0.460093	4.04974
MCM1	YDL089W	2.03516	0.556986	6.19767						8.78982
MCM1	YDL090C	2.03516	0.556986	5.68758						8.27973
MCM1	YDL106C					2.85449	1.41213			4.26662
MCM1	YDL124W		1.14778	2.36594	0.594629					4.10835
MCM1	YDR007W			4.43769						4.43769
MCM1	YDR009W			2.54901		2.40066				4.94967
MCM1	YDR028C		0.887161	1.99348			1.24325			4.12389
MCM1	YDR146C	11.1842		2.71982	1.63192	1.46816	1.45506	2.04647	0.725461	21.2311
MCM1	YDR147W	11.1842		4.3336			1.45506			16.9729
MCM1	YDR150W	2.40568					0.872625	1.21647		4.49477
MCM1	YDR165W			1.90419			1.44903		0.725035	4.07826
MCM1	YDR179C	3.99392	0.415422							4.40934
MCM1	YDR190C	2.77328	0.421733	0.0919955			1.17739		0.722251	5.18665
MCM1	YDR191W	2.77328	0.421733	0.43826					0.453275	4.08655
MCM1	YDR267C					3.11852	1.45245			4.57097
MCM1	YDR293C		0.651051	3.95397	2.43206					7.03708
MCM1	YDR389W	4.83334	0.22731	0.0811449		2.70682				7.84861
MCM1	YDR451C	4.26814	0.495058	2.79013	1.27869		1.41007			10.2421
MCM1	YDR452W	4.26814	1.24517	2.14575						7.65906
MCM1	YDR459C	8.25572	0.767369							9.02309
MCM1	YDR461W	8.25572	0.767369							9.02309
MCM1	YDR462W	8.25572	0.767369							9.02309
MCM1	YDR463W	8.25572	0.767369					2.57417		11.5973
MCM1	YDR490C			4.20752			1.25508			5.4626
MCM1	YDR492W			4.05297	0.692112	0.466816			0.625709	5.83761
MCM1	YDR545W		0.519291				1.08133	2.09461	0.718352	4.41358
MCM1	YEL013W					2.70682	1.50212			4.20894
MCM1	YEL032W	1.73905	0.620072	1.1162	0.581229	1.13618	1.17992			6.37265
MCM1	YEL034W				2.42881		1.21299		0.705969	4.34777
MCM1	YEL040W	6.31849	1.46137	4.38147	0.644838				0.729779	13.5359
MCM1	YEL043W	6.21229			0.337806					6.5501
MCM1	YEL044W	6.21229		1.8651						8.07739
MCM1	YEL046C	6.21229		1.76033	0.666531					8.63915
MCM1	YEL075C	0.944258	0.519291			2.19041		2.16202	0.726713	6.54269
MCM1	YEL076C	0.944258	0.519291					2.15491	0.711355	4.32981
MCM1	YER032W			2.58105	1.82088	3.11852				7.52045
MCM1	YER033C	1.0769	0.620072	4.55079						6.24776
MCM1	YER034W	1.0769	0.620072	4.46453						6.1615
MCM1	YER044C	1.98939		0.160439	1.43963				0.695528	4.28499
MCM1	YER082C				1.86372	2.03433	1.52161			5.41966
MCM1	YER111C	3.87499	0.460081			1.89824	0.975678			7.20899
MCM1	YER112W	3.87499	0.460081			0.580377	1.45752			6.37297
MCM1	YER146W			2.06233		0.881355	1.26589		0.564274	4.77385
MCM1	YER150W	0.139212	0.620072	1.7922	1.57838					4.12986
MCM1	YER189W		0.519291	1.29754				2.16202	0.707991	4.68684
MCM1	YER190W		0.519291				1.07718	2.10314	0.727338	4.42695
MCM1	YFL026W	9.33641	1.59524	13.0087	1.84523					25.7856
MCM1	YFL027C	9.33641	1.59524	14.6184						25.55
MCM1	YFL051C			1.88708	1.6319			1.75488		5.27386
MCM1	YFL065C		0.519291		0.721603			2.15226	0.729852	4.12301
MCM1	YFL066C		0.519291		0.652133			2.14055	0.726262	4.03824
MCM1	YGL021W	3.244	0.0838537	2.47319	2.07767		1.38555		0.732698	9.99696
MCM1	YGL032C	4.19354			1.16622				0.569655	5.92941
MCM1	YGL086W	0.998154	0.574819			2.71926				4.29223
MCM1	YGL110C			0.281293	1.28008		1.49994	0.990146		4.05146
MCM1	YGL116W	10.6831			1.63805	1.89824	1.46279			15.6822
MCM1	YGL201C	3.64697		0.400561		0.94565	1.18283		0.5253	6.70131
MCM1	YGR041W	1.55644	0.460081	3.11246						5.12898
MCM1	YGR047C	8.84916		1.35975						10.2089
MCM1	YGR048W	8.84916		2.29736						11.1465
MCM1	YGR049W	8.84916				2.19041			0.729189	11.7688
MCM1	YGR055W			4.0474						4.0474
MCM1	YGR092W	3.83205	0.364039	0.719007	2.05805				0.64445	7.6176
MCM1	YGR168C	2.40856	0.0664192	4.49204						6.96702

TF	Target	Binding_0-2000	Motif_Score_SC	Other_Yeasts	Two-Hop_BE	Two-Hop_BP	Two-Hop_BY	Two-Hop_BG	Max_Membership	Sum
MCM1	YGR172C		1.24517	0.285594		2.19041			0.716924	4.4381
MCM1	YGR279C			6.42798						6.42798
MCM1	YGR296W		0.519291				1.08133	2.09461	0.730997	4.42623
MCM1	YHR005C	4.20453		0.176663	0.610384		1.52065			6.51223
MCM1	YHR023W	0.553653	0.839899		2.18876				0.73036	4.31267
MCM1	YHR026W		0.431817			2.41608	1.44168		0.713312	5.00289
MCM1	YHR030C		0.235138	1.38593		2.05372	1.25508			4.92987
MCM1	YHR049W		0.767369			2.19041	1.43425			4.39203
MCM1	YHR057C		0.37677			2.63977		1.7329		4.74944
MCM1	YHR098C	8.94367	0.495058	0.652506					0.723879	10.8151
MCM1	YHR099W	8.94367	0.495058	0.266252		2.07298	1.34377			13.1217
MCM1	YHR128W			0.824283		3.11852			0.723885	4.66669
MCM1	YHR129C		1.25987		1.02496	1.89425	1.49191			5.67099
MCM1	YHR148W			0.941211	1.81646		1.1746		0.691626	4.6239
MCM1	YHR149C	1.27427	1.14778	3.52551						5.94756
MCM1	YHR150W	1.27427	1.14778	3.61404						6.03609
MCM1	YHR151C	12.1622	0.495058	12.7134						25.3707
MCM1	YHR152W	12.1622	0.495058	12.0796	0.356416					25.0933
MCM1	YHR154W	0.541242	0.495058		1.14034		1.49039		0.483404	4.15043
MCM1	YIL015W	8.98151			1.28731		1.47169			11.7405
MCM1	YIL017C	8.98151								8.98151
MCM1	YIL069C	6.88319	0.100567				1.13796			8.12172
MCM1	YIL070C	6.88319	0.100567		0.444264					7.42802
MCM1	YIL076W	4.63661								4.63661
MCM1	YIL077C	4.63661								4.63661
MCM1	YIL106W			3.92052	1.12673	0.869761			0.718663	6.63567
MCM1	YIL107C			4.99311						4.99311
MCM1	YIL122W	2.19694	0.439368	1.77296	0.939273					5.34854
MCM1	YIL123W	6.81852	0.0246271	0.95067	0.825893					8.61971
MCM1	YIL158W	11.1162	0.612149	0.478744	1.31139					13.5185
MCM1	YJL049W	4.24222								4.24222
MCM1	YJL051W	12.2769	0.767369		1.27717		0.978964			15.3004
MCM1	YJL052C-A	12.2769	0.767369							13.0443
MCM1	YJL076W			2.13809			1.45618		0.722416	4.31669
MCM1	YJL081C		0.519291			2.24965	1.39208			4.16102
MCM1	YJL104W		1.14778	6.40214	0.584578					8.1345
MCM1	YJL157C	6.78695		2.65876	1.10078	2.09703				12.6435
MCM1	YJL158C	4.00851		0.650932	1.39991				0.595078	6.65443
MCM1	YJL159W	8.31051	1.59524	8.08796	1.09796					19.0917
MCM1	YJL160C	8.31051	1.59524							9.90575
MCM1	YJL168C				0.589663	3.11852	1.09999			4.80817
MCM1	YJL194W	8.56939	0.558218	0.721206			1.34034			11.1892
MCM1	YJL196C	8.56939	0.558218	0.727112	0.675998					10.5307
MCM1	YJL198W	2.87221			1.47963					4.35184
MCM1	YJR004C		0.713704	9.49605						10.2098
MCM1	YJR063W			0.684867	1.77548		1.22354		0.715953	4.39984
MCM1	YJR064W		0.839899	0.827498		2.2791			0.710842	4.65734
MCM1	YJR091C	12.0345	0.767369			1.33123	0.976179			15.1093
MCM1	YJR119C					2.19041	1.49191	1.6734		5.35572
MCM1	YKL059C	2.39209	0.635279	0.0709255				1.72823		4.82652
MCM1	YKL121W			1.282	1.91848		1.52206			4.72254
MCM1	YKL143W		1.42925	1.17415	1.07352				0.604889	4.28181
MCM1	YKL144C				0.461234	2.19041	1.26058		0.691823	4.60405
MCM1	YKL163W	5.37879	0.635279	0.144735	1.69682					7.85562
MCM1	YKL164C	5.37879	0.635279		1.47084				0.601717	8.08663
MCM1	YKL195W				1.63149	1.46205	1.21228			4.30582
MCM1	YKL207W	7.32168	1.42925							8.75093
MCM1	YKL208W	7.32168	1.42925	3.731						12.4819
MCM1	YKL209C	7.32168	1.42925	2.07562						10.8266
MCM1	YKL214C			4.01027	1.63183				0.600063	6.24216
MCM1	YKR036C	1.59724				0.869761	1.39912	1.82535		5.69147
MCM1	YKR042W	3.92787	0.439368	0.587765	0.390575				0.730131	6.07571
MCM1	YKR048C			1.34856		1.89824	1.44808			4.69488
MCM1	YKR090W		0.134511		1.83219		1.48908		0.669342	4.12512
MCM1	YKR097W	3.95442	0.576047	1.62513						6.1556
MCM1	YLR040C		1.14778	6.41843						7.56621
MCM1	YLR084C	7.08061	0.558218	10.0491	1.64148		1.58905		0.681804	21.6003
MCM1	YLR086W	7.08061	0.558218	0.237352						7.87618
MCM1	YLR131C	7.766	1.24517		1.45317		1.27679	1.64872		13.3898
MCM1	YLR133W	7.766	1.24517							9.01117
MCM1	YLR179C			2.26232			1.45083		0.468125	4.18127
MCM1	YLR189C	8.8411		1.41541						10.2565
MCM1	YLR190W	8.8411		1.39224	2.11056				0.650114	12.994
MCM1	YLR254C				1.78472		0.952351	1.36319		4.10026
MCM1	YLR265C		0.767369	3.89412						4.66149
MCM1	YLR273C	4.81895	0.421733	2.30225						7.54293
MCM1	YLR274W	4.81895	0.421733	0.605381	0.484874	0.62927	1.18047		0.537906	8.67858
MCM1	YLR409C	0.541333		1.03091	1.2334		1.48339		0.714257	5.00329
MCM1	YLR429W		0.574819				1.46592	1.8661	0.568056	4.4749
MCM1	YLR452C	0.439428		1.55168	1.25934	0.970317			0.547731	4.7685
MCM1	YLR466W		0.519291		1.17967			2.1372	0.730891	4.56705
MCM1	YLR467W		0.519291				1.08133	2.09461	0.730122	4.42535
MCM1	YML051W	9.503	0.431817	0.139773						10.0746
MCM1	YML052W	9.503	0.266343		1.81483					11.5842
MCM1	YML053C	9.503	0.431817	0.281237						10.2161
MCM1	YML081C-A		1.24517	9.46827						10.7134
MCM1	YML081W		1.24517	8.00205						9.24722
MCM1	YML093W				1.85399		1.45593	0.990146	0.5473	4.84737
MCM1	YML109W	4.41975	0.292216							4.71197
MCM1	YML110C	4.41975	0.292216							4.71197
MCM1	YML119W		0.331447	8.20602					0.726358	9.26383
MCM1	YML120C		0.331447	8.00035						8.3318
MCM1	YML124C	0.180667	1.24517			2.2791			0.628672	4.33361
MCM1	YMR001C	3.63244	0.421733	1.19785	2.06682		1.34043		0.692418	9.35169
MCM1	YMR002W	3.63244	0.421733	1.68268						5.73685
MCM1	YMR003W	3.63244	0.421733							4.05417
MCM1	YMR012W				1.01268	0.885944	1.50874		0.713345	4.12071
MCM1	YMR031C	3.70527						0.982288		4.68756
MCM1	YMR032W	3.70527			2.03491				0.706327	6.44651
MCM1	YMR116C				1.18932	0.949492	1.39189		0.652379	4.18308
MCM1	YMR121C	1.37391	0.651051		0.590607		1.15477		0.729028	4.49937
MCM1	YMR146C		1.24517	0.0437176			1.46149	1.89785	0.587401	5.23563
MCM1	YMR251W-A		1.14778	6.71381	0.904739					8.76633
MCM1	YMR252C	5.33369								5.33369
MCM1	YMR253C	5.33369								5.33369
MCM1	YMR255W	5.33369								5.33369
MCM1	YNL053W	11.1128								11.1128
MCM1	YNL056W	6.83741		0.136414						6.97382
MCM1	YNL058C	6.83741			1.39602				0.615162	8.84859
MCM1	YNL111C	0.445525			1.8653		0.976179		0.726464	4.01347

TF	Target	Binding_0-2000	Motif_Score_SC	Other_Yeasts	Two-Hop_BE	Two-Hop_BP	Two-Hop_BY	Two-Hop_BG	Max_Membership	Sum
MCM1	YNL145W	9.05275	1.0022		1.90316					11.9581
MCM1	YNL146C-A	9.05275	1.0022							10.0549
MCM1	YNL148C	9.05275	1.0022							10.0549
MCM1	YNL160W	2.04522	1.59524	4.66317						8.30363
MCM1	YNL289W	8.97703	0.651051	0.519764			1.32301		0.4966	11.9675
MCM1	YNL291C	8.97703	0.651051							9.62808
MCM1	YNL298W	8.19213		2.59265						10.7848
MCM1	YNL327W		1.02379	2.76259	0.809677				0.616406	5.21246
MCM1	YNL328C		1.02379	3.45575						4.47954
MCM1	YNL339C		0.519291	0.456463			1.08133	2.09461	0.650123	4.80182
MCM1	YNR026C	6.72252	0.186692							6.90921
MCM1	YNR028W	6.72252	0.186692	5.54289						12.4521
MCM1	YNR062C	11.174	0.331447	5.33977						16.8452
MCM1	YNR063W	11.174	0.331447							11.5054
MCM1	YNR067C			1.25737	1.11497		1.17151		0.619425	4.16328
MCM1	YOL108C			6.07673		0.911924				6.98865
MCM1	YOL117W	1.33421		5.81879						7.153
MCM1	YOL119C	1.33421		6.88693						8.22114
MCM1	YOL151W	1.85688			1.13364		1.26551			4.25603
MCM1	YOR066W	2.66815	0.967358	1.98821	0.876516					6.50023
MCM1	YOR116C			2.30366		2.07298				4.37664
MCM1	YOR117W		0.612149	5.28019	1.59365					7.48599
MCM1	YOR126C		0.839899	3.18729						4.02719
MCM1	YOR127W		0.839899	3.17902						4.01892
MCM1	YOR211C		0.358301	4.61899			1.33455			6.31184
MCM1	YOR212W		0.358301	4.81155		1.66744	1.45245			8.28974
MCM1	YOR313C	6.74688	1.59524		0.989994				0.510975	9.84309
MCM1	YOR315W	6.74688	1.59524	4.95963	0.989994				0.653177	14.9449
MCM1	YOR317W				1.29501	2.70682			0.481899	4.48373
MCM1	YOR326W					1.5029	1.46594	1.22678	0.709707	4.90533
MCM1	YOR388C	5.00216								5.00216
MCM1	YOR389W	5.00216			0.640548					5.64271
MCM1	YPL072W		1.10209	2.14766			1.33499			4.58474
MCM1	YPL085W	0.105639					1.20854	2.73577		4.04995
MCM1	YPL183C			1.89448			1.4004	2.21864	0.632697	6.14622
MCM1	YPL184C		0.172867	0.543733	1.27388		1.52841	1.72823		5.24712
MCM1	YPL187W		1.02379	4.99597						6.01976
MCM1	YPL212C		1.25987	3.00411	1.09926		1.09129		0.60576	7.06029
MCM1	YPL270W		0.519291	1.67022		2.19041				4.37992
MCM1	YPL277C	7.15516								7.15516
MCM1	YPL283C		0.519291				1.08133	2.09461	0.732246	4.42748
MCM1	YPR110C			0.837856	1.02896	0.965218	1.17639		0.716965	4.72539
MCM1	YPR112C	8.85183	0.325034	3.36168					0.629925	13.1685
MCM1	YPR113W	8.85183	0.325034	3.59731			1.32901		0.641748	14.7449
MCM1	YPR114W	8.85183	0.325034			2.19041				11.3673
MCM1	YPR119W	7.64189	0.460081	0.185746	1.69833	2.71926	1.49191		0.725537	14.9228
MCM1	YPR120C	0.14041		0.0458315	0.830618	2.19041	1.49191			4.69918
MCM1	YPR202W		0.519291		0.61406			2.15978	0.714327	4.00746
MET18	TW(CCA)J	4.17431								4.17431
MET18	YER188C-A	5.41112								5.41112
MET18	YER189W	5.41112			0.862052			1.53416		7.80733
MET18	YER190W	5.41112			0.862052			1.53416		7.80733
MET18	YLR287C-A	4.28618								4.28618
MET18	YLR288C	4.28618								4.28618
MET18	YLR289W	4.28618								4.28618
MET18	YNL336W	6.3436								6.3436
MET18	YNL339C	6.3436								6.3436
MET28	YOR339C	4.09373								4.09373
MET31	YAL011W	5.3303								5.3303
MET31	YAL012W	5.3303			0.497974				0.714342	6.54262
MET31	YAL014C	5.3303				0.480456				5.81076
MET31	YBR013C	2.23787		1.50446	0.567994					4.31032
MET31	YBR159W		1.87246	5.17335						7.04581
MET31	YBR282W		1.87246	5.10686						6.97932
MET31	YCL036W		1.87246	5.53445						7.40691
MET31	YCL050C			5.36534						5.36534
MET31	YDL058W	1.40358	1.87246	4.18086						7.4569
MET31	YDL059C	1.40358	1.87246	8.63465					0.675426	12.5861
MET31	YDL208W		1.87246	1.47778	0.444242	0.408375				4.20286
MET31	YDR042C	2.30769	1.87246							4.18015
MET31	YDR165W	0.172654	1.87246	4.49185	0.408042					6.94501
MET31	YDR253C	0.675447	1.87246	4.51136					0.706791	7.76606
MET31	YDR254W	0.675447	1.87246	1.73103					0.477325	4.75626
MET31	YDR478W			5.37942						5.37942
MET31	YDR502C	1.81848	1.87246	5.61824	0.899661					10.2088
MET31	YEL002C			5.76344						5.76344
MET31	YEL012W	0.745267		3.70576						4.45103
MET31	YEL058W	0.0638778		4.30018						4.36406
MET31	YEL059C-A		0.224045	4.73437						4.95842
MET31	YEL060C	0.0638778		5.2382					0.474902	5.77698
MET31	YEL065W		1.87246	2.74558						4.61804
MET31	YEL068C		1.87246	3.21267						5.08513
MET31	YER091C	5.68621	1.87246	5.1624					0.709264	13.4303
MET31	YER092W	5.68621	1.87246	7.46368						15.0223
MET31	YFR030W	0.586106	0.224045	1.41303	0.78947			1.38748	0.567915	4.96805
MET31	YFR031C-A	0.39257	1.87246		0.638504	1.90009				4.80362
MET31	YFR034C	0.370912	1.87246	2.40658						4.64995
MET31	YGL043W		0.224045	3.88546						4.10951
MET31	YGL058W			5.39865						5.39865
MET31	YGL063W		0.0972284	3.20263			1.16145			4.46131
MET31	YGL125W	0.567112	0.224045	1.80685	1.02016				0.708114	4.32628
MET31	YGL181W	5.41349								5.41349
MET31	YGL184C	5.41349		5.2964					0.672657	11.3825
MET31	YGR055W		1.87246	9.96256	0.959045					12.7941
MET31	YGR154C	0.0880114	1.87246	1.88227					0.510846	4.35359
MET31	YGR155W	0.0880114	1.87246	5.93884						7.89931
MET31	YGR202C	8.3545	0.224045							8.57855
MET31	YGR204W	8.3545								8.3545
MET31	YGR212W		1.87246	6.72118						8.59364
MET31	YGR254W		1.87246	4.03363						5.90609
MET31	YHL003C			4.81995						4.81995
MET31	YHL036W	0.0612718	1.87246	4.4604					0.730366	7.1245
MET31	YHL038C	0.0612718	1.87246	6.12191						8.05564
MET31	YHR112C		1.87246	6.46807					0.651625	8.99215
MET31	YHR113W		1.87246	5.43316					0.656366	7.96199
MET31	YHR191C	4.44691	0.224045							4.67096
MET31	YHR193C	4.44691								4.44691
MET31	YHR194W	4.44691	0.224045							4.67096
MET31	YIL007C		1.87246	5.57238						7.44484
MET31	YIL046W		1.87246	3.7948						5.66726

TF	Target	Binding_0-2000	Motif_Score_SC	Other_Yeasts	Two-Hop_BE	Two-Hop_BP	Two-Hop_BY	Two-Hop_BG	Max_Membership	Sum
MET31	YIL047C		1.87246	4.95095						6.82341
MET31	YIL094C			4.63977						4.63977
MET31	YIR017C		1.87246	3.57131					0.697679	6.14145
MET31	YIR018W		1.87246	8.8633					0.730521	11.4663
MET31	YJL060W	1.26943	0.224045	6.44801					0.704851	8.64634
MET31	YJL210W	2.3775		2.18645						4.56395
MET31	YJL212C	2.3775		2.48829						4.86579
MET31	YJR010W	0.114158	1.87246	10.0993				0.903162	0.639468	13.6285
MET31	YJR017C		1.87246	5.44483						7.31729
MET31	YJR020W		1.87246	3.25812						5.13058
MET31	YJR137C	4.5971							0.699732	5.29683
MET31	YJR138W	4.5971	0.224045							4.82115
MET31	YKL212W		1.87246	2.33578						4.20824
MET31	YKR044W		1.87246	3.38955						5.26201
MET31	YLL032C		0.224045	3.88404						4.10808
MET31	YLL061W	1.33257	1.87246	2.55627					0.497163	6.25846
MET31	YLR178C	3.81343	1.87246	0.0165996			1.13962		0.729313	7.57142
MET31	YLR179C	3.81343	1.87246	6.1057	0.807833					12.5994
MET31	YLR180W	3.81343	1.87246	2.84672	0.492147					9.02476
MET31	YLR242C	0.660215		4.3975						5.05771
MET31	YLR254C		0.224045	3.98569						4.20974
MET31	YLR321C		1.87246	2.82033						4.69279
MET31	YLR363C		1.87246	3.44255						5.31501
MET31	YLR364W		1.87246	7.08623						8.95869
MET31	YML065W		1.87246	3.39214		0.371778				5.63638
MET31	YMR090W			5.15466	0.5325				0.692905	6.38006
MET31	YMR140W		1.87246	5.66643						7.53889
MET31	YMR145C	0.284245	1.87246	3.02495						5.18166
MET31	YMR156C		0.0972284	7.00431						7.10154
MET31	YMR158W		0.0972284	4.4786						4.57583
MET31	YMR173W		1.87246	3.23001						5.10247
MET31	YMR173W-A		1.87246	2.43083						4.30329
MET31	YMR183C			5.05735			1.12064			6.17799
MET31	YMR185W		0.0972284	5.45427						5.5515
MET31	YMR205C		1.87246	3.55274						5.4252
MET31	YMR206W		1.87246	2.92939						4.80185
MET31	YNL191W	1.4896	1.87246	6.17509						9.53715
MET31	YNL241C		1.87246	5.48444					0.719942	8.07684
MET31	YNL277W	5.42925		2.24263					0.717305	8.38918
MET31	YNL278W		0.224045	4.55116			1.16354			5.93875
MET31	YOL032W		0.224045	4.27762						4.50167
MET31	YPR061C		1.87246	3.22265						5.09511
MET31	YPR108W		0.224045	3.54241			1.0446			4.81105
MET31	YPR167C	3.76073					0.780478		0.661532	5.20274
MET32	TV(AAC)E1	4.53978								4.53978
MET32	YAL011W	5.48026								5.48026
MET32	YAL012W	5.48026			0.913148				0.714342	7.10775
MET32	YAL014C	5.48026								5.48026
MET32	YAL019W			1.85785		0.464671	2.05208			4.3746
MET32	YBL087C	3.03724	0.0972284		1.01754					4.15201
MET32	YBR048W	3.31336			0.961141					4.2745
MET32	YBR159W		1.87246	5.17335						7.04581
MET32	YBR213W	2.70561	1.87246	2.08966						6.66773
MET32	YBR214W	4.94641	0.224045		0.753764					5.92422
MET32	YBR282W		1.87246	5.10686	0.336447					7.31577
MET32	YCL036W		1.87246	5.53445						7.40691
MET32	YCL050C			5.36534						5.36534
MET32	YCR081W	3.00194		2.40878						5.41072
MET32	YCR088W	2.34693			2.47854					4.82547
MET32	YDL002C		0.0972284		2.54975	3.9249				6.57188
MET32	YDL058W	3.41339	1.87246	4.18086						9.46671
MET32	YDL059C	3.41339	1.87246	8.63465					0.675426	14.5959
MET32	YDL208W	0.0760017	1.87246	1.47778	0.874369	0.593774				4.89438
MET32	YDR165W		1.87246	4.49185	0.838169					7.20248
MET32	YDR253C		1.87246	4.51136			0.851448		0.706791	7.94206
MET32	YDR254W		1.87246	1.73103					0.477325	4.08081
MET32	YDR264C				1.34524	0.442799	1.04177	1.6685		4.49831
MET32	YDR478W			5.37942						5.37942
MET32	YDR502C	0.362914	1.87246	5.61824	2.58906					10.4427
MET32	YEL002C	0.314012		5.76344						6.07745
MET32	YEL011W	1.67665		0.823825			1.83964		0.713823	5.05394
MET32	YEL058W			4.30018			0.872599			5.17278
MET32	YEL059C-A		0.224045	4.73437	0.44531					5.40373
MET32	YEL060C			5.2382			0.775281		0.474902	6.48838
MET32	YEL065W		1.87246	2.74558						4.61804
MET32	YEL068C		1.87246	3.21267						5.08513
MET32	YEL071W	0.230171	0.224045	1.11145	2.54207					4.10774
MET32	YER069W		1.87246	1.3608	1.07171				0.503362	4.80833
MET32	YER091C	15.8551	1.87246	5.1624	0.521753				0.709264	24.121
MET32	YER092W	15.8551	1.87246	7.46368						25.1912
MET32	YER134C	4.53978								4.53978
MET32	YER136W	4.53978								4.53978
MET32	YER189W	3.30146			0.431106			1.15401		4.88658
MET32	YER190W	3.30146			0.431106			1.15401		4.88658
MET32	YFL005W					1.06082	0.854819	2.13068		4.04632
MET32	YFR030W	10.2936	0.224045	1.41303	1.03415			1.60332	0.567915	15.1361
MET32	YFR031C-A	0.349084	1.87246		0.938137	3.9249				7.08458
MET32	YFR034C		1.87246	2.40658						4.27904
MET32	YGL043W		0.224045	3.88546			0.848452			4.95796
MET32	YGL058W			5.39865						5.39865
MET32	YGL063W		0.0972284	3.20263			2.04209			5.34195
MET32	YGL065C		0.0972284		2.46178		1.96521			4.52422
MET32	YGL125W	1.66255	0.224045	1.80685	2.55235		0.794783		0.708114	7.74869
MET32	YGL181W	8.72637					0.843997	1.29362		10.864
MET32	YGL184C	8.72637		5.2964					0.672657	14.6954
MET32	YGL253W	5.95717		0.269435						6.2266
MET32	YGL254W	5.95717		0.00176606						5.95894
MET32	YGL255W	4.19942					0.895909			5.09533
MET32	YGR055W	2.44652	1.87246	9.96256	3.03629					17.3178
MET32	YGR154C	0.939975	1.87246	1.88227					0.510846	5.20555
MET32	YGR155W	0.939975	1.87246	5.93884						8.75127
MET32	YGR202C	15.8551	0.224045				0.845083			16.9242
MET32	YGR204C-A	9.58806								9.58806
MET32	YGR204W	15.8551								15.8551
MET32	YGR206W	9.58806								9.58806
MET32	YGR208W	9.58806								9.58806
MET32	YGR212W		1.87246	6.72118	0.560484					9.15412
MET32	YGR254W		1.87246	4.03363	1.01712					6.92321
MET32	YHL003C			4.81995						4.81995
MET32	YHL036W		1.87246	4.4604					0.730366	7.06323

TF	Target	Binding_0-2000	Motif_Score_SC	Other_Yeasts	Two-Hop_BE	Two-Hop_BP	Two-Hop_BY	Two-Hop_BG	Max_Membership	Sum
MET32	YHL038C	0.514391	1.87246	6.12191						8.50876
MET32	YHR112C	0.744547	1.87246	6.46807					0.651625	9.7367
MET32	YHR113W	0.744547	1.87246	5.43316	0.694107				0.656366	9.40064
MET32	YHR115C	4.04275		0.110835			0.905695			5.05928
MET32	YHR117W	4.04275		0.0291362			0.848034			4.91992
MET32	YIL005W		1.87246		2.46519					4.33765
MET32	YIL007C		1.87246	5.57238						7.44484
MET32	YIL046W		1.87246	3.7948						5.66726
MET32	YIL047C		1.87246	4.95095						6.82341
MET32	YIL074C	0.907536			2.94496				0.691515	4.54401
MET32	YIL094C			4.63977						4.63977
MET32	YIR017C	0.915695	1.87246	3.57131	1.95601				0.697679	9.01315
MET32	YIR018W	0.915695	1.87246	8.8633					0.730521	12.382
MET32	YJL060W	3.14063	0.224045	6.44801					0.704851	10.5175
MET32	YJL148W	3.84958			0.743587					4.59317
MET32	YJR009C	4.63155	1.87246	0.156484	0.794596					7.45509
MET32	YJR010W	4.63155	1.87246	10.0993	2.25822			0.944912	0.639468	20.4459
MET32	YJR017C		1.87246	5.44483						7.31729
MET32	YJR020W		1.87246	3.25812						5.13058
MET32	YJR137C	5.7949			1.9531		0.862426	2.41396	0.699732	11.7241
MET32	YJR138W	5.7949	0.224045							6.01895
MET32	YKL180W	0.427283		3.1496	0.96288		0.854353			5.39412
MET32	YKL212W		1.87246	2.33578						4.20824
MET32	YKR044W		1.87246	3.38955	0.559821					5.82183
MET32	YLL032C		0.224045	3.88404						4.10808
MET32	YLL061W		1.87246	2.55627					0.497163	4.92589
MET32	YLR026C			0.937168	2.29265		1.93944			5.16926
MET32	YLR092W	14.0793	0.224045	0.79564	0.328283				0.643613	16.0709
MET32	YLR174W	2.80969					2.07598			4.88567
MET32	YLR178C	5.84586	1.87246	0.0165996			2.02026		0.729313	10.4845
MET32	YLR179C	5.84586	1.87246	6.1057	0.838465					14.6625
MET32	YLR180W	5.84586	1.87246	2.84672	2.56639					13.1314
MET32	YLR229C	1.46276					1.10351	1.60551		4.17178
MET32	YLR242C			4.3975						4.3975
MET32	YLR254C		0.224045	3.98569			0.825956			5.03569
MET32	YLR303W	2.81072	0.224045	1.44985	0.389002				0.713406	5.58702
MET32	YLR321C		1.87246	2.82033						4.69279
MET32	YLR363C		1.87246	3.44255						5.31501
MET32	YLR364W		1.87246	7.08623						8.95869
MET32	YML065W		1.87246	3.39214			0.845401			6.11
MET32	YML072C	6.42909					1.11372	1.70758		9.25039
MET32	YMR090W			5.15466	0.845924				0.692905	6.69349
MET32	YMR119W	1.61122	0.0972284	0.11612	2.54255					4.36712
MET32	YMR140W		1.87246	5.66643						7.53889
MET32	YMR145C		1.87246	3.02495	0.533996					5.43141
MET32	YMR156C		0.0972284	7.00431						7.10154
MET32	YMR158W		0.0972284	4.4786						4.57583
MET32	YMR173W		1.87246	3.23001	0.449827					5.5523
MET32	YMR173W-A		1.87246	2.43083						4.30329
MET32	YMR183C			5.05735			2.00128		0.607816	7.66645
MET32	YMR185W		0.0972284	5.45427						5.5515
MET32	YMR205C		1.87246	3.55274			0.775116			6.20032
MET32	YMR206W		1.87246	2.92939						4.80185
MET32	YMR304W					1.20911	1.11372	1.67747		4.0003
MET32	YNL189W				0.50287	3.33393	1.88019			5.71699
MET32	YNL191W	0.0688416	1.87246	6.17509						8.11639
MET32	YNL241C	0.131898	1.87246	5.48444					0.719942	8.20874
MET32	YNL273W	4.35188								4.35188
MET32	YNL277W	11.7624		2.24263					0.717305	14.7223
MET32	YNL278W		0.224045	4.55116			2.04417			6.81938
MET32	YNL284C	1.29181			2.51057		0.863989			4.66637
MET32	YNL305C		1.87246	1.99028	0.461181					4.32392
MET32	YNR047W		1.87246	1.8324				0.874715		4.57958
MET32	YOL032W		0.224045	4.27762						4.50167
MET32	YOL064C	3.06369	0.224045				1.35808		0.722266	5.36808
MET32	YOL083W	4.05233								4.05233
MET32	YPL015C		1.87246	0.129157			2.04926			4.05088
MET32	YPL075W		0.0972284	3.83951					0.624635	4.56137
MET32	YPR061C		1.87246	3.22265						5.09511
MET32	YPR091C	2.21918					1.87041			4.08959
MET32	YPR108W	0.167184	0.224045	3.54241			1.92523			5.85887
MET32	YPR166C	5.29507			0.471846					5.76692
MET32	YPR167C	5.29507			0.682544		0.92111		0.661532	7.56026
MET32	YPR168W	5.29507	0.0972284							5.3923
MET32	YPR169W	5.29507			0.448314					5.74338
MET4	YAL011W	6.41394	3.37231							9.78625
MET4	YAL012W	6.41394	3.37231	8.1378	0.533859				0.714342	19.1723
MET4	YAL014C	6.41394	3.37231							9.78625
MET4	YAL043C			4.57622						4.57622
MET4	YBL033C	0.634538	1.44243	3.01736						5.09433
MET4	YBL063W	0.217871		2.55536			1.31006			4.08329
MET4	YBR119W	0.618058	1.29891	2.71983						4.6368
MET4	YCR065W	1.71851	1.62032	0.990135	0.674337					5.0033
MET4	YDR184C		1.80911	11.4128						13.2219
MET4	YDR253C			9.84319					0.706791	10.55
MET4	YDR254W			5.21272					0.477325	5.69004
MET4	YDR437W		1.09594	9.69754						10.7935
MET4	YDR496C	0.812581	1.2096	3.22685	0.806418					6.05545
MET4	YDR502C		1.94524	3.11118	0.935547					5.99197
MET4	YEL047C	2.27968	2.60315							4.88283
MET4	YEL066W		2.01757	2.99416						5.01173
MET4	YER003C		2.60143	2.10692						4.70835
MET4	YER005W		2.60143	2.30079						4.90222
MET4	YER042W		1.35965	3.1998						4.55945
MET4	YER087W		1.1898	4.18747						5.37727
MET4	YER091C	7.31458	1.48861	4.76756	0.404681				0.659866	14.6353
MET4	YER092W	7.31458	1.48861	6.50343						15.3066
MET4	YFR030W	5.35813	0.310257						0.567915	6.2363
MET4	YFR031C-A	0.655565	1.30863		0.57683	2.26476				4.80579
MET4	YGL055W		1.84651	3.70316						5.54967
MET4	YGL056C		1.84651	3.67034						5.51685
MET4	YGL181W	8.58636	1.77095							10.3573
MET4	YGL184C	8.58636	1.77095	8.13091					0.672657	19.1609
MET4	YGL253W	1.40634		2.82948						4.23582
MET4	YGR055W	2.97238	3.12408	8.80577	1.29168					16.1939
MET4	YGR066C			8.10384						8.10384
MET4	YGR202C	9.70538								9.70538
MET4	YGR204W	9.70538	0.397713							10.1031
MET4	YGR261C		1.79477	2.36889						4.16366
MET4	YHL036W		2.59843	11.599					0.730366	14.9278

TF	Target	Binding_0-2000	Motif_Score_SC	Other_Yeasts	Two-Hop_BE	Two-Hop_BP	Two-Hop_BY	Two-Hop_BG	Max_Membership	Sum
MET4	YHL038C		2.59843	13.8506						16.449
MET4	YHR034C		2.71536	2.52578						5.24114
MET4	YIL070C			5.48029						5.48029
MET4	YIL074C	1.02842		2.50012	1.20034				0.691515	5.42039
MET4	YIL076W		1.63001	2.41434						4.04435
MET4	YIR017C	1.74644	0.244257	2.9688	0.332627				0.697679	5.9898
MET4	YIR018W	1.74644	0.244257	5.63818					0.730521	8.3594
MET4	YJL080C	0.531004	2.47156	1.66484						4.6674
MET4	YJL118W		2.24431	2.75504						4.99935
MET4	YJR010W	0.543572		4.39901				0.895338	0.602128	6.44005
MET4	YJR137C	0.935376			0.944927			1.52461	0.699732	4.10464
MET4	YKL144C			4.8308						4.8308
MET4	YKL186C			5.21445						5.21445
MET4	YLL025W	2.12594	1.40119		0.676966					4.2041
MET4	YLR004C			4.85417						4.85417
MET4	YLR054C			4.01699						4.01699
MET4	YLR178C	3.98909	3.02732				1.29822		0.729313	9.04394
MET4	YLR179C	3.98909	3.02732	10.7214	1.02919					18.767
MET4	YLR180W	3.98909	3.02732	6.38759	0.821773					14.2258
MET4	YLR186W	0.395042	1.53596	2.53295						4.46395
MET4	YLR242C		1.6193	4.30717						5.92647
MET4	YLR303W	1.56729	0.208347	3.90638						5.68202
MET4	YLR332W		1.83178	2.13476	0.341023					4.30756
MET4	YLR368W	2.26204	1.53129	9.03584	1.34707					14.1762
MET4	YLR399C	0.33543	1.93945	1.9183						4.19318
MET4	YML017W		0.601159	4.22594	1.63413					6.46123
MET4	YML018C		0.601159	4.12671						4.72787
MET4	YML050W			4.39329						4.39329
MET4	YML091C	0.393797	1.2244	7.93307						9.55127
MET4	YMR009W			4.02685					0.645702	4.67255
MET4	YNL066W	1.17362		3.67269						4.84631
MET4	YNL082W		0.831104	7.86826						8.69936
MET4	YNL220W	2.14639	0.0892073	1.9223						4.1579
MET4	YNL277W	4.05163	2.82292	2.43715					0.474908	9.78661
MET4	YNL294C		1.38472	3.17101						4.55573
MET4	YNR044W		0.964493	3.23001						4.1945
MET4	YOL056W			4.13273					0.614338	4.74707
MET4	YOR370C		1.22987	3.23215						4.46202
MET4	YPR086W			4.0076						4.0076
MET4	YPR093C		1.85485	4.34593						6.20078
MET4	YPR158W	0.46928	2.27032	4.68948						7.42908
MGA1	YCR105W	5.04108								5.04108
MGA1	YCR107W	4.64619								4.64619
MGA1	YDR369C	4.46852			0.454898					4.92342
MGA1	YDR370C	4.46852								4.46852
MGA1	YDR371W	4.46852			0.454898					4.92342
MGA1	YMR259C	4.4845								4.4845
MIG1	YBR033W	0.351692		4.05564						4.40733
MIG1	YBR050C	0.936107	1.69286	5.69699						8.32596
MIG1	YBR117C		0.65529	3.24789					0.538478	4.44166
MIG1	YBR281C	0.568102		4.2625						4.8306
MIG1	YBR297W	0.305297	2.19517	11.1877					0.521384	14.2096
MIG1	YCL040W		1.70193	5.30823					0.727922	7.73808
MIG1	YCL043C		1.70193	7.43235						9.13428
MIG1	YCR091W		1.17767	3.15924	0.450204				0.73227	5.51938
MIG1	YDL047W	0.0965	1.43611	5.44295						6.97556
MIG1	YDL048C	0.0965	1.43611	3.46999						5.0026
MIG1	YDL049C		1.40278	4.35917						5.76195
MIG1	YDR233C		1.04474	4.55921						5.60395
MIG1	YDR468C			4.17178						4.17178
MIG1	YDR469W			4.00766						4.00766
MIG1	YEL069C	4.76138	0.353835							5.11522
MIG1	YEL070W	6.20666		3.08792						9.29458
MIG1	YER118C		1.50947	2.64325						4.15272
MIG1	YER150W	0.656482		3.03296	0.399521				0.730806	4.81977
MIG1	YFL025C			5.30616						5.30616
MIG1	YFL039C		0.164953	7.47557						7.64052
MIG1	YFL052W		1.54838	4.7589						6.30728
MIG1	YFR014C		1.83129	8.81853						10.6498
MIG1	YFR015C			5.65993					0.732748	6.39268
MIG1	YFR053C		2.19517	11.6874					0.709322	14.5919
MIG1	YGL064C			6.28865						6.28865
MIG1	YGL080W		1.27672	3.82546						5.10218
MIG1	YGL146C	0.739053	0.777449	2.74867						4.26517
MIG1	YGR067C	0.210465	1.56026	3.38651						5.15724
MIG1	YGR243W	0.940726	0.511225	4.70507					0.526224	6.68325
MIG1	YHL032C	0.124112	2.06545	4.05223					0.708636	6.95043
MIG1	YHR033W	0.0875071		4.17833						4.26584
MIG1	YHR092C		1.57315	3.07715						4.6503
MIG1	YHR206W			4.35966						4.35966
MIG1	YIL156W			4.98342						4.98342
MIG1	YIL157C			5.25183						5.25183
MIG1	YIL162W		1.6529	10.5697						12.2226
MIG1	YKL062W		1.45401	4.92941						6.38342
MIG1	YKR075C	0.0764266	1.04474	4.70331						5.82448
MIG1	YKR076W	0.0764266	1.04474	4.50539						5.62656
MIG1	YLR044C		1.59883	4.41865						6.01748
MIG1	YLR089C		2.19517	10.5631						12.7583
MIG1	YLR090W		2.19517	10.7662						12.9614
MIG1	YML031W			4.75064						4.75064
MIG1	YMR044W		0.939792	4.87545						5.81524
MIG1	YMR081C		1.25334	3.28277					0.478939	5.01505
MIG1	YMR099C	0.629504	1.0306	4.46991						6.13001
MIG1	YMR205C		1.97697	6.40432						8.38129
MIG1	YMR206W		1.97697	6.29857					0.637388	8.91293
MIG1	YNL074C			5.24531						5.24531
MIG1	YNL109W		2.06545	5.96924						8.03469
MIG1	YNL110C		2.06545	16.5368	0.816034					19.4183
MIG1	YNL144C	0.241392	2.06545	7.19112					0.584464	10.0824
MIG1	YOL053W			5.18082						5.18082
MIG1	YOL096C		1.54838	3.21198					0.699371	5.45973
MIG1	YOL156W	4.57219		0.197057						4.76925
MIG1	YOL157C	4.57219		0.205672						4.77786
MIG1	YOR032C		0.434964	3.60063						4.03559
MIG1	YOR256C		1.54838	2.50427	0.507566					4.56022
MIG1	YOR257W		1.54838	2.5336						4.08198
MIG1	YOR258W		1.54838	2.53319						4.08157
MIG1	YOR288C	0.679663		10.0433	0.346293				0.728544	11.7978
MIG1	YPL057C		1.93027	5.90601						7.83628
MIG1	YPL200W	1.60311	0.233834	3.49846	0.842954					6.17836

TF	Target	Binding_0-2000	Motif_Score_SC	Other_Yeasts	Two-Hop_BE	Two-Hop_BP	Two-Hop_BY	Two-Hop_BG	Max_Membership	Sum
MIG1	YPR029C		1.97697	7.34936						9.32633
MIG1	YPR030W		1.97697	7.55434					0.633345	10.1647
MIG1	YPR184W	3.08212	1.28755	1.25422					0.730196	6.35409
MIG1	YPR196W		1.25116	3.63767						4.88883
MIG2	YDR343C	7.09075								7.09075
MIG2	YEL069C	4.04057								4.04057
MIG2	YER049W	3.64667			0.38317					4.02984
MIG2	YOL164W-A	4.93205								4.93205
MIG2	YOL165C	4.93205								4.93205
MIG3	YBR229C	4.78541								4.78541
MIG3	YBR230W-A	4.78541								4.78541
MIG3	YER189W	2.9089			0.350917			1.08327		4.34309
MIG3	YER190W	2.9089			0.350917			1.08327		4.34309
MOT3	YBL029W	0.975259		3.30172						4.27698
MOT3	YBR067C			4.01203						4.01203
MOT3	YFR028C	0.260527		3.74846						4.00899
MOT3	YHR179W	0.0872451		4.01899						4.10624
MOT3	YIL119C	3.16701		0.853046						4.02006
MOT3	YJR154W	1.79274		2.22104						4.01378
MOT3	YLR333C	0.433132		3.2448	0.652822					4.33075
MOT3	YLR363W-A			4.95551						4.95551
MOT3	YMR243C			4.2207						4.2207
MOT3	YMR253C	0.451775		3.69168						4.14345
MOT3	YPR102C	0.132447		3.93711	0.611169					4.68073
MSE	YBR133C			4.77413						4.77413
MSE	YGL162W			4.33251						4.33251
MSE	YGL184C			4.04341						4.04341
MSE	YIL127C			4.20159						4.20159
MSE	YIR021W			4.34166						4.34166
MSE	YJL122W			4.83103						4.83103
MSN1	YPR035W	5.15588								5.15588
MSN2	SNR18	6.13697	1.23206							7.36903
MSN2	TC(GCA)P2	4.76798								4.76798
MSN2	TE(UUC)M	4.02911								4.02911
MSN2	TF(GAA)N	3.40435	1.5458							4.95015
MSN2	TM(CAU)J2	6.34711	0.582795							6.9299
MSN2	TT(AGU)B	4.04704								4.04704
MSN2	TT(AGU)C	4.87888								4.87888
MSN2	TW(CCA)J	7.16596								7.16596
MSN2	TW(CCA)K	5.64344	0.34489							5.98833
MSN2	YAL003W	6.13697	1.23206							7.36903
MSN2	YAL005C	6.13697	1.23206		0.825163	0.77644				8.97063
MSN2	YAL040C	1.58968	1.89196	0.73302						4.21466
MSN2	YBL033C	0.281179		3.87406						4.15524
MSN2	YBL086C	0.376746	1.51051	3.65061	0.806457					6.34432
MSN2	YBL105C			4.43727	1.11871	0.468009				6.02399
MSN2	YBR009C	0.504273	1.51051		0.996285	1.16109	0.908262			5.08042
MSN2	YBR013C	4.04704			0.510637					4.55768
MSN2	YBR052C		1.89196	9.52619					0.723962	12.1421
MSN2	YBR070C	1.1941	1.13945	3.90634					0.671745	6.91164
MSN2	YBR071W		0.885999	3.36977	0.444967					4.70074
MSN2	YBR234C		1.12498	4.22068						5.34566
MSN2	YBR276C		1.89196	6.25912						8.15108
MSN2	YBR278W		1.89196	8.13567						10.0276
MSN2	YCL040W		0.99821	2.58637	0.907234				0.731261	5.22307
MSN2	YCL043C		0.99821	4.22911	0.563701				0.60055	6.39157
MSN2	YCR010C		1.89196	9.18737	0.363535				0.620205	12.0631
MSN2	YCR018C	1.44042		3.30673						4.74715
MSN2	YCR096C	4.87888				1.15236				6.03124
MSN2	YCR097W	4.87888				1.15236	0.862931			6.89417
MSN2	YDL004W		1.02606	2.52236	0.763092	0.3274			0.72703	5.36594
MSN2	YDL005C		1.02606	2.46208		0.678931				4.16707
MSN2	YDL047W	0.75525		8.61801						9.37326
MSN2	YDL130W-A		0.991939	3.06421						4.05615
MSN2	YDL174C		0.470096	3.42668	0.327097					4.22387
MSN2	YDR001C	2.83318	0.379313		0.671036				0.715436	4.59896
MSN2	YDR003W	2.83318	0.379313		0.688973				0.542841	4.44431
MSN2	YDR011W	4.50242	0.0892073						0.637464	5.22909
MSN2	YDR042C	5.0738								5.0738
MSN2	YDR066C	4.27429								4.27429
MSN2	YDR067C	4.27429								4.27429
MSN2	YDR068W	4.27429								4.27429
MSN2	YDR084C	5.25101								5.25101
MSN2	YDR101C	2.73159			1.28148					4.01307
MSN2	YDR132C	2.21225	1.02606		0.91206					4.15037
MSN2	YDR171W	2.19117	1.5458	3.16295	1.05757	0.585908			0.708334	9.25173
MSN2	YDR258C	4.41053	0.173526		1.42023		0.909648		0.614963	7.5289
MSN2	YDR293C		1.02606	2.57872	0.327179				0.67961	4.61157
MSN2	YDR353W	2.99254	0.0818447		0.578059			1.01508	0.645993	5.31352
MSN2	YDR384C			5.55775						5.55775
MSN2	YDR453C	6.4926	0.587698	0.317711	0.675583				0.684814	8.75841
MSN2	YDR456W	6.4926	0.587698							7.0803
MSN2	YDR457W	6.0281								6.0281
MSN2	YDR513W	0.38703	0.34489		0.548503			2.20432	0.723167	4.20791
MSN2	YEL011W	3.18994	0.635279	1.007	0.488408				0.713823	6.03445
MSN2	YER013W	4.68534								4.68534
MSN2	YER019C-A		1.23206	7.38487						8.61693
MSN2	YER020W		1.23206	7.84202					0.480752	9.55483
MSN2	YER044C	3.53861	0.173526	0.400883	0.805657				0.67579	5.59447
MSN2	YER044C-A	3.53861							0.650734	4.18934
MSN2	YER068W	3.70387	0.809349							4.51322
MSN2	YER115C	0.605608	1.89196	4.41643						6.914
MSN2	YER177W	3.40996	1.51051	4.22588	0.56409				0.5105	10.2209
MSN2	YER178W	3.40996	1.51051	3.51442						8.43489
MSN2	YFL014W	0.449881	1.12498	5.44819					0.719124	7.74218
MSN2	YFL016C	0.449881	0.991939	6.13994	0.81498	0.788882	0.825673			10.0113
MSN2	YFR053C	1.90263	0.809349	2.20759	0.856102				0.678155	6.45383
MSN2	YGL036W	1.34114		3.2448					0.703743	5.28968
MSN2	YGL037C	0.818493		3.13921	0.757772				0.731434	5.44691
MSN2	YGL096W	2.0837	1.51051	0.697627						4.29184
MSN2	YGL263W	3.8569			0.549478					4.40638
MSN2	YGR071C	4.29957								4.29957
MSN2	YGR072W	4.29957			0.339861					4.63943
MSN2	YGR074W	4.29957								4.29957
MSN2	YGR086C	1.55506	1.23206	1.01754	0.392395				0.45554	4.65259
MSN2	YGR087C	4.63512	0.379313	0.00176606	0.581298					5.5975
MSN2	YGR088W	4.63512	0.379313		0.749515				0.72862	6.49257
MSN2	YGR096W		1.23206	3.17231						4.40437
MSN2	YGR142W	3.84839			0.452504					4.30089
MSN2	YGR192C	4.30357	0.991939		0.620675					5.91618

TF	Target	Binding_0-2000	Motif_Score_SC	Other_Yeasts	Two-Hop_BE	Two-Hop_BP	Two-Hop_BY	Two-Hop_BG	Max_Membership	Sum
MSN2	YGR207C	5.02403			0.451637					5.47567
MSN2	YGR209C	5.02403	0.470096		1.1308				0.721574	7.3465
MSN2	YGR211W	5.02403	0.470096	0.0149201			0.880303			6.38935
MSN2	YGR243W		1.03399	2.92368					0.678131	4.6358
MSN2	YGR249W	1.98637	1.03399	1.52323						4.54359
MSN2	YGR254W	0.780997	1.5458	5.29363	0.364915	0.598349			0.580515	9.16421
MSN2	YGR256W	2.31217	0.635279	0.776288	0.375369					4.09911
MSN2	YHL036W	2.63135	1.23206	0.515075	0.38765				0.730366	5.4965
MSN2	YHR008C	3.36255	0.34489	0.309644						4.01708
MSN2	YHR088W	3.98859	0.249461	0.220529	1.00147					5.46005
MSN2	YHR138C		1.23206	7.63174	0.340247				0.644714	9.84876
MSN2	YIL057C	1.47482		4.05484	0.36966					5.89932
MSN2	YIL099W	1.77152	0.644384	2.43416					0.641495	5.49156
MSN2	YJL008C	6.34711	0.582795				0.804511			7.73442
MSN2	YJL026W	0.605967		2.06179	0.928015				0.726388	4.32216
MSN2	YJL036W		1.23206	3.00677			0.865791			5.10462
MSN2	YJL052W	1.61637	1.51051	1.25201	0.758177					5.13707
MSN2	YJL076W	1.42881		3.00559						4.4344
MSN2	YJL141C	0.619422	1.89196			1.52481			0.587627	4.62382
MSN2	YJR008W		1.02606	2.55217	0.699038				0.732323	5.00959
MSN2	YJR045C	3.53243	0.991939	0.469463	0.402277					5.39611
MSN2	YJR046W	3.53243	0.991939	0.965392					0.647066	6.13683
MSN2	YJR121W			3.85849		0.3274			0.638163	4.82405
MSN2	YKL013C		1.03399	2.44957	0.669477					4.15304
MSN2	YKL035W	2.94912	1.89196	6.41987	0.422486				0.709578	12.393
MSN2	YKL053C-A	2.37303		1.76677						4.1398
MSN2	YKL062W	2.04171	1.89196	0.162128	0.660159					4.75596
MSN2	YKL071W	3.82779			0.582047					4.40984
MSN2	YKL084W	5.83841	1.51051							7.34892
MSN2	YKL085W	5.83841	1.51051	2.39243					0.728468	10.4698
MSN2	YKL087C	5.83841	1.51051	1.16959	1.51324					10.0317
MSN2	YKL117W		1.89196	7.0472	1.38512					10.3243
MSN2	YKL119C		1.89196	4.55827						6.45023
MSN2	YKL185W			4.61434						4.61434
MSN2	YKL191W		1.03399	3.14588	0.329011					4.50888
MSN2	YKL192C		1.03399	3.06477	1.0539	0.359178	0.89279			6.40463
MSN2	YKL193C		1.03399	3.01059	1.13648				0.663827	5.84489
MSN2	YKL217W		1.51051	1.70494	0.334346				0.63515	4.18495
MSN2	YKR052C	5.35321								5.35321
MSN2	YKR054C	5.617								5.617
MSN2	YKR055W	5.617								5.617
MSN2	YKR056W	5.617			1.17038					6.78738
MSN2	YKR065C	7.02162	0.620681		0.396246					8.03855
MSN2	YKR066C	7.02162	0.620681	0.325027					0.452703	8.42003
MSN2	YKR067W	7.02162	0.620681	0.29015	0.345407				0.730448	9.00831
MSN2	YLL024C	3.40316	0.0892073	0.224239	1.38361					5.10022
MSN2	YLL026W	5.43181	1.02606	1.33258	1.2116		0.911974		0.571465	10.4855
MSN2	YLL039C	0.977673	0.809349	0.106704	0.432873			1.20169	0.693263	4.22155
MSN2	YLL049W	0.12541	1.5458	8.15998						9.83119
MSN2	YLL050C	0.12541	1.5458	3.40173	0.358807					5.43175
MSN2	YLR009W		0.470096	1.47253	1.41591		0.789689			4.14823
MSN2	YLR034C	0.447077	0.885999	1.94332					0.730642	4.00704
MSN2	YLR051C	3.15437	1.23206		0.53844					4.92487
MSN2	YLR053C	3.15437	1.23206	2.29344						6.67987
MSN2	YLR081W	0.32118	1.89196	1.77496					0.702563	4.69066
MSN2	YLR108C	9.15352	1.23206	3.41963	0.836056				0.712379	15.3536
MSN2	YLR109W	9.15352	1.23206	4.82581					0.732701	15.9441
MSN2	YLR110C	3.48144			1.02954					4.51098
MSN2	YLR113W	3.48144			0.990921					4.47236
MSN2	YLR134W		1.23206	6.70816	0.334247					8.27447
MSN2	YLR164W	2.56178	0.470096	0.588604					0.605038	4.22552
MSN2	YLR258W	2.44487	0.443139	3.06885	1.05099				0.717179	7.72503
MSN2	YLR354C	0.614368	1.02606	1.84217					0.717173	4.19977
MSN2	YLR399C	0.102734		5.18653						5.28926
MSN2	YLR423C					1.41267	0.77998	1.25815	0.572603	4.0234
MSN2	YLR437C	0.627149	1.23206	3.98124						5.84045
MSN2	YML002W	0.144602	1.89196	1.59412	0.545636					4.17632
MSN2	YML004C	0.144602	1.89196	4.01892	0.729862				0.508713	7.29406
MSN2	YML007W	3.83094	0.443139			0.340419			0.56147	5.17597
MSN2	YML008C	3.83094	0.443139		0.745944					5.02002
MSN2	YML028W	3.77567			1.15576				0.644274	5.5757
MSN2	YML041C	0.465774	1.03399	3.02267						4.52243
MSN2	YML100W	4.04021	1.89196	4.91854	1.17654				0.71945	12.7467
MSN2	YML101C	4.04021	1.89196	5.4714						11.4036
MSN2	YMR012W	4.02911					0.885248			4.91436
MSN2	YMR089C	1.63489	0.635279			1.73081				4.00098
MSN2	YMR090W	1.63489	0.635279	0.114186	1.03344				0.72016	4.13795
MSN2	YMR105C	0.366297	1.23206	2.11772	1.15517				0.692583	5.56383
MSN2	YMR118C	1.07899		3.276						4.35499
MSN2	YMR119W	1.07899		2.618	1.11054					4.80753
MSN2	YMR173W	6.24307	1.03399	0.254168	0.493472				0.732196	8.7569
MSN2	YMR175W	1.28996	1.89196	0.563438					0.573712	4.31907
MSN2	YMR227C	4.26856	0.249461							4.51802
MSN2	YMR228W	4.26856	0.809349							5.07791
MSN2	YMR251W-A	1.75707	0.582795	4.88649	0.648111				0.672912	8.54738
MSN2	YMR315W	3.04712			0.549744				0.719353	4.31622
MSN2	YNL001W	4.33386					0.812314			5.14617
MSN2	YNL002C	4.33386			1.4325					5.76636
MSN2	YNL003C	4.33386								4.33386
MSN2	YNL007C	3.48635			1.28721	1.49199	0.782765			7.04831
MSN2	YNL083W	3.42256					0.819517			4.24208
MSN2	YNL098C	0.734704	0.628593	4.25349						5.61679
MSN2	YNL132W	3.40435	1.5458		0.588873					5.53902
MSN2	YNL134C	3.40435	1.5458	0.126428	1.44501				0.730175	7.25176
MSN2	YNL135C	3.40435	1.5458		0.871716					5.82187
MSN2	YNL160W	3.48643	1.02606	3.65432					0.698588	8.8654
MSN2	YNL200C		1.23206	3.57806	0.575413				0.721855	6.10739
MSN2	YNL241C	1.48538	0.0892073	2.72362					0.719942	5.01815
MSN2	YNR001C	0.409968	0.635279	0.193736	0.86131	1.41139			0.676201	4.18788
MSN2	YOL052C-A	4.09876	0.841657	3.10539	0.859077					8.90488
MSN2	YOL086W-A		1.89196	9.0353						10.9273
MSN2	YOL087C		1.89196	5.38665						7.27861
MSN2	YOL113W	3.27474	0.635279	0.861912						4.77193
MSN2	YOL114C	3.27474	0.635279	1.22887						5.13889
MSN2	YOL159C	5.06999								5.06999
MSN2	YOR017W	1.92269	1.5458	5.01099						8.47948
MSN2	YOR019W	0.678637	1.03399	2.03805	0.430451					4.18113
MSN2	YOR052C	0.0717344	0.991939	2.21329	0.511266				0.606264	4.39449
MSN2	YOR157C	1.81056	0.885999	0.31918	0.670428		0.972305			4.65847
MSN2	YOR173W		0.258558	10.8524					0.714225	11.8252

TF	Target	Binding_0-2000	Motif_Score_SC	Other_Yeasts	Two-Hop_BE	Two-Hop_BP	Two-Hop_BY	Two-Hop_BG	Max_Membership	Sum
MSN2	YPL054W		1.89196	2.08982					0.646348	4.62813
MSN2	YPL055C		1.89196	3.53087						5.42283
MSN2	YPL154C		1.89196	13.7613	0.865356				0.732657	17.2513
MSN2	YPL177C	1.33602	1.89196				0.819517			4.0475
MSN2	YPL230W	0.583285	1.23206	6.51399	0.502089				0.640081	9.47151
MSN2	YPL239W	1.12654	0.210966	2.37063	0.830551					4.53869
MSN2	YPL240C	1.12654	0.210966	2.5541	1.15223	1.49199			0.693941	7.22977
MSN2	YPL253C	0.312649	0.249461	5.53087						6.09298
MSN2	YPR120C	4.76798								4.76798
MSN2	YPR124W	3.77911			0.329628					4.10874
MSN4	SNR18	4.65694								4.65694
MSN4	TC(GCA)P2	4.19967								4.19967
MSN4	TF(GAA)N	3.95393	0.229268							4.1832
MSN4	TL(UAA)B1	4.29906								4.29906
MSN4	TM(CAU)J2	6.69601	0.582795							7.27881
MSN4	TT(AGU)B	4.20923								4.20923
MSN4	TT(AGU)C	5.87748								5.87748
MSN4	TW(CCA)J	7.62626								7.62626
MSN4	TW(CCA)K	5.4152	0.34489							5.76009
MSN4	YAL003W	4.65694					0.832464			5.4894
MSN4	YAL005C	4.65694			0.97074	0.74134				6.36902
MSN4	YAL016W		0.809349	3.66614	0.540066					5.01555
MSN4	YBL032W		0.809349	3.06745			0.881096			4.7579
MSN4	YBL033C			4.48767						4.48767
MSN4	YBL105C			2.21336	1.15185			0.903344		4.26855
MSN4	YBL108C-A	4.29906								4.29906
MSN4	YBR013C	4.20923			0.759212					4.96844
MSN4	YBR026C		0.809349	2.4893					0.731847	4.0305
MSN4	YBR072W	2.92106	0.99821	1.74205	0.600036				0.646221	6.90758
MSN4	YCL030C		0.229268	4.32119						4.55046
MSN4	YCL040W		0.99821	2.58479	1.38671				0.723255	5.69297
MSN4	YCL042W		0.809349	1.69743	1.00817				0.725481	4.24043
MSN4	YCL052C		0.99821	5.64277						6.64098
MSN4	YCL067C	5.02533			0.969309		0.85516			6.8498
MSN4	YCR096C	5.87748			0.795237	1.42194	0.85516			8.94982
MSN4	YCR097W	5.87748				1.42194	0.85516			8.15458
MSN4	YDL022W	0.622469	0.582795	2.30649	1.14129				0.732085	5.38513
MSN4	YDL101C	0.23056	0.809349	3.00615				0.905028		4.95109
MSN4	YDL180W	4.1748								4.1748
MSN4	YDL182W	4.23842			0.417101					4.65552
MSN4	YDL183C	4.23842								4.23842
MSN4	YDR011W	5.20007								5.20007
MSN4	YDR042C	5.41947								5.41947
MSN4	YDR068W	3.44307					0.845017			4.28809
MSN4	YDR074W	0.690808		2.39732	1.34858	0.444751			0.715536	5.597
MSN4	YDR084C	6.23909								6.23909
MSN4	YDR101C	3.08146			1.18544					4.2669
MSN4	YDR171W	1.43747	0.809349	3.82269	0.877106	0.550808			0.727755	8.22518
MSN4	YDR225W	0.810745		4.48189	0.492207					5.78484
MSN4	YDR258C	3.48838	0.173526		1.23976		0.950612		0.721902	6.57418
MSN4	YDR306C	0.817749	0.229268	2.9834						4.03042
MSN4	YDR309C	0.457089	0.809349	3.66375						4.93019
MSN4	YDR353W	3.19897			0.397595			1.16591	0.645993	5.40847
MSN4	YDR354W	4.18566	0.582795							4.76846
MSN4	YDR356W	4.18566								4.18566
MSN4	YDR391C	0.297298	0.443139	2.88821	0.573084				0.730495	4.93223
MSN4	YDR392W	2.8412	0.443139	1.8321						5.11644
MSN4	YDR453C	7.11298	0.582795							7.69578
MSN4	YDR456W	7.11298	0.582795							7.69578
MSN4	YER013W	4.32851								4.32851
MSN4	YER020W		0.379313	3.69705						4.07636
MSN4	YER067W	0.176213	0.809349	6.21556	0.625726				0.732754	8.5596
MSN4	YER068W	3.67171	0.809349							4.48106
MSN4	YER094C		0.229268	2.50447	0.621962	0.462437	0.852458			4.67059
MSN4	YER095W		0.229268	5.08756		0.462945	0.954372			6.73415
MSN4	YER103W	5.35182	0.201138		0.828796				0.730031	7.11179
MSN4	YER163C	1.16287		2.22104					0.725135	4.10905
MSN4	YER190W	2.72657			0.7067			1.05042		4.48369
MSN4	YFL014W	0.29002	0.443139	3.08763	0.815917					4.63671
MSN4	YFR015C		0.809349	3.4625					0.732748	5.0046
MSN4	YFR017C	0.299626	0.99821	1.78599	0.593234				0.718253	4.39531
MSN4	YFR053C	0.78255	0.809349	2.39582	0.631832				0.724466	5.34402
MSN4	YGL200C		0.229268	2.64929		0.562478	0.844183			4.28522
MSN4	YGL263W	5.07378			0.582609					5.65639
MSN4	YGR008C	1.56472	0.312187	0.857612	0.880833				0.730372	4.34572
MSN4	YGR040W	2.78797				0.66714		0.903346		4.35846
MSN4	YGR052W	2.80963		0.349351	0.519583			0.905028	0.728157	5.31175
MSN4	YGR071C	6.84354								6.84354
MSN4	YGR072W	6.84354			0.763016					7.60656
MSN4	YGR074W	6.84354								6.84354
MSN4	YGR087C	4.79668	0.379313							5.17599
MSN4	YGR088W	4.79668	0.379313	2.28709	0.555756				0.731604	8.75044
MSN4	YGR089W	4.23246								4.23246
MSN4	YGR195W	1.22934	0.582795	1.59118			1.01004			4.41336
MSN4	YGR207C	5.86102								5.86102
MSN4	YGR209C	5.86102		3.55918	0.750888					10.1711
MSN4	YGR211W	5.86102					0.921267			6.78229
MSN4	YGR254W	1.66562	0.809349	2.24387						4.71884
MSN4	YHL038C	2.96901	0.644384	0.746408						4.3598
MSN4	YHR008C	2.19766	0.34489	1.99345						4.536
MSN4	YHR087W	6.06011			0.734451				0.714756	7.50932
MSN4	YHR088W	6.06011		1.31579	1.07433					8.45023
MSN4	YIL051C	2.45064	0.644384	1.97725	0.362534					5.43481
MSN4	YJL005W		0.229268	4.12214						4.35141
MSN4	YJL008C	6.69601	0.582795				0.823234			8.10204
MSN4	YJL052W	1.14006	0.99821	1.24792	1.10676					4.49295
MSN4	YJL078C	0.803534		3.65692				1.66694		6.12739
MSN4	YJL190C	2.58842			1.00536		1.02221			4.61599
MSN4	YKL042W		0.644384	2.50544		0.965221				4.11505
MSN4	YKL084W	4.14933		0.150299						4.29963
MSN4	YKL085W	4.14933		1.10377					0.70988	5.96298
MSN4	YKL087C	4.14933		0.312321	1.05966					5.52131
MSN4	YKR014C		0.229268	3.88334						4.11261
MSN4	YKR052C	3.50058		0.546146	0.368104					4.41483
MSN4	YKR056W	3.06124			1.24324					4.30448
MSN4	YKR065C	7.00902	0.443139							7.45216
MSN4	YKR066C	7.00902	0.443139	5.10898					0.578961	13.1401
MSN4	YKR067W	7.00902	0.443139	4.41272	0.516382				0.731575	13.1128
MSN4	YLL024C	2.63166			1.08128				0.670155	4.38309
MSN4	YLL026W	4.54837	0.379313	2.7171	1.02323		0.952938		0.731132	10.3521

TF	Target	Binding_0-2000	Motif_Score_SC	Other_Yeasts	Two-Hop_BE	Two-Hop_BP	Two-Hop_BY	Two-Hop_BG	Max_Membership	Sum
MSN4	YLR058C			4.02344						4.02344
MSN4	YLR108C	6.93305			0.491021					7.42407
MSN4	YLR109W	6.93305		0.00238954	0.458235					7.39367
MSN4	YLR110C	1.20091		1.66766	1.02697			0.9116		4.80714
MSN4	YLR163C	4.0577		0.366779						4.42448
MSN4	YLR205C			3.23334	0.468586				0.670809	4.37274
MSN4	YLR258W	0.674303	0.443139	1.74509	1.16763				0.731704	4.76187
MSN4	YLR438C-A	3.38496	0.229268	1.48443			0.927298			6.02596
MSN4	YML028W	2.73547		0.138565	0.770406				0.70599	4.35043
MSN4	YML100W	2.25766	0.229268	3.48043	1.30327				0.730709	8.00134
MSN4	YML101C	2.25766	0.809349	2.08822						5.15523
MSN4	YML126C	2.98767			0.406609		0.927368			4.32165
MSN4	YML131W	3.35271			0.86173				0.732622	4.94706
MSN4	YMR052W	4.52682	0.34489							4.87171
MSN4	YMR105C	1.70075	0.809349	0.58404	1.18817				0.726338	5.00865
MSN4	YMR165C	4.84958		0.053443						4.90302
MSN4	YMR169C	1.01647	0.644384	1.58211	0.611181				0.703053	4.5572
MSN4	YMR173W	2.74292	0.379313	1.07677	0.499984					4.69899
MSN4	YMR174C	0.664274	0.809349	3.65471						5.12833
MSN4	YMR175W	0.664274	0.809349	3.37151						4.84513
MSN4	YMR227C	4.07258								4.07258
MSN4	YMR228W	4.07258	0.809349							4.88193
MSN4	YMR251W-A	1.96091	0.582795	1.46233	0.462977				0.622878	5.09189
MSN4	YMR262W		0.443139	4.7326					0.714808	5.89055
MSN4	YMR313C	3.2641		0.844124						4.10822
MSN4	YMR315W	3.2641		1.00439	1.34039				0.731032	6.33991
MSN4	YNL001W	4.69054					0.910583			5.60112
MSN4	YNL002C	4.69054			1.3541		0.790464			6.8351
MSN4	YNL003C	4.69054								4.69054
MSN4	YNL006W	3.35633		1.20126					0.710288	5.26788
MSN4	YNL007C	3.35633		0.521138	1.10725	1.09245			0.720858	6.79803
MSN4	YNL008C	3.35633		0.560086	0.351304					4.26772
MSN4	YNL083W	3.59476					0.898553			4.49331
MSN4	YNL098C		0.443139	4.89566						5.3388
MSN4	YNL132W	3.95393	0.229268		0.618115					4.80131
MSN4	YNL134C	3.95393	0.229268	0.326825	0.991423				0.691841	6.19329
MSN4	YNL135C	3.95393	0.229268		1.04269					5.22589
MSN4	YNL194C	1.63688	0.99821	1.53135	0.990167				0.731011	5.88762
MSN4	YNL336W	7.41655			0.492147					7.9087
MSN4	YNL339C	7.41655			0.554832					7.97138
MSN4	YNR047W		0.809349	2.52427				0.905028		4.23865
MSN4	YOL052C-A	3.08122	0.201138	0.0353396	0.892081					4.20978
MSN4	YOL099C			4.34166						4.34166
MSN4	YOL159C	4.43518								4.43518
MSN4	YOR017W	1.3794	0.809349	4.79833						6.98708
MSN4	YOR134W	1.19405	0.229268	1.81365	0.763666				0.716222	4.71686
MSN4	YOR178C		0.99821	2.4645					0.711611	4.17432
MSN4	YOR224C	3.01424	0.809349	1.97213	0.4726					6.26832
MSN4	YOR226C	3.01424	0.809349	1.06979						4.89338
MSN4	YOR234C	4.29217			0.657328					4.9495
MSN4	YOR236W	4.29217								4.29217
MSN4	YOR237W	4.29217	0.173526							4.4657
MSN4	YOR386W		0.229268	3.09019					0.731466	4.05092
MSN4	YPL014W	0.38127	0.582795	3.16586					0.482515	4.61244
MSN4	YPL247C		0.443139	2.25323			0.897598		0.732727	4.32669
MSN4	YPL263C		0.34489	3.92912	0.368522			0.905798		5.54833
MSN4	YPR074C		0.99821	3.1424	0.397425					4.53803
MSN4	YPR102C	2.32318		1.5081	0.902175					4.73345
MSN4	YPR120C	4.19967								4.19967
MSN4	YPR187W	2.32504			1.01795		0.921491			4.26448
MSN4	YPR198W	3.60053			1.06132					4.66185
MSN4	YPR202W	3.32436			1.39077			1.15813		5.87326
MSN4	YPR203W	3.32436			1.33535			1.15813		5.81784
MSS11	YCR107W	4.24878								4.24878
NDD1	SNR128	6.52042								6.52042
NDD1	SNR190	6.52042								6.52042
NDD1	SNR3	4.04812								4.04812
NDD1	YAL029C	0.185952				0.412221	1.94135	2.5112	0.697086	5.74781
NDD1	YAL035W			6.00224	0.787813				0.677709	7.46776
NDD1	YAL040C	0.267553	1.32479	3.63533			1.87141		0.536372	7.63546
NDD1	YAR002C-A				0.901171	2.92563	1.94458			5.77138
NDD1	YAR018C	15.8551	1.1222	6.38731					0.440601	23.8052
NDD1	YAR019C				2.40872	1.22213	1.98861			5.61946
NDD1	YBL003C				1.96597		1.47117		0.641607	4.07875
NDD1	YBL008W				2.24612		2.09954			4.34566
NDD1	YBL038W					3.9249	1.19148			5.11638
NDD1	YBL061C				2.47607		2.04667			4.52274
NDD1	YBL067C		0.510604	0.112876			1.94048	1.58302		4.14698
NDD1	YBL089W				2.45302	2.92563	2.00458			7.38323
NDD1	YBR010W		0.930574	0.198425	1.8671		1.94091			4.93701
NDD1	YBR020W		0.212276			4.11544				4.32772
NDD1	YBR024W					4.11544				4.11544
NDD1	YBR031W			0.290959	2.93096		0.977664			4.19958
NDD1	YBR036C	15.8551								15.8551
NDD1	YBR037C	15.8551								15.8551
NDD1	YBR038W	15.8551			2.75386					18.609
NDD1	YBR044C	1.3378			2.79701					4.13481
NDD1	YBR054W				1.18511	3.2218				4.40691
NDD1	YBR066C		0.996501			3.2218	1.916		0.662908	6.79721
NDD1	YBR067C	0.906899	0.906304	2.23536						4.04856
NDD1	YBR077C	4.30797	0.870868							5.17884
NDD1	YBR078W	4.30797	0.288988	0.307051	1.3919					6.29591
NDD1	YBR081C	2.84295				3.33393	2.01033			8.18721
NDD1	YBR088C				2.74628		1.16954		0.726537	4.64236
NDD1	YBR092C	1.85997	1.2096	2.6277	1.14841				0.701164	7.54684
NDD1	YBR093C		1.2096	3.57084					0.696367	5.47681
NDD1	YBR138C	9.84376								9.84376
NDD1	YBR139W	9.84376					1.94135		0.598631	12.3837
NDD1	YBR158W	1.21525			2.6244		1.94048			5.78013
NDD1	YBR160W		0.164953			4.11544				4.28039
NDD1	YBR162C	0.319494			0.879923			3.31271		4.51213
NDD1	YBR175W						2.05111	3.00838	0.63591	5.6954
NDD1	YBR188C	1.06072		3.22045						4.28117
NDD1	YBR191W	1.06072			1.1512		1.14712		0.711752	4.07079
NDD1	YBR195C						2.10179	2.86994		4.97173
NDD1	YBR202W			0.0673007	2.82359		1.17171		0.69011	4.75271
NDD1	YBR274W			2.30587	2.756					5.06187
NDD1	YBR291C				2.33976		1.94048			4.28024
NDD1	YCR024C	4.46616	0.0838537							4.55001
NDD1	YCR024C-A	4.46616	0.0838537		1.28261	1.39419			0.678566	7.90538

TF	Target	Binding_0-2000	Motif_Score_SC	Other_Yeasts	Two-Hop_BE	Two-Hop_BP	Two-Hop_BY	Two-Hop_BG	Max_Membership	Sum
NDD1	YCR024C-B	4.46616	0.0838537							4.55001
NDD1	YCR027C						2.04834	2.03852		4.08686
NDD1	YCR032W	0.504208			0.834793		2.02155	1.80312		5.16367
NDD1	YCR065W	3.39672	0.452558		0.710297				0.560804	5.12038
NDD1	YDL003W				2.25438	1.46926			0.725901	4.44954
NDD1	YDL004W				2.66692		2.02438			4.6913
NDD1	YDL010W		0.2631		2.47578		2.08593		0.709014	5.53382
NDD1	YDL020C		0.872616	0.616848	2.56176					4.05122
NDD1	YDL022W	2.66037			1.98809					4.64846
NDD1	YDL106C					4.11544	1.96053			6.07597
NDD1	YDL141W		0.687883	1.66512			2.0739		0.629942	5.05685
NDD1	YDL192W			0.83252	2.55984		2.02155			5.41391
NDD1	YDL239C		0.338493			1.44608	1.18894	1.70535		4.67886
NDD1	YDR009W			0.0930768		4.11544				4.20852
NDD1	YDR011W	0.273251	1.35109	3.2448						4.86914
NDD1	YDR033W	4.29989			1.08898					5.38887
NDD1	YDR077W		0.404676	3.17288	2.52315					6.10071
NDD1	YDR107C			0.243056	2.52721		1.94048			4.71075
NDD1	YDR113C					4.11544				4.11544
NDD1	YDR128W						2.09062	2.8624		4.95302
NDD1	YDR146C	15.8551	0.966785	3.62366	3.0647		2.01033		0.725461	26.246
NDD1	YDR147W	15.8551	0.966785	4.21955			2.0112			23.0526
NDD1	YDR148C	8.44137								8.44137
NDD1	YDR150W	8.44137								8.44137
NDD1	YDR165W		0.030037		1.38376		1.99744		0.725035	4.13627
NDD1	YDR189W	3.07678					2.04834		0.547975	5.67309
NDD1	YDR190C		0.888904	2.21539	1.00772		1.14528		0.722251	5.97954
NDD1	YDR212W					3.33393			0.692753	4.02668
NDD1	YDR253C			0.259278			2.04336	3.39415		5.69679
NDD1	YDR267C			0.0231008		4.11544	2.05968			6.19822
NDD1	YDR313C					2.92563	1.94458			4.87021
NDD1	YDR324C	1.66176		0.0875695	1.12757		2.04362		0.623007	5.54353
NDD1	YDR335W		0.337212			3.67116				4.00837
NDD1	YDR358W		0.730454		1.87178		1.94135	1.38417		5.92775
NDD1	YDR373W						2.05716	3.30935		5.36651
NDD1	YDR378C		0.986232	2.41212		2.01987				5.41822
NDD1	YDR389W		0.728662	0.522713		3.7382				4.98958
NDD1	YDR412W		0.346169	0.858669	1.63591	3.67116			0.496688	7.0086
NDD1	YDR448W					0.602753	2.00458	2.03179		4.63912
NDD1	YDR451C	12.3234	0.672817	6.6413	2.23285		1.95849			23.8289
NDD1	YDR452W	12.3234	0.672817	3.5915						16.5877
NDD1	YDR480W					2.22979	1.87141			4.1012
NDD1	YDR492W				2.55268	2.92563			0.625709	6.10402
NDD1	YDR524C	8.21229	1.32963				1.94048			11.4824
NDD1	YDR524C-B	8.21229	1.32963							9.54192
NDD1	YDR525W-A	8.21229	1.32963	7.49606						17.038
NDD1	YDR527W	8.21229	1.32963	7.60057	0.789124				0.601383	18.533
NDD1	YEL004W	4.21919								4.21919
NDD1	YEL007W	10.1066								10.1066
NDD1	YEL013W		0.179455			3.7382	1.95069			5.86834
NDD1	YEL032W		1.35697	3.141			1.14251			5.64048
NDD1	YEL034W				3.37066		1.12617		0.705969	5.2028
NDD1	YEL040W		0.84517	3.88797					0.729779	5.46292
NDD1	YEL044W	0.669489	0.580342	5.26234						6.51217
NDD1	YEL046C	0.669489	0.580342	5.60841						6.85824
NDD1	YEL066W		1.36819	3.0346						4.40279
NDD1	YEL068C		1.36819	4.25233						5.62052
NDD1	YEL075C				0.421236	3.2218		0.950139		4.59317
NDD1	YER015W				1.05583	3.33393				4.38976
NDD1	YER032W	0.261208			2.57525	4.11544				6.9519
NDD1	YER036C		0.0408413	0.0752491		3.2218			0.688728	4.02662
NDD1	YER053C				2.3563		1.94048			4.29678
NDD1	YER059W		0.610318			3.67116				4.28148
NDD1	YER066W	2.44652			0.400198		2.07529	2.94189		7.8639
NDD1	YER078C	11.7196								11.7196
NDD1	YER079W	11.7196	0.209002							11.9286
NDD1	YER080W	11.7196	0.209002							11.9286
NDD1	YER081W			0.304283	2.61948	1.15859				4.08235
NDD1	YER082C				2.83175		2.10179			4.93354
NDD1	YER111C		1.32479	3.5648		4.11544	1.24058			10.2456
NDD1	YER112W		1.32479	3.55852		2.01987	2.00592			8.9091
NDD1	YER125W		0.420471			2.92563	2.00261			5.34871
NDD1	YER133W					3.67116	1.08798			4.75914
NDD1	YER169W						1.94048	2.91857		4.85905
NDD1	YER172C				2.25337	2.01987	1.02187			5.29511
NDD1	YFL005W						1.99467	2.03785		4.03252
NDD1	YFL026W	0.0630025	1.47029	11.3802	0.743416					13.6569
NDD1	YFL027C	0.0630025	1.47029	12.6913						14.2246
NDD1	YFR017C	6.95711								6.95711
NDD1	YFR019W	6.95711					1.20855			8.16566
NDD1	YFR031C				1.41593		1.0179	2.01332		4.44715
NDD1	YFR045W			3.58526			2.03342			5.61868
NDD1	YGL003C				1.01518	4.11544	2.02093			7.15155
NDD1	YGL006W-A	6.21781	0.378677							6.59649
NDD1	YGL007C-A	6.21781	0.916718							7.13453
NDD1	YGL008C	5.77471	0.916718	4.78663	2.78131	1.04094			0.597854	15.8982
NDD1	YGL014W				2.44548		2.04834		0.701927	5.19575
NDD1	YGL021W	2.39203	0.348086	0.659327	3.12775		1.93395		0.732698	9.19384
NDD1	YGL086W		0.426147			4.11544	1.20272			5.74431
NDD1	YGL097W	0.678587			1.40051		2.02662		0.630283	4.736
NDD1	YGL106W						2.04834	3.41613		5.46447
NDD1	YGL110C	0.520193			2.23424		2.04834			4.80277
NDD1	YGL114W	4.21987		0.956417	0.545048					5.72133
NDD1	YGL116W	15.8551	0.474473	0.325014	2.60887	4.11544	2.0112			25.3901
NDD1	YGL135W	1.69605			0.880756		1.01306		0.715457	4.30532
NDD1	YGL225W					2.92563	1.98443			4.91006
NDD1	YGL258W		1.32479	4.0343						5.35909
NDD1	YGR049W		1.11271			3.2218			0.729189	5.0637
NDD1	YGR060W	2.31168	0.119228				2.02532			4.45623
NDD1	YGR086C		0.82993	3.84966						4.67959
NDD1	YGR092W	5.13732	0.501282		2.81242				0.64445	9.09547
NDD1	YGR108W			0.496579	2.2484		1.94048		0.662002	5.34746
NDD1	YGR109C	0.868209			1.56688		1.94048			4.37557
NDD1	YGR146C		1.18208	10.7185						11.9006
NDD1	YGR152C						2.05126	2.03848	0.661664	4.7514
NDD1	YGR166W		0.786328			3.2218				4.00813
NDD1	YGR172C		0.609098	0.739157		3.2218			0.716924	5.28698
NDD1	YGR185C			0.0785239	0.590489	1.70374	1.11096		0.67325	4.15696
NDD1	YGR189C		1.41285	17.3658	3.09809					21.8767
NDD1	YGR192C	1.67893	0.193919		2.97146		1.10224			5.94655

TF	Target	Binding_0-2000	Motif_Score_SC	Other_Yeasts	Two-Hop_BE	Two-Hop_BP	Two-Hop_BY	Two-Hop_BG	Max_Membership	Sum
NDD1	YGR218W					4.11544				4.11544
NDD1	YGR220C					3.9249	1.16137			5.08627
NDD1	YGR229C	6.92136	0.0117579	0.0529859	1.05283					8.03893
NDD1	YGR230W	6.92136	0.0117579	0.0565373						6.98966
NDD1	YGR279C	0.931141	0.698706	5.42491	1.22619				0.676219	8.95717
NDD1	YGR285C			0.665567	0.646399	3.9249			0.60133	5.8382
NDD1	YHL008C		0.693898	4.18258						4.87648
NDD1	YHL025W	4.12755		0.0291362						4.15669
NDD1	YHL026C			0.0311965	0.797343	3.2218			0.730559	4.7809
NDD1	YHL028W	6.51456	0.310257		2.68747					9.51229
NDD1	YHL029C	6.51456	0.310257	0.459472						7.28429
NDD1	YHR023W	0.433928	0.288988		3.1429				0.73036	4.59618
NDD1	YHR026W		1.12777			3.44746	1.89025		0.713312	7.17879
NDD1	YHR049W					3.2218	1.88282			5.10462
NDD1	YHR057C					3.67116	1.09256			4.76372
NDD1	YHR064C					3.9249			0.625724	4.55062
NDD1	YHR073W					4.11544				4.11544
NDD1	YHR098C	2.12776	0.880184	1.84779					0.723879	5.57961
NDD1	YHR099W	2.12776	0.880184	0.448552		3.33393	1.89218			8.68261
NDD1	YHR103W				2.74608		1.47422			4.2203
NDD1	YHR128W				0.642353	4.11544			0.723885	5.48168
NDD1	YHR129C					2.92563	1.94048			4.86611
NDD1	YHR148W				2.57083		1.10333		0.691626	4.36579
NDD1	YHR151C	15.8551	1.06561	9.21072						26.1314
NDD1	YHR152W	15.8551	1.06561	9.17244	0.623049					26.7162
NDD1	YHR166C					4.11544	1.88471			6.00015
NDD1	YIL035C		0.924805		2.46452		1.88612			5.27544
NDD1	YIL046W					2.04836	1.99913			4.04749
NDD1	YIL061C					3.67116	2.01409			5.68525
NDD1	YIL063C					2.92563	1.97063			4.89626
NDD1	YIL096C		0.107905		1.12427	3.2218			0.668283	5.12226
NDD1	YIL106W		0.015825	1.76846	2.56999	1.22213			0.718663	6.29507
NDD1	YIL107C		0.855113	1.75363	2.74054					5.34928
NDD1	YIL122W		1.06054	3.70448						4.76502
NDD1	YIL123W	1.32064	1.02096	2.28019	2.23652				0.679809	7.53812
NDD1	YIL125W					2.11407	0.791561	1.95419		4.85982
NDD1	YIL133C		0.411		2.4929		1.20292		0.690858	4.79768
NDD1	YIL137C				2.44003	2.92563				5.36566
NDD1	YIL158W	15.8551			2.26555					18.1207
NDD1	YIL169C	1.42018		0.269508	2.46358					4.15327
NDD1	YIR012W				2.48855		2.03222		0.729823	5.25059
NDD1	YJL005W	0.899375				0.440619	0.988487	2.33545		4.66393
NDD1	YJL013C		0.231878			4.11544				4.34732
NDD1	YJL014W					3.33393			0.712104	4.04603
NDD1	YJL030W					4.11544				4.11544
NDD1	YJL047C					2.92563	2.07681			5.00244
NDD1	YJL051W	12.2182	0.948421	0.254168	2.70995		1.41954			17.5503
NDD1	YJL052C-A	12.2182	0.948421							13.1666
NDD1	YJL069C		0.163633	0.172416	2.65261		1.94197		0.686513	5.61714
NDD1	YJL076W	0.48287		2.03952			2.00458		0.722416	5.24939
NDD1	YJL079C	8.47924	0.876111						0.658138	10.0135
NDD1	YJL080C	1.79715	0.876111				2.04815		0.728858	5.45027
NDD1	YJL081C					3.33393	1.94048			5.27441
NDD1	YJL083W		1.18208	3.78058						4.96266
NDD1	YJL100W	5.26367								5.26367
NDD1	YJL101C	5.26367								5.26367
NDD1	YJL110C			1.02198	2.46087		1.87141			5.35426
NDD1	YJL138C			2.43929	0.92158			0.950139		4.31101
NDD1	YJL148W	6.52042			0.665285				0.7273	7.913
NDD1	YJL157C				2.27921	2.22979				4.509
NDD1	YJL158C	8.50131	1.38738	1.59943	2.51806				0.595078	14.6013
NDD1	YJL159W		0.971939	5.70138	1.2827					7.95602
NDD1	YJL168C		0.613978		2.26742	4.11544	1.94271			8.93955
NDD1	YJL176C				2.23491		1.97476		0.558583	4.76825
NDD1	YJL187C	0.372942			0.850806	3.33393	1.92076			6.47844
NDD1	YJL190C	0.0692828			2.49112		1.19868		0.71737	4.47645
NDD1	YJL194W	0.713869	0.663154	4.52193			1.88874			7.78769
NDD1	YJL196C	0.713869	0.663154	4.54758	1.44253					7.36713
NDD1	YJL197W				1.59977		1.94048	1.58462		5.12487
NDD1	YJR009C				0.63419	3.67116				4.30535
NDD1	YJR050W					3.2218	2.01565			5.23745
NDD1	YJR063W			0.332289	2.52985		1.196		0.715953	4.77409
NDD1	YJR064W					3.33393			0.710842	4.04477
NDD1	YJR090C		0.95474			2.22979	1.94135			5.12588
NDD1	YJR091C	15.8551	1.38631	0.762777		1.33615	1.94048			21.2808
NDD1	YJR092W		1.38631	0.765422	2.7276		1.94672		0.722847	7.5489
NDD1	YJR119C					3.2218	1.94048	1.26948		6.43176
NDD1	YJR127C	4.04812		0.0178048						4.06592
NDD1	YJR148W	1.37666	0.15901			4.11544				5.65111
NDD1	YJR149W	1.37666	1.52403		1.69805					4.59874
NDD1	YKL002W					3.44746	1.96434			5.4118
NDD1	YKL021C		0.283174		1.58385		2.03995		0.695625	4.6026
NDD1	YKL034W				2.56421		2.0388			4.60301
NDD1	YKL096W-A	6.10473								6.10473
NDD1	YKL101W		0.889485			4.11544				5.00493
NDD1	YKL104C		1.34519	5.88056					0.700354	7.9261
NDD1	YKL113C		0.676437	0.229225	1.17605	3.44746	1.1228		0.721773	7.37374
NDD1	YKL121W				2.69893		2.07348			4.77241
NDD1	YKL129C		0.209002		2.35534			2.5112		5.07554
NDD1	YKL144C				2.89766	3.2218	1.1793		0.691823	7.99058
NDD1	YKL146W			1.13203	0.468223	2.92563	1.95069			6.47657
NDD1	YKL150W	2.11359			1.61817		2.07297			5.80473
NDD1	YKL151C	2.11359		3.36279						5.47638
NDD1	YKL163W		1.11774	3.39096						4.5087
NDD1	YKL190W						2.06905	3.41613		5.48518
NDD1	YKL193C				1.82376	0.612688	2.02925			4.4657
NDD1	YKL208W	0.275051	0.545889	3.47617						4.29711
NDD1	YKL213C				1.98261		2.04638			4.02899
NDD1	YKR001C				2.24483		1.94048		0.703018	4.88833
NDD1	YKR036C				0.98701	1.22213	2.00634	2.75731		6.97279
NDD1	YKR042W	15.8551	1.04022	1.59913	1.35067				0.730131	20.5753
NDD1	YKR043C	2.7095					1.2024		0.636966	4.54887
NDD1	YKR048C					4.11544	1.99648			6.11192
NDD1	YKR099W				2.49501	1.25944	2.01432		0.516804	6.28557
NDD1	YKR100C		0.860953	1.79775	1.53353					4.19223
NDD1	YLL013C			2.2406			1.94048			4.18108
NDD1	YLL039C				0.941856	4.11544	1.94048			6.99778
NDD1	YLR016C					4.11544				4.11544
NDD1	YLR056W	0.348251			2.5566		1.97144		0.715055	5.59135
NDD1	YLR084C	10.8331	0.749537	5.87018	2.60951		1.58216		0.681804	22.3263

TF	Target	Binding_0-2000	Motif_Score_SC	Other_Yeasts	Two-Hop_BE	Two-Hop_BP	Two-Hop_BY	Two-Hop_BG	Max_Membership	Sum
NDD1	YLR086W	10.8331	0.749537							11.5826
NDD1	YLR105C	3.71484	0.314759							4.0296
NDD1	YLR120C		0.568062		2.04307		2.00502			4.61615
NDD1	YLR131C	15.8551	0.677643		2.81173		1.88402			21.2285
NDD1	YLR132C					4.11544				4.11544
NDD1	YLR133W	15.8551	0.677643							16.5327
NDD1	YLR189C	15.8551	1.36445	3.14881						20.3684
NDD1	YLR190W	15.8551	1.36445	3.03323	3.0647				0.650114	23.9676
NDD1	YLR196W		0.571749	0.718022	1.6409		2.09126		0.67719	5.69912
NDD1	YLR210W				2.582		2.02155			4.60355
NDD1	YLR254C	0.484168			2.53909		1.14867	2.03939		6.21132
NDD1	YLR259C				2.63993			1.80464		4.44457
NDD1	YLR288C					4.11544				4.11544
NDD1	YLR300W	3.47536							0.690512	4.16587
NDD1	YLR344W	1.00507			1.27262		1.13616		0.730782	4.14463
NDD1	YLR367W	3.36844			0.539129		1.19868		0.64435	5.7506
NDD1	YLR373C		1.47815	3.0822						4.56035
NDD1	YLR374C		1.47815	5.61251						7.09066
NDD1	YLR378C	2.08213	0.591372		2.76976		1.1779		0.599746	7.22091
NDD1	YLR380W	2.08213					1.94048			4.02261
NDD1	YLR397C	0.141231	0.731649			3.2218			0.563168	4.65785
NDD1	YLR409C			0.709956	2.44673		2.09062		0.714257	5.96156
NDD1	YLR414C		1.38631	11.7207						13.107
NDD1	YLR417W		1.38631	9.43724						10.8235
NDD1	YLR420W					3.7382			0.64599	4.38419
NDD1	YLR429W						2.05968	2.79808	0.568056	5.42582
NDD1	YLR438C-A	8.09235					1.07527			9.16762
NDD1	YLR439W	8.09235								8.09235
NDD1	YML001W						1.99648	2.03833	0.568417	4.60323
NDD1	YML015C					3.44746	1.89092			5.33838
NDD1	YML022W		0.866788		2.45623				0.727453	4.05047
NDD1	YML024W		0.971367		2.57297		1.09791		0.729339	5.37159
NDD1	YML027W	1.39994	1.06505	1.5795	1.38491		1.95849		0.730894	8.11878
NDD1	YML034W	0.287501	0.460081		2.81533				0.6732	4.23611
NDD1	YML041C		1.41762			3.2218				4.63942
NDD1	YML050W	6.63052								6.63052
NDD1	YML051W	15.8551	1.53129	0.259931						17.6463
NDD1	YML052W	15.8551	1.53129	2.40617	2.76899					22.5615
NDD1	YML053C	15.8551	1.53129	3.83635						21.2227
NDD1	YML058W	0.735968	0.015825	1.58692	2.52339					4.8621
NDD1	YML064C					3.67116	2.04362			5.71478
NDD1	YML078W		1.84946	10.5786			1.12745			13.5555
NDD1	YML093W		0.0603717	1.92375	2.63074		2.04213		0.5473	7.20429
NDD1	YML119W	0.633959	0.845756	12.4249					0.726358	14.631
NDD1	YML120C	0.633959	0.845756	12.9409	1.02493		1.20322			16.6488
NDD1	YML125C	2.13781					1.19628		0.706477	4.04057
NDD1	YML126C	2.13781			2.47323				0.727608	5.33865
NDD1	YML131W	1.31964			1.6148		1.14148			4.07592
NDD1	YMR001C	6.05279	0.548974	0.724968	3.13428		1.88884		0.692418	13.0423
NDD1	YMR002W	6.05279	0.411632	1.2681	2.54971					10.2822
NDD1	YMR003W	6.05279	0.548974	0.297558						6.89932
NDD1	YMR012W					1.92353	2.05716		0.713345	4.69404
NDD1	YMR031C	0.734488	1.03116	2.25104			1.23805	2.06989		7.32463
NDD1	YMR032W	0.734488	1.03116	3.17296	3.17877				0.706327	8.82371
NDD1	YMR049C				2.61809		2.07565		0.689869	5.38361
NDD1	YMR104C		0.95072	3.53131						4.48203
NDD1	YMR116C				0.542652	3.9249	1.99913		0.645004	7.11169
NDD1	YMR119W	1.71683		0.683735	2.32708		1.916			6.64365
NDD1	YMR121C		0.491944	1.9421	0.336036		1.11665		0.729028	4.61576
NDD1	YMR122W-A		0.491944	4.465						4.95694
NDD1	YMR131C				2.53561		2.09567		0.709364	5.34064
NDD1	YMR136W	1.46304			2.90419					4.36723
NDD1	YMR138W				2.01764		2.00675			4.02439
NDD1	YMR143W				2.51211		1.19578		0.690823	4.39871
NDD1	YMR144W	13.2479								13.2479
NDD1	YMR145C	0.827201	0.573591		3.25771		1.18758		0.719494	6.56558
NDD1	YMR146C		0.924805		0.761414		2.06872	2.82983	0.587401	7.17217
NDD1	YMR148W		0.924805	3.56534						4.49015
NDD1	YMR165C	4.98326								4.98326
NDD1	YMR182C	2.80669			2.64951		1.87141	2.93651		10.2641
NDD1	YMR199W	8.87004	0.422995	1.0617		3.2218			0.574583	14.1511
NDD1	YMR223W			1.85628		0.412221	1.97063	1.57536		5.81449
NDD1	YMR238W		1.41762	5.2972						6.71482
NDD1	YMR251W-A		1.42185	6.50356	2.05335					9.97876
NDD1	YMR304W				1.90499	3.9249	1.87616	1.58217		9.28822
NDD1	YMR305C		0.469471	2.34192	1.23034				0.714726	4.75646
NDD1	YMR308C				0.784041	4.11544			0.615556	5.51504
NDD1	YMR315W				2.01299		2.0144			4.02739
NDD1	YNL056W	7.46277	0.791058	3.22648						11.4803
NDD1	YNL058C	7.46277	0.791058	2.23144	2.45254				0.615162	13.553
NDD1	YNL059C	0.334009	0.791058		2.12564		2.04712			5.29783
NDD1	YNL110C			0.636129	2.60435		2.00183			5.24231
NDD1	YNL111C			0.977196	2.46394		1.94048	0.935537	0.726464	7.04362
NDD1	YNL132W	2.14172			2.52224				0.704872	5.36883
NDD1	YNL135C	2.14172		0.12268	0.928917	3.67116				6.86448
NDD1	YNL145W	3.65899	1.06674							4.72573
NDD1	YNL146C-A	3.65899	1.06674							4.72573
NDD1	YNL147W				2.4668	2.01987	1.02666			5.51333
NDD1	YNL148C	3.65899	1.06674							4.72573
NDD1	YNL160W	0.747795	1.41868	8.45061	1.40332					12.0204
NDD1	YNL175C				1.53922	0.687755	2.0723		0.680672	4.97995
NDD1	YNL178W				2.46706		1.1748		0.69735	4.33921
NDD1	YNL241C	4.44763								4.44763
NDD1	YNL288W	1.19644			2.6238	1.22213				5.04237
NDD1	YNL289W	1.16759	1.30701	6.55717			1.87141		0.4966	11.3998
NDD1	YNL298W		0.478221	10.2128						10.691
NDD1	YNR037C					3.2218	0.959812			4.18161
NDD1	YNR047W		1.3996		2.81288					4.21248
NDD1	YNR062C		0.845756	5.25292						6.09868
NDD1	YOL011W		0.50066	1.39108	2.94827				0.479628	5.31964
NDD1	YOL041C	0.391529			2.61912		2.08084			5.09149
NDD1	YOL069W		1.41285	6.05018	2.59031					10.0533
NDD1	YOL070C		1.41285	4.10023	2.68971					8.20279
NDD1	YOL077C				2.5572		2.02662			4.58382
NDD1	YOL091W		0.944396	0.578862			1.24061	1.52667		4.29054
NDD1	YOL145C		1.3996	2.92141			2.03411		0.493643	6.84876
NDD1	YOR025W	0.890361			3.10294		1.87214		0.691494	6.55693
NDD1	YOR033C					3.2218	1.20165		0.691588	5.11504
NDD1	YOR058C		1.45927	2.48919	2.43357			2.08982		8.47185
NDD1	YOR101W						2.04512	2.03838		4.0835

TF	Target	Binding_0-2000	Motif_Score_SC	Other_Yeasts	Two-Hop_BE	Two-Hop_BP	Two-Hop_BY	Two-Hop_BG	Max_Membership	Sum
NDD1	YOR119C			2.15016	2.52766		0.950315			5.62814
NDD1	YOR120W		0.160331	2.17559	1.63701		1.17068			5.14361
NDD1	YOR140W	3.23539			2.2698	0.548492	1.98443			8.03811
NDD1	YOR165W						2.03222	1.65379	0.575974	4.26198
NDD1	YOR210W				2.51239	1.67499			0.671014	4.85839
NDD1	YOR228C		0.587698	10.8835						11.4712
NDD1	YOR229W		0.587698	4.15543						4.74313
NDD1	YOR234C	4.68304			0.620298				0.714797	6.01814
NDD1	YOR236W	4.68304							0.627343	5.31038
NDD1	YOR237W	4.68304								4.68304
NDD1	YOR246C	8.17271		0.304197						8.47691
NDD1	YOR247W	8.17271		0.300868	2.91996				0.483208	11.8767
NDD1	YOR257W				2.83473		2.04834			4.88307
NDD1	YOR272W				2.53191		2.06382		0.670166	5.2659
NDD1	YOR313C	15.8551	0.909199		2.85057				0.510975	20.1258
NDD1	YOR315W	15.8551	0.909199	3.27129	2.85057				0.653177	23.5393
NDD1	YOR316C	3.91733							0.492287	4.40962
NDD1	YOR317W	3.91733				3.7382			0.481899	8.13743
NDD1	YOR369C	1.60587			2.53684		1.1321		0.701818	5.97663
NDD1	YPL004C				0.950697	3.2218				4.1725
NDD1	YPL014W		1.11271	3.34494						4.45765
NDD1	YPL015C		1.11271	4.39961						5.51232
NDD1	YPL081W	1.34223			1.01843		1.12948		0.715401	4.20554
NDD1	YPL141C	3.62702			3.29046				0.706048	7.62353
NDD1	YPL143W	2.34408	0.179455		1.09808				0.730868	4.35248
NDD1	YPL159C		0.680656			3.44746				4.12812
NDD1	YPL183C				0.704878		2.00764	3.1506	0.632697	6.49582
NDD1	YPL184C				2.70666		2.07681			4.78347
NDD1	YPL187W		1.09369	5.98676						7.08045
NDD1	YPL196W	2.54261	0.0664192		1.73699					4.34602
NDD1	YPL210C		1.30701	2.86125						4.16826
NDD1	YPL219W				2.23366	3.2218				5.45546
NDD1	YPL220W		0.753703	0.264347	2.51955		0.950144		0.663164	5.15091
NDD1	YPL242C	0.6933			2.87257		1.89865		0.61232	6.07684
NDD1	YPL248C					4.11544				4.11544
NDD1	YPL263C		0.658921	1.7467	0.561485		2.02246	1.6237	0.537173	7.15044
NDD1	YPL270W		0.633456	0.866583		3.2218				4.72184
NDD1	YPL276W				2.28307		2.08495			4.36802
NDD1	YPR008W	1.59689						2.7252		4.32209
NDD1	YPR028W	0.408312					2.04712	2.97915		5.43458
NDD1	YPR062W		0.518051		2.54668		1.13873			4.20346
NDD1	YPR086W					4.11544	1.12129			5.23673
NDD1	YPR102C				2.44677		1.14019		0.700152	4.28711
NDD1	YPR106W					3.44746	0.801735			4.2492
NDD1	YPR110C				2.47991	1.97824	1.13625		0.716965	6.31137
NDD1	YPR113W			1.89088	0.87003		1.96902		0.641748	5.37168
NDD1	YPR119W	15.8551	1.32479	3.14369	2.87449	4.11544	1.94179		0.725537	29.9808
NDD1	YPR120C	0.118921	0.64499	0.390787	2.6912	3.2218	1.94048			9.00818
NDD1	YPR148C	8.27732								8.27732
NDD1	YPR149W	8.27732		2.57788	2.5613					13.4165
NDD1	YPR152C					4.11544				4.11544
NDD1	YPR156C		0.598714	1.32104	2.46171				0.470355	4.85182
NDD1	YPR188C					2.92563	1.94458			4.87021
NRG1	SNR128	4.19767								4.19767
NRG1	SNR190	4.19767								4.19767
NRG1	YAL022C	2.92706	0.786328	1.56588	1.12043				0.63444	7.03414
NRG1	YAL059W	3.0904	0.786328	1.56645	1.05298	1.0951				7.59126
NRG1	YAL060W	3.0904	0.786328	0.0145276	1.51872					5.40998
NRG1	YAR029W	7.01607			0.998921					8.01499
NRG1	YAR031W	7.01607			0.998921					8.01499
NRG1	YBL029C-A	6.73442								6.73442
NRG1	YBL029W	6.73442								6.73442
NRG1	YBL081W	1.49154	0.786328	2.44237						4.72024
NRG1	YBR018C	2.17251	0.786328	0.805608	0.492865					4.25731
NRG1	YBR058C		0.786328	2.44877		1.0951				4.3302
NRG1	YBR058C-A		0.786328	4.37048						5.15681
NRG1	YBR088C			3.92422			0.786106			4.71033
NRG1	YBR133C		0.786328	4.0632						4.84953
NRG1	YBR150C	5.59336	0.786328	2.35106						8.73075
NRG1	YBR151W	5.59336	0.786328	3.23377						9.61346
NRG1	YBR152W	5.59336	0.786328							6.37969
NRG1	YBR158W	1.47222	0.786328	2.16937						4.42792
NRG1	YBR177C	4.79402	0.786328	1.62064						7.20099
NRG1	YCR010C		0.786328	3.2628	0.561543					4.61067
NRG1	YCR107W	3.61624			0.854129					4.47037
NRG1	YDL019C	3.22884						0.9321		4.16094
NRG1	YDL089W	2.40076	0.786328	2.63631						5.8234
NRG1	YDL090C	2.40076	0.786328	2.13602						5.32311
NRG1	YDL201W		0.786328	3.18454	0.782747					4.75361
NRG1	YDR041W	2.40757	0.786328	1.13459			0.802807			5.13129
NRG1	YDR043C	5.07251	0.786328	0.960936						6.81977
NRG1	YDR111C	2.1903	0.786328	3.73579			0.815577			7.528
NRG1	YDR435C	2.31918	0.786328	5.00603						8.11154
NRG1	YDR436W	2.31918	0.786328	3.61509			0.796135			7.51673
NRG1	YDR441C	5.22799								5.22799
NRG1	YDR466W	2.73055		2.14268						4.87323
NRG1	YDR536W	5.29547	0.786328	0.266675	0.344766					6.69324
NRG1	YEL020W-A	3.61065	0.786328	1.64555		0.325017				6.36754
NRG1	YEL021W	3.61065	0.786328	1.76675						6.16373
NRG1	YEL040W	4.39413	0.786328	0.465631						5.64609
NRG1	YEL046C	2.86049	0.786328		0.520719					4.16754
NRG1	YEL070W	5.50416								5.50416
NRG1	YER073W	0.861695		4.26187						5.12357
NRG1	YER091C	2.72439	0.786328	0.13199	0.572405					4.21511
NRG1	YER096W	3.02033	0.786328	1.06183						4.86849
NRG1	YER109C		0.786328	3.52105						4.30738
NRG1	YER145C	3.3978	0.786328	0.709956						4.89408
NRG1	YER146W	3.3978	0.786328	0.722722	0.360639	0.323926	0.802881			6.3943
NRG1	YFR017C	4.68008			1.4918					6.17188
NRG1	YFR019W	4.68008								4.68008
NRG1	YGL001C	3.6283			1.04882					4.67712
NRG1	YGL012W	6.46009	0.786328	0.795385						8.0418
NRG1	YGL013C	6.46009	0.786328	0.259234						7.50565
NRG1	YGR052W	5.13961			0.376603					5.51621
NRG1	YGR067C	3.33864	0.786328	1.37569	0.334917					5.83557
NRG1	YGR198W	0.430986	0.786328	3.276						4.49331
NRG1	YGR202C			4.03834						4.03834
NRG1	YGR237C	2.23112	0.786328	3.45727	1.35141					7.82613
NRG1	YGR243W	1.75717	0.786328	2.18665	0.371235					5.10138
NRG1	YGR264C	1.8476	0.786328	1.91246	0.721277					5.26767

TF	Target	Binding_0-2000	Motif_Score_SC	Other_Yeasts	Two-Hop_BE	Two-Hop_BP	Two-Hop_BY	Two-Hop_BG	Max_Membership	Sum
NRG1	YGR266W	1.8476	0.786328	2.77211						5.40604
NRG1	YHL021C		0.786328	3.09149	0.684935					4.56275
NRG1	YHL046C	1.53249	0.786328	0.654401	1.23294			0.952761		5.15892
NRG1	YHR094C	4.40583								4.40583
NRG1	YHR136C	0.964349	0.786328	2.63369	0.937784					5.32215
NRG1	YHR137W	0.964349	0.786328	2.70726						4.45794
NRG1	YHR199C		0.786328	3.52983						4.31616
NRG1	YIL004C	7.08407								7.08407
NRG1	YIL018W	6.30756	0.786328		0.585651					7.67954
NRG1	YIL019W	6.30756	0.786328	1.00571	0.395797					8.4954
NRG1	YIL020C	6.30756	0.786328	3.96927						11.0632
NRG1	YIL021W			3.33901			0.784443			4.12345
NRG1	YIL057C	2.87405		0.66839	0.787143					4.32958
NRG1	YIL097W	3.73896	0.786328		1.04168					5.56697
NRG1	YIL099W	10.3796	0.786328	0.269435	1.53156					12.9669
NRG1	YIL101C	10.3796	0.786328	1.81365	1.53156					14.5111
NRG1	YIL118W	5.04464			0.687864					5.7325
NRG1	YIL119C	5.04464								5.04464
NRG1	YIR013C	7.72831	0.786328	4.28773						12.8024
NRG1	YIR014W	7.72831	0.786328	1.14376	1.41475					11.0731
NRG1	YIR015W	7.72831	0.786328	0.323242						8.83788
NRG1	YIR019C	8.68968	0.786328	0.249148						9.72516
NRG1	YJL076W	3.00265	0.786328	3.45015						7.23913
NRG1	YJL078C	3.00265	0.786328	0.245389				1.73331		5.76768
NRG1	YJL106W	3.91191	0.786328			0.939691				5.63793
NRG1	YJL107C	3.91191	0.786328	0.200711						4.89895
NRG1	YJL131C		0.786328	3.86206						4.64839
NRG1	YJL151C		0.786328	2.85453	0.86174					4.5026
NRG1	YJL194W	3.31261					0.798173			4.11078
NRG1	YJR063W	0.386486		2.50352	1.05497		0.805393			4.75037
NRG1	YJR094C	4.09108	0.786328			0.891325				5.76873
NRG1	YKL096W	3.17707		3.4452						6.62227
NRG1	YKL109W	3.3069			0.767138					4.07404
NRG1	YKL141W			5.85585	0.333736					6.18959
NRG1	YKR065C	2.65524	0.786328	0.41036	0.523069					4.375
NRG1	YKR066C	2.65524	0.786328	2.19855		0.351668				5.99179
NRG1	YKR067W	2.65524	0.786328	1.49974	0.942685					5.88399
NRG1	YKR102W	4.05297	0.786328							4.8393
NRG1	YLL011W	3.50798			0.816747					4.32473
NRG1	YLL028W	0.537041	0.786328	4.33975			0.930206			6.59333
NRG1	YLR012C	4.69213	0.786328	8.51095						13.9894
NRG1	YLR013W	4.69213	0.786328	1.67041						7.14887
NRG1	YLR048W	1.3901	0.786328		1.59017		0.789884			4.55648
NRG1	YLR110C	4.54223	0.786328	0.615245						5.9438
NRG1	YLR113W	4.54223	0.786328	0.594788						5.92335
NRG1	YLR187W	5.07471	0.786328	0.298713						6.15975
NRG1	YLR267W	3.20779		0.685156	0.544834					4.43778
NRG1	YLR312C	1.32914	0.786328	4.81872	0.750175					7.68436
NRG1	YLR314C			2.25637		1.09688		0.966682		4.31993
NRG1	YLR393W		0.786328	3.4799						4.26623
NRG1	YML087C		0.786328	3.00664				0.954091		4.74706
NRG1	YML100W		0.786328	3.30599	1.19938					5.2917
NRG1	YML121W	3.25927	0.786328	1.30648						5.35208
NRG1	YML123C	3.25927	0.786328	0.941265			0.865853			5.85272
NRG1	YMR011W	4.14539								4.14539
NRG1	YMR055C	4.67335								4.67335
NRG1	YMR058W	4.67335								4.67335
NRG1	YMR070W	4.52759								4.52759
NRG1	YMR084W	2.04974	0.786328		0.446953			0.928457		4.21148
NRG1	YMR085W	2.04974	0.786328		0.491057			0.928457		4.25558
NRG1	YMR150C			3.68548					0.61418	4.29966
NRG1	YMR152W			5.19784						5.19784
NRG1	YMR195W	3.57202		1.33058						4.9026
NRG1	YMR261C	0.0665725	0.786328	2.63467					0.704983	4.19255
NRG1	YMR263W	0.0665725	0.786328	4.24234						5.09524
NRG1	YMR319C	5.53396	0.786328	0.716084						7.03637
NRG1	YNL065W	6.06232								6.06232
NRG1	YNL087W	3.65983			0.378097					4.03793
NRG1	YNL194C	0.579222		0.98091	1.78998				0.679449	4.02956
NRG1	YNL231C	3.92776		0.466341	0.351851					4.74595
NRG1	YNR038W		0.786328	4.21037						4.9967
NRG1	YNR060W	6.93382	0.786328	1.66066	0.373588					9.7544
NRG1	YNR070W	3.40981	0.786328							4.19614
NRG1	YOL014W	1.30337	0.786328	5.19457						7.28427
NRG1	YOL028C	3.72092	0.786328	0.364464	0.604038					5.47575
NRG1	YOL029C	3.72092	0.786328	0.370008						4.87726
NRG1	YOL084W	5.37258			1.92962					7.3022
NRG1	YOL086C	5.37258			0.387821	0.479019				6.23942
NRG1	YOL154W	0.210579	0.786328	3.40736						4.40427
NRG1	YOL156W	4.7921			0.506181					5.29828
NRG1	YOL157C	4.7921								4.7921
NRG1	YOL158C	2.50409	0.786328	0.632618	0.515552		0.886182	0.996587		6.32136
NRG1	YOR049C	4.19371								4.19371
NRG1	YOR113W		0.786328	3.46321						4.24954
NRG1	YOR138C	7.74792	0.786328	2.47693						11.0112
NRG1	YOR140W	7.74792	0.786328	1.81077						10.345
NRG1	YOR152C	5.17717		0.288875						5.46605
NRG1	YOR153W	5.17717								5.17717
NRG1	YOR178C	4.47508								4.47508
NRG1	YOR179C	3.37777	0.786328							4.1641
NRG1	YOR273C	4.68306								4.68306
NRG1	YOR274W	4.68306								4.68306
NRG1	YOR297C		0.786328	4.11668		0.344373				5.24738
NRG1	YOR311C	1.40889	0.786328	1.91971						4.11493
NRG1	YPL021W	1.32277	0.786328	5.11798						7.22708
NRG1	YPL024W	2.98694	0.786328	1.26061						5.03388
NRG1	YPL034W	4.6041								4.6041
NRG1	YPL230W	2.50896			1.61915					4.12811
NRG1	YPL233W		0.786328	3.21655						4.00288
NRG1	YPL253C	4.01552								4.01552
NRG1	YPR145W	0.136607	0.786328	4.08963						5.01256
NRG1	YPR156C	2.48346	0.786328		0.381281		0.822066			4.47314
NRG1	YPR158W	3.8993	0.786328							4.68563
OAF1	YAL035W	3.64057				0.589547				4.23012
OAF1	YBR214W	4.50473								4.50473
OAF1	YDR256C	4.12293								4.12293
OAF1	YHR115C	4.63729								4.63729
OAF1	YHR117W	4.63729								4.63729
OAF1	YLR175W	3.49983			0.329448	0.627541				4.45682
OAF1	YML072C	5.10588								5.10588

TF	Target	Binding_0-2000	Motif_Score_SC	Other_Yeasts	Two-Hop_BE	Two-Hop_BP	Two-Hop_BY	Two-Hop_BG	Max_Membership	Sum
OPI1	YBR021W		0.343611	5.16832						5.51193
OPI1	YCR027C		0.343611	3.97475						4.31836
OPI1	YDR035W		0.343611	3.7267						4.07031
OPI1	YDR053W			4.41158						4.41158
OPI1	YDR262W		0.343611	4.12925						4.47286
OPI1	YDR363W-A		0.343611	4.41704						4.76065
OPI1	YER012W		0.343611	4.30172						4.64533
OPI1	YER075C	0.128292	0.343611	4.76293						5.23483
OPI1	YFL010C		0.343611	4.30178						4.64539
OPI1	YIL020C		0.343611	4.21547						4.55908
OPI1	YJL006C	0.684963	0.343611	3.0628						4.09137
OPI1	YJR052W		0.343611	4.82354						5.16715
OPI1	YJR101W	0.9048	0.343611	5.05412						6.30253
OPI1	YKL007W			4.0752						4.0752
OPI1	YKL128C		0.343611	4.72857						5.07218
OPI1	YKL182W	2.58521		1.52078		0.80276		1.2049		6.11365
OPI1	YLR303W		0.343611	7.5558						7.89941
OPI1	YLR312C			5.24555						5.24555
OPI1	YMR246W		0.343611	3.69168						4.03529
OPI1	YPL231W	3.58385	0.343611			0.533176		1.02495		5.48559
PDC2	YLL025W	6.62514								6.62514
PDR1	TE(UUC)M	4.1985								4.1985
PDR1	YAR028W	3.81826		1.54801						5.36627
PDR1	YBL005W		1.93655	6.6956	1.5289					10.161
PDR1	YBL006C		1.93655	7.69265						9.6292
PDR1	YBL007C		1.93655	4.70363						6.64018
PDR1	YBL027W	3.67448			0.786461		0.973224			5.43417
PDR1	YBL028C	3.67448		0.576949	1.6338					5.88523
PDR1	YBR040W	5.32377								5.32377
PDR1	YBR041W	5.32377								5.32377
PDR1	YBR048W	3.81826			1.03961		0.781709			5.63958
PDR1	YBR057C	4.41514						1.14833		5.56347
PDR1	YCL067C	8.70904			0.614629					9.32367
PDR1	YCR010C		1.17159	3.84542						5.01701
PDR1	YCR061W		1.17159	3.19263						4.36422
PDR1	YDL020C		1.17159	3.8022	0.653407					5.6272
PDR1	YDL148C	3.2333			0.885174		0.814255			4.93273
PDR1	YDR072C		1.93655	3.30715						5.2437
PDR1	YDR073W		1.93655	3.70266		0.708129				6.34734
PDR1	YDR297W		1.17159	3.18589						4.35748
PDR1	YDR322C-A		1.93655	9.88428						11.8208
PDR1	YDR332W	5.58781								5.58781
PDR1	YDR345C		1.93655	4.66619			0.816725			7.41947
PDR1	YDR366C	6.45454			1.07516					7.5297
PDR1	YDR368W	6.45454								6.45454
PDR1	YDR470C	3.31473	0.468845	0.934486						4.71806
PDR1	YDR471W	3.31473	0.468845		1.10198					4.88556
PDR1	YDR545W	6.6751								6.6751
PDR1	YER074W	3.36389			1.08343					4.44732
PDR1	YER074W-A	3.36389	1.17159							4.53548
PDR1	YER115C	1.99561		3.06412						5.05973
PDR1	YER168C			5.74249						5.74249
PDR1	YFL017W-A		1.93655	3.39053		0.379038				5.70612
PDR1	YFL018C		1.93655	5.24467						7.18122
PDR1	YGL209W		1.93655	8.4856						10.4222
PDR1	YGR017W		1.93655	6.28143						8.21798
PDR1	YGR035C		1.17159	4.31842						5.49001
PDR1	YGR161C		1.17159	4.54036	0.41362					6.12557
PDR1	YGR280C		1.17159	1.79804	1.12269					4.09232
PDR1	YHL013C	2.3047			0.601327			1.28661		4.19264
PDR1	YHR029C		1.93655	7.57431						9.51086
PDR1	YHR087W	6.822								6.822
PDR1	YHR088W	6.822			0.990839					7.81284
PDR1	YHR091C	8.99386		0.727112						9.72097
PDR1	YHR140W		1.93655	2.8173						4.75385
PDR1	YHR141C	4.30636	1.17159		1.12279					6.60074
PDR1	YHR142W	4.30636								4.30636
PDR1	YHR148W	1.76318	1.17159	0.45278	0.870981					4.25853
PDR1	YHR191C	7.50005								7.50005
PDR1	YHR193C	7.50005								7.50005
PDR1	YHR194W	7.50005								7.50005
PDR1	YIL018W	2.83919			1.22774					4.06693
PDR1	YIL056W		1.93655	5.14232						7.07887
PDR1	YIL057C		1.93655	9.75716						11.6937
PDR1	YIL133C	2.92905			1.22694		0.89169			5.04768
PDR1	YIR017C		1.17159	3.91474						5.08633
PDR1	YIR018W		1.17159	6.4691						7.64069
PDR1	YJL082W		1.93655	8.38787						10.3244
PDR1	YJL190C	4.43033			1.09747					5.5278
PDR1	YKL007W		1.93655	8.1273						10.0639
PDR1	YKL008C		1.93655	2.44568						4.38223
PDR1	YKL039W			2.31912	1.30625		1.16359			4.78896
PDR1	YKL201C		1.17159	1.52309	1.62225					4.31693
PDR1	YLL028W		1.17159	5.85407						7.02566
PDR1	YLL066C	7.03644						1.12437		8.16081
PDR1	YLR099C		1.17159	4.27287						5.44446
PDR1	YLR099W-A		1.17159	5.06908						6.24067
PDR1	YLR207W	6.28643								6.28643
PDR1	YLR250W			4.36708	1.25035					5.61743
PDR1	YLR346C		1.93655	3.54497						5.48152
PDR1	YML063W	0.462155	1.17159	1.50664	1.12604					4.26642
PDR1	YML074C			5.1643	0.536909					5.70121
PDR1	YML125C	0.743029	1.93655	5.03326			0.789945			8.50278
PDR1	YMR012W	4.1985			0.704698		0.908598			5.8118
PDR1	YMR015C	1.26059		2.70998	0.642819					4.61339
PDR1	YMR022W			3.94287	0.621352					4.56422
PDR1	YMR165C	4.73651								4.73651
PDR1	YMR195W		1.93655	7.98594						9.92249
PDR1	YMR241W	2.72003			0.347602		1.18761			4.25524
PDR1	YMR246W		0.812883	0.987167			0.844495	1.50285		4.14739
PDR1	YNL069C	2.24179			1.25669		0.780419			4.2789
PDR1	YNL149C		0.812883	3.27534						4.08822
PDR1	YNL160W		1.93655	5.45758						7.39413
PDR1	YNL231C		1.93655	6.14353						8.08008
PDR1	YNL236W	5.84412				0.492856				6.33698
PDR1	YNL336W	9.26697								9.26697
PDR1	YNL339C	9.26697			0.516558					9.78353
PDR1	YOL080C	3.45132			1.05446					4.50578
PDR1	YOL157C		1.93655	3.44127						5.37782
PDR1	YOR152C		1.93655	6.30172						8.23827

TF	Target	Binding_0-2000	Motif_Score_SC	Other_Yeasts	Two-Hop_BE	Two-Hop_BP	Two-Hop_BY	Two-Hop_BG	Max_Membership	Sum
PDR1	YOR153W		1.93655	3.58897						5.52552
PDR1	YOR162C	0.477448	1.93655	2.32949						4.74349
PDR1	YOR163W		1.93655	4.73443			1.07853			7.74951
PDR1	YOR179C			7.43486						7.43486
PDR1	YOR273C	1.1712	1.17159	2.34371			0.776383			5.46288
PDR1	YOR274W	1.1712	1.17159	4.81645						7.15924
PDR1	YOR369C	1.19497		1.32842	0.896864		1.08412			4.50437
PDR1	YPL018W	3.13024	1.17159							4.30183
PDR1	YPL061W		1.93655	10.6093						12.5458
PDR1	YPL062W		1.93655	2.14575						4.0823
PDR1	YPL063W			5.7283						5.7283
PDR1	YPL066W		1.93655	7.53544						9.47199
PDR1	YPL068C		1.93655	8.64236						10.5789
PDR1	YPL249C-A	2.13507	1.17159	0.0658715	0.653227					4.02576
PDR1	YPL253C	5.58654						0.937251		6.52379
PDR1	YPR149W		1.93655	5.03319						6.96974
PDR1	YPR166C	3.13761			1.10306					4.24067
PDR1	YPR202W	3.13699			1.76876			1.82704		6.73279
PDR1	YPR203W	3.13699			1.71334			1.12437		5.9747
PDR3	YBL005W	3.39819	1.91187	5.83302						11.1431
PDR3	YBL006C	3.39819	1.91187	6.8015						12.1116
PDR3	YBL007C	3.39819	1.91187	5.12236						10.4324
PDR3	YBR034C		1.91187	1.7938	0.432804					4.13847
PDR3	YDR072C		1.91187	3.32505						5.23692
PDR3	YDR073W		1.91187	6.40833						8.3202
PDR3	YDR322C-A		1.91187	6.23688						8.14875
PDR3	YDR345C		1.91187	2.65026						4.56213
PDR3	YER115C			4.16113						4.16113
PDR3	YFL017W-A		1.91187	5.94612						7.85799
PDR3	YFL018C		1.91187	6.1695						8.08137
PDR3	YGL209W		1.91187	11.7195						13.6314
PDR3	YGR017W		1.91187	8.27163						10.1835
PDR3	YHR029C		1.91187	7.06674						8.97861
PDR3	YHR140W		1.91187	3.86409						5.77596
PDR3	YIL056W		1.91187	6.50009						8.41196
PDR3	YIL057C		1.91187	6.18009						8.09196
PDR3	YIR018W			5.71623						5.71623
PDR3	YJL082W		1.91187	9.16317						11.075
PDR3	YKL007W		1.91187	7.12623						9.0381
PDR3	YKL039W			4.23783						4.23783
PDR3	YLR099C			4.12891						4.12891
PDR3	YLR250W			4.16808						4.16808
PDR3	YLR346C		1.91187	5.30612						7.21799
PDR3	YML125C		1.91187	8.75255						10.6644
PDR3	YMR195W		1.91187	6.62117						8.53304
PDR3	YNL160W		1.91187	6.30227						8.21414
PDR3	YNL231C		1.91187	3.07569						4.98756
PDR3	YNL258C	4.2499								4.2499
PDR3	YOR049C		1.91187	3.16192						5.07379
PDR3	YOR152C		1.91187	4.08592						5.99779
PDR3	YOR162C		1.91187	3.55077						5.46264
PDR3	YOR163W		1.91187	5.84291						7.75478
PDR3	YOR179C			4.49606						4.49606
PDR3	YPL061W		1.91187	6.98194						8.89381
PDR3	YPL066W	0.628915	1.91187	8.12766						10.6684
PDR3	YPL068C	0.628915	1.91187	5.19183						7.73262
PDR3	YPL137C		1.91187	2.22494						4.13681
PDR3	YPR149W		1.91187	2.70744						4.61931
PHD1	SNR3	7.01541								7.01541
PHD1	TC(GCA)P2	4.35019								4.35019
PHD1	TL(UAA)B1	5.35686								5.35686
PHD1	YAL063C	7.23069						1.27077		8.50146
PHD1	YAR003W			3.61433			0.991479			4.60581
PHD1	YAR050W	2.15276			0.682378			1.44031		4.27545
PHD1	YBL005W		0.25661	4.16794						4.42455
PHD1	YBL007C			5.94723						5.94723
PHD1	YBL015W	1.76092	0.0259801	1.77034					0.699562	4.2568
PHD1	YBL023C	0.803329		2.48583			1.05126			4.34042
PHD1	YBL027W	2.53633			1.51399		1.02516			5.07548
PHD1	YBL029C-A	6.20759	0.238396	0.31004						6.75603
PHD1	YBL029W	6.20759	0.238396	0.407511						6.8535
PHD1	YBL030C	3.53454	0.238396		0.957756					4.73069
PHD1	YBL041W		0.397713	8.63185			1.04604			10.0756
PHD1	YBL065W			4.19102						4.19102
PHD1	YBL072C	1.24201		4.93537	1.67807		0.961392			8.81684
PHD1	YBL108C-A	5.35686						1.42761		6.78447
PHD1	YBR106W	0.276911	0.368498	2.0974	1.30944					4.05225
PHD1	YBR141C			6.11699						6.11699
PHD1	YBR142W			4.66356	0.444168					5.10773
PHD1	YBR234C		0.25661	4.9948						5.25141
PHD1	YBR253W	0.24509	0.386299	6.83754						7.46893
PHD1	YBR288C		0.415422	2.82584	0.706513		0.776672			4.72445
PHD1	YBR296C			4.23886						4.23886
PHD1	YBR301W			3.2448				1.42213		4.66693
PHD1	YCL049C			2.20488	1.80864					4.01352
PHD1	YCR065W	6.93631		0.301529	0.453246					7.69109
PHD1	YCR098C	4.13153					1.03531			5.16684
PHD1	YDL047W	3.02601	0.386299	3.28064						6.69295
PHD1	YDL214C	1.90002	0.238396	2.49212						4.63054
PHD1	YDR261C	4.92675	0.25661							5.18336
PHD1	YDR275W			4.84483	0.357465					5.2023
PHD1	YDR303C			5.13836						5.13836
PHD1	YDR343C	7.09353			1.40068					8.49421
PHD1	YDR373W	1.39009			1.03011		0.941588	1.41701		4.7788
PHD1	YEL007W	2.96248	0.397713	4.03198						7.39217
PHD1	YEL037C		0.397713	5.30541						5.70312
PHD1	YEL040W	7.09492								7.09492
PHD1	YEL075C	3.56256			0.398628			1.38232		5.34351
PHD1	YEL076C	2.80146			0.608127			1.38232		4.79191
PHD1	YEL076C-A	2.80146			0.608127			1.38232		4.79191
PHD1	YEL077C	2.80146			0.50438			1.20106		4.5069
PHD1	YER033C	5.87592		4.90811						10.784
PHD1	YER034W	5.87592		0.87635						6.75227
PHD1	YER035W	5.87592		3.91652						9.79244
PHD1	YFL055W			2.9834	0.480922				0.62108	4.0854
PHD1	YGL035C		0.415422	6.85623		0.482954		1.49026		9.24487
PHD1	YGL154C			3.38777	1.7306					5.11837
PHD1	YGL233W		0.415422	5.23316						5.64858
PHD1	YGR035C		0.386299	6.02569						6.41199
PHD1	YGR105W		0.25661	4.99128						5.24789

TF	Target	Binding_0-2000	Motif_Score_SC	Other_Yeasts	Two-Hop_BE	Two-Hop_BP	Two-Hop_BY	Two-Hop_BG	Max_Membership	Sum
PHD1	YGR220C		0.238396	4.23761			0.948521			5.42453
PHD1	YGR249W	5.5478								5.5478
PHD1	YGR251W	4.09206			0.467436					4.5595
PHD1	YHR005C	4.14634					0.979603			5.12594
PHD1	YHR152W	0.445986		5.06886						5.51485
PHD1	YIL010W	5.10246	0.226657							5.32912
PHD1	YIL011W	6.37866	0.226657	0.997441				1.42431		9.02707
PHD1	YIL013C	6.37866	0.226657							6.60532
PHD1	YIL056W	3.46154		3.3582						6.81974
PHD1	YIL057C	3.46154		3.48127					0.732343	7.67515
PHD1	YIL099W	6.31852			0.702004					7.02052
PHD1	YIL101C	6.31852	0.226657		0.702004					7.24718
PHD1	YIL108W			4.42137						4.42137
PHD1	YIL118W	2.36592	0.368498	0.131625	0.67567		0.827593			4.36931
PHD1	YIL119C	2.36592	0.368498	2.68841	0.381984				0.600154	6.40497
PHD1	YIL124W			8.61984	0.741111					9.36095
PHD1	YIL133C	1.73711			1.53862		1.05019			4.32592
PHD1	YJL076W	1.18911	0.25661	2.87315			0.802954			5.12182
PHD1	YJL078C	1.18911	0.25661	1.32895				1.38232		4.15699
PHD1	YJL105W	5.42638								5.42638
PHD1	YJL136W-A	4.56847								4.56847
PHD1	YJL138C	4.56847			1.19422			1.20106		6.96375
PHD1	YJL139C	4.56847								4.56847
PHD1	YJL177W			1.85823	1.71519		0.995333			4.56875
PHD1	YJR094C	3.99926	0.415422	0.205672		0.416708				5.03706
PHD1	YJR109C		0.415422	3.69168						4.1071
PHD1	YJR122W			4.07937						4.07937
PHD1	YJR123W		0.415422	2.60643	1.67747		1.04323			5.74255
PHD1	YJR127C	7.01541								7.01541
PHD1	YJR147W	0.267117		5.10611						5.37323
PHD1	YKL004W	0.305682		3.56247	0.406798					4.27495
PHD1	YKL062W	8.23501	0.25661	3.01962	1.16751					12.6788
PHD1	YKL063C	8.23501	0.25661	1.0666						9.55822
PHD1	YKL099C	1.52863		2.26232	0.674606					4.46556
PHD1	YKL109W	4.2507	0.397713	1.18262						5.83103
PHD1	YKL110C	4.23951	0.397713		0.437315					5.07454
PHD1	YKL204W		0.00700926	4.19378						4.20079
PHD1	YKL217W			2.92694	0.441478				0.726502	4.09492
PHD1	YKR072C	6.23727								6.23727
PHD1	YKR075C		0.368498	3.14324	1.33499					4.84673
PHD1	YLR170C		0.415422	6.31386						6.72928
PHD1	YLR312W-A	0.225596		3.01819	0.933446					4.17723
PHD1	YLR314C			6.03861			1.00758			7.04619
PHD1	YLR315W			4.11607						4.11607
PHD1	YLR401C			5.26285						5.26285
PHD1	YML116W	2.68125	0.415422	6.69622						9.79289
PHD1	YML128C		0.25661	5.15822	0.472348				0.463596	6.35077
PHD1	YML131W	4.15714								4.15714
PHD1	YMR003W		0.25661	6.24209						6.4987
PHD1	YMR016C	7.21046	0.238396	0.59611						8.04497
PHD1	YMR017W	7.21046	0.238396							7.44886
PHD1	YMR018W	6.18077	0.00700926							6.18778
PHD1	YMR019W	5.67857								5.67857
PHD1	YMR043W		0.415422	4.62686		0.476303				5.51858
PHD1	YMR135C	10.8564	0.415422	2.17747						13.4493
PHD1	YMR136W	10.8564	0.415422	1.38278						12.6546
PHD1	YNL178W	6.75236			0.748115					7.50048
PHD1	YNL180C	6.75236					0.854379			7.60674
PHD1	YNR018W		0.415422	5.89818						6.3136
PHD1	YOL065C		0.25661	4.13908						4.39569
PHD1	YOL110W			4.11525						4.11525
PHD1	YOR023C		0.00700926	6.90871						6.91572
PHD1	YOR025W			6.83473						6.83473
PHD1	YOR028C	2.33563	0.25661	1.50108						4.09332
PHD1	YOR152C		0.368498	6.64385	0.436684					7.44903
PHD1	YOR178C	4.19487	0.397713							4.59258
PHD1	YOR179C	5.53171								5.53171
PHD1	YOR348C	0.478093		1.28276	1.51259		0.871124		0.683086	4.82765
PHD1	YPL024W	4.76099		3.58283						8.34382
PHD1	YPL026C	4.76099		3.60616						8.36715
PHD1	YPL177C	5.68579					0.979603			6.66539
PHD1	YPL230W	3.01257		2.93435	0.789575					6.7365
PHD1	YPR013C	7.09541								7.09541
PHD1	YPR120C	4.35019			0.835434					5.18562
PHD1	YPR202W	2.92554			0.554417			1.38232		4.86228
PHD1	YPR203W	2.92554			0.554417			1.38232		4.86228
PHO2	TG(GCC)J1	4.20683								4.20683
PHO2	YJL024C	4.20683								4.20683
PHO4	YAL038W	0.653081	0.519291	5.47059						6.64296
PHO4	YAL053W		0.316687	4.58552						4.90221
PHO4	YBR093C	1.60043	0.519291	3.74568	1.08457					6.94997
PHO4	YBR094W	1.60043	0.519291	2.62426						4.74398
PHO4	YBR222C		0.316687	3.82842						4.14511
PHO4	YBR296C	2.01688	0.519291	3.67736					0.561311	6.77484
PHO4	YCL031C		0.519291	5.53277						6.05206
PHO4	YCL042W		0.316687	4.42791						4.7446
PHO4	YCL043C		0.316687	7.75411						8.0708
PHO4	YCR052W		0.519291	4.22418						4.74347
PHO4	YCR066W	4.84404				0.645253				5.48929
PHO4	YCR098C	2.27603	0.316687	1.72911						4.32183
PHO4	YDR281C	8.41126	0.519291	2.65066					0.535908	12.1171
PHO4	YDR441C	2.19163	0.519291	1.29919						4.01011
PHO4	YDR481C	2.40503	0.519291	1.83862						4.76294
PHO4	YDR483W	2.40503	0.519291	3.42125						6.34557
PHO4	YDR503C		0.519291	4.13605						4.65534
PHO4	YEL018W			4.13121						4.13121
PHO4	YER001W	1.96434	0.519291	1.5772						4.06083
PHO4	YER052C		0.519291	4.45977						4.97906
PHO4	YER053C		0.316687	2.61162	1.53663					4.46494
PHO4	YER078C	7.70693								7.70693
PHO4	YER079W	7.70693			0.336315	0.349056				8.3923
PHO4	YER080W	7.70693								7.70693
PHO4	YER129W	5.01881								5.01881
PHO4	YFL009W	2.4312				0.582738		0.992912		4.00685
PHO4	YGL019W	0.0967996		4.30146						4.39826
PHO4	YGL035C	0.938338	0.519291	2.91478						4.37241
PHO4	YGL058W	3.00039				1.14295				4.14334
PHO4	YGL079W	4.4833								4.4833
PHO4	YGL099W	3.60102			0.624122					4.22514
PHO4	YGR146C	1.774	0.519291	2.67799						4.97128

TF	Target	Binding_0-2000	Motif_Score_SC	Other_Yeasts	Two-Hop_BE	Two-Hop_BP	Two-Hop_BY	Two-Hop_BG	Max_Membership	Sum
PHO4	YGR273C	2.11097		6.14093						8.2519
PHO4	YGR276C			4.90882						4.90882
PHO4	YGR278W			4.84599						4.84599
PHO4	YHL004W		0.519291	4.72875						5.24804
PHO4	YHL007C		0.519291	3.76464						4.28393
PHO4	YHR018C			4.50693						4.50693
PHO4	YHR107C	0.146731	0.519291	7.94639						8.61241
PHO4	YHR123W			6.80563						6.80563
PHO4	YHR131C	4.06188	0.316687							4.37857
PHO4	YHR135C	3.54899	0.519291	3.62831		1.47151				9.1681
PHO4	YHR136C	3.54899	0.519291	1.98596	1.27796					7.3322
PHO4	YHR137W	3.54899	0.519291	3.48174	0.82929					8.37931
PHO4	YHR170W			3.16714	0.359316	0.616864			0.678213	4.82153
PHO4	YIL026C			4.20566		0.512183				4.71784
PHO4	YIL127C			3.95651	0.653038					4.60955
PHO4	YIL157C			4.4765						4.4765
PHO4	YJL052W	1.15056	0.519291	3.40568	0.327088					5.40262
PHO4	YJR009C		0.519291	4.57245	0.483289					5.57503
PHO4	YJR058C	2.26931	0.519291	3.25539		0.666394				6.71039
PHO4	YJR059W	2.26931	0.519291	2.81385						5.60245
PHO4	YJR060W		0.519291	3.98718						4.50647
PHO4	YJR137C		0.519291	3.48489						4.00418
PHO4	YJR138W		0.519291	3.92811						4.4474
PHO4	YKL190W	1.52866	0.519291	2.44867				1.46347		5.96009
PHO4	YKR035W-A	0.316461	0.519291	4.33229						5.16804
PHO4	YKR045C		0.519291	4.36944						4.88873
PHO4	YKR048C		0.519291	4.95743						5.47672
PHO4	YLR152C	6.90482								6.90482
PHO4	YLR266C	2.41868	0.519291	2.54631						5.48428
PHO4	YLR267W	2.41868	0.519291	4.0982						7.03617
PHO4	YLR397C	4.80102								4.80102
PHO4	YLR427W	0.128788	0.519291	6.55325						7.20133
PHO4	YML089C			4.27682						4.27682
PHO4	YML121W	3.17549	0.519291	3.05654						6.75132
PHO4	YML123C	3.17549	0.519291	2.57578	0.375314				0.639462	7.28534
PHO4	YMR290C	0.987455	0.519291	3.82334	0.939513				0.689282	6.95888
PHO4	YMR291W	0.987455	0.519291	2.23056	0.39451					4.13182
PHO4	YMR303C		0.519291	4.74068						5.25997
PHO4	YNL094W		0.519291	4.00394						4.52323
PHO4	YNR013C		0.519291	3.90295						4.42224
PHO4	YOL136C	2.76881	0.519291	2.23365						5.52175
PHO4	YOR163W	2.5123	0.316687	1.21206						4.04105
PHO4	YOR165W	2.99616	0.316687					1.10972		4.42257
PHO4	YPL018W	2.61423	0.519291	2.06041						5.19393
PHO4	YPL019C	2.61423	0.519291	1.67607					0.724211	5.5338
PHO4	YPL211W			3.98235	0.994578					4.97693
PHO4	YPL253C	5.44066								5.44066
PHO4	YPR047W	0.863394	0.519291	3.76218						5.14486
PHO4	YPR102C		0.519291	3.39551	0.473964					4.38876
PHO4	YPR168W		0.519291	4.11266		0.406192				5.03814
PIP2	YML072C	4.46152								4.46152
PPR1	YBR015C		1.79972	3.45745						5.25717
PPR1	YBR016W		1.79972	3.58356						5.38328
PPR1	YCR014C			4.38925						4.38925
PPR1	YCR092C		1.21508	4.08478						5.29986
PPR1	YCR093W		1.21508	4.49684						5.71192
PPR1	YDR004W	0.248948		8.10384						8.35279
PPR1	YDR076W			5.0097						5.0097
PPR1	YDR310C			6.07673						6.07673
PPR1	YDR328C			5.08095		0.420049				5.501
PPR1	YEL021W		0.898191	6.38183						7.28002
PPR1	YER022W	2.05607		3.78368						5.83975
PPR1	YGL122C	0.107197	1.79972	2.43715						4.34407
PPR1	YGL187C			4.07208						4.07208
PPR1	YGR125W		1.21508	4.1275						5.34258
PPR1	YIR034C			4.43039						4.43039
PPR1	YKL216W		3.08321	10.6693						13.7525
PPR1	YLR002C		1.51519	6.34761	0.577937					8.44074
PPR1	YLR419W	2.85983		2.30576						5.16559
PPR1	YOL164W-A	4.00732								4.00732
PPR1	YOL165C	4.00732								4.00732
PPR1	YOR094W	3.44614	0.562527							4.00867
PPR1	YOR237W			9.55906						9.55906
PPR1	YOR245C			5.50744						5.50744
PPR1	YPL177C		1.79972	3.94063						5.74035
PUT3	YBL063W	1.98193	1.64121		0.520896					4.14404
PUT3	YBR015C	0.0654848	1.90168	2.57246						4.53962
PUT3	YBR016W	0.0654848	1.90168	2.88083						4.84799
PUT3	YBR132C		1.90168	6.56749						8.46917
PUT3	YCL040W		1.90168	3.35241						5.25409
PUT3	YCL043C		1.90168	3.13031						5.03199
PUT3	YCL064C	0.96312	0.778041	2.58161						4.32277
PUT3	YDL022W	2.05271	1.90168	3.84796						7.80235
PUT3	YDR079W	5.0022								5.0022
PUT3	YDR540C		1.64121	3.06412						4.70533
PUT3	YER017C	0.581592	1.36766	2.52727						4.47652
PUT3	YER019W	0.581592	1.36766	3.21979						5.16904
PUT3	YER073W				1.97407			3.06853		5.0426
PUT3	YER145C		1.36766	2.98106						4.34872
PUT3	YFR017C		1.36766	4.58242						5.95008
PUT3	YFR033C	0.992505	0.113903	9.49264						10.599
PUT3	YGL127C		2.15071	2.30366						4.45437
PUT3	YGL253W	6.20727		0.0944496						6.30172
PUT3	YGL254W	6.20727								6.20727
PUT3	YGR202C		0.113903	2.30045			1.80529			4.21964
PUT3	YHL016C	2.68079			2.15534					4.83613
PUT3	YHR037W	11.0516	1.08079	0.837689				3.06853		16.0386
PUT3	YHR057C			4.97521						4.97521
PUT3	YHR059W			4.03605						4.03605
PUT3	YHR080C		1.08079	2.98226						4.06305
PUT3	YHR081W		1.08079	3.97699	0.492408					5.55019
PUT3	YIL133C	4.27266	1.08079		0.575966					5.92942
PUT3	YJL042W		0.571749	2.42164	2.39899					5.39238
PUT3	YJR016C		1.64121	2.88499						4.5262
PUT3	YJR046W		1.90168	2.67678						4.57846
PUT3	YJR121W		1.08079	2.52705		0.435708				4.04355
PUT3	YKL187C			8.95641	0.340478					9.29689
PUT3	YKL217W	0.0666162	1.64121	0.79548	2.47756					4.98087
PUT3	YKR066C		0.240351	4.25904						4.49939
PUT3	YKR067W		0.240351	4.02965						4.27

TF	Target	Binding_0-2000	Motif_Score_SC	Other_Yeasts	Two-Hop_BE	Two-Hop_BP	Two-Hop_BY	Two-Hop_BG	Max_Membership	Sum
PUT3	YLR055C	0.910488	1.90168	4.89435		0.517149				8.22367
PUT3	YLR056W	0.910488	1.90168	5.19126	0.46746					8.47089
PUT3	YLR139C	13.9261	1.36766	2.24955						17.5433
PUT3	YLR142W	13.9261	1.36766	2.90196				2.55055		20.7463
PUT3	YLR237W			4.45506						4.45506
PUT3	YLR258W	0.68159	0.452558	4.93865						6.0728
PUT3	YLR312C	2.46727	1.64121	4.91816	0.512056					9.5387
PUT3	YLR312W-A	2.46727	1.64121	4.36925						8.47773
PUT3	YLR401C			4.22881						4.22881
PUT3	YLR437C			8.73794						8.73794
PUT3	YLR438W			10.6527						10.6527
PUT3	YML058W	0.584024	1.64121	2.86416		0.352711				5.44211
PUT3	YML082W		1.64121	2.85444						4.49565
PUT3	YMR037C		1.64121	2.54908						4.19029
PUT3	YNL147W	0.210996	1.90168	2.59115						4.70383
PUT3	YNL149C	0.210996	1.90168	4.81446						6.92714
PUT3	YNL196C		1.36766	3.70601						5.07367
PUT3	YNL207W	0.355485	0.113903	4.08608						4.55547
PUT3	YOR140W		0.113903	4.31683						4.43073
PUT3	YOR161C		0.113903	5.04986						5.16376
PUT3	YOR267C	0.261456	2.15071	3.10618						5.51835
PUT3	YOR269W	0.261456	2.15071	7.10227						9.51444
PUT3	YOR302W	2.68127	1.90168							4.58295
PUT3	YOR303W	2.68127	1.90168							4.58295
PUT3	YOR306C	7.6494	1.64121	2.74512						12.0357
PUT3	YPL088W		1.64121	2.47249						4.1137
PUT3	YPL230W			4.12506						4.12506
PUT3	YPR040W	2.74488	1.64121							4.38609
PUT3	YPR065W			6.15548	2.11011					8.26559
PUT3	YPR148C	0.918354	1.64121	3.89768						6.45724
PUT3	YPR149W	0.918354	1.64121	3.49664						6.0562
RAP1	YAL024C			6.07653						6.07653
RAP1	YBL003C		1.24244	2.5957	0.594569				0.541905	4.97461
RAP1	YBL027W	2.15265	1.24244		0.826868				0.731102	4.95306
RAP1	YBL028C	2.15265	1.24244	2.46489	0.663921				0.732035	7.25594
RAP1	YBL072C	1.65473		1.32792	0.918622				0.72958	4.63085
RAP1	YBL087C	2.31807	0.832277	1.71617	0.865994				0.730586	6.4631
RAP1	YBL092W	1.68865	1.24244	0.302168	0.854532				0.732408	4.8202
RAP1	YBL102W			4.67789						4.67789
RAP1	YBL103C			5.13993						5.13993
RAP1	YBR048W	2.37612	1.01641		0.953537				0.732681	5.07875
RAP1	YBR084C-A	2.24793	0.176821		0.864182				0.731401	4.02033
RAP1	YBR117C	1.65672	0.996501	1.05903	0.434847					4.1471
RAP1	YBR118W	1.65672	0.996501	1.16222	0.503273				0.638732	4.95745
RAP1	YBR179C		1.24244	7.88175						9.12419
RAP1	YBR180W		1.24244	5.95167						7.19411
RAP1	YBR181C	2.36983		0.103755	0.881502				0.730727	4.08581
RAP1	YBR191W	2.09732	0.103904	1.38852	0.906583				0.725883	5.22221
RAP1	YCR011C		1.24244	4.79653						6.03897
RAP1	YCR012W		1.24244	5.01731						6.25975
RAP1	YCR069W	0.488219	1.01641	4.51376						6.01839
RAP1	YDL021W		1.01641	3.64679						4.6632
RAP1	YDL035C	1.4343	0.176821	3.16036						4.77148
RAP1	YDL075W	2.37437		1.22134	0.821144				0.72997	5.14682
RAP1	YDL076C	2.37437		2.34469						4.71906
RAP1	YDL082W	2.01924	1.01641		0.819967				0.731111	4.58673
RAP1	YDL083C	2.01924	1.01641	1.29924	0.876696				0.729148	5.94073
RAP1	YDL130W	1.66015	1.01641	0.64578	0.548426	0.434883				4.30565
RAP1	YDL136W	2.01126	0.832277		0.830068				0.732158	4.40576
RAP1	YDL186W	1.94433	1.01641	1.22335						4.18409
RAP1	YDL188C	1.94433	1.01641	1.46755		0.410822				4.83911
RAP1	YDL191W	2.63359	1.01641		0.726842				0.729949	5.10679
RAP1	YDR025W	2.03221	0.996501	0.58926	0.885006				0.732106	5.23508
RAP1	YDR036C	1.39645	1.01641	3.97881						6.39167
RAP1	YDR037W	1.39645	1.01641	6.42261					0.73173	9.5672
RAP1	YDR064W	1.96154	0.832277	0.77472	0.815285				0.732202	5.11602
RAP1	YDR109C	1.11329	0.996501	5.62925						7.73904
RAP1	YDR110W	1.11329	0.996501	6.17529						8.28508
RAP1	YDR111C	1.49535	0.760243	1.70864					0.698667	4.6629
RAP1	YDR185C		1.24244	8.35612					0.674719	10.2733
RAP1	YDR225W			9.81002	0.458655				0.688578	10.9573
RAP1	YDR412W		1.01641	1.64514	0.620941				0.732763	4.01525
RAP1	YDR418W	1.93666	0.832277	0.208569	0.959837				0.71499	4.65233
RAP1	YDR447C	1.7802	1.01641	0.61264	0.770639				0.723762	4.90365
RAP1	YDR448W	1.7802	1.01641	2.42843						5.22504
RAP1	YDR449C	2.5687	0.176821	1.48942	0.720283				0.732725	5.68795
RAP1	YDR450W	2.5687	0.176821	0.229146	0.909133				0.72879	4.61259
RAP1	YDR471W	2.16277	0.257908		0.890252				0.729612	4.04054
RAP1	YEL009C	1.64378	0.760243	2.76835					0.682819	5.85519
RAP1	YEL022W	1.95028	1.24244	9.09969					0.679519	12.9719
RAP1	YEL023C	1.95028	1.24244	6.95448						10.1472
RAP1	YEL034W	1.2375	0.996501	1.89542	0.516046				0.456537	5.102
RAP1	YEL036C	1.2375	0.996501	1.95351						4.18751
RAP1	YEL054C	2.22111	0.257908	0.241333	0.946113	0.541172			0.729125	4.93676
RAP1	YER056C-A	1.61125	1.01641	0.675913	0.844803				0.727253	4.87563
RAP1	YER074W	2.18075	0.257908	0.727112	0.91193				0.731944	4.80964
RAP1	YER101C	1.83782	1.01641	4.14277						6.997
RAP1	YER102W	1.83782	1.01641	2.05106	0.933242				0.732364	6.5709
RAP1	YER116C	2.27368	0.832277	1.45348						4.55944
RAP1	YER117W	2.27368	0.832277	0.192219	0.854213				0.725555	4.87794
RAP1	YER168C	1.91172		7.0115						8.92322
RAP1	YER169W	1.91172		5.92525						7.83697
RAP1	YFR017C		1.24244	2.84726						4.0897
RAP1	YFR031C	2.01792	1.01641	2.78922						5.82355
RAP1	YFR031C-A	2.01792	0.590148	4.44027	0.898134				0.730116	8.67659
RAP1	YFR032C-A	0.395697	1.01641	4.1131	0.560866					6.08607
RAP1	YGL030W	2.52243	0.43811		0.879289				0.73004	4.56987
RAP1	YGL031C	2.52243		0.0676482	0.907272				0.730011	4.22736
RAP1	YGL103W	2.58758	0.257908	1.32535	0.785722				0.723774	5.68033
RAP1	YGL115W	1.71967	0.257908	4.97361						6.95119
RAP1	YGL123W	2.15211	1.01641	2.28747	0.817821				0.732364	7.00618
RAP1	YGL124C	2.15211	1.01641	2.06732						5.23584
RAP1	YGL135W	1.87282	1.01641	0.473473	0.890326				0.731883	4.98491
RAP1	YGL147C	1.79342	0.832277	0.797871	0.936341				0.732443	5.09235
RAP1	YGL189C	2.05091	1.01641	5.40499	0.842563				0.728682	10.0436
RAP1	YGR027C	0.817867		5.0361	0.868052				0.73095	7.45297
RAP1	YGR033C	2.43237	0.832277	1.7292						4.99385
RAP1	YGR034W	2.43237	0.832277	0.493406	0.936252				0.732578	5.42688
RAP1	YGR043C	1.1653	0.996501	3.85335	0.412441					6.42759
RAP1	YGR085C	0.505554	1.01641	4.50408	0.889397				0.730832	7.64627

TF	Target	Binding_0-2000	Motif_Score_SC	Other_Yeasts	Two-Hop_BE	Two-Hop_BP	Two-Hop_BY	Two-Hop_BG	Max_Membership	Sum
RAP1	YGR094W	0.744855	1.01641	4.96074					0.729665	7.45167
RAP1	YGR117C	1.94127	0.760243	2.94395						5.64546
RAP1	YGR118W	1.94127	0.760243		0.90914				0.732026	4.34268
RAP1	YGR148C	2.67419			0.876271				0.73119	4.28165
RAP1	YGR159C		1.01641	6.13132					0.677433	7.82516
RAP1	YGR213C	1.72347	1.24244	9.02026						11.9862
RAP1	YGR214W	1.72347	1.24244		0.960666				0.729189	4.65576
RAP1	YGR258C	1.78887	1.24244	5.76814						8.79945
RAP1	YGR260W	1.78887	1.24244	3.14749	0.401593					6.58039
RAP1	YHL033C	0.381223	0.832277	1.64393	0.847725				0.730565	4.43572
RAP1	YHL036W	1.31256	1.01641	2.15241						4.48138
RAP1	YHL038C	1.31256	1.01641	4.32773						6.6567
RAP1	YHR021C	2.42661	0.176821	2.03117	0.613179				0.729462	5.97724
RAP1	YHR024C			4.13028						4.13028
RAP1	YHR033W	2.08957	1.01641	4.03093						7.13691
RAP1	YHR141C	2.52042	1.01641		0.822748				0.732431	5.09201
RAP1	YHR142W	2.52042	1.01641	0.0968201					0.578254	4.2119
RAP1	YHR203C	2.32823	1.24244	3.08573	0.866098				0.729712	8.25221
RAP1	YHR204W	2.32823	1.24244	4.37865					0.63933	8.58865
RAP1	YIL018W	2.17266	1.24244	2.31792	0.911933				0.730612	7.37556
RAP1	YIL020C	2.17266	1.24244						0.71142	4.12652
RAP1	YIL053W	0.956849	0.996501	2.98385					0.728125	5.66533
RAP1	YIL055C	0.956849	0.996501	2.66651						4.61986
RAP1	YIL070C	1.40477	0.832277	1.8945						4.13155
RAP1	YIL108W		0.832277	4.64352						5.4758
RAP1	YIL148W	2.51185		0.557996	0.82215				0.72265	4.61465
RAP1	YIL149C	2.51185		1.71039						4.22224
RAP1	YIR009W		1.24244	5.96579						7.20823
RAP1	YJL026W	1.51125	0.996501	1.49212	0.430311	0.341755				4.77194
RAP1	YJL136C	2.41878	0.590148		0.908634				0.728163	4.64573
RAP1	YJL177W	2.14951	1.01641	3.11122	0.931328				0.732323	7.94079
RAP1	YJL178C		1.01641	5.88683						6.90324
RAP1	YJL190C	2.26627		4.26211	0.907311				0.731944	8.16764
RAP1	YJR009C	0.257945		3.47659	0.647571				0.532435	4.91454
RAP1	YJR088C			4.64079						4.64079
RAP1	YJR123W	1.55889	1.01641	2.18893	0.917104				0.730495	6.41183
RAP1	YJR145C	1.41786	0.176821	2.43959	0.798085				0.7321	5.56446
RAP1	YJR147W	1.41786	0.996501	3.65862						6.07298
RAP1	YKL006W	2.63419	1.01641		0.868071				0.731492	5.25016
RAP1	YKL060C	1.18344	0.257908	2.39758	0.371016					4.20994
RAP1	YKL081W	1.11438	1.24244	2.93961	0.764744				0.716437	6.77761
RAP1	YKL082C	1.11438	1.24244	4.66447	0.470929				0.730163	8.22238
RAP1	YKR094C	1.53331	1.24244		0.889539				0.732378	4.39767
RAP1	YKR095W	1.53331	1.24244	1.33047						4.10622
RAP1	YLR003C		0.832277	1.68357	0.779731				0.732566	4.02814
RAP1	YLR047C	2.98738	0.176821	1.65738						4.82158
RAP1	YLR048W	2.98738	0.176821		0.920987				0.729935	4.81512
RAP1	YLR312C	1.18799	1.01641	2.28547	0.534683					5.02455
RAP1	YLR325C	1.91379	0.257908	1.67584	0.928581				0.725651	5.50177
RAP1	YLR333C	2.26015		0.501239	0.738427				0.718784	4.2186
RAP1	YLR344W	2.04684	1.01641	0.849584	0.93055				0.730782	5.57417
RAP1	YLR372W		0.590148	2.19257	0.733983				0.732009	4.24871
RAP1	YLR388W	2.52475			0.88121				0.7297	4.13566
RAP1	YLR441C	1.32439	0.996501	1.4406	0.930637				0.73224	5.42437
RAP1	YLR447C	2.83583	1.01641	1.77337					0.688311	6.31392
RAP1	YLR448W	2.83583	1.01641	1.01716	0.951945				0.728588	6.54993
RAP1	YML024W	2.56481		0.053443	0.938197				0.730225	4.28667
RAP1	YML063W	2.40194			0.909095				0.730694	4.04173
RAP1	YML073C	2.51986			0.864112				0.728154	4.11213
RAP1	YMR014W	0.563481	1.24244	8.44395	0.752331				0.731704	11.7339
RAP1	YMR142C	2.43429	1.01641		0.877834				0.730592	5.05913
RAP1	YMR144W	1.54096	1.01641	2.85331					0.509001	5.91968
RAP1	YMR146C		1.01641	2.86825	0.39511				0.732739	5.01251
RAP1	YMR147W		1.01641	3.08689						4.1033
RAP1	YMR224C	1.65696	0.832277	1.3	0.711673					4.50091
RAP1	YMR266W	1.46042	0.103904	3.69168						5.256
RAP1	YNL069C	2.71999	0.567447		0.845334				0.731454	4.86423
RAP1	YNL096C	2.64142			0.882893				0.732584	4.2569
RAP1	YNL162W	2.85147		2.03805	0.898605				0.7309	6.51903
RAP1	YNL163C	2.85147		2.06749					0.731601	5.65056
RAP1	YNL165W	0.840618	0.996501	3.26483						5.10195
RAP1	YNL166C	0.840618	0.996501	3.76703						5.60415
RAP1	YNL302C	2.31348	0.760243		0.689955				0.730777	4.49446
RAP1	YNL327W	2.28766	1.24244						0.711625	4.24172
RAP1	YNL329C	2.28766	1.24244	1.36372						4.89382
RAP1	YOL039W	2.61484		3.62143	0.699055	0.434883			0.732698	8.10291
RAP1	YOL040C	2.61484		3.56639	0.872026				0.728934	7.78219
RAP1	YOL052C-A	1.97599	1.24244	9.4445						12.6629
RAP1	YOL059W	2.31818	0.43811	4.40696						7.16325
RAP1	YOL060C	2.31818	0.43811	3.57416						6.33045
RAP1	YOL086C	0.899129	1.01641	2.82688	0.429092	0.347946				5.51946
RAP1	YOL109W	1.9698	0.568062	1.92172	0.5116					4.97118
RAP1	YOL120C	1.8567	0.176821	0.667164	0.915606				0.732047	4.34834
RAP1	YOL121C	1.8567	0.760243	0.21995	0.922596				0.722269	4.48176
RAP1	YOL127W	2.7528	0.590148	1.34856	0.918759				0.717543	6.32781
RAP1	YOL128C	2.7528	0.590148	2.41875						5.7617
RAP1	YOR043W	0.190448	0.832277	3.86669						4.88942
RAP1	YOR056C	1.21556	0.833451	4.58349	0.41445				0.731352	7.7783
RAP1	YOR057W	1.21556	0.833451	5.32188						7.37089
RAP1	YOR095C	2.10442	1.01641	4.89859	0.437008				0.730202	9.18663
RAP1	YOR096W	2.10442	1.01641	1.6233	0.915239				0.730703	6.39007
RAP1	YOR234C	2.21911	1.01641	0.477967	0.810865				0.728391	5.25274
RAP1	YOR292C	2.85652	1.01641	2.65633						6.52926
RAP1	YOR293W	2.85652	1.01641	0.386706	0.878609				0.732032	5.87028
RAP1	YOR294W	2.85652	1.01641	5.8429	0.661489	0.601354			0.731164	11.7098
RAP1	YOR295W	0.270393		6.81086					0.73239	7.81364
RAP1	YOR311C	2.18629	0.832277	11.3417						14.3603
RAP1	YOR312C	2.18629	0.590148		0.843456				0.730976	4.35087
RAP1	YOR340C	0.325426	1.01641	1.60569	0.41355				0.698254	4.05933
RAP1	YOR344C	2.31424		3.20886					0.610278	6.13338
RAP1	YPL016W	2.67226	0.996501	1.1468						4.81556
RAP1	YPL017C	2.67226	0.996501	2.65426						6.32302
RAP1	YPL036W		0.832277	9.30564						10.1379
RAP1	YPL037C		0.832277	7.78643	0.572242				0.641305	9.83225
RAP1	YPL090C	1.89717	1.01641		0.898343				0.732003	4.54393
RAP1	YPL131W	1.93335		1.40182	0.71841				0.730645	4.78423
RAP1	YPL162C	1.40524	1.01641	2.29228						4.71393
RAP1	YPL249C-A	2.19142	0.590148	1.69777	0.496903					4.97624
RAP1	YPR057W	0.178708	1.24244	7.63661						9.05776
RAP1	YPR102C	2.83778	0.760243	0.0520559	0.844092	0.606415			0.730832	5.83142

TF	Target	Binding_0-2000	Motif_Score_SC	Other_Yeasts	Two-Hop_BE	Two-Hop_BP	Two-Hop_BY	Two-Hop_BG	Max_Membership	Sum
RAP1	YPR103W	2.83778	0.760243	0.984332						4.58235
RAP1	YPR105C	1.19833	1.01641	2.68017						4.89491
RAP1	YPR106W	1.19833	1.01641	1.95162						4.16636
RAP1	YPR110C	0.828965	1.01641	3.70565	0.69755				0.732402	6.98098
RAP1	YPR131C	2.56699	1.01641	2.46492						6.04832
RAP1	YPR132W	2.56699	1.01641		0.860731				0.732361	5.17649
RAP1	YPR161C	1.27284	1.01641	2.77296						5.06221
RCO1	YAL062W	4.42872								4.42872
RCS1	SNR128	4.18985								4.18985
RCS1	SNR190	4.18985								4.18985
RCS1	TT(AGU)C	4.40539								4.40539
RCS1	TW(CCA)J	4.86029								4.86029
RCS1	YAL019W	0.0565523		4.41411						4.47066
RCS1	YAL020C			5.70922						5.70922
RCS1	YBR008C				2.16643		0.954139	1.51646	0.698782	5.33581
RCS1	YBR041W	3.08005					1.35827			4.43832
RCS1	YBR046C	1.61242	0.0381421	5.52301			1.39608			8.56965
RCS1	YBR047W	0.370459		4.7871						5.15756
RCS1	YBR048W	1.61242	0.0381421		1.3226		1.0276			4.00076
RCS1	YBR102C	4.1743				0.346489				4.52079
RCS1	YBR103W	4.1743					1.30057			5.47487
RCS1	YBR106W	1.35061	0.221432	2.08966	0.742597					4.4043
RCS1	YBR109C	1.91318				1.23556	1.20312			4.35186
RCS1	YBR162C		0.131856	4.4225						4.55436
RCS1	YBR163W		0.131856	6.04269						6.17455
RCS1	YBR166C	2.77992	0.131856		1.36424					4.27602
RCS1	YBR198C				1.56445		1.04361	1.58444		4.1925
RCS1	YBR204C	6.29705	0.221432		0.683485					7.20197
RCS1	YBR207W	6.29705	0.221432	0.805136						7.32362
RCS1	YBR217W	0.741225		0.00497938	0.343446	3.33393				4.42358
RCS1	YBR247C			0.388256	1.84986	2.10344			0.61988	4.96144
RCS1	YCL039W	0.209659		1.11787			1.31229	1.77065		4.41047
RCS1	YCL059C				1.60815	2.10344	1.07671		0.532103	5.3204
RCS1	YCL067C	2.23344			0.680279		1.15126			4.06498
RCS1	YCR021C	2.18755	0.627985	1.11998					0.592294	4.52781
RCS1	YCR057C			1.11559	0.898744	2.10344	1.29643	1.60123	0.613608	7.62904
RCS1	YCR066W		0.131856	1.46038			1.1814	1.77606		4.5497
RCS1	YCR096C	4.40539				1.02454	1.1814			6.61133
RCS1	YCR097W	4.40539				1.02454	1.22726			6.65719
RCS1	YCR102C				2.2218		1.38327		0.731563	4.33663
RCS1	YCR105W	10.3377								10.3377
RCS1	YCR107W	11.3597			0.465553				0.729735	12.555
RCS1	YDL195W			1.32978		1.22999	1.21461	1.66207		5.43645
RCS1	YDR084C	4.23186	0.627985							4.85984
RCS1	YDR098C	0.389745		2.79278		3.15041				6.33293
RCS1	YDR151C	8.42931								8.42931
RCS1	YDR152W	8.42931			1.34217					9.77148
RCS1	YDR246W	1.0486		3.11017						4.15877
RCS1	YDR264C	8.65613			1.45879		1.19247			11.3074
RCS1	YDR265W	8.65613					1.22791	1.7406		11.6246
RCS1	YDR270W	9.0793	0.809349	6.19289		0.887403			0.588008	17.5569
RCS1	YDR320C			2.90153			1.20305			4.10458
RCS1	YDR321W			3.37303	0.694962			1.94798		6.01597
RCS1	YDR324C				2.08814		1.24888		0.707465	4.04448
RCS1	YDR534C	1.44442		1.83178	1.1911				0.62252	5.08982
RCS1	YEL058W	4.83501	0.809349	3.701						9.34536
RCS1	YEL060C	4.83501	0.809349	10.2245					0.731258	16.6001
RCS1	YEL065W	8.17305	0.645597	8.90227	1.99827					19.7192
RCS1	YER006W			2.80926	1.10161				0.716536	4.62741
RCS1	YER009W			6.15032						6.15032
RCS1	YER075C	0.0599228		0.7367		3.33393				4.13055
RCS1	YER109C			1.34438			1.08895	1.70085		4.13418
RCS1	YER117W	0.074772			0.677309	3.33393				4.08601
RCS1	YER145C	7.35621	0.627985	6.43284	1.42407				0.638922	16.48
RCS1	YER146W	7.35621	0.627985	5.61943						13.6036
RCS1	YER175C	1.38098		2.98297						4.36395
RCS1	YFL041W		0.221432	3.1196	0.666942	1.34467				5.35264
RCS1	YFR001W	4.97565			1.85027					6.82592
RCS1	YFR002W	4.97565								4.97565
RCS1	YFR024C-A	2.83471			1.4391			2.07375		6.34756
RCS1	YFR031C-A			2.35704	1.34704	1.22999				4.93407
RCS1	YGL003C					1.27543	1.3087	1.6617		4.24583
RCS1	YGL006W			3.92203	1.0458					4.96783
RCS1	YGL019W			7.52018						7.52018
RCS1	YGL071W	13.9261	0.809349	11.3734						26.1088
RCS1	YGL135W			7.28717	0.796437		1.00946			9.09307
RCS1	YGL136C			7.4296						7.4296
RCS1	YGL145W		0.645597	3.43178						4.07738
RCS1	YGL181W	3.50982					1.03353	1.65584		6.19919
RCS1	YGR009C	6.7092								6.7092
RCS1	YGR010W	6.7092								6.7092
RCS1	YGR040W	0.204723		0.662865		3.33393				4.20152
RCS1	YGR123C				1.40135		1.11096	1.17062	0.611668	4.2946
RCS1	YGR175C	2.88575			0.33655		1.13067			4.35297
RCS1	YGR238C	4.45423		0.261887			1.16511	0.932767		6.81399
RCS1	YGR248W			6.98939	0.416165					7.40556
RCS1	YGR257C	4.25631		3.1106						7.36691
RCS1	YHL010C	0.24755				2.35265	1.21969	1.19908		5.01897
RCS1	YHL013C	2.48401			1.39783		1.16425			5.04609
RCS1	YHL035C	2.0174	0.0320644	5.32545	1.74667				0.729606	9.85119
RCS1	YHL039W	6.93264	0.627985	6.03477	1.06529					14.6607
RCS1	YHL040C	6.93264	0.627985	1.81087	2.08607				0.726446	12.184
RCS1	YHL047C	7.68839	0.809349		2.2989				0.717211	11.5138
RCS1	YHR088W	1.52585		1.52235	1.21381		1.10716		0.632078	6.00125
RCS1	YIL004C	3.18158			1.72751					4.90909
RCS1	YIL009W		0.645597	5.14963	0.449563					6.24479
RCS1	YIL057C	1.72522	0.627985	4.45297	0.663612				0.62947	8.09926
RCS1	YIL061C	0.829823				3.33393				4.16375
RCS1	YIL153W	0.435925	0.221432	2.62336	0.882945					4.16366
RCS1	YJL069C	0.874689		0.38443	1.29581		1.19896		0.617902	4.37179
RCS1	YJL128C			0.988751		3.33393				4.32268
RCS1	YJL164C					3.33393			0.71839	4.05232
RCS1	YJL200C	3.05547		0.0361853	0.888686				0.570016	4.55036
RCS1	YKL001C	4.19154								4.19154
RCS1	YKL005C		0.627985	2.29506			1.13921			4.06226
RCS1	YKL039W	0.380263	0.645597	9.46926						10.4951
RCS1	YKL040C	0.380263	0.645597	9.57898						10.6048
RCS1	YKL099C	2.25392			1.70837				0.702337	4.66463
RCS1	YKL101W	5.33477								5.33477
RCS1	YKL103C	5.33477			0.985898				0.731067	7.05173

TF	Target	Binding_0-2000	Motif_Score_SC	Other_Yeasts	Two-Hop_BE	Two-Hop_BP	Two-Hop_BY	Two-Hop_BG	Max_Membership	Sum
RCS1	YKL178C	0.0579204		1.77273		2.17204				4.00269
RCS1	YKL191W			6.78189						6.78189
RCS1	YKL192C		0.0198892	6.46689						6.48678
RCS1	YKL193C		0.0198892	6.46017	0.779856	0.546896	0.922294			8.72911
RCS1	YKL213C			0.399872	0.966243		1.30212	1.64327		4.31151
RCS1	YKR052C	6.80877			0.627311					7.43608
RCS1	YLL008W	3.10914		0.478725	1.72842				0.65815	5.97443
RCS1	YLL011W	9.10783			1.00618		1.17488	1.6592	0.499921	13.448
RCS1	YLL049W	5.18709								5.18709
RCS1	YLL051C	5.18709		1.28201	1.93596	0.351756				8.75682
RCS1	YLR002C	2.93159			1.2835				0.53489	4.74998
RCS1	YLR034C	0.173829	0.221432	3.4026	1.74357				0.730642	6.27207
RCS1	YLR061W	4.19805			0.348024					4.54607
RCS1	YLR063W	4.19805								4.19805
RCS1	YLR108C	7.38825							0.712379	8.10063
RCS1	YLR109W	7.38825		0.452972	0.921875				0.732701	9.4958
RCS1	YLR129W				2.14053		1.27414		0.67173	4.0864
RCS1	YLR136C	12.2201	0.627985	6.79427	1.37319				0.715436	21.731
RCS1	YLR137W	12.2201	0.627985	3.3824						16.2305
RCS1	YLR138W	12.2201	0.627985							12.8481
RCS1	YLR196W	0.949958			1.6942		1.31229		0.643558	4.60001
RCS1	YLR206W	5.89836			0.430107					6.32847
RCS1	YLR213C	5.92479		0.497748						6.42254
RCS1	YLR214W	5.92479		0.509429	2.2691				0.610269	9.31359
RCS1	YLR222C				2.03841	3.33393	1.29643		0.643399	7.31217
RCS1	YLR254C	1.47871		5.18653			0.796768	1.65875		9.12076
RCS1	YLR397C	6.14993							0.612798	6.76273
RCS1	YLR399C			3.2873				0.862185		4.14949
RCS1	YLR409C	0.364443			1.744		1.32774		0.706412	4.1426
RCS1	YLR423C			2.90102		0.936134	0.895212	1.21854		5.95091
RCS1	YLR438C-A	1.61112				1.82114	1.12075			4.55301
RCS1	YLR460C	0.134468			2.0489		1.38428		0.731604	4.29925
RCS1	YML116W	2.10851					0.952206	1.27088	0.587553	4.91915
RCS1	YMR016C	4.28964								4.28964
RCS1	YMR017W	4.28964								4.28964
RCS1	YMR023C	0.123586		3.23576			0.875743			4.23509
RCS1	YMR041C	2.02142		0.103755	0.434912		1.59539	0.864447		5.01992
RCS1	YMR043W	2.02142				1.02454	1.60664			4.6526
RCS1	YMR055C	10.1695	0.0320644		0.98669					11.1883
RCS1	YMR056C	1.815	0.0320644	1.80654	0.622819					4.27642
RCS1	YMR058W	10.1695	0.0320644	3.39041	1.53327					15.1252
RCS1	YMR131C				1.15854		1.32057	1.68884	0.670554	4.8385
RCS1	YMR251W	6.06415			0.836906					6.90106
RCS1	YMR252C	5.15218								5.15218
RCS1	YMR253C	5.15218								5.15218
RCS1	YMR255W	5.15218								5.15218
RCS1	YMR319C	15.8551	0.645597	5.17618						21.6769
RCS1	YNL002C	1.19874			1.72868		1.08928		0.724947	4.74165
RCS1	YNL061W				1.72239	3.33393			0.703126	5.75945
RCS1	YNL082W	0.210423	0.809349	4.91258						5.93235
RCS1	YNL112W	5.87225			1.12012				0.686815	7.67919
RCS1	YNL115C	5.87225							0.726124	6.59837
RCS1	YNL207W			3.24374			1.03647			4.28021
RCS1	YNL241C	5.46544		0.517371					0.445131	6.42794
RCS1	YNL259C	3.89739		1.22056		2.28627				7.40422
RCS1	YNL260C	3.89739		1.16061						5.058
RCS1	YNL300W			5.61475						5.61475
RCS1	YNR060W	7.61584		1.33349						8.94933
RCS1	YOL091W			3.01807		0.546896	0.960125	1.66163		6.18672
RCS1	YOL158C	6.8982	0.645597	2.8432	2.18855				0.667074	13.2426
RCS1	YOL165C	1.69452			2.43988			0.923352	0.729896	5.78765
RCS1	YOR101W					1.50406	1.5137	1.19113		4.20889
RCS1	YOR316C	3.95571			0.882458					4.83817
RCS1	YOR381W	2.86794	0.627985	4.82616						8.32208
RCS1	YOR382W	4.12743	0.809349	9.38776	2.39626				0.725161	17.446
RCS1	YOR383C	5.70196		1.10504	0.938084				0.693824	8.43891
RCS1	YOR384W	5.70196		1.41195						7.11391
RCS1	YPL019C	3.96488						1.69274		5.65762
RCS1	YPL043W	0.263173			1.69075	0.48408	1.15926		0.670715	4.26798
RCS1	YPL098C			4.54565						4.54565
RCS1	YPL135W	2.6791		1.2035					0.732675	4.61528
RCS1	YPL156C	2.80235	0.221432	2.87355						5.89733
RCS1	YPL177C	1.09028	0.627985	4.053	0.798322		1.21997			7.78956
RCS1	YPL253C	6.13058		1.81365						7.94423
RCS1	YPR048W			6.8641	1.22917					8.09327
RCS1	YPR049C			5.2395			0.951189			6.19069
RCS1	YPR124W	3.13717			0.926836					4.06401
RCS1	YPR137W	1.01924			1.38445		1.31348		0.686595	4.40376
RCS1	YPR141C	1.08887		0.47047		1.49026	1.23558	1.29185		5.57703
RCS1	YPR151C	2.43925	0.221432	2.80439	0.789679				0.468454	6.72321
RCS1	YPR178W			0.788164		1.6306	1.04361	1.59644		5.05881
RDS1	TL(CAA)G1	4.11773								4.11773
RDS1	YBR008C			3.23001	2.32305		1.1335			6.68656
RDS1	YBR015C	2.23506	1.16053	1.6606						5.05619
RDS1	YBR016W	2.23506	1.16053	1.78686						5.18245
RDS1	YBR063C		1.44612	7.43388						8.88
RDS1	YBR166C	4.25192								4.25192
RDS1	YBR167C	4.25192								4.25192
RDS1	YBR168W	4.25192								4.25192
RDS1	YBR182C	0.753571	1.44612	4.10732						6.30701
RDS1	YBR183W	0.753571	1.44612	5.3414						7.54109
RDS1	YCR102C	0.974907		0.653594	2.37844		1.47331			5.48025
RDS1	YCR105W	10.78	1.44612	5.69661	0.472936		0.994205			19.3899
RDS1	YCR106W	5.81209		1.49892						7.31101
RDS1	YCR107W	12.1264	1.44612		0.44562		1.09254			15.1107
RDS1	YDL078C		0.664967	3.59412						4.25909
RDS1	YDL178W	0.251938	1.44612	6.1334			1.43807			9.26953
RDS1	YDR074W		1.16053	2.72321	1.33823					5.22197
RDS1	YDR256C			7.33327						7.33327
RDS1	YDR328C			4.00365						4.00365
RDS1	YDR372C	4.07003								4.07003
RDS1	YDR373W	4.07003								4.07003
RDS1	YDR400W			8.49644						8.49644
RDS1	YDR530C		1.44612	7.30749						8.75361
RDS1	YDR531W		1.44612	9.3804						10.8265
RDS1	YER043C	3.11519	1.44612	0.160999					0.730876	5.45319
RDS1	YER044C	3.11519		0.963928	1.65308				0.695528	6.42773
RDS1	YER133W	1.94993			0.672675	0.347758	1.09662			4.06698
RDS1	YER170W		0.664967	5.32012						5.98509
RDS1	YFL007W	3.60258	1.16053							4.76311

TF	Target	Binding_0-2000	Motif_Score_SC	Other_Yeasts	Two-Hop_BE	Two-Hop_BP	Two-Hop_BY	Two-Hop_BG	Max_Membership	Sum
RDS1	YFL023W	2.79506	1.16053	1.66985						5.62544
RDS1	YFL024C	2.79506	1.16053	2.80012						6.75571
RDS1	YFL055W	0.591835		1.21771	1.45118		1.27963			4.54035
RDS1	YFL056C	0.591835			2.39417		1.08288			4.06888
RDS1	YFR033C	0.731588	1.16053	4.12146						6.01358
RDS1	YGL156W	7.98352	1.44612		1.278					10.7076
RDS1	YGL157W	7.98352	1.44612	6.14142	1.278					16.8491
RDS1	YGR100W	2.53657	1.44612	3.29599						7.27868
RDS1	YGR175C	7.86409	1.44612	4.44489	0.683005				0.698268	15.1364
RDS1	YKL217W		0.503769	1.75149	1.28985		0.951782			4.49689
RDS1	YLR460C	0.375539			2.20552		1.47431			4.05537
RDS1	YMR060C			8.51234	0.350987					8.86333
RDS1	YMR102C	8.45993	1.44612							9.90605
RDS1	YMR137C			4.04035						4.04035
RDS1	YMR228W		1.44612	2.9834						4.42952
RDS1	YNL149C	0.602446	1.16053	2.81196						4.57494
RDS1	YNL241C	2.06329	1.16053	1.32831						4.55213
RDS1	YNL305C	0.883829	1.16053	2.01711						4.06147
RDS1	YNR043W		1.16053	0.673106	1.67694				0.731437	4.24201
RDS1	YOL109W			6.96186						6.96186
RDS1	YOL111C			5.47147						5.47147
RDS1	YOL125W	2.9207	0.503769	0.900136						4.32461
RDS1	YOL165C	1.59694			2.59652		0.96739			5.16085
RDS1	YOR316C	3.48797	0.0932193	1.226						4.80719
RDS1	YOR317W	3.48797	0.0932193	0.905425	1.13493				0.481899	6.10344
RDS1	YPL088W		1.16053	5.47057			1.30836			7.93946
RDS1	YPL139C		1.44612	7.50551						8.95163
RDS1	YPL229W			5.10459						5.10459
RDS1	YPR065W	1.37423	1.44612	6.24273						9.06308
RDS1	YPR127W	7.08814	1.16053	4.60783	1.22709		1.68407			15.7677
REB1	SNR189	6.14198	0.648627							6.79061
REB1	TP(UGG)H	3.68096	0.648627							4.32959
REB1	YAL030W	3.72743				0.859189				4.58662
REB1	YBL013W	2.40228	0.648627				0.978195			4.0291
REB1	YBL036C	3.09578	0.648627	0.701962						4.44637
REB1	YBL050W	3.18485			0.333131	0.859189				4.37717
REB1	YBL058W	4.84043	0.648627							5.48906
REB1	YBL059C-A	4.84043	0.648627							5.48906
REB1	YBR035C	1.36219	0.648627	2.4645						4.47532
REB1	YBR081C	2.03081	0.648627	0.802614		0.529571		1.02751		5.03913
REB1	YBR143C	1.73803	0.648627	0.0589267	0.766531		0.960608			4.17272
REB1	YBR164C	3.19656	0.648627	2.08029						5.92548
REB1	YBR165W	3.19656	0.648627	0.878809						4.724
REB1	YBR236C	4.00326	0.648627	0.150974		0.53038				5.33324
REB1	YBR237W	4.00326	0.648627	0.408668						5.06055
REB1	YCR031C	6.14198	0.648627		0.403274		0.929923			8.1238
REB1	YCR032W	6.14198	0.648627	2.31047				1.22464		10.3257
REB1	YCR067C	2.37765	0.648627	2.10786	0.587543		0.794881			6.51656
REB1	YCR068W	2.37765	0.648627	1.78253						4.80881
REB1	YCR094W	2.12367	0.648627	1.90942						4.68172
REB1	YCR095C	0.919657	0.648627	3.6503	0.508879					5.72746
REB1	YDL007W	3.9109	0.648627	0.600826	0.545574	0.718159				6.42409
REB1	YDL045W-A	2.56803	0.648627	2.57789						5.79455
REB1	YDL070W	2.46848	0.648627		0.346759			1.02315		4.48702
REB1	YDL085W		0.648627	3.41117						4.0598
REB1	YDL137W	2.3712	0.648627	0.552007			0.853828			4.42566
REB1	YDL140C	2.57486	0.648627			0.915982				4.13947
REB1	YDL185W	3.90663	0.648627			0.427566				4.98282
REB1	YDL186W	3.90663	0.648627	1.23308						5.78834
REB1	YDL188C	3.90663	0.648627	1.26798	0.335755	0.452521				6.61151
REB1	YDL207W	1.98581	0.648627	3.46843						6.10287
REB1	YDL208W	1.98581	0.648627	2.85728	0.608845		0.979697			7.08026
REB1	YDL209C	1.98581	0.648627	2.16909		0.758126				5.56165
REB1	YDL212W	3.01123	0.648627	2.13591						5.79577
REB1	YDL213C	3.01123	0.648627	1.92316	0.377355					5.96037
REB1	YDL214C	3.01123	0.648627	0.381201						4.04106
REB1	YDL225W	3.10887	0.648627	1.52963						5.28713
REB1	YDL226C	3.10887	0.648627	1.17964		0.57141		1.26859		6.77714
REB1	YDR003W	2.70509	0.648627	1.05145						4.40517
REB1	YDR004W	2.70509	0.648627				0.981491			4.33521
REB1	YDR028C	4.1754	0.648627	0.265876			0.833987			5.92389
REB1	YDR036C	2.54716	0.648627			1.02589				4.22168
REB1	YDR101C	1.02253	0.648627	4.59879	1.00017	0.664614				7.93473
REB1	YDR103W	1.02253	0.648627	1.75752	0.538512	0.619561				4.58675
REB1	YDR144C	0.467754	0.648627	3.34712			0.881154			5.34465
REB1	YDR153C	2.72713	0.648627		0.679989					4.05575
REB1	YDR155C	2.72713	0.648627	3.07057		0.780226				7.22655
REB1	YDR156W	2.72713	0.648627	2.51151	0.758735					6.646
REB1	YDR174W	2.49883	0.648627	2.22868						5.37614
REB1	YDR190C	1.93945	0.648627	4.02709	0.469837		0.829024			7.91403
REB1	YDR191W	1.93945	0.648627	4.44527						7.03335
REB1	YDR219C	1.49524	0.648627	2.60232						4.74619
REB1	YDR221W	1.49524	0.648627	2.42038			0.788779			5.35303
REB1	YDR257C	3.65578	0.648627	0.206847						4.51125
REB1	YDR313C	3.70068	0.648627	0.664556			0.800077	1.66456		7.4785
REB1	YDR323C	4.93314								4.93314
REB1	YDR411C		0.648627	5.0233						5.67193
REB1	YDR412W		0.648627	4.37622	0.824812	0.489899				6.33956
REB1	YDR465C	4.53801	0.648627	0.534361	0.639743					6.36074
REB1	YDR466W	4.53801	0.648627	0.534239						5.72088
REB1	YDR468C	3.79287				0.841522				4.63439
REB1	YEL011W	2.99262	0.648627		0.678361					4.31961
REB1	YEL022W	1.96685	0.648627	2.11504						4.73052
REB1	YEL023C	1.96685	0.648627	2.08401						4.69949
REB1	YEL026W	1.93529	0.648627	2.52047	0.607251		0.977435			6.68907
REB1	YEL027W	1.93529	0.648627	2.34694						4.93086
REB1	YEL051W	0.107508	0.648627	2.60724		0.699365	0.857786			4.92053
REB1	YER013W	4.67237	0.648627	0.56183			0.886302			6.76913
REB1	YER021W	0.497209	0.648627	1.9057	0.424388	0.527626				4.00355
REB1	YER039C	2.69244	0.648627					1.00028		4.34135
REB1	YER039C-A	2.69244	0.648627					1.00028		4.34135
REB1	YER048W-A	3.78197	0.648627	1.62948						6.06008
REB1	YER049W	3.78197	0.648627		0.83864					5.26924
REB1	YER068W	2.43898	0.648627	1.36624		0.55243				5.00628
REB1	YER091C		0.648627	4.80955						5.45818
REB1	YER105C	3.31083	0.648627	2.91201						6.87147
REB1	YER106W	3.31083	0.648627	2.99358						6.95304
REB1	YER111C	0.458101	0.648627	3.81612		0.934195	0.780118			6.63716
REB1	YER112W	0.458101	0.648627	4.47131						5.57804
REB1	YER157W	3.9179				0.445595				4.3635

TF	Target	Binding_0-2000	Motif_Score_SC	Other_Yeasts	Two-Hop_BE	Two-Hop_BP	Two-Hop_BY	Two-Hop_BG	Max_Membership	Sum
REB1	YFL004W	3.94624	0.648627							4.59487
REB1	YFL005W	3.94624	0.648627	0.803902		0.654787		1.28411		7.33767
REB1	YFL023W	2.44289	0.648627	1.14481	0.383835					4.62016
REB1	YFL024C	2.44289	0.648627	0.942175						4.03369
REB1	YFL039C	0.49212	0.648627	2.76556	0.494452	0.739196				5.13995
REB1	YFR050C	1.69919	0.648627	0.711678	0.473102	0.729058	0.877876			5.13953
REB1	YFR052W	1.54653	0.648627	0.489486	0.899013	0.700517				4.28417
REB1	YGL043W	1.05661	0.648627	1.05342		0.53038	0.977467			4.2665
REB1	YGL068W	2.57073	0.648627	0.882905			0.948084			5.05035
REB1	YGL070C	2.57073	0.648627	1.01028	0.539199	0.727658	0.978986			6.47548
REB1	YGL119W	4.43064	0.648627	2.75464						7.83391
REB1	YGL120C	4.43064	0.648627	2.42755	0.783667	0.758126				9.04861
REB1	YGL154C	2.90944	0.648627		0.484111	0.344658				4.38684
REB1	YGL161C	2.09239	0.648627	1.66677		0.90059				5.30838
REB1	YGL181W	4.02831	0.648627					1.26932		5.94626
REB1	YGL183C	4.02831	0.648627							4.67694
REB1	YGL184C	4.02831	0.648627							4.67694
REB1	YGL198W	3.0357	0.648627			0.90059				4.58492
REB1	YGL200C	3.0357	0.648627			0.948658				4.63298
REB1	YGL237C	2.10834	0.648627	1.37221						4.12918
REB1	YGL246C	1.01861	0.648627	2.43207	0.76594					4.86525
REB1	YGL247W	2.98883	0.648627	2.38478						6.02224
REB1	YGL248W	2.98883	0.648627	0.926313	0.828145					5.39192
REB1	YGR002C	1.42246		2.8213						4.24376
REB1	YGR009C	1.08116	0.648627	1.27012		0.841522	0.812984			4.65441
REB1	YGR012W	2.97044	0.648627	2.99855						6.61762
REB1	YGR013W	2.97044	0.648627			0.758126				4.37719
REB1	YGR026W		0.648627	3.80977						4.4584
REB1	YGR037C	0.984605	0.648627	3.84811	0.469179			1.01809		6.96861
REB1	YGR038W	0.984605	0.648627	3.44998						5.08321
REB1	YGR044C	0.349898	0.648627	4.42763						5.42615
REB1	YGR046W	0.349898	0.648627	5.60575						6.60427
REB1	YGR053C	0.44197	0.648627	4.98177						6.07237
REB1	YGR054W	0.44197	0.648627	7.44396						8.53456
REB1	YGR077C	3.34838	0.648627	0.15307						4.15008
REB1	YGR078C	3.34838	0.648627	1.81701	0.485328					6.29934
REB1	YGR079W	3.34838	0.648627	2.40729						6.4043
REB1	YGR080W	3.34838	0.648627		0.702906					4.69991
REB1	YGR083C	1.72195	0.648627	5.12982	0.501849		0.884387			8.88663
REB1	YGR132C	2.65654	0.648627	2.48024						5.78541
REB1	YGR133W	2.65654	0.648627	2.60908			0.816004			6.73025
REB1	YGR135W	1.86122			0.592936	0.699365	0.981002			4.13452
REB1	YGR141W	1.92415	0.648627	2.7812						5.35398
REB1	YGR145W	1.88905	0.648627	1.68665	1.06527					5.2896
REB1	YGR146C-A	4.27633	0.648627							4.92496
REB1	YGR147C	4.27633	0.648627	4.57884						9.5038
REB1	YGR156W	1.52222	0.648627	1.3468		0.976136				4.49378
REB1	YGR172C	1.74193	0.648627	1.29205		0.654787				4.33739
REB1	YGR173W	1.74193	0.648627	1.32642	0.502512					4.21949
REB1	YGR179C	2.90521	0.648627	1.61491						5.16875
REB1	YGR184C	3.54166	0.648627	1.25212		0.761378				6.20378
REB1	YGR186W	3.54166	0.648627			0.338734				4.52902
REB1	YGR220C	0.697267	0.648627	1.69874		0.840824	0.958501			4.84396
REB1	YGR231C	2.68511	0.648627	1.66706						5.0008
REB1	YGR232W	2.68511	0.648627	1.79374		0.718159				5.84564
REB1	YGR235C	3.37989	0.648627	3.38571						7.41423
REB1	YGR241C	4.06611	0.648627	0.261785						4.97652
REB1	YGR243W	4.06611	0.648627	0.898363	0.485215					6.09832
REB1	YGR285C		0.648627	2.01003	0.731999	0.613497	0.840771			4.84492
REB1	YHL004W		0.648627	1.52216		1.02589	0.954812			4.15149
REB1	YHL031C	2.26078	0.648627	1.21082		0.89294				5.01317
REB1	YHR039C-A	3.95578			0.459476					4.41526
REB1	YHR144C	3.68096	0.648627							4.32959
REB1	YHR146W	3.68096	0.648627	0.222218						4.5518
REB1	YHR170W	3.7779	0.648627	0.749908	0.917004					6.09344
REB1	YIL022W	4.51153	0.648627	0.543733						5.70389
REB1	YIL023C	4.51153	0.648627	0.269435						5.42959
REB1	YIL024C	4.51153	0.648627							5.16016
REB1	YIL075C	3.43055	0.648627	2.53653	0.605092	0.791372				8.01217
REB1	YIL108W	3.39182	0.648627							4.04045
REB1	YIL109C	3.39182	0.648627			0.948658				4.98911
REB1	YIL112W	3.50037	0.648627			0.571483				4.72048
REB1	YIL114C	3.50037	0.648627							4.149
REB1	YIL148W	2.59767	0.648627		0.872009					4.11831
REB1	YIR002C	5.97773	0.648627	0.907682			0.949207			8.48325
REB1	YIR003W	5.97773	0.648627	1.06034						7.6867
REB1	YJL001W	4.17628				0.643054	0.972937			5.79227
REB1	YJL002C	4.17628				0.734617	0.881154			5.79205
REB1	YJL042W	2.29611	0.648627	1.05544						4.00018
REB1	YJL043W	2.29611	0.648627	1.57817						4.52291
REB1	YJL044C	2.29611	0.648627	1.56068						4.50542
REB1	YJL105W	5.06145	0.648627							5.71008
REB1	YJL111W	1.92471	0.648627	3.07363				1.10758		6.75455
REB1	YJL194W	2.94994	0.648627	2.64315		0.527626	0.959071			7.72841
REB1	YJL196C	2.94994	0.648627	2.00526						5.60383
REB1	YJR007W	3.13958	0.648627	0.723941	0.719394		0.96013			6.19167
REB1	YJR008W	3.13958	0.648627		0.351083		0.888074			5.02736
REB1	YJR085C	3.12702	0.648627	0.697437						4.47308
REB1	YJR086W	3.12702	0.648627	0.643929						4.41958
REB1	YJR090C	3.83431	0.648627	0.024418		0.427566				4.93492
REB1	YJR098C	6.19295	0.648627	0.148675						6.99025
REB1	YJR099W	6.19295	0.648627	0.161804						7.00338
REB1	YKL009W	0.823749	0.648627	2.16598	0.789035		0.927985			5.35538
REB1	YKL051W	2.28909	0.648627	1.10544						4.04316
REB1	YKL058W	4.93188	0.648627	1.05158						6.63209
REB1	YKL059C	4.93188	0.648627	0.979415						6.55992
REB1	YKL072W	2.53442		1.25295		0.849556				4.63693
REB1	YKL081W	3.21432			1.00131	0.76021				4.97584
REB1	YKL082C	3.21432			0.478315		0.812919			4.50555
REB1	YKL104C	4.13203	0.648627	1.03762		0.512475				6.33075
REB1	YKL142W	1.69131	0.648627	1.91789	0.62819					4.88602
REB1	YKL145W	3.27045	0.648627	1.06979	0.676399	0.891049				6.55631
REB1	YKL146W	2.30961	0.648627	1.16949	0.364997					4.49272
REB1	YKL148C	2.30961	0.648627	1.13318	1.08961					5.18103
REB1	YKL203C	1.35703	0.648627	1.50926		0.562123				4.07704
REB1	YKR015C	1.95072	0.648627	2.73748						5.33683
REB1	YKR016W	1.95072	0.648627	2.78437						5.38372
REB1	YKR048C		0.648627	1.96877	0.490994	0.934195	0.78157			4.82416
REB1	YKR068C	4.08365	0.648627	1.21772						5.95
REB1	YKR069W	4.08365	0.648627	2.81079						7.54307

TF	Target	Binding_0-2000	Motif_Score_SC	Other_Yeasts	Two-Hop_BE	Two-Hop_BP	Two-Hop_BY	Two-Hop_BG	Max_Membership	Sum
REB1	YLL040C	4.3041	0.648627	0.106704						5.05943
REB1	YLL049W	2.69479	0.648627	2.93788						6.2813
REB1	YLL050C	2.69479	0.648627	3.62842						6.97184
REB1	YLR093C		0.648627	5.22377		0.69505				6.56745
REB1	YLR115W	1.65349	0.648627	0.792374		0.976136				4.07063
REB1	YLR168C	2.42695	0.648627	5.21382						8.2894
REB1	YLR170C	2.42695	0.648627	5.42882						8.5044
REB1	YLR208W		0.648627	4.33085		0.772157				5.75163
REB1	YLR224W	2.59767	0.648627	3.57327						6.81957
REB1	YLR229C	0.751455	0.648627	4.0046						5.40468
REB1	YLR262C	3.29875	0.648627		0.523172	0.654787		1.12728		6.25262
REB1	YLR277C	0.867989	0.648627	1.55075		0.976136	0.929651			4.97315
REB1	YLR298C	0.809207	0.648627	2.16247		0.637168				4.25747
REB1	YLR367W	1.72498	0.648627		1.03572		0.980876			4.3902
REB1	YLR378C	3.29816	0.648627	2.20184	0.868302		0.982448			7.99938
REB1	YLR380W	3.29816	0.648627	2.16114						6.10793
REB1	YLR390W-A	2.01023	0.648627	1.73263						4.39149
REB1	YLR457C	1.22859	0.648627	3.71666	0.822267					6.41614
REB1	YLR459W	1.22859	0.648627	2.56756						4.44478
REB1	YML001W	3.52265	0.648627	2.25558		0.654787		1.28452		8.36616
REB1	YML042W	0.667556	0.648627	5.76608	0.526918					7.60918
REB1	YML043C	0.667556	0.648627	4.75867						6.07485
REB1	YML119W	4.29502	0.648627							4.94365
REB1	YML120C	4.29502	0.648627		0.684039		0.90898			6.53667
REB1	YML129C	4.03721	0.648627	1.15409	0.464977					6.3049
REB1	YML130C	4.03721	0.648627			0.772157				5.45799
REB1	YMR001C	1.52073	0.648627	1.16615	0.727032					4.06254
REB1	YMR002W	1.52073	0.648627	2.44518	0.853012					5.46755
REB1	YMR039C	3.71298	0.648627	1.19202						5.55363
REB1	YMR040W	3.71298	0.648627	1.18603	0.424467					5.9721
REB1	YMR047C	2.49676	0.648627	0.586361		0.948658	0.887922			5.56833
REB1	YMR048W	2.49676	0.648627	1.26802						4.41341
REB1	YMR116C	3.58855	0.648627		0.559082	0.840824				5.63708
REB1	YMR117C	3.58855	0.648627							4.23718
REB1	YMR120C	3.43282	0.648627		0.604631					4.68608
REB1	YMR121C	3.43282	0.648627		0.787266		0.977435			5.84615
REB1	YMR122W-A	3.43282	0.648627							4.08145
REB1	YMR182C	2.755	0.648627	2.5719						5.97553
REB1	YMR183C	0.298927	0.648627	3.76218			0.884503			5.59424
REB1	YMR186W	2.62122	0.648627	1.17276	0.609032					5.05164
REB1	YMR187C	5.11618	0.648627		0.496927					6.26173
REB1	YMR188C	5.11618	0.648627	1.76592	0.465875	0.332369	0.97478			9.30375
REB1	YMR189W	5.11618	0.648627	2.03434						7.79915
REB1	YMR214W	4.03306	0.648627	0.613851	0.359086					5.65462
REB1	YMR215W	4.03306	0.648627							4.68169
REB1	YMR280C	3.01533	0.648627	1.75865						5.42261
REB1	YMR281W	3.01533	0.648627	0.53793						4.20189
REB1	YMR310C	1.91566	0.648627		0.77565		0.803366			4.1433
REB1	YNL090W	3.03152	0.648627	1.77496						5.45511
REB1	YNL091W	5.21815	0.648627	1.53309						7.39987
REB1	YNL112W	3.73724	0.648627		0.452941					4.83881
REB1	YNL113W	3.73724	0.648627		0.60218	0.537126				5.52517
REB1	YNL115C	3.73724	0.648627							4.38587
REB1	YNL161W	3.42683	0.648627		0.411836					4.48729
REB1	YNL169C	4.66245								4.66245
REB1	YNL245C	2.21339	0.648627	1.7934						4.65542
REB1	YNL284C	2.56351	0.648627			0.841629	0.956673			5.01044
REB1	YNL308C	3.13412	0.648627	0.168228	0.767938					4.71891
REB1	YNR011C	6.61323	0.648627				0.789268			8.05113
REB1	YNR012W	6.61323	0.648627				0.789268			8.05113
REB1	YNR015W	2.53112	0.648627				0.899748			4.07949
REB1	YNR039C	3.97147	0.648627	0.698654						5.31875
REB1	YNR040W	3.97147	0.648627	0.600385						5.22048
REB1	YOL004W	3.75719	0.648627	0.158308		0.519462				5.08359
REB1	YOL005C	3.75719	0.648627	1.49965	0.500041	0.53038	0.922061			7.85795
REB1	YOL006C	3.75719	0.648627	0.0430074						4.44882
REB1	YOL031C		0.648627	3.42912	0.375686					4.45343
REB1	YOL124C	1.17714	0.648627		1.4312		0.871194			4.12816
REB1	YOL144W	2.3382	0.648627	1.32585	0.395888	0.509296				5.21786
REB1	YOL145C	2.3382	0.648627	1.2512		0.718159				4.95619
REB1	YOR021C	3.55628	0.648627	0.402365	0.840284					5.44756
REB1	YOR044W	2.8231	0.648627	3.22905						6.70078
REB1	YOR045W	2.8231	0.648627		0.722383					4.19411
REB1	YOR076C	1.6032	0.648627	2.04114						4.29297
REB1	YOR078W	1.6032	0.648627	3.47235						5.72418
REB1	YOR093C	2.35	0.648627	3.29111						6.28974
REB1	YOR094W	2.35	0.648627	3.79639						6.79502
REB1	YOR101W	0.989799	0.648627	1.76341	0.547641			1.28456		5.23404
REB1	YOR116C	3.30564	0.648627	0.0274717		0.653636				4.63537
REB1	YOR117W	3.30564	0.648627	0.799389	0.711637	0.527626				5.99292
REB1	YOR122C	2.62131	0.648627	2.11704						5.38698
REB1	YOR128C	3.28434	0.648627	0.779579						4.71255
REB1	YOR148C	2.69829	0.648627	1.81745						5.16437
REB1	YOR149C	4.27682	0.648627							4.92545
REB1	YOR150W	4.27682	0.648627	1.90636		0.336783	0.966221			8.13481
REB1	YOR151C	2.77125	0.648627			0.72545				4.14533
REB1	YOR157C	2.99223	0.648627	1.17216	0.503309	0.962689	0.979483			7.2585
REB1	YOR158W	2.99223	0.648627	0.41966						4.06052
REB1	YOR185C	4.01787	0.648627	0.287088	0.576424					5.53001
REB1	YOR187W	4.01787	0.648627	0.459655	0.478324					5.60448
REB1	YOR271C	2.98592	0.648627	1.84663	0.448417					5.92959
REB1	YOR272W	2.98592	0.648627	1.28265	1.00705					5.92425
REB1	YOR289W	2.25741	0.648627		0.936039		0.807001			4.64908
REB1	YOR319W	3.71935	0.648627	0.32501			0.840722			5.53371
REB1	YOR335C	5.11969	0.648627	0.540292	0.66221					6.97082
REB1	YOR336W	5.11969	0.648627	0.403281						6.1716
REB1	YOR340C	1.02941	0.648627	1.70327	0.683026					4.06433
REB1	YOR369C		0.648627	2.02038	1.25033		0.915975			4.83531
REB1	YPL160W	0.964514	0.648627	1.12547	0.643225		0.977845			4.35968
REB1	YPL161C	0.964514	0.648627	2.89963	0.447522	0.653658				5.61395
REB1	YPL180W	3.9662	0.648627	1.07391			0.86786			6.5566
REB1	YPL204W	4.43363	0.648627	1.22948		0.336783				6.64852
REB1	YPL206C	4.43363	0.648627	2.98391						8.06617
REB1	YPL211W	2.67999	0.648627	0.910844	0.658817					4.89828
REB1	YPL212C	2.67999	0.648627	0.877617	0.619563					4.8258
REB1	YPL237W	1.23241	0.648627	0.816901	0.591899		0.976614			4.26645
REB1	YPR025C	3.27766	0.648627	0.637897						4.56418
REB1	YPR026W	3.27766	0.648627	1.32697						5.25326
REB1	YPR029C	1.62527	0.648627	2.02809						4.30199
REB1	YPR052C	2.62291	0.648627				0.800077	0.995328		5.06694

TF	Target	Binding_0-2000	Motif_Score_SC	Other_Yeasts	Two-Hop_BE	Two-Hop_BP	Two-Hop_BY	Two-Hop_BG	Max_Membership	Sum
REB1	YPR055W	2.62291	0.648627	0.797852	0.370683					4.44007
REB1	YPR067W	3.46227	0.648627	2.90917						7.02007
REB1	YPR074C	2.212	0.648627	5.29468	0.372707					8.52801
REB1	YPR080W		0.648627	3.8453	0.717154					5.21108
REB1	YPR098C	3.82213	0.648627		0.657924					5.12868
REB1	YPR101W	3.82213	0.648627	0.210387		0.758126				5.43927
REB1	YPR103W		0.648627	0.892498	0.53086	0.962689	0.97882			4.01349
REB1	YPR119W	4.07073	0.648627				0.800938			5.5203
REB1	YPR163C	3.07197	0.648627	2.02313	1.08051					6.82424
REB1	YPR164W	3.07197	0.648627	1.38495						5.10555
REB1	YPR181C	4.1243	0.648627	0.38443		0.75092				5.90828
REB1	YPR182W	4.1243	0.648627	0.448552		0.840824				6.0623
REB1	YPR183W	4.1243	0.648627							4.77293
RFA2	YBR030W			4.06806						4.06806
RFA2	YBR173C			5.44622						5.44622
RFA2	YBR276C			5.65266						5.65266
RFA2	YBR279W			5.33849						5.33849
RFA2	YDL091C		1.34466	2.91359						4.25825
RFA2	YDR275W			4.30436						4.30436
RFA2	YDR297W		0.235138	4.67789						4.91303
RFA2	YDR331W		1.63918	6.89009						8.52927
RFA2	YDR358W		0.591372	6.86475						7.45612
RFA2	YER134C			5.48244						5.48244
RFA2	YER145C			6.1844						6.1844
RFA2	YER146W			4.24366						4.24366
RFA2	YGR042W			5.39874						5.39874
RFA2	YHL024W		0.202449	6.6989						6.90135
RFA2	YHL039W			8.69157						8.69157
RFA2	YHR063C		0.202449	4.39651						4.59896
RFA2	YIL152W		0.867954	3.61248						4.48043
RFA2	YIL154C		0.299955	4.53386						4.83381
RFA2	YJL132W		1.88368	6.47563						8.35931
RFA2	YJL148W		1.09089	5.61697						6.70786
RFA2	YJL198W			4.31322						4.31322
RFA2	YJR045C		1.09089	3.05086						4.14175
RFA2	YJR046W		1.09089	3.00351						4.0944
RFA2	YJR104C			7.16453						7.16453
RFA2	YJR105W			4.28895						4.28895
RFA2	YJR122W		0.591372	7.7471						8.33847
RFA2	YKL099C		0.235138	3.90639						4.14153
RFA2	YKR046C			4.32076						4.32076
RFA2	YLR357W			4.92187						4.92187
RFA2	YMR009W			4.01354						4.01354
RFA2	YMR135C		1.39854	7.82286						9.2214
RFA2	YMR136W		1.39854	4.83736						6.2359
RFA2	YNL190W			6.01439						6.01439
RFA2	YOR023C			6.17704						6.17704
RFA2	YOR025W			6.18472						6.18472
RFA2	YOR152C			5.02127						5.02127
RFA2	YOR237W			7.93026						7.93026
RFA2	YPL111W		2.10496	12.5476						14.6526
RFA2	YPL112C		2.10496	6.13571						8.24067
RFA2	YPR144C		1.09089	4.32271						5.4136
RFA2	YPR145W		1.09089	6.73502						7.82591
RFX1	TM(CAU)J1	3.16273	2.76648							5.92921
RFX1	YAL035W		1.84259	5.2233						7.06589
RFX1	YBR139W		0.791058	3.39393						4.18499
RFX1	YBR202W	0.369887	1.86806	7.57028						9.80823
RFX1	YDL085C-A		3.38952	15.6516						19.0411
RFX1	YDL085W		3.38952	18.8763						22.2658
RFX1	YDL129W	3.12854	1.03852	0.657426						4.82449
RFX1	YDL229W	3.72994	1.69336	6.01896						11.4423
RFX1	YDL231C	3.72994	1.69336	0.524832						5.94813
RFX1	YDR410C	4.09756	1.77344	7.38215						13.2531
RFX1	YDR412W	4.09756	1.77344	0.761152	0.395687					7.02784
RFX1	YEL016C		1.47553	4.44198						5.91751
RFX1	YGR120C			4.27386						4.27386
RFX1	YGR135W			4.23096						4.23096
RFX1	YGR172C		0.70651	4.79301						5.49952
RFX1	YGR180C	1.95108	3.38952	20.4045	0.69313	0.689078				27.1273
RFX1	YGR181W	1.95108	3.38952	2.78983						8.13043
RFX1	YIL064W	2.31264	2.76648	6.12216						11.2013
RFX1	YIL066C	2.31264	2.76648	12.097	0.426318					17.6024
RFX1	YIL078W		1.97409	3.16508						5.13917
RFX1	YIL079C		1.97409	3.40671	0.501662					5.88246
RFX1	YIR038C		1.05434	3.04855	0.459433					4.56232
RFX1	YJL026W	3.16273	2.76648	10.2143	0.445623	0.361989				16.9511
RFX1	YJL029C	3.16273	2.76648	5.82033						11.7495
RFX1	YJL068C	0.156014	1.28322	5.26166						6.70089
RFX1	YJL134W	3.13354	1.86806	13.5633						18.5649
RFX1	YJL136C	3.13354	1.86806							5.0016
RFX1	YJL137C	3.13354	1.86806							5.0016
RFX1	YKL120W		0.879603	3.80058						4.68018
RFX1	YLL033W		2.84394	5.60759						8.45153
RFX1	YLL034C		2.84394	9.78815						12.6321
RFX1	YLR002C	0.144168	0.0811749	5.81439	0.848933					6.88867
RFX1	YLR286C	4.27152	1.14334	4.8624						10.2773
RFX1	YML008C		1.61316	2.81664						4.4298
RFX1	YML053C		0.481967	4.04643						4.5284
RFX1	YML058W	0.737951	0.910356	4.39537		0.459594				6.50327
RFX1	YML058W-A		0.910356	5.5244						6.43476
RFX1	YMR062C		0.586473	3.90682						4.49329
RFX1	YMR063W			5.64295						5.64295
RFX1	YMR138W		1.10489	4.04681						5.1517
RFX1	YMR177W	2.03696	1.44296	2.47495						5.95487
RFX1	YMR279C	1.79172	1.34894	3.40162						6.54228
RFX1	YNL211C	0.936437		3.91959						4.85603
RFX1	YNL247W		1.90217	2.95103						4.8532
RFX1	YOR125C		1.38738	4.00863						5.39601
RFX1	YOR127W		1.38738	2.84319						4.23057
RFX1	YOR267C	3.67401	2.42657							6.10058
RFX1	YOR268C	3.67401	2.42657	0.505799						6.60638
RFX1	YOR269W	3.67401	2.42657	3.20507						9.30565
RFX1	YOR279C	3.9539	2.84394							6.79784
RFX1	YOR280C	3.9539	2.84394	4.52852						11.3264
RFX1	YOR283W	3.9539	2.84394							6.79784
RFX1	YPL097W		1.97409	4.4625						6.43659
RFX1	YPL099C		1.97409	5.92096						7.89505
RFX1	YPL213W		2.15582	11.2772						13.433
RFX1	YPL233W		0.354473	4.17639						4.53086

TF	Target	Binding_0-2000	Motif_Score_SC	Other_Yeasts	Two-Hop_BE	Two-Hop_BP	Two-Hop_BY	Two-Hop_BG	Max_Membership	Sum
RFX1	YPL234C		0.354473	4.26844						4.62291
RGT1	YHR094C	4.36464								4.36464
RIM101	TK(CUU)C	5.32866								5.32866
RIM101	TR(UCU)E	4.62056								4.62056
RIM101	YCR019W	5.32866								5.32866
RIM101	YDL035C	0.554068		3.71339						4.26746
RIM101	YDL179W		0.752513	3.98281						4.73532
RIM101	YDL192W		0.752513	4.13616						4.88867
RIM101	YDL206W			4.0036						4.0036
RIM101	YDR084C	4.16088								4.16088
RIM101	YEL005C			4.34166						4.34166
RIM101	YGL001C	4.39741								4.39741
RIM101	YGR158C		0.752513	2.86134	0.57446					4.18831
RIM101	YGR160W		0.752513	3.86127						4.61378
RIM101	YGR284C		0.752513	3.30681			0.792892			4.85222
RIM101	YIR005W		0.752513	3.80573						4.55824
RIM101	YIR013C		0.752513	3.96837						4.72088
RIM101	YIR035C		0.752513	3.68785						4.44036
RIM101	YIR038C	0.211766	0.752513	3.18904						4.15332
RIM101	YLR047C	4.19428	0.752513	1.72135						6.66814
RIM101	YLR048W	4.19428	0.752513		0.337441					5.28423
RIM101	YLR348C		0.752513	3.42132						4.17383
RIM101	YLR350W		0.752513	3.59119						4.3437
RIM101	YLR357W			3.47437				0.874937		4.34931
RIM101	YMR258C	5.58037								5.58037
RIM101	YOR001W	4.11389		0.671526	0.412616					5.19803
RIM101	YOR303W		0.752513	4.51898						5.27149
RIM101	YOR309C		0.752513	4.48229						5.2348
RIM101	YPL177C		0.752513	3.276						4.02851
RLM1	SNR72	4.15476								4.15476
RLM1	SNR73	4.15476								4.15476
RLM1	SNR74	4.15476								4.15476
RLM1	SNR75	4.15476								4.15476
RLM1	SNR76	4.15476								4.15476
RLM1	YAL003W	3.47296	0.382489	0.554721						4.41017
RLM1	YAL005C	3.47296	0.382489	0.472916						4.32836
RLM1	YBR087W	0.652529		2.48435		0.982993				4.11987
RLM1	YBR160W	1.04832	0.382489	2.61396						4.04477
RLM1	YDR054C	0.913743	0.382489	2.83038						4.12661
RLM1	YDR055W	0.913743	0.382489	5.01938						6.31561
RLM1	YDR077W	4.34289	0.382489	2.05475	0.438794				0.511755	7.73068
RLM1	YDR085C	2.60842	0.382489	4.40856						7.39947
RLM1	YDR249C	0.0659708	0.382489	3.94912						4.39758
RLM1	YDR308C			4.35859						4.35859
RLM1	YDR350C		0.382489	8.81689						9.19938
RLM1	YDR524C	3.06729	0.382489	2.33471						5.78449
RLM1	YEL040W	6.7766								6.7766
RLM1	YER001W	4.92355	0.382489	0.101764						5.4078
RLM1	YER034W		0.382489	5.12546						5.50795
RLM1	YER137C	0.819167		3.19543						4.0146
RLM1	YGL158W	3.78095	0.382489			0.38487		0.905064		5.45337
RLM1	YGR102C		0.382489	4.47187						4.85436
RLM1	YGR189C	3.87579		0.484547	0.53421					4.89455
RLM1	YGR282C	0.446236	0.382489	2.45956	0.764838					4.05312
RLM1	YHR181W		0.382489	3.85838						4.24087
RLM1	YHR191C	4.25149	0.382489							4.63398
RLM1	YHR193C	4.25149	0.382489							4.63398
RLM1	YHR194W	4.25149	0.382489							4.63398
RLM1	YHR209W		0.382489	4.38067	1.14869					5.91185
RLM1	YIL122W	4.04756	0.382489	5.59306						10.0231
RLM1	YJL095W		0.382489	2.7321				0.903386		4.01797
RLM1	YJL158C	3.29323			0.910672					4.2039
RLM1	YJL159W	1.98615	0.382489	2.56872						4.93736
RLM1	YKL067W	0.159119		2.11533	0.859526	1.47375				4.60773
RLM1	YKL095W	8.9644	0.382489							9.34689
RLM1	YKL096C-B	8.9644	0.382489							9.34689
RLM1	YKL096W	8.9644	0.382489	5.31645					0.499434	15.1628
RLM1	YKR070W			4.67924						4.67924
RLM1	YLL049W			5.5843						5.5843
RLM1	YLR110C	3.68167	0.382489	2.16993						6.23409
RLM1	YLR113W	3.68167	0.382489	2.14713		0.411672		0.903382		7.52634
RLM1	YLR300W	5.66492								5.66492
RLM1	YLR332W	6.51864	0.382489	5.20998						12.1111
RLM1	YLR342W	4.57348								4.57348
RLM1	YLR389C	4.42171	0.382489	1.59838						6.40258
RLM1	YLR390W-A	4.42171	0.382489	2.21682						7.02102
RLM1	YML067C			4.69652						4.69652
RLM1	YMR013C	4.15476								4.15476
RLM1	YMR013W-A	4.15476								4.15476
RLM1	YMR014W	5.19155			0.444388					5.63594
RLM1	YNL288W	4.0513								4.0513
RLM1	YOR208W		0.382489	3.66951		0.829166				4.88116
RLM1	YOR313C	5.76327			0.789069					6.55234
RLM1	YOR315W	5.76327			0.789069					6.55234
RLR1	YDL085C-A			5.20872						5.20872
RLR1	YDL085W			5.8124						5.8124
RLR1	YDL116W		0.914984	3.13406						4.04904
RLR1	YDR261C		1.6193	3.45375						5.07305
RLR1	YDR382W			8.20129	0.451738					8.65303
RLR1	YDR391C		1.6193	3.27838						4.89768
RLR1	YEL006W			4.2367						4.2367
RLR1	YER044C-A	0.882677	1.38418	2.53653						4.80339
RLR1	YFL012W		1.32855	4.19198						5.52053
RLR1	YFR032C		1.32855	5.27353						6.60208
RLR1	YFR032C-A		1.32855	5.16607						6.49462
RLR1	YGL091C		1.32855	5.73265						7.0612
RLR1	YGL174W		1.32855	3.18398						4.51253
RLR1	YGR071C	2.46959	1.32855	0.439333						4.23747
RLR1	YGR074W	2.46959	1.32855	1.44452						5.24266
RLR1	YGR268C		1.38418	3.46858						4.85276
RLR1	YGR283C			5.04283						5.04283
RLR1	YHL034C	1.38431		7.37971						8.76402
RLR1	YHR032W			4.53714						4.53714
RLR1	YHR063C		1.6193	3.2448						4.8641
RLR1	YHR070W	0.382182	1.32049	5.76818						7.47085
RLR1	YHR196W			3.83411	0.478646					4.31276
RLR1	YJL139C	1.95593	1.6193	1.67022						5.24545
RLR1	YJL160C			4.15576						4.15576
RLR1	YJR063W			5.21214						5.21214
RLR1	YJR068W		0.725075	5.70922						6.4343

TF	Target	Binding_0-2000	Motif_Score_SC	Other_Yeasts	Two-Hop_BE	Two-Hop_BP	Two-Hop_BY	Two-Hop_BG	Max_Membership	Sum
RLR1	YKL078W			6.58247						6.58247
RLR1	YKL098W		0.725075	3.53557						4.26065
RLR1	YKL100C			6.5059						6.5059
RLR1	YKR060W	0.440707		4.23507						4.67578
RLR1	YKR093W		0.168911	4.26724						4.43615
RLR1	YLR216C		1.6193	4.09896		0.374713				6.09297
RLR1	YLR219W		1.6193	3.82082						5.44012
RLR1	YLR286C		0.687883	4.13028						4.81816
RLR1	YMR115W			4.7471						4.7471
RLR1	YNL026W			4.2205						4.2205
RLR1	YNL236W			4.13177						4.13177
RLR1	YNL250W			5.64926						5.64926
RLR1	YOL104C			4.25147						4.25147
RLR1	YOR226C		0.914984	5.51863						6.43361
RLR1	YOR227W		1.11718	6.19716						7.31434
RLR1	YOR363C		0.0898762	4.74742						4.8373
RLR1	YPR141C	0.419095	0.725075	3.19511						4.33928
RLR1	YPR182W		1.6193	2.65407						4.27337
RLR1	YPR186C			4.23886						4.23886
RLR1	YPR189W			7.47919						7.47919
RME1	SNR128	6.07885	0.0758137							6.15466
RME1	SNR190	6.07885	0.0758137							6.15466
RME1	TT(AGU)J	4.28051	0.0758137							4.35632
RME1	YAL053W			6.42544						6.42544
RME1	YAR002C-A			3.41502		0.716391				4.13141
RME1	YBL028C	0.8406	0.0758137	1.22239	1.3414				0.697617	4.17782
RME1	YBR011C	0.178222	0.0758137	3.55769	0.583502					4.39523
RME1	YBR181C			2.46397	0.947249				0.709091	4.12031
RME1	YBR191W	0.680276		3.75803	1.02508				0.564858	6.02824
RME1	YBR217W	2.62523		2.10684						4.73207
RME1	YBR289W			4.38774						4.38774
RME1	YCR028C		2.02661	2.56931						4.59592
RME1	YCR036W			4.95572						4.95572
RME1	YDL070W		2.02661	1.48246	1.03482					4.54389
RME1	YDR433W		0.0758137	4.33294						4.40875
RME1	YDR505C		2.02661	6.24025						8.26686
RME1	YEL056W			5.54883						5.54883
RME1	YER006W		0.0758137	2.01837	1.43943				0.690696	4.22431
RME1	YER078C	6.97861								6.97861
RME1	YER079W	6.97861								6.97861
RME1	YER080W	6.97861								6.97861
RME1	YER082C			2.68733	1.35439				0.696916	4.73864
RME1	YFR034C	0.248101	2.02661	2.87197						5.14668
RME1	YGL134W	3.55835	0.0758137	1.26833						4.90249
RME1	YGL135W	3.55835			0.765265					4.32362
RME1	YGL136C	3.55835	0.0758137	1.9232						5.55736
RME1	YGL153W			5.07594						5.07594
RME1	YGL186C		2.02661	3.2542						5.28081
RME1	YHR106W			2.57998	1.48832					4.0683
RME1	YIL158W	8.62288	0.0758137	0.773522						9.47222
RME1	YJL148W	6.07885	0.0758137	0.572049					0.709252	7.43596
RME1	YJL190C	1.34865		1.27014	1.00892				0.456693	4.0844
RME1	YJL198W	4.28051	0.0758137		0.573008				0.495186	5.42452
RME1	YJL200C	4.28051	0.0758137							4.35632
RME1	YJR002W			4.70262	1.56582				0.71761	6.98605
RME1	YJR093C		0.0758137	9.02965						9.10546
RME1	YJR094W-A		2.02661	1.52314	0.894247					4.444
RME1	YLR061W	2.23523			1.06989				0.723827	4.02895
RME1	YLR063W	2.23523		4.68926						6.92449
RME1	YLR065C	7.78172								7.78172
RME1	YLR066W	7.78172								7.78172
RME1	YLR367W	2.80308			0.919549				0.593752	4.31638
RME1	YLR372W			5.1842	0.342701					5.5269
RME1	YLR438C-A	1.87573	0.0758137	4.79893						6.75047
RME1	YML014W		2.02661	8.93069						10.9573
RME1	YML125C	2.08275	2.02661							4.10936
RME1	YMR242C		2.02661	0.646572	1.05751				0.728265	4.45896
RME1	YNL032W	2.18926	0.0758137	2.1789						4.44397
RME1	YNL214W		2.02661	2.53653						4.56314
RME1	YNL286W			6.01678						6.01678
RME1	YOL052C-A			4.05186						4.05186
RME1	YOL154W	8.71739								8.71739
RME1	YOR210W		2.02661	3.06628	1.03505					6.12794
RME1	YOR212W			6.00263						6.00263
RME1	YOR215C			3.70204	1.10416					4.8062
RME1	YOR234C	4.06564		0.150974	0.867694					5.08431
RME1	YOR236W	4.06564		0.054367						4.12001
RME1	YOR237W	4.06564								4.06564
RME1	YOR337W		0.0758137	6.0559						6.13171
RME1	YOR339C		2.02661	1.99028						4.01689
RME1	YPL090C	0.905221		2.84726	0.58932				0.677234	5.01904
RME1	YPL143W	3.55519			0.971965				0.715348	5.2425
RME1	YPL219W		0.0758137	5.17019						5.246
RME1	YPL255W		2.02661	4.25745						6.28406
RME1	YPL256C		2.02661	4.99703						7.02364
RME1	YPR136C		2.02661	5.05845					0.594401	7.67946
ROX1	TD(GUC)I1	4.45254	0.473848							4.92639
ROX1	TE(CUC)D	4.07051								4.07051
ROX1	TR(UCU)M1	3.49115	0.806403							4.29755
ROX1	TT(AGU)I2	4.45254	0.473848							4.92639
ROX1	YAL044C			5.93508						5.93508
ROX1	YAL044W-A			4.80662			0.816182			5.6228
ROX1	YAL063C	4.2922								4.2922
ROX1	YBL029C-A	5.83893								5.83893
ROX1	YBL029W	5.83893								5.83893
ROX1	YBR011C		0.473848	2.93573	0.82014					4.22972
ROX1	YBR061C			3.88135	0.636552					4.5179
ROX1	YBR186W		0.806403	4.75427						5.56067
ROX1	YBR191W	0.112961	0.806403	4.55451	1.30083					6.7747
ROX1	YBR202W	0.976063	0.806403	1.94177			0.814195			4.53843
ROX1	YBR214W			4.50922						4.50922
ROX1	YCR105W	4.6032								4.6032
ROX1	YDL051W	0.178011	0.806403	2.9834	1.36412					5.33193
ROX1	YDL194W			4.20063						4.20063
ROX1	YDL197C			5.3952						5.3952
ROX1	YDR159W		0.806403	3.31044						4.11684
ROX1	YDR231C		0.806403	9.86114	0.481527					11.1491
ROX1	YDR232W		0.806403	4.30652						5.11292
ROX1	YDR259C	4.5992								4.5992
ROX1	YDR279W	4.07051								4.07051

TF	Target	Binding_0-2000	Motif_Score_SC	Other_Yeasts	Two-Hop_BE	Two-Hop_BP	Two-Hop_BY	Two-Hop_BG	Max_Membership	Sum
ROX1	YDR306C	1.48039		4.04967						5.53006
ROX1	YDR441C	4.60908								4.60908
ROX1	YEL058W	1.44182		2.61171						4.05353
ROX1	YEL060C	1.44182		2.68681						4.12863
ROX1	YER046W	3.47649		0.767386						4.24388
ROX1	YER107C		0.120558	4.41997	0.615205		1.12051			6.27624
ROX1	YFR053C			5.02157	0.443721					5.46529
ROX1	YGL048C	1.80131	0.806403	0.783524	0.427147	0.471439				4.28982
ROX1	YGL134W	4.83389								4.83389
ROX1	YGL135W	4.83389			0.750204					5.58409
ROX1	YGL136C	4.83389								4.83389
ROX1	YGL162W		0.473848	5.1387						5.61255
ROX1	YGR123C	1.9487	0.120558		1.12717		0.953361			4.14979
ROX1	YGR177C			5.62165						5.62165
ROX1	YHR007C	0.612254	0.806403	3.65658						5.07524
ROX1	YHR008C		0.806403	5.69675						6.50315
ROX1	YHR088W	2.45218			0.939611		0.949568			4.34136
ROX1	YHR123W	0.154181	0.806403	3.74855						4.70913
ROX1	YIL014C-A	4.45254	0.120558							4.5731
ROX1	YIL014W	4.45254	0.473848							4.92639
ROX1	YIL070C	0.246678	0.806403	7.36653	0.461392					8.881
ROX1	YIL099W	6.97912			0.836947					7.81607
ROX1	YIL101C	6.97912			0.836947					7.81607
ROX1	YIL118W	4.54421	0.473848	0.611086			1.06469			6.69383
ROX1	YIL119C	4.54421	0.473848	0.573725						5.59178
ROX1	YIR019C	8.59942	0.473848							9.07327
ROX1	YJL148W	3.22252			1.42234					4.64486
ROX1	YJR096W		0.806403	3.86764	0.521504					5.19555
ROX1	YJR100C	3.09074	0.806403	3.6868						7.58394
ROX1	YJR101W	3.09074	0.806403	7.34524						11.2424
ROX1	YKL018C-A		0.473848	7.07346						7.54731
ROX1	YKR074W	1.52333		2.52027						4.0436
ROX1	YKR098C		0.806403	6.33651						7.14291
ROX1	YKR099W		0.806403	8.50399						9.31039
ROX1	YLL011W	7.76554			0.818323					8.58386
ROX1	YLL027W	2.44575	0.806403	3.69168	0.947261					7.89109
ROX1	YLL034C	0.687678	0.473848	3.90662	0.999163					6.06731
ROX1	YLR303W			5.44863						5.44863
ROX1	YLR304C	0.994249		3.22122						4.21547
ROX1	YLR384C		0.806403	4.63707						5.44347
ROX1	YLR385C		0.806403	4.51408						5.32048
ROX1	YML116W	3.51437		0.632618						4.14699
ROX1	YMR014W	2.50447			1.54042					4.04489
ROX1	YMR135C	2.49097		2.18472						4.67569
ROX1	YMR173W	3.52023	0.806403							4.32663
ROX1	YMR203W		0.473848	4.28276						4.75661
ROX1	YMR208W			4.86957						4.86957
ROX1	YMR239C	3.49115			1.69147					5.18262
ROX1	YMR319C	5.42305		2.41303						7.83608
ROX1	YNL075W			4.41977	1.25482					5.67459
ROX1	YNL087W	2.3268		0.515075	1.2478		0.949568			5.03924
ROX1	YNL132W	2.25162			0.741666	1.15012				4.14341
ROX1	YNL165W			5.31596						5.31596
ROX1	YNL175C	1.82673			1.31725		1.02704			4.17102
ROX1	YNL178W	1.51447		4.41657	1.27254		0.779966			7.98355
ROX1	YNR027W			7.23591						7.23591
ROX1	YOL080C	1.94948			1.53084		1.02169			4.50201
ROX1	YOL156W	3.88485	0.473848							4.3587
ROX1	YOL157C	3.88485	0.473848	3.21928						7.57798
ROX1	YOR178C	4.55463		1.5787						6.13333
ROX1	YOR234C	3.33603			0.724609					4.06064
ROX1	YOR319W	4.03609								4.03609
ROX1	YPL013C		0.806403	3.58376						4.39016
ROX1	YPL034W			5.23978						5.23978
ROX1	YPL043W		0.120558	4.51009	1.41655		0.971208			7.01841
ROX1	YPL118W			4.16662						4.16662
ROX1	YPR004C			4.1986						4.1986
ROX1	YPR063C	3.71079	0.473848							4.18464
ROX1	YPR065W	3.71079	0.473848	0.574497						4.75913
ROX1	YPR066W	3.71079	0.473848							4.18464
RPH1	YAL060W	1.2724	1.48547	3.73352						6.49139
RPH1	YBL061C			4.75064						4.75064
RPH1	YBR072W			6.36127						6.36127
RPH1	YBR135W	0.680938		3.93711						4.61805
RPH1	YBR234C	0.689318	1.38418	3.10288						5.17638
RPH1	YCL031C		1.48547	8.42908						9.91455
RPH1	YCL048W		1.48547	7.85646						9.34193
RPH1	YCL049C		1.48547	8.2716						9.75707
RPH1	YCR066W			4.0836						4.0836
RPH1	YCR105W	4.54711								4.54711
RPH1	YCR107W	7.1334			0.552376					7.68578
RPH1	YDR153C	2.74588	1.48547							4.23135
RPH1	YDR154C		1.48547	4.7191						6.20457
RPH1	YDR155C	2.74588	1.48547	14.3917						18.623
RPH1	YDR156W	2.74588	1.48547	11.2218						15.4532
RPH1	YDR158W	2.74588	1.48547	2.57789						6.80924
RPH1	YDR185C			5.28769						5.28769
RPH1	YDR402C	0.196848	1.18429	3.94422						5.32536
RPH1	YDR516C		1.48547	10.6775						12.163
RPH1	YDR517W		1.48547	11.0178						12.5033
RPH1	YEL013W		1.18429	4.19376						5.37805
RPH1	YER053C	0.758645		12.591	0.366793				0.598537	14.315
RPH1	YER067W		1.07686	6.93828					0.61092	8.62606
RPH1	YER073W		1.18429	3.39806						4.58235
RPH1	YFR022W	0.310753	1.38418	2.39073						4.08566
RPH1	YGL002W			5.80325						5.80325
RPH1	YGL010W	0.637519		4.27048						4.908
RPH1	YHL021C	0.23379	1.48547	6.68066	0.373732				0.672959	9.44661
RPH1	YHL032C		1.38418	3.38144						4.76562
RPH1	YHR139C		1.18429	2.82891						4.0132
RPH1	YHR140W		1.18429	2.88425						4.06854
RPH1	YHR156C	1.40234		2.62621						4.02855
RPH1	YHR195W	1.62171		4.16664						5.78835
RPH1	YJL068C	3.79186		0.469715						4.26157
RPH1	YJL069C	3.79186		0.274916	0.521184					4.58796
RPH1	YJL177W			3.68142	0.603536					4.28496
RPH1	YJL210W	1.1583		4.51009						5.66839
RPH1	YJL212C	1.1583		4.21418						5.37248
RPH1	YJR143C	4.4039								4.4039
RPH1	YKL034W			5.70281						5.70281

TF	Target	Binding_0-2000	Motif_Score_SC	Other_Yeasts	Two-Hop_BE	Two-Hop_BP	Two-Hop_BY	Two-Hop_BG	Max_Membership	Sum
RPH1	YKR002W			5.59673						5.59673
RPH1	YKR062W	2.83947		1.43912						4.27859
RPH1	YLL026W	0.123985	1.38418	4.84319	0.401591				0.571465	7.32441
RPH1	YLR108C	5.85331		2.13978						7.99309
RPH1	YLR109W	1.07216	1.07686	2.67158					0.669506	5.49011
RPH1	YLR268W			6.45992						6.45992
RPH1	YLR336C	5.9227								5.9227
RPH1	YLR438C-A	4.19968	1.48547			0.428613				6.11376
RPH1	YLR439W	4.19968								4.19968
RPH1	YML070W	0.139126		4.49126					0.69402	5.32441
RPH1	YMR041C	0.247557		2.24737			1.0992	0.941036	0.55851	5.09367
RPH1	YMR063W			4.05186						4.05186
RPH1	YNL042W		2.84065	11.3173						14.1579
RPH1	YNL234W		1.48547	4.84576						6.33123
RPH1	YNL241C	4.92403							0.704007	5.62804
RPH1	YNR001C		1.18429	2.91028						4.09457
RPH1	YNR038W	0.0871106		4.30403	0.441101					4.83224
RPH1	YOL052C-A		1.38418	3.61638	0.357273					5.35783
RPH1	YOL153C			3.43055					0.728579	4.15913
RPH1	YOR114W		1.48547	4.04608						5.53155
RPH1	YOR120W			3.67928			1.09784		0.679223	5.45634
RPH1	YOR161C		1.48547	9.147					0.674218	11.3067
RPH1	YOR226C	0.421706	1.18429	2.40585						4.01185
RPH1	YOR227W	0.421706	1.18429	2.59046						4.19646
RPH1	YOR386W		2.84065	1.53988						4.38053
RPH1	YPL113C	0.751374		7.47557						8.22694
RPH1	YPL230W		1.48547	4.61429					0.628376	6.72814
RPH1	YPL272C			4.4582						4.4582
RPN4	YAL053W		1.93317	6.19705						8.13022
RPN4	YAL054C		1.93317	4.64983						6.583
RPN4	YBL020W	3.98339	1.93317	2.86559						8.78215
RPN4	YBL022C	3.98339	1.93317	0.484654					0.482374	6.88359
RPN4	YBL041W	1.37937	1.93317	6.91883	1.26016	0.5048	1.16109			13.1574
RPN4	YBL042C	1.37937	1.93317	2.88576		0.350963				6.54926
RPN4	YBL058W	0.362621	1.28918	4.60492						6.25672
RPN4	YBL077W		1.93317	2.3564						4.28957
RPN4	YBR008C	1.15411	0.698105	4.6312					0.527861	7.01128
RPN4	YBR049C	5.02927	1.93317	4.16834	0.329707					11.4605
RPN4	YBR051W		1.93317	2.15548						4.08865
RPN4	YBR170C	1.68821	0.852775	0.852794	1.0362	1.83199				6.26197
RPN4	YBR233W	8.51091	1.93317							10.4441
RPN4	YBR283C	3.57758	1.93317	3.42922			1.1532			10.0932
RPN4	YBR284W	3.57758	1.93317	4.3682	1.43065					11.3096
RPN4	YCL004W			5.02279						5.02279
RPN4	YCL040W		1.93317	2.47159	0.366608					4.77137
RPN4	YCL043C		1.93317	6.05829	0.760869		1.11519			9.86752
RPN4	YCL057C-A		1.93317	3.08029						5.01346
RPN4	YCL057W	0.901117	1.93317	2.72671						5.561
RPN4	YCL059C	0.901117	1.93317		1.09071		0.805345			4.73034
RPN4	YCR059C	3.90138	1.93317							5.83455
RPN4	YCR061W	3.90138	1.93317	7.08779						12.9223
RPN4	YCR082W	4.60243	1.93317	3.80502						10.3406
RPN4	YCR083W	4.60243	1.93317	4.70121						11.2368
RPN4	YCR092C	4.36629	1.06505	0.74363	1.04703					7.222
RPN4	YCR093W	4.36629	1.06505	0.757135		0.677373				6.86585
RPN4	YDL007W	3.88496	1.93317	2.45918	1.72367	1.4853				11.4863
RPN4	YDL037C	2.19588	1.93317							4.12905
RPN4	YDL047W		1.93317			1.05651		1.37229		4.36197
RPN4	YDL070W	1.57014	1.93317	4.62414	0.450482			1.14123		9.71916
RPN4	YDL072C	1.57014	1.93317	5.03183	0.499395					9.03453
RPN4	YDL095W	4.58482	1.93317	2.32937						8.84736
RPN4	YDL097C	4.58482	1.93317		0.863788	1.29477				8.67655
RPN4	YDL098C	4.58482	1.93317							6.51799
RPN4	YDL126C		1.93317	3.48571	1.06176	1.83199				8.31263
RPN4	YDL143W	3.82517	1.28918	2.03						7.14435
RPN4	YDL144C	3.82517	1.28918	2.0298						7.14415
RPN4	YDL145C	3.82517	1.28918			0.823451				5.9378
RPN4	YDL147W		1.93317	3.55979	1.68703	1.71726				8.89725
RPN4	YDL148C		1.93317	2.97344	1.06865					5.97526
RPN4	YDL182W	3.2121	0.852775		0.713514					4.77839
RPN4	YDL183C	3.2121	0.852775							4.06487
RPN4	YDL188C		1.93317			1.71726	0.942813	1.36348		5.95672
RPN4	YDR117C	0.913774	1.28918	4.39809						6.60104
RPN4	YDR127W	2.92179				1.14081				4.0626
RPN4	YDR152W	2.28335	1.93317							4.21652
RPN4	YDR155C	1.59118	0.852775	1.05608	0.81505					4.31508
RPN4	YDR156W	1.59118	1.93317	1.04877						4.57312
RPN4	YDR178W	0.593834	1.93317	2.21784						4.74484
RPN4	YDR270W	4.3328	1.06505							5.39785
RPN4	YDR334W	3.23838	0.852775							4.09115
RPN4	YDR363W-A	2.57907	1.28918	2.55155						6.4198
RPN4	YDR381W		1.06505			1.94232	1.09781			4.10518
RPN4	YDR394W	3.31038	1.93317	2.84074	1.10838	1.29477				10.4874
RPN4	YDR426C		1.93317	2.69048						4.62365
RPN4	YDR427W	5.31684	1.93317	7.12774	1.564	1.29477				17.2365
RPN4	YDR504C		1.93317	2.27512						4.20829
RPN4	YEL032W		1.93317	2.91793		0.356909	1.13861			6.34662
RPN4	YEL037C	2.18752	1.28918	2.41711		1.4853				7.37911
RPN4	YEL070W	4.57819	1.93317							6.51136
RPN4	YER004W	0.546102	1.28918	2.33256						4.16784
RPN4	YER012W	2.4801	1.93317	6.38456	1.56561	1.9078	1.1359			15.4071
RPN4	YER013W	2.4801	1.93317							4.41327
RPN4	YER021W	3.55607	1.93317	5.95353	1.38241	1.29477				14.1199
RPN4	YER093C-A	5.54188	1.93317							7.47505
RPN4	YER094C	5.54188	1.93317	3.87427	1.52707	1.71726	1.1359			15.7295
RPN4	YER095W	5.54188	1.93317	3.58908		0.538405	1.12533			12.7279
RPN4	YER142C	4.58692	0.30704	0.148095						5.04205
RPN4	YER143W	4.58692	0.30704	0.0908016		0.599469			0.588744	6.17297
RPN4	YER163C	2.04261	0.852775	1.86802						4.7634
RPN4	YER166W		1.93317	2.3174						4.25057
RPN4	YER177W	3.14583	1.93317	3.27928	0.585594					8.94387
RPN4	YER178W	3.14583	1.93317		1.04843					6.12743
RPN4	YFL023W	7.85404	1.93317	4.84098						14.6282
RPN4	YFL024C	7.85404	1.93317	4.39095						14.1782
RPN4	YFL045C	1.62014	1.93317	1.1666						4.71991
RPN4	YFL055W	3.59399	1.93317		1.43094		0.82627			7.78437
RPN4	YFL056C	3.59399	1.93317		0.810294			1.20672	0.73237	8.27654
RPN4	YFL057C	3.59399	1.93317		1.43058			1.20672	0.716806	8.88127
RPN4	YFR003C	3.84526	1.93317	5.21677	0.896446					11.8916
RPN4	YFR004W	3.84526	1.93317	5.19178	1.69598	1.29477				13.961

TF	Target	Binding_0-2000	Motif_Score_SC	Other_Yeasts	Two-Hop_BE	Two-Hop_BP	Two-Hop_BY	Two-Hop_BG	Max_Membership	Sum
RPN4	YFR010W	1.99068	0.852775	1.00198	1.12873	1.4853				6.45946
RPN4	YFR024C-A	1.35431	1.93317		1.23062					4.5181
RPN4	YFR050C	3.83582	1.93317	7.90533	0.711915	1.71726	0.969106			17.0726
RPN4	YFR051C	1.35833	1.93317	4.91551		0.677373				8.88438
RPN4	YFR052W	1.35833	1.93317	6.53131	1.68304	1.29477				12.8006
RPN4	YGL010W	7.67727	1.93317	8.67584						18.2863
RPN4	YGL011C	7.67727	1.93317	9.07868	1.09087	1.14081	0.885183			21.806
RPN4	YGL047W	2.14083	1.93317	7.28234						11.3563
RPN4	YGL048C	2.14083	1.93317	6.12078	1.74035	1.29477				13.2299
RPN4	YGL049C	2.14083	1.93317							4.074
RPN4	YGL062W		1.28918	3.8571						5.14628
RPN4	YGL122C	3.47401	1.93317	4.29175						9.69893
RPN4	YGL130W	1.19662	1.93317		0.559905	0.459213				4.14891
RPN4	YGL172W	3.3486	1.28918	1.72437		1.10923				7.47138
RPN4	YGL173C	3.3486	1.28918	1.28231						5.92009
RPN4	YGL223C		0.0637322	4.21646						4.28019
RPN4	YGL238W		1.93317	4.88181						6.81498
RPN4	YGL240W		1.93317	3.3979	0.475597					5.80667
RPN4	YGR016W			4.37447						4.37447
RPN4	YGR047C	7.39642	1.28918	0.778485			0.963722			10.4278
RPN4	YGR048W	7.39642	1.28918	1.38046	0.859178					10.9252
RPN4	YGR049W	7.39642	1.28918		0.832935					9.51853
RPN4	YGR060W	2.01291	1.93317	3.62463		0.387154				7.95786
RPN4	YGR135W	2.25833	1.93317	2.82538	1.60959	1.71726	0.892165			11.2359
RPN4	YGR136W	2.25833	1.93317	1.04852						5.24002
RPN4	YGR209C	2.82679	1.06505	0.31473	0.98749				0.721574	5.91563
RPN4	YGR211W	2.82679	1.06505				0.851605			4.74345
RPN4	YGR253C	3.36903	1.93317	8.19999		0.5048	0.885183			14.8922
RPN4	YGR254W	3.36903	1.93317	0.335911						5.63811
RPN4	YGR262C	1.58444	0.698105			0.703794	1.10407	1.67592		5.76633
RPN4	YHL024W		1.93317	2.59822	0.611361					5.14275
RPN4	YHL030W	0.715108	1.93317	4.97204		1.29477				8.91509
RPN4	YHL031C	0.715108	1.93317	7.28361						9.93189
RPN4	YHR021C	1.11711	1.93317		0.35795		1.05761			4.46584
RPN4	YHR027C		1.93317	2.58315		1.4853				6.00162
RPN4	YHR190W		0.349364	5.31744	0.722021					6.38883
RPN4	YHR199C	1.35093	1.28918	1.62292						4.26303
RPN4	YHR200W	1.35093	1.28918	1.6218		1.71726				5.97917
RPN4	YIL075C	5.977	1.93317	3.88735		0.678962				12.4765
RPN4	YIR012W			4.39979						4.39979
RPN4	YJL001W	2.66927	1.93317	7.6957	1.30752	1.71726	1.13646			16.4594
RPN4	YJL002C	2.66927	1.93317	10.8742						15.4766
RPN4	YJL008C	1.16558	0.852775	0.487199		0.433502	1.09233			4.03139
RPN4	YJL045W		1.93317	2.15271						4.08588
RPN4	YJL102W	0.704366	1.93317	5.17141						7.80895
RPN4	YJL103C	0.704366	1.93317	4.57034						7.20788
RPN4	YJL116C		1.93317	5.89076						7.82393
RPN4	YJR006W	3.26481	0.852775	0.241337						4.35892
RPN4	YJR100C			5.64954						5.64954
RPN4	YKL009W	2.2286			0.904602		1.05761			4.19081
RPN4	YKL015W	5.19218	1.28918	0.61278						7.09414
RPN4	YKL016C	5.19218	1.28918	1.30927						7.79063
RPN4	YKL017C	5.19218	1.28918				0.798271			7.27963
RPN4	YKL054C	4.51074	1.93317	2.93963						9.38354
RPN4	YKL145W	6.11221	1.93317	2.06549	1.34983	1.08006				12.5408
RPN4	YKL195W	5.22962		0.108465	1.09681					6.43489
RPN4	YKL196C	5.22962								5.22962
RPN4	YKL197C	5.22962		0.92638						6.156
RPN4	YKL210W	3.57578	1.93317	3.12298						8.63193
RPN4	YKL211C	3.57578	1.93317	3.72645						9.2354
RPN4	YKL212W	0.25061	1.93317	3.51641						5.70019
RPN4	YKR093W			3.19543			0.861838			4.05727
RPN4	YLL039C	2.83079	1.93317	2.59971	0.391307	1.07354				8.82852
RPN4	YLR095C		1.93317	4.21618						6.14935
RPN4	YLR096W		1.93317	4.47191	0.604938	1.05047				8.06049
RPN4	YLR149C		1.93317	2.76194	0.505952					5.20106
RPN4	YLR200W	1.39167	1.06505		0.471131	1.57827	1.14084			5.64696
RPN4	YLR287C	3.08488	0.852775		0.380668					4.31832
RPN4	YLR421C	2.73889	1.28918	2.39096	1.49122	1.4853				9.39555
RPN4	YLR422W	2.73889	1.28918	0.589713						4.61778
RPN4	YML007W	2.2233	1.93317	2.7538	0.589867				0.56147	8.06161
RPN4	YML008C	2.2233	1.93317	1.25234	0.895971					6.30478
RPN4	YML048W	2.05939	1.93317	4.63021						8.62277
RPN4	YML049C			4.29248						4.29248
RPN4	YML050W		1.93317	2.19401						4.12718
RPN4	YML057W		1.93317	4.63659				1.37229		7.94205
RPN4	YML092C	4.7191	1.93317	6.25238	0.720218	1.71726	0.885183			16.2273
RPN4	YML093W	0.559632	1.06505		1.03892		1.03143	1.12226		4.81729
RPN4	YML111W			5.27633						5.27633
RPN4	YMR012W	2.44399				1.14081	0.798712			4.38351
RPN4	YMR068W	0.932242	1.28918	2.61983	0.78649					5.62774
RPN4	YMR186W	2.98267			0.941859	1.14081				5.06534
RPN4	YMR202W		1.93317	4.80207	0.602655					7.33789
RPN4	YMR275C	3.12526	1.93317	5.92319						10.9816
RPN4	YMR276W	3.12526				1.4488				4.57406
RPN4	YMR277W	3.12526	1.93317							5.05843
RPN4	YMR310C		1.93317	2.29892	0.454456					4.68655
RPN4	YMR314W	0.328718	0.852775	0.837527	1.40023	1.71726	0.9002			6.03671
RPN4	YNL055C			3.21384	0.846695					4.06053
RPN4	YNL111C	2.60392			1.56854					4.17246
RPN4	YNL155W	6.71312	1.93317	4.97292						13.6192
RPN4	YNL156C	6.71312	1.93317	4.04393	0.972515					13.6627
RPN4	YNL241C	0.507458	1.28918	2.20769					0.445131	4.44946
RPN4	YNL250W	3.02518	1.93317	3.24075						8.1991
RPN4	YNL251C	3.02518	1.93317	3.36705						8.3254
RPN4	YNL262W		1.93317	3.51693			0.874323			6.32442
RPN4	YNL264C		1.93317	8.28436						10.2175
RPN4	YNR032C-A		1.93317	2.31031						4.24348
RPN4	YOL038W	2.1796	1.93317	7.61394	1.40825	1.71726	0.885183			15.7374
RPN4	YOL040C	2.1796	1.93317		0.419528		1.16109			5.69339
RPN4	YOL046C		1.93317	2.1669						4.10007
RPN4	YOL165C	1.66788			1.11401			1.20672	0.729896	4.71851
RPN4	YOR006C	6.19777	1.93317							8.13094
RPN4	YOR007C	6.19777	1.93317	6.49566	1.38236		1.10445			17.1134
RPN4	YOR051C	4.36512	1.93317							6.29829
RPN4	YOR052C	4.36512	1.93317	7.26754	0.833689					14.3995
RPN4	YOR116C	1.022	1.93317	3.90502						6.86019
RPN4	YOR117W	1.022	1.93317	6.11512	1.29689	1.29477				11.6619
RPN4	YOR124C			4.00694						4.00694
RPN4	YOR156C	5.25277	0.852775							6.10555

TF	Target	Binding_0-2000	Motif_Score_SC	Other_Yeasts	Two-Hop_BE	Two-Hop_BP	Two-Hop_BY	Two-Hop_BG	Max_Membership	Sum
RPN4	YOR157C	5.25277	0.852775	0.659903	1.27932	1.10923	1.14396			10.298
RPN4	YOR158W	5.25277	0.852775	0.625041						6.73059
RPN4	YOR259C	3.83023	1.93317	4.42757	1.59716	1.29477				13.0829
RPN4	YOR260W	3.83023	1.93317	4.90977						10.6732
RPN4	YOR261C	5.13369	0.852775	0.399984	1.58241	1.29477				9.26363
RPN4	YOR262W	5.13369	0.852775	0.819826			0.799017			7.60531
RPN4	YOR264W	5.13369	0.852775							5.98646
RPN4	YOR362C	0.996853	1.93317	9.06972		1.9078	0.885183			14.7927
RPN4	YOR374W			3.42551	0.98614					4.41165
RPN4	YPL060W		1.28918	2.84039						4.12957
RPN4	YPR023C	0.768736	1.28918	2.75075		1.76498				6.57365
RPN4	YPR024W	0.768736	1.28918	3.04875						5.10667
RPN4	YPR095C	0.150948	1.93317	1.15748				1.11489		4.35649
RPN4	YPR102C	0.760634	1.93317	5.21746	0.628276		1.07658			9.61612
RPN4	YPR103W	0.760634	1.93317	7.08451	1.18832	1.71726	1.14434			13.8282
RPN4	YPR107C	3.6474	1.93317	5.52599	0.817552					11.9241
RPN4	YPR108W	3.6474	1.93317	6.13889	1.65576	1.29477				14.67
RPN4	YPR109W	1.6187	1.93317	2.61087						6.16274
RPN4	YPR148C			4.79562						4.79562
RPN4	YPR185W		1.93317	1.84561		0.860953				4.63973
RTG1	TW(CCA)J	5.42982								5.42982
RTG1	TW(CCA)K	4.77569								4.77569
RTG1	YBR109C	0.203959		9.54135						9.74531
RTG1	YBR110W	2.35933		4.81019						7.16952
RTG1	YCR096C	3.28634				0.722448				4.00879
RTG1	YCR097W	3.28634				0.722448				4.00879
RTG1	YDR042C	4.40327		0.824389						5.22766
RTG1	YDR084C	4.81865								4.81865
RTG1	YDR287W			4.19047						4.19047
RTG1	YGL253W	4.01833		0.845917						4.86425
RTG1	YGL254W	4.01833								4.01833
RTG1	YHR016C			4.26724						4.26724
RTG1	YLR397C	4.54965								4.54965
RTG1	YLR452C	0.372761		3.67028						4.04304
RTG1	YML048W			4.45248						4.45248
RTG1	YMR068W	0.412424		4.39717						4.80959
RTG1	YPR016C	0.135854		3.81635		0.362237				4.31444
RTG3	TK(CUU)C	5.14354								5.14354
RTG3	TN(GUU)N2	6.75277								6.75277
RTG3	TR(UCU)E	4.63641								4.63641
RTG3	TW(CCA)J	4.40326								4.40326
RTG3	YBL043W	3.90149	0.0972284		0.663117					4.66184
RTG3	YCR019W	5.14354								5.14354
RTG3	YCR105W	5.03139								5.03139
RTG3	YCR107W	4.98209								4.98209
RTG3	YDL066W	4.85983	0.0972284	0.00838309	0.824812					5.79025
RTG3	YDL067C	4.85983	0.0972284	0.0459366	0.467354					5.47035
RTG3	YDL069C	4.85983	0.0972284	0.827498						5.78456
RTG3	YDL182W	3.6016		0.0991053				0.875301		4.57601
RTG3	YDR380W	0.804342		2.30576			0.953045		0.73259	4.79574
RTG3	YEL071W	3.78293	0.0972284	0.9421					0.451597	5.27386
RTG3	YER013W	2.97841		1.22134						4.19975
RTG3	YER069W	3.63214					0.867026		0.503362	5.00253
RTG3	YER137C		0.0972284	4.4496						4.54683
RTG3	YFR017C	2.71766	0.0972284		0.469868				0.718253	4.00301
RTG3	YGR071C	4.77859								4.77859
RTG3	YGR072W	4.77859		1.72183						6.50042
RTG3	YGR074W	4.77859								4.77859
RTG3	YHR018C	2.28949			0.791561		0.986024			4.06707
RTG3	YHR161C	3.07889			1.18595					4.26484
RTG3	YIL056W	2.91108		1.29754						4.20862
RTG3	YIL057C	2.91108		1.26683	0.397194					4.5751
RTG3	YJL088W	5.72024	0.0972284					0.954582	0.606408	7.37846
RTG3	YJR109C	4.42858	0.0972284	0.150299	0.354601	0.490112		1.18733		6.70815
RTG3	YJR110W	4.42858	0.0972284	0.145014						4.67082
RTG3	YKL067W	3.60895	0.0972284	0.00590778	0.818342				0.717067	5.2475
RTG3	YKL085W	1.7897			1.04809	1.65821			0.70988	5.20588
RTG3	YKR072C	0.547074	0.0972284	2.99161					0.670284	4.3062
RTG3	YLR003C		0.0972284	3.71339	0.811686					4.6223
RTG3	YLR004C			4.34166						4.34166
RTG3	YLR304C	6.21528	0.0972284	1.49965	0.912797		0.938902		0.730879	10.3947
RTG3	YMR099C		0.0972284	3.94098						4.03821
RTG3	YMR117C	2.67503		1.57577	0.452506					4.70331
RTG3	YNL036W	5.43826	0.0972284						0.711135	6.24662
RTG3	YNL037C	5.43826	0.0972284		1.07071	1.15481	0.930667		0.726555	9.41823
RTG3	YNR001C	6.75277			1.28249		0.828707		0.731143	9.59511
RTG3	YOL058W	1.89484		3.56346			0.976993		0.543651	6.97894
RTG3	YOL128C		0.0972284	5.14423						5.24146
RTG3	YOR136W	6.28542	0.0972284	0.578516	1.37795	0.92612	0.930667		0.730169	10.9261
RTG3	YOR303W	2.42569				1.0308	0.902214	1.2681		5.6268
RTG3	YPL135W	3.08424		0.202449	0.849987					4.13668
RTG3	YPL224C		0.0972284	6.41819						6.51542
RTG3	YPR040W	5.8522								5.8522
RTG3	YPR198W	3.5483	0.0972284		0.383554		0.898368			4.92745
SCB	YDL170W			5.68458						5.68458
SCB	YFL045C			4.56835						4.56835
SCB	YGR076C			4.37037						4.37037
SCB	YKL113C			4.15076						4.15076
SCB	YLR207W			5.01296						5.01296
SCB	YMR199W			6.14475						6.14475
SCB	YMR286W			5.80926						5.80926
SFP1	YBL087C	2.0791		1.41498	1.13107				0.730586	5.35574
SFP1	YBR048W	3.64238			1.16745				0.732681	5.54251
SFP1	YBR181C	1.45836	0.301244	2.7265	1.11422				0.730727	6.33105
SFP1	YBR188C	2.40389	0.592597	12.0471						15.0436
SFP1	YBR189W	2.40389	0.592597		1.20731				0.732326	4.93612
SFP1	YBR191W	2.09168	0.592597	2.4681	1.22102				0.725883	7.09928
SFP1	YBR276C			4.4225					0.708621	5.13112
SFP1	YBR279W			5.20872		0.433264				5.64198
SFP1	YCR069W		0.73344	6.18374						6.91718
SFP1	YDL061C	0.60056	0.592597	2.91573	0.818625				0.731842	5.65935
SFP1	YDL075W	1.39906		1.69899	0.765808				0.72997	4.59383
SFP1	YDL076C	1.39906		3.4433						4.84236
SFP1	YDL082W	2.24954	0.73344		1.00141				0.731111	4.7155
SFP1	YDL083C	2.24954	0.73344	1.57577	1.00644				0.729148	6.29434
SFP1	YDL130W	2.2989	0.73344	1.77364	0.695994					5.50197
SFP1	YDL133W	2.48511	0.784553	4.28052						7.55018
SFP1	YDR046C			5.36772					0.593958	5.96168
SFP1	YDR047W			5.87448						5.87448
SFP1	YDR064W	2.63987		0.183276	0.973566				0.712068	4.50878

TF	Target	Binding_0-2000	Motif_Score_SC	Other_Yeasts	Two-Hop_BE	Two-Hop_BP	Two-Hop_BY	Two-Hop_BG	Max_Membership	Sum
SFP1	YDR076W			5.11319						5.11319
SFP1	YDR405W	0.26332	0.784553	4.99659						6.04446
SFP1	YDR418W	0.205076		10.4497	1.10239				0.71499	12.4722
SFP1	YDR447C	1.31983	0.73344	2.8046	1.06349				0.723762	6.64512
SFP1	YDR448W	1.31983	0.73344	5.46008						7.51335
SFP1	YDR449C	0.202843	0.592597	2.78559	0.351873				0.732725	4.66563
SFP1	YDR501W	1.65135		2.82397						4.47532
SFP1	YEL054C	0.933608		1.05963	0.991079	0.455885			0.729125	4.16933
SFP1	YEL056W		0.356388	4.54767						4.90406
SFP1	YER002W			4.46486					0.631641	5.0965
SFP1	YER074W	2.35546		1.23606	1.21304				0.731944	5.5365
SFP1	YER101C	0.194034		4.2046						4.39863
SFP1	YER116C	1.64838	0.356388	5.8135						7.81827
SFP1	YER117W	1.64838	0.356388	2.40721	1.04477				0.725555	6.1823
SFP1	YFR031C-A	1.4408	1.00391	2.08074	1.04788				0.730116	6.30345
SFP1	YFR032C-A		0.592597	3.48391	0.494377					4.57088
SFP1	YGL031C	1.11479	0.301244	2.93587	1.07973				0.730011	6.16165
SFP1	YGL103W	2.80252		0.949917	1.05914				0.723774	5.53535
SFP1	YGL115W			5.76337						5.76337
SFP1	YGL123W	0.372797	0.503769	4.1864	0.915885				0.732364	6.71121
SFP1	YGL124C	0.372797	0.503769	3.88234						4.75891
SFP1	YGL135W	2.55316	0.73344	7.54348	1.1412				0.731883	12.7032
SFP1	YGL136C	2.55316	0.73344	8.24858						11.5352
SFP1	YGL189C	0.83793	0.73344	8.12307	0.907263				0.728682	11.3304
SFP1	YGR027C	1.34041		1.94075	1.03085				0.73095	5.04296
SFP1	YGR085C		0.73344	5.00608	1.29098				0.730832	7.76133
SFP1	YGR117C	2.25174		3.6583						5.91004
SFP1	YGR118W	2.25174	0.0522983		1.13594				0.732026	4.172
SFP1	YGR148C	0.449731		2.41674	1.14911				0.73119	4.74677
SFP1	YGR159C		0.73344	9.04021					0.677433	10.4511
SFP1	YGR213C			4.36637						4.36637
SFP1	YHL015W	0.756865		5.29949	0.835372		0.775847		0.732108	8.39968
SFP1	YHR021C	2.6608		2.2946	0.766581				0.729462	6.45144
SFP1	YIL018W	0.583709	0.73344	0.901819	1.08733				0.730612	4.03691
SFP1	YIL023C			4.00766						4.00766
SFP1	YIL133C	2.59466		0.0972045	1.18621				0.732757	4.61083
SFP1	YIL149C	0.696481	0.592597	3.31993						4.60901
SFP1	YJL098W			5.73285					0.732367	6.46522
SFP1	YJL134W	3.14119	0.784553	2.71599						6.64173
SFP1	YJL136C	3.14119	0.784553		1.08549				0.728163	5.7394
SFP1	YJL184W	0.150754		5.92695						6.0777
SFP1	YJL190C	0.771385		4.85951	1.07718				0.731944	7.44002
SFP1	YJL200C	2.95349		0.538867					0.732601	4.22496
SFP1	YJR014W	0.317269		3.81344					0.579316	4.71002
SFP1	YJR123W	2.88187	0.73344	8.3982	1.30037				0.730495	14.0444
SFP1	YJR145C	0.466874		2.90163	1.1663				0.7321	5.2669
SFP1	YJR147W	0.466874		6.56119						7.02806
SFP1	YKL180W	0.731296	0.592597	2.08595	0.843012				0.731451	4.98431
SFP1	YLL045C	1.06805	0.592597	2.84185	0.945035				0.732701	6.18023
SFP1	YLR042C	1.76008		2.07637					0.504447	4.3409
SFP1	YLR047C	0.102159	0.592597	7.48042						8.17518
SFP1	YLR061W	5.11876	0.301244		1.21311				0.724454	7.35757
SFP1	YLR063W	5.11876	0.301244						0.726197	6.1462
SFP1	YLR074C	0.185183		4.0139	0.416662				0.586315	5.20206
SFP1	YLR075W			3.14745	0.96857				0.726646	4.84267
SFP1	YLR167W	1.03922		2.41811	1.00294				0.732273	5.19254
SFP1	YLR183C	2.76818	0.356388	4.56149						7.68606
SFP1	YLR185W	2.76818	0.356388		0.933458				0.727582	4.78561
SFP1	YLR186W	2.76818	0.356388		0.328934				0.725587	4.17909
SFP1	YLR201C		0.301244	3.98357						4.28481
SFP1	YLR287C-A	1.0749	0.592597	2.27512	0.355347				0.566434	4.8644
SFP1	YLR372W		1.00391	5.81948	0.725337				0.732009	8.28074
SFP1	YLR381W		0.592597	7.6602						8.2528
SFP1	YLR447C	1.23923	0.73344	3.00123					0.688311	5.66221
SFP1	YLR448W	1.23923	0.73344	1.77273	1.07596				0.728588	5.54995
SFP1	YML024W	2.28568	0.592597	1.1028	1.10243				0.730225	5.81373
SFP1	YML025C	2.28568	0.592597	3.50434						6.38262
SFP1	YML026C	2.11962	0.592597	0.558554	1.11891				0.724718	5.1144
SFP1	YML063W	1.8178	0.0522983	0.716307	1.34971				0.728705	4.66482
SFP1	YML073C	3.30797	0.592597		1.06695				0.728154	5.69567
SFP1	YMR013C		0.73344	2.79793					0.471388	4.00276
SFP1	YMR014W	0.72413	0.73344	2.98649	0.345193				0.731704	5.52096
SFP1	YMR116C		0.592597	2.36708	0.799047				0.730888	4.48961
SFP1	YMR142C	2.80762	0.73344	0.404435	1.19594				0.730592	5.87203
SFP1	YMR144W	1.61533	0.73344	3.28504					0.509001	6.14281
SFP1	YMR194W	1.76591	1.00391	1.97029	1.11371				0.713101	6.56692
SFP1	YMR195W			4.02991						4.02991
SFP1	YMR205C	0.468978	1.00391	3.80906						5.28195
SFP1	YMR206W	0.468978	1.00391	3.72246						5.19535
SFP1	YNL069C	2.67767	0.356388		1.08413		0.775566		0.731454	5.62521
SFP1	YNL162W	2.34338			1.03921				0.7309	4.11349
SFP1	YNL178W			1.74981	1.24839		0.776718		0.691676	4.46659
SFP1	YNR019W	0.815938	0.592597	9.44381						10.8523
SFP1	YOL039W	0.807454	0.356388	5.16506	0.817902				0.732698	7.8795
SFP1	YOL040C	0.723042	0.356388	4.88657	1.0304		0.777982		0.728934	8.50332
SFP1	YOL086C		0.73344	3.35257						4.08601
SFP1	YOL109W			9.25325	0.392275					9.64552
SFP1	YOL111C			8.29658						8.29658
SFP1	YOL121C	1.9821	1.00391	2.62438	1.19395				0.722269	7.52661
SFP1	YOL127W	1.04292	1.00391	1.91233	1.17956				0.717543	5.85626
SFP1	YOL128C	1.04292	1.00391	10.062						12.1088
SFP1	YOR095C		0.503769	3.62223	0.582134				0.730202	5.43834
SFP1	YOR184W	0.311883	0.592597	4.73886						5.64334
SFP1	YOR218C			6.31513						6.31513
SFP1	YOR234C	2.12255		1.35431	1.28501				0.728391	5.49026
SFP1	YOR312C	2.3763	1.00391	3.44721	1.03104				0.730976	8.58944
SFP1	YOR369C	0.870549	0.592597	2.45695	1.13963				0.732475	5.7922
SFP1	YPL090C	2.18599	0.73344		1.21645				0.732003	4.86788
SFP1	YPL199C		0.592597	5.89093	0.561869					7.0454
SFP1	YPL220W			2.4246	1.17885				0.730958	4.33441
SFP1	YPL249C-A	1.12333	1.00391	10.1603	0.617301					12.9048
SFP1	YPR042C	1.38182	0.592597	3.71653						5.69095
SFP1	YPR102C	4.39505		1.66241	1.36095				0.730832	8.14924
SFP1	YPR103W	4.39505		2.39956						6.79461
SFP1	YPR105C	0.356901	0.73344	3.34236						4.4327
SFP1	YPR106W	0.356901	0.73344	2.9339						4.02424
SFP1	YPR110C		0.503769	3.53575	1.0774				0.732402	5.84932
SFP1	YPR131C	1.65278	0.73344	5.03336						7.41958
SFP1	YPR132W	1.65278	0.73344		1.27289				0.732361	4.39147
SFP1	YPR161C	0.831666	0.503769	4.47906						5.81449

TF	Target	Binding_0-2000	Motif_Score_SC	Other_Yeasts	Two-Hop_BE	Two-Hop_BP	Two-Hop_BY	Two-Hop_BG	Max_Membership	Sum
SIG1	TE(CUC)D	3.51136	0.970222							4.48158
SIG1	TE(UUC)E2	4.93164								4.93164
SIG1	TF(GAA)F	4.31857	0.301888							4.62046
SIG1	TK(CUU)C	2.99815	1.50061							4.49876
SIG1	YBL071W-A	1.27388		3.76978						5.04366
SIG1	YBL072C			3.90193	0.397329					4.29926
SIG1	YBR112C		1.50061	3.41931						4.91992
SIG1	YBR161W	0.352001		6.03692						6.38892
SIG1	YBR211C	0.41748	1.49383	2.57861						4.48992
SIG1	YCL021W-A			4.47187						4.47187
SIG1	YCR019W	2.99815	1.50061							4.49876
SIG1	YCR052W	0.136296	1.38259	3.14181						4.6607
SIG1	YCR105W	5.89466	0.377406	0.268609						6.54067
SIG1	YDL113C		0.928844	3.58458						4.51342
SIG1	YDL154W	2.15601	1.03513	1.63025						4.82139
SIG1	YDR249C		1.50061	2.65407						4.15468
SIG1	YDR250C		1.50061	2.53653						4.03714
SIG1	YDR279W	3.51136	0.970222							4.48158
SIG1	YDR280W		1.15942	3.27181						4.43123
SIG1	YEL007W			4.07143						4.07143
SIG1	YEL038W		0.928844	4.4818						5.41064
SIG1	YEL042W	0.2864		5.11088						5.39728
SIG1	YER077C			5.99361						5.99361
SIG1	YER098W	4.93164								4.93164
SIG1	YFR007W	4.31857	0.301888	0.263084						4.88354
SIG1	YFR008W	4.31857	0.301888							4.62046
SIG1	YGL028C			6.42544						6.42544
SIG1	YGR060W	0.538011	1.50061	2.79793						4.83655
SIG1	YGR242W		1.50061	2.70083						4.20144
SIG1	YHL008C			4.91518						4.91518
SIG1	YHR156C	5.48492	0.379948							5.86487
SIG1	YHR157W	5.48492	0.379948							5.86487
SIG1	YHR178W			4.83137						4.83137
SIG1	YIL134W			4.59178						4.59178
SIG1	YIL135C		0.494436	4.39901						4.89345
SIG1	YIR026C		0.928844	4.49718						5.42602
SIG1	YJL160C			8.27332						8.27332
SIG1	YJR080C	0.725772	0.236442	4.60103						5.56324
SIG1	YKL007W	0.190609		3.89091						4.08152
SIG1	YKL092C		0.986803	6.3434						7.3302
SIG1	YKR007W			4.15267						4.15267
SIG1	YKR095W			10.5491						10.5491
SIG1	YLR028C		1.15277	5.63863						6.7914
SIG1	YLR215C			9.70345						9.70345
SIG1	YLR314C			5.34166						5.34166
SIG1	YLR315W			5.403						5.403
SIG1	YNL015W		0.0965604	5.28019						5.37675
SIG1	YNL213C	0.207051		4.01317						4.22022
SIG1	YNL250W	0.618143		7.47557						8.09371
SIG1	YOL038W		1.0965	4.4342						5.5307
SIG1	YOL040C		1.0965	4.40675	0.456177					5.95943
SIG1	YOR069W			4.28348						4.28348
SIG1	YOR089C		1.43611	4.38634						5.82245
SIG1	YOR187W	0.614196		4.34166						4.95586
SIG1	YOR303W			4.28914			0.835972			5.12511
SIG1	YPR118W		1.15942	3.48368						4.6431
SIG1	YPR151C		1.49383	2.93494						4.42877
SIG1	YPR165W	0.0568947	1.09706	3.462						4.61595
SIP4	TQ(CUG)M	3.25812	1.1261							4.38422
SIP4	TS(UGA)E	4.09952	0.295442							4.39496
SIP4	YAL053W		1.2577	6.62844						7.88614
SIP4	YAL054C		1.2577	5.27293	3.04784		1.87534		0.463455	11.9173
SIP4	YAR035W			3.31613	2.69806					6.01419
SIP4	YBL015W				3.15545		2.00234			5.15779
SIP4	YBL045C		1.47081		2.63557					4.10638
SIP4	YBL049W		0.515569	0.611086	2.7789				0.705655	4.61121
SIP4	YCR004C		1.40119	4.18747						5.58866
SIP4	YCR005C		1.80169	15.2863			1.89669		0.459955	19.4446
SIP4	YCR082W		1.18539	5.83707						7.02246
SIP4	YDL085C-A		0.134511	5.84586						5.98037
SIP4	YDL133W		0.753108	5.38272						6.13583
SIP4	YDR086C	1.37369	1.0965	2.28574						4.75593
SIP4	YDR099W		1.23479	5.94491						7.1797
SIP4	YDR148C			4.60158	2.55794					7.15952
SIP4	YDR256C		0.674024	0.465631	2.24771		1.99446			5.38183
SIP4	YEL019C		1.08528	5.33292						6.4182
SIP4	YER024W	2.1394		0.54141	2.6208					5.30161
SIP4	YER065C	15.8551	2.63184	21.9017	2.45345		2.11022	2.9908		47.9431
SIP4	YER066W	4.09952								4.09952
SIP4	YER189W	2.30695	0.919607					0.974805		4.20136
SIP4	YER190W	2.30695	0.919607				2.00244	0.974805		6.2038
SIP4	YFL055W	0.721782	1.39694	1.03091	2.46077					5.6104
SIP4	YGL023C				2.51624		1.94048			4.45672
SIP4	YGL126W		1.12498	6.19233						7.31731
SIP4	YGL128C		1.24735	4.94067						6.18802
SIP4	YGR077C			2.05323	2.51537					4.5686
SIP4	YGR218W	1.41782				4.11544				5.53326
SIP4	YIL146C		1.00561	4.40455						5.41016
SIP4	YIR031C						2.11022	2.98682		5.09704
SIP4	YJL144W		1.14334	4.22464						5.36798
SIP4	YJR076C			4.29776						4.29776
SIP4	YJR095W	15.8551	2.2466	15.7231	3.44552					37.2703
SIP4	YKL058W	0.439627		3.64956						4.08919
SIP4	YKR058W		2.63184				2.08494			4.71678
SIP4	YKR097W	15.8551	2.42879	15.0223	3.26571					36.5719
SIP4	YLR153C		1.40119	2.38582			1.87534			5.66235
SIP4	YLR154C		1.40119	3.4901						4.89129
SIP4	YLR254C		1.23862	1.36372			2.01605			4.61839
SIP4	YLR308W	15.8551	2.45396	10.2383						28.5474
SIP4	YLR377C		2.47464	0.904865	3.46977					6.84928
SIP4	YML042W	0.496801	1.15998	4.33709					0.707445	6.70132
SIP4	YML043C	0.496801	1.15998	4.30535						5.96213
SIP4	YML091C		0.634064	0.0413062		3.9249				4.60027
SIP4	YMR080C		0.688485			4.11544				4.80393
SIP4	YMR271C	3.25812	1.1261	1.52078						5.905
SIP4	YNL023C		1.40119	4.22344						5.62463
SIP4	YNL045W	0.648263		1.51146			2.04728			4.207
SIP4	YNL052W			1.78546	2.77631					4.56177
SIP4	YNL054W			0.269508	2.32199		2.01202			4.60352
SIP4	YNL116W	4.01152								4.01152

TF	Target	Binding_0-2000	Motif_Score_SC	Other_Yeasts	Two-Hop_BE	Two-Hop_BP	Two-Hop_BY	Two-Hop_BG	Max_Membership	Sum
SIP4	YNL117W	15.8551	2.19701	9.81854	3.44552		2.11022	2.9908		36.4172
SIP4	YNL118C	15.8551	2.19701	7.06081						25.1129
SIP4	YNR008W		1.53544	2.78059						4.31603
SIP4	YOL125W	2.63071	1.98751	13.3546						17.9728
SIP4	YOL126C	2.63071	1.98751	9.48468						14.1029
SIP4	YOL149W			3.71773		4.11544				7.83317
SIP4	YOR095C	2.78837	1.87636	10.8575	0.439723					15.962
SIP4	YOR096W	2.78837	1.87636		0.662416					5.32715
SIP4	YOR100C		1.63103	3.93046	2.6813					8.24279
SIP4	YOR101W		1.63103	2.46608						4.09711
SIP4	YOR196C			4.33489						4.33489
SIP4	YPL051W				2.37282		1.94048			4.3133
SIP4	YPL119C	0.235523	2.23424	2.04535	0.369861				0.52046	5.40543
SIP4	YPL235W	0.59728		3.92864						4.52592
SIP4	YPL236C	0.59728	0.210966	3.82285						4.6311
SIP4	YPR006C						2.1148	2.99258		5.10738
SIP4	YPR009W		1.30377	4.37892						5.68269
SKN7	YBL072C	0.312114	0.0831841	1.82086	0.797722		0.998151			4.01203
SKN7	YBL099W	4.23553	0.756677	1.55558	0.364915	0.768689			0.709026	8.39042
SKN7	YBR053C	3.73155			0.618254				0.725525	5.07533
SKN7	YBR066C	3.6052			0.570083					4.17528
SKN7	YBR078W	3.58224			0.920363					4.5026
SKN7	YBR126C			5.44846	0.437975	0.518656			0.723196	7.12829
SKN7	YBR157C	3.57222							0.495954	4.06817
SKN7	YCL057C-A		0.753108	4.37287						5.12598
SKN7	YCR002C			4.19325						4.19325
SKN7	YCR021C	4.00033							0.494109	4.49444
SKN7	YDL004W			6.99569	0.336161	0.978684			0.72703	9.03756
SKN7	YDL005C			6.94669		1.04867				7.99536
SKN7	YDL006W	3.36814	0.287697				0.880011			4.53585
SKN7	YDL022W	2.23449		0.583011	0.768653				0.689215	4.27537
SKN7	YDL049C	4.43187	0.0144696	1.94792						6.39426
SKN7	YDL137W	3.44247		0.38443		0.455577	1.02354			5.30602
SKN7	YDR153C	4.61967	0.897031							5.5167
SKN7	YDR155C	4.61967	0.897031	1.07391		0.362316				6.95293
SKN7	YDR156W	4.61967	0.341052							4.96072
SKN7	YDR158W	4.61967	0.897031	1.0414						6.5581
SKN7	YDR171W	3.61391	0.671006		1.00682				0.708334	6.00007
SKN7	YDR309C	4.46315			1.14738					5.61053
SKN7	YDR343C	0.306604	0.979948	1.64555	0.546385				0.572949	4.05144
SKN7	YDR513W		0.749537	2.55309	1.17244			1.77533	0.718033	6.96843
SKN7	YDR518W			3.33018	0.73259		0.8163			4.87907
SKN7	YDR524C	8.04529	0.0144696							8.05976
SKN7	YDR524C-B	8.04529	0.0144696							8.05976
SKN7	YDR525W-A	8.04529	0.0144696	5.04182						13.1016
SKN7	YDR527W	8.04529	0.0144696	5.07234	0.540825					13.6729
SKN7	YEL043W	4.83559			1.14733					5.98292
SKN7	YEL044W	4.83559								4.83559
SKN7	YEL046C	4.83559								4.83559
SKN7	YEL056W		0.688485	2.82585			1.08742			4.60175
SKN7	YER010C	4.54101	0.453812							4.99482
SKN7	YER011W	4.54101	0.453812	1.85665						6.85147
SKN7	YER012W	1.83847	0.908041	1.34652		0.531015	0.953027			5.57707
SKN7	YER018C			6.67353						6.67353
SKN7	YER088C	1.51929	0.760837	7.98054	0.393958				0.497157	11.1518
SKN7	YER098W	1.27225			1.42589		1.02354	1.55293		5.27461
SKN7	YER149C	3.10518		1.30973						4.41491
SKN7	YER150W	3.10518		2.558	1.29715				0.709146	7.66948
SKN7	YER152C	3.83969	0.908041		1.35077					6.0985
SKN7	YER153C	3.83969	0.908041	0.824719						5.57245
SKN7	YER154W	3.83969	0.362765		1.35057					5.55303
SKN7	YER177W	0.787528	0.908041	4.57425	0.80226					7.07208
SKN7	YFL021W	2.75737	0.982805	4.1057						7.84587
SKN7	YFL022C	2.75737	0.982805	3.94881						7.68898
SKN7	YFL030W	0.738546	0.666779	2.31496					0.725643	4.44593
SKN7	YFR033C	1.07236	0.671006	6.05706	0.385283				0.627097	8.81281
SKN7	YGL036W	4.56624	0.756677						0.703743	6.02666
SKN7	YGL038C	4.56624	0.756677	4.27376						9.59668
SKN7	YGL056C	2.43614		1.59451						4.03065
SKN7	YGR023W	0.974566	0.975945	3.55619					0.505093	6.01179
SKN7	YGR043C	1.7778			1.71185				0.723278	4.21293
SKN7	YGR052W	5.19272	0.362765	1.4736	0.54573					7.57481
SKN7	YGR086C	4.20089	0.897031	5.34556					0.45554	10.899
SKN7	YGR088W	2.7286		0.830355	1.59322				0.704611	5.85679
SKN7	YGR152C	0.485624			0.558746		1.02354	2.00841		4.07632
SKN7	YGR173W	0.173108	0.453812	3.1627	0.752833	0.429073				4.97153
SKN7	YGR207C	6.63606						0.900758		7.53682
SKN7	YGR209C	6.63606			0.644488				0.712171	7.99272
SKN7	YGR211W	6.63606								6.63606
SKN7	YGR243W			3.74109	0.871812				0.460407	5.07331
SKN7	YGR249W	5.16729	0.674024	1.03461						6.87592
SKN7	YGR279C	3.54661	0.00972326		0.519665					4.076
SKN7	YHR002W	3.00402		1.18668	0.433841					4.62454
SKN7	YHR007C	0.169698	0.897031	4.7722						5.83893
SKN7	YHR203C			4.15647	0.858412					5.01488
SKN7	YHR204W			5.89371						5.89371
SKN7	YIL053W			6.36112					0.720691	7.08181
SKN7	YIL099W	7.03375	0.353835	0.36124	0.848105				0.641495	9.23843
SKN7	YIL101C	7.03375	0.975945		0.848105				0.65717	9.51497
SKN7	YIL113W	1.95229	0.671006	1.17955	1.0896	0.855867				5.74831
SKN7	YIL115C	1.95229	0.671006	0.448608	1.66371			1.29209		6.0277
SKN7	YIL118W	6.18718	0.982805	1.41285						8.58283
SKN7	YIL119C	6.18718	0.982805	2.03299					0.600154	9.80313
SKN7	YIR017C	1.76213	0.982805	2.57748					0.451221	5.77364
SKN7	YIR018W	1.76213	0.982805	4.6207						7.36564
SKN7	YJL042W		0.986803	3.76465						4.75145
SKN7	YJL115W	3.9542		0.0538503						4.00805
SKN7	YJL116C	3.9542		0.0598217						4.01402
SKN7	YJL158C	6.19301	0.00972326		0.952978					7.15571
SKN7	YJR077C	5.90797	0.90051	3.60721					0.717983	11.1337
SKN7	YJR078W	5.90797	0.90051	3.43744					0.441297	10.6872
SKN7	YJR079W	5.90797	0.90051						0.560305	7.36878
SKN7	YJR148W	4.4207	0.0998995							4.5206
SKN7	YJR149W	4.4207								4.4207
SKN7	YKL032C		0.353835	9.96169						10.3155
SKN7	YKL035W	2.19726	0.366588	0.732107	0.34001				0.666162	4.30213
SKN7	YKL062W	4.05289	0.0670908	0.0430238	0.398858					4.56186
SKN7	YKL063C	4.05289	0.0670908							4.11998
SKN7	YKL085W	5.09455	0.756677	1.47778					0.728468	8.05748
SKN7	YKL086W	5.09455	0.756677							5.85123

TF	Target	Binding_0-2000	Motif_Score_SC	Other_Yeasts	Two-Hop_BE	Two-Hop_BP	Two-Hop_BY	Two-Hop_BG	Max_Membership	Sum
SKN7	YKL087C	5.09455	0.756677	1.49892	1.02693					8.37708
SKN7	YKL096W-A	6.66492	0.975945	6.26762	0.859601					14.7681
SKN7	YKL109W	3.46147	0.609098	5.80968						9.88025
SKN7	YKL110C	3.25148	0.609098	0.335071						4.19565
SKN7	YKR042W	8.19338	0.982805	3.87515	0.544356					13.5957
SKN7	YKR052C	3.3476		0.232173	0.635539					4.21531
SKN7	YKR066C	1.02863	0.756677	4.32108					0.452703	6.55909
SKN7	YKR067W	1.02863	0.756677	3.78076	0.865065				0.730448	7.16158
SKN7	YKR092C	4.90265	0.674024							5.57667
SKN7	YKR093W	4.90265	0.674024		0.516125					6.0928
SKN7	YKR097W	3.8921	0.265046							4.15715
SKN7	YLL018C-A	4.02701								4.02701
SKN7	YLL019C	5.4264	0.692696	2.57583					0.62034	9.31527
SKN7	YLR037C	7.26588								7.26588
SKN7	YLR108C	6.7728		0.0673007	0.684104					7.5242
SKN7	YLR109W	6.7728			0.812356				0.669506	8.25466
SKN7	YLR110C	3.43314						1.44355		4.87669
SKN7	YLR113W	3.43314		0.832692		1.03592				5.30175
SKN7	YLR121C	0.851682	0.982805	1.9458	1.49828					5.27857
SKN7	YLR178C			1.81748	1.55531		1.07815		0.706453	5.15739
SKN7	YLR257W	3.76971			1.25812				0.488321	5.51615
SKN7	YLR293C	3.45764	0.337212		0.703435					4.49829
SKN7	YLR295C	3.45764	0.337212	0.0416114		0.978684				4.81515
SKN7	YLR297W	3.45764	0.337212	0.725679					0.499278	5.01981
SKN7	YLR304C	3.17113	0.982805		0.869829				0.625099	5.64886
SKN7	YLR348C			4.17499						4.17499
SKN7	YLR350W			4.04516	0.911196					4.95636
SKN7	YLR414C	3.38622	0.0912137		0.744955					4.22239
SKN7	YML007W	4.55276	0.753108							5.30587
SKN7	YML008C	4.55276	0.753108	0.729202						6.03507
SKN7	YML027W	7.47242								7.47242
SKN7	YML028W	7.47242			0.88921					8.36163
SKN7	YML052W	2.93244	0.0831841	1.28876	1.10827					5.41265
SKN7	YML053C	2.93244	0.0831841	1.21771						4.23333
SKN7	YML072C		0.365951	5.21121			1.02354	1.58382		8.18452
SKN7	YML100W	1.26668		1.67249	0.953977				0.620194	4.51334
SKN7	YMR016C	8.04329								8.04329
SKN7	YMR017W	8.04329		0.397968						8.44126
SKN7	YMR055C	2.95865		1.27243						4.23108
SKN7	YMR070W	3.38155	0.975945							4.3575
SKN7	YMR135C	7.06337	0.975945	3.15454						11.1939
SKN7	YMR136W	7.06337	0.975945	3.26616						11.3055
SKN7	YMR171C	4.44754								4.44754
SKN7	YMR172W	4.44754	0.0670908			0.762076				5.27671
SKN7	YMR173W	5.71296	0.688485	0.53373	1.24862				0.728887	8.91268
SKN7	YMR195W	1.04312	0.671006	1.89515					0.663736	4.27301
SKN7	YMR251W-A	5.53755	0.269583		1.21192				0.656633	7.67569
SKN7	YMR266W	0.488552	0.0543177	4.95436						5.49723
SKN7	YMR305C	3.81479		0.802794	0.358848					4.97643
SKN7	YMR306W	3.81479		0.810492						4.62528
SKN7	YNL014W	4.44335	0.197858	0.187729						4.82894
SKN7	YNL087W	2.62353	0.0361169		0.673742		0.834725			4.16811
SKN7	YNL098C	2.07537	0.604824	2.93785			0.968879	1.84959		8.43651
SKN7	YNL172W	4.24472		0.463141						4.70786
SKN7	YNL173C	4.24472		1.12318					0.695616	6.06352
SKN7	YNL175C	4.24472			1.31718					5.5619
SKN7	YNL178W	2.44271	0.986803	2.0446	0.910756		0.947739			7.33261
SKN7	YNL192W	4.76037			0.96736					5.72773
SKN7	YNL196C	0.682098	0.34489	3.68714	1.1113					5.82543
SKN7	YNL207W		0.015825	3.20992			1.00431			4.23006
SKN7	YNL233W	4.86541								4.86541
SKN7	YNL234W	4.86541								4.86541
SKN7	YNL241C	0.536542	0.366588	2.16518	1.03238					4.10069
SKN7	YNL304W	0.542965	0.90051	6.0451			0.886461			8.37504
SKN7	YNL305C		0.90051	4.86667	1.16126				0.580052	7.50849
SKN7	YNR001C	0.65881	0.362765	1.40109	0.709358	1.21058			0.676201	5.0188
SKN7	YNR016C	0.454	0.760837	2.14857				0.918661		4.28207
SKN7	YNR017W	0.454	0.760837	4.32869						5.54353
SKN7	YNR018W	2.77802	0.205727	1.25763						4.24138
SKN7	YNR019W	2.77802	0.692696	0.601546	0.671189					4.74345
SKN7	YNR042W			4.24295						4.24295
SKN7	YOL005C			2.88287	0.629397		1.00565			4.51792
SKN7	YOL039W	2.57721		0.850896	0.632043					4.06015
SKN7	YOL040C	2.57721		0.871709	0.938814		0.980071			5.3678
SKN7	YOL052C-A	8.22897		0.447741	0.469628					9.14634
SKN7	YOL058W	3.53963	0.453812	0.308919						4.30236
SKN7	YOL109W	6.71027	0.277353	0.27871	0.850337					8.11667
SKN7	YOL111C	6.71027	0.277353	0.287221						7.27484
SKN7	YOL125W	4.73126								4.73126
SKN7	YOL126C	4.73126			0.639995					5.37125
SKN7	YOR036W	0.202555	0.692696		0.348204	2.0066	1.02102			4.27107
SKN7	YOR101W	1.45912					1.02354	2.00828		4.49094
SKN7	YOR138C		0.979948	3.73939						4.71934
SKN7	YOR140W			5.78514						5.78514
SKN7	YOR208W	2.32522			1.10686	0.762076				4.19416
SKN7	YOR224C	3.16229	0.756677	2.13618	0.394203					6.44935
SKN7	YOR226C	3.16229	0.756677	2.02605						5.94502
SKN7	YOR227W	3.16229	0.756677	2.13689						6.05586
SKN7	YOR273C	3.31338	0.197858	0.167967					0.673789	4.35299
SKN7	YOR327C	1.07313				2.0066	0.953944			4.03367
SKN7	YOR348C	2.53441	0.604824	0.561698	0.737791				0.683086	5.12181
SKN7	YOR374W		0.365951	8.92424	0.386111				0.710763	10.3871
SKN7	YPL024W	6.62295		2.86143						9.48438
SKN7	YPL026C	6.62295		1.28879						7.91174
SKN7	YPL041C	5.20058	0.609098	0.427821						6.2375
SKN7	YPL042C	5.20058	0.609098							5.80968
SKN7	YPL055C		0.903987	6.19563						7.09962
SKN7	YPL061W		0.986803	11.0768						12.0636
SKN7	YPL230W	1.99342	0.44942	3.60279	0.935676				0.639697	7.621
SKN7	YPL232W	0.174054		2.22286		2.0066	1.00946			5.41297
SKN7	YPL236C			2.77507				1.32534		4.10041
SKN7	YPR004C	1.89603					1.0388	1.75489	0.561176	5.2509
SKN7	YPR009W	4.08147								4.08147
SKN7	YPR063C	4.06796	0.366588		0.327525					4.76207
SKN7	YPR065W	4.06796	0.366588	1.06008						5.49463
SKN7	YPR066W	4.06796								4.06796
SKN7	YPR149W	3.60925			0.409898				0.726529	4.74568
SKN7	YPR151C	1.77		2.95944						4.72944
SKO1	YER075C	1.18539		4.59956						5.78495
SKO1	YGL254W			5.77914						5.77914

TF	Target	Binding_0-2000	Motif_Score_SC	Other_Yeasts	Two-Hop_BE	Two-Hop_BP	Two-Hop_BY	Two-Hop_BG	Max_Membership	Sum
SKO1	YHR033W	2.5658		1.85785						4.42365
SKO1	YJL106W	1.75042		2.92547						4.67589
SKO1	YMR247C	0.244475		4.75054						4.99502
SKO1	YMR252C			5.19832						5.19832
SKO1	YMR253C			4.4103						4.4103
SKO1	YMR255W			4.20638						4.20638
SKO1	YPR062W			4.67526						4.67526
SMP1	TM(CAU)J3	4.08753								4.08753
SMP1	TS(AGA)D3	10.7046	0.0670908							10.7717
SMP1	YAL022C	4.63542								4.63542
SMP1	YBL027W	2.96012			0.789009		0.865157			4.61429
SMP1	YBL063W			0.644541	2.81269			1.40994		4.86717
SMP1	YBL087C	2.31585			2.32833					4.64418
SMP1	YBR010W				3.26626		0.789154			4.05541
SMP1	YBR046C	3.9295	1.42079							5.35029
SMP1	YBR048W	3.9295			1.10026		2.06992			7.09968
SMP1	YBR154C			1.97102	2.5195		0.810391			5.30091
SMP1	YBR182C	15.8551								15.8551
SMP1	YBR183W	15.8551								15.8551
SMP1	YBR191W	2.12904			2.24735		0.90412			5.28051
SMP1	YBR249C			4.35635						4.35635
SMP1	YBR250W			4.21142						4.21142
SMP1	YBR283C			1.80884	1.72381		0.845677			4.37833
SMP1	YCL067C	2.59045					2.00261			4.59306
SMP1	YCL069W		1.42079	3.2448						4.66559
SMP1	YDL004W			4.70262						4.70262
SMP1	YDL005C			3.83986		0.890021				4.72988
SMP1	YDL061C	1.93876			2.67996					4.61872
SMP1	YDR119W		1.42079		2.83797					4.25876
SMP1	YDR143C		0.0670908	4.39053						4.45762
SMP1	YDR161W		1.00789	6.88693						7.89482
SMP1	YDR286C		1.00789	5.19713						6.20502
SMP1	YDR288W		1.00789	6.14307						7.15096
SMP1	YDR350C	6.09946		1.10588						7.20534
SMP1	YDR351W	6.09946		1.61325						7.71271
SMP1	YDR418W	3.63194			1.16104		0.806457			5.59944
SMP1	YDR420W	15.8551	1.42079	5.5791						22.855
SMP1	YDR447C		1.31833		2.25779		0.842362			4.41848
SMP1	YDR449C			5.08333	0.711548					5.79488
SMP1	YDR450W			2.71761	2.28113					4.99874
SMP1	YEL011W			3.20091			2.02703			5.22794
SMP1	YEL040W	15.8551		1.07055						16.9257
SMP1	YEL065W		1.42079	3.68495						5.10574
SMP1	YER010C	9.62476								9.62476
SMP1	YER011W	9.62476								9.62476
SMP1	YER034W		1.00789	5.47214						6.48003
SMP1	YER168C				2.46865		2.07464			4.54329
SMP1	YFR048W			4.4743						4.4743
SMP1	YGL059W				2.49648		1.87829			4.37477
SMP1	YGL077C	1.72701			2.70128					4.42829
SMP1	YGL119W			4.44321						4.44321
SMP1	YGL120C			4.21589	0.708991	1.84662				6.7715
SMP1	YGL135W	1.90396			2.31345					4.21741
SMP1	YGL147C			1.01607	2.40005		0.809476			4.2256
SMP1	YGL178W			6.98939						6.98939
SMP1	YGL258W-A	4.88982						1.33197		6.22179
SMP1	YGL259W	4.88982						1.33197		6.22179
SMP1	YGL261C	4.88982								4.88982
SMP1	YGR118W	1.32058			2.37659		0.801188			4.49836
SMP1	YGR185C			0.119392	2.61751		2.02387			4.76077
SMP1	YGR282C	11.9838			3.10037					15.0842
SMP1	YHR024C						1.87029	2.92753		4.79782
SMP1	YHR062C			5.69649						5.69649
SMP1	YHR080C	3.96769		3.47478						7.44247
SMP1	YHR081W	3.96769		6.78453						10.7522
SMP1	YHR091C	7.07259								7.07259
SMP1	YHR181W		2.78979	5.53041						8.3202
SMP1	YHR191C	6.57961								6.57961
SMP1	YHR193C	6.57961								6.57961
SMP1	YHR194W	6.57961								6.57961
SMP1	YHR209W			5.40087	0.983655					6.38453
SMP1	YIL049W			6.71812	1.72621					8.44433
SMP1	YIL133C	2.84975			1.08439		0.78913			4.72327
SMP1	YIR013C	11.1589								11.1589
SMP1	YIR014W	11.1589								11.1589
SMP1	YIR015W	11.1589								11.1589
SMP1	YIR019C	6.22178		0.296061						6.51784
SMP1	YJL110C			4.713						4.713
SMP1	YJL191W	1.64402			2.5316					4.17562
SMP1	YJR044C	4.08753								4.08753
SMP1	YKL068W			2.27212				1.84884		4.12096
SMP1	YKL085W			4.36788						4.36788
SMP1	YKL095W	8.15647								8.15647
SMP1	YKL096C-B	8.15647								8.15647
SMP1	YKL096W	8.15647		1.03091						9.18738
SMP1	YKL121W		1.42079		1.59504		2.00261			5.01844
SMP1	YKL163W	13.2575			2.66356					15.9211
SMP1	YKL164C	13.2575			2.66356					15.9211
SMP1	YKL185W	2.23547			2.14206					4.37753
SMP1	YKR017C			4.16963						4.16963
SMP1	YKR026C			2.36336		2.28887				4.65223
SMP1	YKR051W			4.36799			2.00261			6.3706
SMP1	YKR102W	8.544								8.544
SMP1	YKR105C		1.42079	2.79793						4.21872
SMP1	YLR300W	5.26062								5.26062
SMP1	YLR340W			3.54521	1.9435					5.48871
SMP1	YLR389C	15.8551	1.00789	4.77165						21.6346
SMP1	YLR390W	9.353								9.353
SMP1	YLR390W-A	15.8551	1.00789	9.25825	2.30958					28.4308
SMP1	YMR012W	0.367794			2.96076		0.80053			4.12908
SMP1	YMR149W			5.6727						5.6727
SMP1	YMR317W						1.17939	3.04163		4.22102
SMP1	YNL113W	3.15248	1.11606	0.181561	0.502776					4.95288
SMP1	YNL236W	4.61289		0.345747		1.15533				6.11397
SMP1	YNL288W	4.07407								4.07407
SMP1	YNL327W			0.505197	2.41907		1.27897			4.20324
SMP1	YNL336W	9.67795			2.42517					12.1031
SMP1	YNL339C	9.67795								9.67795
SMP1	YOR019W			8.10384	1.59519					9.69903
SMP1	YOR137C	1.86816		1.1735	1.46846					4.51012

TF	Target	Binding_0-2000	Motif_Score_SC	Other_Yeasts	Two-Hop_BE	Two-Hop_BP	Two-Hop_BY	Two-Hop_BG	Max_Membership	Sum
SMP1	YOR138C	4.88989								4.88989
SMP1	YOR140W	4.88989				1.08055				5.97044
SMP1	YOR155C			4.55826						4.55826
SMP1	YOR271C			1.81748	2.84383					4.66131
SMP1	YOR272W			3.5486	2.4116		1.91333			7.87353
SMP1	YOR369C	1.39812			2.46843		0.976053			4.8426
SMP1	YPL059W	3.09065		1.10509						4.19574
SMP1	YPL090C	1.30165			2.86817		0.802979			4.9728
SMP1	YPL135W		1.42079	0.191376	2.93082		1.87592			6.41891
SMP1	YPL160W				2.29321		2.0689			4.36211
SMP1	YPL175W	5.11086								5.11086
SMP1	YPL176C	5.11086								5.11086
SMP1	YPL192C	1.02981		3.60369						4.6335
SMP1	YPL228W			5.71844						5.71844
SMP1	YPL253C	4.72174								4.72174
SMP1	YPR029C			2.11478			1.91551			4.03029
SMP1	YPR124W	15.8551								15.8551
SMP1	YPR198W	2.77828	1.31833	1.61647						5.71308
SNT2	SNR56	8.46718	2.34674							10.8139
SNT2	YAL038W	1.64547	2.51969	2.69295						6.85811
SNT2	YAL039C	7.5504	2.51969	14.5674						24.6375
SNT2	YBL073W		2.34674	3.2811						5.62784
SNT2	YBL075C	8.46718	2.34674	7.07845						17.8924
SNT2	YBR121C		1.55149	2.27228	0.876712					4.70048
SNT2	YBR138C	4.10965	1.96353	7.158						13.2312
SNT2	YBR139W	4.10965	1.96353	9.08634					0.729882	15.8894
SNT2	YBR143C	5.76346	2.34674	9.64537						17.7556
SNT2	YBR145W	5.76346	2.34674							8.1102
SNT2	YCL038C	0.0867496	2.34674	13.6464						16.0799
SNT2	YCR010C		0.623726	3.57265						4.19638
SNT2	YCR028C-A		1.13445	3.64569						4.78014
SNT2	YCR090C	7.98221	2.34674	2.34128						12.6702
SNT2	YCR091W	7.98221	2.34674	13.9031	1.48275					25.7148
SNT2	YDL195W			0.876162		1.56802	1.05868	1.64768		5.15054
SNT2	YDL214C		0.683669	10.0606						10.7443
SNT2	YDR046C	1.00121	2.07582	5.00358						8.08061
SNT2	YDR047W	1.00121	2.07582	6.96581						10.0428
SNT2	YDR233C		2.2571	4.04345						6.30055
SNT2	YDR282C		1.25661	2.77463	0.922708					4.95395
SNT2	YDR498C		1.66253	4.92898						6.59151
SNT2	YDR499W		1.66253	5.10531		0.609648				7.37749
SNT2	YER134C			4.74084						4.74084
SNT2	YER145C			6.15914						6.15914
SNT2	YER146W			5.86053						5.86053
SNT2	YER177W			2.75467		1.30786				4.06253
SNT2	YER189W	2.97478			0.364375			1.09515		4.43431
SNT2	YER190W	2.97478			0.364375			1.09515		4.43431
SNT2	YFR017C		0.930574	0.519764	1.61742	1.65462				4.72238
SNT2	YFR036W		0.0117579	5.25702						5.26878
SNT2	YGL006W			5.09621						5.09621
SNT2	YGL106W				1.25304		0.964486	1.99746		4.21499
SNT2	YGL153W		2.07582	2.23511						4.31093
SNT2	YGL206C	0.103472	2.07582	2.37845						4.55774
SNT2	YGR110W	1.25561		1.86764	1.20788					4.33113
SNT2	YGR162W	0.648054	1.79972	2.5957	0.664757					5.70823
SNT2	YHR138C	3.43015	2.51969	11.539	0.896557				0.514583	18.9
SNT2	YHR160C	1.02574	1.84799	11.6408	0.722443	2.08935				17.3263
SNT2	YIL159W	8.34988	2.51969	8.42062						19.2902
SNT2	YIL160C	8.34988	2.51969	9.64755						20.5171
SNT2	YJL092W	6.6711	2.34674	7.19018						16.208
SNT2	YJL093C	6.6711	2.34674	7.91345						16.9313
SNT2	YJL180C		1.25661	2.99432						4.25093
SNT2	YJR130C	1.67669	1.60293	4.01684						7.29646
SNT2	YJR131W	1.67669	1.60293	3.35826						6.63788
SNT2	YKL141W		0.926536	3.88118						4.80772
SNT2	YKL190W			1.16972			0.965116	1.99746		4.1323
SNT2	YKR019C		1.29026	4.8822						6.17246
SNT2	YKR021W		1.29026	3.71506						5.00532
SNT2	YKR105C		1.78535	2.64088						4.42623
SNT2	YLR149C	7.12443	2.2571	5.79663	1.25446				0.72233	17.1549
SNT2	YLR150W	7.12443	2.2571	5.48037						14.8619
SNT2	YLR176C	4.92621	2.2571	2.36134						9.54465
SNT2	YLR177W	4.92621	2.2571	1.90419					0.576323	9.66382
SNT2	YLR211C		1.10768	7.06917						8.17685
SNT2	YLR228C	2.03052	1.67266	5.38386						9.08704
SNT2	YLR327C		1.36071	4.85848	1.19047				0.662571	8.07223
SNT2	YLR446W		1.35537	5.32375						6.67912
SNT2	YML054C	0.500756	1.1633	4.29843	0.953592					6.91608
SNT2	YML100W		0.306396	2.21334	1.03712				0.708774	4.26563
SNT2	YML120C		1.938	1.52078			1.18268			4.64146
SNT2	YMR251W-A		2.2571	9.10595	1.06999				0.622878	13.0559
SNT2	YMR317W		1.938	1.63452				1.05975		4.63227
SNT2	YNL022C		1.1727	2.63623	1.71778					5.52671
SNT2	YNL078W		1.67266	10.0278						11.7005
SNT2	YNL079C		1.67266	10.1543	0.695694					12.5227
SNT2	YNL181W	8.58061	2.07582	5.07769						15.7341
SNT2	YNL182C	8.58061	2.07582	6.59562						17.2521
SNT2	YNR060W		1.06618	3.5666						4.63278
SNT2	YOL020W		1.79972	3.40715						5.20687
SNT2	YOL041C		0.962773	2.3996	1.38815					4.75052
SNT2	YOL115W	7.17547								7.17547
SNT2	YOL116W	7.17547								7.17547
SNT2	YOR084W		1.07742	4.42848						5.5059
SNT2	YOR148C		2.07582	6.07512						8.15094
SNT2	YOR369C		0.483839	4.21558			0.786741			5.48616
SNT2	YPL259C	0.438126	2.51969	6.38931						9.34713
SNT2	YPR104C	4.31014	2.07582	8.32079						14.7067
SNT2	YPR120C		1.21454	3.12473						4.33927
SNT2	YPR158W	7.25044	2.07582	10.2494						19.5757
SNT2	YPR184W	8.13684	2.51969	10.0378	1.45115				0.465802	22.6113
SOK2	YBL029C-A	3.17212		4.56551						7.73763
SOK2	YBL029W	3.17212		5.21042						8.38254
SOK2	YDR077W	4.34949		0.214796						4.56429
SOK2	YDR408C			4.55665						4.55665
SOK2	YDR447C			4.21591	0.552193					4.7681
SOK2	YDR448W			5.57115						5.57115
SOK2	YEL040W	3.71854		1.28879						5.00733
SOK2	YER114C			4.03628						4.03628
SOK2	YGR078C	0.322923		6.36858						6.6915
SOK2	YGR079W	0.322923		5.5663						5.88922

TF	Target	Binding_0-2000	Motif_Score_SC	Other_Yeasts	Two-Hop_BE	Two-Hop_BP	Two-Hop_BY	Two-Hop_BG	Max_Membership	Sum
SOK2	YGR083C	1.09241		2.996						4.08841
SOK2	YHL024W	0.442447		3.94041						4.38286
SOK2	YHR050W	0.92885		3.55187						4.48072
SOK2	YHR077C			4.35129						4.35129
SOK2	YHR089C			5.09451						5.09451
SOK2	YHR166C			4.62657						4.62657
SOK2	YHR168W			4.51167						4.51167
SOK2	YIL003W			4.85811						4.85811
SOK2	YIL110W			5.24423						5.24423
SOK2	YIR019C	2.75067		1.77273						4.5234
SOK2	YJL063C			4.45541						4.45541
SOK2	YJL209W			6.06108						6.06108
SOK2	YJR094C	3.02335		1.64278						4.66613
SOK2	YKL062W	2.47489		2.37359						4.84848
SOK2	YLL058W			5.06322						5.06322
SOK2	YLR338W			4.28206						4.28206
SOK2	YMR013C			5.53202						5.53202
SOK2	YMR017W	2.8557		5.86528						8.72098
SOK2	YMR112C			4.93122						4.93122
SOK2	YMR199W	1.25603		2.94624						4.20227
SOK2	YMR260C			4.18747						4.18747
SOK2	YMR305C	1.58816		1.72887	0.786216					4.10325
SOK2	YNR028W	2.5847		2.04814						4.63284
SOK2	YOL032W			5.44032						5.44032
SOK2	YOL132W			4.52158						4.52158
SOK2	YOR171C	0.480827		5.42243						5.90326
SOK2	YOR313C	1.77077		5.01011						6.78088
SOK2	YOR374W			4.98032						4.98032
SOK2	YPR098C			4.62912						4.62912
SOK2	YPR125W			5.21444						5.21444
SOK2	YPR180W			4.17706						4.17706
SPT2	YOR234C	4.4396								4.4396
SPT2	YOR236W	4.4396								4.4396
SPT2	YOR237W	4.4396								4.4396
SPT23	SNR37	4.85851								4.85851
SPT23	TK(CUU)C	6.77911								6.77911
SPT23	TR(UCU)E	7.06242								7.06242
SPT23	YCR019W	6.77911		1.5189						8.29801
SPT23	YCR020C	5.56124								5.56124
SPT23	YCR020C-A	5.56124								5.56124
SPT23	YCR076C			4.2326						4.2326
SPT23	YCR096C	3.4269		1.04455		0.760391				5.23184
SPT23	YCR097W	3.4269				0.760391				4.18729
SPT23	YDR063W			4.10286						4.10286
SPT23	YDR084C	4.1101								4.1101
SPT23	YGL001C	4.78069		0.168268	0.383252					5.33221
SPT23	YHR019C			3.78629	0.871164					4.65745
SPT23	YHR142W			4.44392						4.44392
SPT23	YHR192W			4.9006						4.9006
SPT23	YJR010C-A	4.69699								4.69699
SPT23	YJR011C	4.69699		1.85823						6.55522
SPT23	YJR013W	4.69699		1.97213						6.66912
SPT23	YKL073W			4.56541	0.341453					4.90686
SPT23	YKL081W			3.39269	1.05602					4.44871
SPT23	YLR047C	5.61777								5.61777
SPT23	YLR048W	5.61777			0.641904					6.25967
SPT23	YMR193W	4.88427		0.916845			0.827075			6.62819
SPT23	YMR194W	4.88427			0.784874					5.66914
SPT23	YMR258C	6.31868								6.31868
SPT23	YMR271C	0.309518		5.65139			0.878051			6.83896
SPT23	YNL084C			5.18625						5.18625
SPT23	YNL175C			5.70839	0.587956					6.29635
SPT23	YNL178W			2.57159	1.26279		0.9141			4.74848
SPT23	YNL237W			5.39332						5.39332
SPT23	YOR001W	6.03375			0.67207					6.70582
SPT23	YOR243C			1.79514	1.45655		0.894896			4.14659
SPT23	YPL060W			4.24216						4.24216
STB1	SNR40	5.75532								5.75532
STB1	YAL037W		0.267639	3.73649						4.00413
STB1	YBR088C	0.264051	0.757271	9.22046	0.428683				0.726537	11.397
STB1	YBR162C		0.518051	5.12523					0.603648	6.24693
STB1	YBR162W-A		0.518051	5.27084					0.518151	6.30704
STB1	YBR163W		0.518051	5.33504						5.85309
STB1	YBR202W			4.39925						4.39925
STB1	YCR065W	2.76358	0.598102	7.6288	0.340752				0.560804	11.892
STB1	YDL003W		0.757271	9.18184	0.441308				0.725901	11.1063
STB1	YDL055C		0.598102	9.07397	0.925851				0.640668	11.2386
STB1	YDL192W		0.518051	5.08515						5.6032
STB1	YDL219W		0.0603717	10.768						10.8284
STB1	YDL220C		0.0603717	7.21489						7.27526
STB1	YDR222W		0.757271	10.4279						11.1852
STB1	YDR288W			4.38165						4.38165
STB1	YDR306C		0.757271	6.72171						7.47898
STB1	YDR318W			5.51273						5.51273
STB1	YDR500C	4.40167	0.757271		0.469397				0.724669	6.35301
STB1	YDR501W	4.40167	0.757271	11.8956						17.0545
STB1	YDR525W-A	2.13351	0.0630603	2.06519						4.26176
STB1	YDR527W	2.13351	0.0630603	2.04447					0.601383	4.84242
STB1	YEL040W	1.50179	0.0529715	2.94925					0.729779	5.23379
STB1	YEL061C			8.4487					0.712379	9.16108
STB1	YER104W		0.598102	8.21634						8.81444
STB1	YGR152C		0.757271	2.65288				0.943118	0.661664	5.01493
STB1	YGR153W		0.757271	4.32208						5.07935
STB1	YGR221C	2.45823	0.598102	9.5676	0.46229				0.681171	13.7674
STB1	YGR222W	2.45823	0.598102	7.32402						10.3804
STB1	YHR061C	3.13606		0.986761						4.12282
STB1	YIL123W		0.277353	3.81141					0.679809	4.76857
STB1	YIL132C		0.757271	6.20302					0.660086	7.62038
STB1	YIL140W	1.10086	0.720889	7.4437	0.868912				0.721283	10.8556
STB1	YJL018W			4.13538						4.13538
STB1	YJL019W		0.720889	4.04819						4.76908
STB1	YJL020C		0.720889	5.20208						5.92297
STB1	YJL186W		0.480094	6.38775						6.86784
STB1	YJL187C		0.480094	6.88999						7.37008
STB1	YJR054W		0.720889	10.5813					0.541304	11.8435
STB1	YKL007W		0.480094	4.66035						5.14044
STB1	YKL096W-A	3.52016	0.0529715	1.49584						5.06897
STB1	YKL101W	0.215646	0.518051	3.95075					0.590437	5.27488
STB1	YKL103C	0.215646	0.518051	5.21917	0.49982					6.45269
STB1	YKL127W		0.757271	11.6591					0.449042	12.8654

TF	Target	Binding_0-2000	Motif_Score_SC	Other_Yeasts	Two-Hop_BE	Two-Hop_BP	Two-Hop_BY	Two-Hop_BG	Max_Membership	Sum
STB1	YKL165C		0.757271	6.58855					0.574959	7.92078
STB1	YLL021W		0.757271	10.5745					0.609788	11.9416
STB1	YLL022C		0.757271	11.6348						12.3921
STB1	YLR049C		0.56068	6.54054						7.10122
STB1	YLR183C		0.757271	8.30495					0.614766	9.67699
STB1	YLR233C		0.598102	5.64027						6.23837
STB1	YLR332W		0.757271	8.45226	0.427063					9.63659
STB1	YLR342W	2.72081	0.757271						0.725314	4.2034
STB1	YLR372W		0.757271	9.38004					0.720077	10.8574
STB1	YML058W		0.277353	5.11383						5.39118
STB1	YML058W-A		0.277353	6.99066						7.26801
STB1	YML059C		0.277353	4.90233					0.500288	5.67997
STB1	YNL065W			4.67719						4.67719
STB1	YNL082W		0.598102	4.87888						5.47698
STB1	YNL272C			4.35173						4.35173
STB1	YNL282W		0.757271	10.145						10.9023
STB1	YNL283C		0.757271	2.9834					0.618067	4.35874
STB1	YNL289W	5.75532		1.26893						7.02425
STB1	YNL291C	5.75532								5.75532
STB1	YNL300W	1.12043	0.237745	2.45054	0.35146				0.721879	4.88205
STB1	YOL007C	3.86495	0.757271	6.28654	0.765121					11.6739
STB1	YOL090W	0.389149	0.757271	6.06952					0.551439	7.76738
STB1	YOL097C			5.27718					0.667118	5.9443
STB1	YOR093C			5.435						5.435
STB1	YOR114W		0.757271	12.615						13.3723
STB1	YOR138C	4.49831								4.49831
STB1	YOR140W	4.49831								4.49831
STB1	YOR246C	4.26327	0.720889	3.05463						8.03879
STB1	YOR247W	4.26327	0.720889	3.60121						8.58537
STB1	YOR342C	0.0947368	0.757271	4.7165						5.56851
STB1	YOR372C	4.00334	0.277353	2.68497						6.96566
STB1	YOR373W	4.00334	0.277353	2.8453						7.12599
STB1	YPL057C	2.70693		0.868652					0.486015	4.0616
STB1	YPL124W		0.757271	7.9121		0.892547				9.56192
STB1	YPL194W			4.52501						4.52501
STB1	YPL256C	3.91645			0.754602				0.676277	5.34733
STB2	YJR088C	5.22441								5.22441
STB2	YJR089W	5.22441								5.22441
STB2	YLR375W	3.99044			0.745846					4.73629
STB4	YBL015W			3.75166	1.70499		1.47039			6.92704
STB4	YBL027W				2.59981		1.7393			4.33911
STB4	YBL072C				2.94335		1.67074			4.61409
STB4	YBL087C				2.9542		1.52862			4.48282
STB4	YBL092W				2.8663		1.74637			4.61267
STB4	YBR045C	12.7941	0.748942							13.543
STB4	YBR046C	12.7941	0.748942				1.15487			14.6979
STB4	YBR047W	12.7941	0.748942							13.543
STB4	YBR048W	12.7941	0.748942							13.543
STB4	YBR084C-A				2.5735		1.7393			4.3128
STB4	YBR181C				2.7122		1.7178			4.43
STB4	YBR189W				2.87814		1.75895			4.63709
STB4	YBR191W			0.0219322	2.94318		1.58089			4.546
STB4	YBR222C	13.2403	0.841657	10.5966						24.6786
STB4	YCL043C		0.795194	3.32743	0.968195					5.09082
STB4	YCR031C				2.44793		1.76197			4.2099
STB4	YCR083W			6.71017						6.71017
STB4	YDL061C				2.4806		1.61324			4.09384
STB4	YDL074C	1.43618					0.940935	1.63236		4.00948
STB4	YDL083C				2.86236		1.74565			4.60801
STB4	YDL191W	0.264817			2.3353		1.56492			4.16504
STB4	YDL225W			4.03422						4.03422
STB4	YDR012W				2.58675		1.7178			4.30455
STB4	YDR045C		0.795194	3.17055			1.75892			5.72466
STB4	YDR064W				2.84301		1.74637			4.58938
STB4	YDR125C	0.126478		4.35271						4.47919
STB4	YDR233C			4.48666						4.48666
STB4	YDR321W	0.694022			2.6433		1.54378			4.8811
STB4	YDR418W				2.97183		1.73477			4.7066
STB4	YDR447C				2.50015		1.7178			4.21795
STB4	YDR450W				2.86359		1.72329			4.58688
STB4	YDR500C				2.7608		1.62417			4.38497
STB4	YDR536W				2.36384		1.75724			4.12108
STB4	YEL015W		0.795194	1.31917		2.70184				4.8162
STB4	YEL018W	4.03905	0.841657							4.88071
STB4	YEL020C	4.03905	0.841657	11.0851						15.9658
STB4	YEL026W		0.748942		2.19637		1.74637			4.69168
STB4	YEL054C				2.86051		1.73477			4.59528
STB4	YER007C-A	4.27937			1.61404		1.7178			7.61121
STB4	YER065C				1.52796		1.57828	2.17437		5.28061
STB4	YER074W				2.91696		1.56072			4.47768
STB4	YER080W		0.841657	5.20526						6.04692
STB4	YER102W				2.94181		1.67074			4.61255
STB4	YER117W				2.9142		1.52862			4.44282
STB4	YFR023W		0.748942	3.74599						4.49493
STB4	YGL001C		0.795194	6.51512			1.6997			9.01001
STB4	YGL030W				2.84856		1.59317			4.44173
STB4	YGL076C				2.96695		1.73105			4.698
STB4	YGL103W			0.456463	2.03556		1.57213			4.06415
STB4	YGL135W				2.87526		1.74753			4.62279
STB4	YGL147C				2.9093		1.75687			4.66617
STB4	YGL184C	0.285398	0.841657	6.90619						8.03324
STB4	YGL196W		0.841657	5.60407						6.44573
STB4	YGR034W				2.86959		1.75234			4.62193
STB4	YGR085C				2.74971		1.68101			4.43072
STB4	YGR094W	2.45649			1.85068					4.30717
STB4	YGR118W				2.6644		1.76362			4.42802
STB4	YGR175C			4.27552			1.79686			6.07238
STB4	YGR214W				2.9523		1.76334			4.71564
STB4	YGR289C	1.00444		3.01663						4.02107
STB4	YHL015W				2.6244		1.76035			4.38475
STB4	YHL033C		0.795194		2.7959		1.75746			5.34855
STB4	YHR021C			0.0194507	2.47722		1.7178			4.21447
STB4	YHR068W	1.84245			1.71968		1.7023			5.26443
STB4	YHR141C				2.55083		1.74637			4.2972
STB4	YHR203C				2.90284		1.7178			4.62064
STB4	YIL133C		0.841657	2.2072	2.89519		1.74759			7.69164
STB4	YIL166C	13.7294	0.795194	8.13954						22.6641
STB4	YJL030W		0.841657	3.22716						4.06882
STB4	YJL158C		0.841657	4.79852						5.64018
STB4	YJL177W		0.748942		2.94714		1.73091			5.42699

TF	Target	Binding_0-2000	Motif_Score_SC	Other_Yeasts	Two-Hop_BE	Two-Hop_BP	Two-Hop_BY	Two-Hop_BG	Max_Membership	Sum
STB4	YJL190C				2.7481		1.76369			4.51179
STB4	YJL191W				2.30243		1.76197			4.0644
STB4	YJL210W		0.748942	6.17803						6.92697
STB4	YJL212C		0.748942	4.56066						5.3096
STB4	YJR043C		0.748942	2.20462			1.64476			4.59832
STB4	YJR078W		0.795194	5.91801						6.7132
STB4	YJR094W-A				2.6613		1.7178			4.3791
STB4	YJR116W			4.09439						4.09439
STB4	YJR123W				2.93483		1.75833			4.69316
STB4	YJR145C		0.795194	0.842849	2.60075		1.7178			5.95659
STB4	YKL096W		0.841657	7.52421						8.36587
STB4	YKL129C			3.83343				1.63236		5.46579
STB4	YKL180W				2.58546		1.73284			4.3183
STB4	YKL182W						1.74323	2.52257		4.2658
STB4	YKL188C			1.71801	1.57168		1.53601			4.8257
STB4	YKR006C			5.82877						5.82877
STB4	YKR008W			4.04829						4.04829
STB4	YLL045C				2.67292		1.75746			4.43038
STB4	YLL055W	12.3206								12.3206
STB4	YLL056C	12.3206								12.3206
STB4	YLL058W	8.31251	0.841657	2.96155			1.68104			13.7968
STB4	YLL060C	8.31251	0.841657							9.15417
STB4	YLR029C				2.39976		1.74637			4.14613
STB4	YLR048W				2.95677		1.75512			4.71189
STB4	YLR075W				2.46478		1.64397			4.10875
STB4	YLR133W			5.99361						5.99361
STB4	YLR141W		0.795194	4.20906						5.00425
STB4	YLR185W				2.80291		1.62417			4.42708
STB4	YLR264W				2.60999		1.61324			4.22323
STB4	YLR274W	1.26014			1.70081		1.75763			4.71858
STB4	YLR297W		0.795194	3.58919						4.38438
STB4	YLR330W	1.24461	0.795194	3.2448						5.2846
STB4	YLR340W				2.32341		1.7135			4.03691
STB4	YLR344W				2.85667		1.75234			4.60901
STB4	YLR367W				2.6517		1.76369			4.41539
STB4	YLR388W				2.93266		1.61324			4.5459
STB4	YLR441C		0.841657		2.87305		1.76083			5.47554
STB4	YML024W			0.321102	2.55213		1.7178			4.59103
STB4	YML026C				2.80404		1.72329			4.52733
STB4	YML057W		0.841657	6.70932						7.55098
STB4	YML063W				2.95372		1.76083			4.71455
STB4	YML088W			4.53291						4.53291
STB4	YML106W		0.748942	2.20144	2.18012		1.52286			6.65336
STB4	YMR031C	5.95608								5.95608
STB4	YMR032W	5.95608								5.95608
STB4	YMR121C				2.56061		1.74637			4.30698
STB4	YMR143W				2.87086		1.74565			4.61651
STB4	YMR226C		0.795194	5.78848						6.58367
STB4	YMR228W			4.7471						4.7471
STB4	YMR283C			4.56347						4.56347
STB4	YMR286W	0.0697872	0.795194	8.94379						9.80877
STB4	YNL027W			5.37436						5.37436
STB4	YNL067W				2.40581		1.7532			4.15901
STB4	YNL069C				2.95358		1.76166			4.71524
STB4	YNL117W	11.3244	0.841657	9.87927						22.0453
STB4	YNL118C	11.3244	0.841657	5.10677						17.2728
STB4	YNL162W				2.55141		1.74637			4.29778
STB4	YNL178W				2.88592		1.76204			4.64796
STB4	YNL305C		0.841657	1.94012			1.53564			4.31742
STB4	YOL039W				2.68735		1.56072			4.24807
STB4	YOL040C				2.89984		1.76275			4.66259
STB4	YOL121C			0.519764	2.68966		1.7178			4.92722
STB4	YOL127W				2.77857		1.73206			4.51063
STB4	YOR115C	1.376		6.64505						8.02105
STB4	YOR145C			0.684426	1.85063		1.7178			4.25286
STB4	YOR167C				2.89499		1.61324			4.50823
STB4	YOR178C		0.795194			3.2891	1.53191			5.6162
STB4	YOR369C				2.64774		1.66801			4.31575
STB4	YPL028W			1.7532	1.89488		1.52252			5.1706
STB4	YPL064C			5.04283						5.04283
STB4	YPL079W				2.81895		1.58089			4.39984
STB4	YPL090C				2.79684		1.7178			4.51464
STB4	YPL160W				2.45888		1.75081			4.20969
STB4	YPL198W				2.94176		1.73105			4.67281
STB4	YPL220W				2.92359		1.74753			4.67112
STB4	YPR006C		0.841657				1.58286	2.17617		4.60069
STB4	YPR043W	0.0654177			2.91869		1.7178			4.70191
STB4	YPR102C				2.88316		1.68101			4.56417
STB4	YPR110C				2.48128		1.72218			4.20346
STB4	YPR128C	4.3798	0.795194	3.7066						8.88159
STB4	YPR129W	4.3798	0.795194	4.89852						10.0735
STB4	YPR132W				2.75922		1.76362			4.52284
STB5	YBR119W		0.405309	3.63889						4.0442
STB5	YCR016W		0.0831841	5.01118	0.36675					5.46111
STB5	YDL049C	3.89728	0.545889	2.63763						7.0808
STB5	YDL197C			2.98889				1.28739		4.27628
STB5	YDL213C		0.531677	3.57835						4.11003
STB5	YDR221W	3.56338		0.93091						4.49429
STB5	YDR231C	5.47573	0.133847	4.88275	2.49333					12.9857
STB5	YDR232W	5.47573	0.133847	3.88402	1.50409					10.9977
STB5	YDR248C	10.3477	1.24244	12.5164						24.1065
STB5	YDR262W	0.431787	0.246209	3.65692						4.33492
STB5	YDR287W	0.913811			2.06562	3.07073				6.05016
STB5	YDR399W		0.0543177	4.80459						4.85891
STB5	YEL041W	15.8551	1.24244	11.6719						28.7694
STB5	YER075C		1.24244	7.77756						9.02
STB5	YER142C		1.24244	5.08392	0.517588					6.84395
STB5	YER177W		0.701709	4.1543						4.85601
STB5	YFL014W			3.39153					0.719124	4.11065
STB5	YFR049W			4.96316						4.96316
STB5	YGL037C		0.701709	4.3354	2.35016					7.38727
STB5	YGL216W			0.301529		2.75445		1.02274		4.07872
STB5	YGL220W		1.24244	5.59919						6.84163
STB5	YGL221C		1.24244	7.95793						9.20037
STB5	YGL254W		0.701709	3.5343						4.23601
STB5	YGR112W		0.643171	5.21214	1.89123					7.74654
STB5	YGR199W			6.97498						6.97498
STB5	YHR156C	5.60238								5.60238
STB5	YHR157W	5.60238								5.60238
STB5	YHR161C	15.2955	1.24244		0.487419					17.0254

TF	Target	Binding_0-2000	Motif_Score_SC	Other_Yeasts	Two-Hop_BE	Two-Hop_BP	Two-Hop_BY	Two-Hop_BG	Max_Membership	Sum
STB5	YHR163W	15.2955	1.24244	9.21466						25.7526
STB5	YHR178W	2.00391		2.66211						4.66602
STB5	YHR183W	13.7085	0.386934	0.624774						14.7202
STB5	YHR196W		0.643171	0.568521	0.346946	2.75445				4.31309
STB5	YIL160C	2.68075		1.00298	1.52743					5.21116
STB5	YIR006C					3.96438		0.968773		4.93315
STB5	YJL109C			4.19172	0.361088					4.55281
STB5	YJR088C	3.46661		1.25626						4.72287
STB5	YJR096W		0.287051	1.99478	1.85378		1.32181			5.45742
STB5	YKL134C			4.92847						4.92847
STB5	YKL179C	4.71691		0.0630329						4.77994
STB5	YKR058W				2.50557		1.93078			4.43635
STB5	YLR042C			3.54374	2.24012					5.78386
STB5	YLR152C	12.8934	1.24244	7.77004					0.567334	22.4732
STB5	YLR174W	0.729646	1.08753	2.66245						4.47963
STB5	YLR277C		0.531677	3.82355						4.35523
STB5	YLR354C	5.20902	0.274117	3.59371						9.07685
STB5	YMR059W			1.25212		2.75445				4.00657
STB5	YMR097C		0.747751	3.02343	1.41223					5.18341
STB5	YMR158W	1.21089			1.79385		1.22634			4.23108
STB5	YMR180C			4.72503		0.801685				5.52672
STB5	YMR272C	4.20413								4.20413
STB5	YMR315W	3.7098	0.226004	7.74908	1.51588				0.719353	13.9201
STB5	YNL273W	11.1341		0.387577						11.5217
STB5	YNL274C	11.1341		0.513164	1.08279				0.690685	13.4207
STB5	YNR037C			9.68572						9.68572
STB5	YOL151W			2.39113	1.64057				0.725065	4.75676
STB5	YOR007C			3.56689	0.625734					4.19262
STB5	YOR086C	3.1543	0.226004	0.655441						4.03575
STB5	YOR087W	3.1543	0.226004	0.653594						4.0339
STB5	YOR092W			4.16574						4.16574
STB5	YOR115C			4.26369						4.26369
STB5	YOR119C	0.666367	1.24244	10.0104						11.9192
STB5	YOR120W	0.666367	1.24244	10.867	1.79262				0.679223	15.2477
STB5	YPL061W	3.57667	0.133847	7.19101						10.9015
STB5	YPR074C	10.6843		0.0488645	0.440105					11.1733
STB5	YPR165W			5.48244						5.48244
STB5	YPR179C		0.911513	5.62723						6.53874
STB5	YPR180W		0.911513	5.56856						6.48007
STE12	SNR40	15.8551								15.8551
STE12	TA(AGC)H	6.64036								6.64036
STE12	TA(AGC)K2	5.01764								5.01764
STE12	TD(GUC)J1	5.22759								5.22759
STE12	TD(GUC)L2	5.55613								5.55613
STE12	TF(GAA)D	9.61353								9.61353
STE12	TG(GCC)B	15.8551								15.8551
STE12	TG(GCC)G1	4.72194								4.72194
STE12	TH(GUG)E2	4.71443								4.71443
STE12	TI(AAU)P2	5.2102								5.2102
STE12	TK(CUU)E1	12.45								12.45
STE12	TK(UUU)G2	7.51691								7.51691
STE12	TK(UUU)P	4.03489								4.03489
STE12	TM(CAU)D	3.84682	0.205071							4.05189
STE12	TN(GUU)O2	5.77294								5.77294
STE12	TP(UGG)N1	5.78809								5.78809
STE12	TQ(UUG)D2	5.68355								5.68355
STE12	TR(ACG)L	4.14481								4.14481
STE12	TR(CCU)J	5.43457								5.43457
STE12	TT(AGU)N1	5.06723	0.205071							5.2723
STE12	TV(AAC)K2	4.50681								4.50681
STE12	TW(CCA)G1	5.19886								5.19886
STE12	TX(XXX)D	11.5464								11.5464
STE12	TY(GUA)D	10.2115								10.2115
STE12	YAL003W		0.205071	0.0330061	1.87058		2.00393		0.731639	4.84423
STE12	YAL021C					0.496309	0.892214	2.65421		4.04273
STE12	YAL024C			2.14365			1.86465			4.0083
STE12	YAL029C	2.40177				0.901149	1.87498	3.4051	0.697086	9.28009
STE12	YAL039C	4.18027								4.18027
STE12	YAL059W	0.594312		1.88708	1.10236				0.658745	4.2425
STE12	YAR014C	12.345		0.589792		2.27958				15.2144
STE12	YAR015W	12.345		0.729798						13.0748
STE12	YAR020C	4.14802		1.45255						5.60057
STE12	YAR061W				1.39322			3.41613		4.80935
STE12	YBL001C			4.28026	0.62146					4.90172
STE12	YBL004W	4.35062								4.35062
STE12	YBL016W	2.98596		0.652709	2.50548	4.11544	1.98861	0.859094		13.1073
STE12	YBL017C	2.98596				1.51574				4.5017
STE12	YBL049W	1.06093	0.205071		0.611551	2.90244				4.77999
STE12	YBL060W	6.03157								6.03157
STE12	YBL061C	6.03157				0.420309				6.45188
STE12	YBL087C				2.18924	2.08726			0.713488	4.98999
STE12	YBL105C				3.5944	0.466308		0.859092		4.9198
STE12	YBL112C				1.69656			3.40826		5.10482
STE12	YBR009C				3.56331	3.33393	1.51549		0.636584	9.04931
STE12	YBR011C	9.45428			3.59808		0.921742			13.9741
STE12	YBR023C	2.16712	0.205071			1.46353			0.640169	4.47589
STE12	YBR040W	5.9159	0.205071	2.90863	3.13682				0.440728	12.6071
STE12	YBR041W	5.9159	0.205071					1.07292		7.19389
STE12	YBR052C			4.65561	2.60879					7.2644
STE12	YBR060C	2.63333		2.77781		1.49222	1.99707			8.90043
STE12	YBR066C	2.8032			1.48438					4.28758
STE12	YBR078W	1.63539			1.91356				0.652121	4.20107
STE12	YBR081C	1.90138				1.06368	1.99707	1.37597		6.3381
STE12	YBR082C	15.8551								15.8551
STE12	YBR083W	15.8551		0.0811535	0.416338	3.33393				19.6865
STE12	YBR084W	2.38509			1.08515		1.0696		0.577039	5.11688
STE12	YBR088C				1.54554	1.36958	1.23106		0.726537	4.87272
STE12	YBR109C				2.94104	2.38752	2.04834			7.3769
STE12	YBR138C		0.205071	5.73938	1.35439					7.29884
STE12	YBR150C	0.468346		4.81594						5.28429
STE12	YBR154C			0.217655	2.30735		1.25291		0.606218	4.38413
STE12	YBR155W				1.8593	1.59189	2.0201			5.47129
STE12	YBR156C	4.51989		0.428008	0.416292				0.71837	6.08256
STE12	YBR158W	4.8358			2.66731				0.717246	8.22036
STE12	YBR160W			0.782341		3.17687	1.76244			5.72165
STE12	YBR162C	1.87684		0.0286172	1.46469			2.49209	0.603648	6.46589
STE12	YBR162W-A	1.87684			1.76451				0.518151	4.1595
STE12	YBR189W				2.15407		1.24005		0.661198	4.05532
STE12	YBR191W				2.16871		1.40065		0.711752	4.28111
STE12	YBR200W	2.36808				1.43298			0.453794	4.25485

TF	Target	Binding_0-2000	Motif_Score_SC	Other_Yeasts	Two-Hop_BE	Two-Hop_BP	Two-Hop_BY	Two-Hop_BG	Max_Membership	Sum
STE12	YBR212W			0.908182	1.56028		0.950773	1.26961		4.68884
STE12	YBR217W				2.56548	1.60601				4.17149
STE12	YBR267W				1.74222		2.00261		0.720385	4.46521
STE12	YBR274W					1.49629	1.81192	0.85909		4.1673
STE12	YBR286W		0.205071	3.47275			0.865157			4.54298
STE12	YBR288C				2.46637		1.88582			4.35219
STE12	YBR291C	0.853761			2.36572		1.94048			5.15996
STE12	YCL027W	15.8551			2.8761	1.09168			0.725414	20.5483
STE12	YCL029C	15.8551					0.950129	0.985586		17.7908
STE12	YCL039W					2.90244	1.99707			4.89951
STE12	YCL043C				2.76788		1.08945		0.577297	4.43463
STE12	YCL055W	15.8551			2.49835				0.729409	19.0829
STE12	YCL056C	15.8551								15.8551
STE12	YCL059C				3.58005	0.819222	1.22417		0.713339	6.33678
STE12	YCL066W			0.181561	0.733464	4.11544				5.03047
STE12	YCR023C	3.81055		0.526196			1.63083			5.96758
STE12	YCR065W	0.756034			3.54895				0.560804	4.86579
STE12	YCR067C				3.59181		1.82696	3.02833		8.4471
STE12	YCR089W	15.8551			0.414261			1.63503		17.9044
STE12	YDL010W			0.307051	2.67931		2.05602		0.709014	5.7514
STE12	YDL038C						1.80002	3.13438	0.726845	5.66125
STE12	YDL047W					2.27958	2.00866		0.727476	5.01572
STE12	YDL055C	0.130925	0.205071	0.483284	3.59239				0.692944	5.10461
STE12	YDL059C		0.205071	5.5625		1.6886				7.45617
STE12	YDL070W				3.03235		1.69093	1.37522		6.0985
STE12	YDL081C			2.26811	1.43528				0.719124	4.42251
STE12	YDL101C			0.0430238	0.737683	2.66881		0.860776	0.656947	4.96724
STE12	YDL127W	15.8551								15.8551
STE12	YDL128W	5.20236								5.20236
STE12	YDL135C	5.11336				0.466308				5.57967
STE12	YDL137W				2.32231		2.02155			4.34386
STE12	YDL145C				0.420544	1.03632	1.2828	1.20167	0.637573	4.57891
STE12	YDL153C				1.83838	3.44746	2.00261		0.709375	7.99782
STE12	YDL154W				2.60879		1.60679			4.21558
STE12	YDL180W	1.20978				4.11544				5.32522
STE12	YDL186W					4.11544				4.11544
STE12	YDL192W				2.23553		2.02155			4.25708
STE12	YDL197C		0.205071		0.892888		1.96739	1.24271	0.610551	4.91861
STE12	YDR075W			0.189689	0.589135	3.33393	2.04554			6.15829
STE12	YDR085C	9.69699		0.938795	1.60503					12.2408
STE12	YDR097C	5.68355					1.60679		0.687202	7.97754
STE12	YDR098C	5.68355								5.68355
STE12	YDR113C			3.88913	0.659648	2.32362				6.8724
STE12	YDR144C	2.17172			1.58791		2.05602		0.716231	6.53188
STE12	YDR146C	3.19144			1.23632	3.67116	2.06905	2.02793	0.693765	12.8897
STE12	YDR147W	3.19144					1.2319			4.42334
STE12	YDR169C	5.05677								5.05677
STE12	YDR171W	2.0776		0.206847	1.38404	0.690189				4.35868
STE12	YDR190C			2.94369	1.64254		2.0712		0.722251	7.37968
STE12	YDR212W				0.826683	2.66881			0.692753	4.18825
STE12	YDR224C	0.145871			0.448432	3.33393			0.667385	4.59562
STE12	YDR235W				1.12574	3.419				4.54474
STE12	YDR240C	10.2115			1.7409	3.419				15.3714
STE12	YDR242W	10.2115		0.0569602	1.37602					11.6445
STE12	YDR251W	3.37709	0.205071		2.95785		0.777169			7.31718
STE12	YDR253C		0.205071	8.27919			2.00261			10.4869
STE12	YDR272W			2.5957	2.00235		0.8877			5.48575
STE12	YDR281C	4.66682			0.343309					5.01013
STE12	YDR295C			3.69168			2.08792	3.41613		9.19573
STE12	YDR309C	5.67279	0.205071		2.15688					8.03474
STE12	YDR310C	2.20851		2.42609			1.99648			6.63108
STE12	YDR311W	2.20851		2.2072		1.38415				5.79986
STE12	YDR312W	4.19776			1.5105					5.70826
STE12	YDR319C				1.17213	4.11544				5.28757
STE12	YDR323C						2.03748	3.28268		5.32016
STE12	YDR328C				2.27734		1.88582			4.16316
STE12	YDR339C	11.5464			1.0791				0.565471	13.191
STE12	YDR349C	3.84682	0.205071							4.05189
STE12	YDR364C					3.25978	2.01033			5.27011
STE12	YDR371W			2.48347	2.57439					5.05786
STE12	YDR373W	1.67415			0.441164		2.03748	1.61207		5.76486
STE12	YDR379W	3.20302		0.819958						4.02298
STE12	YDR388W					4.11544				4.11544
STE12	YDR400W		0.205071	3.70785					0.671654	4.58458
STE12	YDR408C			3.93147			1.56636			5.49783
STE12	YDR412W	0.944942			2.02612	0.480225			0.690162	4.14145
STE12	YDR429C			0.223418	2.50152		0.942125		0.629362	4.29643
STE12	YDR447C		0.205071	0.210551	1.71401		1.22081		0.723017	4.07346
STE12	YDR448W					1.25421	2.00303	0.937177		4.19442
STE12	YDR450W				2.11676		1.19105		0.718836	4.02665
STE12	YDR459C	8.15294	0.205071							8.35801
STE12	YDR461W	8.15294	0.205071							8.35801
STE12	YDR462W	8.15294	0.205071							8.35801
STE12	YDR463W	8.15294	0.205071					1.1339		9.49191
STE12	YDR480W			0.296061		4.11544				4.4115
STE12	YDR496C		0.205071		2.24934		1.38954		0.682062	4.52601
STE12	YDR528W	0.774522		4.33409						5.10861
STE12	YDR529C	2.44891				3.33393				5.78284
STE12	YDR545W						1.70069	3.04517		4.74586
STE12	YEL020C			2.58689	1.57125		1.62041			5.77855
STE12	YEL026W	0.350611	0.205071	4.19993	1.46498		1.28734		0.727573	8.2355
STE12	YEL032W				2.96114		1.22607		0.681423	4.86863
STE12	YEL034W				2.87038		1.2041		0.456537	4.53102
STE12	YEL040W	5.88909			0.86435				0.729779	7.48322
STE12	YER016W					3.7382			0.472508	4.21071
STE12	YER025W				1.01022	2.66881			0.710126	4.38916
STE12	YER044C-A	2.56513			2.86505					5.43018
STE12	YER048W-A	5.82707								5.82707
STE12	YER049W	5.82707							0.71935	6.54642
STE12	YER064C	4.2497			0.49395					4.74365
STE12	YER066W		0.205071	1.15683			1.29599	1.74585		4.40374
STE12	YER094C	3.13279			0.475356		0.964671			4.57282
STE12	YER095W	3.13279			0.998282	2.08726	1.29359		0.45128	7.9632
STE12	YER102W			1.90419	1.58654		1.1989		0.713993	5.40362
STE12	YER107C		0.205071	2.43026			0.825477		0.651438	4.11225
STE12	YER113C			4.89513					0.686583	5.58171
STE12	YER118C	5.56921				4.11544			0.723639	10.4083
STE12	YER133W					2.72357	2.03499			4.75856
STE12	YER136W	2.7295			2.19107					4.92057
STE12	YER148W				3.595	0.42155				4.01655

TF	Target	Binding_0-2000	Motif_Score_SC	Other_Yeasts	Two-Hop_BE	Two-Hop_BP	Two-Hop_BY	Two-Hop_BG	Max_Membership	Sum
STE12	YER155C	9.76028	0.205071			2.97732			0.544443	13.4871
STE12	YER157W	9.76028	0.205071							9.96535
STE12	YER159C	0.552576			1.58544		2.05602			4.19404
STE12	YER190W						1.95523	3.0537		5.00893
STE12	YFL017W-A			3.54141		3.22846	1.26943			8.0393
STE12	YFL026W	15.8551	0.205071		2.59095					18.6511
STE12	YFL027C	15.8551	0.205071	1.73487						17.795
STE12	YFL037W	4.6541					0.802609		0.694369	6.15108
STE12	YFL038C			5.99824		2.47011				8.46835
STE12	YFL039C	4.6541			1.28209	4.11544				10.0516
STE12	YFL065C				1.63725			3.40485		5.0421
STE12	YFL066C				1.56779			3.39949		4.96728
STE12	YFR016C				1.39286		1.9984	1.17119		4.56245
STE12	YFR019W						1.26362	2.965		4.22862
STE12	YGL011C		0.205071	2.54676	2.45825	0.490464	1.26026			6.96081
STE12	YGL028C	4.85342			2.45799				0.710135	8.02154
STE12	YGL032C	15.8551		0.147651	2.68572				0.569655	19.2581
STE12	YGL050W	3.51822		0.169143				0.953657		4.64102
STE12	YGL061C	1.184		2.47309		1.40833				5.06542
STE12	YGL062W	5.73313								5.73313
STE12	YGL064C	5.73313								5.73313
STE12	YGL070C	1.46998		1.64211	1.03483		1.31216			5.45908
STE12	YGL075C						1.93741	3.28268		5.22009
STE12	YGL082W			4.08491						4.08491
STE12	YGL086W			1.68572		2.32362	0.956206			4.96555
STE12	YGL095C	0.947699				1.3252	1.86703			4.13993
STE12	YGL097W	5.19316	0.205071	0.670716			1.97063		0.732003	8.77158
STE12	YGL106W			0.272635			2.04834	1.88246		4.20344
STE12	YGL115W			4.15816						4.15816
STE12	YGL116W	9.21765		0.31918		0.924521	2.01033			12.4717
STE12	YGL117W	5.19886								5.19886
STE12	YGL120C				1.68064	3.25978			0.718311	5.65873
STE12	YGL137W				1.48885	0.626821	1.2828		0.667106	4.06558
STE12	YGL157W				2.94861		1.73422		0.686322	5.36915
STE12	YGL158W	2.9776					1.87443	0.860776		5.71281
STE12	YGL162W	4.53548								4.53548
STE12	YGL163C	4.53548				0.680996		1.86265		7.07913
STE12	YGL178W			0.997441		4.11544	2.04834			7.16122
STE12	YGL192W	0.777626		4.66695		3.7382				9.18278
STE12	YGL205W			0.130444	2.60786		1.63083			4.36913
STE12	YGL223C			0.0194507	2.5771	2.47011				5.06666
STE12	YGR006W		0.205071	4.05233			1.83244			6.08984
STE12	YGR013W	13.2752				2.59193				15.8671
STE12	YGR014W	15.8551							0.638611	16.4937
STE12	YGR032W	2.30114		0.0871662	0.65516	0.466308			0.717355	4.22713
STE12	YGR040W					4.11544	1.81192	0.859094		6.78645
STE12	YGR130C				1.30243		1.87687	2.89939		6.07869
STE12	YGR152C	1.06266			1.56522	0.474558	1.23842		0.661664	5.00252
STE12	YGR159C					1.60598	2.04834		0.673957	4.32828
STE12	YGR172C	4.72194				4.11544			0.716924	9.5543
STE12	YGR174W-A	4.72194								4.72194
STE12	YGR189C	7.67352		3.05282	1.91478				0.555406	13.1965
STE12	YGR209C			4.79408						4.79408
STE12	YGR210C			3.6926					0.54101	4.23361
STE12	YGR214W				3.52017		1.62694		0.726502	5.87361
STE12	YGR221C	3.54494			0.707742				0.681171	4.93385
STE12	YGR233C	4.97276								4.97276
STE12	YGR234W	4.97276		0.0117152	0.646365	0.490464				6.1213
STE12	YGR238C				0.408047		2.04936	1.24271	0.716349	4.41647
STE12	YGR285C			0.087376		2.97732	1.24181		0.687871	4.99438
STE12	YGR294W	2.54875		0.431665	2.62812					5.60853
STE12	YGR296W						1.70069	3.04517		4.74586
STE12	YHL001W				2.99339		1.10123		0.690646	4.78527
STE12	YHL002W			2.43242				1.88397		4.31639
STE12	YHL013C				1.77029		0.83041	3.1312	0.621892	6.35379
STE12	YHL031C					2.47011	1.89541			4.36552
STE12	YHR005C	15.8551			2.24708	4.11544	1.77566			23.9933
STE12	YHR023W				1.15847	1.09168	1.98122		0.73036	4.96173
STE12	YHR030C			0.304283	0.641462	1.71811	1.87443	0.859094		5.39738
STE12	YHR064C				0.639635	2.97732	1.06754		0.707454	5.39195
STE12	YHR070W			1.56685			1.26588	1.19307	0.501663	4.52746
STE12	YHR079C	1.09497			3.09492		0.903939	1.05625		6.15008
STE12	YHR082C	5.5488				0.738593		0.859086		7.14648
STE12	YHR083W	5.5488								5.5488
STE12	YHR084W	15.8551				3.33393			0.6083	19.7973
STE12	YHR086W	5.42675				3.419	0.918485	1.25393		11.0182
STE12	YHR089C		0.205071	4.67606	1.498	0.819222			0.692996	7.89135
STE12	YHR097C	0.686931		0.909078	2.74824					4.34425
STE12	YHR102W				1.86079	1.49886		0.859099		4.21875
STE12	YHR141C	9.00759			1.59771				0.681933	11.2872
STE12	YHR142W	9.00759		3.62641					0.578254	13.2123
STE12	YHR144C			2.99161	1.08965				0.439906	4.52117
STE12	YHR154W		0.205071	0.908191	0.64364		2.0088		0.708026	4.47373
STE12	YHR158C			1.41541		4.11544	2.04939	1.68026		9.2605
STE12	YHR170W				0.742825	2.66881			0.730903	4.14254
STE12	YHR184W		0.205071	4.71972						4.92479
STE12	YHR203C			2.89152	1.75286		1.21369		0.72605	6.58412
STE12	YHR205W	5.85282					1.98861	0.85909		8.70052
STE12	YIL015W	14.2567			3.11728		1.96333			19.3373
STE12	YIL017C	14.2567								14.2567
STE12	YIL036W	7.20346								7.20346
STE12	YIL037C	7.20346			2.48269					9.68615
STE12	YIL061C	0.476097		0.422567		3.419	0.940618			5.25828
STE12	YIL083C	5.7516								5.7516
STE12	YIL084C	5.7516								5.7516
STE12	YIL123W	11.301			1.76539				0.679809	13.7462
STE12	YIL136W		0.205071		1.13906		2.05332	0.984071		4.38152
STE12	YIL138C					1.09168	1.98978	1.26741	0.519134	4.868
STE12	YIL144W					3.67116	1.89071	3.25364		8.81551
STE12	YIL148W				2.02838		1.47427		0.702745	4.2054
STE12	YIL159W					4.11544	1.84345			5.95889
STE12	YIR012W		0.205071	5.10079			2.03084		0.729823	8.06652
STE12	YJL002C				1.59572		2.05182		0.593562	4.2411
STE12	YJL005W					0.987169	1.88769	2.78963		5.66449
STE12	YJL036W			0.196745			2.10292	2.99806		5.29772
STE12	YJL057C					4.11544			0.59453	4.70997
STE12	YJL076W	5.63288			0.397696				0.722416	6.75299
STE12	YJL078C	5.63288			0.72981		1.82144			8.18413
STE12	YJL112W	5.22759								5.22759
STE12	YJL138C				0.909594	2.66881		3.01037	0.638221	7.22699

TF	Target	Binding_0-2000	Motif_Score_SC	Other_Yeasts	Two-Hop_BE	Two-Hop_BP	Two-Hop_BY	Two-Hop_BG	Max_Membership	Sum
STE12	YJL145W	2.75794		1.08774	1.9004				0.637602	6.38368
STE12	YJL148W	1.25713			2.43964				0.7273	4.42407
STE12	YJL157C	5.91838			2.51273	1.64961				10.0807
STE12	YJL158C	1.11932	0.205071	1.73554	2.31556				0.705204	6.0807
STE12	YJL161W	3.1879			1.27117					4.45907
STE12	YJL170C	5.49822			2.8675					8.36572
STE12	YJL171C	5.49822		1.21531	1.2125					7.92603
STE12	YJL186W	7.34397		1.65659	0.567156					9.56772
STE12	YJL187C	7.34397		0.560743	1.60037		1.10771	1.0564		11.6692
STE12	YJL190C			0.286249	2.05781		1.28411		0.718044	4.34621
STE12	YJL194W	6.13784				0.546166	1.09595			7.77996
STE12	YJL196C	6.13784			1.06392					7.20176
STE12	YJL197W				1.22116		2.00261	1.12907		4.35284
STE12	YJR054W	3.54958		0.0587272	1.17259				0.63696	5.41786
STE12	YJR055W	5.43457								5.43457
STE12	YJR062C				2.65412	1.60601	1.88162			6.14175
STE12	YJR154W	5.62992								5.62992
STE12	YKL009W				2.30516		1.54693		0.728122	4.58021
STE12	YKL010C					3.33393	1.88582			5.21975
STE12	YKL021C		0.205071	0.0999981	1.86508		1.26066		0.695625	4.12643
STE12	YKL047W			3.42173	0.868314					4.29004
STE12	YKL067W		0.205071	0.900001	1.17864	2.66881			0.617231	5.56975
STE12	YKL081W				1.15068	2.08726	0.879435		0.716437	4.83381
STE12	YKL082C				1.57132		2.0201		0.71477	4.30619
STE12	YKL094W	13.3913								13.3913
STE12	YKL095W	13.3913								13.3913
STE12	YKL096C-B	10.5416								10.5416
STE12	YKL096W	10.5416			1.3086				0.64856	12.4988
STE12	YKL101W	0.878487	0.205071		0.58484	2.32362		0.85909	0.590437	5.44154
STE12	YKL103C	0.878487	0.205071		1.77747	3.33393				6.19496
STE12	YKL104C	9.04786		0.297558	1.13523	0.490464			0.700354	11.6715
STE12	YKL129C				3.15116		1.08945	3.40567		7.64628
STE12	YKL161C					1.49629	1.87443	0.860776		4.2315
STE12	YKL179C	2.67352					1.97091	3.36085		8.00528
STE12	YKL185W			2.22494	1.31093				0.576942	4.11281
STE12	YKL189W	7.55988	0.205071		3.00683					10.7718
STE12	YKL190W			2.44711			2.02573	2.76981		7.24265
STE12	YKL193C				3.59813	0.574399	1.14137			5.3139
STE12	YKL195W			0.0730709	0.438556	3.2218	1.83164			5.56507
STE12	YKL207W	9.46157								9.46157
STE12	YKL208W	9.46157								9.46157
STE12	YKL209C	9.46157		0.039594						9.50116
STE12	YKR011C	8.60665			2.94966				0.603888	12.1602
STE12	YKR013W	8.60665			2.60726			2.94117	0.698386	14.8535
STE12	YKR041W	5.01764		2.35675						7.37439
STE12	YKR042W	4.94391			1.42681				0.730131	7.10085
STE12	YKR048C					2.66881	1.58529			4.2541
STE12	YKR059W				0.924749			3.01037	0.726276	4.6614
STE12	YKR061W	2.31894			1.69185					4.01079
STE12	YKR076W	2.64462		1.09777	0.50517					4.24756
STE12	YKR094C				2.09368		1.47427		0.727734	4.29568
STE12	YLL024C					4.11544			0.560889	4.67633
STE12	YLL039C					4.11544	1.94048			6.05592
STE12	YLL045C				2.0491		1.28798		0.732701	4.06978
STE12	YLL067C		0.205071		0.508032		0.925763	3.06194		4.70081
STE12	YLR002C			2.50031	1.3827				0.725778	4.60879
STE12	YLR045C					2.92563	1.96332	1.24853		6.13748
STE12	YLR084C	8.97964	0.205071	0.054367	0.483417				0.681804	10.4043
STE12	YLR086W	8.97964	0.205071	0.776478				0.991891		10.9531
STE12	YLR114C	3.51483			0.830356					4.34519
STE12	YLR147C		0.205071	0.919856		3.419			0.624761	5.16869
STE12	YLR153C				3.25459		0.876503			4.13109
STE12	YLR167W			0.685156	1.63692		1.45418		0.727042	4.5033
STE12	YLR185W				3.59556		1.15446		0.718329	5.46835
STE12	YLR196W	0.514883			1.56249	0.901149	2.0088		0.731223	5.71854
STE12	YLR197W				2.32036		1.16273		0.729178	4.21227
STE12	YLR228C	6.86226								6.86226
STE12	YLR243W				2.21052		1.21621		0.696505	4.12324
STE12	YLR257W			1.04692	1.73562		1.88711		0.456561	5.12621
STE12	YLR268W			1.60979		2.47011	1.71169			5.79159
STE12	YLR274W				2.86479		1.22664			4.09143
STE12	YLR275W	4.64933	0.205071			3.419	2.02573			10.2991
STE12	YLR293C				1.15706	1.2372	1.24561		0.713391	4.35326
STE12	YLR309C						2.07209	2.81386		4.88595
STE12	YLR314C						2.07543	3.00653		5.08196
STE12	YLR323C			2.4971			2.00261			4.49971
STE12	YLR332W	4.03522	0.205071		0.447683				0.56656	5.25453
STE12	YLR335W				0.424568	1.2372	1.83724		0.663187	4.16219
STE12	YLR340W			0.834308	1.39867		1.12648		0.696778	4.05624
STE12	YLR353W	8.201								8.201
STE12	YLR354C	0.273328			2.45623	1.71811	0.851497			5.29917
STE12	YLR362W					4.11544	1.73308			5.84852
STE12	YLR413W	2.87337		1.99028	2.44661				0.712969	8.02323
STE12	YLR417W					4.11544				4.11544
STE12	YLR424W					3.25978	0.912731			4.17251
STE12	YLR431C						1.96442	3.41601		5.38043
STE12	YLR452C	15.8551			2.8761	4.11544			0.710249	23.5569
STE12	YLR453C	4.12212				3.2218				7.34392
STE12	YLR454W	4.12212								4.12212
STE12	YLR457C	0.157493			3.56081	4.11544				7.83374
STE12	YLR466W						1.92052	3.08778		5.0083
STE12	YLR467W		0.205071				1.70069	3.04517		4.95093
STE12	YML005W	2.18959	0.205071		2.88018			1.19307		6.46791
STE12	YML024W			0.41051	2.04156		1.22081		0.729339	4.40222
STE12	YML038C				0.851142	4.11544				4.96658
STE12	YML046W	5.78977				3.419				9.20877
STE12	YML047C	5.78977			2.93665					8.72642
STE12	YML055W		0.205071	4.64329	0.432952					5.28131
STE12	YML085C					4.11544			0.691535	4.80698
STE12	YML093W				1.51523		1.80877	0.984071	0.728579	5.03665
STE12	YML124C	1.16207	0.205071			2.97541	0.802609		0.628672	5.77383
STE12	YML125C			3.54403			0.869771		0.706477	5.12028
STE12	YML126C			2.92392	2.83602	0.901149	1.2712		0.573269	8.50556
STE12	YML133C				0.486766		1.68445	3.04517		5.21639
STE12	YMR001C			0.773829	1.30003		1.87443	0.85909	0.692418	5.4998
STE12	YMR004W					4.11544				4.11544
STE12	YMR012W				0.924968	0.73423	2.02057		0.713345	4.39311
STE12	YMR023C	1.61494	0.205071	2.50544						4.32545
STE12	YMR043W	1.4366				4.11544	1.95849			7.51053
STE12	YMR065W	4.57056			1.75233		0.866301			7.18919

TF	Target	Binding_0-2000	Motif_Score_SC	Other_Yeasts	Two-Hop_BE	Two-Hop_BP	Two-Hop_BY	Two-Hop_BG	Max_Membership	Sum
STE12	YMR075W	1.30031					2.05602	1.37632		4.73265
STE12	YMR116C				2.47206	2.97732	1.21983		0.69845	7.36766
STE12	YMR119W	1.81809			3.5933		0.844076			6.25547
STE12	YMR121C	0.0987851		0.828276	1.51956		1.26314		0.729028	4.43879
STE12	YMR125W		0.205071	0.916845		3.25978				4.3817
STE12	YMR131C			4.58869	1.1205		1.31637		0.729688	7.75525
STE12	YMR133W			4.38814	1.47122					5.85936
STE12	YMR146C				0.562362		1.28943	1.63377	0.717496	4.20306
STE12	YMR164C	6.24967								6.24967
STE12	YMR167W			0.256794		2.93572	0.926474			4.11899
STE12	YMR197C	6.22495				0.641359				6.86631
STE12	YMR198W	6.22495				0.392196	2.05347	0.963722		9.63434
STE12	YMR199W	6.72083			2.00698	1.43298			0.574583	10.7354
STE12	YMR204C	1.11315	0.205071	4.15932						5.47754
STE12	YMR222C	3.74137					1.87141			5.61278
STE12	YMR223W	3.74137			1.23251		2.00261			6.97649
STE12	YMR232W	4.42981	0.205071		0.783919					5.4188
STE12	YMR235C	5.25619			1.8071			1.43261	0.532414	9.02831
STE12	YMR236W	5.25619		0.269603						5.52579
STE12	YMR237W	5.25619								5.25619
STE12	YMR242C			0.550722	1.87961		1.38954		0.726555	4.54643
STE12	YMR246W	2.93789			0.630281		0.914017	1.60955	0.583816	6.67555
STE12	YMR293C			2.77154			1.43595			4.20749
STE12	YMR305C	5.35238			2.14999				0.714726	8.2171
STE12	YMR306W	5.35238								5.35238
STE12	YNL001W				2.24435		1.27279		0.589974	4.10711
STE12	YNL006W				2.56983		1.91039			4.48022
STE12	YNL011C		0.205071	5.17126						5.37633
STE12	YNL025C	2.44865					1.87829			4.32694
STE12	YNL042W-B	5.78809								5.78809
STE12	YNL053W	9.8696						0.901459		10.7711
STE12	YNL061W		0.205071	0.0673007	1.09555	2.27958			0.636523	4.28402
STE12	YNL067W				2.14821		1.23182		0.69605	4.07608
STE12	YNL079C					1.09168	2.09021	1.2638		4.44569
STE12	YNL087W	4.18859			0.728772		2.00261		0.715023	7.63499
STE12	YNL106C	4.38135				0.396194				4.77754
STE12	YNL110C				2.2872	0.819222	0.917045	1.01014	0.724475	5.75808
STE12	YNL145W	11.6321			3.23476					14.8669
STE12	YNL146C-A	11.6321								11.6321
STE12	YNL148C	11.6321								11.6321
STE12	YNL169C	0.334138	0.205071	3.63038					0.684433	4.85402
STE12	YNL175C		0.205071		1.1468		0.970772	1.25145	0.72884	4.30293
STE12	YNL178W	2.0159			2.46116		1.25639		0.69735	6.4308
STE12	YNL180C	2.0159					2.04834			4.06424
STE12	YNL189W					1.81042	2.00261		0.599907	4.41294
STE12	YNL192W	6.24111			2.8258	0.5517				9.61861
STE12	YNL207W	4.43973				2.27958	1.306		0.450312	8.47562
STE12	YNL208W	4.43973		0.452114	2.88176					7.7736
STE12	YNL216W	3.16684				0.948071				4.11491
STE12	YNL225C				1.22757		2.08325	1.73234		5.04316
STE12	YNL255C				1.74837			1.59126	0.731712	4.07134
STE12	YNL272C	5.95204		0.285286			2.09425			8.33158
STE12	YNL279W	15.8551	0.205071		2.49835					18.5585
STE12	YNL280C	15.8551		0.103191						15.9583
STE12	YNL289W	15.8551		1.54005						17.3952
STE12	YNL291C	15.8551								15.8551
STE12	YNL298W	12.3783			1.29828					13.6766
STE12	YNL304W	5.33171					2.04834		0.658382	8.03843
STE12	YNL307C	5.33171					1.98861			7.32032
STE12	YNL321W	5.6099								5.6099
STE12	YNL322C	5.6099		0.92444	0.848947					7.38329
STE12	YNL327W	0.35047			0.992505		2.06793		0.711625	4.12253
STE12	YNL339C						1.96308	3.04517		5.00825
STE12	YNR006W						1.9172	2.99645		4.91365
STE12	YNR026C	6.64287								6.64287
STE12	YNR028W	6.64287								6.64287
STE12	YNR044W	2.59252			2.93363				0.709255	6.2354
STE12	YNR053C		0.205071	1.86275	0.833024		0.825795		0.714019	4.44066
STE12	YNR054C	4.23401			0.991509				0.530267	5.75579
STE12	YNR067C				2.19256		1.79087		0.619425	4.60285
STE12	YOL006C						2.03887	3.00497		5.04384
STE12	YOL018C					4.11544			0.684459	4.7999
STE12	YOL030W	3.71428			0.54996				0.633032	4.89727
STE12	YOL031C	3.71428			0.612979					4.32726
STE12	YOL039W	0.375397	0.205071	3.43358	1.50325		1.22199		0.542078	7.28137
STE12	YOL040C	0.375397		0.36272	1.39629		1.27872		0.699987	4.11311
STE12	YOL041C	0.375397	0.205071	6.19164	1.06836		2.02714		0.451007	10.3186
STE12	YOL052C-A			4.74722						4.74722
STE12	YOL082W	4.22705		0.697446						4.9245
STE12	YOL090W				1.65103	1.36958	1.60679		0.551439	5.17884
STE12	YOL108C		0.205071	0.722055		3.67116				4.59829
STE12	YOL115W			4.0799						4.0799
STE12	YOL128C			2.40432			1.88884			4.29316
STE12	YOL138C			4.54033	0.707358					5.24769
STE12	YOL159C	4.61628			1.37895					5.99523
STE12	YOL165C	3.91967			1.16826					5.08793
STE12	YOR020C			4.54284						4.54284
STE12	YOR036W				1.07918	4.11544	2.05395			7.24857
STE12	YOR075W				2.51508			1.75582		4.2709
STE12	YOR086C	5.77294	0.205071							5.97801
STE12	YOR087W	5.77294								5.77294
STE12	YOR094W	2.32368					1.99707			4.32075
STE12	YOR096W	2.2847			1.89298				0.625809	4.80349
STE12	YOR129C	6.24096		0.025038						6.266
STE12	YOR130C	3.76069			0.471765					4.23245
STE12	YOR145C				1.99947	0.843276	1.28406		0.730093	4.8569
STE12	YOR165W				1.23114		1.95708	1.56355	0.575974	5.32774
STE12	YOR177C						2.1128	2.98885		5.10165
STE12	YOR191W				2.97814		0.861756	0.90113		4.74103
STE12	YOR198C		0.205071		0.797035		2.09502	1.66649		4.76362
STE12	YOR212W	0.12817				4.11544	1.92076			6.16437
STE12	YOR216C						0.942126	3.41601		4.35814
STE12	YOR234C	1.82199			1.92345				0.728391	4.47383
STE12	YOR246C	2.09813		2.16391			0.927069			5.18911
STE12	YOR247W	2.09813		3.66349	1.55341					7.31503
STE12	YOR273C			3.03844			1.59786		0.556685	5.19299
STE12	YOR287C				2.12389		1.36487		0.631638	4.1204
STE12	YOR288C	3.95876		0.118005	1.50784		1.5042			7.0888
STE12	YOR289W				2.5705		1.50517			4.07567
STE12	YOR312C				2.01306		1.38954		0.72231	4.12491

TF	Target	Binding_0-2000	Motif_Score_SC	Other_Yeasts	Two-Hop_BE	Two-Hop_BP	Two-Hop_BY	Two-Hop_BG	Max_Membership	Sum
STE12	YOR315W	3.58177			0.43858				0.65267	4.67302
STE12	YOR326W					1.2746	2.07816	3.39578	0.709707	7.45825
STE12	YOR342C	8.0094								8.0094
STE12	YOR344C	4.43311		0.197935	0.834244				0.610278	6.07557
STE12	YOR353C					0.5321	1.97552	2.78393		5.29155
STE12	YOR369C				1.36837		2.05823		0.701818	4.12842
STE12	YOR396W						1.89445	3.08778		4.98223
STE12	YPL031C				0.425404	4.11544	1.81192			6.35276
STE12	YPL037C				1.23644	2.66881			0.641305	4.54655
STE12	YPL043W				1.18842	0.819222	0.908856	0.956815	0.695291	4.5686
STE12	YPL048W	5.67306							0.586409	6.25947
STE12	YPL049C	5.67306				4.11544	1.89131			11.6798
STE12	YPL075W		0.205071		2.55974	0.690189	2.00824			5.46324
STE12	YPL079W				2.0067		1.40065		0.702792	4.11014
STE12	YPL131W			1.41823	1.38179		1.22872		0.691222	4.71996
STE12	YPL141C	0.416935	0.205071	1.42407	0.875896			0.860776	0.706048	4.4888
STE12	YPL146C	1.31913			2.07246				0.630626	4.02222
STE12	YPL151C	0.0792108		0.867632		3.25978				4.20662
STE12	YPL154C			0.159961	2.84174		1.98861			4.99031
STE12	YPL156C	5.96908			2.82001					8.78909
STE12	YPL163C	11.4851			1.72779				0.5241	13.737
STE12	YPL164C	11.4851					0.92565			12.4107
STE12	YPL174C						2.05107	3.06137		5.11244
STE12	YPL177C	1.96485					2.05602			4.02087
STE12	YPL178W					3.22846	0.897458	1.50498		5.6309
STE12	YPL183C				0.326389		1.22834	1.95457	0.679874	4.18917
STE12	YPL186C			0.142388	2.20581		2.088	0.901459		5.33766
STE12	YPL192C	5.55781		0.0311115	2.91555					8.50447
STE12	YPL198W				2.05251		1.31234		0.721324	4.08617
STE12	YPL203W				0.561178		1.98861	0.859094	0.731132	4.14001
STE12	YPL211W			1.73016	1.54717	3.67116	1.36946		0.732064	9.05001
STE12	YPL218W			2.60909			1.84618			4.45527
STE12	YPL237W				2.28663		1.18959		0.614136	4.09036
STE12	YPL239W				2.23227		1.39258		0.723903	4.34875
STE12	YPL247C			1.04814	0.980184		2.06905			4.09737
STE12	YPL253C					0.392196	1.98396	3.41607		5.79223
STE12	YPL256C				2.2211	1.43298			0.676277	4.33036
STE12	YPL283C						1.70069	3.04517		4.74586
STE12	YPR005C	4.14815								4.14815
STE12	YPR028W				1.00877		2.02573	1.7831		4.8176
STE12	YPR043W		0.205071		2.17018		1.24657		0.726837	4.34866
STE12	YPR049C		0.205071				2.09624	3.4069		5.70821
STE12	YPR072W			0.365539		0.42155	1.99132	1.30775		4.08616
STE12	YPR075C	5.18266	0.205071	1.55495						6.94268
STE12	YPR079W	5.18266	0.205071	1.02213						6.40986
STE12	YPR110C				1.21325	1.49629	1.22242		0.731017	4.66298
STE12	YPR119W	4.03489			1.25302	2.32362	1.94091		0.660814	10.2133
STE12	YPR120C	3.92883			0.507419					4.43625
STE12	YPR122W	5.74608		0.0522211						5.7983
STE12	YPR133C	6.40981					2.06905	1.07679		9.55565
STE12	YPR135W	6.40981							0.711185	7.121
STE12	YPR141C	2.06298		0.630648	0.388786	1.51574	2.0932	2.94903		9.64038
STE12	YPR155C	4.26488								4.26488
STE12	YPR165W	2.33751			2.28442	1.21566	2.04834			7.88593
STE12	YPR182W					3.22846	1.10765			4.33611
STE12	YPR202W				1.52971			3.40826		4.93797
STP1	TT(AGU)C	4.65789								4.65789
STP1	TT(AGU)J	5.26447								5.26447
STP1	TW(CCA)J	5.48713								5.48713
STP1	YAL053W		1.76548	4.30177						6.06725
STP1	YAL054C		1.76548	3.168						4.93348
STP1	YBL101C		3.35279	1.00045						4.35324
STP1	YBR068C	1.09011	2.92442	8.50907						12.5236
STP1	YBR069C	0.683414	1.84897	4.16159						6.69397
STP1	YCL024W	9.52262		0.637349						10.16
STP1	YCL025C	9.52262		0.641842						10.1645
STP1	YCL039W		3.35279	9.30066						12.6535
STP1	YCL043C		3.35279	2.82757						6.18036
STP1	YCL066W		2.92442			1.0927				4.01712
STP1	YCR010C		1.76548	8.92501						10.6905
STP1	YCR057C		1.2052	2.13406	0.34734	0.512361				4.19896
STP1	YCR059C		2.08618	4.38298						6.46916
STP1	YCR096C	4.65789				1.0927				5.75059
STP1	YCR097W	4.65789				1.0927				5.75059
STP1	YDL180W	2.94018	1.58549							4.52567
STP1	YDL182W	4.38001								4.38001
STP1	YDL183C	4.38001		0.293348						4.67336
STP1	YDR006C		1.59781	2.54943						4.14724
STP1	YDR007W		1.59781	3.75779						5.3556
STP1	YDR034W-B	0.946345		6.51132						7.45767
STP1	YDR042C	4.09743								4.09743
STP1	YDR084C	4.45568								4.45568
STP1	YDR091C			3.47059	0.812387					4.28298
STP1	YDR155C		1.59781	4.31017		0.454301				6.36228
STP1	YDR158W		1.59781	2.64447						4.24228
STP1	YDR171W		1.49853	5.43091						6.92944
STP1	YDR310C		1.75353	2.93362						4.68715
STP1	YDR384C		2.17576	2.67138						4.84714
STP1	YDR385W		2.17576	2.46972						4.64548
STP1	YDR489W		1.49853	2.90001						4.39854
STP1	YDR508C	5.188		0.301529						5.48953
STP1	YDR510W	5.188		0.774229		0.411022				6.37325
STP1	YDR511W	5.188								5.188
STP1	YEL024W		1.76548	5.0565	0.835115					7.65709
STP1	YEL025C		1.76548	3.30573						5.07121
STP1	YEL034W			5.7432	1.22019					6.96339
STP1	YEL036C			5.38878						5.38878
STP1	YEL043W	3.43106	1.58549	0.345747	0.389014	0.445306				6.19662
STP1	YEL044W	3.43106	1.58549	1.14332						6.15987
STP1	YEL046C	3.43106	1.58549	1.14618						6.16273
STP1	YEL060C			5.21175						5.21175
STP1	YER054C		1.48599	3.74571						5.2317
STP1	YFL008W	0.251266		2.96491				0.860225		4.0764
STP1	YFR017C		1.57006	2.3745	0.333822					4.27838
STP1	YGL001C	5.14798			0.375432					5.52341
STP1	YGL064C		1.49853	3.26663						4.76516
STP1	YGL072C		2.93414	2.65407						5.58821
STP1	YGL125W		1.76548	5.58588						7.35136
STP1	YGR055W	3.77046	1.21782	0.847576						5.83586
STP1	YGR071C	6.84234								6.84234

TF	Target	Binding_0-2000	Motif_Score_SC	Other_Yeasts	Two-Hop_BE	Two-Hop_BP	Two-Hop_BY	Two-Hop_BG	Max_Membership	Sum
STP1	YGR072W	6.84234								6.84234
STP1	YGR074W	6.84234								6.84234
STP1	YGR117C	1.00607	1.82242	4.08282						6.91131
STP1	YGR138C		1.48599	2.83295						4.31894
STP1	YIL004C	5.85699								5.85699
STP1	YIL031W	0.660855	3.54651	1.9458						6.15317
STP1	YIL033C	0.660855	3.54651	1.8651						6.07247
STP1	YIL034C		3.54651	1.08759						4.6341
STP1	YIL057C		2.71789	9.66301						12.3809
STP1	YJL011C	0.783748		3.67776						4.46151
STP1	YJL044C		2.02043	3.82551						5.84594
STP1	YJL102W		1.75353	4.00706						5.76059
STP1	YJL198W	5.26447								5.26447
STP1	YJL200C	5.26447					0.829697			6.09417
STP1	YJR010C-A	7.23726								7.23726
STP1	YJR011C	7.23726			0.328403					7.56566
STP1	YJR013W	7.23726								7.23726
STP1	YKL032C		1.84897	3.09517						4.94414
STP1	YKL166C			4.13407						4.13407
STP1	YKR048C		2.08618			2.21542				4.3016
STP1	YKR049C		2.08618	6.21421						8.30039
STP1	YKR050W		2.08618	4.01792	0.369576					6.47368
STP1	YKR066C		1.48599	6.79967						8.28566
STP1	YKR067W		1.48599	4.29296						5.77895
STP1	YLL038C			6.92454						6.92454
STP1	YLR007W		2.08618	3.66409						5.75027
STP1	YLR136C		2.02043	2.58035						4.60078
STP1	YLR137W		2.02043	2.66877						4.6892
STP1	YLR263W	3.53017				0.858561				4.38873
STP1	YLR270W			4.16574						4.16574
STP1	YLR356W			5.45483						5.45483
STP1	YMR019W	4.95913								4.95913
STP1	YMR091C			4.48619						4.48619
STP1	YMR135C	0.209971	1.76548	2.52799						4.50344
STP1	YMR145C		1.30971	2.15701	0.811703					4.27842
STP1	YMR251W-A		2.11151	6.14075						8.25226
STP1	YNL186W			4.41997						4.41997
STP1	YNL193W		3.15181	6.47389						9.6257
STP1	YNL194C		3.15181	8.84125	0.741037					12.7341
STP1	YNR016C		1.30971	2.23211				1.3101		4.85192
STP1	YNR017W		1.30971	3.04528						4.35499
STP1	YNR049C	4.34053								4.34053
STP1	YOL011W		2.11151	3.22901						5.34052
STP1	YOL012C		2.11151	3.22629						5.3378
STP1	YOL055C			4.21285						4.21285
STP1	YOL057W	4.98273								4.98273
STP1	YOL058W	4.96072					0.865361			5.82608
STP1	YOL125W	2.16262	1.48599	1.75744	0.355491					5.76154
STP1	YOL126C	2.16262	1.48599	0.698195						4.3468
STP1	YOR081C		1.76548	4.21864						5.98412
STP1	YOR084W		1.76548	5.98962						7.7551
STP1	YOR273C	1.34769	1.48599	1.2307						4.06438
STP1	YOR274W	1.34769	1.48599	1.21504						4.04872
STP1	YOR301W		0.699307	3.893						4.59231
STP1	YOR375C		1.48599	7.89768						9.38367
STP1	YPL015C		0.909199	3.97719						4.88639
STP1	YPL112C		2.08618	2.92508						5.01126
STP1	YPL265W	2.31397	1.48599	1.32139						5.12135
STP1	YPL273W	1.58734	0.149752	1.60792			0.787725			4.13274
STP1	YPR001W		1.57006	2.56887						4.13893
STP1	YPR131C		1.82242	2.21773						4.04015
STP1	YPR144C	1.60642		1.68219	0.83973					4.12834
STP1	YPR145W	1.60642		1.76341	0.723349					4.09318
STP2	YDL186W	4.13217								4.13217
STP4	YIL159W	4.04213								4.04213
STP4	YIL160C	4.04213				0.423311				4.46544
STRE	YBL077W			4.44708						4.44708
STRE	YBL105C			6.28332						6.28332
STRE	YBR072W			5.71925						5.71925
STRE	YBR126C			9.12102						9.12102
STRE	YCL040W			4.21538						4.21538
STRE	YCL051W			9.80954						9.80954
STRE	YCL052C			9.0205						9.0205
STRE	YCL054W			4.48645						4.48645
STRE	YCR075C			5.59036						5.59036
STRE	YDR510W			4.15254						4.15254
STRE	YDR511W			4.48068						4.48068
STRE	YER067W			6.86024						6.86024
STRE	YFR015C			4.90305						4.90305
STRE	YFR017C			5.0201						5.0201
STRE	YFR053C			5.85269						5.85269
STRE	YGL006W			6.81569						6.81569
STRE	YGR254W			8.10441						8.10441
STRE	YHR025W			5.87448						5.87448
STRE	YIR013C			4.06813						4.06813
STRE	YIR033W			4.17096						4.17096
STRE	YJL052W			6.7279						6.7279
STRE	YJL076W			4.94188						4.94188
STRE	YLL055W			4.40381						4.40381
STRE	YLR327C			6.32966						6.32966
STRE	YNL234W			7.91754						7.91754
STRE	YPL050C			6.3297						6.3297
STRE	YPL219W			4.13028						4.13028
STRE	YPR074C			8.45115						8.45115
STRE	YPR100W			4.11797						4.11797
STRE	YPR128C			5.13201						5.13201
SUM1	YAL017W	2.14954	0.376134	4.04276						6.56843
SUM1	YAL018C		0.376134	5.23893						5.61506
SUM1	YAL023C	3.92347	0.175503	1.5979						5.69687
SUM1	YBR017C	4.4247	0.130528	1.7776						6.33283
SUM1	YBR084W	1.73219	0.459454	3.69134		0.451719				6.3347
SUM1	YBR148W	4.75576		1.77945						6.53521
SUM1	YBR249C		0.376134	7.52701	0.408496					8.31164
SUM1	YBR250W		0.376134	7.46035						7.83648
SUM1	YCL048W	2.96601	0.213585	2.53898						5.71857
SUM1	YCR045C			7.69721						7.69721
SUM1	YDL079C			4.81435	1.06296					5.87731
SUM1	YDL114W	0.256025	0.130528	4.20493	1.15166					5.74314
SUM1	YDL115C		0.130528	4.16914						4.29967
SUM1	YDL123W		0.346808	4.49722						4.84403

TF	Target	Binding_0-2000	Motif_Score_SC	Other_Yeasts	Two-Hop_BE	Two-Hop_BP	Two-Hop_BY	Two-Hop_BG	Max_Membership	Sum
SUM1	YDL145C			1.17938	0.83178	1.47625	1.15057	1.30405		5.94203
SUM1	YDL185W	8.9886	0.376134		0.836467					10.2012
SUM1	YDL186W	8.9886	0.376134	3.04568						12.4104
SUM1	YDL188C	8.9886	0.376134	3.44122						12.806
SUM1	YDL211C			7.82658						7.82658
SUM1	YDR042C	3.33214		0.999225						4.33136
SUM1	YDR121W			6.62188						6.62188
SUM1	YDR281C	2.49385		4.96164	0.593224					8.04871
SUM1	YDR310C	4.07434		0.710656						4.785
SUM1	YDR311W	4.07434		0.329606		2.17306				6.57701
SUM1	YDR322W	4.07219	0.386299	4.11169						8.57018
SUM1	YDR402C	4.97315								4.97315
SUM1	YDR403W	4.97315								4.97315
SUM1	YDR522C	8.31313								8.31313
SUM1	YDR523C	4.49779		0.575906		1.48029				6.55399
SUM1	YEL006W			4.81568						4.81568
SUM1	YEL037C		0.118562	2.39556		0.935173	0.799516			4.24881
SUM1	YER085C	0.26465		6.83904						7.10369
SUM1	YER086W	0.26465		10.7696						11.0343
SUM1	YER105C	1.41871	0.301888	3.01814						4.73874
SUM1	YER106W	1.41871	0.301888	3.40174						5.12234
SUM1	YFL010W-A	4.99005	0.342972							5.33302
SUM1	YFL040W	4.34318		0.201309						4.54449
SUM1	YFR016C			4.87917						4.87917
SUM1	YFR031C-A	2.98927	0.342972		1.12613					4.45837
SUM1	YFR032C	2.98927	0.342972	0.700897						4.03314
SUM1	YGL082W			4.1666						4.1666
SUM1	YGL137W	6.78449	0.579729	10.1257	1.28671					18.7766
SUM1	YGL138C	6.78449	0.579729	8.42144						15.7857
SUM1	YGL169W	2.91281	0.301888	3.6169						6.8316
SUM1	YGL170C	2.91281	0.301888	4.62741						7.84211
SUM1	YGR043C		0.301888	5.19434	0.363352					5.85958
SUM1	YGR258C	8.65985	0.251412	3.06494		0.935173				12.9114
SUM1	YGR260W	8.65985	0.251412	0.934486						9.84575
SUM1	YGR273C		0.342972	4.24096						4.58393
SUM1	YHR015W	0.680292	0.130528	2.33986	0.996695					4.14738
SUM1	YHR124W	1.39187	0.380583	3.84857						5.62102
SUM1	YHR184W	2.40965	0.508119	7.49534						10.4131
SUM1	YHR185C	3.69563		0.692014						4.38764
SUM1	YHR195W		0.342972	4.35941						4.70238
SUM1	YIR027C	8.66603		1.14945						9.81548
SUM1	YIR028W	8.66603		1.17589						9.84192
SUM1	YJL043W	2.10131	0.459454	4.31615						6.87691
SUM1	YJL044C	2.10131	0.459454	8.51773						11.0785
SUM1	YJL052W		0.175503	2.21699	1.71094					4.10343
SUM1	YJR024C	2.35063	0.305752	3.98838						6.64476
SUM1	YJR025C	2.35063	0.305752	5.53171						8.18809
SUM1	YJR077C	5.60808		0.474984						6.08306
SUM1	YJR078W	5.60808		1.38806						6.99614
SUM1	YJR079W	5.60808								5.60808
SUM1	YKL104C		0.579729	10.0968						10.6765
SUM1	YKR031C	4.17552	0.24751							4.42303
SUM1	YKR035W-A	4.17552	0.24751	3.21984						7.64287
SUM1	YLR049C		0.579729	11.0652						11.6449
SUM1	YLR053C			5.8124						5.8124
SUM1	YLR134W		0.141142	6.2171						6.35824
SUM1	YLR213C	1.86505	0.579729	7.96196						10.4067
SUM1	YLR214W	1.86505	0.579729	7.23026	0.404685					10.0797
SUM1	YLR231C	1.09555		3.67269						4.76824
SUM1	YLR308W	1.50748	0.579729	7.6649						9.75211
SUM1	YLR341W	1.17731		4.79534						5.97265
SUM1	YLR343W	2.98404	0.0529715	0.99029						4.0273
SUM1	YML066C			4.64183						4.64183
SUM1	YMR125W			6.35453						6.35453
SUM1	YMR232W		0.342972	4.23499	0.937064					5.51503
SUM1	YNL016W			9.14065	1.11127					10.2519
SUM1	YNL034W			4.25538	0.459018					4.7144
SUM1	YNL035C		0.583408	9.25872						9.84213
SUM1	YNL105W			5.60676						5.60676
SUM1	YNL199C		0.511846	4.67022		0.790979				5.97304
SUM1	YNL201C		0.583408	12.124						12.7074
SUM1	YNL317W	4.39959		0.390581						4.79017
SUM1	YNL318C	4.39959								4.39959
SUM1	YNL328C	0.236541	0.251412	3.92959						4.41754
SUM1	YOL015W		0.251412	4.04203						4.29344
SUM1	YOL091W	0.649248		11.5205						12.1697
SUM1	YOL102C	4.97319	0.251412	5.99355						11.2182
SUM1	YOL132W			4.3165						4.3165
SUM1	YOR027W				0.653283	0.88528	1.14955	1.85985		4.54796
SUM1	YOR213C	3.82689		1.27348						5.10037
SUM1	YOR264W		0.118562	3.92295						4.04151
SUM1	YOR311C	0.248223		6.15548						6.4037
SUM1	YOR312C	5.42386			0.628804					6.05266
SUM1	YOR313C	5.42386		7.74674	0.719737					13.8903
SUM1	YOR315W	5.42386			0.719737					6.1436
SUM1	YOR339C		0.508119	7.99838						8.5065
SUM1	YOR362C			0.850664		2.07127	1.25011			4.17204
SUM1	YOR365C	5.15776	0.000897989	1.13035						6.28901
SUM1	YOR367W	5.15776	0.000897989	1.10695						6.26561
SUM1	YOR368W	5.15776	0.000897989							5.15866
SUM1	YPL033C	0.435505		4.42214						4.85764
SUM1	YPL075W	3.54021	0.135174	2.73221						6.40759
SUM1	YPL082C			4.92664						4.92664
SUM1	YPL129W	2.53817	0.175503	1.44172						4.15539
SUM1	YPL255W	0.964622	0.508119	5.56867						7.04141
SUM1	YPL256C	0.964622	0.508119	7.08409						8.55683
SUT1	YAL019W		1.00903	4.11903						5.12806
SUT1	YAL020C		1.00903	5.53207						6.5411
SUT1	YAL063C	4.05702								4.05702
SUT1	YBL029C-A	4.70276	0.592597	0.657826						5.95318
SUT1	YBL029W	4.70276	0.592597	0.532978						5.82833
SUT1	YBL033C		0.348086	4.9972						5.34529
SUT1	YBL043W	5.95274		2.07389	0.874016					8.90065
SUT1	YBL045C	5.95274			0.427178					6.37992
SUT1	YBR069C	3.00638	1.39269	3.98889						8.38796
SUT1	YBR071W	3.00638	1.39269							4.39907
SUT1	YBR106W		1.32156	10.1411	0.47987					11.9425
SUT1	YBR297W		1.12554	4.62865	0.764242					6.51843
SUT1	YCL036W		1.53648	6.92746						8.46394
SUT1	YDL047W	0.863476		6.65573						7.51921

TF	Target	Binding_0-2000	Motif_Score_SC	Other_Yeasts	Two-Hop_BE	Two-Hop_BP	Two-Hop_BY	Two-Hop_BG	Max_Membership	Sum
SUT1	YDL048C	0.863476		3.31613						4.17961
SUT1	YDR073W	0.0624429	1.07574	3.47722						4.6154
SUT1	YDR231C		0.778041	2.93678	0.769573					4.48439
SUT1	YDR343C	3.53203			0.560618					4.09265
SUT1	YDR441C	5.07169	0.111238							5.18293
SUT1	YEL044W			4.09708						4.09708
SUT1	YEL046C			4.57114						4.57114
SUT1	YER088C		1.70897	9.87033						11.5793
SUT1	YFR033C		0.970794	8.57645	0.707756					10.255
SUT1	YGL035C		1.52507	3.62195						5.14702
SUT1	YGL055W	2.18842	0.421733	1.70354						4.31369
SUT1	YGL158W	3.18034	0.53477	0.437712						4.15282
SUT1	YGL163C	3.20278	0.00293581	0.964303						4.17002
SUT1	YGR067C	4.48511	1.28918	3.28214	1.17381					10.2302
SUT1	YGR195W			4.20023						4.20023
SUT1	YHL004W		1.13945	4.56567						5.70512
SUT1	YHL027W	2.82419		1.46107						4.28526
SUT1	YHR007C	0.372399	1.01187	3.006						4.39027
SUT1	YHR050W	1.05788	1.23042	2.03077						4.31907
SUT1	YHR137W	0.600526		4.60399						5.20452
SUT1	YIL056W		0.635887	10.6853						11.3212
SUT1	YIL057C		0.635887	9.70331	0.564169					10.9034
SUT1	YIL101C	3.04671	1.34948							4.39619
SUT1	YIL118W	4.75086	0.627377							5.37824
SUT1	YIL119C	4.75086	0.627377							5.37824
SUT1	YIL121W		0.526107	5.37135						5.89746
SUT1	YIR019C	4.67634								4.67634
SUT1	YKL032C		0.885418	3.34881						4.23423
SUT1	YKL062W	4.12257	1.34627	9.37247	0.439987					15.2813
SUT1	YKL063C	4.12257	1.34627	0.21821						5.68705
SUT1	YKL109W	2.62248	0.760837	1.72961						5.11293
SUT1	YKR042W		0.970794	3.17627	0.337102					4.48417
SUT1	YLR113W	1.40801	0.143793	5.03171						6.58351
SUT1	YLR153C	2.79968	0.52239		0.933667					4.25574
SUT1	YLR304C	3.32821	1.87392							5.20213
SUT1	YMR037C	4.03634								4.03634
SUT1	YMR070W	4.72642	1.34948							6.0759
SUT1	YMR122W-A	0.679283		3.35777						4.03705
SUT1	YMR135C	4.3089	1.52507	1.82825						7.66222
SUT1	YMR136W	4.3089	1.52507	1.7934						7.62737
SUT1	YNL178W	2.69123	1.19035		0.645974					4.52755
SUT1	YNL216W			6.96654						6.96654
SUT1	YOL084W	0.549106	1.70897	4.15694	0.431728					6.84674
SUT1	YOR084W	1.17524	1.12944	3.05612						5.3608
SUT1	YOR138C		1.31726	4.35244						5.6697
SUT1	YOR140W		1.23042	4.15108						5.3815
SUT1	YOR178C	3.04978	1.84455		0.336965					5.2313
SUT1	YOR245C		0.0778247	4.34877						4.42659
SUT1	YOR301W		0.635887	4.88211						5.518
SUT1	YOR332W		1.53648	5.95429						7.49077
SUT1	YOR370C		1.40968	5.90561						7.31529
SUT1	YPL024W	3.70574		0.51651						4.22225
SUT1	YPL026C	3.70574		0.488449						4.19419
SUT1	YPL177C	2.81914	0.838141	0.621502						4.27878
SUT1	YPL232W			4.54572		0.676522				5.22224
SUT1	YPL256C			6.98939						6.98939
SUT1	YPR006C	2.09051		2.07692						4.16743
SUT1	YPR151C	1.55373	0.307684	2.42991						4.29132
SUT1	YPR192W	1.07346		3.79298						4.86644
SWI4	SNR40	11.5513	0.330165							11.8815
SWI4	YAL020C		0.494436	2.27909	1.39732					4.17085
SWI4	YAR007C		0.164293		1.74196	1.05856	1.06056			4.02537
SWI4	YBL002W	3.41306	0.722683		1.63043				0.730527	6.4967
SWI4	YBL003C	3.41306	0.722683		1.75646		1.26553		0.730926	7.88866
SWI4	YBL063W		0.254011	2.31397	1.63773		1.07507	1.1061		6.38688
SWI4	YBL064C			4.35407	0.999099					5.35317
SWI4	YBR009C	0.826115		0.624953	2.74736	1.59208	1.14382		0.729277	7.6636
SWI4	YBR010W	0.826115	0.254011	0.707403	1.86871	1.59208	1.3202		0.72791	7.29643
SWI4	YBR069C	8.61486	0.254011	0.164065					0.723838	9.75677
SWI4	YBR070C	8.61486	0.254011	2.35937	1.10444				0.710669	13.0434
SWI4	YBR071W	8.61486	0.254011	3.2796	1.36827				0.707254	14.224
SWI4	YBR077C	8.64676								8.64676
SWI4	YBR078W	8.64676	0.254011		1.62782				0.652121	11.1807
SWI4	YBR088C		0.840485	6.93111	1.53029		0.95103		0.732264	10.9852
SWI4	YBR162C	8.73238	0.840485	5.0406	1.62801			2.18869	0.727658	19.1578
SWI4	YBR162W-A	8.73238	0.840485	4.95771					0.728066	15.2586
SWI4	YBR163W	8.73238	0.840485	5.71066					0.540652	15.8242
SWI4	YBR202W		0.254011	3.25555			1.02507			4.53463
SWI4	YCL055W				2.69953	1.37337				4.0729
SWI4	YCL059C			0.423753	2.74978	1.00617			0.730058	4.90976
SWI4	YCR065W	11.6968	1.02663	6.55325	0.992496				0.560804	20.83
SWI4	YCR067C		0.254011		2.74839		0.978685	1.73328		5.71437
SWI4	YDL003W	1.50398	0.840485	4.40229	1.71274				0.725901	9.1854
SWI4	YDL006W					1.59519	1.13706	1.30899		4.04124
SWI4	YDL055C	3.08984	0.840485	5.18794	2.75069				0.731108	12.6001
SWI4	YDL124W	2.36142			1.23801		1.15314		0.623849	5.37642
SWI4	YDL127W	7.91717	1.22768	5.70854					0.661532	15.5149
SWI4	YDL154W		1.02663	1.9458			1.43068			4.40311
SWI4	YDL161W		0.840485	5.5256						6.36608
SWI4	YDL164C		0.840485	4.97887	1.40479				0.704649	7.92879
SWI4	YDL191W		0.840485		1.6239		1.53037		0.729949	4.7247
SWI4	YDL192W		0.840485	5.18828			1.37257			7.40133
SWI4	YDL211C	1.20245	1.02663	8.24346						10.4725
SWI4	YDL219W		0.657711	5.5346						6.19231
SWI4	YDL220C		0.657711	4.25909						4.9168
SWI4	YDL227C	8.32883	0.351919	0.999039						9.67979
SWI4	YDR013W		0.840485	5.48231						6.3228
SWI4	YDR014W		0.840485	6.56526						7.40575
SWI4	YDR042C	0.220032	1.02663	5.57496						6.82162
SWI4	YDR097C		1.05039		1.41401	0.422679	1.43068		0.687202	5.00496
SWI4	YDR146C		0.840485		1.15362	1.52826	1.36611		0.725461	5.61394
SWI4	YDR171W				1.01583	1.97037	1.44975			4.43595
SWI4	YDR189W		0.048931	2.83536			1.07703		0.547975	4.5093
SWI4	YDR222W	0.158609	0.840485	4.6112						5.61029
SWI4	YDR224C	6.50779		0.662715	1.60868	1.59208			0.728946	11.1002
SWI4	YDR225W	6.50779		0.67794	1.60868	1.59208			0.731721	11.1182
SWI4	YDR226W	6.50779								6.50779
SWI4	YDR227W	2.20959					1.08057	0.883695		4.17386
SWI4	YDR228C			4.02184						4.02184
SWI4	YDR238C				1.56443	2.0021			0.690597	4.25713

TF	Target	Binding_0-2000	Motif_Score_SC	Other_Yeasts	Two-Hop_BE	Two-Hop_BP	Two-Hop_BY	Two-Hop_BG	Max_Membership	Sum
SWI4	YDR288W		0.254011	4.39651						4.65052
SWI4	YDR306C		0.840485	8.41979						9.26028
SWI4	YDR309C	7.58533	0.254011	0.0875695	1.85046					9.77737
SWI4	YDR441C	4.65104			1.16725					5.81829
SWI4	YDR451C	10.572			1.49461					12.0666
SWI4	YDR452W	10.572								10.572
SWI4	YDR500C	6.89391	0.840485		1.83526				0.728007	10.2977
SWI4	YDR501W	6.89391	0.840485	5.22336						12.9578
SWI4	YDR507C	4.87522	1.28647	0.218007	1.60388				0.730234	8.71381
SWI4	YDR508C	2.06535	0.577888				1.15258		0.560417	4.35623
SWI4	YDR524C	11.018	0.351919					1.38575		12.7557
SWI4	YDR524C-B	11.018	0.351919							11.3699
SWI4	YDR525W-A	11.018	0.494436	0.278077						11.7905
SWI4	YDR527W	11.018	0.351919	0.307838	0.951667				0.601383	13.2308
SWI4	YEL024W		0.553289		1.38877		1.41094		0.648131	4.00113
SWI4	YEL040W	9.0136	0.254011	4.44657	1.11005				0.729779	15.554
SWI4	YEL061C	0.0502829	0.254011	4.77565			0.788168		0.712379	6.58049
SWI4	YER001W	7.04692	0.840485	6.2197	1.81483					15.9219
SWI4	YER070W	4.76111	0.840485		1.67553				0.73244	8.00957
SWI4	YER078C	7.42637								7.42637
SWI4	YER079W	7.42637			0.94481					8.37118
SWI4	YER080W	7.42637								7.42637
SWI4	YER085C		1.02663	5.9153						6.94193
SWI4	YER086W		1.02663	5.69563	1.04938	2.0021			0.732572	10.5063
SWI4	YER098W				0.557055		1.37257	2.08859		4.01821
SWI4	YER104W		0.840485	6.04167						6.88216
SWI4	YER110C		0.351919		1.58352	1.97037			0.66972	4.57553
SWI4	YER111C	4.84537	1.02663			1.08759				6.95959
SWI4	YER112W	4.84537	1.02663			1.19853	0.828646			7.89918
SWI4	YER146W	2.39002				1.19853	1.01586		0.564274	5.16868
SWI4	YER148W				2.75002	0.748099	1.04725			4.54537
SWI4	YER151C			1.05886			1.37257	2.10522		4.53665
SWI4	YER178W	3.1854	0.254011			0.557567			0.696361	4.69334
SWI4	YFR017C	4.67694	0.840485							5.51743
SWI4	YFR019W	4.67694	0.840485	1.15683			0.971683			7.64594
SWI4	YGL036W	7.09504	0.29028							7.38532
SWI4	YGL038C	7.09504	0.29028	1.42612						8.81144
SWI4	YGL135W				1.86636		1.50387		0.731883	4.10211
SWI4	YGL147C	0.899296			1.65545		0.924422		0.732443	4.21161
SWI4	YGL179C	7.2397	0.840485	1.63084						9.71102
SWI4	YGL225W		0.254011	1.15591	1.91589				0.721268	4.04708
SWI4	YGL226W		1.02663	4.58652						5.61315
SWI4	YGR013W	6.28424		0.53793						6.82217
SWI4	YGR014W	5.217	0.254011						0.638611	6.10962
SWI4	YGR034W		0.494436		1.88129		0.892784		0.732578	4.00109
SWI4	YGR041W	6.4295	0.254011						0.730448	7.41396
SWI4	YGR109C	5.07596			1.20909		1.3202		0.695842	8.30109
SWI4	YGR140W	0.966667	0.840485	2.27828						4.08543
SWI4	YGR152C	4.58993	0.840485	5.48988	1.25881		1.37257	2.54407	0.731575	16.8273
SWI4	YGR153W	4.58993	0.840485	6.70895						12.1394
SWI4	YGR189C	8.94637	0.254011		1.60835				0.555406	11.3641
SWI4	YGR214W		0.494436		2.74786		1.04725		0.729189	5.01873
SWI4	YGR221C	9.14104	0.840485	3.41236	0.99695				0.681171	15.072
SWI4	YGR222W	9.14104	0.840485	3.54734						13.5289
SWI4	YHL001W				2.11424	0.698448	1.00202		0.715451	4.53016
SWI4	YHR061C	6.66954	0.254011		1.43783					8.36138
SWI4	YHR108W			3.67776			1.05154	0.963404		5.6927
SWI4	YHR149C	3.20367		0.189689					0.654063	4.04742
SWI4	YHR150W	3.20367	0.254011	0.186967					0.730017	4.37467
SWI4	YHR153C		1.02663	12.0876					0.626651	13.7409
SWI4	YHR154W		1.02663	5.853			1.14878		0.708026	8.73644
SWI4	YIL057C	2.76727	0.217509	1.50263	1.131					5.61841
SWI4	YIL066C		1.05039	0.519394	1.36107	1.12056			0.72473	4.77614
SWI4	YIL121W	2.24291			0.733152		1.5073			4.48336
SWI4	YIL123W	9.61734	1.02663	9.9361	1.68962			2.02923	0.723322	25.0222
SWI4	YIL132C		0.840485	4.51853	0.549545				0.660086	6.56865
SWI4	YIL133C		0.840485	3.97009	2.0514		1.21757		0.732757	8.8123
SWI4	YIL140W	0.347629	1.02663	3.99831	1.89935				0.721283	7.9932
SWI4	YIR019C	4.38028	0.494436						0.703408	5.57812
SWI4	YJL019W	0.349064	1.02663	3.76568		0.82414				5.96551
SWI4	YJL020C	0.349064	1.02663	4.25514						5.63083
SWI4	YJL157C				1.34973	1.97189			0.724267	4.04589
SWI4	YJL158C	5.63269	0.141805	0.104381	1.97162				0.705204	8.5557
SWI4	YJL177W			0.655441	1.98158		0.980761		0.732323	4.35011
SWI4	YJL183W	3.09682	0.254011		1.65889				0.636094	5.64582
SWI4	YJL186W	8.18792	1.02663	9.69973	1.20541					20.1197
SWI4	YJL187C	8.18792	1.02663	7.64522	1.69356					18.5533
SWI4	YJL194W	3.47192	1.71299				1.03303			6.21794
SWI4	YJL196C	3.47192	1.71299							5.18491
SWI4	YJR007W			2.33356	1.01493		0.927584		0.732639	5.00871
SWI4	YJR047C	1.23623	1.57675		1.05961		0.977079		0.723454	5.57312
SWI4	YJR048W	1.23623	1.57675		0.621808				0.716601	4.15139
SWI4	YJR054W	4.40461	1.02663	6.65585	0.928399				0.63696	13.6524
SWI4	YJR094W-A		0.185376	1.43089	1.72234		0.904938		0.732642	4.97619
SWI4	YJR145C	1.61419	0.254011		1.68482		0.900981		0.7321	5.1861
SWI4	YJR148W	4.89904	0.254011	3.75572						8.90877
SWI4	YJR149W	4.89904	0.254011		0.821045					5.9741
SWI4	YKL007W	3.89195	1.02663	11.284	0.713096					16.9157
SWI4	YKL008C	3.89195	1.02663	6.58844					0.534351	12.0414
SWI4	YKL078W			2.9834	0.628286				0.714533	4.32622
SWI4	YKL095W	6.07206								6.07206
SWI4	YKL096C-B	6.07206								6.07206
SWI4	YKL096W	6.07206		1.51973					0.64856	8.24035
SWI4	YKL096W-A	8.99816	0.141805	1.31632						10.4563
SWI4	YKL101W	2.09731	0.840485	6.77877	0.757236				0.590437	11.0642
SWI4	YKL103C	2.09731	0.840485	7.54283	1.53327	0.810582				12.8245
SWI4	YKL113C		1.8421		1.65713		0.788256		0.732334	5.01982
SWI4	YKL127W		0.840485	7.7964			1.2204		0.449042	10.3063
SWI4	YKL165C	0.642281	0.840485	2.50179	0.822253				0.60965	5.41646
SWI4	YKL180W	1.09208			1.86021		0.937326		0.731451	4.62107
SWI4	YKR011C	7.41124	0.494436	1.08358	1.50484				0.603888	11.098
SWI4	YKR013W	7.41124	0.494436		1.69413				0.724748	10.3246
SWI4	YKR042W	2.93363	0.048931		1.68681			2.02923	0.730131	7.42873
SWI4	YKR055W			4.52956			0.884412			5.41397
SWI4	YKR077W		1.02663	10.5879	1.23117				0.634381	13.4801
SWI4	YKR092C	2.34402		0.0895763	0.414599			0.859469	0.730187	4.43785
SWI4	YLL002W		0.254011	1.76313	1.47286				0.717763	4.20776
SWI4	YLL011W		1.22768		0.943619		1.11523		0.725983	4.01251
SWI4	YLL021W		0.840485	4.52289		0.557567			0.609788	6.53073
SWI4	YLL022C		0.840485	4.71538	0.99148					6.54734

TF	Target	Binding_0-2000	Motif_Score_SC	Other_Yeasts	Two-Hop_BE	Two-Hop_BP	Two-Hop_BY	Two-Hop_BG	Max_Membership	Sum
SWI4	YLR044C	2.37704	0.254011			1.97037			0.718408	5.31983
SWI4	YLR049C		1.02663	6.92028	1.47413				0.478182	9.89922
SWI4	YLR055C	4.71973	0.515569	0.864031			1.06939			7.16872
SWI4	YLR056W	4.71973	0.515569	1.08935	0.348024				0.715055	7.38773
SWI4	YLR084C	3.28554	0.254011	0.313321					0.542187	4.39506
SWI4	YLR121C	1.43479	0.119228	0.503563	1.87399		0.894437			4.82601
SWI4	YLR183C		0.840485	4.57873	1.24628				0.614766	7.28026
SWI4	YLR185W		1.02663		2.75098				0.727582	4.50519
SWI4	YLR196W		0.286405		1.16771	1.00617	1.08807		0.732226	4.28058
SWI4	YLR222C				1.24195	2.48623	0.92557		0.727036	5.38079
SWI4	YLR229C		0.254011	0.168268		2.37667	1.37257	2.01264		6.18416
SWI4	YLR233C		0.840485	4.3284						5.16889
SWI4	YLR234W		0.840485	3.63594	0.656418					5.13284
SWI4	YLR300W	7.58418	0.260505		1.01312				0.690512	9.54832
SWI4	YLR332W	9.17392	0.840485	6.26369	1.25115				0.56656	18.0958
SWI4	YLR342W	1.77508	0.840485		1.90552				0.726039	5.24712
SWI4	YLR372W		0.840485	3.63495	1.85459				0.732009	7.06203
SWI4	YLR438C-A	5.04991				1.14331	0.954138			7.14736
SWI4	YLR439W	5.04991	0.254011							5.30392
SWI4	YML024W	1.35129			1.64326		0.967468		0.730225	4.69224
SWI4	YML026C	1.35129			1.58842		0.819596		0.730894	4.4902
SWI4	YML027W	9.18158	0.393911		1.50628				0.730894	11.8127
SWI4	YML058W		0.362127	1.71837	1.26329	1.12056				4.46435
SWI4	YML100W	3.35728	0.00429395		1.00285					4.36442
SWI4	YMR006C	1.12464	1.35323		1.5178				0.617289	4.61296
SWI4	YMR016C	5.4303	0.254011						0.53308	6.21739
SWI4	YMR017W	5.4303	0.254011	0.0811535						5.76546
SWI4	YMR049C		0.254011		1.03511	1.10116	1.06541		0.700912	4.1566
SWI4	YMR070W	6.02899								6.02899
SWI4	YMR121C	0.858823	0.577888		1.57896		0.957041		0.729028	4.70174
SWI4	YMR135C	6.21281	0.834037							7.04685
SWI4	YMR136W	6.21281	0.834037							7.04685
SWI4	YMR144W	2.12267	1.02663	9.32445					0.509001	12.9828
SWI4	YMR145C	1.50711	0.254011		1.70607		1.20223		0.719494	5.38891
SWI4	YMR179W	4.36508	1.15555		1.32032					6.84095
SWI4	YMR199W	7.91469	0.254011		1.70057				0.574583	10.4439
SWI4	YMR214W		1.09985	0.478066	1.0996	2.0021				4.67962
SWI4	YMR304W				1.02799	1.26414	1.37257	2.08775		5.75245
SWI4	YMR305C	9.32598		0.324592	1.84357				0.73256	12.2267
SWI4	YMR306W	9.32598								9.32598
SWI4	YMR307W	11.4359	0.494436	1.77935	1.07575				0.732493	15.5179
SWI4	YMR308C	3.38136			0.5878	1.53412			0.732678	6.23596
SWI4	YNL015W		0.351919	3.17231	1.64753					5.17176
SWI4	YNL030W		0.330165	0.745399	1.56054		1.14382		0.731422	4.51135
SWI4	YNL031C		0.330165	0.866919	1.45459		1.03065		0.728083	4.41041
SWI4	YNL055C		0.254011	2.98683	1.04545					4.28629
SWI4	YNL082W		0.840485	4.21067	1.08043				0.555972	6.68756
SWI4	YNL098C	0.343144	0.254011				1.3179	2.38526		4.30032
SWI4	YNL175C		0.840485	0.361938	1.92441		1.06659		0.731372	4.9248
SWI4	YNL178W	3.19029			1.76587		0.958596		0.732428	6.64718
SWI4	YNL180C	3.19029					1.07662			4.26691
SWI4	YNL186W						1.37257	2.09783	0.732141	4.20254
SWI4	YNL204C			1.1344			1.37257	1.38595	0.525526	4.41845
SWI4	YNL231C	8.10581	0.840485	3.34883	1.54654				0.5356	14.3773
SWI4	YNL282W		0.840485	4.57986						5.42035
SWI4	YNL289W	11.5513	1.20026	1.55076						14.3023
SWI4	YNL291C	11.5513	1.20026							12.7516
SWI4	YNL298W	5.55813	0.577888							6.13602
SWI4	YNL299W	7.80684	1.02663						0.731234	9.5647
SWI4	YNL300W	7.80684	1.02663	9.69969	1.61992				0.72818	20.8813
SWI4	YNL301C	7.80684	1.02663		1.64875				0.732387	11.2146
SWI4	YNR041C	11.2175			1.09041				0.705083	13.013
SWI4	YNR044W	11.2175			0.670695				0.728447	12.6166
SWI4	YOL007C		0.840485	4.43584	1.93022				0.442852	7.6494
SWI4	YOL011W	5.30886	0.254011		1.76597				0.721274	8.05011
SWI4	YOL012C	5.30886	0.254011		1.45492		1.26553		0.681329	8.96465
SWI4	YOL034W		0.840485	3.22475						4.06523
SWI4	YOL082W			3.88135	1.09492					4.97627
SWI4	YOL090W		0.840485	2.64426	1.61528		1.43068		0.551439	7.08214
SWI4	YOL097C			2.14927	0.738657	0.422679	0.868583		0.685876	4.86507
SWI4	YOL113W	9.41419	0.254011	0.0106948						9.6789
SWI4	YOL114C	9.41419	0.254011							9.6682
SWI4	YOR101W		0.217509	0.0475158	0.731711		1.37257	2.54394	0.732502	5.64575
SWI4	YOR114W		0.840485	5.16879	1.41305					7.42233
SWI4	YOR194C		0.840485	10.855			1.05154			12.747
SWI4	YOR195W		0.840485	2.93312					0.684759	4.45836
SWI4	YOR210W				1.61953	2.43239	1.04669		0.727869	5.82648
SWI4	YOR246C	8.29749	1.02663	2.60184					0.713597	12.6396
SWI4	YOR247W	8.29749	1.02663	2.41411	1.58016				0.727092	14.0455
SWI4	YOR313C	5.43151	0.834037		0.721298				0.510975	7.49782
SWI4	YOR315W	5.43151	0.834037		0.721298				0.730125	7.71697
SWI4	YOR342C		0.840485	3.16183					0.481532	4.48385
SWI4	YOR344C	2.36633			1.09425				0.610278	4.07086
SWI4	YOR372C	6.99885	0.254011	0.176082						7.42894
SWI4	YOR373W	6.99885	0.141805	0.334569		0.557567				8.03279
SWI4	YOR378W	2.75416	0.254011				1.17555			4.18372
SWI4	YPL016W	1.61064	0.840485	1.58861	0.842909					4.88264
SWI4	YPL017C	1.61064	0.840485	2.44688						4.89801
SWI4	YPL024W	6.35659	0.254011							6.6106
SWI4	YPL026C	6.35659	0.254011	0.605381						7.21598
SWI4	YPL054W	7.29864	0.254011							7.55265
SWI4	YPL056C	7.29864	0.254011							7.55265
SWI4	YPL057C	7.29864	0.680054						0.486015	8.46471
SWI4	YPL081W	1.68806	0.254011		1.37358		1.01937		0.715401	5.05042
SWI4	YPL124W	1.73716	0.840485	4.3212						6.89884
SWI4	YPL126W	5.04314	1.02663	10.3026			1.03419		0.718564	18.1251
SWI4	YPL127C	5.04314	1.02663	7.17472	0.804099				0.715636	14.7642
SWI4	YPL128C	5.04314	1.02663	0.34116						6.41093
SWI4	YPL160W	0.750949			1.74753		0.990298		0.732455	4.22123
SWI4	YPL163C	9.98505	0.254011		1.70895				0.580565	12.5286
SWI4	YPL164C	9.98505	0.254011							10.2391
SWI4	YPL199C		0.254011	3.96749						4.2215
SWI4	YPL220W			0.259931	1.74028		1.50387		0.730958	4.23504
SWI4	YPL256C	0.323114	0.254011		1.93008	1.14331			0.725258	4.37577
SWI4	YPL266W	2.92253	0.217509		0.681528				0.732006	4.55357
SWI4	YPL267W	2.92253	0.217509	2.07649	1.13534				0.640037	6.99191
SWI4	YPR009W	3.78619	0.254011						0.527236	4.56744
SWI4	YPR098C		0.254011	2.53653	1.44323					4.23377
SWI4	YPR101W		0.254011	2.79793		1.28393				4.33587
SWI4	YPR119W	4.63896				1.26414	1.37257		0.725537	8.00121

TF	Target	Binding_0-2000	Motif_Score_SC	Other_Yeasts	Two-Hop_BE	Two-Hop_BP	Two-Hop_BY	Two-Hop_BG	Max_Membership	Sum
SWI5	TM(CAU)J3	6.48675								6.48675
SWI5	TS(AGA)L	13.0487								13.0487
SWI5	YAR020C				2.56649			2.95905		5.52554
SWI5	YBL038W					3.54208	1.93639			5.47847
SWI5	YBL072C	0.950641		1.40194	1.03537		0.923751			4.3117
SWI5	YBL112C				1.84628			2.36086	0.690999	4.89814
SWI5	YBL113C				1.74234			2.36086	0.522452	4.62565
SWI5	YBR041W	1.39966					1.69213	0.973354		4.06514
SWI5	YBR048W				2.25753		1.93878			4.19631
SWI5	YBR071W	2.98831			2.02922				0.707254	5.72478
SWI5	YBR088C				2.36279		1.91445			4.27724
SWI5	YBR158W	6.31867			3.24347				0.722592	10.2847
SWI5	YBR189W			0.430853	2.57628		1.8744			4.88153
SWI5	YBR191W	1.05991			1.14209		1.89203			4.09403
SWI5	YCL049C			5.07711	2.63516					7.71227
SWI5	YCR089W						2.06793	2.35398		4.42191
SWI5	YDL035C	4.49513								4.49513
SWI5	YDL061C				2.7148		1.84265			4.55745
SWI5	YDL117W	4.46712			2.48003					6.94715
SWI5	YDL119C	4.46712		0.00803108						4.47515
SWI5	YDL173W	5.48665			0.984477					6.47113
SWI5	YDL174C	5.48665								5.48665
SWI5	YDL178W	7.38564		0.509184						7.89482
SWI5	YDL179W	7.38564		0.639451	3.01209				0.690764	11.7279
SWI5	YDL201W				2.51021		1.84476			4.35497
SWI5	YDL223C	3.5251					2.05449	2.36086		7.94045
SWI5	YDL225W	7.87268								7.87268
SWI5	YDL226C	7.87268								7.87268
SWI5	YDL227C	10.0634								10.0634
SWI5	YDR055W	0.940465			2.70366				0.702188	4.34631
SWI5	YDR064W				2.51708		1.86157			4.37865
SWI5	YDR237W				2.61561		1.93025			4.54586
SWI5	YDR398W	2.05175		1.72135	0.356317					4.12942
SWI5	YDR418W	1.03677			1.06986		1.90249			4.00912
SWI5	YDR518W			2.23833			1.9042		0.70249	4.84502
SWI5	YDR545W	9.61584			1.10015		2.00422	1.19062	0.718352	14.6292
SWI5	YEL026W				2.67878		1.89614			4.57492
SWI5	YEL034W				2.90318		1.87109			4.77427
SWI5	YEL054C				2.54961		1.90249			4.4521
SWI5	YEL075C	0.590598			2.02045			2.36086	0.726713	5.69862
SWI5	YEL076C	0.250283			2.05916			2.36086	0.711355	5.38166
SWI5	YEL076C-A	0.250283			1.42772			2.36086		4.03886
SWI5	YEL077C	0.250283			2.06689			2.36086		4.67803
SWI5	YER007C-A				2.52281		1.83591			4.35872
SWI5	YER078C	8.51135			0.75217					9.26352
SWI5	YER079W	8.51135		0.0475158	0.46819					9.02706
SWI5	YER080W	8.51135		0.0497819						8.56113
SWI5	YER112W			0.646101	1.15413	3.73262	1.7914			7.32425
SWI5	YER124C	0.0578023		1.44615	2.60538				0.699157	4.80849
SWI5	YER146W			0.49124		3.73262	1.9521		0.564274	6.74023
SWI5	YER172C			1.96312		3.73262	1.76679			7.46253
SWI5	YER188C-A	9.99892								9.99892
SWI5	YER189W	9.99892			1.79922			2.36086	0.707991	14.867
SWI5	YER190W	9.99892			1.79922		1.99707	2.36086	0.727338	16.8834
SWI5	YFL039C			1.39129		1.96053	1.00328			4.3551
SWI5	YFL051C				0.981473			3.41613		4.3976
SWI5	YFL064C				2.09183			1.35848	0.70437	4.15468
SWI5	YFL065C				2.046			2.36086	0.729852	5.13671
SWI5	YFL066C				1.43867			2.36086	0.726262	4.52579
SWI5	YFL067W			1.29924	2.00876				0.725792	4.03379
SWI5	YFR017C	6.36034		0.0529859						6.41333
SWI5	YFR019W	6.36034					1.95348			8.31382
SWI5	YFR031C	2.45195		1.79781						4.24976
SWI5	YFR031C-A	2.45195		1.74814	0.800999					5.00109
SWI5	YGL028C	2.22889		2.27152	2.71369				0.710135	7.92424
SWI5	YGL056C	0.161774		6.3917						6.55347
SWI5	YGL061C			2.73293		1.82907				4.562
SWI5	YGL068W			1.34652	2.17986		1.83413			5.36051
SWI5	YGL106W			2.3501	0.647739		1.00328	1.0878		5.08892
SWI5	YGL108C	5.03334								5.03334
SWI5	YGL110C	5.03334								5.03334
SWI5	YGL135W	0.33803		0.436033	2.30504		1.75797			4.83707
SWI5	YGL173C					3.73262			0.526242	4.25886
SWI5	YGL227W	5.12741								5.12741
SWI5	YGR041W	2.61768		1.27254	2.79144					6.68166
SWI5	YGR044C			1.07703	2.73667				0.630415	4.44412
SWI5	YGR091W				0.356553	3.73262	1.92078			6.00995
SWI5	YGR189C	12.1821		0.450897	0.961494				0.555406	14.1499
SWI5	YGR213C			4.06409						4.06409
SWI5	YGR220C					3.54208	1.90629			5.44837
SWI5	YGR253C	5.61852					1.94685			7.56537
SWI5	YGR254W	5.61852		0.483284	0.809643					6.91145
SWI5	YGR296W				1.4464		2.00422	1.19062	0.730997	5.37224
SWI5	YHL033C				2.40209		1.90646			4.30855
SWI5	YHL049C				2.19673			1.41878	0.70533	4.32084
SWI5	YHL050C				1.97896			2.36086	0.732314	5.07213
SWI5	YHR008C	0.360321		5.75132						6.11164
SWI5	YHR138C	4.47081			1.13695					5.60776
SWI5	YHR141C				2.24195		1.92989			4.17184
SWI5	YHR147C				2.20862		1.92741			4.13603
SWI5	YHR218W				2.05827			1.41922	0.728318	4.20581
SWI5	YHR219W				1.91872			2.36086	0.731709	5.01129
SWI5	YIL009W	4.83939			2.62879				0.660192	8.12837
SWI5	YIL015W				0.671379		1.19663	2.34536	0.713218	4.92659
SWI5	YIL049W	3.95573			0.468038					4.42377
SWI5	YIL050W	3.95573		0.911671						4.8674
SWI5	YIL051C	3.95573		0.726045						4.68178
SWI5	YIL052C	3.95573			0.575159					4.53089
SWI5	YIL128W	4.72459								4.72459
SWI5	YIL129C	4.72459								4.72459
SWI5	YIL133C			0.411702	2.24742		1.89991			4.55903
SWI5	YIL169C			0.955128	2.30567			3.00567	0.719787	6.98625
SWI5	YIL177C				1.76722		1.99655		0.731868	4.49564
SWI5	YJL063C			0.114552	2.74308		1.91311			4.77074
SWI5	YJL076W	4.77324		0.222325					0.722416	5.71798
SWI5	YJL078C	4.77324		0.224711	0.665909		0.820821	2.30719	0.573929	9.3658
SWI5	YJL080C	3.61293			0.491979				0.728858	4.83377
SWI5	YJL124C			0.51822	0.379838	3.73262	1.88663			6.51731
SWI5	YJL148W	1.24694			3.01154					4.25848
SWI5	YJL151C				2.6245	1.5833				4.2078

TF	Target	Binding_0-2000	Motif_Score_SC	Other_Yeasts	Two-Hop_BE	Two-Hop_BP	Two-Hop_BY	Two-Hop_BG	Max_Membership	Sum
SWI5	YJL159W	15.8551		1.60409	3.238					20.6972
SWI5	YJL160C	15.8551		0.605081						16.4602
SWI5	YJL190C	1.60895			2.35105		1.9436			5.9036
SWI5	YJL225C				1.49396		1.99655		0.726156	4.21667
SWI5	YJR044C	6.48675								6.48675
SWI5	YJR094W-A				2.50771		1.83912			4.34683
SWI5	YJR123W				2.24175		1.89975			4.1415
SWI5	YJR143C	6.29342		2.43715					0.707973	9.43854
SWI5	YKL009W				2.54029		1.86031			4.4006
SWI5	YKL113C			1.26503	2.53455		1.86772			5.6673
SWI5	YKL142W	0.227638		2.73393	1.62282					4.58439
SWI5	YKL156W				2.31688		1.83476			4.15164
SWI5	YKL163W	15.8551			3.238					19.0931
SWI5	YKL164C	15.8551		1.06575	3.19421				0.601717	20.7168
SWI5	YKL180W				2.42068		1.82592			4.2466
SWI5	YKL185W	7.71213			3.12266				0.576942	11.4117
SWI5	YKL186C	7.71213								7.71213
SWI5	YKR013W			1.5189	2.32195				0.698386	4.53924
SWI5	YKR053C			4.34166						4.34166
SWI5	YLL021W			0.0636945	2.85211	0.648492			0.609788	4.17408
SWI5	YLL066C				1.88996			2.36086		4.25082
SWI5	YLL067C				1.9083			2.36086	0.727001	4.99616
SWI5	YLR010C	13.0487		3.21928						16.268
SWI5	YLR011W	13.0487								13.0487
SWI5	YLR012C	11.9699								11.9699
SWI5	YLR013W	11.9699								11.9699
SWI5	YLR048W				2.32019		1.89111			4.2113
SWI5	YLR049C	0.93219		1.21772	2.89485				0.722864	5.76762
SWI5	YLR079W	1.68515			2.45268				0.61359	4.75142
SWI5	YLR172C			0.145014	2.98261		1.86748			4.9951
SWI5	YLR193C	7.46121								7.46121
SWI5	YLR194C	7.46121			1.57603				0.635179	9.67242
SWI5	YLR207W	6.78748								6.78748
SWI5	YLR259C				2.91665			1.69711		4.61376
SWI5	YLR274W				2.45405		1.88838			4.34243
SWI5	YLR276C	1.72379			2.50975					4.23354
SWI5	YLR286C	3.5774		0.824239	2.51607				0.71213	7.62984
SWI5	YLR312W-A			2.39732	2.84212					5.23944
SWI5	YLR346C	4.82661			0.552467					5.37908
SWI5	YLR388W				2.59799		1.84265			4.44064
SWI5	YLR406C	4.23227								4.23227
SWI5	YLR407W	4.23227								4.23227
SWI5	YLR413W				2.40128		1.89541			4.29669
SWI5	YLR438C-A	14.4371					1.0052			15.4423
SWI5	YLR439W	14.4371								14.4371
SWI5	YLR450W			1.46067		1.6676	1.83258			4.96085
SWI5	YLR462W				2.10151			1.41893	0.731425	4.25187
SWI5	YLR464W				1.85526			2.36086	0.732463	4.94858
SWI5	YLR466W				1.3833		2.00433	2.36086	0.730891	6.47938
SWI5	YML024W				2.26521		1.84283			4.10804
SWI5	YML063W	0.262349			2.34826		1.92705			4.53766
SWI5	YML111W	3.84684		0.177391						4.02423
SWI5	YML133C				0.854112		2.00592	1.19062		4.05065
SWI5	YMR173W	6.97256			0.680945					7.65351
SWI5	YMR251W-A	5.34988			2.08862					7.4385
SWI5	YMR261C	2.91692		1.68219						4.59911
SWI5	YMR262W	2.91692			1.10898					4.0259
SWI5	YNL046W	0.384365		1.74733	2.37597				0.659218	5.16688
SWI5	YNL078W	3.00246		0.841228	3.013				0.721175	7.57786
SWI5	YNL147W					3.73262	0.956595			4.68921
SWI5	YNL164C			4.76024						4.76024
SWI5	YNL165W			4.45582						4.45582
SWI5	YNL175C			1.81748	3.21702					5.0345
SWI5	YNL178W				2.32279		1.91972			4.24251
SWI5	YNL185C				2.10672	3.54208				5.6488
SWI5	YNL186W			1.73487	2.2624		1.00328	1.5268		6.52735
SWI5	YNL192W	8.03942		1.47695	1.80772					11.3241
SWI5	YNL238W	4.19803								4.19803
SWI5	YNL241C	8.56269								8.56269
SWI5	YNL284C			2.7204		3.54208	1.88147			8.14395
SWI5	YNL327W	15.8551			2.94971		1.3095		0.636728	20.751
SWI5	YNL328C	15.8551								15.8551
SWI5	YNL329C	15.8551								15.8551
SWI5	YNL339C	1.59424			1.23421		2.00422	1.19062	0.650123	6.67341
SWI5	YNR004W			0.371468		3.67116				4.04263
SWI5	YNR067C				2.60704		1.96587		0.619425	5.19233
SWI5	YOL041C	0.0646216		1.40257	2.75938					4.22657
SWI5	YOR138C	5.07108								5.07108
SWI5	YOR140W	5.07108								5.07108
SWI5	YOR150W				2.29894		1.93994			4.23888
SWI5	YOR264W	1.16219			2.54104				0.654427	4.35766
SWI5	YPL050C	3.84742		1.07948	0.479582				0.64697	6.05345
SWI5	YPL093W				2.67989		1.89617			4.57606
SWI5	YPL157W	15.8551		0.292173						16.1473
SWI5	YPL158C	15.8551		0.313739	2.48685				0.625436	19.2811
SWI5	YPL160W				2.55856		1.9131		0.538487	5.01015
SWI5	YPL212C				2.32315		1.91693			4.24008
SWI5	YPR102C			2.09807	1.12959		1.88511			5.11277
SWI5	YPR128C			2.44309	2.45835		0.844679			5.74612
SWI5	YPR183W	2.36597				1.5833			0.604029	4.5533
SWI5	YPR187W				2.47415		1.93085			4.405
SWI5	YPR203W				1.70815			2.36086	0.73168	4.80069
SWI5	YPR204W				1.74266		2.00785	2.36086	0.722583	6.83395
SWI6	SNR40	15.8551	0.254011							16.1091
SWI6	TM(CAU)J3	4.0987								4.0987
SWI6	YAL040C	1.54706					1.9272		0.536372	4.01063
SWI6	YAR007C	0.50779		0.506209	2.22336	2.16018	1.36336			6.7609
SWI6	YBL003C			0.4414	2.4055		1.68755		0.730926	5.26538
SWI6	YBL016W			2.07705	2.68303		1.88582			6.6459
SWI6	YBL027W				2.0301		1.66404		0.731102	4.42524
SWI6	YBL063W		0.254011	1.85844	1.96012		1.48316	1.00196		6.55769
SWI6	YBL064C			4.97582	0.900566				0.73234	6.60873
SWI6	YBL067C						1.94048	2.96024		4.90072
SWI6	YBL072C				2.40279		1.61763		0.723202	4.74362
SWI6	YBL092W				1.69315		1.64952		0.732408	4.07508
SWI6	YBL099W				2.18723			2.11183	0.675512	4.97457
SWI6	YBR009C			0.539561	2.46843	0.373777	1.44662		0.729277	5.55767
SWI6	YBR010W		0.254011	0.564689	2.24358	0.388084	1.74221		0.72791	5.92048
SWI6	YBR031W				2.46449		1.63236		0.731372	4.82822
SWI6	YBR048W				2.44012		1.66608		0.732681	4.83888

TF	Target	Binding_0-2000	Motif_Score_SC	Other_Yeasts	Two-Hop_BE	Two-Hop_BP	Two-Hop_BY	Two-Hop_BG	Max_Membership	Sum
SWI6	YBR057C					2.22116	0.798221	1.42723		4.44661
SWI6	YBR070C	1.51038	0.254011	5.90192	1.86883				0.710669	10.2458
SWI6	YBR071W	1.51038	0.254011	7.023	1.86451				0.707254	11.3592
SWI6	YBR077C	13.7684		0.475838						14.2442
SWI6	YBR078W	13.7684	0.254011	0.00227775	2.31482				0.652121	16.9916
SWI6	YBR084C-A				2.50291		1.66404		0.731401	4.89835
SWI6	YBR088C	0.263267	0.241002	6.35499	2.14761	0.653559	1.66289		0.732264	12.0556
SWI6	YBR127C				2.08893			2.11272		4.20165
SWI6	YBR143C				2.21148		1.6675		0.731082	4.61006
SWI6	YBR162C	0.714119	0.241002	4.24705	2.37347			2.84603	0.727658	11.1493
SWI6	YBR162W-A	0.714119	0.241002	4.13431					0.728066	5.8175
SWI6	YBR163W	0.714119	0.241002	6.34918					0.540652	7.84495
SWI6	YBR189W				2.18802		1.659		0.728515	4.57553
SWI6	YBR191W				2.41393		1.58774		0.725883	4.72755
SWI6	YBR202W		0.254011	2.30431			1.61398			4.1723
SWI6	YBR265W				1.61284		1.97898		0.678143	4.26996
SWI6	YBR267W		0.254011		1.77125		1.49276		0.728902	4.24692
SWI6	YBR278W		0.241002	3.78072						4.02172
SWI6	YCL024W		0.241002	1.23569	1.96288				0.678093	4.11766
SWI6	YCL059C				1.40721	0.863253	1.58166		0.730058	4.58218
SWI6	YCR027C						1.94048	3.41573		5.35621
SWI6	YCR031C				2.04919		1.66721		0.730119	4.44652
SWI6	YCR032W			1.03245			1.43778	1.76728		4.23751
SWI6	YCR065W	3.37881	0.241002	4.20687	1.68679				0.560804	10.0743
SWI6	YDL003W	3.87928	0.254011	4.37787	2.34766	0.583661			0.725901	12.1684
SWI6	YDL014W		0.254011	1.47654	1.68945		1.64285		0.727787	5.79064
SWI6	YDL017W	4.85093		0.324592						5.17552
SWI6	YDL018C	4.85093		0.331556	1.75111		1.80434			8.73794
SWI6	YDL055C	5.11478	0.241002	5.82942	1.05152				0.731108	12.9678
SWI6	YDL061C				1.74371		1.56011		0.732364	4.03618
SWI6	YDL083C				2.32142		1.6434		0.729148	4.69397
SWI6	YDL099W	3.00764	0.254011				0.907291			4.16894
SWI6	YDL101C	3.00764			0.404769				0.656947	4.06936
SWI6	YDL124W	1.16625			1.10887		1.99692		0.714404	4.98644
SWI6	YDL127W	7.14698	0.254011	3.55202	1.59929				0.661532	13.2138
SWI6	YDL153C				2.49765		1.38449		0.732155	4.6143
SWI6	YDL156W			0.65286	1.54042		1.49276		0.610166	4.29621
SWI6	YDL161W		0.241002	4.86622						5.10722
SWI6	YDL164C		0.241002	4.2942	1.97392		1.20775		0.704649	8.42152
SWI6	YDL191W		0.254011		1.94629		1.25386		0.729949	4.18411
SWI6	YDL192W		0.241002	9.06511			1.94048			11.2466
SWI6	YDL197C	0.193584			1.23837		0.916003	1.30483	0.610551	4.26334
SWI6	YDL211C	1.86225	0.241002	9.27017						11.3734
SWI6	YDL219W			6.78967						6.78967
SWI6	YDL220C			4.8278						4.8278
SWI6	YDL227C	10.9191	0.254011	0.087376						11.2605
SWI6	YDR012W				2.29443		1.63236		0.732434	4.65922
SWI6	YDR014W		0.241002	2.99672			0.889478			4.1272
SWI6	YDR025W				2.38051		1.66608		0.732106	4.7787
SWI6	YDR064W				2.26367		1.64285		0.732202	4.63872
SWI6	YDR069C						1.94048	2.9606		4.90108
SWI6	YDR075W		0.254011	2.03986	2.07772				0.727805	5.0994
SWI6	YDR097C				1.99764		1.81244		0.687202	4.49728
SWI6	YDR099W		0.254011			2.74229	1.10208			4.09838
SWI6	YDR101C		0.254011		3.01766				0.730741	4.00241
SWI6	YDR113C	2.14185			1.89714					4.03899
SWI6	YDR144C				1.94571		1.4001		0.716231	4.06204
SWI6	YDR146C		0.241002		1.88789	1.32035	1.87141		0.725461	6.04611
SWI6	YDR189W			5.5188			1.37984		0.547975	7.44661
SWI6	YDR202C			2.65407	1.46697					4.12104
SWI6	YDR222W		0.241002	5.96542						6.20642
SWI6	YDR224C	1.99475		3.25699	0.686796	0.373777			0.728946	7.04126
SWI6	YDR225W	1.99475		3.79494	1.47305	0.373777			0.731721	8.36824
SWI6	YDR249C		0.254011	4.05628						4.31029
SWI6	YDR251W		0.241002	2.37873	1.48157					4.1013
SWI6	YDR263C	12.006			0.916067		1.33914		0.727391	14.9886
SWI6	YDR264C	1.76333					1.47122	2.94994		6.18449
SWI6	YDR309C	10.7685	0.254011		2.51467					13.5372
SWI6	YDR324C	1.2647			1.27244		1.28717		0.732666	4.55698
SWI6	YDR358W				1.51559		1.10294	1.34833	0.727104	4.69396
SWI6	YDR399W		0.254011	3.06109					0.725235	4.04034
SWI6	YDR418W				2.47652		1.65373		0.71499	4.84524
SWI6	YDR447C				1.77721		1.62992		0.723762	4.13089
SWI6	YDR448W						1.35523	3.34929		4.70452
SWI6	YDR450W		0.254011		1.94775		1.61856		0.72879	4.54911
SWI6	YDR451C	14.1664			2.58361		1.49276			18.2428
SWI6	YDR452W	14.1664								14.1664
SWI6	YDR454C			0.0993078	2.25068		1.18357		0.700034	4.23359
SWI6	YDR500C	9.92917	0.241002		2.36209		1.26259		0.724669	14.5195
SWI6	YDR501W	9.92917	0.241002	5.71812	1.26621				0.732428	17.8869
SWI6	YDR507C	2.98541			1.39799				0.730234	5.11363
SWI6	YDR524C	15.8551					1.10208			16.9572
SWI6	YDR524C-B	15.8551								15.8551
SWI6	YDR525W-A	15.8551								15.8551
SWI6	YDR527W	15.8551							0.601383	16.4565
SWI6	YEL026W				1.91452		1.65073		0.732578	4.29783
SWI6	YEL034W				1.85654		1.65668		0.705969	4.21919
SWI6	YEL040W	5.70374	0.254011	2.04195	1.74676				0.729779	10.4762
SWI6	YEL054C				2.24007		1.65373		0.729125	4.62292
SWI6	YEL061C	0.718017	0.254011	7.24162			0.953257		0.712379	9.87928
SWI6	YER001W	9.85936	0.241002	3.05966	2.47904					15.6391
SWI6	YER012W				1.66683	2.35071	1.62847			5.64601
SWI6	YER029C		0.241002		0.935801	2.13251	0.778386		0.703549	4.79125
SWI6	YER056C-A		0.254011		2.26531		1.23521		0.727253	4.48178
SWI6	YER070W	9.49219	0.254011	0.632307	1.80636				0.73244	12.9173
SWI6	YER074W				2.36881		1.25641		0.7296	4.35482
SWI6	YER078C	9.97601								9.97601
SWI6	YER079W	9.97601			0.815669					10.7917
SWI6	YER080W	9.97601								9.97601
SWI6	YER081W		0.254011		2.32804	3.2218				5.80385
SWI6	YER082C				1.25491	0.863253	1.39086		0.73215	4.24117
SWI6	YER085C		0.241002	4.23063						4.47163
SWI6	YER086W		0.241002	3.22603	2.0956	2.16018			0.732572	8.45538
SWI6	YER093C-A	9.31793								9.31793
SWI6	YER094C	9.31793		0.173548	1.42332		1.63684			12.5516
SWI6	YER095W	9.31793		0.186967	0.902009		1.65473		0.643734	12.7054
SWI6	YER098W				1.52349		1.94048	2.96024		6.42421
SWI6	YER102W				2.32865		1.61763		0.730565	4.67685
SWI6	YER104W		0.241002	5.23575						5.47675
SWI6	YER110C		0.254011		2.47568	2.16018			0.66972	5.55959

TF	Target	Binding_0-2000	Motif_Score_SC	Other_Yeasts	Two-Hop_BE	Two-Hop_BP	Two-Hop_BY	Two-Hop_BG	Max_Membership	Sum
SWI6	YER111C	6.25148	0.254011			2.18772				8.69321
SWI6	YER112W	6.25148	0.254011			2.13251	1.27016			9.90816
SWI6	YER146W	1.17141				2.13251	1.64219		0.564274	5.51038
SWI6	YER151C			0.838583			1.94048	2.97684		5.7559
SWI6	YFL038C	0.648933	0.254011		1.36584			3.41505		5.68383
SWI6	YFL039C	0.73777	0.254011		2.93226		1.1025			5.02654
SWI6	YFR017C	6.75823	0.254011							7.01224
SWI6	YFR019W	6.75823	0.254011	0.538867			1.66635			9.21746
SWI6	YFR027W		0.241002	2.12348	0.521801	2.25464				5.14092
SWI6	YGL011C				1.8622	2.16018	1.66143			5.68381
SWI6	YGL030W				2.35931		1.53788		0.713324	4.61051
SWI6	YGL036W	9.54392			1.37715					10.9211
SWI6	YGL038C	9.54392		0.184576						9.7285
SWI6	YGL076C				2.36857		1.64156		0.732293	4.74242
SWI6	YGL093W		0.241002	6.49368			0.920809			7.65549
SWI6	YGL094C		0.241002	5.08491						5.32591
SWI6	YGL097W		0.254011		2.96174		1.10208		0.732003	5.04983
SWI6	YGL103W		0.254011		1.9084		1.40156		0.723774	4.28775
SWI6	YGL106W						1.42865	2.66086		4.08951
SWI6	YGL116W			0.0767158	1.15017	2.18772	1.39489			4.8095
SWI6	YGL135W				2.39321		1.61197		0.731883	4.73706
SWI6	YGL147C				2.18228		1.65867		0.732443	4.57339
SWI6	YGL179C	6.46985	0.241002	3.23144	1.29766					11.24
SWI6	YGL225W		0.254011		2.23827		1.10208		0.721268	4.31563
SWI6	YGR013W	6.06766								6.06766
SWI6	YGR014W	6.82919	0.254011						0.705133	7.78833
SWI6	YGR034W		0.254011		2.40814		1.6479		0.732578	5.04263
SWI6	YGR041W	6.68084	0.254011						0.730448	7.6653
SWI6	YGR085C	0.170009	0.254011		1.79622		1.54169		0.730832	4.49276
SWI6	YGR109C	12.0418		0.168202	1.94336		1.74221		0.695842	16.5914
SWI6	YGR110W	5.84134	0.254011	0.0752491						6.1706
SWI6	YGR152C	1.49519	0.241002	2.20097	1.92302		1.94048	3.41571	0.731575	11.9479
SWI6	YGR153W	1.49519	0.241002	3.60813						5.34432
SWI6	YGR180C	2.84307	0.254011	0.47919	1.29489		1.04545		0.718206	6.63482
SWI6	YGR185C				1.96741		1.65104		0.67325	4.2917
SWI6	YGR189C	8.55133	0.254011		2.56409				0.555406	11.9248
SWI6	YGR214W				2.43422		1.64379		0.729189	4.8072
SWI6	YGR221C	11.4977	0.241002	4.72188	1.70912				0.681171	18.8509
SWI6	YGR222W	11.4977	0.241002	3.31742						15.0561
SWI6	YGR233C	0.15998					0.919431	3.40961		4.48902
SWI6	YGR234W	0.15998			1.36296	2.16018	1.02527			4.70839
SWI6	YGR279C	4.90226		1.77353						6.67579
SWI6	YHL001W				2.99339		1.30483		0.715451	5.01367
SWI6	YHL033C				2.20466		1.65215		0.730565	4.58738
SWI6	YHR021C				1.9247		1.64432		0.729462	4.29848
SWI6	YHR061C	7.67145	0.254011		2.183					10.1085
SWI6	YHR064C			2.95806	2.03351				0.707454	5.69902
SWI6	YHR104W				1.81654		1.96294		0.731645	4.51112
SWI6	YHR108W			1.94028			1.35435	1.33845		4.63308
SWI6	YHR110W		0.254011	2.43833	0.43211		1.10208		0.623362	4.84989
SWI6	YHR126C		0.241002	4.34166						4.58266
SWI6	YHR127W		0.241002	3.95182	1.05028				0.462754	5.70586
SWI6	YHR141C		0.254011		1.82355		1.648		0.732431	4.45799
SWI6	YHR149C	6.42891							0.654063	7.08297
SWI6	YHR150W	6.42891	0.254011							6.68292
SWI6	YHR153C		0.241002	11.1378					0.626651	12.0055
SWI6	YHR154W		0.241002	4.9835	0.539526		1.45158		0.708026	7.92363
SWI6	YHR169W		0.254011		1.8842	1.61623			0.730929	4.48537
SWI6	YHR193C	2.34794					1.54145		0.719576	4.60897
SWI6	YHR203C				2.4273		1.61578		0.729712	4.77279
SWI6	YIL026C	2.77224	0.254011	0.384363	0.843619	0.882514				5.13675
SWI6	YIL066C		0.254011	0.297199	2.02528	2.39774			0.72473	5.69896
SWI6	YIL112W				2.10835			2.18647		4.29482
SWI6	YIL115C			0.899652	3.25906				0.659919	4.81863
SWI6	YIL123W	13.2653	0.254011	6.01004	2.42387				0.723322	22.6765
SWI6	YIL132C		0.241002	4.53747	0.691403				0.660086	6.12996
SWI6	YIL133C		0.241002	1.21673	2.37378		2.06135		0.732757	6.62562
SWI6	YIL140W	0.210657	0.241002	5.80201	2.2121				0.721283	9.18705
SWI6	YIL148W		0.254011		2.15508		1.23136		0.72265	4.3631
SWI6	YIL169C		0.254011	3.19543	1.2436					4.69304
SWI6	YJL005W						1.88582	2.65738		4.5432
SWI6	YJL019W		0.241002	4.28304						4.52404
SWI6	YJL092W	3.27125					1.57951		0.592617	5.44338
SWI6	YJL093C	3.27125		0.89194						4.16319
SWI6	YJL115W	2.04775		0.690456	1.46056	0.412253				4.61102
SWI6	YJL124C				1.84981	2.13251	0.979274		0.515411	5.47701
SWI6	YJL157C				0.820498	3.92827			0.724267	5.47304
SWI6	YJL158C	8.70513			2.71681				0.705204	12.1271
SWI6	YJL177W				2.30396		1.55402		0.732323	4.5903
SWI6	YJL183W	3.87753	0.254011		2.53806				0.636094	7.3057
SWI6	YJL185C	3.87753	0.254011							4.13154
SWI6	YJL186W	11.6637	0.254011	4.45354	0.529293					16.9005
SWI6	YJL187C	11.6637	0.241002	5.64821	2.13604	1.40621				21.0952
SWI6	YJL190C				2.07145		1.66858		0.731944	4.47197
SWI6	YJL191W				2.40824		1.66721		0.7041	4.77955
SWI6	YJL194W	5.62816			0.651213		1.65428			7.93365
SWI6	YJL196C	5.62816								5.62816
SWI6	YJL197W						1.94048	2.96182		4.9023
SWI6	YJR007W			1.9806	1.26544		1.63323		0.732639	5.61191
SWI6	YJR044C	4.0987		0.355393						4.45409
SWI6	YJR054W	4.12819	0.241002	4.50022	0.799259				0.63696	10.3056
SWI6	YJR090C					3.92827	1.66732			5.59559
SWI6	YJR091C	1.32011		2.01897		3.03462	1.10208			7.47578
SWI6	YJR094W-A			0.78993	2.24917		1.65763		0.732642	5.42937
SWI6	YJR123W		0.254011		2.44044		1.65258		0.726798	5.07383
SWI6	YJR145C	1.24809	0.254011		2.19654		1.61578		0.7321	6.04652
SWI6	YJR148W	4.35547	0.254011	0.967925		0.357452				5.93486
SWI6	YJR149W	4.35547	0.254011		1.34186					5.95134
SWI6	YKL006W				2.33806		1.30483		0.731492	4.37438
SWI6	YKL007W	1.84138	0.241002	6.96935	1.52808					10.5798
SWI6	YKL008C	1.84138	0.241002	3.73165					0.534351	6.34838
SWI6	YKL009W				1.7277		1.63388		0.732742	4.09432
SWI6	YKL081W				1.85133		1.54101		0.716437	4.10878
SWI6	YKL096W-A	10.5662		1.48124						12.0474
SWI6	YKL101W		0.254011	5.67997	1.46724				0.590437	7.99166
SWI6	YKL103C		0.254011	7.87146	1.86379				0.71049	10.6998
SWI6	YKL112W	5.92612	0.254011							6.18013
SWI6	YKL113C	5.92612	0.254011		2.32833		1.34406		0.732334	10.5849
SWI6	YKL127W		0.254011	7.27409			2.06418		0.449042	10.0413
SWI6	YKL129C		0.254011		1.99916		0.874875	1.24242		4.37047

TF	Target	Binding_0-2000	Motif_Score_SC	Other_Yeasts	Two-Hop_BE	Two-Hop_BP	Two-Hop_BY	Two-Hop_BG	Max_Membership	Sum
SWI6	YKL150W			1.25201	1.26199		1.97721			4.49121
SWI6	YKL156W		0.254011		1.67831		1.64432		0.730947	4.30759
SWI6	YKL165C		0.241002	2.51239	0.693113				0.60965	4.05615
SWI6	YKL180W				2.18259		1.57576		0.731451	4.4898
SWI6	YKL190W						1.42905	2.66086		4.08991
SWI6	YKR011C	6.77905	0.254011		1.46873				0.726144	9.22794
SWI6	YKR013W	6.77905	0.254011		2.35835		1.10208		0.724748	11.2182
SWI6	YKR042W	4.02934			2.84419				0.730131	7.60366
SWI6	YKR077W		0.241002	5.184	2.08122				0.634381	8.1406
SWI6	YLL002W		0.254011	1.85467	2.13707				0.717763	4.96351
SWI6	YLL021W	0.671013	0.241002	6.63691	0.714739				0.609788	8.87345
SWI6	YLL022C	0.671013	0.241002	7.41424	1.51831					9.84456
SWI6	YLL039C				1.16265	2.18772	1.10208			4.45245
SWI6	YLL045C				1.84208		1.65215		0.732701	4.22693
SWI6	YLL058W		0.254011		1.41648		1.98767		0.696678	4.35484
SWI6	YLR029C				2.10422		1.64285		0.729166	4.47624
SWI6	YLR048W		0.254011		2.45076		1.63315		0.729935	5.06786
SWI6	YLR049C	0.238159	0.254011	3.69092	1.67188				0.478182	6.33315
SWI6	YLR055C	3.27449					1.37219			4.64668
SWI6	YLR056W	3.27449		0.174052					0.715055	4.1636
SWI6	YLR060W		0.254011		2.22629		1.3534		0.719857	4.55356
SWI6	YLR089C				1.95104		1.99478		0.585074	4.53089
SWI6	YLR103C	4.30586			1.71401				0.653051	6.67292
SWI6	YLR104W	4.30586								4.30586
SWI6	YLR175W		0.241002	1.82668	1.30507				0.732258	4.10501
SWI6	YLR178C				2.64951		0.853112		0.724496	4.22712
SWI6	YLR183C		0.254011	3.7099	1.91049				0.614766	6.48917
SWI6	YLR185W		0.254011		2.05433		1.26259		0.727582	4.29851
SWI6	YLR196W				1.88088	0.863253	1.43185		0.732226	4.90821
SWI6	YLR222C				1.76878	1.47505	1.22837		0.727036	5.19924
SWI6	YLR229C		0.254011			2.74229	1.94048	2.88426		7.82104
SWI6	YLR262C				0.931856		0.899052	3.24263		5.07354
SWI6	YLR264W				1.8173	1.0786	1.55415		0.726769	5.17682
SWI6	YLR300W	7.24623							0.690512	7.93674
SWI6	YLR303W	3.18084			0.960913					4.14175
SWI6	YLR332W	4.83877	0.254011	4.53399	2.71919				0.56656	12.9125
SWI6	YLR340W				2.04187		1.60747		0.730078	4.37942
SWI6	YLR342W	1.97561	0.241002		2.22788				0.726039	5.17053
SWI6	YLR344W				2.3328		1.6479		0.730782	4.71148
SWI6	YLR367W	0.451494			1.4694		1.66858		0.729524	4.319
SWI6	YLR372W		0.241002	5.33238	2.28932				0.732009	8.59471
SWI6	YLR381W		0.254011	4.18183						4.43584
SWI6	YLR388W		0.254011		2.36041		1.56011		0.726625	4.90116
SWI6	YLR436C	2.91282	0.254011		1.11751					4.28434
SWI6	YLR438C-A	8.50962				1.5229	1.66434			11.6969
SWI6	YLR439W	8.50962	0.254011							8.76363
SWI6	YLR441C				2.08211		1.65976		0.73224	4.47411
SWI6	YML024W	0.0933167			2.26329		1.62992		0.730225	4.71675
SWI6	YML026C	0.0933167			2.20843		1.61856		0.730894	4.6512
SWI6	YML027W	13.5285			2.67014				0.730894	16.9295
SWI6	YML058W		0.254011	2.88915	1.1826	2.39774				6.7235
SWI6	YML063W				2.2751		1.65976		0.730694	4.66555
SWI6	YML072C						1.94048	2.99112		4.9316
SWI6	YML085C		0.241002	3.13539					0.691535	4.06793
SWI6	YML100W	4.25902			2.02953				0.71945	7.008
SWI6	YML101C	4.25902								4.25902
SWI6	YMR012W		0.254011		0.887104	2.16018	1.37533		0.713345	5.38997
SWI6	YMR016C	4.85241	0.254011						0.53308	5.6395
SWI6	YMR017W	4.85241	0.254011	1.3489			0.925058			7.38038
SWI6	YMR033W			4.04829					0.515537	4.56383
SWI6	YMR047C			0.345937		2.45485	1.41484		0.538214	4.75384
SWI6	YMR049C		0.254011		1.82806	1.00993	1.44394		0.700912	5.23685
SWI6	YMR070W	4.53413								4.53413
SWI6	YMR076C	8.96207	0.254011						0.456209	9.67229
SWI6	YMR078C	0.53099			1.61036	0.469122	1.65977			4.27024
SWI6	YMR105C		0.254011		1.74347	3.14676			0.692583	5.83682
SWI6	YMR121C	2.92021			2.0126		1.64285		0.729028	7.30469
SWI6	YMR135C	7.76466								7.76466
SWI6	YMR136W	7.76466								7.76466
SWI6	YMR143W				2.2879		1.6434		0.730087	4.66139
SWI6	YMR144W	5.16829	0.241002	5.00566					0.509001	10.924
SWI6	YMR145C		0.254011		2.02842		2.04601		0.719494	5.04793
SWI6	YMR179W	8.90286	0.254011		2.43309					11.59
SWI6	YMR199W	15.1617	0.254011		2.36478				0.574583	18.3551
SWI6	YMR202W	3.67924			2.43568				0.707254	6.82217
SWI6	YMR215W	3.39447		0.992833	2.44752				0.607875	7.4427
SWI6	YMR223W						1.94048	2.95257		4.89305
SWI6	YMR239C		0.254011		1.76537		1.25076		0.730322	4.00046
SWI6	YMR260C				1.73214		1.55953		0.730439	4.02211
SWI6	YMR262W	2.0649		0.430853	1.40817				0.729072	4.633
SWI6	YMR304W				1.5488	2.20244	1.94048	2.95937		8.65109
SWI6	YMR305C	11.4104		0.855646	2.50778				0.73256	15.5064
SWI6	YMR306W	11.4104		0.757089						12.1675
SWI6	YMR307W	13.0141	0.254011		2.1043				0.732493	16.1049
SWI6	YMR308C	1.10457			1.91198	1.24641			0.732678	4.99564
SWI6	YNL030W			0.609827	1.88686		1.44662		0.731422	4.67473
SWI6	YNL067W				1.8878		1.66014		0.731789	4.27973
SWI6	YNL069C				2.27628		2.05863		0.731454	5.06636
SWI6	YNL072W		0.241002	5.52388	0.630898		1.64291		0.730518	8.76921
SWI6	YNL082W		0.241002	5.5468	2.12247				0.555972	8.46624
SWI6	YNL096C				2.41746		1.23692		0.732584	4.38696
SWI6	YNL098C	0.585092	0.254011				1.88582	3.2569		5.98182
SWI6	YNL134C		0.254011		1.71769		2.0161		0.730175	4.71798
SWI6	YNL162W				1.88225		1.648		0.7309	4.26115
SWI6	YNL175C		0.254011	0.160999	2.91252		1.4541		0.731372	5.513
SWI6	YNL178W	0.752181			2.2927		1.6445		0.69735	5.38673
SWI6	YNL186W				0.873562		1.94048	2.96947	0.732141	6.51565
SWI6	YNL231C	4.94547	0.241002	2.02748	2.53465				0.5356	10.2842
SWI6	YNL241C	8.1632			1.70105					9.86425
SWI6	YNL244C		0.254011	4.07935			1.37309		0.669201	6.37565
SWI6	YNL272C			4.35429						4.35429
SWI6	YNL273W	3.52037		0.132463	1.62141					5.27424
SWI6	YNL274C	3.52037		0.210644	1.95291				0.730419	6.41434
SWI6	YNL282W		0.254011	4.90691						5.16092
SWI6	YNL289W	15.8551	0.254011	0.582502						16.6916
SWI6	YNL291C	15.8551	0.254011							16.1091
SWI6	YNL298W	7.60788								7.60788
SWI6	YNL299W	10.3859	0.254011						0.731234	11.3711
SWI6	YNL300W	10.3859	0.254011	6.34838	2.22041				0.72818	19.9369
SWI6	YNL301C	10.3859	0.254011		2.26879				0.72319	13.6319

TF	Target	Binding_0-2000	Motif_Score_SC	Other_Yeasts	Two-Hop_BE	Two-Hop_BP	Two-Hop_BY	Two-Hop_BG	Max_Membership	Sum
SWI6	YNL302C				2.07279		1.63236		0.730777	4.43593
SWI6	YNL310C		0.241002	4.87021						5.11121
SWI6	YNL311C		0.241002	6.16337						6.40437
SWI6	YNR018W	6.21019		0.147266						6.35746
SWI6	YNR019W	6.21019			0.532318					6.74251
SWI6	YNR041C	7.47169			1.97839				0.705083	10.1552
SWI6	YNR044W	7.47169			1.2452				0.728447	9.44534
SWI6	YOL004W				2.12588	0.807139	1.10208			4.0351
SWI6	YOL007C		0.254011	2.42203	2.59444				0.442852	5.71333
SWI6	YOL011W	1.1711	0.254011	0.170668	1.75752				0.721274	4.07457
SWI6	YOL012C	1.1711	0.254011		2.2001		1.68755			5.31276
SWI6	YOL039W			1.62765	2.0271		1.27142		0.732698	5.65887
SWI6	YOL040C			1.64854	2.1855		1.66927		0.728934	6.23224
SWI6	YOL041C				2.03812		1.49276		0.731812	4.26269
SWI6	YOL082W			2.14218	1.61574				0.700381	4.4583
SWI6	YOL090W		0.241002	6.32145	2.09668		1.81244		0.551439	11.023
SWI6	YOL097C			1.72916	1.26549		1.55078		0.685876	5.23131
SWI6	YOL113W	7.13119	0.254011							7.3852
SWI6	YOL114C	7.13119	0.254011							7.3852
SWI6	YOL121C				1.78441		1.63236		0.722269	4.13904
SWI6	YOL124C			0.590982	1.94165		1.41605		0.727145	4.67583
SWI6	YOL127W				2.17107		1.60968		0.717543	4.49829
SWI6	YOL133W		0.254011	0.127609		2.77063	1.40938			4.56163
SWI6	YOR074C	5.01471			1.56962					6.58433
SWI6	YOR075W	5.01471								5.01471
SWI6	YOR086C						1.94048	2.98552		4.926
SWI6	YOR096W				2.37254		1.23692		0.730703	4.34016
SWI6	YOR101W		0.241002				1.94048	3.41558	0.732502	6.32956
SWI6	YOR114W	0.107813	0.254011	7.09417	0.388772				0.728468	8.57323
SWI6	YOR120W				1.96557		2.02911		0.609175	4.60386
SWI6	YOR145C				1.63267		1.64033		0.732496	4.0055
SWI6	YOR157C				1.55759	1.16088	1.66092			4.37939
SWI6	YOR167C				2.08641	1.0786	1.55415		0.720385	5.43954
SWI6	YOR194C		0.241002	4.04907			1.35435			5.64442
SWI6	YOR210W				1.16516	3.7382	1.40305		0.727869	7.03428
SWI6	YOR234C	1.5117	0.254011		2.02677				0.726115	4.5186
SWI6	YOR246C	15.4841	0.254011	1.42526					0.713597	17.877
SWI6	YOR247W	15.4841	0.254011	2.00073	2.31443				0.727092	20.7804
SWI6	YOR313C	6.12596			0.863156				0.510975	7.50009
SWI6	YOR315W	6.12596			0.914689				0.730125	7.77077
SWI6	YOR326W						1.38795	2.25045	0.709707	4.34811
SWI6	YOR342C	0.462943	0.241002	5.67933	1.71214				0.481532	8.57695
SWI6	YOR344C	3.27572			1.01356				0.610278	4.89956
SWI6	YOR369C				2.3225		1.64948		0.732475	4.70445
SWI6	YOR372C	5.93307	0.254011							6.18708
SWI6	YOR373W	5.93307								5.93307
SWI6	YPL031C					4.11544				4.11544
SWI6	YPL043W		0.254011		1.96619		1.44815		0.730847	4.3992
SWI6	YPL049C		0.254011			3.92827	1.89131			6.07359
SWI6	YPL054W	6.4983	0.254011	0.259931						7.01224
SWI6	YPL056C	6.4983	0.254011							6.75231
SWI6	YPL057C	6.4983	0.254011						0.486015	7.23833
SWI6	YPL079W				2.09697		1.58774		0.729993	4.4147
SWI6	YPL081W	0.893352	0.254011		1.29823		1.659		0.715401	4.81999
SWI6	YPL090C				2.14939		1.62408		0.732003	4.50547
SWI6	YPL093W				2.15188		1.64818		0.7308	4.53086
SWI6	YPL124W		0.241002	5.06483						5.30583
SWI6	YPL126W	4.51373	0.241002	3.21252			0.940227		0.718564	9.62604
SWI6	YPL127C	4.51373	0.241002	3.1442					0.715636	8.61457
SWI6	YPL128C	4.51373	0.241002							4.75473
SWI6	YPL131W				1.93253		1.63597		0.730645	4.29915
SWI6	YPL160W				2.06991		1.64062		0.732455	4.44299
SWI6	YPL163C	13.2366	0.254011		2.75804				0.580565	16.8292
SWI6	YPL164C	13.2366	0.254011							13.4906
SWI6	YPL198W		0.254011		2.44846		1.64156		0.724487	5.06852
SWI6	YPL199C	0.354386	0.254011	3.57799						4.18639
SWI6	YPL220W				2.26711		1.61197		0.730958	4.61004
SWI6	YPL234C		0.254011		2.38003		1.49276		0.647656	4.77446
SWI6	YPL255W	1.76919	0.254011		1.53944	2.73846	0.97379			7.27489
SWI6	YPL256C	1.76919	0.254011	1.70452	2.5789	1.5229			0.725258	8.55478
SWI6	YPL267W	0.798967	0.241002	3.18574	2.37362				0.640037	7.23937
SWI6	YPR016C		0.254011		1.69339	2.22116	1.60293		0.732129	6.50362
SWI6	YPR041W					2.22116	1.55601		0.732458	4.50963
SWI6	YPR043W				2.30754		1.65763		0.727283	4.69245
SWI6	YPR075C	7.61909							0.706799	8.32589
SWI6	YPR079W	7.61909			1.0629					8.68199
SWI6	YPR102C		0.254011		1.98482		1.54169		0.726884	4.50741
SWI6	YPR103W				1.67195	2.35071	1.6603			5.68296
SWI6	YPR119W	8.71419			0.385982	2.20244	1.94048		0.725537	13.9686
SWI6	YPR120C	4.3776			2.21839					6.59599
SWI6	YPR132W		0.254011		1.58573		1.66927		0.698195	4.20721
SWI6	YPR174C		0.241002	3.43274	1.44738				0.712365	5.83349
SWI6	YPR175W		0.241002	3.53749	1.84688				0.726429	6.3518
TEA1	YBR015C		2.02186	2.79869						4.82055
TEA1	YBR016W		2.02186	3.28562						5.30748
TEA1	YBR132C		2.02186	7.43782						9.45968
TEA1	YCL040W		1.74804	2.39181						4.13985
TEA1	YCL043C		1.74804	2.68837						4.43641
TEA1	YCR052W			4.02754						4.02754
TEA1	YDR540C		1.74804	3.05792						4.80596
TEA1	YFR017C		1.15776	4.01974						5.1775
TEA1	YFR033C			9.02098						9.02098
TEA1	YGR088W			4.61974						4.61974
TEA1	YGR180C		1.15776	3.42048						4.57824
TEA1	YHR057C			4.16963						4.16963
TEA1	YHR081W		1.15776	2.93674						4.0945
TEA1	YJR016C		1.74804	3.09317						4.84121
TEA1	YJR121W		1.15776	3.02826						4.18602
TEA1	YKL187C			7.30854						7.30854
TEA1	YKR066C			6.21903						6.21903
TEA1	YKR067W			5.7432						5.7432
TEA1	YLR055C		1.74804	2.43217						4.18021
TEA1	YLR056W		1.74804	2.32027						4.06831
TEA1	YLR312C		1.74804	4.69706						6.4451
TEA1	YLR312W-A		1.74804	3.97708						5.72512
TEA1	YLR401C			5.19585						5.19585
TEA1	YLR437C			6.85713						6.85713
TEA1	YLR438W			8.94987						8.94987
TEA1	YNL149C		1.74804	4.74023						6.48827
TEA1	YNL196C		1.15776	2.99749						4.15525

TF	Target	Binding_0-2000	Motif_Score_SC	Other_Yeasts	Two-Hop_BE	Two-Hop_BP	Two-Hop_BY	Two-Hop_BG	Max_Membership	Sum
TEA1	YOR140W		0.0691049	4.70196						4.77106
TEA1	YOR161C			4.92664						4.92664
TEA1	YOR269W		2.02186	4.99553						7.01739
TEA1	YOR306C		1.74804	3.14446						4.8925
TEA1	YPR065W			6.88693						6.88693
TEC1	TA(AGC)H	4.52279								4.52279
TEC1	TA(AGC)K2	5.05121								5.05121
TEC1	YBL016W	2.62727			0.516236	0.894476				4.03798
TEC1	YBR040W	3.01329		1.00614	0.824359					4.84379
TEC1	YBR041W	3.01329		1.28977						4.30306
TEC1	YBR082C	6.2393								6.2393
TEC1	YBR083W	6.2393				0.728674				6.96797
TEC1	YBR085W	3.40885		0.804566						4.21342
TEC1	YBR114W			3.2795		0.335279		1.01007		4.62485
TEC1	YCL027W	5.01658			0.439462	1.09139				6.54743
TEC1	YCL029C	5.01658								5.01658
TEC1	YCR028C-A			3.99205		0.335279				4.32733
TEC1	YCR089W	4.76375						1.21861		5.98236
TEC1	YDL124W	3.9028		0.957208						4.86001
TEC1	YDL126C	3.9028		0.39841						4.30121
TEC1	YDL127W	6.11704								6.11704
TEC1	YDL170W			4.10031						4.10031
TEC1	YDR108W			4.04466						4.04466
TEC1	YDR180W	4.71317		0.419711						5.13288
TEC1	YDR317W	4.78687								4.78687
TEC1	YER048W-A	2.37369		2.74428						5.11797
TEC1	YER189W	3.6981			0.51213			1.41063		5.62086
TEC1	YER190W	3.6981			0.690438			1.22549		5.61403
TEC1	YFL037W	4.65303								4.65303
TEC1	YFL039C	4.65303				1.18953				5.84256
TEC1	YGR014W	4.73658		0.364464						5.10104
TEC1	YGR040W	2.38024		0.661276		1.51018				4.5517
TEC1	YGR163W			4.34166						4.34166
TEC1	YHR005C	3.92359				0.544529				4.46812
TEC1	YHR084W	6.20437				0.738104				6.94247
TEC1	YIL118W	4.67473								4.67473
TEC1	YIL119C	4.67473								4.67473
TEC1	YKL094W	3.99708		0.795803						4.79288
TEC1	YKL095W	3.99708		0.919904						4.91698
TEC1	YKR041W	5.05121								5.05121
TEC1	YKR042W	5.05121		0.246655						5.29786
TEC1	YLL066C	4.12678			0.602871			1.22549		5.95514
TEC1	YLR329W			4.44613						4.44613
TEC1	YMR164C	1.93376		3.71339						5.64715
TEC1	YNL053W	5.13821		0.484833						5.62304
TEC1	YNL279W	4.53475			0.513564					5.04831
TEC1	YNL280C	4.53475								4.53475
TEC1	YNL336W	4.03761								4.03761
TEC1	YNL339C	4.03761								4.03761
TEC1	YNR044W	3.64514			0.71961					4.36475
TEC1	YPL163C	3.67047		1.55228						5.22275
THI2	YBR069C	0.250865		5.35851						5.60938
THI2	YBR088C	0.595596		2.93802	0.533862					4.06748
THI2	YBR091C	5.88727	1.10768							6.99495
THI2	YBR092C	5.88727	1.10768	9.03983	0.392868					16.4276
THI2	YCR020C	6.20723	0.504391	7.96258						14.6742
THI2	YCR020C-A	6.20723								6.20723
THI2	YCR020W-B	6.20723	0.504391	7.93424						14.6459
THI2	YDL080C	5.62549	0.889485	7.87869						14.3937
THI2	YDL099W		0.796375	6.64923	0.33212					7.77773
THI2	YDL101C		0.796375	5.1068						5.90318
THI2	YDL107W	0.619503		5.6727						6.2922
THI2	YDR346C		0.286405	6.88693						7.17334
THI2	YDR386W		0.85745	4.18186						5.03931
THI2	YDR438W	7.63515		6.37448						14.0096
THI2	YDR439W	7.63515								7.63515
THI2	YEL050C	3.39902		0.833903						4.23292
THI2	YEL055C		0.593209	3.4567						4.04991
THI2	YEL071W	2.11859		4.36218						6.48077
THI2	YER060W-A	7.44007	0.907462							8.34753
THI2	YER069W	0.753608		2.5957			0.878875			4.22818
THI2	YER115C			3.62245	0.470922					4.09337
THI2	YFL059W		1.13055	6.04807						7.17862
THI2	YFL060C		1.13055	6.08503	0.581937					7.79752
THI2	YFR031C-A		0.568676	5.19455	0.328819					6.09204
THI2	YGL001C	3.80321	0.85745							4.66066
THI2	YGL085W			5.91279						5.91279
THI2	YGR071C	2.03519		2.17376						4.20895
THI2	YGR074W	2.03519		2.16492		0.886708				5.08682
THI2	YGR173W			4.42498						4.42498
THI2	YGR261C		0.796375	5.07117						5.86755
THI2	YHR029C		0.630417	3.9109						4.54132
THI2	YHR063C		0.445652	7.18909						7.63474
THI2	YHR098C			6.33749						6.33749
THI2	YHR105W		0.123883	4.36341						4.48729
THI2	YHR179W	4.82587								4.82587
THI2	YIL001W		0.763807	2.89918	0.777109		0.935109			5.3752
THI2	YIL071C	0.342923		3.97991						4.32283
THI2	YJL132W	0.758608	1.42396	4.29275						6.47532
THI2	YJL167W	0.626002	0.504391	5.82169						6.95208
THI2	YJL168C	0.626002	0.504391	3.65484						4.78523
THI2	YJR010C-A	3.56472	0.620681							4.1854
THI2	YJR011C	3.56472		0.896191						4.46091
THI2	YJR013W	3.56472	0.620681	0.378019						4.56342
THI2	YLR004C	4.9723	1.10768	8.36232						14.4423
THI2	YLR005W	4.9723	1.10768	6.47115						12.5511
THI2	YLR034C		0.395812	4.52093						4.91674
THI2	YLR223C	0.46302	0.398346	4.03054						4.89191
THI2	YLR224W	0.46302	0.398346	6.65699						7.51836
THI2	YMR053C			5.51748						5.51748
THI2	YMR054W			5.71764						5.71764
THI2	YMR187C			4.55826						4.55826
THI2	YMR311C	2.6053		1.63279						4.23809
THI2	YNL287W		0.66013	5.49544						6.15557
THI2	YNL334C		1.13055	1.90419	1.05651					4.09125
THI2	YOL055C	5.71668	1.4498	17.0731						24.2396
THI2	YOL121C		1.4498	6.7768	0.674217					8.90082
THI2	YOL127W	1.55172	1.15776	1.5189	0.350637					4.57902
THI2	YOL128C	1.55172	1.15776	5.1675		0.356544				8.23352
THI2	YOR143C	5.24753	0.235138	1.6297						7.11237

TF	Target	Binding_0-2000	Motif_Score_SC	Other_Yeasts	Two-Hop_BE	Two-Hop_BP	Two-Hop_BY	Two-Hop_BG	Max_Membership	Sum
THI2	YOR254C	0.554817	1.15776	4.71788						6.43046
THI2	YOR266W		0.245559	3.99631						4.24187
THI2	YPL213W	4.60061		0.406374						5.00698
THI2	YPL214C	4.60061		0.345717			0.924508	1.30974		7.18057
THI2	YPL258C	7.09929	1.4498	14.7109				0.859484		24.1195
THI2	YPR121W	4.82339	0.66013	3.99157						9.47509
THI2	YPR133C		1.16551	6.37311						7.53862
THI2	YPR134W		1.16551	5.197						6.36251
TYE7	TC(GCA)B	4.58458	0.0421904							4.62677
TYE7	TE(UUC)B	8.61329	0.0421904							8.65548
TYE7	TP(UGG)A	4.30847	0.0421904							4.35066
TYE7	TY(GUA)D	4.15176								4.15176
TYE7	YAL007C	4.30847	0.0421904							4.35066
TYE7	YAL012W		1.14889	1.62655	0.548455				0.714342	4.03824
TYE7	YAL026C	1.55372	1.14889	4.8999						7.60251
TYE7	YBR039W	3.16921	1.14889	6.4194						10.7375
TYE7	YBR049C	0.115275	1.14889	4.40957						5.67374
TYE7	YBR143C	3.86925			0.699896	0.511492				5.08064
TYE7	YBR210W	8.61329	0.0421904							8.65548
TYE7	YBR246W			4.34472						4.34472
TYE7	YBR247C		1.14889		2.05933	1.0489				4.25712
TYE7	YCL034W		1.14889	8.93856						10.0875
TYE7	YCL035C	0.0719734	1.14889	10.1178	0.414806					11.7535
TYE7	YCR087C-A	2.01275	0.534152	2.17735						4.72425
TYE7	YCR088W	2.01275	0.534152	1.65216		1.0489				5.24796
TYE7	YCR092C		1.14889	3.80545						4.95434
TYE7	YCR093W		1.14889	7.34313		0.344078				8.8361
TYE7	YDL020C		1.14889	9.01419						10.1631
TYE7	YDL027C	4.95814								4.95814
TYE7	YDL028C	4.95814								4.95814
TYE7	YDL051W	3.45933			0.882943					4.34227
TYE7	YDL073W	1.51768	1.14889	5.54629						8.21286
TYE7	YDL074C	1.51768	1.14889	7.82185						10.4884
TYE7	YDL112W		1.14889	3.8849			0.779957			5.81375
TYE7	YDR023W		0.666779	2.25093	0.646168	1.0489				4.61278
TYE7	YDR113C		1.14889	5.42564						6.57453
TYE7	YDR115W		1.14889	5.14975			0.870937			7.16958
TYE7	YDR160W	6.39201								6.39201
TYE7	YDR240C	4.15176								4.15176
TYE7	YDR242W	4.15176								4.15176
TYE7	YEL001C			6.90127	0.435339					7.33661
TYE7	YER021W	3.0414	1.02719	0.519764						4.58835
TYE7	YER043C			3.76474	0.608492					4.37323
TYE7	YER060W-A	4.67948	1.14889							5.82837
TYE7	YER080W	0.429327	1.14889	2.73989						4.31811
TYE7	YER091C	0.344496	1.02719	4.6441					0.709264	6.72505
TYE7	YER092W	0.344496	1.02719	9.70779						11.0795
TYE7	YER112W	0.804967	1.14889	2.87222						4.82608
TYE7	YER114C	4.89642	0.534152	2.53973						7.9703
TYE7	YER124C	1.27425	1.02719	4.92369						7.22513
TYE7	YER125W	1.27425	1.02719	4.74158						7.04302
TYE7	YER177W	3.41899	0.534152	1.3803		0.375589				5.70903
TYE7	YFL017W-A		1.14889	5.02268		0.765538				6.93711
TYE7	YFL018C		1.14889	6.87215						8.02104
TYE7	YFL023W			3.08635	0.921741					4.00809
TYE7	YFL024C			7.17319						7.17319
TYE7	YFR030W	5.15072	0.342972	3.18889	0.465388			1.1016	0.567915	10.8175
TYE7	YGL001C	3.75428		3.1484						6.90268
TYE7	YGL127C		1.02719	3.88312						4.91031
TYE7	YGR128C	8.5322	1.02719		0.486915					10.0463
TYE7	YGR192C	3.97882			1.16456					5.14338
TYE7	YGR223C	1.95293	1.14889	5.1702						8.27202
TYE7	YGR224W	1.95293	1.14889	1.11998			0.91648			5.13828
TYE7	YHR001W-A	1.24016	1.14889	5.6406						8.02965
TYE7	YHR089C	2.28421	1.14889	0.990605	1.02368	0.578613				6.026
TYE7	YHR137W		1.02719	3.23114						4.25833
TYE7	YIL127C		1.14889	10.0267	2.06151					13.2371
TYE7	YJL167W		1.14889	5.23658						6.38547
TYE7	YJL186W	1.90633	1.14889	5.04388						8.0991
TYE7	YJL187C	1.90633	1.14889	3.12802		0.702024				6.88526
TYE7	YJR009C	4.2753	1.02719	1.66995	1.104					8.07644
TYE7	YJR010W	4.2753	1.02719	3.10265					0.639468	9.04461
TYE7	YJR095W		1.14889	6.89832	0.522255					8.56946
TYE7	YJR119C		1.14889	2.07901				1.41579		4.64369
TYE7	YJR121W		1.14889	2.8217		1.30006				5.27065
TYE7	YJR137C	3.01059	1.14889	2.78885	0.902559			1.48724	0.699732	10.0379
TYE7	YJR138W	3.01059	1.14889	7.35053						11.51
TYE7	YJR139C	2.17101	1.14889	8.61128	0.812457					12.7436
TYE7	YKL001C		1.14889	6.30913				1.32949		8.78751
TYE7	YKL007W	0.577408	1.14889	2.4548						4.1811
TYE7	YKL015W	5.42602	1.14889	3.08139						9.6563
TYE7	YKL016C	5.42602	1.14889	6.34149						12.9164
TYE7	YKL017C	5.42602	1.14889							6.57491
TYE7	YKL191W	1.21552	1.02719	5.10395						7.34666
TYE7	YKL192C	1.21552	1.02719	5.03838						7.28109
TYE7	YKL193C	1.21552	1.02719	4.89324	0.657895					7.79384
TYE7	YKR057W	3.37443			1.01083					4.38526
TYE7	YKR058W	3.37443					0.938724		0.553176	4.86633
TYE7	YLL015W	5.74199								5.74199
TYE7	YLR283W		1.02719	3.02884						4.05603
TYE7	YLR303W			3.71175					0.713406	4.42516
TYE7	YML121W		1.14889	7.70772						8.85661
TYE7	YML123C		1.14889	4.93554						6.08443
TYE7	YMR037C	5.1742	0.666779	0.457035						6.29801
TYE7	YMR108W			4.13204						4.13204
TYE7	YMR196W	5.68791	1.14889	4.01367					0.482929	11.3334
TYE7	YMR290C			5.59889	1.9453	0.807843				8.35203
TYE7	YMR291W			5.74078	0.3551					6.09588
TYE7	YMR301C	2.28566	1.14889	2.46512						5.89967
TYE7	YNL036W		1.14889	5.50069					0.711135	7.36071
TYE7	YNL037C		1.14889	5.92685	0.534205					7.60994
TYE7	YNL220W			7.72136						7.72136
TYE7	YNL232W	4.79334	1.02719	4.86677						10.6873
TYE7	YNL277W	0.678822	1.14889	6.9668					0.474908	9.26942
TYE7	YNR051C	2.56149		2.39827						4.95976
TYE7	YOL064C	3.29563		1.63726					0.722266	5.65516
TYE7	YOR001W		1.14889	1.55208	2.02602	0.938736				5.66573
TYE7	YOR039W	3.28367	1.14889	9.19733						13.6299
TYE7	YOR040W	3.28367	1.14889							4.43256
TYE7	YOR099W			7.78917						7.78917

TF	Target	Binding_0-2000	Motif_Score_SC	Other_Yeasts	Two-Hop_BE	Two-Hop_BP	Two-Hop_BY	Two-Hop_BG	Max_Membership	Sum
TYE7	YOR298C-A		1.02719	3.74114						4.76833
TYE7	YOR299W		1.02719	4.04723						5.07442
TYE7	YPL093W		1.02719	3.9497	1.11361					6.0905
TYE7	YPL190C		0.666779	5.18676						5.85354
TYE7	YPL204W	7.39059	1.14889	0.899652						9.43913
TYE7	YPL206C	7.39059	1.14889	1.0206						9.56008
TYE7	YPL236C		1.14889	1.70057				1.46393		4.31339
UGA3	TK(CUU)C	4.17962								4.17962
UGA3	TR(UCU)E	6.54746								6.54746
UGA3	YBL015W		1.192	3.50495						4.69695
UGA3	YBR127C			3.853	0.577096					4.4301
UGA3	YBR247C			5.81879	0.569466					6.38826
UGA3	YBR297W		1.47396	9.00717						10.4811
UGA3	YCR019W	4.17962								4.17962
UGA3	YCR057C			4.82617		0.479289				5.30546
UGA3	YCR060W			6.74414						6.74414
UGA3	YDL210W	0.128018	1.47396	7.02409						8.62607
UGA3	YDR079W	3.92545	0.895871							4.82132
UGA3	YDR084C	4.06093								4.06093
UGA3	YER188C-A	4.04754								4.04754
UGA3	YER189W	4.04754			0.58351			1.28845		5.9195
UGA3	YER190W	4.04754			0.58351			1.28845		5.9195
UGA3	YFL021W		1.47396	2.9834						4.45736
UGA3	YFL037W		0.895871	4.18768						5.08355
UGA3	YGL009C		1.192	3.26838						4.46038
UGA3	YGL055W		1.192	3.21649						4.40849
UGA3	YGR019W	1.34224	1.47396	8.06987						10.8861
UGA3	YHL004W		1.192	4.63165						5.82365
UGA3	YHR016C	4.36697								4.36697
UGA3	YHR204W			6.73898						6.73898
UGA3	YJL093C		1.192	5.69803						6.89003
UGA3	YJR077C		1.47396	7.42823	0.442612					9.3448
UGA3	YJR078W		1.47396	8.14754						9.6215
UGA3	YLL066C	2.80838			0.674251			1.28845		4.77108
UGA3	YLR073C		1.192	3.63017						4.82217
UGA3	YLR436C		0.582795	4.03469						4.61749
UGA3	YLR437C		0.582795	5.88333						6.46612
UGA3	YML008C			4.19193	0.618619					4.81055
UGA3	YMR055C			3.07276				1.02527		4.09803
UGA3	YMR275C		1.192	2.89097						4.08297
UGA3	YNL241C	3.67311	0.582795	0.146161						4.40207
UGA3	YNL304W			4.51276						4.51276
UGA3	YNL336W	5.9369								5.9369
UGA3	YNL339C	5.9369								5.9369
UGA3	YNR018W		1.192	3.05314						4.24514
UGA3	YNR041C			5.02042						5.02042
UGA3	YOR177C		1.192	3.57777				1.20695		5.97672
UGA3	YPR035W		1.47396	3.5016						4.97556
UGA3	YPR117W	0.0820195		4.13103						4.21305
UME1	SNR53	4.93143	0.753703							5.68513
UME1	TK(CUU)C	7.61515								7.61515
UME1	TR(UCU)E	9.51131	0.254011							9.76532
UME1	TT(AGU)C	3.1964	1.24189							4.43829
UME1	YAL036C			4.56619						4.56619
UME1	YBL005W		1.05095	3.59196						4.64291
UME1	YBL007C		1.05095	3.43728						4.48823
UME1	YBL016W		0.435593	4.81999						5.25558
UME1	YBL049W			4.25224						4.25224
UME1	YBR008C			4.45956	2.0588					6.51836
UME1	YBR046C	3.00053	0.439996				1.35061			4.79114
UME1	YBR048W	3.00053	0.439996	1.80739	0.747671					5.99559
UME1	YCL038C			4.04166						4.04166
UME1	YCL067C	5.48712	0.70591		0.421637					6.61467
UME1	YCR019W	7.61515								7.61515
UME1	YCR020C	2.34991	0.495681		1.75531					4.6009
UME1	YCR065W		1.24189	3.75945	0.518757					5.5201
UME1	YCR096C	3.1964	1.24189		0.88957	0.698169				6.02603
UME1	YCR097W	3.1964	1.24189			0.698169	0.809329			5.94579
UME1	YCR105W	9.95034					0.82151			10.7719
UME1	YCR107W	10.8328								10.8328
UME1	YDL098C		1.22823	6.37189						7.60012
UME1	YDR084C	4.31683	0.612149							4.92898
UME1	YDR184C		0.254011	3.42271	0.751191					4.42791
UME1	YDR218C		0.532296	3.66606						4.19836
UME1	YDR258C		1.05095	6.30478	0.39463					7.75036
UME1	YDR304C	0.466413		4.44309						4.9095
UME1	YDR318W		1.24189	3.2448						4.48669
UME1	YDR443C	0.169596		5.5695						5.7391
UME1	YDR444W	0.169596		4.76051						4.93011
UME1	YEL011W		1.24189	7.96454						9.20643
UME1	YEL013W		1.24189	12.1873						13.4292
UME1	YEL050C	4.93143	0.753703							5.68513
UME1	YER015W			4.00706						4.00706
UME1	YER044C	2.64477	0.495681		1.24675					4.3872
UME1	YER115C			4.16113						4.16113
UME1	YER122C		1.24189	8.15384			0.779191			10.1749
UME1	YER123W		1.24189	4.20988						5.45177
UME1	YER127W		1.22823	6.3996						7.62783
UME1	YER188C-A	3.16533	0.860953							4.02628
UME1	YER189W	3.16533	0.860953		0.4033			1.12948		5.55906
UME1	YER190W	3.16533	0.860953		0.4033			1.12948		5.55906
UME1	YFL017C		0.473223	4.14079						4.61401
UME1	YGL001C	5.70654	0.0496046		0.621311		1.31977			7.69723
UME1	YGL020C		1.24189	5.58107						6.82296
UME1	YGL032C			4.97413						4.97413
UME1	YGL049C			4.15254						4.15254
UME1	YGL171W			4.74562	1.47528					6.2209
UME1	YGL204C		0.943245	4.61945						5.56269
UME1	YGR060W		1.24189	3.84582		1.37579				6.4635
UME1	YGR147C		1.05095	3.80542						4.85637
UME1	YHL032C	0.332841	0.943245	3.55949						4.83558
UME1	YHL036W			4.17066						4.17066
UME1	YHL038C			4.1086						4.1086
UME1	YHR007C			5.5306						5.5306
UME1	YHR011W	1.26898	1.05095	1.13091	0.863882					4.31472
UME1	YIL070C			4.43039	0.405053					4.83544
UME1	YJR011C	3.69946		0.555389	0.669869					4.92472
UME1	YJR013W	3.69946		0.478555						4.17802
UME1	YJR040W		0.315402	3.76218						4.07758
UME1	YKL041W	0.439712	0.30704	3.25565						4.0024

TF	Target	Binding_0-2000	Motif_Score_SC	Other_Yeasts	Two-Hop_BE	Two-Hop_BP	Two-Hop_BY	Two-Hop_BG	Max_Membership	Sum
UME1	YKR063C		1.24189	5.71456						6.95645
UME1	YKR075C			5.38313						5.38313
UME1	YKR076W			4.84973						4.84973
UME1	YLR095C	1.45621	1.05095	2.37326						4.88042
UME1	YLR096W	1.45621	1.05095	1.5875						4.09466
UME1	YLR449W		1.05095	2.61187	0.380068					4.04289
UME1	YMR193W	4.44998	1.22823	2.63791						8.31612
UME1	YMR194W	4.44998	1.22823	0.487637	0.451587					6.61743
UME1	YMR258C	5.01153								5.01153
UME1	YMR287C		1.05095	3.20856						4.25951
UME1	YNL032W			7.03423						7.03423
UME1	YNL339C	2.60844	0.860953	0.690988						4.16038
UME1	YNR049C		1.22823	8.80748						10.0357
UME1	YOL104C	3.1279	0.473223	0.964204						4.56533
UME1	YOR001W	7.18281	0.175503		0.334552					7.69287
UME1	YOR056C	0.113221	1.03682	5.04283	0.826995					7.01987
UME1	YOR298W		1.24189	6.83385						8.07574
UME1	YOR308C		1.05095	3.61329						4.66424
UME1	YPL027W		1.05095	3.85795						4.9089
UME1	YPL041C			4.09047						4.09047
UME1	YPL047W		1.05095	4.17669						5.22764
UME1	YPL260W		1.05095	4.27046						5.32141
UME1	YPR011C		1.05095	4.52008						5.57103
UME1	YPR110C		0.15901	2.81015	1.50157					4.47073
UME1	YPR127W	3.88909	0.612149		1.05761		1.53219			7.09104
UME1	YPR131C		1.05095	3.01689						4.06784
UME6	TG(GCC)F2	3.04741	2.27351							5.32092
UME6	YAL005C	0.255921	1.50061	1.72938	1.0005					4.48641
UME6	YAL038W	0.218851	0.471347	9.23047						9.92067
UME6	YAL053W	2.80129	2.11198	4.00306						8.91633
UME6	YAL054C	2.80129	2.11198	2.70578	0.496454					8.1155
UME6	YBL015W	2.99337	1.40066	1.6152	0.457217				0.720993	7.18744
UME6	YBL063W	4.65645	2.27351	2.61186				1.02755		10.5694
UME6	YBL064C	4.65645	2.27351	4.35881					0.719843	12.0086
UME6	YBL066C	4.65645	2.27351		0.837041					7.767
UME6	YBL078C	4.6546	0.752513	0.162681						5.56979
UME6	YBL085W	1.86921	2.27351							4.14272
UME6	YBL086C	1.86921	2.27351	6.14189						10.2846
UME6	YBR088C	0.839185	2.11198	3.86192	0.541401	0.537129			0.726537	8.61815
UME6	YBR089C-A	0.839185	2.11198	3.89979				1.14575		7.99671
UME6	YBR114W	2.09058	1.36606	0.845248				0.94983		5.25172
UME6	YBR182C-A	3.27829	1.50061							4.7789
UME6	YBR184W	3.27829	1.50061	1.36457						6.14347
UME6	YBR240C		2.11198	2.59513						4.70711
UME6	YBR249C		1.21124	2.94175	0.697788					4.85078
UME6	YBR250W		1.21124	2.90594						4.11718
UME6	YBR256C	1.62744	2.11198	2.32498						6.0644
UME6	YBR257W	1.62744	2.11198	2.26217						6.00159
UME6	YBR283C	2.02314	1.36606	4.5522						7.9414
UME6	YBR284W	2.02314	1.36606	4.44663	0.544966					8.3808
UME6	YCL024W	3.8891	2.27351	6.78017	0.509806	0.84438		0.868528		15.1655
UME6	YCL025C	3.8891	2.27351	6.14824						12.3109
UME6	YCL039W		2.27351	1.49567				0.863947		4.63313
UME6	YCL043C		2.27351	3.03704						5.31055
UME6	YCR010C		2.11198	1.92805	0.609042				0.620205	5.26928
UME6	YCR059C	3.4744	2.27351	0.503866						6.25178
UME6	YCR061W	3.4744	2.27351	8.20152					0.488063	14.4375
UME6	YDL072C		2.27351	7.52247	0.490629					10.2866
UME6	YDL183C	0.316681	2.11198	2.7962						5.22486
UME6	YDL193W	3.44309	2.27351	0.974508						6.69111
UME6	YDL210W	3.34907	0.86912	0.641329						4.85952
UME6	YDL215C	2.66784	0.718495	0.796504						4.18284
UME6	YDL239C	0.21039	2.11198	1.73136		0.537129	0.783071	0.977383		6.35131
UME6	YDR054C	3.27068	2.11198	1.95759						7.34025
UME6	YDR055W	3.27068	2.11198	2.09672						7.47938
UME6	YDR091C	2.67882	2.27351	1.04814						6.00047
UME6	YDR092W	2.67882	2.27351		0.403605					5.35593
UME6	YDR284C	2.5579	2.27351	1.56614						6.39755
UME6	YDR285W	2.5579	2.27351	7.41489						12.2463
UME6	YDR359C	2.17669	2.27351	9.02513						13.4753
UME6	YDR374C	0.861477	2.27351	7.7076						10.8426
UME6	YDR487C	1.50613	2.27351	5.05016						8.8298
UME6	YDR508C	2.13254	1.50061	0.722541						4.35569
UME6	YDR510W	2.13254	1.50061	1.53234		0.353127				5.51862
UME6	YDR511W	2.13254	1.50061	1.46656						5.09971
UME6	YEL024W	2.51549	2.11198	3.24775	0.412167				0.530965	8.81835
UME6	YEL025C	2.51549	2.11198	1.87725						6.50472
UME6	YEL056W		2.27351	11.3685	0.683408					14.3254
UME6	YEL057C		2.27351	10.4584						12.7319
UME6	YER044C	2.20908	2.11198		0.474759					4.79582
UME6	YER044C-A	2.20908	2.11198							4.32106
UME6	YER111C	4.49012	2.27351	10.8013			0.781489			18.3464
UME6	YER112W	4.49012	2.27351	7.5399		0.5067				14.8102
UME6	YER130C	2.16621	1.54838	2.07991				0.905307		6.69981
UME6	YER131W	2.16621	1.54838		0.749642					4.46423
UME6	YER179W	2.30567	2.11198	0.677939						5.09559
UME6	YFL021W	2.4141	2.27351	2.06551						6.75312
UME6	YFL022C	2.4141	2.27351	1.91264						6.60025
UME6	YFL036W	0.167336	0.866205	5.3635						6.39704
UME6	YFR016C	3.04741	2.27351	0.98091	0.651494			1.25897		8.21229
UME6	YFR017C	3.01166	2.27351	1.88919						7.17436
UME6	YFR019W	3.01166	2.27351	0.58404				0.94514		6.81435
UME6	YGL033W		2.27351	4.85868						7.13219
UME6	YGL062W	0.122313	2.11198	4.3743						6.60859
UME6	YGL064C	0.122313	2.11198	3.43232						5.66661
UME6	YGL181W	2.44646	2.11198	0.501549				1.25663		6.31662
UME6	YGL184C	2.44646	2.11198	2.07823						6.63667
UME6	YGL225W		2.11198	3.64853						5.76051
UME6	YGL249W	2.88618	1.36606							4.25224
UME6	YGL250W	2.88618	1.36606	0.967446						5.21969
UME6	YGR138C	1.07389	2.11198	1.4278						4.61367
UME6	YGR140W	1.07389	2.11198	0.853309						4.03918
UME6	YGR194C	1.57267	0.866205	4.18053	0.491501				0.64731	7.75822
UME6	YGR195W	1.57267	0.866205	3.58623		0.4317				6.45681
UME6	YGR264C	1.96441	2.27351	6.16714	0.578792					10.9839
UME6	YGR266W	1.96441	2.27351	8.85633						13.0942
UME6	YGR267C	3.46547	0.866205							4.33167
UME6	YGR268C	3.46547	0.866205	0.557422					0.647154	5.53625
UME6	YGR270W	3.46547	0.866205	0.55041						4.88209
UME6	YGR280C	3.9485	0.598714	0.871181	0.380395					5.79879

TF	Target	Binding_0-2000	Motif_Score_SC	Other_Yeasts	Two-Hop_BE	Two-Hop_BP	Two-Hop_BY	Two-Hop_BG	Max_Membership	Sum
UME6	YGR281W	3.9485	0.598714	0.0658525					0.701316	5.31438
UME6	YHL024W	1.88393	2.11198	2.78481	0.52414				0.58364	7.8885
UME6	YHL030W	3.86021	0.718495	1.78929						6.368
UME6	YHL031C	3.86021	0.718495	2.69352						7.27223
UME6	YHL032C	3.86021	0.718495							4.57871
UME6	YHR013C	1.74471	2.27351	4.63331						8.65153
UME6	YHR014W	1.74471	2.27351	4.50013						8.51835
UME6	YHR124W	2.0649	1.22987	0.83869						4.13346
UME6	YHR135C	2.91329	1.50061					0.868528		5.28243
UME6	YHR136C	2.91329	1.50061	2.64846						7.06236
UME6	YHR137W	2.91329	1.50061	5.69094					0.643833	10.7487
UME6	YHR153C	2.73606	2.11198	3.83715					0.626651	9.31184
UME6	YHR154W	2.73606	2.11198	2.0331						6.88114
UME6	YHR156C		1.36606	3.6916						5.05766
UME6	YHR157W		1.36606	5.64536						7.01142
UME6	YIL026C	0.179338	2.27351	8.15203						10.6049
UME6	YIL031W	2.47714	2.27351	9.23419						13.9848
UME6	YIL033C	2.47714	2.27351	9.56618				0.901846		15.2187
UME6	YIL072W		2.27351	5.21389		0.471521				7.95892
UME6	YIL073C		2.27351	5.21324						7.48675
UME6	YIL144W	2.2781		4.01185			0.802859	0.939919		8.03273
UME6	YIL145C	2.2781		8.39094						10.669
UME6	YJL089W	2.69624	1.50061	3.08783		0.441159		0.89473		8.62057
UME6	YJL090C	2.69624	1.50061	2.24685						6.4437
UME6	YJL103C	2.84361	0.572977	0.181211	0.464153				0.71333	4.77528
UME6	YJL106W	1.32015	1.52039	9.01851				0.868528		12.7276
UME6	YJL107C	1.32015	1.52039	2.15414						4.99468
UME6	YJL133W	1.69126	1.62644	2.78909						6.10679
UME6	YJL149W	3.12989	2.27351						0.59639	5.99979
UME6	YJL153C	3.12989	2.27351	4.73923						10.1426
UME6	YJR006W	1.39955	2.27351	5.76904					0.67584	10.1179
UME6	YJR095W	1.96788	2.11198	0.261105						4.34096
UME6	YJR119C	2.17264	2.11198	2.79249				1.29343		8.37054
UME6	YJR121W	2.17264	2.11198	3.58729		0.730375			0.627945	9.23023
UME6	YJR137C	0.466677	2.27351	1.77954						4.51973
UME6	YKL015W	1.86261	1.50061	0.444057				1.04494		4.85222
UME6	YKL016C	1.86261	1.50061	2.15044		0.4218				5.93546
UME6	YKL078W	3.37669	2.27351	8.39643						14.0466
UME6	YKL085W	1.23482	1.40066	0.305535	0.400621	0.356005			0.728468	4.42611
UME6	YKL190W	1.71717	1.36606	0.00695364				1.29887		4.38905
UME6	YKL217W	1.11749	2.11198	2.04373	0.712758				0.63515	6.62111
UME6	YKR009C	1.98872	0.783962	0.189127	0.457392				0.668148	4.08735
UME6	YLL019C	3.77907	2.27351	6.29114	0.442914			0.868528	0.62034	14.2755
UME6	YLL029W	3.92883	2.27351	6.31316						12.5155
UME6	YLL031C	3.92883	2.27351	5.03619						11.2385
UME6	YLL039C	3.51991	2.11198	1.05481					0.693263	7.37996
UME6	YLL046C	3.65957	2.27351	5.16569						11.0988
UME6	YLL048C	3.65957	2.27351							5.93308
UME6	YLR096W	3.33374						0.868528		4.20227
UME6	YLR114C	2.61117	0.866205	1.91582						5.3932
UME6	YLR115W	2.61117	0.866205	1.12536						4.60274
UME6	YLR131C	1.50334	2.11198	2.57429						6.18961
UME6	YLR259C	2.1873	2.11198	1.57428	0.862285			1.09369		7.82953
UME6	YLR260W	2.1873	2.11198	1.85392						6.1532
UME6	YLR295C	3.31571	1.37939	1.85414		0.4218				6.97104
UME6	YLR297W	3.31571	1.37939	0.166969						4.86207
UME6	YLR314C	3.73802	2.11198	3.07257		0.885166	0.78686	0.930876		11.5255
UME6	YLR315W	3.73802	2.11198	2.73439						8.58439
UME6	YLR332W	1.61871	1.36606	1.03305						4.01782
UME6	YLR344W	1.53242	1.72653		0.967972					4.22692
UME6	YLR345W	1.53242	1.72653		0.738386				0.64166	4.639
UME6	YLR375W	0.893883	1.52039	1.63265	0.775642					4.82256
UME6	YLR414C	1.69898	1.52039	1.06055	0.331995					4.61191
UME6	YLR417W	1.69898	1.52039	1.06687						4.28624
UME6	YLR437C	2.33398	1.1898	3.60805						7.13183
UME6	YLR438W	2.33398		5.67304						8.00702
UME6	YLR446W	1.23402	2.27351	8.18172						11.6893
UME6	YLR450W	0.905318	2.11198	2.50391						5.52121
UME6	YML042W	3.52101	2.27351	9.69093	0.791518				0.54542	16.8224
UME6	YML043C	3.52101	2.27351	8.71741	0.374507					14.8864
UME6	YML088W	1.76799	1.50061	0.993222						4.26182
UME6	YML101C	1.41568	2.27351	1.13984						4.82903
UME6	YMR018W	1.57188	1.36606	4.34036						7.2783
UME6	YMR070W	2.51116	2.27351	1.59118						6.37585
UME6	YMR076C	3.93495	1.36606	0.78197				1.09904		7.18202
UME6	YMR099C		1.36606	6.48165						7.84771
UME6	YMR100W		1.36606	5.82464						7.1907
UME6	YMR101C	3.07746	2.27351	7.81784						13.1688
UME6	YMR108W	2.30047	1.72653							4.027
UME6	YMR132C	0.444178	2.11198	3.72355						6.27971
UME6	YMR133W	0.444178	2.11198	5.74525					0.629675	8.93108
UME6	YMR186W	4.64153	2.27351	6.26697	0.533586				0.453193	14.1688
UME6	YMR280C	2.24436	1.50061	1.47915		0.563145				5.78726
UME6	YMR281W	2.24436	1.50061	2.30355						6.04852
UME6	YMR290C	2.79433	2.27351	5.6783	0.521585					11.2677
UME6	YMR291W	2.79433	2.27351	6.65799				0.868528		12.5944
UME6	YMR305C	1.49626	0.866205	1.88662	0.331599					4.58068
UME6	YNL012W		0.866205	3.22542						4.09163
UME6	YNL146W	2.48715	1.52039							4.00754
UME6	YNL147W	2.48715	1.52039	0.515075		1.04741				5.57003
UME6	YNL149C	2.48715	1.52039	1.10992						5.11746
UME6	YNL151C	2.48715	1.52039	0.0452576	0.79619					4.84899
UME6	YNL156C	0.827697	2.27351	8.00371	0.441901					11.5468
UME6	YNL186W	2.98218	2.27351	0.182949	0.577788					6.01643
UME6	YNL196C	0.774884	2.27351	6.48143						9.52982
UME6	YNL209W	1.25947	2.27351		0.790807					4.32379
UME6	YNL210W	1.25947	2.27351	5.32506						8.85804
UME6	YNL211C	1.25947	2.27351	6.40989						9.94287
UME6	YNL251C	0.67209	1.50061	2.66524						4.83794
UME6	YNL271C	1.14939	2.27351	4.13297						7.55587
UME6	YNL304W	1.75586	1.21124	1.05338					0.658382	4.67886
UME6	YNR001C	1.92892	0.544655	0.156484	0.83142				0.676201	4.13768
UME6	YNR049C			4.52491						4.52491
UME6	YOL052C-A	3.4944	0.658316	0.79184						4.94456
UME6	YOL091W	2.59986	2.27351					1.39814		6.27151
UME6	YOL092W	2.59986	2.27351	7.53005	0.350392					12.7538
UME6	YOL094C	2.59986	2.27351	0.205834						5.0792
UME6	YOL100W	4.10538	2.11198	0.797852	0.36269			0.868528	0.528357	8.77479
UME6	YOL101C	4.10538	2.11198	2.26639						8.48375
UME6	YOL104C	4.00841	2.11198	3.94154						10.0619

TF	Target	Binding_0-2000	Motif_Score_SC	Other_Yeasts	Two-Hop_BE	Two-Hop_BP	Two-Hop_BY	Two-Hop_BG	Max_Membership	Sum
UME6	YOL105C	4.00841	2.11198							6.12039
UME6	YOL110W	2.88703	2.11198							4.99901
UME6	YOL123W	2.16663	2.11198	5.79337						10.072
UME6	YOL124C	2.16663	2.11198	5.52029	0.439178					10.2381
UME6	YOL131W	2.27126	1.50061	2.09873					0.548471	6.41907
UME6	YOR020C	1.63446	2.11198	4.43225		0.425757				8.60445
UME6	YOR076C	2.20932	2.27351	4.18753						8.67036
UME6	YOR078W	2.20932	2.27351	5.68039						10.1632
UME6	YOR081C	1.79552	2.27351							4.06903
UME6	YOR084W	1.79552	1.22987	6.31571						9.3411
UME6	YOR085W	1.79552	2.27351	0.0871662						4.1562
UME6	YOR100C	3.87332	2.11198	2.53285						8.51815
UME6	YOR101W	3.87332	2.11198	1.80506				0.999516		8.78988
UME6	YOR152C	1.39459	1.50061	0.831266	0.572645					4.29911
UME6	YOR177C	1.28058	1.33554	1.60694				1.02753		5.25059
UME6	YOR290C	4.25378	1.72653	1.83125				0.918418		8.72998
UME6	YOR291W	4.25378	1.72653	2.25738						8.23769
UME6	YOR338W	2.4013	2.27351	7.81427						12.4891
UME6	YOR351C	0.670905	2.27351	9.02023		0.880905		0.868528		13.7141
UME6	YOR352W	0.670905	2.27351	6.11211	0.791431					9.84796
UME6	YOR354C	2.83541	2.27351	2.05033						7.15925
UME6	YOR355W	2.83541	2.27351							5.10892
UME6	YOR387C	1.58197	2.11198	0.573725						4.26768
UME6	YPL016W	3.34474	2.27351	3.4695						9.08775
UME6	YPL017C	3.34474	2.27351	7.19456						12.8128
UME6	YPL111W	1.29163	2.27351	4.04388		0.81002				8.41904
UME6	YPL112C	1.29163	2.27351	0.935962						4.5011
UME6	YPL120W	1.73942	2.11198	2.5531						6.4045
UME6	YPL121C	1.73942	2.11198	1.99294						5.84434
UME6	YPL122C	1.73942	2.11198	2.09978						5.95118
UME6	YPL160W	4.31654	2.27351	0.0785239	0.535427					7.204
UME6	YPL161C	4.31654	2.27351	0.260114	0.392246					7.24241
UME6	YPL198W	3.12863	2.27351		0.778087					6.18023
UME6	YPL200W	3.12863	2.27351	7.2011						12.6032
UME6	YPL201C	3.12863	2.27351	3.73887	1.14914					10.2902
UME6	YPR006C	2.7104	0.718495	1.51933						4.94822
UME6	YPR007C	1.26965	1.50061	2.77409						5.54435
UME6	YPR008W	1.26965	1.50061	2.45973				0.921247		6.15124
UME6	YPR029C	2.92059	2.27351	6.17154						11.3656
UME6	YPR030W	2.92059	2.27351	8.92808	0.914168				0.464312	15.5007
UME6	YPR065W	1.89041	1.2063	1.1236						4.22031
UME6	YPR119W	1.76779	1.36606	0.199916		1.04741				4.38118
UME6	YPR151C	3.87253	2.27351	5.7543	0.326324					12.2267
UME6	YPR163C	2.31761	2.11198	2.75909	0.695982					7.88466
UME6	YPR164W	2.31761	2.11198	1.74576						6.17535
UPC2	YJR088C	5.10776								5.10776
UPC2	YJR089W	5.10776								5.10776
WTM1	TL(CAA)M	4.36581								4.36581
WTM1	TV(AAC)L	4.90966								4.90966
WTM1	YDR317W	6.21246								6.21246
WTM1	YLR272C	4.90966								4.90966
WTM1	YMR118C	4.36581								4.36581
WTM1	YMR119W	4.36581			0.428635					4.79444
XBP1	YBL011W			4.84542						4.84542
XBP1	YBR002C		0.752513	3.93745						4.68996
XBP1	YBR145W		0.752513	4.34166						5.09417
XBP1	YCL028W	0.673285	0.752513	4.37463						5.80043
XBP1	YCR105W	7.7022								7.7022
XBP1	YCR107W	8.64005								8.64005
XBP1	YDL168W	0.291128	0.752513	1.30973	1.32008		1.15787			4.83132
XBP1	YDR044W		0.752513	3.36365						4.11616
XBP1	YDR287W		0.752513	3.58089						4.3334
XBP1	YDR309C	5.13066		0.250565	0.536889					5.91811
XBP1	YER009W		0.752513	4.17211						4.92462
XBP1	YER018C		0.752513	3.71629						4.4688
XBP1	YER171W		0.752513	2.2546			1.15635			4.16346
XBP1	YER188C-A	5.6401								5.6401
XBP1	YER189W	5.6401			0.908828			1.57543		8.12436
XBP1	YER190W	5.6401			0.908828			1.57543		8.12436
XBP1	YGL067W		0.752513	4.67052						5.42303
XBP1	YGR096W		0.752513	3.73537						4.48788
XBP1	YGR100W			4.61549						4.61549
XBP1	YGR249W	4.76218	0.752513	1.71214						7.22683
XBP1	YHR041C	0.790856	0.752513	2.65961						4.20298
XBP1	YHR050W	1.83779	0.752513	1.60409						4.19439
XBP1	YIL118W	3.93119	0.752513							4.6837
XBP1	YIL119C	3.93119	0.752513	0.711142						5.39484
XBP1	YJR030C		0.752513	3.49265						4.24516
XBP1	YJR094C	3.445	0.752513	0.395034						4.59255
XBP1	YJR119C	0.195944	0.752513	2.27263				1.02786		4.24895
XBP1	YJR121W	0.195944	0.752513	3.28775						4.23621
XBP1	YKL043W	4.4811	0.752513							5.23361
XBP1	YLR178C	3.85858			0.79674					4.65532
XBP1	YLR179C	3.85858		0.301136						4.15972
XBP1	YLR204W	0.855564	0.752513	2.54628						4.15436
XBP1	YLR299W			3.80171			1.03981			4.84152
XBP1	YML053C	2.03235	0.752513	1.41928						4.20414
XBP1	YMR173W	4.8462		0.803399						5.6496
XBP1	YMR286W		0.752513	3.31469						4.0672
XBP1	YNL024C		0.752513	3.25373						4.00624
XBP1	YNL036W	3.09482		1.43137						4.52619
XBP1	YNL037C	3.09482		2.18507						5.27989
XBP1	YNL042W	0.801582	0.752513	3.71752						5.27161
XBP1	YNL058C		0.752513	4.60959						5.3621
XBP1	YNL087W	3.5552	0.752513	0.187729						4.49544
XBP1	YNL336W	4.26627								4.26627
XBP1	YNL339C	4.26627			0.343818					4.61009
XBP1	YPL068C		0.752513	3.88421						4.63672
XBP1	YPL262W	0.822966	0.752513	3.2718						4.84728
XBP1	YPR127W	2.5975			0.598372		1.27474			4.47061
XBP1	YPR173C	6.62968		2.0258						8.65548
XBP1	YPR174C	6.62968		0.359404	0.688085					7.67717
XBP1	YPR175W	6.62968		2.25856	0.688085					9.57632
YAP1	SNR55	4.07557								4.07557
YAP1	SNR57	4.07557								4.07557
YAP1	SNR61	4.07557								4.07557
YAP1	TF(GAA)N	5.88675	1.1155							7.00225
YAP1	TH(GUG)K	4.70241								4.70241
YAP1	TK(CUU)C	5.58662	0.579729							6.16635
YAP1	TR(UCU)E	5.86189								5.86189

TF	Target	Binding_0-2000	Motif_Score_SC	Other_Yeasts	Two-Hop_BE	Two-Hop_BP	Two-Hop_BY	Two-Hop_BG	Max_Membership	Sum
YAP1	YAL003W	1.58168			2.37278				0.72938	4.68384
YAP1	YBL002W	0.777831			1.83524	2.50759			0.658657	5.77932
YAP1	YBL027W	0.750579			2.62951		1.71476		0.722762	5.81761
YAP1	YBL034C	1.97464	0.579729	1.4301			0.872121			4.85659
YAP1	YBL041W				1.75128	3.34364	1.75459			6.84951
YAP1	YBL072C				2.18362		1.67074		0.72958	4.58394
YAP1	YBL087C	1.19528			2.64421				0.71817	4.55766
YAP1	YBL092W				2.42841		1.66261		0.731214	4.82223
YAP1	YBR008C	0.533044	0.349364	3.47003	0.470144		1.5925		0.698782	7.11386
YAP1	YBR009C	0.398071			2.31626	2.50759			0.66069	5.88261
YAP1	YBR030W	1.00659		0.436033	2.15973				0.711567	4.31392
YAP1	YBR031W			1.25178	2.4886		1.7178		0.712285	6.17047
YAP1	YBR048W	0.835771			2.46478		1.64397		0.732299	5.67682
YAP1	YBR084C-A				2.33383		1.71476		0.72904	4.77763
YAP1	YBR088C			2.23741	0.71015		1.71797		0.729644	5.39517
YAP1	YBR121C	1.8251			1.60818				0.71817	4.15145
YAP1	YBR143C	0.709068			1.6641		1.71391		0.731082	4.81816
YAP1	YBR154C	0.287593			2.24531		1.60496		0.70968	4.84754
YAP1	YBR181C				2.63972		1.60205		0.730727	4.9725
YAP1	YBR189W				2.52915		1.74057		0.732326	5.00205
YAP1	YBR191W	0.956438			2.40963		1.7178		0.725484	5.80935
YAP1	YBR283C			2.89663			1.75107			4.6477
YAP1	YBR284W			2.71375	1.51396					4.22771
YAP1	YCL035C				1.2736		0.844948	1.90474		4.02329
YAP1	YCR019W	5.58662	0.579729							6.16635
YAP1	YCR020C	2.37349		7.11583						9.48932
YAP1	YCR020W-B	0.355604		5.46365						5.81925
YAP1	YCR031C	0.69373			2.28156		1.74162		0.730243	5.44715
YAP1	YCR072C				2.29918	3.09856			0.558879	5.95662
YAP1	YCR090C			1.63395	2.4153					4.04925
YAP1	YCR102C	2.03654	1.1155	4.44359	1.35005		1.63689		0.731563	11.3141
YAP1	YDL020C	12.9961			0.635515					13.6316
YAP1	YDL025C	1.87318	0.579729	1.42807	1.06328					4.94426
YAP1	YDL045C			6.99566						6.99566
YAP1	YDL051W				1.25153	3.09856			0.723331	5.07342
YAP1	YDL061C			1.05205	2.10209		1.61324		0.729762	5.49714
YAP1	YDL083C				2.44639		1.69394		0.729148	4.86948
YAP1	YDL110C			3.96589	1.07507				0.728867	5.76983
YAP1	YDL142C	2.23914		1.78637						4.02551
YAP1	YDL182W	2.19112		0.173548	1.89834				0.59159	4.8546
YAP1	YDL201W		1.1155	1.5434	0.663773				0.728353	4.05103
YAP1	YDR003W	1.16601		2.32052	0.783333				0.542841	4.8127
YAP1	YDR011W	10.4805								10.4805
YAP1	YDR012W				2.66795		1.7178		0.721873	5.10762
YAP1	YDR014W	0.995671		5.96841			0.890539			7.85462
YAP1	YDR025W				2.43996		1.64397		0.708486	4.79242
YAP1	YDR036C				0.6853	2.50759	1.65843			4.85132
YAP1	YDR041W	3.31798	0.579729	5.04953			1.63895		0.491976	11.0782
YAP1	YDR064W	0.850837			1.72568		1.65576		0.69192	4.9242
YAP1	YDR101C				1.67365	3.09856			0.730334	5.50254
YAP1	YDR132C	3.4046	0.680656	4.42877	0.485153					8.99918
YAP1	YDR138W	1.6128		3.50393						5.11673
YAP1	YDR155C			1.07578	1.52143	2.50759				5.1048
YAP1	YDR224C	1.8993			0.667297	2.50759			0.690265	5.76445
YAP1	YDR225W	1.8993	1.1155		1.97159	2.50759			0.702449	8.19643
YAP1	YDR259C	5.04564	0.349364							5.395
YAP1	YDR261C	4.71898		0.292173						5.01115
YAP1	YDR339C	2.52071			2.13661				0.582466	5.23979
YAP1	YDR418W			0.245389	2.56769		1.61764		0.714979	5.1457
YAP1	YDR429C				1.85147		1.51202		0.717801	4.08129
YAP1	YDR447C				2.5638		1.6083		0.712227	4.88433
YAP1	YDR450W				2.6322		1.56472		0.718836	4.91576
YAP1	YDR496C				1.87735	2.50759			0.683905	5.06885
YAP1	YDR500C	1.09628			2.01435		1.7178		0.724669	5.5531
YAP1	YDR513W				2.02773		0.837362	2.2596	0.723167	5.84786
YAP1	YDR533C	10.0552	1.1155	4.18473	1.31357				0.729324	17.3983
YAP1	YEL034W			0.871061	1.74715	2.50759	1.74637		0.705969	7.57814
YAP1	YEL043W	5.6379	0.579729		1.34421					7.56184
YAP1	YEL044W	5.6379	0.579729	0.621502						6.83913
YAP1	YEL046C	5.6379	0.579729	0.643929						6.86156
YAP1	YEL054C			0.15307	1.86432		1.61764		0.729125	4.36415
YAP1	YEL071W	1.83205					1.56768		0.686639	4.08637
YAP1	YER006W	0.125649			1.43663	3.09856			0.690696	5.35154
YAP1	YER074W			0.0694499	1.835		1.56072		0.731944	4.19711
YAP1	YER078C	4.63036								4.63036
YAP1	YER079W	4.63036			1.39799				0.706268	6.73462
YAP1	YER080W	4.63036								4.63036
YAP1	YER082C				1.83858	1.2339	1.51202		0.696916	5.28142
YAP1	YER102W				2.1345		1.67074		0.732364	4.5376
YAP1	YER126C	0.119779			1.74828	3.09856			0.725942	5.69256
YAP1	YER131W	0.807603			2.52317				0.726933	4.05771
YAP1	YER146W				2.57775	0.836711	1.71997			5.13443
YAP1	YFL001W			5.25733						5.25733
YAP1	YFL055W	4.80389	0.680656		1.51459					6.99914
YAP1	YFL056C	4.80389	0.680656	1.01716	0.640154		0.790708	1.42474	0.73237	10.0897
YAP1	YFL057C	4.80389	0.680656		1.51454		1.25839	1.42474	0.716806	10.399
YAP1	YFR019W	2.38734					1.73709			4.12443
YAP1	YGL001C	1.70536		0.571769	0.439394	0.390718	1.40914		0.724161	5.24054
YAP1	YGL030W				2.48387		1.7258		0.73004	4.93971
YAP1	YGL068W					2.50759	1.73293			4.24052
YAP1	YGL070C		0.579729		1.9331		1.68532		0.605167	4.80332
YAP1	YGL076C				2.05896		1.64552		0.731243	4.43572
YAP1	YGL103W				2.53717		1.69972		0.688831	4.92572
YAP1	YGL120C				1.89759	2.50759			0.728691	5.13387
YAP1	YGL135W	2.59887			2.70647		1.56593		0.72901	7.60028
YAP1	YGL147C				2.47744		1.70573		0.732443	4.91561
YAP1	YGL189C			0.732037	2.69192				0.715155	4.13911
YAP1	YGR010W	2.9926			1.6585					4.6511
YAP1	YGR027C	1.31719			2.76207				0.724921	4.80418
YAP1	YGR034W				2.57069		1.60343		0.732578	4.9067
YAP1	YGR052W			3.93751					0.728157	4.66567
YAP1	YGR085C	0.290222			2.20615		1.57683		0.730334	4.80354
YAP1	YGR088W	1.77134		1.10795	1.34707				0.72862	4.95498
YAP1	YGR103W				1.49471	3.09856			0.723202	5.31647
YAP1	YGR118W				2.5554		1.74162		0.732026	5.02905
YAP1	YGR155W	0.766103	0.579729	2.78983						4.13566
YAP1	YGR195W	0.870853			1.87548		1.67619		0.583657	5.00618
YAP1	YGR209C	3.3631		0.914602	1.61833				0.721574	6.61761
YAP1	YGR211W	3.3631			1.95392		1.67074		0.732267	7.72003
YAP1	YGR214W				2.54642		1.75287		0.727092	5.02638

TF	Target	Binding_0-2000	Motif_Score_SC	Other_Yeasts	Two-Hop_BE	Two-Hop_BP	Two-Hop_BY	Two-Hop_BG	Max_Membership	Sum
YAP1	YGR220C	1.35996			1.73149	1.08629	1.57199			5.74973
YAP1	YGR233C	3.83985		0.937452						4.7773
YAP1	YGR234W	3.83985		1.46284	0.369609				0.730422	6.40272
YAP1	YGR245C				1.6185	3.09856			0.72532	5.44238
YAP1	YGR264C	0.316998		1.41587	1.92499				0.72865	4.38651
YAP1	YGR284C		0.579729	3.41878	0.470319		0.823178		0.700451	5.99246
YAP1	YHL015W			1.3143	2.516		1.74344		0.489647	6.06339
YAP1	YHL033C	0.476359	0.0992319		2.48305		1.67847		0.703449	5.44056
YAP1	YHL039W	1.58508	1.1155	10.9902	0.792867				0.716073	15.1997
YAP1	YHL040C	1.58508	1.1155	3.5841			1.63352	2.31655	0.726446	10.9612
YAP1	YHR043C	0.692637		1.1748	1.13284		1.25371			4.25399
YAP1	YHR048W	5.25126		1.76341			1.65922			8.67389
YAP1	YHR052W				1.98453	3.09856			0.713028	5.79612
YAP1	YHR085W				1.66724	3.09856				4.7658
YAP1	YHR141C				2.56462		1.56733		0.719218	4.85117
YAP1	YHR146W			4.60399						4.60399
YAP1	YHR148W	0.447384			1.85137		1.71892			4.01767
YAP1	YHR169W			2.30576	2.19906				0.709108	5.21393
YAP1	YHR170W		1.1155		1.72943	2.50759			0.730903	6.08342
YAP1	YHR179W	3.72444		3.21059	1.36527	2.50759			0.725971	11.5339
YAP1	YHR197W				1.70998	3.09856			0.725232	5.53377
YAP1	YHR203C				2.70875		1.59141		0.729283	5.02944
YAP1	YIL052C			1.39954	2.36901				0.717792	4.48634
YAP1	YIL070C		0.349364	2.9556		2.50759				5.81255
YAP1	YIL093C			2.30584		2.50759				4.81343
YAP1	YIL133C				2.1611		1.61658		0.732757	4.51044
YAP1	YIL153W		1.1155	5.62548						6.74098
YAP1	YIR012W				2.05445	3.2891			0.693181	6.03673
YAP1	YJL098W	11.5892								11.5892
YAP1	YJL100W	10.1615	1.1155	10.7976						22.0746
YAP1	YJL101C	10.1615	1.1155	11.5839	2.1163					24.9772
YAP1	YJL122W			1.57131	2.20437	2.50759			0.711596	6.99487
YAP1	YJL124C				2.2903	0.836711	1.51945			4.64646
YAP1	YJL177W			0.849894	2.58272		1.72531		0.724507	5.88243
YAP1	YJL189W				2.53203		1.52308		0.669908	4.72502
YAP1	YJL190C				2.74157		1.70648		0.72517	5.17322
YAP1	YJL191W				2.58661		1.74162		0.697259	5.02549
YAP1	YJL198W	1.1824			2.42682				0.495186	4.10441
YAP1	YJR002W			4.3097	1.61041				0.71761	6.63772
YAP1	YJR007W			1.51839	2.11808		1.59476		0.694006	5.92524
YAP1	YJR063W				2.04506		1.67996		0.73239	4.45741
YAP1	YJR084W	1.34291	0.680656	13.2679						15.2915
YAP1	YJR094W-A				1.68743		1.6083		0.710915	4.00664
YAP1	YJR104C	2.84766	0.680656	6.52573	1.68074	1.03926			0.715542	13.4896
YAP1	YJR105W	2.84766	0.680656	4.80817	1.90624					10.2427
YAP1	YJR122W	1.7698	1.1155	8.57402	1.93491				0.706178	14.1004
YAP1	YJR123W	2.13861			2.64157		1.68442		0.730495	7.1951
YAP1	YJR145C	0.210621	0.0992319		2.71958		1.59141		0.703229	5.32407
YAP1	YKL009W	0.571387			2.16221	3.09856	1.6169		0.729832	8.17889
YAP1	YKL067W	4.70241			0.53761					5.24002
YAP1	YKL081W	0.150095	0.579729	0.818832	2.67886	2.50759	0.853082		0.678841	8.26703
YAP1	YKL085W	2.51452	0.680656		0.620667	2.50759			0.583097	6.90653
YAP1	YKL086W	2.51452	0.680656	2.98198						6.17716
YAP1	YKL087C	2.51452	0.680656	2.42238	1.57591					7.19347
YAP1	YKL101W	0.279754	1.1155	4.15266		1.2339				6.78181
YAP1	YKL103C	0.279754	1.1155	6.05358	1.55358				0.731067	9.73348
YAP1	YKL113C	1.46012		0.916845			1.69727		0.488245	4.56248
YAP1	YKL122C			1.57884	2.33275				0.606188	4.51778
YAP1	YKL180W				2.45071		1.7353		0.727385	4.91339
YAP1	YKL187C		1.1155	10.2838					0.560153	11.9595
YAP1	YKR017C			5.12084						5.12084
YAP1	YKR052C	5.81514	0.680656	3.50143	1.07914					11.0764
YAP1	YKR056W	2.44041			1.6301				0.674417	4.74493
YAP1	YKR061W	0.888001	0.680656	3.92466						5.49332
YAP1	YKR071C	12.0407								12.0407
YAP1	YKR076W	0.903054	1.1155	4.43039	0.345446				0.732569	7.52696
YAP1	YKR081C				2.19217	3.09856			0.732094	6.02282
YAP1	YLL045C				2.52726		1.67847		0.732701	4.93843
YAP1	YLL050C				0.41287	2.50759	1.22693			4.14739
YAP1	YLL056C	1.12492	1.1155	1.73487			0.900988			4.87628
YAP1	YLL058W	5.39713	0.680656				1.68319			7.76098
YAP1	YLL060C	5.39713	0.680656	2.5408					0.73202	9.35061
YAP1	YLR009W				1.61495	2.50759			0.672695	4.79523
YAP1	YLR029C	0.595973			2.64951		1.65576		0.719649	5.62089
YAP1	YLR048W	1.0399		0.300868	2.50762		1.75161		0.729935	6.32993
YAP1	YLR060W				1.6647		1.68631		0.684477	4.03549
YAP1	YLR073C	12.7941	1.1155		1.20125				0.702997	15.8138
YAP1	YLR074C	12.7941	1.1155	9.36906					0.586315	23.865
YAP1	YLR075W	12.7941	1.1155	7.60953	0.704585				0.726646	22.9504
YAP1	YLR077W	12.7941	1.1155							13.9096
YAP1	YLR079W	6.59164			2.46824					9.05988
YAP1	YLR105C	4.50391								4.50391
YAP1	YLR108C	9.4603								9.4603
YAP1	YLR109W	9.4603			0.77769				0.732701	10.9707
YAP1	YLR185W				2.38574		1.7178		0.723478	4.82702
YAP1	YLR196W	1.1049			2.10535	2.50759			0.727884	6.44572
YAP1	YLR197W				1.76611		1.74637		0.729178	4.24166
YAP1	YLR200W			0.00105133	2.47602		1.75583			4.2329
YAP1	YLR256W	0.707274	0.680656		1.98062		1.37382			4.74237
YAP1	YLR264W				2.30994		1.61324		0.726769	4.64995
YAP1	YLR304C			2.46397			1.64603			4.11
YAP1	YLR340W			0.0967616	2.24869	1.60763	1.59316		0.718484	6.26473
YAP1	YLR344W	0.381167			2.34598		1.60343		0.687598	5.01818
YAP1	YLR367W	3.0589			2.69321		1.70648		0.725314	8.1839
YAP1	YLR388W		0.579729		2.55679		1.61324		0.7297	5.47946
YAP1	YLR438C-A	3.70962					1.68566			5.39528
YAP1	YLR441C				2.77374		1.60419		0.723744	5.10167
YAP1	YLR460C	4.36575	1.1155	1.41005	1.31828				0.731604	8.94118
YAP1	YLR461W	4.36575	1.1155							5.48125
YAP1	YML024W				1.6816		1.6083		0.725851	4.01575
YAP1	YML026C				2.76116		1.56472		0.719523	5.0454
YAP1	YML059C						1.5125	2.73727		4.24977
YAP1	YML063W				2.67878		1.60419		0.725291	5.00826
YAP1	YML115C	11.0487								11.0487
YAP1	YML116W	12.7356		1.00721	1.59046		0.821587		0.587553	16.7424
YAP1	YML129C		0.579729	3.42573	0.36746					4.37292
YAP1	YML130C	4.92525		0.416023	2.28418				0.559586	8.18504
YAP1	YML131W	3.73984	0.579729	0.27585	1.95224		0.885236		0.731528	8.16442
YAP1	YMR005W				1.85452	2.18232			0.576587	4.61343
YAP1	YMR015C	0.409662	0.579729	4.20444	1.79836				0.689761	7.68195

TF	Target	Binding_0-2000	Motif_Score_SC	Other_Yeasts	Two-Hop_BE	Two-Hop_BP	Two-Hop_BY	Two-Hop_BG	Max_Membership	Sum
YAP1	YMR017W		1.1155	7.02037						8.13587
YAP1	YMR038C	4.45992	0.680656	3.9433		0.604023			0.638186	10.3261
YAP1	YMR052W			5.87119						5.87119
YAP1	YMR074C				2.64034		1.5173			4.15764
YAP1	YMR121C				2.70099		1.65576		0.705884	5.06263
YAP1	YMR143W				2.17349		1.69394		0.730342	4.59777
YAP1	YMR173W	2.16136			1.27714				0.732196	4.1707
YAP1	YMR193W	5.33992	0.680656				0.887675			6.90825
YAP1	YMR194W	5.33992	0.680656		1.80984				0.713101	8.54352
YAP1	YMR258C	3.73064	0.349364		1.52227					5.60227
YAP1	YMR260C	0.138124			2.44742		1.54723		0.676582	4.80936
YAP1	YMR318C	1.77741	1.1155	8.93171	0.331102		0.8468			13.0025
YAP1	YNL002C	0.129947		0.261887	1.6332	3.09856			0.728562	5.85216
YAP1	YNL025C	4.6071	0.0992319	2.2363						6.94263
YAP1	YNL067W				2.31796		1.71141		0.674362	4.70373
YAP1	YNL069C				2.55316		1.67557		0.721758	4.95049
YAP1	YNL087W	3.89542	1.1155						0.656454	5.66737
YAP1	YNL110C				1.92842	3.09856			0.657625	5.68461
YAP1	YNL124W			3.56739	0.396661				0.640909	4.60496
YAP1	YNL132W	5.88675	1.1155		0.518529				0.586823	8.1076
YAP1	YNL134C	5.88675	1.1155	4.78584	2.16509		1.20261		0.730175	15.886
YAP1	YNL135C	5.88675	1.1155		0.532431		0.823474			8.35816
YAP1	YNL147W				2.437	0.836711	1.56481			4.83852
YAP1	YNL162W				2.38661		1.56733		0.729283	4.68322
YAP1	YNL175C		0.579729		1.65468	2.50759			0.693507	5.43551
YAP1	YNL178W				2.49023		1.68729		0.691676	4.8692
YAP1	YNL182C				1.82477	3.09856			0.728811	5.65214
YAP1	YNL209W	0.282846		1.73463	2.43513				0.693586	5.14619
YAP1	YNL231C	1.76183			2.44473				0.484149	4.69071
YAP1	YNL259C	1.16292		1.0273	2.1078					4.29802
YAP1	YNL302C				2.49602		1.7178		0.585831	4.79965
YAP1	YNL331C			4.13805	0.955708				0.71832	5.81208
YAP1	YNR053C				2.17621	3.09856				5.27477
YAP1	YOL031C	1.67699		0.38443	1.92537				0.693175	4.67997
YAP1	YOL040C	0.15709			2.60896		1.75666		0.722533	5.24524
YAP1	YOL041C	0.15709			0.927635	2.50759			0.631057	4.22337
YAP1	YOL049W	1.31933	0.579729	2.84168						4.74074
YAP1	YOL056W		0.680656	1.70769	2.04878					4.43713
YAP1	YOL117W	8.42789	1.1155							9.54339
YAP1	YOL119C	8.42789	1.1155		0.387955					9.93134
YAP1	YOL121C				2.49943		1.7178		0.719485	4.93671
YAP1	YOL124C	1.73468			2.10566		1.61534			5.45568
YAP1	YOL127W	0.313463			2.62738		1.5736		0.597161	5.1116
YAP1	YOL139C				2.1861	2.18232	1.38648			5.7549
YAP1	YOL151W	2.83933			1.73296		0.839051		0.731443	6.14278
YAP1	YOR020C	0.261217			2.30101		0.788331		0.678246	4.0288
YAP1	YOR063W		0.579729		2.44029		1.7474		0.711614	5.47903
YAP1	YOR128C	1.67916	0.579729	0.246655			1.54398			4.04952
YAP1	YOR145C			0.0500832	2.45443		1.7178		0.730093	4.95241
YAP1	YOR167C				2.2502		1.61324		0.728582	4.59202
YAP1	YOR231W		1.1155	8.02397	0.43191					9.57138
YAP1	YOR234C	2.51609			2.23493				0.726115	5.47714
YAP1	YOR285W			6.32307	1.73171		1.44546	2.20178		11.702
YAP1	YOR310C	0.194021			1.36851		1.74637		0.732414	4.04131
YAP1	YOR315W	3.04214			0.485971				0.730125	4.25824
YAP1	YOR362C			1.55955			1.73665		0.730386	4.02659
YAP1	YOR369C	0.749903			2.45491		1.53673		0.687214	5.42876
YAP1	YPL010W	0.969407	0.579729	1.733	1.80297					5.08511
YAP1	YPL013C					2.50759	1.57594			4.08353
YAP1	YPL042C		0.579729	2.77371		1.07899				4.43243
YAP1	YPL050C	0.443773	0.579729			2.81794			0.604578	4.44602
YAP1	YPL061W	0.692417	1.1155	10.0847					0.545643	12.4383
YAP1	YPL079W				2.12358		1.7178		0.732411	4.57379
YAP1	YPL081W				2.02305		1.74057		0.684685	4.4483
YAP1	YPL090C				2.2539		1.60205		0.706263	4.56221
YAP1	YPL091W	1.71735	0.680656	6.1405	1.24921					9.78772
YAP1	YPL093W		0.579729		1.75291	3.09856	1.76003		0.617897	7.80913
YAP1	YPL131W				2.14564		1.7178		0.655586	4.51903
YAP1	YPL160W				2.23193		1.75324		0.700307	4.68548
YAP1	YPL198W				1.9938		1.64552		0.725393	4.36471
YAP1	YPL202C	2.39872	0.349364	1.84893						4.59701
YAP1	YPL220W		0.0992319	0.376845	2.53892		1.56593		0.729597	5.31052
YAP1	YPL237W				1.64667		1.76003		0.732484	4.13918
YAP1	YPR016C	0.349249				3.09856			0.648765	4.09657
YAP1	YPR043W				2.40245		1.6083		0.726837	4.73759
YAP1	YPR048W	1.39256			1.73493		0.893794			4.02128
YAP1	YPR063C	11.5584	0.579729	1.28954						13.4277
YAP1	YPR065W	11.5584	0.579729	3.02747						15.1656
YAP1	YPR066W	11.5584	0.579729							12.1381
YAP1	YPR102C				2.68426		1.57683		0.730832	4.99192
YAP1	YPR110C	0.200805	0.349364		1.40186		1.71391		0.731017	4.39696
YAP1	YPR132W				2.46036		1.74162		0.732361	4.93434
YAP1	YPR166C	0.36936	0.579729	0.709094	2.59501		1.64222			5.89541
YAP1	YPR167C	0.36936	0.579729	3.6318	0.712959					5.29385
YAP1	YPR168W	0.36936	0.579729	3.7667						4.71579
YAP1	YPR169W	0.36936	0.579729	2.12416	1.87894					4.95219
YAP1	YPR190C		0.349364	1.20825	1.82398				0.692762	4.07436
YAP3	YBL001C	3.76459		0.437955						4.20254
YAP3	YBR102C	4.13987								4.13987
YAP3	YBR103W	4.13987								4.13987
YAP3	YGL001C	0.415289		3.63474						4.05003
YAP3	YGL189C			5.05069	0.455193					5.50588
YAP3	YGR109C			4.09924						4.09924
YAP3	YGR110W			4.69671						4.69671
YAP3	YHR170W			3.65692	0.661681					4.3186
YAP3	YJR138W			4.12547						4.12547
YAP3	YKL069W	0.0634616		3.99664						4.0601
YAP3	YKL071W	0.362623		4.24357						4.60619
YAP3	YKL107W			4.34166						4.34166
YAP3	YNL331C	0.368696		4.07633						4.44503
YAP3	YOL042W	0.941991		3.69216						4.63415
YAP3	YOL043C	0.941991		3.39089						4.33288
YAP3	YOL048C			4.85554						4.85554
YAP5	YBL028C	2.71733		1.01486	0.656414					4.3886
YAP5	YDL114W	4.04033								4.04033
YAP5	YDL115C	4.04033								4.04033
YAP5	YDR545W	5.18811							0.718352	5.90646
YAP5	YEL075C	3.6163			0.63971			1.50742	0.726713	6.49014
YAP5	YEL076C	3.6163			0.774576			1.38083	0.711355	6.48306
YAP5	YEL076C-A	3.6163			0.774576			1.38083		5.77171

TF	Target	Binding_0-2000	Motif_Score_SC	Other_Yeasts	Two-Hop_BE	Two-Hop_BP	Two-Hop_BY	Two-Hop_BG	Max_Membership	Sum
YAP5	YEL077C	3.6163			0.596427			1.38083		5.59356
YAP5	YER189W	2.80048			0.755366			1.50742	0.707991	5.77126
YAP5	YER190W	2.80048			0.922473			1.21074	0.727338	5.66103
YAP5	YFR001W	1.1488		2.12202	0.883714					4.15453
YAP5	YGL189C	1.20768		5.05069	0.681177					6.93955
YAP5	YGR109C			4.09924					0.579914	4.67915
YAP5	YGR110W			4.69671						4.69671
YAP5	YGR149W	1.93928		2.9761						4.91538
YAP5	YHR091C	5.16604								5.16604
YAP5	YHR170W			3.65692	0.458384					4.1153
YAP5	YIL018W	3.30823		0.0145276	0.89768					4.22044
YAP5	YJR138W			4.12547						4.12547
YAP5	YKL071W	0.628898		4.24357						4.87247
YAP5	YKL107W			4.34166						4.34166
YAP5	YLL066C	5.26268			0.419509			1.38082		7.06301
YAP5	YNL331C			4.07633						4.07633
YAP5	YNL336W	5.37396								5.37396
YAP5	YNL339C	5.37396		1.07137	0.807276				0.650123	7.90273
YAP5	YOL042W	0.644443		3.69216						4.3366
YAP5	YOL043C	0.981518		3.39089						4.37241
YAP5	YOL048C			4.85554						4.85554
YAP5	YOR319W	4.90845								4.90845
YAP5	YPR202W	4.56017			0.973511			1.50741	0.714327	7.75542
YAP5	YPR203W	4.56017			0.918098			1.38082	0.73168	7.59077
YAP6	SNR37	4.10187								4.10187
YAP6	SNR53	5.75874								5.75874
YAP6	TK(CUU)C	6.9073								6.9073
YAP6	TR(UCU)E	7.69927								7.69927
YAP6	YAL005C	2.69582			1.44284					4.13866
YAP6	YAL063C	5.39799								5.39799
YAP6	YAR002W				1.40635	0.823283		1.88735		4.11698
YAP6	YBL008W	0.180301			2.57638		1.99372			4.7504
YAP6	YBL026W	3.68256			1.7071				0.48603	5.87569
YAP6	YBL027W	3.23688			2.28905		1.47064		0.722762	7.71933
YAP6	YBL028C	3.23688		1.01486	1.15378				0.732035	6.13755
YAP6	YBL029C-A	15.2955								15.2955
YAP6	YBL029W	15.2955								15.2955
YAP6	YBL030C	3.6074			0.539835					4.14724
YAP6	YBL040C			0.92444	2.27383		1.978			5.17627
YAP6	YBL072C			0.20912	2.45316		1.40689		0.72958	4.79875
YAP6	YBL087C	2.1579		0.131925	2.42807				0.71817	5.43607
YAP6	YBL092W				2.00845		1.48251		0.731214	4.22217
YAP6	YBR025C	2.69582		0.517098	0.96166				0.687481	4.86206
YAP6	YBR031W				2.06626		1.45393		0.712285	4.23247
YAP6	YBR045C					4.11544	1.89131			6.00675
YAP6	YBR048W	0.874367		0.381193	2.42685		1.49819		0.732299	5.9129
YAP6	YBR084C-A				2.04247		1.47064		0.72904	4.24215
YAP6	YBR121C			2.82585	1.92825		1.2697		0.71817	6.74197
YAP6	YBR143C	0.0780855		0.338839	2.0297		1.42343		0.731082	4.60114
YAP6	YBR181C				2.38216		1.45393		0.730727	4.56682
YAP6	YBR189W	0.365036			2.36843		1.48953		0.732326	4.95532
YAP6	YBR191W	0.849111		0.032047	2.49316		1.36607		0.725484	5.46587
YAP6	YBR202W			1.91114		1.31677	1.49859			4.7265
YAP6	YBR260C					4.0153				4.0153
YAP6	YCR019W	6.9073								6.9073
YAP6	YCR031C			0.155627	1.73418		1.4987		0.730243	4.11875
YAP6	YCR102C	2.77759			0.766932		1.27052			4.81504
YAP6	YCR107W	4.23745			0.46872					4.70617
YAP6	YDL014W				2.02247		1.48251		0.631835	4.13682
YAP6	YDL037C	7.74895								7.74895
YAP6	YDL061C	0.814584			2.10057		1.34938		0.732364	4.9969
YAP6	YDL083C	1.30114			2.23758		1.48506		0.729148	5.75293
YAP6	YDL137W			2.56246			2.00338			4.56584
YAP6	YDL166C				2.5032		1.42797		0.651485	4.58265
YAP6	YDL201W			0.762613	1.53927		1.41072		0.728353	4.44096
YAP6	YDR025W			1.32978	2.42966		1.49819		0.708486	5.96612
YAP6	YDR041W	3.56454			0.340576		1.45719			5.36231
YAP6	YDR042C	3.14301		1.52078						4.66379
YAP6	YDR064W	0.281962		1.08406	2.18423		1.48251		0.732202	5.76496
YAP6	YDR077W	5.33933			0.329205					5.66854
YAP6	YDR084C	2.61193		0.226234		1.54483				4.38299
YAP6	YDR101C			2.08934	1.55888				0.730741	4.37896
YAP6	YDR152W	1.56541		1.85373	0.751834				0.678143	4.84912
YAP6	YDR245W	1.7759			2.03222				0.638148	4.44627
YAP6	YDR259C	5.86321								5.86321
YAP6	YDR418W			0.168268	2.39727		1.4763		0.714979	4.75682
YAP6	YDR441C	9.64308								9.64308
YAP6	YDR447C			0.629411	2.16461		1.45393		0.723017	4.97097
YAP6	YDR450W	0.240347			2.24824		1.36263		0.718836	4.57005
YAP6	YDR500C				2.13039		1.38747		0.724669	4.24253
YAP6	YDR524C	1.25133		1.81093				1.09189		4.15415
YAP6	YEL034W	0.193591		2.73964	1.48914		1.43088			5.85325
YAP6	YEL040W	2.93493			2.38937				0.66226	5.98656
YAP6	YEL050C	5.75874								5.75874
YAP6	YEL054C	0.620323			1.67987		1.4763		0.729125	4.50562
YAP6	YER007C-A	1.69365			1.39564		1.45393			4.54322
YAP6	YER055C			2.00215	1.4142				0.674329	4.09068
YAP6	YER074W	0.554447			2.46896		1.29685		0.731944	5.0522
YAP6	YER102W				2.47722		1.40689		0.732364	4.61647
YAP6	YER107C			0.93091	1.86727		1.99372		0.651438	5.44334
YAP6	YER117W				2.31299	1.66681			0.715709	4.69551
YAP6	YER133W			0.847576	1.37115	4.11544		1.47784		7.81201
YAP6	YER146W				1.26252	0.829407	1.44023		0.557424	4.08958
YAP6	YFL004W	4.81632								4.81632
YAP6	YFL029C			3.51687			0.836431			4.3533
YAP6	YGL001C	6.19371		3.63474	1.067					10.8955
YAP6	YGL029W	0.298713		2.22104	1.03556				0.581079	4.13639
YAP6	YGL030W	0.139371			2.42603		1.33649		0.73004	4.63193
YAP6	YGL031C	0.139371		1.6938	2.30876				0.72862	4.87055
YAP6	YGL073W					4.11544				4.11544
YAP6	YGL076C			0.426695	2.48473		1.45152		0.731243	5.09419
YAP6	YGL103W	0.463668		0.735621	2.21498		1.33106		0.654148	5.39948
YAP6	YGL135W	1.1412			2.43952		1.4507		0.72901	5.76043
YAP6	YGL147C				2.49652		1.45437		0.732443	4.68333
YAP6	YGL156W	3.7109			1.13087					4.84177
YAP6	YGL157W	3.7109		1.12797	0.484752				0.686322	6.00994
YAP6	YGL189C	0.0659851		5.05069	2.24874				0.715155	8.08057
YAP6	YGL225W				3.0433		1.89692			4.94022
YAP6	YGR027C	0.439811		0.800448	2.08463				0.724921	4.04981
YAP6	YGR034W				2.47215		1.41936		0.723809	4.61532

TF	Target	Binding_0-2000	Motif_Score_SC	Other_Yeasts	Two-Hop_BE	Two-Hop_BP	Two-Hop_BY	Two-Hop_BG	Max_Membership	Sum
YAP6	YGR085C	0.458507		0.599733	2.48164		1.42347		0.730334	5.69368
YAP6	YGR109C			4.09924	0.326442					4.42568
YAP6	YGR110W			4.69671						4.69671
YAP6	YGR118W				1.98487		1.48907		0.732026	4.20597
YAP6	YGR123C				1.60335		1.28459	1.38806	0.725214	5.00121
YAP6	YGR175C	2.39389			1.19258		0.81774		0.49966	4.90387
YAP6	YGR200C				0.989977	0.531348	1.97241		0.727517	4.22125
YAP6	YGR209C	1.59694		2.99161	0.415963					5.00451
YAP6	YGR214W				2.44584		1.49678		0.726502	4.66912
YAP6	YGR234W	2.99299		0.386079	0.539953				0.730422	4.64944
YAP6	YGR238C	5.66094					1.38707			7.04801
YAP6	YGR249W	7.51512								7.51512
YAP6	YHL033C				2.20192		1.48706		0.703449	4.39243
YAP6	YHR021C				1.89277		1.45393		0.715788	4.06249
YAP6	YHR141C				2.2122		1.43709		0.719218	4.36851
YAP6	YHR170W			3.65692	0.801691				0.730903	5.18951
YAP6	YHR179W	2.10633		2.88378					0.632993	5.6231
YAP6	YHR203C			0.157853	2.33614		1.45393		0.729283	4.67721
YAP6	YIL045W				2.83475	3.2218				6.05655
YAP6	YIL053W				1.56145		2.05097		0.611275	4.2237
YAP6	YIL099W	6.41629			0.721975					7.13827
YAP6	YIL101C	6.41629			0.721975					7.13827
YAP6	YIL118W	15.4841								15.4841
YAP6	YIL119C	15.4841								15.4841
YAP6	YIL133C			0.196745	2.31369		1.49568		0.732757	4.73887
YAP6	YIR019C	15.8551		0.256695						16.1118
YAP6	YIR021W	4.83844								4.83844
YAP6	YJL002C	1.68333					1.978		0.491912	4.15324
YAP6	YJL080C				1.90014		2.00007		0.712285	4.6125
YAP6	YJL148W	2.32382			1.45401				0.7273	4.50513
YAP6	YJL177W			0.721206	2.49028		1.44082		0.720036	5.37234
YAP6	YJL189W	1.82616			1.58578		1.34938		0.721412	5.48273
YAP6	YJL190C	0.178229			2.26559		1.48756		0.72517	4.65655
YAP6	YJL191W	1.85059			1.79409		1.4987		0.7041	5.84748
YAP6	YJL214W	6.2284								6.2284
YAP6	YJL216C	6.2284								6.2284
YAP6	YJL217W	5.3309								5.3309
YAP6	YJR010C-A	5.37261		0.181211						5.55382
YAP6	YJR011C	5.37261								5.37261
YAP6	YJR013W	5.37261								5.37261
YAP6	YJR094C	13.5148		0.0749886						13.5898
YAP6	YJR094W-A	3.94651			1.91733		1.38747		0.710915	7.96223
YAP6	YJR095W	6.94192								6.94192
YAP6	YJR123W				2.45256		1.48872		0.730495	4.67178
YAP6	YJR138W			4.12547						4.12547
YAP6	YJR145C	6.07864		0.624458	2.21133		1.45393		0.703229	11.0716
YAP6	YJR147W	6.07864					0.917405			6.99605
YAP6	YKL018W					2.48601	1.95327			4.43928
YAP6	YKL043W	9.70162								9.70162
YAP6	YKL050C	1.20784		0.343415	2.44954					4.0008
YAP6	YKL067W	2.89678		1.43834	0.827454					5.16257
YAP6	YKL071W	0.3651		4.24357						4.60867
YAP6	YKL081W			0.85744	1.94854	0.372501	1.94967		0.715152	5.8433
YAP6	YKL107W			4.34166						4.34166
YAP6	YKL143W				1.19853	2.30515			0.619683	4.12336
YAP6	YKL180W				2.05289		1.40414		0.727385	4.18441
YAP6	YKL205W			2.65407	0.799837	0.823283			0.653884	4.93107
YAP6	YKL217W				2.52483		1.92866			4.45349
YAP6	YKR036C					3.18287	0.829794			4.01266
YAP6	YLL045C				2.27093		1.48706		0.732701	4.49069
YAP6	YLR029C	0.696638			2.21671		1.48251		0.719649	5.11551
YAP6	YLR047C	4.29484								4.29484
YAP6	YLR048W	4.29484		1.42303	2.26963		1.49279		0.729935	10.2102
YAP6	YLR075W	1.97659			2.03116		1.42343		0.726646	6.15783
YAP6	YLR081W	4.22257								4.22257
YAP6	YLR121C	4.96535			0.874619					5.83997
YAP6	YLR185W				2.16598		1.38747		0.723478	4.27693
YAP6	YLR196W	0.112364			1.58059		2.00926		0.727884	4.4301
YAP6	YLR258W				0.89303	4.11544				5.00847
YAP6	YLR264W			0.229722	2.07983	0.385111	1.34938		0.726769	4.77081
YAP6	YLR293C	3.65987			2.35887				0.661538	6.68028
YAP6	YLR295C	3.65987			0.520788					4.18066
YAP6	YLR297W	3.65987		1.21772						4.87759
YAP6	YLR330W			0.00514648	2.5644			1.5951		4.16465
YAP6	YLR344W				2.38766		1.41936		0.730782	4.5378
YAP6	YLR367W	1.06946			1.78367		1.48756		0.725314	5.066
YAP6	YLR388W				2.23407		1.34938		0.7297	4.31315
YAP6	YLR409C				1.57267		1.97092		0.725335	4.26893
YAP6	YLR413W	3.47615			0.519072				0.712969	4.70819
YAP6	YLR441C				2.47518		1.48567		0.723744	4.68459
YAP6	YML024W				2.29133		1.45393		0.729339	4.4746
YAP6	YML026C				2.2018		1.36263		0.730894	4.29532
YAP6	YML063W				2.23693		1.48567		0.730694	4.45329
YAP6	YML116W	9.6151								9.6151
YAP6	YML131W	3.67882			0.39463					4.07345
YAP6	YMR016C	2.89186		3.9849						6.87676
YAP6	YMR121C				2.0793		1.48251		0.729028	4.29084
YAP6	YMR143W				2.46056		1.48506		0.730342	4.67596
YAP6	YMR193W	4.45499			0.95031					5.4053
YAP6	YMR194W	4.45499			1.9889				0.731663	7.17555
YAP6	YMR223W				2.81667		1.89692			4.71359
YAP6	YMR239C	3.42347		2.74499	1.07607				0.712151	7.95668
YAP6	YMR258C	4.57614		0.00497938						4.58112
YAP6	YMR319C	9.67951								9.67951
YAP6	YNL067W				2.39431		1.46076		0.69605	4.55112
YAP6	YNL069C			0.369348	2.46125		1.49717		0.721758	5.04953
YAP6	YNL072W			2.73393			1.48913		0.730518	4.95358
YAP6	YNL087W	4.87518							0.715023	5.5902
YAP6	YNL119W			1.11145	2.45021				0.718631	4.28029
YAP6	YNL132W	2.16013			1.72306				0.704872	4.58806
YAP6	YNL148C	2.38429		1.67204						4.05633
YAP6	YNL162W				2.29035		1.43709		0.729283	4.45672
YAP6	YNL178W	10.5467		0.805136	1.29862		0.929349		0.69735	14.2772
YAP6	YNL180C	10.5467					0.813447			11.3601
YAP6	YNL264C				2.2576		1.89692			4.15452
YAP6	YNL302C	0.524724			1.49845		1.45393		0.680692	4.1578
YAP6	YNL331C			4.07633	0.367708					4.44404
YAP6	YNR060W	7.02285			0.897635					7.92049
YAP6	YOL039W	0.197173			2.11666		1.25524		0.713113	4.28219
YAP6	YOL040C				2.36773		1.49988		0.722533	4.59014

TF	Target	Binding_0-2000	Motif_Score_SC	Other_Yeasts	Two-Hop_BE	Two-Hop_BP	Two-Hop_BY	Two-Hop_BG	Max_Membership	Sum
YAP6	YOL048C			4.85554						4.85554
YAP6	YOL054W			1.77273		3.33393				5.10666
YAP6	YOL121C	1.95677			2.34929		1.45393		0.719485	6.47947
YAP6	YOL127W			0.422567	2.27945		1.48712		0.597161	4.7863
YAP6	YOL156W	15.8065								15.8065
YAP6	YOL157C	15.8065								15.8065
YAP6	YOR001W	6.42008			0.617256					7.03734
YAP6	YOR028C	9.65618								9.65618
YAP6	YOR030W	9.65618								9.65618
YAP6	YOR032C	1.91315			2.58637					4.49952
YAP6	YOR049C	7.29636								7.29636
YAP6	YOR119C			2.0955	0.539526		1.48833			4.12336
YAP6	YOR167C	1.174			2.28531	0.385111	1.34938		0.728582	5.92238
YAP6	YOR178C	15.8551								15.8551
YAP6	YOR179C	9.81922								9.81922
YAP6	YOR181W	3.34116			0.782999					4.12416
YAP6	YOR234C	3.02636			1.90355				0.726115	5.65602
YAP6	YOR272W	1.30429			1.12966		1.96348		0.720089	5.11752
YAP6	YOR285W	3.34101			0.709634					4.05064
YAP6	YOR312C			0.819362	2.46024				0.730976	4.01058
YAP6	YOR313C	6.23713		0.586323	0.885865					7.70932
YAP6	YOR315W	6.23713			1.3827				0.65267	8.2725
YAP6	YOR319W	2.94929					1.38707			4.33636
YAP6	YOR369C	2.10905			2.46452		1.35554		0.701818	6.63093
YAP6	YPL050C				2.85173	0.714462			0.604578	4.17077
YAP6	YPL079W				2.36968		1.36607		0.732411	4.46816
YAP6	YPL081W				2.07455		1.48953		0.715401	4.27948
YAP6	YPL090C				2.42003		1.45393		0.706263	4.58022
YAP6	YPL131W				2.32331		1.45393		0.684891	4.46213
YAP6	YPL143W	1.82571			2.22182				0.731055	4.77858
YAP6	YPL177C	0.609639		1.50446	2.38353		1.978			6.47563
YAP6	YPL198W				2.49242		1.45152		0.725393	4.66933
YAP6	YPL220W				2.49914		1.4507		0.729597	4.67944
YAP6	YPL273W			0.563572	1.31341		1.99328		0.722313	4.59258
YAP6	YPR041W				2.09894		1.36607		0.725839	4.19085
YAP6	YPR043W				2.20331		1.38747		0.726837	4.31762
YAP6	YPR046W			1.99821	2.54339					4.5416
YAP6	YPR093C				2.36416		1.978			4.34216
YAP6	YPR102C				2.50502		1.42347		0.730832	4.65932
YAP6	YPR110C			0.0688803	1.46138	0.646528	1.47009		0.731017	4.3779
YAP6	YPR118W	0.914546		0.478066	1.5549		1.30442		0.696179	4.94811
YAP6	YPR124W	1.0481		2.3946	1.21269					4.65539
YAP6	YPR132W				2.29241		1.48907		0.732361	4.51384
YAP6	YPR148C	8.97909								8.97909
YAP6	YPR149W	8.97909		0.635857						9.61495
YAP6	YPR183W				2.78032	0.359959	0.897642			4.03792
YAP7	TE(CUC)D	4.24974								4.24974
YAP7	TF(GAA)N	5.77052								5.77052
YAP7	TK(CUU)C	4.09099								4.09099
YAP7	TR(UCU)E	4.28862								4.28862
YAP7	YAL002W			4.03114						4.03114
YAP7	YAL012W	3.94159		0.150974						4.09256
YAP7	YAL060W			3.69168	0.867709					4.55939
YAP7	YBL027W	3.76091			1.32344		1.0057		0.535102	6.62515
YAP7	YBL028C	3.76091		1.25514	1.16212				0.726886	6.90506
YAP7	YBR008C	2.10603		7.17316	0.522339		0.959034		0.698782	11.4593
YAP7	YBR045C	6.54579								6.54579
YAP7	YBR046C	6.54579								6.54579
YAP7	YBR047W	6.54579								6.54579
YAP7	YBR048W	6.54579			0.976159		0.812599			8.33455
YAP7	YBR173C	3.66064			0.504344				0.731055	4.89604
YAP7	YBR176W	3.66064			0.401379					4.06202
YAP7	YBR179C	4.32685		0.0776149						4.40446
YAP7	YBR180W	4.32685		0.0728919						4.39974
YAP7	YBR254C	4.43668		0.147651						4.58433
YAP7	YBR255W	4.43668								4.43668
YAP7	YCR019W	4.09099		0.676493						4.76748
YAP7	YCR102C	3.10098		2.28698	0.716482		1.00343		0.731563	7.83943
YAP7	YDL020C	4.2776		0.599773						4.87737
YAP7	YDL124W	2.10982		1.07263	0.424174		0.894549		0.725739	5.22691
YAP7	YDL204W	3.49235			0.889601				0.515634	4.89759
YAP7	YDL210W	1.79372		3.2448			0.849971			5.88849
YAP7	YDL237W	4.81739			0.441992					5.25938
YAP7	YDL239C	4.81739								4.81739
YAP7	YDL243C	3.05302		2.14575						5.19877
YAP7	YDR032C			6.05165	0.626478					6.67813
YAP7	YDR054C	1.43992		5.68984						7.12976
YAP7	YDR055W	1.43992		7.60996						9.04988
YAP7	YDR063W			5.94252						5.94252
YAP7	YDR110W	0.112511		5.21658						5.32909
YAP7	YDR132C	6.18884		5.40985	0.987507					12.5862
YAP7	YDR135C	4.46452		2.71441						7.17893
YAP7	YDR137W	4.46452		3.58591						8.05043
YAP7	YDR181C	4.52321								4.52321
YAP7	YDR182W	4.52321								4.52321
YAP7	YDR182W-A	4.52321								4.52321
YAP7	YDR196C	3.95083					0.925872			4.8767
YAP7	YDR279W	4.24974			0.470809					4.72055
YAP7	YDR280W			4.37631						4.37631
YAP7	YDR353W			1.73952	0.629542			1.16878	0.564357	4.1022
YAP7	YDR485C	3.65682						1.12415		4.78097
YAP7	YDR513W			4.29577	0.835398			1.54269	0.626856	7.30071
YAP7	YDR533C	4.16432		1.14537	1.28985				0.729324	7.32886
YAP7	YEL050C	2.94737			1.06957					4.01694
YAP7	YER045C			5.54883						5.54883
YAP7	YER089C	4.15886								4.15886
YAP7	YER090W	4.15886						0.893086		5.05195
YAP7	YER141W	5.35122		1.70164	0.575409					7.62827
YAP7	YER148W	2.9677		0.73477	0.78436	0.944898	0.93637			6.3681
YAP7	YFL055W	5.50892			1.48827					6.99719
YAP7	YFL056C	5.50892		0.559419	0.61641		0.873485	1.55179	0.73237	9.84239
YAP7	YFL057C	5.50892			1.48827		0.873485	1.55179	0.716806	10.1393
YAP7	YGL048C	1.74281		0.899652	0.686424	0.755168				4.08405
YAP7	YGL067W			4.21772						4.21772
YAP7	YGL114W	4.76656		0.556791			0.858145			6.1815
YAP7	YGL135W	1.39042			1.59891		1.01393			4.00326
YAP7	YGL202W	1.033		2.70373			0.842211			4.57894
YAP7	YGL252C	3.01695		0.390581	0.635071	1.20253	0.796807			6.04194
YAP7	YGR009C	3.82905						1.09312		4.92217
YAP7	YGR010W	3.82905			0.71411					4.54316

TF	Target	Binding_0-2000	Motif_Score_SC	Other_Yeasts	Two-Hop_BE	Two-Hop_BP	Two-Hop_BY	Two-Hop_BG	Max_Membership	Sum
YAP7	YGR040W	4.27011								4.27011
YAP7	YGR100W			4.28115						4.28115
YAP7	YGR113W			5.38831						5.38831
YAP7	YGR181W	1.16399		5.00908						6.17307
YAP7	YGR207C	5.04083			0.627577					5.66841
YAP7	YGR209C	5.04083		8.64319	1.0242				0.721574	15.4298
YAP7	YGR211W	5.04083					0.937139			5.97797
YAP7	YGR233C	4.24965								4.24965
YAP7	YGR234W	4.24965		1.02225	0.412798					5.6847
YAP7	YHL040C	1.00156					1.00006	1.34433	0.726446	4.0724
YAP7	YHR015W	1.60483		4.64806						6.25289
YAP7	YHR028C	4.57117								4.57117
YAP7	YHR029C	4.57117							0.677671	5.24884
YAP7	YHR048W	3.76425		1.63906			1.02575			6.42906
YAP7	YHR111W	4.37176								4.37176
YAP7	YHR179W	2.85316		0.677939	1.03142		0.796049		0.725971	6.08454
YAP7	YHR198C	4.11334			1.02629					5.13963
YAP7	YHR199C	4.11334		0.192219		0.414572				4.72013
YAP7	YHR200W	4.11334		0.638789	0.443788					5.19592
YAP7	YIL044C			2.79217	0.651503			1.31373		4.7574
YAP7	YIL142W	0.567329		5.05497	0.781878					6.40418
YAP7	YIL143C	0.567329		4.3705						4.93783
YAP7	YJL100W	5.53832								5.53832
YAP7	YJL101C	5.53832			1.01415					6.55247
YAP7	YJL134W			4.2844						4.2844
YAP7	YJL163C			5.44421					0.723167	6.16738
YAP7	YJR030C			4.02184						4.02184
YAP7	YJR104C	3.21368		3.23609	0.477376	1.34956				8.27671
YAP7	YJR105W	3.21368		1.65683	0.577253					5.44776
YAP7	YJR109C	3.77981				0.350557				4.13037
YAP7	YJR122W	4.56804			0.832755					5.40079
YAP7	YJR150C			5.8358						5.8358
YAP7	YKL039W	4.63909								4.63909
YAP7	YKL040C	4.63909					0.825171			5.46426
YAP7	YKL067W	3.86452			1.02866					4.89318
YAP7	YKL071W	4.19714		1.0511	1.39437					6.64261
YAP7	YKL085W	6.69971			0.449512					7.14922
YAP7	YKL086W	6.69971		1.83002						8.52973
YAP7	YKL087C	6.69971		3.18044	0.763203					10.6434
YAP7	YKL103C	2.27974			0.685951	0.758513			0.731067	4.45527
YAP7	YKL187C			5.00685						5.00685
YAP7	YKR052C	3.3222		1.34541	0.739183					5.40679
YAP7	YKR061W	3.34996		1.09653						4.44649
YAP7	YKR071C	5.68676		0.608151	0.494769					6.78968
YAP7	YKR098C	4.80115								4.80115
YAP7	YKR099W	4.80115								4.80115
YAP7	YLL052C	3.27914						0.967002		4.24614
YAP7	YLL053C	3.27914						0.967002		4.24614
YAP7	YLL058W	4.12812					1.04972			5.17784
YAP7	YLL060C	4.12812		1.20196	0.341462				0.73202	6.40356
YAP7	YLR048W	2.09775			1.68042		1.02151			4.79968
YAP7	YLR075W	2.4559			1.18841		0.910372			4.55468
YAP7	YLR093C			8.01353						8.01353
YAP7	YLR108C	5.48177		1.38402	1.012				0.712379	8.59017
YAP7	YLR109W	5.48177		1.54065	0.753947				0.732701	8.50907
YAP7	YLR215C	0.692721		4.48903						5.18175
YAP7	YLR254C	3.37628		2.29389			0.793212			6.46338
YAP7	YLR299W	2.96997			0.338752				0.725112	4.03383
YAP7	YLR346C			8.67392						8.67392
YAP7	YLR460C	3.01157		1.35187	0.734744				0.731604	5.82979
YAP7	YML026C			2.76658	1.52767		0.989688			5.28394
YAP7	YML116W	7.34023		0.124299	0.64607				0.587553	8.69815
YAP7	YML129C			4.41456	1.22238					5.63694
YAP7	YML130C			7.61622	0.503218				0.701707	8.82115
YAP7	YML131W	4.77596			1.08474		0.898819		0.731528	7.49105
YAP7	YMR016C			8.78265						8.78265
YAP7	YMR038C	5.60938		3.90856		0.702833			0.638186	10.859
YAP7	YMR090W	1.86023		0.90656	0.516418				0.72016	4.00337
YAP7	YMR193W	5.56851			0.488197		0.927338			6.98404
YAP7	YMR194W	5.56851		0.0318724	1.17579					6.77617
YAP7	YMR308C			4.65867	0.719543	0.636428				6.01464
YAP7	YMR318C	3.66663					0.954259			4.62089
YAP7	YNL132W	5.77052			1.06618	0.459191				7.29589
YAP7	YNL134C	5.77052			0.961741				0.730175	7.46244
YAP7	YNL135C	5.77052			0.875147		0.903518			7.54919
YAP7	YNL176C	5.09277								5.09277
YAP7	YNL231C	1.6481		4.64515	0.347114				0.484149	7.12451
YAP7	YNL260C	4.35602		1.05175						5.40777
YAP7	YNL261W	5.04884								5.04884
YAP7	YNL284C			1.91233		1.148	0.961946			4.02228
YAP7	YOL113W	3.8222		0.229722						4.05192
YAP7	YOL151W	2.08723			1.25813		0.825404		0.731443	4.90221
YAP7	YOR028C			4.86351						4.86351
YAP7	YOR120W			2.53988	0.385288		0.904252		0.679223	4.50864
YAP7	YOR178C	1.75491				1.60233	0.798315			4.15555
YAP7	YOR224C	3.67491			0.561492					4.2364
YAP7	YOR226C	3.67491			0.386066				0.728139	4.78911
YAP7	YOR303W	1.90873				0.85566		1.29855		4.06294
YAP7	YOR362C			5.13742			1.01411		0.730386	6.88192
YAP7	YPL091W	5.59492		10.0488	0.675799					16.3195
YAP7	YPL123C	0.802853		3.23001						4.03286
YAP7	YPL170W	5.58674		1.47778						7.06452
YAP7	YPL171C	5.58674		1.45732			0.796049			7.84011
YAP7	YPL207W	1.29624		1.90419	1.09455					4.29498
YAP7	YPR007C	0.423828		4.33332						4.75715
YAP7	YPR008W	0.423828		8.16867				1.01163		9.60413
YAP7	YPR048W	5.96956		2.59394	1.02551		0.880148			10.4692
YAP7	YPR067W	4.23079		2.40737						6.63816
YAP7	YPR085C	4.11037								4.11037
YAP7	YPR086W	4.11037				0.670544	1.01277			5.79368
YAP7	YPR166C	3.682			0.483196					4.1652
YAP7	YPR167C	3.682			0.436338				0.575877	4.69421
YAP7	YPR169W	3.682			0.57186					4.25386
YDR026C	YBL036C		1.60139	6.90701						8.5084
YDR026C	YBL086C		2.13721	5.02626						7.16347
YDR026C	YBR035C		2.24294	5.94256						8.1855
YDR026C	YBR179C		2.13721	8.42673						10.5639
YDR026C	YBR180W		2.13721	9.07941						11.2166
YDR026C	YBR198C		2.24294	7.2817						9.52464
YDR026C	YBR199W		2.24294	6.85731						9.10025

TF	Target	Binding_0-2000	Motif_Score_SC	Other_Yeasts	Two-Hop_BE	Two-Hop_BP	Two-Hop_BY	Two-Hop_BG	Max_Membership	Sum
YDR026C	YCR095C		1.60139	3.04396						4.64535
YDR026C	YDL040C		0.00972326	4.74742						4.75714
YDR026C	YDL045W-A		1.60139	5.77595						7.37734
YDR026C	YDL085C-A		2.24294	11.4217						13.6646
YDR026C	YDL085W		2.24294	13.6109						15.8538
YDR026C	YDL212W		2.24294	10.6021						12.845
YDR026C	YDL213C		2.24294	12.9607						15.2036
YDR026C	YDR016C		2.13721	7.42709						9.5643
YDR026C	YDR101C		2.13721	5.7888	0.673019					8.59903
YDR026C	YDR103W		2.13721	4.14552						6.28273
YDR026C	YDR143C		2.24294	5.42716						7.6701
YDR026C	YDR298C		0.606046	8.26294						8.86899
YDR026C	YDR299W		0.606046	6.72716	0.424431					7.75764
YDR026C	YDR313C		1.60139	2.98267						4.58406
YDR026C	YDR378C		0.606046	4.53419						5.14024
YDR026C	YDR379W		0.606046	4.71344						5.31949
YDR026C	YDR498C		2.13721	5.15647						7.29368
YDR026C	YDR499W		2.13721	7.99133						10.1285
YDR026C	YEL026W		1.60139	2.58981						4.1912
YDR026C	YEL055C		2.24294	6.47988						8.72282
YDR026C	YER048W-A		1.60139	4.20942						5.81081
YDR026C	YER062C		1.71751	2.75238						4.46989
YDR026C	YER063W		1.71751	3.50136						5.21887
YDR026C	YER091C		2.13721	2.42952						4.56673
YDR026C	YER092W		2.13721	3.93116						6.06837
YDR026C	YFL005W		1.71751	2.48895						4.20646
YDR026C	YFL036W			4.16963						4.16963
YDR026C	YGL006W		2.24294	7.34161						9.58455
YDR026C	YGL098W		1.82095	2.9371						4.75805
YDR026C	YGL119W		1.60139	4.24285						5.84424
YDR026C	YGL120C		1.60139	4.15729	0.77648					6.53516
YDR026C	YGL160W		2.24294	3.50516						5.7481
YDR026C	YGL161C		2.24294	7.87469		0.532662				10.6503
YDR026C	YGL237C		1.45401	4.01436						5.46837
YDR026C	YGR024C		2.13721	3.09834						5.23555
YDR026C	YGR026W		2.13721	4.35333						6.49054
YDR026C	YGR150C			4.11169						4.11169
YDR026C	YGR179C		1.82095	3.88915						5.7101
YDR026C	YGR218W		1.60139	3.62917		0.492057				5.72262
YDR026C	YGR232W		1.57263	2.47742						4.05005
YDR026C	YGR237C		2.24294	7.40756						9.6505
YDR026C	YGR243W			5.22341						5.22341
YDR026C	YHL004W		2.24294	10.6493						12.8922
YDR026C	YHL007C		2.24294	6.824						9.06694
YDR026C	YIR013C		0.683669	4.01874						4.70241
YDR026C	YJL106W		2.24294	1.8045						4.04744
YDR026C	YJR098C		1.60139	2.57072						4.17211
YDR026C	YJR099W		1.60139	4.15637						5.75776
YDR026C	YKL142W		0.683669	7.08548						7.76915
YDR026C	YKL145W		2.13721	2.70702						4.84423
YDR026C	YKL203C		2.13721	3.32521						5.46242
YDR026C	YKR011C		1.71751	3.38611						5.10362
YDR026C	YKR068C		0.940368	3.89255						4.83292
YDR026C	YKR069W		0.940368	3.11329						4.05366
YDR026C	YLL049W		1.60139	2.70367						4.30506
YDR026C	YLR457C		2.13721	11.0555	0.390097					13.5828
YDR026C	YLR459W		2.13721	8.65004						10.7873
YDR026C	YML041C		1.60139	3.78137						5.38276
YDR026C	YML102W		1.45401	5.53406						6.98807
YDR026C	YML103C		1.45401	2.84982						4.30383
YDR026C	YML121W		0.727467	7.5416						8.26907
YDR026C	YML123C		0.727467	6.51586						7.24333
YDR026C	YMR001C		0.940368	3.84848						4.78885
YDR026C	YMR002W		0.940368	4.74762						5.68799
YDR026C	YMR023C		2.24294	1.8651						4.10804
YDR026C	YMR024W		2.24294	1.76618						4.00912
YDR026C	YMR039C		1.60139	3.34009						4.94148
YDR026C	YMR040W		1.60139	4.25101						5.8524
YDR026C	YMR123W		2.24294	7.89205						10.135
YDR026C	YMR124W		2.24294	2.54447						4.78741
YDR026C	YMR140W		0.940368	3.7298						4.67017
YDR026C	YMR257C		1.60139	8.83544						10.4368
YDR026C	YMR281W		1.93221	2.21553						4.14774
YDR026C	YMR290C		2.24294	2.7813	0.767837					5.79208
YDR026C	YMR291W		2.24294	2.18439						4.42733
YDR026C	YNL078W		2.24294	3.79711						6.04005
YDR026C	YNL079C		2.24294	3.93992						6.18286
YDR026C	YNL090W		1.57263	2.43715						4.00978
YDR026C	YNL099C		2.24294	11.1994						13.4423
YDR026C	YNL132W		2.13721	2.71042	0.399701					5.24733
YDR026C	YNL133C		2.13721	3.94973						6.08694
YDR026C	YNL308C		1.60139	4.23987	0.374488					6.21575
YDR026C	YNR012W		1.82095	2.8145						4.63545
YDR026C	YOL005C		1.60139	3.29297						4.89436
YDR026C	YOL092W		2.13721	2.50577						4.64298
YDR026C	YOL109W		1.30863	5.31333						6.62196
YDR026C	YOL111C		1.30863	5.13412						6.44275
YDR026C	YOR196C		1.60139	3.97389						5.57528
YDR026C	YOR197W		1.60139	3.94082						5.54221
YDR026C	YOR271C		1.93221	4.97599						6.9082
YDR026C	YOR272W		1.93221	2.52709						4.4593
YDR026C	YPL078C		1.71751	2.44357						4.16108
YDR026C	YPL215W		2.24294	7.52292						9.76586
YDR026C	YPL255W		2.24294	6.91493						9.15787
YDR026C	YPL256C		2.24294	10.8431						13.086
YDR026C	YPL260W		0.00972326	6.62188						6.6316
YDR026C	YPR025C		1.60139	4.6758						6.27719
YDR026C	YPR026W		1.60139	2.76285						4.36424
YDR026C	YPR067W			4.01882						4.01882
YDR026C	YPR181C		1.82095	2.39039		0.5048				4.71614
YDR026C	YPR182W		1.82095	3.3837						5.20465
YDR026C	YPR183W		1.82095	2.57701						4.39796
YDR520C	YBR286W	4.09729								4.09729
YHP1	TC(GCA)B	5.8924								5.8924
YHP1	TE(UUC)B	5.8924								5.8924
YHP1	YBR210W	5.8924								5.8924
YHP1	YDR190C	5.09805								5.09805
YHP1	YDR191W	5.09805								5.09805
YHP1	YLR312C	3.72685			0.339448					4.0663
YHP1	YMR270C	4.43878								4.43878

TF	Target	Binding_0-2000	Motif_Score_SC	Other_Yeasts	Two-Hop_BE	Two-Hop_BP	Two-Hop_BY	Two-Hop_BG	Max_Membership	Sum
YHP1	YOR297C	4.30419			0.380198					4.68439
YHP1	YOR298W	4.30419			0.380198					4.68439
YJL206C	TT(AGU)B	6.6295								6.6295
YJL206C	YBL039C	0.640377			0.447815	3.0127				4.10089
YJL206C	YBR013C	6.6295			0.460098					7.0896
YJL206C	YDR168W	2.98024					1.03948			4.01972
YJL206C	YEL043W	6.97712			0.407598	0.377048				7.76177
YJL206C	YEL044W	6.97712								6.97712
YJL206C	YEL046C	6.97712								6.97712
YJL206C	YGL262W	7.72707								7.72707
YJL206C	YGL263W	3.60949			1.07701					4.6865
YJL206C	YHL008C	10.214								10.214
YJL206C	YNR018W	5.24336								5.24336
YJL206C	YNR019W	5.24336								5.24336
YJL206C	YNR071C	4.19082								4.19082
YJL206C	YNR072W	4.19082								4.19082
YJL206C	YOR388C	12.476								12.476
YJL206C	YOR389W	12.476								12.476
YJL206C	YPL277C	9.78987								9.78987
YOX1	SNR37	4.83627	0.0623883							4.89866
YOX1	SNR53	4.67277								4.67277
YOX1	TG(GCC)G2	2.84583	1.79625							4.64208
YOX1	TK(CUU)C	5.21515								5.21515
YOX1	TL(CAA)M	4.42	1.79625							6.21625
YOX1	TR(UCU)E	4.03677								4.03677
YOX1	TW(CCA)J	3.21603	1.79625							5.01228
YOX1	YAR018C	4.9421								4.9421
YOX1	YBR029C		0.0784949	4.99325						5.07174
YOX1	YBR030W		0.0784949	5.35729						5.43578
YOX1	YBR072W		1.23862	3.81256						5.05118
YOX1	YBR202W	1.08001	0.205727		0.928925	1.46537	0.86534			4.54537
YOX1	YCR019W	5.21515								5.21515
YOX1	YDR041W	2.94398	0.475722				0.904386			4.32409
YOX1	YDR084C	4.29661								4.29661
YOX1	YDR190C	3.36949	1.80762	3.10106			0.851951			9.13012
YOX1	YDR496C	0.891304		3.11672	1.10825					5.11627
YOX1	YEL032W	3.30438			0.511895	1.46537	0.86651			6.14816
YOX1	YEL050C	4.67277								4.67277
YOX1	YER102W			1.9458	1.28916		0.877566			4.11253
YOX1	YGL001C	5.84801	1.80762							7.65563
YOX1	YGL111W		1.79625	9.1683	1.26143					12.226
YOX1	YGL226W			4.67789						4.67789
YOX1	YGR019W		1.53026	4.00478			0.881659			6.4167
YOX1	YGR071C	3.09761	0.539097	7.22085						10.8576
YOX1	YGR074W	3.09761	0.539097	1.31644						4.95315
YOX1	YGR100W		1.79625	2.81349						4.60974
YOX1	YGR175C			3.19543	1.12196		0.777151			5.09454
YOX1	YGR218W	2.84583	1.79625			0.517812				5.15989
YOX1	YJL011C	2.28055	1.79625	1.21725						5.29405
YOX1	YJL076W		0.475722	3.94204						4.41776
YOX1	YJL078C		0.475722	3.65692						4.13264
YOX1	YJR011C	2.32792		1.92999						4.25791
YOX1	YJR100C	0.239727		4.03422						4.27395
YOX1	YKL062W			11.4747						11.4747
YOX1	YKL090W			4.50878						4.50878
YOX1	YKL175W			5.87495						5.87495
YOX1	YLR022C			3.0676			0.953195			4.02079
YOX1	YLR047C	5.15616								5.15616
YOX1	YLR048W	5.15616			0.855634		0.969785			6.98158
YOX1	YLR052W			4.80525						4.80525
YOX1	YLR243W		0.0784949	5.9792			0.915992			6.97369
YOX1	YLR273C	6.03838								6.03838
YOX1	YLR274W	6.03838			0.67542	0.727319	0.866205			8.30732
YOX1	YLR286C			6.12287						6.12287
YOX1	YMR038C		1.53026	6.59012						8.12038
YOX1	YMR039C			4.34694						4.34694
YOX1	YMR040W		1.53026	2.50544						4.0357
YOX1	YMR117C	4.42	1.23862	1.07391						6.73253
YOX1	YMR118C	4.42	1.79625							6.21625
YOX1	YMR119W	4.42	1.79625		0.436787					6.65304
YOX1	YMR193W	3.95481	0.122554							4.07736
YOX1	YMR194W	3.95481	0.122554		0.69058					4.76794
YOX1	YMR218C		2.0612	5.7943						7.8555
YOX1	YMR258C	4.47197	0.0623883							4.53436
YOX1	YMR317W			7.47557						7.47557
YOX1	YNL145W	4.08637								4.08637
YOX1	YNL146C-A	4.08637								4.08637
YOX1	YNL148C	4.08637								4.08637
YOX1	YOL079W		1.53026	5.47712						7.00738
YOX1	YOR001W	5.00471		0.97655	0.6748					6.65606
YOX1	YOR062C			4.02685						4.02685
YOX1	YOR196C		1.53026	3.22007						4.75033
YOX1	YOR295W			4.92778						4.92778
YOX1	YOR375C		0.205727	4.75242						4.95815
YRR1	YGR184C	3.68281				0.351398				4.03421
YRR1	YJR088C	4.20446								4.20446
YRR1	YJR089W	4.20446								4.20446
ZAP1	YAL011W	0.33169		3.79822						4.12991
ZAP1	YBL029W		0.832277	3.40699						4.23927
ZAP1	YBR011C			5.45568	0.426412					5.88209
ZAP1	YBR151W		0.0697762	1.77273	2.28812					4.13063
ZAP1	YCL067C	7.28135					0.820693			8.10204
ZAP1	YDR112W			5.09226						5.09226
ZAP1	YDR190C		1.51415	3.31539			1.11393			5.94347
ZAP1	YDR205W		0.832277	3.74656						4.57884
ZAP1	YDR407C		0.52115	4.03605						4.5572
ZAP1	YDR490C		2.32334	2.93498						5.25832
ZAP1	YDR492W		2.32334	2.99382						5.31716
ZAP1	YDR510W			4.76051						4.76051
ZAP1	YDR511W			4.79893						4.79893
ZAP1	YER007C-A	0.619158		2.7699			1.12705			4.51611
ZAP1	YER122C		0.292861	2.79465			0.989848			4.07736
ZAP1	YER168C			4.66711						4.66711
ZAP1	YER169W			4.75064						4.75064
ZAP1	YFR031C		1.62491	3.39438						5.01929
ZAP1	YGL098W	0.161243		4.06593						4.22717
ZAP1	YGL101W		0.292861	3.96147						4.25433
ZAP1	YGL241W	3.66157		0.569698						4.23127
ZAP1	YGL255W	3.15011	3.6677	10.3875			0.989848			18.1952
ZAP1	YGL256W			4.80333						4.80333

TF	Target	Binding_0-2000	Motif_Score_SC	Other_Yeasts	Two-Hop_BE	Two-Hop_BP	Two-Hop_BY	Two-Hop_BG	Max_Membership	Sum
ZAP1	YGL257C			4.85488						4.85488
ZAP1	YGR089W	0.285233	1.59986	5.29773						7.18282
ZAP1	YGR248W			4.30528						4.30528
ZAP1	YGR249W		0.7161	5.28193						5.99803
ZAP1	YHR146W	3.20115					0.989848			4.191
ZAP1	YIL066C		0.497548	5.71844	0.346492					6.56248
ZAP1	YJL054W	13.5498	3.3494							16.8992
ZAP1	YJL055W	13.5498	3.3494	9.46805						26.3672
ZAP1	YJL056C	13.5498	3.3494	2.79793						19.6971
ZAP1	YJL190C	3.11656			0.56822		1.18601			4.87079
ZAP1	YKL165C		1.28864	6.02702						7.31566
ZAP1	YKL175W	6.86437	3.48218	11.2259						21.5724
ZAP1	YKL176C	6.86437	3.48218	8.05266						18.3992
ZAP1	YLL020C		2.12321	2.03805						4.16126
ZAP1	YLR130C	10.6998	3.70634	8.91473						23.3209
ZAP1	YML014W		1.81602	8.22663						10.0427
ZAP1	YML065W	8.28575								8.28575
ZAP1	YML066C	8.28575		0.545191						8.83094
ZAP1	YML067C	8.28575								8.28575
ZAP1	YMR179W		0.509983	3.73464						4.24462
ZAP1	YMR243C		1.19366	6.53689						7.73055
ZAP1	YMR244W		1.19366	2.88686						4.08052
ZAP1	YMR259C		1.04926	5.2544						6.30366
ZAP1	YNL027W			5.54883						5.54883
ZAP1	YNL248C			4.26622						4.26622
ZAP1	YNL253W	5.44141	3.01815	13.8246			1.03672			23.3209
ZAP1	YNL254C	5.44141	3.01815	16.263						24.7226
ZAP1	YNL255C		3.01815				0.995971			4.01412
ZAP1	YNR039C		1.38791	4.30266						5.69057
ZAP1	YNR040W		1.38791	6.45215						7.84006
ZAP1	YOL053W			6.04269						6.04269
ZAP1	YOL084W		2.84106	2.93263						5.77369
ZAP1	YOL086C		2.84106	3.21928						6.06034
ZAP1	YOL154W		1.2085	7.20864						8.41714
ZAP1	YOR228C		0.00361492	4.9222						4.92581
ZAP1	YPL051W			4.31535						4.31535
ZAP1	YPR068C			4.17777						4.17777
ZAP1	YPR070W			5.07256						5.07256
ZAP1	YPR147C			4.64933						4.64933
