# Supplementary material for: Integrated Assessment and Prediction of Transcription Factor Binding
Source: PLoS Comput Biol. 2006 Jun 16;2(6):e70. doi: 10.1371/journal.pcbi.0020070 (PMC1479087; doi:10.1371/journal.pcbi.0020070)
Supplement: Supplementary file 2 [file pcbi.0020070.st004.pdf]

Cluster	Module	# Overl	Cluster	# Targets	p-value	Avg. MB	Avg. MB	Centrality	SD Centr.
Centroid_AA_Starvation-0	FHL1_RAP1_SFP1_YAP6	5	727	5	-4.6	0.99	0.82	1.21	0.01
Centroid_AA_Starvation-0	FHL1_SFP1_YAP6	10	727	10	-9.2	0.99	0.82	1.21	0.01
Centroid_AA_Starvation-0	FHL1_RAP1_YAP6	7	727	8	-5.6	0.99	0.82	1.21	0.01
Centroid_AA_Starvation-0	FHL1_SFP1_YAP1_YAP6	6	727	6	-5.5	0.99	0.82	1.21	0.01
Centroid_AA_Starvation-0	FHL1_SFP1_YAP1	7	727	7	-6.4	0.99	0.82	1.21	0.01
Centroid_AA_Starvation-0	FHL1_YAP6	21	727	23	-17.1	0.98	0.82	1.20	0.04
Centroid_AA_Starvation-0	FHL1_YAP1_YAP6	11	727	11	-10.1	0.97	0.82	1.19	0.05
Centroid_AA_Starvation-0	FHL1_RAP1	45	727	66	-25.9	0.96	0.82	1.17	0.14
Centroid_AA_Starvation-0	CIN5_FHL1_YAP6	6	727	8	-4.2	0.95	0.82	1.17	0.06
Centroid_AA_Starvation-0	SFP1	45	727	73	-23.2	0.95	0.82	1.16	0.16
Centroid_AA_Starvation-0	FHL1_SFP1	44	727	67	-24.3	0.95	0.82	1.16	0.16
Centroid_AA_Starvation-0	YAP6	27	727	48	-12.7	0.95	0.82	1.16	0.14
Centroid_AA_Starvation-0	YAP1_YAP6	12	727	18	-7.1	0.94	0.82	1.16	0.13
Centroid_AA_Starvation-0	FHL1_RAP1_SFP1	22	727	30	-13.9	0.94	0.82	1.15	0.17
Centroid_AA_Starvation-0	RAP1_SFP1	22	727	32	-13.0	0.94	0.82	1.15	0.17
Centroid_AA_Starvation-0	RAP1	51	727	109	-18.9	0.93	0.82	1.14	0.20
Centroid_AA_Starvation-0	FHL1	153	727	252	-79.4	0.93	0.82	1.14	0.20
Centroid_AA_Starvation-0	ACE2_FHL1_RAP1	6	727	6	-5.5	0.92	0.82	1.12	0.22
Centroid_AA_Starvation-0	ACE2	27	727	93	-5.1	0.91	0.82	1.12	0.21
Centroid_AA_Starvation-0	RME1	10	727	18	-5.0	0.89	0.82	1.08	0.21
Centroid_AA_Starvation-0	YAP1	30	727	102	-5.7	0.88	0.82	1.07	0.29
Centroid_AA_Starvation-0	SWI4	36	727	149	-4.6	0.74	0.82	0.90	0.32
Centroid_AA_Starvation-2	CBF1_MET32	16	474	20	-14.2	0.95	0.68	1.39	0.12
Centroid_AA_Starvation-2	CBF1_MET31_MET32	13	474	14	-13.3	0.94	0.68	1.39	0.13
Centroid_AA_Starvation-2	MET32_MET4	12	474	17	-9.7	0.94	0.68	1.38	0.13
Centroid_AA_Starvation-2	MET31_MET32_MET4	11	474	15	-9.2	0.94	0.68	1.37	0.14
Centroid_AA_Starvation-2	MET31_MET4	11	474	16	-8.7	0.94	0.68	1.37	0.14
Centroid_AA_Starvation-2	CBF1_MET32_TYE7	6	474	7	-5.8	0.93	0.68	1.37	0.14
Centroid_AA_Starvation-2	CBF1_MET32_MET4	8	474	9	-7.9	0.92	0.68	1.35	0.15
Centroid_AA_Starvation-2	CBF1_MET31_MET32_MET4	7	474	7	-7.7	0.91	0.68	1.34	0.17
Centroid_AA_Starvation-2	MET31_MET32_TYE7	5	474	5	-5.5	0.91	0.68	1.34	0.15
Centroid_AA_Starvation-2	CBF1_MET31_MET32_TYE7	4	474	4	-4.4	0.91	0.68	1.34	0.17
Centroid_AA_Starvation-2	MET32	29	474	64	-15.3	0.90	0.68	1.33	0.21
Centroid_AA_Starvation-2	MET32_MET4_TYE7	5	474	5	-5.5	0.90	0.68	1.33	0.14
Centroid_AA_Starvation-2	CBF1_MET32_MET4_TYE7	4	474	4	-4.4	0.90	0.68	1.32	0.16
Centroid_AA_Starvation-2	MET4	14	474	35	-6.8	0.89	0.68	1.31	0.28
Centroid_AA_Starvation-2	MET31_MET32	24	474	47	-14.2	0.89	0.68	1.31	0.23
Centroid_AA_Starvation-2	MET31_MET32_MET4_TYE7	4	474	4	-4.4	0.89	0.68	1.31	0.15
Centroid_AA_Starvation-2	CBF1	33	474	156	-7.0	0.88	0.68	1.29	0.28
Centroid_AA_Starvation-2	MET31	25	474	49	-14.8	0.87	0.68	1.28	0.28
Centroid_AA_Starvation-2	MSN2	28	474	95	-9.4	0.77	0.68	1.13	0.38
Centroid_AA_Starvation-2	SKN7	21	474	95	-5.0	0.67	0.68	0.99	0.46
Centroid_AA_Starvation-2	MSN2_SKN7	8	474	18	-4.5	0.57	0.68	0.84	0.45
Centroid_AA_Starvation-2	RTG3	10	474	16	-7.4	0.56	0.68	0.83	0.37
Centroid_AA_Starvation-3	CIN5	12	208	58	-6.3	0.62	0.55	1.11	0.44
Centroid_AA_Starvation-3	CIN5_FKH2_RLM1	3	208	3	-4.4	0.58	0.55	1.04	0.64
Centroid_AA_Starvation-8	HIR1_HIR2_SWI4	6	224	6	-8.6	0.93	0.52	1.79	0.12
Centroid_AA_Starvation-8	HIR1_HIR2_HIR3_SWI4	4	224	4	-5.7	0.92	0.52	1.76	0.14
Centroid_AA_Starvation-8	HIR1_HIR2_SWI4_YAP1	3	224	3	-4.3	0.92	0.52	1.76	0.17
Centroid_AA_Starvation-8	HIR1_HIR2	7	224	8	-9.2	0.89	0.52	1.70	0.26
Centroid_AA_Starvation-8	HIR2	7	224	9	-8.5	0.89	0.52	1.70	0.26
Centroid_AA_Starvation-8	HIR1	7	224	12	-7.2	0.89	0.52	1.70	0.26
Centroid_AA_Starvation-8	HIR1_HIR2_HIR3	5	224	5	-7.2	0.86	0.52	1.64	0.30
Centroid_AA_Starvation-8	HIR3	5	224	6	-6.4	0.86	0.52	1.64	0.30
Centroid_AA_Starvation-8	HIR1_HIR2_MBP1	3	224	3	-4.3	0.84	0.52	1.61	0.40
Centroid_AA_Starvation-8	FKH2_HIR1_HIR2_HIR3	3	224	3	-4.3	0.83	0.52	1.59	0.38
Centroid_AA_Starvation-8	GCR2	7	224	15	-6.3	0.78	0.52	1.49	0.43
Centroid_AA_Starvation-8	SWI4	25	224	149	-9.8	0.64	0.52	1.22	0.53
Centroid_AA_Starvation-8	SWI4_SWI6	19	224	86	-9.7	0.59	0.52	1.12	0.50
Centroid_AA_Starvation-8	SWI6	23	224	120	-10.3	0.58	0.52	1.11	0.50
Centroid_AA_Starvation-8	STB1_SWI4_SWI6	7	224	21	-5.2	0.58	0.52	1.11	0.52
Centroid_AA_Starvation-8	FKH2	16	224	145	-4.1	0.57	0.52	1.10	0.52
Centroid_AA_Starvation-8	STB1	9	224	47	-4.3	0.57	0.52	1.10	0.46
Centroid_AA_Starvation-8	STB1_SWI6	8	224	22	-6.2	0.56	0.52	1.08	0.49
Centroid_AA_Starvation-8	STE12_SWI4_SWI6	7	224	25	-4.6	0.53	0.52	1.01	0.45
Centroid_AA_Starvation-8	STE12_SWI6	7	224	29	-4.2	0.53	0.52	1.01	0.45
Centroid_AA_Starvation-9	BAS1	10	167	30	-8.4	0.77	0.52	1.49	0.34
Centroid_AA_Starvation-9	GCN4	18	167	76	-11.9	0.49	0.52	0.96	0.43
Centroid_AA_Starvation-9	RTG3	7	167	16	-7.0	0.48	0.52	0.94	0.25
Centroid_Carb_source-1-1	SKN7	16	240	95	-6.1	0.65	0.56	1.16	0.51
Centroid_Carb_source-1-1	MSN2_MSN4	7	240	27	-4.2	0.64	0.56	1.15	0.39
Centroid_Carb_source-1-1	MSN2	15	240	95	-5.4	0.58	0.56	1.04	0.43
Centroid_Carb_source-1-1	HAP4	22	240	62	-15.3	0.51	0.56	0.92	0.48
Centroid_Carb_source-1-1	PHD1	8	240	35	-4.3	0.37	0.56	0.65	0.25
Centroid_Carb_source-1-1	PHD1_SUT1	4	240	7	-4.1	0.36	0.56	0.65	0.29
Centroid_Carb_source-1-1	PHD1_SKN7	6	240	10	-6.2	0.34	0.56	0.60	0.21
Centroid_Carb_source-1-1	PHD1_SKN7_SUT1	3	240	3	-4.2	0.28	0.56	0.50	0.07
Centroid_Carb_source-1-10	MIG1	6	76	39	-5.1	0.47	0.40	1.18	0.53
Centroid_Carb_source-1-3	GCR2	7	460	15	-4.3	0.86	0.71	1.20	0.14
Centroid_Carb_source-1-3	FHL1_RAP1_SFP1_YAP1	4	460	4	-4.5	0.83	0.71	1.17	0.13
Centroid_Carb_source-1-3	FHL1_RAP1_SFP1	17	460	30	-11.4	0.83	0.71	1.16	0.20
Centroid_Carb_source-1-3	RAP1_SFP1	17	460	32	-10.8	0.83	0.71	1.16	0.20
Centroid_Carb_source-1-3	FHL1_RAP1_YAP6	7	460	8	-7.0	0.82	0.71	1.16	0.15
Centroid_Carb_source-1-3	CIN5_YAP6	7	460	14	-4.5	0.82	0.71	1.16	0.21
Centroid_Carb_source-1-3	ACE2_FHL1_RAP1	5	460	6	-4.8	0.82	0.71	1.16	0.29
Centroid_Carb_source-1-3	FHL1_YAP6_YAP7	4	460	4	-4.5	0.82	0.71	1.15	0.24
Centroid_Carb_source-1-3	FHL1_RAP1	35	460	66	-21.8	0.82	0.71	1.15	0.22
Centroid_Carb_source-1-3	CIN5_YAP1_YAP6	5	460	6	-4.8	0.80	0.71	1.13	0.25
Centroid_Carb_source-1-3	RAP1	39	460	109	-16.6	0.80	0.71	1.13	0.23
Centroid_Carb_source-1-3	ACE2	22	460	93	-5.9	0.80	0.71	1.13	0.27
Centroid_Carb_source-1-3	CIN5_FHL1_YAP6	6	460	8	-5.3	0.80	0.71	1.12	0.21
Centroid_Carb_source-1-3	YAP1_YAP6	9	460	18	-5.7	0.79	0.71	1.12	0.20
Centroid_Carb_source-1-3	FHL1_SFP1_YAP1	6	460	7	-5.9	0.79	0.71	1.11	0.14

Cluster	Module	# Overla	Cluster	# Targets	p-value	Avg. MB	Avg. MB	Centrality	SD Centr.
Centroid_Carb_source-1-3	FHL1_RAP1_SFP1_YAP6	5	460	5	-5.6	0.78	0.71	1.10	0.12
Centroid_Carb_source-1-3	YAP1	20	460	102	-4.1	0.78	0.71	1.10	0.30
Centroid_Carb_source-1-3	FHL1_YAP6	15	460	23	-11.4	0.78	0.71	1.10	0.27
Centroid_Carb_source-1-3	FHL1	88	460	252	-36.9	0.78	0.71	1.09	0.27
Centroid_Carb_source-1-3	FHL1_YAP1_YAP6	8	460	11	-6.8	0.77	0.71	1.09	0.18
Centroid_Carb_source-1-3	YAP6	22	460	48	-12.1	0.77	0.71	1.09	0.29
Centroid_Carb_source-1-3	FHL1_SFP1_YAP1_YAP6	5	460	6	-4.8	0.77	0.71	1.08	0.13
Centroid_Carb_source-1-3	CIN5_FHL1_YAP1_YAP6	4	460	4	-4.5	0.76	0.71	1.07	0.25
Centroid_Carb_source-1-3	FHL1_SFP1	31	460	67	-17.1	0.76	0.71	1.07	0.29
Centroid_Carb_source-1-3	SFP1	31	460	73	-15.7	0.76	0.71	1.07	0.29
Centroid_Carb_source-1-3	FHL1_SFP1_YAP6	9	460	10	-9.1	0.73	0.71	1.03	0.29
Centroid_Carb_source-1-4	HAP1_HAP4	8	463	12	-6.4	0.72	0.64	1.12	0.29
Centroid_Carb_source-1-4	HAP4	29	463	62	-16.1	0.64	0.64	0.99	0.37
Centroid_Carb_source-1-4	PHD1	11	463	35	-4.4	0.63	0.64	0.99	0.44
Centroid_Carb_source-1-4	SKN7	23	463	95	-6.3	0.56	0.64	0.87	0.41
Centroid_Carb_source-1-4	PHD1_SKN7	7	463	10	-5.8	0.53	0.64	0.83	0.45
Centroid_Carb_source-1-5	GAL4	6	70	28	-6.2	0.94	0.45	2.10	0.26
Centroid_Carb_source-1-6	GAL4	7	55	28	-8.4	0.66	0.50	1.32	0.70
Centroid_Carb_source-2-0	ACE2_FHL1_RAP1	5	472	6	-4.8	0.74	0.53	1.40	0.45
Centroid_Carb_source-2-0	FHL1_YAP1_YAP6	6	472	11	-4.1	0.72	0.53	1.36	0.41
Centroid_Carb_source-2-0	YAP1_YAP6	8	472	18	-4.5	0.68	0.53	1.28	0.47
Centroid_Carb_source-2-0	GCN4	19	472	76	-5.4	0.65	0.53	1.22	0.46
Centroid_Carb_source-2-0	GCR2	7	472	15	-4.2	0.62	0.53	1.17	0.29
Centroid_Carb_source-2-0	FHL1_RAP1_SFP1	17	472	30	-11.2	0.62	0.53	1.17	0.46
Centroid_Carb_source-2-0	RAP1_SFP1	17	472	32	-10.6	0.62	0.53	1.17	0.46
Centroid_Carb_source-2-0	RAP1	35	472	109	-12.9	0.59	0.53	1.11	0.47
Centroid_Carb_source-2-0	YAP1	25	472	102	-6.7	0.59	0.53	1.10	0.51
Centroid_Carb_source-2-0	FHL1_RAP1	33	472	66	-19.1	0.58	0.53	1.08	0.47
Centroid_Carb_source-2-0	FHL1_YAP6	11	472	23	-6.5	0.57	0.53	1.08	0.50
Centroid_Carb_source-2-0	YAP6	17	472	48	-7.3	0.57	0.53	1.07	0.50
Centroid_Carb_source-2-0	FHL1	95	472	252	-42.3	0.56	0.53	1.05	0.43
Centroid_Carb_source-2-0	FHL1_SFP1	31	472	67	-16.7	0.55	0.53	1.03	0.46
Centroid_Carb_source-2-0	SFP1	32	472	73	-16.4	0.55	0.53	1.03	0.45
Centroid_Carb_source-2-0	ACE2	24	472	93	-7.0	0.53	0.53	1.00	0.47
Centroid_Carb_source-2-0	FHL1_SFP1_YAP6	6	472	10	-4.4	0.46	0.53	0.87	0.56
Centroid_Carb_source-2-12	FHL1	23	132	252	-8.4	0.43	0.42	1.02	0.41
Centroid_Carb_source-2-12	FHL1_SFP1	8	132	67	-4.0	0.41	0.42	0.97	0.28
Centroid_Carb_source-2-3	RAP1	23	315	109	-8.2	0.61	0.49	1.23	0.55
Centroid_Carb_source-2-3	FHL1_RAP1	20	315	66	-10.2	0.59	0.49	1.19	0.58
Centroid_Carb_source-2-3	FHL1	57	315	252	-21.7	0.58	0.49	1.17	0.53
Centroid_Carb_source-2-3	FHL1_SFP1	16	315	67	-6.7	0.55	0.49	1.12	0.48
Centroid_Carb_source-2-3	SFP1	17	315	73	-6.9	0.53	0.49	1.08	0.50
Centroid_Carb_source-2-4	MIG1	11	382	39	-4.7	0.52	0.51	1.02	0.59
Centroid_Carb_source-2-5	HAP1_HAP4	9	426	12	-8.1	0.84	0.55	1.52	0.41
Centroid_Carb_source-2-5	HAP4	34	426	62	-22.9	0.79	0.55	1.43	0.46
Centroid_Carb_source-2-5	RTG3	8	426	16	-5.3	0.69	0.55	1.25	0.38
Centroid_Carb_source-2-5	PHD1_SKN7	6	426	10	-4.7	0.65	0.55	1.18	0.28
Centroid_Carb_source-2-5	MSN2	19	426	95	-4.5	0.63	0.55	1.14	0.41
Centroid_Carb_source-2-5	SKN7	24	426	95	-7.6	0.62	0.55	1.13	0.44
Centroid_Carb_source-2-5	MSN2_MSN4	10	426	27	-5.1	0.62	0.55	1.12	0.38
Centroid_Carb_source-2-5	MSN4	13	426	55	-4.1	0.60	0.55	1.08	0.36
Centroid_Carb_source-2-5	MIG1	15	426	39	-7.6	0.56	0.55	1.02	0.43
Centroid_Carb_source-2-6	GAL4	10	44	28	-14.8	0.73	0.48	1.51	0.56
Centroid_Carb_source-2-9	RCS1	10	170	69	-4.7	0.64	0.46	1.40	0.59
Centroid_Cell-cycle-0	FKH2_MCM1_NDD1_SWI4	3	79	7	-4.1	0.93	0.71	1.31	0.13
Centroid_Cell-cycle-0	FKH2_MCM1_NDD1_STE12	4	79	6	-6.4	0.93	0.71	1.31	0.16
Centroid_Cell-cycle-0	FKH2_NDD1_STE12	4	79	9	-5.5	0.93	0.71	1.31	0.16
Centroid_Cell-cycle-0	MCM1_NDD1_STE12	4	79	13	-4.7	0.93	0.71	1.31	0.16
Centroid_Cell-cycle-0	FKH2_MCM1_NDD1	14	79	26	-19.9	0.89	0.71	1.26	0.13
Centroid_Cell-cycle-0	MCM1_NDD1	14	79	51	-14.9	0.89	0.71	1.26	0.13
Centroid_Cell-cycle-0	MCM1	14	79	103	-10.4	0.89	0.71	1.26	0.13
Centroid_Cell-cycle-0	FKH1_FKH2_NDD1	5	79	10	-7.1	0.88	0.71	1.24	0.14
Centroid_Cell-cycle-0	FKH1_FKH2	5	79	31	-4.3	0.88	0.71	1.24	0.14
Centroid_Cell-cycle-0	FKH2_NDD1	20	79	48	-25.6	0.87	0.71	1.23	0.21
Centroid_Cell-cycle-0	NDD1	25	79	143	-21.6	0.86	0.71	1.21	0.22
Centroid_Cell-cycle-0	FKH1_FKH2_MCM1_NDD1	4	79	7	-6.0	0.85	0.71	1.21	0.13
Centroid_Cell-cycle-0	FKH2	26	79	145	-22.8	0.84	0.71	1.19	0.26
Centroid_Cell-cycle-0	FKH2_NDD1_SWI4	4	79	12	-4.9	0.83	0.71	1.18	0.29
Centroid_Cell-cycle-0	RCS1	7	79	69	-4.5	0.57	0.71	0.80	0.31
Centroid_Cell-cycle-1	FKH2_NDD1	8	61	48	-7.7	0.87	0.65	1.34	0.16
Centroid_Cell-cycle-1	NDD1	13	61	143	-9.0	0.82	0.65	1.27	0.20
Centroid_Cell-cycle-1	MCM1_NDD1	8	61	51	-7.5	0.81	0.65	1.25	0.22
Centroid_Cell-cycle-1	MCM1	13	61	103	-10.8	0.74	0.65	1.15	0.29
Centroid_Cell-cycle-1	FKH2	13	61	145	-8.9	0.72	0.65	1.11	0.41
Centroid_Cell-cycle-11	CBF1_MET31_MET32_MET4	2	11	7	-4.2	0.77	0.76	1.02	0.38
Centroid_Cell-cycle-11	MET31_MET32_MET4	4	11	15	-8.1	0.73	0.76	0.96	0.26
Centroid_Cell-cycle-11	MET31_MET4	4	11	16	-8.0	0.73	0.76	0.96	0.26
Centroid_Cell-cycle-11	MET32_MET4	4	11	17	-7.8	0.73	0.76	0.96	0.26
Centroid_Cell-cycle-11	MET4	4	11	35	-6.5	0.73	0.76	0.96	0.26
Centroid_Cell-cycle-11	MET31_MET32	4	11	47	-6.0	0.73	0.76	0.96	0.26
Centroid_Cell-cycle-11	MET31	4	11	49	-5.9	0.73	0.76	0.96	0.26
Centroid_Cell-cycle-11	MET32	4	11	64	-5.4	0.73	0.76	0.96	0.26
Centroid_Cell-cycle-16	ACE2_FKH1_FKH2	2	11	5	-4.5	0.95	0.61	1.57	0.09
Centroid_Cell-cycle-16	STE12_SWI4	3	11	31	-4.7	0.26	0.61	0.43	0.09
Centroid_Cell-cycle-19	DIG1_STE12	7	13	68	-10.5	0.63	0.47	1.34	0.77
Centroid_Cell-cycle-19	DIG1	7	13	86	-9.8	0.63	0.47	1.34	0.77
Centroid_Cell-cycle-19	STE12	11	13	157	-15.7	0.51	0.47	1.08	0.70
Centroid_Cell-cycle-19	MCM1_STE12	4	13	20	-7.2	0.33	0.47	0.72	0.29
Centroid_Cell-cycle-19	MCM1	4	13	103	-4.3	0.33	0.47	0.72	0.29
Centroid_Cell-cycle-2	ACE2	7	38	93	-5.8	0.77	0.64	1.19	0.32
Centroid_Cell-cycle-2	ACE2_SWI5	6	38	13	-10.2	0.74	0.64	1.14	0.31
Centroid_Cell-cycle-2	SWI5	16	38	44	-25.2	0.67	0.64	1.05	0.35
Centroid_Cell-cycle-2	ACE2_FKH1_FKH2_STE12_SWI5	2	38	2	-4.4	0.65	0.64	1.02	0.04

Cluster	Module	# Overla	Cluster	# Targets	p-value	Avg. MB	Avg. MB	Centrality	SD Centr.
Centroid_Cell-cycle-2	MCM1	7	38	103	-5.5	0.61	0.64	0.95	0.42
Centroid_Cell-cycle-2	ACE2_FKH2_STE12_SWI5	3	38	3	-6.6	0.59	0.64	0.92	0.16
Centroid_Cell-cycle-2	ACE2_STE12_SWI5	3	38	4	-6.0	0.59	0.64	0.92	0.16
Centroid_Cell-cycle-2	ACE2_FKH2_SWI5	3	38	5	-5.6	0.59	0.64	0.92	0.16
Centroid_Cell-cycle-2	STE12	10	38	157	-7.6	0.50	0.64	0.78	0.28
Centroid_Cell-cycle-5	MBP1	18	84	174	-10.8	0.70	0.68	1.03	0.35
Centroid_Cell-cycle-5	SWI6	16	84	120	-11.3	0.64	0.68	0.93	0.37
Centroid_Cell-cycle-5	SWI4_SWI6	13	84	86	-9.9	0.59	0.68	0.86	0.36
Centroid_Cell-cycle-5	STE12	13	84	157	-6.7	0.58	0.68	0.85	0.38
Centroid_Cell-cycle-5	STE12_SWI6	7	84	29	-7.0	0.57	0.68	0.83	0.35
Centroid_Cell-cycle-5	SWI4	20	84	149	-14.2	0.55	0.68	0.81	0.40
Centroid_Cell-cycle-5	STE12_SWI4	8	84	31	-8.2	0.55	0.68	0.80	0.37
Centroid_Cell-cycle-5	STB1_SWI4	10	84	44	-9.6	0.54	0.68	0.79	0.43
Centroid_Cell-cycle-5	STB1	10	84	47	-9.3	0.54	0.68	0.79	0.43
Centroid_Cell-cycle-5	STE12_SWI4_SWI6	6	84	25	-6.1	0.51	0.68	0.75	0.31
Centroid_Cell-cycle-5	DIG1_STE12_SWI4_SWI6	4	84	9	-5.4	0.51	0.68	0.75	0.40
Centroid_Cell-cycle-5	DIG1_SWI4_SWI6	4	84	10	-5.2	0.51	0.68	0.75	0.40
Centroid_Cell-cycle-5	DIG1_STE12_SWI6	4	84	11	-5.0	0.51	0.68	0.75	0.40
Centroid_Cell-cycle-5	DIG1_STE12_SWI4	4	84	11	-5.0	0.51	0.68	0.75	0.40
Centroid_Cell-cycle-6	SWI5_YAP5	5	97	5	-9.0	0.99	0.70	1.41	0.02
Centroid_Cell-cycle-6	MSN4_PDR1_YAP5	3	97	3	-5.4	0.99	0.70	1.41	0.02
Centroid_Cell-cycle-6	YAP5	9	97	12	-14.0	0.95	0.70	1.35	0.19
Centroid_Cell-cycle-6	SWI5	8	97	44	-6.4	0.89	0.70	1.27	0.36
Centroid_Cell-cycle-6	MBP1_SWI4	8	97	49	-6.0	0.87	0.70	1.24	0.20
Centroid_Cell-cycle-6	GAL4_TEC1_YAP5	3	97	3	-5.4	0.86	0.70	1.22	0.34
Centroid_Cell-cycle-6	SWI4	13	97	149	-6.2	0.76	0.70	1.08	0.35
Centroid_Cell-cycle-6	MBP1	22	97	174	-13.7	0.65	0.70	0.92	0.43
Centroid_Cell-cycle-6	MBP1_SWI6	10	97	53	-8.1	0.57	0.70	0.81	0.44
Centroid_Cell-cycle-6	SWI6	12	97	120	-6.4	0.56	0.70	0.79	0.41
Centroid_Cell-cycle-7	HIR1_HIR2_MBP1	3	63	3	-6.0	1.00	0.68	1.48	0.00
Centroid_Cell-cycle-7	HIR1_HIR2_SWI4_YAP1	3	63	3	-6.0	1.00	0.68	1.48	0.00
Centroid_Cell-cycle-7	HIR1_HIR2_SWI4	6	63	6	-12.0	1.00	0.68	1.48	0.00
Centroid_Cell-cycle-7	HIR1_HIR2	8	63	8	-16.0	1.00	0.68	1.48	0.00
Centroid_Cell-cycle-7	HIR2	8	63	9	-15.1	1.00	0.68	1.48	0.00
Centroid_Cell-cycle-7	HIR1	8	63	12	-13.4	1.00	0.68	1.48	0.00
Centroid_Cell-cycle-7	HIR1_HIR2_HIR3	5	63	5	-10.0	1.00	0.68	1.48	0.00
Centroid_Cell-cycle-7	HIR3	5	63	6	-9.2	1.00	0.68	1.48	0.00
Centroid_Cell-cycle-7	FKH2_HIR1_HIR2_HIR3	3	63	3	-6.0	1.00	0.68	1.48	0.00
Centroid_Cell-cycle-7	HIR1_HIR2_HIR3_SWI4	4	63	4	-8.0	1.00	0.68	1.48	0.00
Centroid_Cell-cycle-7	NDD1_SWI4_SWI6	4	63	24	-4.0	0.81	0.68	1.19	0.31
Centroid_Cell-cycle-7	SWI4	21	63	149	-18.3	0.80	0.68	1.18	0.37
Centroid_Cell-cycle-7	SWI6	17	63	120	-14.7	0.78	0.68	1.15	0.37
Centroid_Cell-cycle-7	SWI4_SWI6	15	63	86	-14.4	0.77	0.68	1.14	0.39
Centroid_Cell-cycle-7	MBP1_SWI4	6	63	49	-5.0	0.75	0.68	1.11	0.46
Centroid_Cell-cycle-7	FKH2	14	63	145	-9.8	0.60	0.68	0.89	0.47
Centroid_Cell-cycle-7	FKH2_SWI4_SWI6	5	63	17	-6.2	0.59	0.68	0.87	0.45
Centroid_Cell-cycle-7	FKH2_SWI6	5	63	21	-5.7	0.59	0.68	0.87	0.45
Centroid_Cell-cycle-8	MBP1_STB1	7	134	14	-8.2	0.90	0.78	1.15	0.28
Centroid_Cell-cycle-8	MBP1_STB1_SWI4	6	134	13	-6.8	0.89	0.78	1.13	0.30
Centroid_Cell-cycle-8	DIG1_MBP1_STE12	4	134	8	-4.8	0.87	0.78	1.11	0.23
Centroid_Cell-cycle-8	STB1_SWI6	7	134	22	-6.5	0.87	0.78	1.11	0.27
Centroid_Cell-cycle-8	MBP1_STB1_SWI6	5	134	9	-6.2	0.86	0.78	1.10	0.33
Centroid_Cell-cycle-8	STB1_SWI4_SWI6	6	134	21	-5.3	0.85	0.78	1.09	0.29
Centroid_Cell-cycle-8	STB1	15	134	47	-13.5	0.84	0.78	1.07	0.25
Centroid_Cell-cycle-8	MBP1_STB1_SWI4_SWI6	4	134	8	-4.8	0.83	0.78	1.06	0.36
Centroid_Cell-cycle-8	STB1_SWI4	14	134	44	-12.6	0.83	0.78	1.06	0.26
Centroid_Cell-cycle-8	MBP1_SWI4	19	134	49	-18.9	0.82	0.78	1.05	0.33
Centroid_Cell-cycle-8	MBP1_NDD1_SWI4_SWI6	6	134	14	-6.5	0.80	0.78	1.02	0.36
Centroid_Cell-cycle-8	NDD1_SWI4_SWI6	6	134	24	-5.0	0.80	0.78	1.02	0.36
Centroid_Cell-cycle-8	NDD1_SWI4	6	134	28	-4.6	0.80	0.78	1.02	0.36
Centroid_Cell-cycle-8	MBP1	58	134	174	-55.8	0.79	0.78	1.01	0.32
Centroid_Cell-cycle-8	SWI4	32	134	149	-22.9	0.79	0.78	1.01	0.31
Centroid_Cell-cycle-8	SWI4_SWI6	18	134	86	-12.6	0.79	0.78	1.01	0.33
Centroid_Cell-cycle-8	MBP1_STE12_SWI4	5	134	8	-6.6	0.79	0.78	1.01	0.33
Centroid_Cell-cycle-8	MBP1_SWI6	24	134	53	-25.9	0.78	0.78	1.00	0.30
Centroid_Cell-cycle-8	MBP1_SWI4_SWI6	15	134	34	-16.0	0.78	0.78	1.00	0.36
Centroid_Cell-cycle-8	MBP1_STE12_SWI6	5	134	9	-6.2	0.77	0.78	0.98	0.31
Centroid_Cell-cycle-8	SWI6	28	134	120	-21.0	0.77	0.78	0.98	0.31
Centroid_Cell-cycle-8	MBP1_NDD1_SWI6	7	134	15	-7.9	0.76	0.78	0.98	0.35
Centroid_Cell-cycle-8	NDD1_SWI6	7	134	27	-5.8	0.76	0.78	0.98	0.35
Centroid_Cell-cycle-8	MBP1_STE12_SWI4_SWI6	4	134	6	-5.5	0.74	0.78	0.94	0.34
Centroid_DES459_MMS-0	HAL9_YAP1_YAP7	2	53	2	-4.1	1.00	0.87	1.15	0.00
Centroid_DES459_MMS-0	CAD1_RCS1_YAP1	3	53	5	-5.2	0.99	0.87	1.14	0.01
Centroid_DES459_MMS-0	RCS1	6	53	69	-4.6	0.99	0.87	1.14	0.01
Centroid_DES459_MMS-0	MSN2_YAP1_YAP7	3	53	7	-4.7	0.99	0.87	1.14	0.01
Centroid_DES459_MMS-0	YAP1	14	53	102	-13.0	0.99	0.87	1.14	0.02
Centroid_DES459_MMS-0	YAP1_YAP7	11	53	34	-14.7	0.99	0.87	1.13	0.02
Centroid_DES459_MMS-0	CAD1_YAP1	9	53	28	-12.0	0.99	0.87	1.13	0.03
Centroid_DES459_MMS-0	CIN5_YAP1	5	53	15	-6.9	0.99	0.87	1.13	0.04
Centroid_DES459_MMS-0	CAD1_YAP1_YAP7	6	53	20	-7.9	0.98	0.87	1.13	0.03
Centroid_DES459_MMS-0	CAD1_YAP7	6	53	29	-6.8	0.98	0.87	1.13	0.03
Centroid_DES459_MMS-0	MSN2_SKN7	4	53	18	-4.8	0.98	0.87	1.13	0.01
Centroid_DES459_MMS-0	CIN5_YAP1_YAP7	4	53	7	-6.7	0.98	0.87	1.13	0.04
Centroid_DES459_MMS-0	CAD1	10	53	43	-11.7	0.98	0.87	1.12	0.03
Centroid_DES459_MMS-0	RPN4_YAP1_YAP7	4	53	5	-7.6	0.98	0.87	1.12	0.04
Centroid_DES459_MMS-0	CAD1_RPN4_YAP1_YAP7	3	53	3	-6.2	0.97	0.87	1.12	0.04
Centroid_DES459_MMS-0	YAP7	12	53	67	-12.6	0.95	0.87	1.09	0.15
Centroid_DES459_MMS-0	CIN5	8	53	58	-7.5	0.94	0.87	1.08	0.14
Centroid_DES459_MMS-0	MSN2_MSN4	4	53	27	-4.1	0.94	0.87	1.07	0.12
Centroid_DES459_MMS-0	ARG81_GCN4_GLN3_RTG3	2	53	2	-4.1	0.81	0.87	0.93	0.05
Centroid_DES459_MMS-3	HAP4	10	52	62	-10.1	0.99	0.84	1.18	0.01
Centroid_DES459_MMS-3	HAP1_HAP4	8	52	12	-14.1	0.99	0.84	1.18	0.01
Centroid_DES459_MMS-3	HAP1	24	52	112	-28.2	0.99	0.84	1.18	0.04

Cluster	Module	# Overla	Cluster	# Targets	p-value	Avg. MB	Avg. MB	Centrality	SD Centr.
Centroid_DES459_MMS-4	ACE2_FKH2_STE12_SWI5	2	20	3	-4.5	0.99	0.70	1.42	0.01
Centroid_DES459_MMS-4	ACE2_STE12_SWI5	2	20	4	-4.2	0.99	0.70	1.42	0.01
Centroid_DES459_MMS-4	ACE2_FKH2_SWI5	3	20	5	-6.5	0.97	0.70	1.40	0.04
Centroid_DES459_MMS-4	ACE2_SWI5	3	20	13	-5.1	0.97	0.70	1.40	0.04
Centroid_DES459_MMS-4	ACE2_FKH1_FKH2	3	20	5	-6.5	0.97	0.70	1.40	0.02
Centroid_DES459_MMS-4	SWI5	6	20	44	-8.4	0.94	0.70	1.35	0.12
Centroid_DES459_MMS-4	ACE2	7	20	93	-8.0	0.88	0.70	1.27	0.22
Centroid_DES459_MMS-4	FKH2	7	20	145	-6.6	0.78	0.70	1.12	0.48
Centroid_DES460_MMS-0	CAD1_YAP1_YAP7	6	171	20	-4.9	1.00	0.91	1.10	0.00
Centroid_DES460_MMS-0	YAP1_YAP7	10	171	34	-7.7	1.00	0.91	1.10	0.00
Centroid_DES460_MMS-0	YAP1	14	171	102	-6.0	1.00	0.91	1.10	0.01
Centroid_DES460_MMS-0	YAP7	12	171	67	-6.5	1.00	0.91	1.10	0.01
Centroid_DES460_MMS-0	CIN5_YAP1	6	171	15	-5.7	1.00	0.91	1.10	0.00
Centroid_DES460_MMS-0	CIN5_YAP1_YAP7	4	171	7	-4.7	1.00	0.91	1.10	0.01
Centroid_DES460_MMS-0	AFT2	7	171	39	-4.0	1.00	0.91	1.10	0.00
Centroid_DES460_MMS-0	CAD1_YAP1	9	171	28	-7.4	0.99	0.91	1.10	0.01
Centroid_DES460_MMS-0	MSN2_MSN4	6	171	27	-4.1	0.99	0.91	1.10	0.01
Centroid_DES460_MMS-0	CAD1_CIN5_YAP1	3	171	4	-4.1	0.99	0.91	1.10	0.01
Centroid_DES460_MMS-0	MSN2	12	171	95	-4.9	0.99	0.91	1.09	0.01
Centroid_DES460_MMS-0	RCS1	10	171	69	-4.7	0.99	0.91	1.09	0.01
Centroid_DES460_MMS-0	CAD1	10	171	43	-6.6	0.98	0.91	1.08	0.04
Centroid_DES460_MMS-0	MSN4	10	171	55	-5.6	0.98	0.91	1.08	0.05
Centroid_DES460_MMS-0	MBP1	16	171	174	-4.5	0.98	0.91	1.08	0.05
Centroid_DES460_MMS-0	MBP1_SWI6	10	171	53	-5.7	0.98	0.91	1.08	0.06
Centroid_DES460_MMS-0	CIN5	10	171	58	-5.4	0.96	0.91	1.06	0.13
Centroid_DES460_MMS-2	HIR3	4	82	6	-6.3	0.99	0.93	1.06	0.01
Centroid_DES460_MMS-2	HIR1_HIR2_HIR3_SWI4	3	82	4	-5.0	0.98	0.93	1.05	0.01
Centroid_DES460_MMS-2	HIR1_HIR2_HIR3	3	82	5	-4.6	0.98	0.93	1.05	0.01
Centroid_DES460_MMS-2	HIR1_HIR2_SWI4_YAP1	3	82	3	-5.6	0.98	0.93	1.05	0.01
Centroid_DES460_MMS-2	SWI4	12	82	149	-6.2	0.98	0.93	1.05	0.02
Centroid_DES460_MMS-2	HIR1_HIR2_SWI4	5	82	6	-8.6	0.98	0.93	1.05	0.01
Centroid_DES460_MMS-2	HIR1_HIR2	5	82	8	-7.6	0.98	0.93	1.05	0.01
Centroid_DES460_MMS-2	HIR2	5	82	9	-7.3	0.98	0.93	1.05	0.01
Centroid_DES460_MMS-2	HIR1	5	82	12	-6.5	0.98	0.93	1.05	0.01
Centroid_DES460_MMS-2	FKH2_NDD1_SWI4	5	82	12	-6.5	0.98	0.93	1.05	0.03
Centroid_DES460_MMS-2	NDD1_SWI4	5	82	28	-4.5	0.98	0.93	1.05	0.03
Centroid_DES460_MMS-2	FKH2_MCM1_NDD1_SWI4	4	82	7	-6.0	0.97	0.93	1.04	0.04
Centroid_DES460_MMS-2	MCM1_NDD1_SWI4	4	82	13	-4.7	0.97	0.93	1.04	0.04
Centroid_DES460_MMS-2	NDD1	17	82	143	-11.4	0.97	0.93	1.04	0.04
Centroid_DES460_MMS-2	HAP1_HAP4	7	82	12	-10.3	0.97	0.93	1.04	0.03
Centroid_DES460_MMS-2	FKH2_NDD1	15	82	48	-16.7	0.97	0.93	1.04	0.05
Centroid_DES460_MMS-2	HAP4	10	82	62	-8.1	0.97	0.93	1.04	0.04
Centroid_DES460_MMS-2	FKH1_FKH2_MCM1_NDD1_SWI4_SWI6	3	82	3	-5.6	0.96	0.93	1.03	0.04
Centroid_DES460_MMS-2	FKH1_FKH2_MCM1_NDD1_SWI6	3	82	4	-5.0	0.96	0.93	1.03	0.04
Centroid_DES460_MMS-2	FKH1_FKH2_SWI4_SWI6	3	82	5	-4.6	0.96	0.93	1.03	0.04
Centroid_DES460_MMS-2	FKH2_MCM1_NDD1_SWI4_SWI6	3	82	6	-4.3	0.96	0.93	1.03	0.04
Centroid_DES460_MMS-2	FKH1_SWI4_SWI6	3	82	6	-4.3	0.96	0.93	1.03	0.04
Centroid_DES460_MMS-2	FKH1_FKH2_MCM1_NDD1	3	82	7	-4.1	0.96	0.93	1.03	0.04
Centroid_DES460_MMS-2	FKH2_MCM1_NDD1_SWI6	3	82	7	-4.1	0.96	0.93	1.03	0.04
Centroid_DES460_MMS-2	FKH1_FKH2_SWI6	3	82	7	-4.1	0.96	0.93	1.03	0.04
Centroid_DES460_MMS-2	HAP1	29	82	112	-30.1	0.96	0.93	1.03	0.07
Centroid_DES460_MMS-2	MCM1_NDD1	9	82	51	-7.7	0.96	0.93	1.03	0.05
Centroid_DES460_MMS-2	MCM1	9	82	103	-5.0	0.96	0.93	1.03	0.05
Centroid_DES460_MMS-2	FKH2_MCM1_NDD1	8	82	26	-9.0	0.96	0.93	1.03	0.05
Centroid_DES460_MMS-2	FKH2	20	82	145	-14.7	0.94	0.93	1.01	0.12
Centroid_DES460_MMS-2	HAP1_RDS1	6	82	8	-9.8	0.90	0.93	0.97	0.13
Centroid_DES460_MMS-2	RDS1	6	82	29	-5.7	0.90	0.93	0.97	0.13
Centroid_DES460_MMS-2	YAP1	9	82	102	-5.1	0.89	0.93	0.95	0.22
Centroid_DES460_MMS-3	ACE2_SWI5	4	32	13	-6.3	0.98	0.83	1.19	0.03
Centroid_DES460_MMS-3	ACE2_FKH2_SWI5	3	32	5	-5.9	0.98	0.83	1.18	0.03
Centroid_DES460_MMS-3	ACE2_FKH1_FKH2	3	32	5	-5.9	0.98	0.83	1.18	0.03
Centroid_DES460_MMS-3	ACE2_FKH2_STE12_SWI5	2	32	3	-4.1	0.97	0.83	1.17	0.04
Centroid_DES460_MMS-3	ACE2	9	32	93	-9.1	0.91	0.83	1.10	0.26
Centroid_DES460_MMS-3	FKH2	8	32	145	-6.2	0.90	0.83	1.08	0.22
Centroid_DES460_MMS-3	SWI5	9	32	44	-12.2	0.87	0.83	1.05	0.25
Centroid_DES460_MMS-5	HSF1	5	33	74	-4.3	0.92	0.67	1.38	0.20
Centroid_DES460_MMS-5	GCN4	12	33	76	-14.7	0.81	0.67	1.22	0.44
Centroid_DES460_MMS-5	MET32_MET4	3	33	17	-4.0	0.66	0.67	0.99	0.58
Centroid_DES460_MMS-5	MET32	6	33	64	-6.0	0.62	0.67	0.93	0.43
Centroid_DES460_MMS-5	CBF1_MET32	5	33	20	-7.3	0.58	0.67	0.87	0.45
Centroid_Diamide-1	STE12_SWI4_SWI6	6	39	25	-8.1	0.81	0.62	1.31	0.46
Centroid_Diamide-1	STE12_SWI6	6	39	29	-7.7	0.81	0.62	1.31	0.46
Centroid_Diamide-1	STE12_SWI4	6	39	31	-7.5	0.81	0.62	1.31	0.46
Centroid_Diamide-1	DIG1_STE12_SWI4_SWI6	5	39	9	-9.0	0.79	0.62	1.27	0.50
Centroid_Diamide-1	DIG1_STE12_SWI6	5	39	11	-8.4	0.79	0.62	1.27	0.50
Centroid_Diamide-1	DIG1_STE12_SWI4	5	39	11	-8.4	0.79	0.62	1.27	0.50
Centroid_Diamide-1	SWI4	8	39	149	-5.4	0.78	0.62	1.25	0.48
Centroid_Diamide-1	SWI4_SWI6	7	39	86	-6.0	0.75	0.62	1.21	0.50
Centroid_Diamide-1	SWI6	7	39	120	-5.0	0.75	0.62	1.21	0.50
Centroid_Diamide-1	STE12	9	39	157	-6.3	0.73	0.62	1.17	0.49
Centroid_Diamide-1	DIG1_SWI4_SWI6	6	39	10	-11.0	0.72	0.62	1.16	0.53
Centroid_Diamide-1	DIG1_STE12	7	39	68	-6.7	0.68	0.62	1.10	0.53
Centroid_Diamide-1	DIG1	9	39	86	-8.6	0.60	0.62	0.96	0.53
Centroid_Diamide-11	HIR1_HIR2_MBP1	2	13	3	-4.9	0.98	0.56	1.76	0.04
Centroid_Diamide-11	HIR1_HIR2	4	13	8	-9.0	0.97	0.56	1.73	0.07
Centroid_Diamide-11	HIR2	4	13	9	-8.8	0.97	0.56	1.73	0.07
Centroid_Diamide-11	HIR1_HIR2_SWI4	2	13	6	-4.2	0.96	0.56	1.71	0.11
Centroid_Diamide-11	HIR1_HIR2_HIR3	2	13	5	-4.4	0.94	0.56	1.68	0.07
Centroid_Diamide-11	HIR3	2	13	6	-4.2	0.94	0.56	1.68	0.07
Centroid_Diamide-11	HIR1	5	13	12	-10.8	0.83	0.56	1.49	0.54
Centroid_Diamide-12	STE12_SWI4_SWI6	3	15	25	-4.6	0.55	0.48	1.15	0.81
Centroid_Diamide-12	STE12_SWI6	3	15	29	-4.4	0.55	0.48	1.15	0.81
Centroid_Diamide-12	STE12_SWI4	3	15	31	-4.3	0.55	0.48	1.15	0.81

Cluster	Module	# Overla	Cluster	# Targets	p-value	Avg. MB	Avg. MB	Centrality	SD Centr.
Centroid_Diamide-2	FHL1_RAP1_SFP1_YAP1	4	184	4	-6.1	1.00	0.85	1.18	0.00
Centroid_Diamide-2	FHL1_RAP1_SFP1_YAP1_YAP6	3	184	3	-4.5	1.00	0.85	1.18	0.01
Centroid_Diamide-2	FHL1_RAP1_SFP1_YAP6	5	184	5	-7.6	0.99	0.85	1.18	0.00
Centroid_Diamide-2	FHL1_MBP1_YAP1_YAP6	3	184	3	-4.5	0.99	0.85	1.18	0.00
Centroid_Diamide-2	FHL1_RAP1_YAP6	6	184	8	-7.7	0.99	0.85	1.18	0.00
Centroid_Diamide-2	FHL1_SFP1_YAP6	10	184	10	-15.2	0.99	0.85	1.17	0.02
Centroid_Diamide-2	FHL1_YAP6	18	184	23	-23.1	0.99	0.85	1.17	0.01
Centroid_Diamide-2	FHL1_SFP1_YAP1	7	184	7	-10.6	0.99	0.85	1.17	0.02
Centroid_Diamide-2	FHL1_NDD1_YAP1_YAP6	3	184	3	-4.5	0.99	0.85	1.17	0.02
Centroid_Diamide-2	FHL1_YAP1_YAP6	10	184	11	-14.2	0.99	0.85	1.17	0.02
Centroid_Diamide-2	FHL1_SFP1_YAP1_YAP6	6	184	6	-9.1	0.99	0.85	1.17	0.02
Centroid_Diamide-2	CIN5_FHL1_YAP6	5	184	8	-5.9	0.99	0.85	1.17	0.02
Centroid_Diamide-2	ACE2_FHL1_RAP1	6	184	6	-9.1	0.98	0.85	1.16	0.03
Centroid_Diamide-2	FHL1_YAP1_YAP6_YAP7	3	184	3	-4.5	0.98	0.85	1.15	0.03
Centroid_Diamide-2	FHL1_RAP1	37	184	66	-39.3	0.97	0.85	1.15	0.11
Centroid_Diamide-2	FHL1	113	184	252	-116.9	0.95	0.85	1.13	0.12
Centroid_Diamide-2	FHL1_SFP1	36	184	67	-37.3	0.95	0.85	1.13	0.13
Centroid_Diamide-2	SFP1	36	184	73	-35.5	0.95	0.85	1.13	0.13
Centroid_Diamide-2	FHL1_RAP1_SFP1	18	184	30	-19.8	0.95	0.85	1.12	0.15
Centroid_Diamide-2	RAP1_SFP1	18	184	32	-19.1	0.95	0.85	1.12	0.15
Centroid_Diamide-2	RAP1	39	184	109	-31.8	0.95	0.85	1.12	0.17
Centroid_Diamide-2	YAP1_YAP6	11	184	18	-12.4	0.93	0.85	1.10	0.24
Centroid_Diamide-2	YAP1	19	184	102	-9.8	0.91	0.85	1.07	0.23
Centroid_Diamide-2	ACE2	14	184	93	-6.2	0.87	0.85	1.03	0.32
Centroid_Diamide-2	YAP6	23	184	48	-22.2	0.86	0.85	1.02	0.30
Centroid_Diamide-2	ROX1_YAP6	4	184	7	-4.6	0.82	0.85	0.98	0.40
Centroid_Diamide-2	CIN5_YAP1_YAP6	4	184	6	-4.9	0.82	0.85	0.97	0.39
Centroid_Diamide-2	ROX1_YAP1_YAP6	3	184	3	-4.5	0.77	0.85	0.91	0.46
Centroid_Diamide-2	CIN5_YAP1	5	184	15	-4.2	0.74	0.85	0.88	0.40
Centroid_Diamide-2	CIN5	11	184	58	-6.0	0.73	0.85	0.86	0.39
Centroid_Diamide-2	CIN5_YAP6	8	184	14	-8.8	0.72	0.85	0.86	0.43
Centroid_Diamide-2	CIN5_SWI4_SWI6_YAP6	4	184	4	-6.1	0.63	0.85	0.74	0.50
Centroid_Diamide-2	SWI4_SWI6_YAP6	5	184	6	-6.8	0.61	0.85	0.72	0.43
Centroid_Diamide-2	MCM1_NDD1_SWI4_SWI6_YAP6	3	184	3	-4.5	0.36	0.85	0.42	0.19
Centroid_Diamide-3	ARO80	4	281	4	-5.3	0.94	0.79	1.19	0.04
Centroid_Diamide-3	MSN2_MSN4_SKN7	6	281	7	-7.2	0.94	0.79	1.19	0.07
Centroid_Diamide-3	MSN2_MSN4	13	281	27	-10.4	0.94	0.79	1.18	0.10
Centroid_Diamide-3	MSN4_SKN7	8	281	10	-9.1	0.93	0.79	1.18	0.08
Centroid_Diamide-3	MSN4_SKN7_STP1	4	281	4	-5.3	0.92	0.79	1.17	0.08
Centroid_Diamide-3	RPH1	10	281	31	-6.1	0.92	0.79	1.16	0.11
Centroid_Diamide-3	MSN4	22	281	55	-15.1	0.92	0.79	1.16	0.11
Centroid_Diamide-3	MSN2_SKN7	8	281	18	-6.2	0.91	0.79	1.15	0.12
Centroid_Diamide-3	MSN2	29	281	95	-16.1	0.90	0.79	1.13	0.21
Centroid_Diamide-3	SKN7	18	281	95	-6.5	0.89	0.79	1.13	0.13
Centroid_Diamide-3	HSF1_MSN4	5	281	9	-4.6	0.86	0.79	1.08	0.16
Centroid_Diamide-3	SNT2	10	281	49	-4.2	0.86	0.79	1.08	0.28
Centroid_Diamide-3	HSF1	13	281	74	-4.5	0.78	0.79	0.98	0.31
Centroid_Diamide-3	YAP1	17	281	102	-5.4	0.77	0.79	0.97	0.32
Centroid_Diamide-3	CAD1_YAP1	8	281	28	-4.5	0.74	0.79	0.93	0.38
Centroid_Diamide-3	CAD1	11	281	43	-5.5	0.73	0.79	0.92	0.32
Centroid_Diamide-3	MIG1	9	281	39	-4.2	0.72	0.79	0.91	0.36
Centroid_Diamide-3	YAP7	12	281	67	-4.3	0.66	0.79	0.83	0.33
Centroid_Diamide-4	HAP4	12	51	62	-13.3	0.66	0.60	1.10	0.47
Centroid_Diamide-4	HAP1_HAP4	4	51	12	-5.7	0.63	0.60	1.06	0.49
Centroid_Diamide-4	HAP2_HAP3_HAP4	2	51	2	-4.2	0.39	0.60	0.66	0.36
Centroid_Diauxic_shift-0	RPH1	9	384	31	-4.0	0.91	0.71	1.27	0.20
Centroid_Diauxic_shift-0	MSN2_MSN4	12	384	27	-7.5	0.86	0.71	1.21	0.24
Centroid_Diauxic_shift-0	MSN4	19	384	55	-9.4	0.86	0.71	1.20	0.28
Centroid_Diauxic_shift-0	SKN7	22	384	95	-7.1	0.83	0.71	1.17	0.28
Centroid_Diauxic_shift-0	MSN2	32	384	95	-15.1	0.82	0.71	1.15	0.29
Centroid_Diauxic_shift-0	HAP1_HAP4	9	384	12	-8.5	0.81	0.71	1.14	0.27
Centroid_Diauxic_shift-0	HSF1_MSN2	6	384	13	-4.1	0.79	0.71	1.11	0.36
Centroid_Diauxic_shift-0	HAP4	26	384	62	-15.0	0.75	0.71	1.05	0.31
Centroid_Diauxic_shift-0	HSF1	21	384	74	-8.5	0.73	0.71	1.02	0.36
Centroid_Diauxic_shift-1	FHL1_RAP1_SFP1_YAP6	5	802	5	-4.4	0.98	0.83	1.18	0.03
Centroid_Diauxic_shift-1	FHL1_RAP1_YAP6	7	802	8	-5.3	0.93	0.83	1.12	0.15
Centroid_Diauxic_shift-1	FHL1_SFP1_YAP6	9	802	10	-6.9	0.92	0.83	1.12	0.11
Centroid_Diauxic_shift-1	FHL1_SFP1_YAP1	6	802	7	-4.5	0.92	0.83	1.11	0.13
Centroid_Diauxic_shift-1	RAP1	53	802	109	-18.6	0.92	0.83	1.11	0.14
Centroid_Diauxic_shift-1	FHL1_RAP1	45	802	66	-24.1	0.91	0.83	1.10	0.14
Centroid_Diauxic_shift-1	FHL1_YAP6	19	802	23	-13.0	0.90	0.83	1.09	0.12
Centroid_Diauxic_shift-1	YAP1_YAP6	11	802	18	-5.5	0.90	0.83	1.08	0.12
Centroid_Diauxic_shift-1	FHL1_RAP1_SFP1	21	802	30	-11.8	0.90	0.83	1.08	0.17
Centroid_Diauxic_shift-1	RAP1_SFP1	21	802	32	-11.0	0.90	0.83	1.08	0.17
Centroid_Diauxic_shift-1	FHL1_YAP1_YAP6	10	802	11	-7.8	0.89	0.83	1.08	0.12
Centroid_Diauxic_shift-1	FHL1	144	802	252	-63.3	0.89	0.83	1.07	0.16
Centroid_Diauxic_shift-1	YAP6	28	802	48	-12.6	0.89	0.83	1.07	0.17
Centroid_Diauxic_shift-1	FHL1_SFP1	40	802	67	-18.3	0.88	0.83	1.06	0.16
Centroid_Diauxic_shift-1	SFP1	41	802	73	-17.4	0.88	0.83	1.06	0.16
Centroid_Diauxic_shift-1	ACE2	32	802	93	-6.8	0.88	0.83	1.06	0.21
Centroid_Diauxic_shift-1	YAP1	31	802	102	-5.3	0.85	0.83	1.02	0.28
Centroid_Diauxic_shift-1	STB1_SWI4_SWI6	11	802	21	-4.7	0.83	0.83	1.00	0.20
Centroid_Diauxic_shift-1	STB1_SWI6	12	802	22	-5.3	0.82	0.83	0.99	0.19
Centroid_Diauxic_shift-1	STE12_SWI4_SWI6	13	802	25	-5.4	0.80	0.83	0.96	0.19
Centroid_Diauxic_shift-1	STE12_SWI6	13	802	29	-4.5	0.80	0.83	0.96	0.19
Centroid_Diauxic_shift-1	STE12_SWI4	13	802	31	-4.1	0.80	0.83	0.96	0.19
Centroid_Diauxic_shift-1	SWI4	43	802	149	-6.4	0.78	0.83	0.94	0.28
Centroid_Diauxic_shift-1	STE12	43	802	157	-5.8	0.77	0.83	0.93	0.29
Centroid_Diauxic_shift-1	SWI6	36	802	120	-5.9	0.76	0.83	0.92	0.28
Centroid_Diauxic_shift-1	NDD1_STE12	11	802	23	-4.2	0.75	0.83	0.91	0.29
Centroid_Diauxic_shift-1	SWI4_SWI6	31	802	86	-7.2	0.75	0.83	0.90	0.30
Centroid_Diauxic_shift-1	GCR2	10	802	15	-5.6	0.72	0.83	0.87	0.25
Centroid_Diauxic_shift-3	SIP4	16	383	36	-9.9	0.74	0.60	1.22	0.34

Cluster	Module	# Overla	Cluster	# Targets	p-value	Avg. MB	Avg. MB	Centrality	SD Centr.
Centroid_Diauxic_shift-3	UME6	25	383	171	-4.2	0.70	0.60	1.16	0.35
Centroid_Diauxic_shift-3	MIG1	12	383	39	-5.5	0.66	0.60	1.09	0.50
Centroid_Diauxic_shift-3	HAP4	15	383	62	-5.3	0.53	0.60	0.87	0.40
Centroid_Diauxic_shift-4	BAS1	10	47	30	-14.1	0.85	0.58	1.46	0.29
Centroid_Diauxic_shift-9	CAD1_HAP1_MSN2_RCS1_RPH1_SKN7_YAP7	2	19	2	-5.0	0.76	0.47	1.61	0.63
Centroid_Diauxic_shift-9	CAD1_MSN2_SKN7_YAP7	2	19	3	-4.5	0.76	0.47	1.61	0.63
Centroid_Diauxic_shift-9	CAD1_RCS1_YAP7	2	19	3	-4.5	0.76	0.47	1.61	0.63
Centroid_Diauxic_shift-9	MSN2_RCS1_RPH1	2	19	3	-4.5	0.76	0.47	1.61	0.63
Centroid_Diauxic_shift-9	MSN2_RPH1_SKN7	2	19	3	-4.5	0.76	0.47	1.61	0.63
Centroid_Diauxic_shift-9	CAD1_MSN2_YAP7	2	19	4	-4.2	0.76	0.47	1.61	0.63
Centroid_Diauxic_shift-9	CAD1_HAP1_YAP7	2	19	4	-4.2	0.76	0.47	1.61	0.63
Centroid_Diauxic_shift-9	CAD1_MSN2_SKN7	2	19	4	-4.2	0.76	0.47	1.61	0.63
Centroid_Diauxic_shift-9	MSN2_SKN7_YAP7	2	19	4	-4.2	0.76	0.47	1.61	0.63
Centroid_Diauxic_shift-9	RCS1	4	19	69	-4.3	0.57	0.47	1.21	0.65
Centroid_DTT-1-0	MSN2_SKN7	6	107	18	-6.4	0.85	0.68	1.26	0.30
Centroid_DTT-1-0	YAP1	9	107	102	-4.1	0.85	0.68	1.25	0.26
Centroid_DTT-1-0	MSN2	10	107	95	-5.2	0.78	0.68	1.16	0.40
Centroid_DTT-1-0	MSN2_SKN7_YAP7	3	107	4	-4.7	0.74	0.68	1.09	0.36
Centroid_DTT-1-0	SKN7	11	107	95	-6.1	0.72	0.68	1.07	0.44
Centroid_DTT-1-0	CAD1_MSN2_YAP7	3	107	4	-4.7	0.66	0.68	0.98	0.35
Centroid_DTT-1-0	STE12	11	107	157	-4.0	0.50	0.68	0.73	0.42
Centroid_DTT-1-1	FHL1_RAP1_SFP1_YAP1_YAP6	3	192	3	-4.5	0.99	0.68	1.45	0.01
Centroid_DTT-1-1	FHL1_SFP1_YAP1_YAP6	4	192	6	-4.8	0.98	0.68	1.43	0.04
Centroid_DTT-1-1	FHL1_SFP1_YAP1	4	192	7	-4.5	0.98	0.68	1.43	0.04
Centroid_DTT-1-1	FHL1_RAP1_SFP1_YAP6	4	192	5	-5.3	0.97	0.68	1.41	0.07
Centroid_DTT-1-1	FHL1_SFP1_YAP6	7	192	10	-8.5	0.96	0.68	1.40	0.06
Centroid_DTT-1-1	ACE2_FHL1_RAP1	4	192	6	-4.8	0.94	0.68	1.38	0.04
Centroid_DTT-1-1	CIN5_FHL1_YAP6	4	192	8	-4.2	0.92	0.68	1.34	0.19
Centroid_DTT-1-1	FHL1_YAP1_YAP6	7	192	11	-8.0	0.92	0.68	1.34	0.16
Centroid_DTT-1-1	YAP1_YAP6	7	192	18	-6.1	0.92	0.68	1.34	0.16
Centroid_DTT-1-1	FHL1_NDD1_YAP1_YAP6	3	192	3	-4.5	0.92	0.68	1.34	0.13
Centroid_DTT-1-1	FHL1_YAP6	14	192	23	-15.3	0.87	0.68	1.27	0.30
Centroid_DTT-1-1	YAP6	14	192	48	-9.9	0.87	0.68	1.27	0.30
Centroid_DTT-1-1	YAP1	13	192	102	-4.7	0.86	0.68	1.26	0.31
Centroid_DTT-1-1	SFP1	32	192	73	-28.8	0.86	0.68	1.25	0.27
Centroid_DTT-1-1	FHL1_SFP1	31	192	67	-28.8	0.86	0.68	1.25	0.28
Centroid_DTT-1-1	FHL1_RAP1_SFP1	17	192	30	-17.8	0.83	0.68	1.21	0.33
Centroid_DTT-1-1	RAP1_SFP1	17	192	32	-17.2	0.83	0.68	1.21	0.33
Centroid_DTT-1-1	FHL1	90	192	252	-76.4	0.82	0.68	1.20	0.31
Centroid_DTT-1-1	FHL1_RAP1_YAP6	5	192	8	-5.8	0.82	0.68	1.20	0.49
Centroid_DTT-1-1	RAP1	32	192	109	-22.3	0.78	0.68	1.14	0.39
Centroid_DTT-1-1	FHL1_RAP1	31	192	66	-29.0	0.77	0.68	1.13	0.39
Centroid_DTT-1-1	ACE2	14	192	93	-5.9	0.69	0.68	1.02	0.45
Centroid_DTT-1-1	FHL1_PDR1_YAP6_YAP7	3	192	3	-4.5	0.65	0.68	0.95	0.56
Centroid_DTT-1-12	YAP1_YAP7	4	11	34	-6.6	0.57	0.59	0.97	0.42
Centroid_DTT-1-12	YAP1	4	11	102	-4.6	0.57	0.59	0.97	0.42
Centroid_DTT-1-12	MSN2_MSN4	3	11	27	-4.9	0.56	0.59	0.95	0.54
Centroid_DTT-1-12	MSN4	4	11	55	-5.7	0.55	0.59	0.93	0.44
Centroid_DTT-1-12	CAD1_YAP1_YAP7	3	11	20	-5.3	0.51	0.59	0.87	0.46
Centroid_DTT-1-12	CAD1_YAP1	3	11	28	-4.8	0.51	0.59	0.87	0.46
Centroid_DTT-1-12	YAP7	5	11	67	-7.2	0.50	0.59	0.85	0.45
Centroid_DTT-1-12	MSN2	4	11	95	-4.8	0.47	0.59	0.80	0.53
Centroid_DTT-1-12	CAD1_YAP7	4	11	29	-6.9	0.44	0.59	0.75	0.45
Centroid_DTT-1-12	CAD1	4	11	43	-6.1	0.44	0.59	0.75	0.45
Centroid_DTT-1-12	CAD1_MSN2_SKN7_YAP7	2	11	3	-5.0	0.44	0.59	0.74	0.53
Centroid_DTT-1-12	CAD1_MSN2_SKN7	2	11	4	-4.7	0.44	0.59	0.74	0.53
Centroid_DTT-1-12	MSN2_SKN7_YAP7	2	11	4	-4.7	0.44	0.59	0.74	0.53
Centroid_DTT-1-12	CAD1_MSN2_MSN4_YAP1_YAP7	2	11	2	-5.5	0.43	0.59	0.73	0.55
Centroid_DTT-1-12	CAD1_MSN2_MSN4_YAP1	2	11	3	-5.0	0.43	0.59	0.73	0.55
Centroid_DTT-1-12	CAD1_MSN2_YAP1_YAP7	2	11	3	-5.0	0.43	0.59	0.73	0.55
Centroid_DTT-1-12	CAD1_MSN2_YAP1	2	11	4	-4.7	0.43	0.59	0.73	0.55
Centroid_DTT-1-12	MSN2_MSN4_YAP1_YAP7	2	11	5	-4.5	0.43	0.59	0.73	0.55
Centroid_DTT-1-12	MSN2_MSN4_YAP1	2	11	6	-4.3	0.43	0.59	0.73	0.55
Centroid_DTT-1-12	MSN2_YAP1_YAP7	2	11	7	-4.2	0.43	0.59	0.73	0.55
Centroid_DTT-1-12	CAD1_MSN2_YAP7	3	11	4	-7.7	0.36	0.59	0.61	0.44
Centroid_DTT-1-3	FHL1	27	228	252	-6.1	0.63	0.73	0.87	0.34
Centroid_DTT-2-1	MSN2	20	421	95	-5.2	0.49	0.54	0.90	0.47
Centroid_DTT-2-1	MSN2_SKN7	8	421	18	-4.9	0.47	0.54	0.88	0.50
Centroid_DTT-2-12	MCM1_NDD1_STE12_SWI4_SWI6	3	87	7	-4.0	0.91	0.45	2.04	0.27
Centroid_DTT-2-12	STE12_SWI4_SWI6	6	87	25	-6.0	0.74	0.45	1.64	0.67
Centroid_DTT-2-12	STE12_SWI4	7	87	31	-6.7	0.68	0.45	1.52	0.70
Centroid_DTT-2-12	STE12_SWI6	7	87	29	-6.9	0.68	0.45	1.51	0.71
Centroid_DTT-2-12	SKN7_STE12_SWI4_SWI6	3	87	4	-4.9	0.67	0.45	1.50	0.84
Centroid_DTT-2-12	SKN7_SWI4_SWI6	3	87	6	-4.2	0.67	0.45	1.50	0.84
Centroid_DTT-2-12	DIG1_STE12_SWI6	4	87	11	-4.9	0.51	0.45	1.13	0.73
Centroid_DTT-2-2	RCS1	16	456	69	-4.4	0.73	0.68	1.06	0.36
Centroid_DTT-2-2	FHL1	44	456	252	-7.0	0.65	0.68	0.95	0.39
Centroid_DTT-2-3	SIP4	8	246	36	-4.1	0.42	0.51	0.83	0.29
Centroid_DTT-2-4	CIN5_FHL1_YAP1_YAP6	4	482	4	-4.4	0.99	0.70	1.41	0.02
Centroid_DTT-2-4	CIN5_FHL1_YAP6	6	482	8	-5.2	0.98	0.70	1.39	0.03
Centroid_DTT-2-4	ACE2_FHL1_RAP1	6	482	6	-6.6	0.95	0.70	1.36	0.03
Centroid_DTT-2-4	HIR1_HIR2_HIR3_SWI4	4	482	4	-4.4	0.95	0.70	1.35	0.06
Centroid_DTT-2-4	HIR1_HIR2_HIR3	5	482	5	-5.5	0.95	0.70	1.35	0.05
Centroid_DTT-2-4	HIR3	5	482	6	-4.7	0.95	0.70	1.35	0.05
Centroid_DTT-2-4	FHL1_YAP1_YAP6	11	482	11	-12.1	0.94	0.70	1.34	0.18
Centroid_DTT-2-4	FHL1_RAP1_SFP1	19	482	30	-13.6	0.93	0.70	1.33	0.16
Centroid_DTT-2-4	RAP1_SFP1	19	482	32	-12.9	0.93	0.70	1.33	0.16
Centroid_DTT-2-4	FHL1_SFP1_YAP6	9	482	10	-8.9	0.93	0.70	1.32	0.20
Centroid_DTT-2-4	HIR1_HIR2_SWI4	6	482	6	-6.6	0.93	0.70	1.32	0.09
Centroid_DTT-2-4	HIR1	10	482	12	-9.2	0.92	0.70	1.32	0.10
Centroid_DTT-2-4	FHL1_YAP6	19	482	23	-17.1	0.92	0.70	1.31	0.17
Centroid_DTT-2-4	FHL1_SFP1_YAP1	7	482	7	-7.7	0.92	0.70	1.31	0.23
Centroid_DTT-2-4	FHL1_RAP1_YAP6	6	482	8	-5.2	0.91	0.70	1.30	0.25

Cluster	Module	# Overla	Cluster	# Targets	p-value	Avg. MB	Avg. MB	Centrality	SD Centr.
Centroid_DTT-2-4	FHL1_RAP1	40	482	66	-27.1	0.91	0.70	1.30	0.21
Centroid_DTT-2-4	HIR1_HIR2	8	482	8	-8.8	0.91	0.70	1.30	0.10
Centroid_DTT-2-4	FHL1_SFP1_YAP1_YAP6	6	482	6	-6.6	0.91	0.70	1.29	0.24
Centroid_DTT-2-4	CIN5_YAP6	8	482	14	-5.5	0.91	0.70	1.29	0.25
Centroid_DTT-2-4	YAP1_YAP6	12	482	18	-9.1	0.90	0.70	1.29	0.26
Centroid_DTT-2-4	YAP6	24	482	48	-13.8	0.90	0.70	1.29	0.20
Centroid_DTT-2-4	FHL1_RAP1_SFP1_YAP6	5	482	5	-5.5	0.90	0.70	1.28	0.27
Centroid_DTT-2-4	FHL1_SFP1	38	482	67	-24.3	0.90	0.70	1.28	0.26
Centroid_DTT-2-4	RAP1	45	482	109	-21.3	0.89	0.70	1.27	0.24
Centroid_DTT-2-4	SFP1	39	482	73	-23.6	0.89	0.70	1.27	0.26
Centroid_DTT-2-4	HIR2	9	482	9	-9.9	0.89	0.70	1.27	0.13
Centroid_DTT-2-4	CIN5_YAP1_YAP6	5	482	6	-4.7	0.89	0.70	1.27	0.33
Centroid_DTT-2-4	FHL1_RAP1_SFP1_YAP1	4	482	4	-4.4	0.89	0.70	1.26	0.31
Centroid_DTT-2-4	FHL1	129	482	252	-77.7	0.88	0.70	1.26	0.26
Centroid_DTT-2-4	YAP1	28	482	102	-8.5	0.81	0.70	1.15	0.38
Centroid_DTT-2-4	ACE2	25	482	93	-7.4	0.80	0.70	1.14	0.34
Centroid_DTT-2-6	MCM1	7	57	103	-4.3	0.56	0.47	1.18	0.46
Centroid_DTT-2-6	MCM1_NDD1	6	57	51	-5.1	0.55	0.47	1.17	0.51
Centroid_DTT-2-6	FKH2_MCM1_NDD1	5	57	26	-5.4	0.51	0.47	1.07	0.50
Centroid_DTT-2-6	NDD1	11	57	143	-7.2	0.51	0.47	1.07	0.43
Centroid_DTT-2-6	FKH2_NDD1	6	57	48	-5.3	0.47	0.47	0.98	0.49
Centroid_DTT-2-6	FKH2	11	57	145	-7.1	0.44	0.47	0.92	0.42
Centroid_Gamma-0	ACE2	10	66	93	-7.3	0.80	0.58	1.37	0.47
Centroid_Gamma-0	ACE2_SWI5	6	66	13	-8.6	0.78	0.58	1.34	0.52
Centroid_Gamma-0	SWI5	11	66	44	-12.2	0.78	0.58	1.33	0.50
Centroid_Gamma-0	ACE2_FKH2_STE12_SWI5	3	66	3	-5.9	0.75	0.58	1.29	0.68
Centroid_Gamma-0	ACE2_STE12_SWI5	3	66	4	-5.3	0.75	0.58	1.29	0.68
Centroid_Gamma-0	ACE2_FKH1_FKH2	3	66	5	-4.9	0.74	0.58	1.27	0.66
Centroid_Gamma-0	FKH1_FKH2	5	66	31	-4.7	0.72	0.58	1.24	0.53
Centroid_Gamma-0	ACE2_FKH2_SWI5	4	66	5	-7.2	0.69	0.58	1.18	0.60
Centroid_Gamma-0	FKH2	9	66	145	-4.6	0.68	0.58	1.17	0.46
Centroid_Gamma-0	HIR1_HIR2_SWI4_YAP1	3	66	3	-5.9	0.53	0.58	0.91	0.47
Centroid_Gamma-0	HIR1_HIR2_SWI4	4	66	6	-6.7	0.53	0.58	0.91	0.38
Centroid_Gamma-0	HIR1_HIR2	5	66	8	-8.1	0.46	0.58	0.80	0.41
Centroid_Gamma-0	HIR2	5	66	9	-7.8	0.46	0.58	0.80	0.41
Centroid_Gamma-0	HIR1	5	66	12	-7.0	0.46	0.58	0.80	0.41
Centroid_Gamma-1	MSN4	7	108	55	-4.3	0.62	0.59	1.04	0.36
Centroid_Gamma-15	CBF1_MET31_MET32_MET4_TYE7	2	24	3	-4.3	0.95	0.56	1.70	0.10
Centroid_Gamma-15	MET31_MET32_MET4_TYE7	2	24	4	-4.0	0.95	0.56	1.70	0.10
Centroid_Gamma-15	CBF1_MET31_MET32_TYE7	3	24	4	-6.7	0.92	0.56	1.64	0.13
Centroid_Gamma-15	MET31_MET32_TYE7	3	24	5	-6.3	0.92	0.56	1.64	0.13
Centroid_Gamma-15	CBF1_MET32_MET4_TYE7	3	24	4	-6.7	0.88	0.56	1.58	0.22
Centroid_Gamma-15	MET32_MET4_TYE7	3	24	5	-6.3	0.88	0.56	1.58	0.22
Centroid_Gamma-15	CBF1_MET32_TYE7	4	24	7	-8.2	0.88	0.56	1.56	0.18
Centroid_Gamma-15	CBF1_TYE7	4	24	40	-4.8	0.88	0.56	1.56	0.18
Centroid_Gamma-15	TYE7	5	24	62	-5.4	0.80	0.56	1.42	0.36
Centroid_Gamma-15	CBF1_MET31_MET32_MET4	3	24	7	-5.7	0.79	0.56	1.42	0.50
Centroid_Gamma-15	CBF1_MET32_MET4	5	24	9	-10.1	0.75	0.56	1.35	0.37
Centroid_Gamma-15	MET31_MET32_MET4	4	24	15	-6.6	0.73	0.56	1.30	0.47
Centroid_Gamma-15	MET31_MET4	4	24	16	-6.5	0.73	0.56	1.30	0.47
Centroid_Gamma-15	CBF1	9	24	156	-8.4	0.72	0.56	1.28	0.37
Centroid_Gamma-15	MET32_MET4	6	24	17	-10.6	0.72	0.56	1.28	0.37
Centroid_Gamma-15	MET4	6	24	35	-8.5	0.72	0.56	1.28	0.37
Centroid_Gamma-15	MET31	8	24	49	-11.1	0.71	0.56	1.26	0.41
Centroid_Gamma-15	CBF1_MET31_MET32	6	24	14	-11.2	0.70	0.56	1.25	0.44
Centroid_Gamma-15	CBF1_MET32	8	24	20	-14.7	0.70	0.56	1.25	0.37
Centroid_Gamma-15	MET32	9	24	64	-11.9	0.68	0.56	1.22	0.36
Centroid_Gamma-15	MET31_MET32	7	24	47	-9.5	0.68	0.56	1.21	0.42
Centroid_Gamma-3	KSS1	3	186	3	-4.5	0.79	0.74	1.06	0.43
Centroid_Gamma-5	RCS1	7	65	69	-5.1	0.82	0.53	1.53	0.36
Centroid_Gamma-6	MSN4	13	192	55	-8.0	0.86	0.69	1.24	0.36
Centroid_Gamma-6	MSN2	19	192	95	-10.1	0.80	0.69	1.16	0.32
Centroid_Gamma-6	SKN7	12	192	95	-4.4	0.65	0.69	0.95	0.44
Centroid_Gamma-6	GCN4	13	192	76	-6.2	0.55	0.69	0.79	0.35
Centroid_Gamma-9	GCN4	6	33	76	-5.5	0.45	0.50	0.89	0.40
Centroid_H2O2-0	FHL1_SFP1	19	460	67	-6.5	0.78	0.65	1.20	0.30
Centroid_H2O2-0	SFP1	19	460	73	-5.9	0.78	0.65	1.20	0.30
Centroid_H2O2-0	FHL1_RAP1_SFP1	11	460	30	-5.2	0.78	0.65	1.19	0.31
Centroid_H2O2-0	RAP1_SFP1	11	460	32	-4.9	0.78	0.65	1.19	0.31
Centroid_H2O2-0	HIR1_HIR2_HIR3	5	460	5	-5.6	0.77	0.65	1.19	0.23
Centroid_H2O2-0	HIR3	5	460	6	-4.8	0.77	0.65	1.19	0.23
Centroid_H2O2-0	HIR1	9	460	12	-7.8	0.77	0.65	1.18	0.26
Centroid_H2O2-0	FHL1_RAP1	26	460	66	-12.3	0.77	0.65	1.18	0.32
Centroid_H2O2-0	RAP1	29	460	109	-8.9	0.76	0.65	1.17	0.33
Centroid_H2O2-0	FHL1	78	460	252	-28.4	0.75	0.65	1.15	0.32
Centroid_H2O2-0	HIR1_HIR2	8	460	8	-9.0	0.74	0.65	1.14	0.24
Centroid_H2O2-0	HIR2	8	460	9	-8.0	0.74	0.65	1.14	0.24
Centroid_H2O2-0	HIR1_HIR2_HIR3_SWI4	4	460	4	-4.5	0.74	0.65	1.14	0.23
Centroid_H2O2-0	ACE2	19	460	93	-4.2	0.71	0.65	1.10	0.36
Centroid_H2O2-0	YAP1	20	460	102	-4.1	0.70	0.65	1.07	0.36
Centroid_H2O2-0	FHL1_YAP6	13	460	23	-8.8	0.70	0.65	1.07	0.37
Centroid_H2O2-0	HIR1_HIR2_SWI4	6	460	6	-6.7	0.69	0.65	1.06	0.22
Centroid_H2O2-0	FHL1_SFP1_YAP1	5	460	7	-4.3	0.68	0.65	1.05	0.36
Centroid_H2O2-0	FHL1_YAP1_YAP6	7	460	11	-5.4	0.68	0.65	1.05	0.30
Centroid_H2O2-0	YAP6	15	460	48	-5.8	0.67	0.65	1.03	0.38
Centroid_H2O2-0	FKH2	26	460	145	-4.5	0.67	0.65	1.03	0.35
Centroid_H2O2-0	ROX1	11	460	30	-5.2	0.66	0.65	1.02	0.37
Centroid_H2O2-0	FHL1_SFP1_YAP6	6	460	10	-4.5	0.63	0.65	0.97	0.35
Centroid_H2O2-0	RME1	8	460	18	-4.6	0.61	0.65	0.94	0.34
Centroid_H2O2-0	NDD1	25	460	143	-4.2	0.59	0.65	0.91	0.38
Centroid_H2O2-0	FKH2_NDD1	13	460	48	-4.4	0.57	0.65	0.87	0.32
Centroid_H2O2-16	FHL1	11	44	252	-5.9	0.64	0.52	1.24	0.44
Centroid_H2O2-22	HSF1	6	19	74	-7.2	0.36	0.42	0.85	0.24

Cluster	Module	# Overla	Cluster	# Targets	p-value	Avg. MB	Avg. MB	Centrality	SD Centr.
Centroid_H2O2-22	HSF1_MSN2	3	19	13	-5.1	0.32	0.42	0.75	0.17
Centroid_H2O2-24	CIN5_YAP6	4	65	14	-4.9	0.59	0.43	1.37	0.42
Centroid_H2O2-24	CIN5_SWI4_SWI6_YAP6	3	65	4	-5.3	0.51	0.43	1.20	0.30
Centroid_H2O2-24	SWI4_SWI6_YAP6	3	65	6	-4.6	0.51	0.43	1.20	0.30
Centroid_H2O2-24	FHL1_RAP1_YAP6	3	65	8	-4.2	0.49	0.43	1.14	0.71
Centroid_H2O2-24	FHL1	12	65	252	-4.9	0.46	0.43	1.07	0.55
Centroid_H2O2-24	NDD1_RLM1_SWI4_SWI6	3	65	3	-5.9	0.45	0.43	1.05	0.48
Centroid_H2O2-24	RLM1_SWI4_SWI6	3	65	4	-5.3	0.45	0.43	1.05	0.48
Centroid_H2O2-4	RPH1	8	235	31	-4.8	0.75	0.48	1.55	0.44
Centroid_H2O2-4	MSN2_RCS1_RPH1	3	235	3	-4.2	0.73	0.48	1.52	0.47
Centroid_H2O2-4	MSN2	18	235	95	-7.7	0.63	0.48	1.32	0.56
Centroid_H2O2-4	CAD1_RCS1_YAP1	4	235	5	-5.0	0.63	0.48	1.31	0.49
Centroid_H2O2-4	CAD1_RCS1_YAP7	3	235	3	-4.2	0.59	0.48	1.22	0.42
Centroid_H2O2-4	MSN4	12	235	55	-6.0	0.57	0.48	1.19	0.55
Centroid_H2O2-4	RCS1	12	235	69	-4.9	0.56	0.48	1.16	0.48
Centroid_H2O2-4	CAD1	18	235	43	-14.2	0.56	0.48	1.15	0.39
Centroid_H2O2-4	CAD1_RPN4_YAP1_YAP7	3	235	3	-4.2	0.54	0.48	1.13	0.12
Centroid_H2O2-4	CAD1_YAP1	14	235	28	-12.5	0.54	0.48	1.12	0.38
Centroid_H2O2-4	YAP7	22	235	67	-14.6	0.54	0.48	1.12	0.39
Centroid_H2O2-4	CAD1_YAP7	14	235	29	-12.2	0.54	0.48	1.12	0.34
Centroid_H2O2-4	YAP1	23	235	102	-11.4	0.51	0.48	1.06	0.45
Centroid_H2O2-4	CAD1_YAP1_YAP7	12	235	20	-12.0	0.50	0.48	1.04	0.29
Centroid_H2O2-4	YAP1_YAP7	15	235	34	-12.3	0.50	0.48	1.03	0.40
Centroid_H2O2-6	RPN4	17	218	137	-5.1	0.40	0.47	0.86	0.37
Centroid_H2O2-7	MSN2_SKN7	6	153	18	-5.4	0.71	0.50	1.42	0.60
Centroid_H2O2-7	SKN7	12	153	95	-5.4	0.63	0.50	1.26	0.54
Centroid_H2O2-7	MSN2_MSN4	7	153	27	-5.5	0.63	0.50	1.26	0.78
Centroid_H2O2-7	MSN2	19	153	95	-11.8	0.61	0.50	1.23	0.65
Centroid_H2O2-7	MSN4	9	153	55	-5.1	0.57	0.50	1.14	0.72
Centroid_H2O2-8	AZF1	6	108	42	-4.0	0.45	0.54	0.84	0.52
Centroid_HS_25_37-2-0	MSN2_MSN4	14	823	27	-5.6	0.87	0.69	1.26	0.26
Centroid_HS_25_37-2-0	MSN2_SKN7	10	823	18	-4.5	0.86	0.69	1.26	0.33
Centroid_HS_25_37-2-0	MSN2	41	823	95	-11.9	0.82	0.69	1.20	0.31
Centroid_HS_25_37-2-0	MSN4	24	823	55	-7.3	0.81	0.69	1.19	0.27
Centroid_HS_25_37-2-0	HSF1_MSN4	7	823	9	-4.6	0.76	0.69	1.11	0.29
Centroid_HS_25_37-2-0	SKN7	32	823	95	-6.3	0.75	0.69	1.10	0.37
Centroid_HS_25_37-2-0	HSF1	38	823	74	-14.0	0.71	0.69	1.04	0.38
Centroid_HS_25_37-2-0	UME6	47	823	171	-6.0	0.71	0.69	1.03	0.38
Centroid_HS_25_37-2-0	HSF1_MSN2	10	823	13	-6.4	0.70	0.69	1.01	0.33
Centroid_HS_25_37-2-0	MIG1	16	823	39	-4.6	0.69	0.69	1.01	0.40
Centroid_HS_25_37-2-1	FKH2_MBP1_SWI4_SWI6	5	320	8	-4.7	0.75	0.55	1.36	0.52
Centroid_HS_25_37-2-1	MBP1_SWI4_SWI6	13	320	34	-8.1	0.71	0.55	1.29	0.36
Centroid_HS_25_37-2-1	NDD1_STE12_SWI4_SWI6	5	320	10	-4.1	0.71	0.55	1.29	0.52
Centroid_HS_25_37-2-1	FKH2_SWI4_SWI6	7	320	17	-4.9	0.69	0.55	1.25	0.55
Centroid_HS_25_37-2-1	FKH2_SWI6	7	320	21	-4.2	0.69	0.55	1.25	0.55
Centroid_HS_25_37-2-1	NDD1_STE12_SWI4	6	320	12	-4.8	0.68	0.55	1.25	0.47
Centroid_HS_25_37-2-1	NDD1_SWI6	11	320	27	-7.3	0.68	0.55	1.24	0.42
Centroid_HS_25_37-2-1	DIG1_SWI4_SWI6	7	320	10	-6.9	0.68	0.55	1.23	0.49
Centroid_HS_25_37-2-1	MBP1_SWI6	18	320	53	-10.1	0.68	0.55	1.23	0.44
Centroid_HS_25_37-2-1	MBP1_NDD1_SWI6	8	320	15	-6.6	0.68	0.55	1.23	0.36
Centroid_HS_25_37-2-1	SWI4_SWI6	28	320	86	-14.9	0.67	0.55	1.22	0.43
Centroid_HS_25_37-2-1	NDD1_SWI4_SWI6	10	320	24	-6.8	0.66	0.55	1.21	0.43
Centroid_HS_25_37-2-1	SWI6	34	320	120	-15.9	0.66	0.55	1.21	0.45
Centroid_HS_25_37-2-1	STE12_SWI4_SWI6	10	320	25	-6.6	0.66	0.55	1.20	0.48
Centroid_HS_25_37-2-1	STE12_SWI6	10	320	29	-5.9	0.66	0.55	1.20	0.48
Centroid_HS_25_37-2-1	MBP1_SWI4	19	320	49	-11.8	0.66	0.55	1.20	0.34
Centroid_HS_25_37-2-1	NDD1_SWI4	11	320	28	-7.1	0.66	0.55	1.19	0.41
Centroid_HS_25_37-2-1	STE12_SWI4	11	320	31	-6.6	0.65	0.55	1.19	0.46
Centroid_HS_25_37-2-1	MBP1_NDD1_SWI4_SWI6	7	320	14	-5.5	0.65	0.55	1.18	0.35
Centroid_HS_25_37-2-1	DIG1_STE12_SWI4_SWI6	6	320	9	-5.8	0.64	0.55	1.17	0.51
Centroid_HS_25_37-2-1	DIG1_STE12_SWI6	6	320	11	-5.1	0.64	0.55	1.17	0.51
Centroid_HS_25_37-2-1	DIG1_STE12_SWI4	6	320	11	-5.1	0.64	0.55	1.17	0.51
Centroid_HS_25_37-2-1	NDD1_STE12	8	320	23	-4.8	0.64	0.55	1.17	0.51
Centroid_HS_25_37-2-1	MBP1	30	320	174	-8.2	0.63	0.55	1.14	0.42
Centroid_HS_25_37-2-1	SWI4	38	320	149	-16.0	0.62	0.55	1.13	0.42
Centroid_HS_25_37-2-1	MBP1_STB1	6	320	14	-4.3	0.61	0.55	1.10	0.25
Centroid_HS_25_37-2-1	STE12	27	320	157	-7.4	0.56	0.55	1.02	0.45
Centroid_HS_25_37-2-1	DIG1	15	320	86	-4.4	0.56	0.55	1.02	0.49
Centroid_HS_25_37-2-1	DIG1_STE12	13	320	68	-4.3	0.54	0.55	0.98	0.49
Centroid_HS_25_37-2-1	STB1	12	320	47	-5.4	0.52	0.55	0.94	0.38
Centroid_HS_25_37-2-1	STB1_SWI4	11	320	44	-4.9	0.49	0.55	0.90	0.37
Centroid_HS_25_37-2-2	HSF1	19	451	74	-5.9	0.40	0.59	0.69	0.29
Centroid_HS_25_37-2-3	FHL1_RAP1	47	799	66	-26.6	0.88	0.75	1.17	0.20
Centroid_HS_25_37-2-3	FHL1_RAP1_YAP6	7	799	8	-5.3	0.87	0.75	1.16	0.17
Centroid_HS_25_37-2-3	RAP1	54	799	109	-19.5	0.86	0.75	1.15	0.24
Centroid_HS_25_37-2-3	FHL1_RAP1_SFP1	21	799	30	-11.9	0.85	0.75	1.13	0.24
Centroid_HS_25_37-2-3	RAP1_SFP1	21	799	32	-11.0	0.85	0.75	1.13	0.24
Centroid_HS_25_37-2-3	SFP1	43	799	73	-19.4	0.84	0.75	1.11	0.22
Centroid_HS_25_37-2-3	FHL1_SFP1	42	799	67	-20.4	0.84	0.75	1.11	0.22
Centroid_HS_25_37-2-3	FHL1	147	799	252	-66.6	0.83	0.75	1.11	0.25
Centroid_HS_25_37-2-3	FHL1_RAP1_SFP1_YAP6	5	799	5	-4.4	0.83	0.75	1.10	0.17
Centroid_HS_25_37-2-3	ACE2	31	799	93	-6.3	0.83	0.75	1.10	0.29
Centroid_HS_25_37-2-3	FHL1_SFP1_YAP1	7	799	7	-6.1	0.81	0.75	1.08	0.24
Centroid_HS_25_37-2-3	FHL1_SFP1_YAP6	10	799	10	-8.8	0.81	0.75	1.08	0.20
Centroid_HS_25_37-2-3	CIN5_YAP6	9	799	14	-4.9	0.80	0.75	1.06	0.33
Centroid_HS_25_37-2-3	YAP6	26	799	48	-10.8	0.79	0.75	1.06	0.32
Centroid_HS_25_37-2-3	YAP1_YAP6	12	799	18	-6.6	0.79	0.75	1.05	0.30
Centroid_HS_25_37-2-3	FHL1_YAP6	20	799	23	-14.5	0.79	0.75	1.05	0.30
Centroid_HS_25_37-2-3	FHL1_SFP1_YAP1_YAP6	6	799	6	-5.3	0.79	0.75	1.05	0.24
Centroid_HS_25_37-2-3	RME1	11	799	18	-5.6	0.79	0.75	1.05	0.34
Centroid_HS_25_37-2-3	FHL1_YAP1_YAP6	11	799	11	-9.7	0.77	0.75	1.03	0.31
Centroid_HS_25_37-2-3	YAP1	33	799	102	-6.3	0.77	0.75	1.03	0.34
Centroid_HS_25_37-2-4	ARG81_GCN4_RTG3	3	92	4	-4.9	0.54	0.57	0.96	0.61

Cluster	Module	# Overla	Cluster	# Targets	p-value	Avg. MB	Avg. MB	Centrality	SD Centr.
Centroid_HS_25_37-2-4	ARG81_GCN4	4	92	6	-6.1	0.50	0.57	0.89	0.52
Centroid_HS_25_37-2-4	ARG81	4	92	8	-5.5	0.50	0.57	0.89	0.52
Centroid_HS_25_37-2-6	HSF1	10	123	74	-5.7	0.51	0.46	1.10	0.51
Centroid_HS_25_37-2-7	HIR1_HIR2_SWI4_YAP1	3	131	3	-5.0	0.95	0.48	1.97	0.11
Centroid_HS_25_37-2-7	HIR1_HIR2_SWI4	6	131	6	-10.0	0.94	0.48	1.97	0.14
Centroid_HS_25_37-2-7	HIR1_HIR2_MBP1	3	131	3	-5.0	0.92	0.48	1.92	0.24
Centroid_HS_25_37-2-7	HIR1_HIR2	7	131	8	-10.8	0.92	0.48	1.92	0.18
Centroid_HS_25_37-2-7	HIR2	7	131	9	-10.2	0.92	0.48	1.92	0.18
Centroid_HS_25_37-2-7	HIR1	7	131	12	-8.8	0.92	0.48	1.92	0.18
Centroid_HS_25_37-2-7	HIR1_HIR2_HIR3_SWI4	4	131	4	-6.7	0.92	0.48	1.92	0.16
Centroid_HS_25_37-2-7	HIR1_HIR2_HIR3	5	131	5	-8.3	0.89	0.48	1.86	0.19
Centroid_HS_25_37-2-7	HIR3	5	131	6	-7.6	0.89	0.48	1.86	0.19
Centroid_HS_25_37-2-7	FKH2_HIR1_HIR2_HIR3	3	131	3	-5.0	0.87	0.48	1.82	0.23
Centroid_HS_25_37-2-7	SWI4	14	131	149	-5.4	0.64	0.48	1.33	0.70
Centroid_HS_25_37-2-7	YAP1	11	131	102	-4.9	0.55	0.48	1.15	0.58
Centroid_HS_25_37-2-7	SWI6	13	131	120	-5.8	0.55	0.48	1.14	0.62
Centroid_HS_25_37-2-7	SWI4_SWI6	9	131	86	-4.0	0.53	0.48	1.11	0.69
Centroid_HS_25_37-2-7	FHL1	20	131	252	-6.4	0.46	0.48	0.95	0.47
Centroid_HS_25_37-2-9	RTG3	6	219	16	-4.9	0.45	0.52	0.86	0.51
Centroid_HS_37_25-0	MSN2	21	427	95	-5.7	0.75	0.55	1.36	0.43
Centroid_HS_37_25-0	MSN2_MSN4	9	427	27	-4.2	0.75	0.55	1.36	0.38
Centroid_HS_37_25-0	MSN4	13	427	55	-4.0	0.72	0.55	1.30	0.40
Centroid_HS_37_25-0	HSF1	19	427	74	-6.2	0.70	0.55	1.26	0.45
Centroid_HS_37_25-0	HSF1_MSN2	9	427	13	-7.6	0.64	0.55	1.16	0.50
Centroid_HS_37_25-1	ARG81_GCN4	4	279	6	-4.2	0.50	0.55	0.90	0.33
Centroid_HS_37_25-1	GCN4	14	279	76	-5.1	0.45	0.55	0.81	0.24
Centroid_HS_37_25-2	ACE2_FHL1_RAP1	6	301	6	-7.8	1.00	0.68	1.47	0.00
Centroid_HS_37_25-2	FHL1_SFP1	19	301	67	-9.5	0.98	0.68	1.43	0.11
Centroid_HS_37_25-2	SFP1	19	301	73	-8.8	0.98	0.68	1.43	0.11
Centroid_HS_37_25-2	FHL1_RAP1	23	301	66	-13.6	0.98	0.68	1.43	0.10
Centroid_HS_37_25-2	FHL1_RAP1_SFP1	12	301	30	-8.1	0.97	0.68	1.42	0.14
Centroid_HS_37_25-2	RAP1_SFP1	12	301	32	-7.7	0.97	0.68	1.42	0.14
Centroid_HS_37_25-2	RAP1	25	301	109	-10.1	0.96	0.68	1.41	0.14
Centroid_HS_37_25-2	FHL1_YAP6	8	301	23	-5.0	0.96	0.68	1.41	0.17
Centroid_HS_37_25-2	FHL1	62	301	252	-27.0	0.94	0.68	1.38	0.25
Centroid_HS_37_25-2	YAP1	18	301	102	-5.6	0.81	0.68	1.19	0.35
Centroid_HS_37_25-2	HIR1	7	301	12	-6.3	0.75	0.68	1.10	0.27
Centroid_HS_37_25-2	HIR1_HIR2_SWI4	5	301	6	-5.8	0.73	0.68	1.08	0.24
Centroid_HS_37_25-2	HIR1_HIR2	6	301	8	-6.4	0.71	0.68	1.04	0.24
Centroid_HS_37_25-2	HIR2	6	301	9	-6.0	0.71	0.68	1.04	0.24
Centroid_HS_37_25-3	DAL82_GLN3	6	497	10	-4.3	0.72	0.65	1.10	0.40
Centroid_HS_37_25-3	MBP1	32	497	174	-4.9	0.57	0.65	0.88	0.40
Centroid_HS_37_25-4	MBP1	31	191	174	-14.9	0.58	0.50	1.16	0.52
Centroid_HS_37_25-4	SWI4_SWI6	11	191	86	-4.1	0.57	0.50	1.16	0.32
Centroid_HS_37_25-4	HAP1	14	191	112	-5.0	0.56	0.50	1.13	0.54
Centroid_HS_37_25-4	SWI4	15	191	149	-4.2	0.53	0.50	1.06	0.37
Centroid_HS_37_25-4	SWI6	16	191	120	-6.0	0.51	0.50	1.03	0.42
Centroid_HS_37_25-4	MBP1_SWI6	10	191	53	-5.3	0.49	0.50	0.99	0.49
Centroid_HS_37_25-7	MBP1	17	120	174	-7.3	0.55	0.51	1.08	0.51
Centroid_HS_37_25-8	GCN4	16	175	76	-9.5	0.44	0.46	0.96	0.43
Centroid_HS_mild-0	KSS1	3	217	3	-4.3	0.53	0.64	0.83	0.49
Centroid_HS_mild-10	ARG81_CIN5_GCN4	2	25	2	-4.8	0.43	0.46	0.94	0.55
Centroid_HS_mild-10	CIN5	4	25	58	-4.1	0.39	0.46	0.84	0.37
Centroid_HS_mild-17	STB1_SWI4	4	34	44	-4.0	0.24	0.35	0.69	0.13
Centroid_HS_mild-17	MBP1_STB1_SWI4	3	34	13	-4.3	0.22	0.35	0.63	0.05
Centroid_HS_mild-17	MBP1_STB1	3	34	14	-4.2	0.22	0.35	0.63	0.05
Centroid_HS_mild-3	HSF1	8	116	74	-4.1	0.65	0.51	1.27	0.49
Centroid_HS_mild-5	HAP1_HAP4	5	122	12	-5.6	0.72	0.46	1.57	0.42
Centroid_HS_mild-5	HAP4	11	122	62	-7.5	0.70	0.46	1.54	0.56
Centroid_HS_mild-7	MSN2_MSN4_SKN7	4	198	7	-4.4	0.88	0.54	1.64	0.12
Centroid_HS_mild-7	MSN2_MSN4	10	198	27	-8.2	0.84	0.54	1.56	0.32
Centroid_HS_mild-7	MSN2_SKN7	7	198	18	-6.1	0.81	0.54	1.51	0.44
Centroid_HS_mild-7	MSN4	11	198	55	-5.9	0.80	0.54	1.48	0.39
Centroid_HS_mild-7	MSN2	20	198	95	-10.8	0.78	0.54	1.45	0.46
Centroid_HS_mild-7	SKN7	14	198	95	-5.7	0.70	0.54	1.30	0.45
Centroid_HS_mild-7	RPH1	7	198	31	-4.3	0.62	0.54	1.15	0.49
Centroid_HS_mild-7	HSF1	14	198	74	-7.0	0.54	0.54	1.01	0.55
Centroid_HS_various_temp-0	ACE2_FHL1_RAP1	6	803	6	-5.3	1.00	0.92	1.08	0.00
Centroid_HS_various_temp-0	FHL1_RAP1_YAP6	7	803	8	-5.3	1.00	0.92	1.08	0.00
Centroid_HS_various_temp-0	FHL1_YAP1_YAP6	10	803	11	-7.8	1.00	0.92	1.08	0.00
Centroid_HS_various_temp-0	FHL1_SFP1_YAP1	6	803	7	-4.5	1.00	0.92	1.08	0.00
Centroid_HS_various_temp-0	FHL1_SFP1_YAP6	9	803	10	-6.9	1.00	0.92	1.08	0.00
Centroid_HS_various_temp-0	FHL1_RAP1_SFP1_YAP6	5	803	5	-4.4	1.00	0.92	1.08	0.00
Centroid_HS_various_temp-0	YAP1_YAP6	11	803	18	-5.5	1.00	0.92	1.08	0.00
Centroid_HS_various_temp-0	ACE2	28	803	93	-4.8	0.99	0.92	1.07	0.04
Centroid_HS_various_temp-0	FHL1_YAP6	19	803	23	-13.0	0.99	0.92	1.07	0.06
Centroid_HS_various_temp-0	FHL1	154	803	252	-73.7	0.98	0.92	1.07	0.08
Centroid_HS_various_temp-0	FHL1_RAP1	50	803	66	-30.3	0.98	0.92	1.07	0.09
Centroid_HS_various_temp-0	RAP1	56	803	109	-21.1	0.98	0.92	1.06	0.09
Centroid_HS_various_temp-0	STB1_SWI6	11	803	22	-4.4	0.98	0.92	1.06	0.03
Centroid_HS_various_temp-0	FHL1_SFP1	44	803	67	-22.5	0.98	0.92	1.06	0.10
Centroid_HS_various_temp-0	SFP1	46	803	73	-22.4	0.98	0.92	1.06	0.10
Centroid_HS_various_temp-0	RME1	10	803	18	-4.6	0.97	0.92	1.05	0.08
Centroid_HS_various_temp-0	FHL1_RAP1_SFP1	25	803	30	-17.1	0.97	0.92	1.05	0.13
Centroid_HS_various_temp-0	RAP1_SFP1	26	803	32	-17.3	0.97	0.92	1.05	0.13
Centroid_HS_various_temp-0	YAP6	26	803	48	-10.7	0.97	0.92	1.05	0.11
Centroid_HS_various_temp-0	SWI4_SWI6	31	803	86	-7.1	0.94	0.92	1.02	0.14
Centroid_HS_various_temp-0	SWI6	35	803	120	-5.4	0.94	0.92	1.02	0.14
Centroid_HS_various_temp-0	SWI4	40	803	149	-5.1	0.92	0.92	1.00	0.21
Centroid_HS_various_temp-3	MSN2_MSN4	12	531	27	-6.0	1.00	0.83	1.20	0.00
Centroid_HS_various_temp-3	HSF1_MSN4	7	531	9	-5.9	0.99	0.83	1.20	0.01
Centroid_HS_various_temp-3	MSN4	24	531	55	-11.2	0.99	0.83	1.20	0.02
Centroid_HS_various_temp-3	MSN2_SKN7	8	531	18	-4.2	0.98	0.83	1.18	0.04

Cluster	Module	# Overla	Cluster	# Targets	p-value	Avg. MB	Avg. MB	Centrality	SD Centr.
Centroid_HS_various_temp-3	ARO80	4	531	4	-4.2	0.98	0.83	1.18	0.05
Centroid_HS_various_temp-3	MSN4_SKN7	6	531	10	-4.1	0.98	0.83	1.18	0.04
Centroid_HS_various_temp-3	HSF1	34	531	74	-16.6	0.95	0.83	1.15	0.20
Centroid_HS_various_temp-3	MSN2	30	531	95	-9.7	0.95	0.83	1.14	0.20
Centroid_HS_various_temp-3	SKN7	26	531	95	-7.0	0.94	0.83	1.14	0.14
Centroid_HS_various_temp-3	HSF1_MSN2	8	531	13	-5.5	0.90	0.83	1.08	0.32
Centroid_HS_various_temp-3	RTG3	9	531	16	-5.7	0.87	0.83	1.05	0.32
Centroid_HS_various_temp-3	MIG1	14	531	39	-5.5	0.80	0.83	0.97	0.35
Centroid_Hyper_osmotic_stress-0	FHL1_RAP1	16	247	66	-8.3	0.68	0.67	1.01	0.43
Centroid_Hyper_osmotic_stress-0	RAP1	16	247	109	-5.1	0.68	0.67	1.01	0.43
Centroid_Hyper_osmotic_stress-0	FHL1_RAP1_SFP1	11	247	30	-7.9	0.63	0.67	0.94	0.43
Centroid_Hyper_osmotic_stress-0	RAP1_SFP1	11	247	32	-7.6	0.63	0.67	0.94	0.43
Centroid_Hyper_osmotic_stress-0	ACE2	16	247	93	-6.0	0.62	0.67	0.92	0.44
Centroid_Hyper_osmotic_stress-0	FHL1	52	247	252	-22.9	0.58	0.67	0.87	0.39
Centroid_Hyper_osmotic_stress-0	FHL1_SFP1	16	247	67	-8.2	0.58	0.67	0.86	0.40
Centroid_Hyper_osmotic_stress-0	SFP1	17	247	73	-8.4	0.58	0.67	0.86	0.39
Centroid_Hyper_osmotic_stress-0	YAP6	10	247	48	-4.7	0.54	0.67	0.80	0.37
Centroid_Hyper_osmotic_stress-0	FHL1_YAP6	8	247	23	-5.7	0.49	0.67	0.74	0.37
Centroid_Hyper_osmotic_stress-0	FHL1_YAP1_YAP6	5	247	11	-4.4	0.42	0.67	0.62	0.28
Centroid_Hyper_osmotic_stress-1	MSN4	18	334	55	-9.4	0.81	0.65	1.24	0.21
Centroid_Hyper_osmotic_stress-1	MSN2_MSN4	10	334	27	-6.1	0.78	0.65	1.20	0.24
Centroid_Hyper_osmotic_stress-1	MSN4_SKN7	6	334	10	-5.3	0.72	0.65	1.11	0.21
Centroid_Hyper_osmotic_stress-1	MSN2	30	334	95	-15.0	0.72	0.65	1.11	0.35
Centroid_Hyper_osmotic_stress-1	SKN7	16	334	95	-4.2	0.71	0.65	1.10	0.30
Centroid_Hyper_osmotic_stress-1	MSN2_SKN7	7	334	18	-4.5	0.70	0.65	1.08	0.25
Centroid_Hyper_osmotic_stress-1	PUT3	8	334	26	-4.3	0.68	0.65	1.05	0.48
Centroid_Hyper_osmotic_stress-1	HSF1_MSN2	6	334	13	-4.5	0.60	0.65	0.92	0.42
Centroid_Hyper_osmotic_stress-12	HSF1	7	20	74	-8.7	0.57	0.47	1.22	0.45
Centroid_Hyper_osmotic_stress-13	MIG1	4	19	39	-5.3	0.70	0.53	1.32	0.59
Centroid_Hyper_osmotic_stress-3	FHL1_MBP1_YAP1_YAP6	3	211	3	-4.4	0.73	0.56	1.30	0.21
Centroid_Hyper_osmotic_stress-3	RAP1	15	211	109	-5.3	0.67	0.56	1.19	0.36
Centroid_Hyper_osmotic_stress-3	FHL1_RAP1	12	211	66	-5.6	0.63	0.56	1.13	0.34
Centroid_Hyper_osmotic_stress-3	FHL1_SFP1	18	211	67	-11.2	0.62	0.56	1.11	0.46
Centroid_Hyper_osmotic_stress-3	FHL1	55	211	252	-29.3	0.61	0.56	1.09	0.45
Centroid_Hyper_osmotic_stress-3	MBP1	21	211	174	-6.2	0.61	0.56	1.08	0.36
Centroid_Hyper_osmotic_stress-3	FHL1_RAP1_SFP1	7	211	30	-4.2	0.60	0.56	1.06	0.37
Centroid_Hyper_osmotic_stress-3	RAP1_SFP1	7	211	32	-4.0	0.60	0.56	1.06	0.37
Centroid_Hyper_osmotic_stress-3	FHL1_YAP1_YAP6	6	211	11	-6.2	0.59	0.56	1.06	0.36
Centroid_Hyper_osmotic_stress-3	YAP1_YAP6	6	211	18	-4.6	0.59	0.56	1.06	0.36
Centroid_Hyper_osmotic_stress-3	SFP1	20	211	73	-12.6	0.59	0.56	1.06	0.47
Centroid_Hyper_osmotic_stress-3	ACE2_FHL1_RAP1	4	211	6	-4.7	0.59	0.56	1.05	0.50
Centroid_Hyper_osmotic_stress-3	FKH2	17	211	145	-5.0	0.57	0.56	1.02	0.43
Centroid_Hyper_osmotic_stress-3	YAP6	11	211	48	-6.3	0.57	0.56	1.01	0.44
Centroid_Hyper_osmotic_stress-3	FHL1_YAP6	9	211	23	-7.4	0.56	0.56	0.99	0.40
Centroid_Hyper_osmotic_stress-3	ACE2	13	211	93	-4.7	0.56	0.56	0.99	0.35
Centroid_Hyper_osmotic_stress-3	SWI4	18	211	149	-5.4	0.54	0.56	0.96	0.40
Centroid_Hyper_osmotic_stress-4	MSN2	10	110	95	-5.1	0.48	0.51	0.95	0.45
Centroid_Hyper_osmotic_stress-6	BAS1	4	25	30	-5.2	0.38	0.50	0.75	0.37
Centroid_Hyper_osmotic_stress-7	SWI6	6	31	120	-4.6	0.65	0.52	1.25	0.61
Centroid_Hyper_osmotic_stress-7	FKH1_FKH2_MBP1_SWI4_SWI6	2	31	3	-4.1	0.62	0.52	1.19	0.95
Centroid_Hyper_osmotic_stress-7	SWI4_SWI6	5	31	86	-4.2	0.62	0.52	1.19	0.66
Centroid_Hyper_osmotic_stress-7	SWI4	6	31	149	-4.0	0.55	0.52	1.07	0.66
Centroid_Hyper_osmotic_stress-7	FHL1	8	31	252	-4.5	0.52	0.52	1.00	0.51
Centroid_Hyper_osmotic_stress-7	DIG1_STE12_SWI4_SWI6	3	31	9	-5.0	0.41	0.52	0.79	0.53
Centroid_Hyper_osmotic_stress-7	DIG1_SWI4_SWI6	3	31	10	-4.8	0.41	0.52	0.79	0.53
Centroid_Hyper_osmotic_stress-7	DIG1_STE12_SWI6	3	31	11	-4.7	0.41	0.52	0.79	0.53
Centroid_Hyper_osmotic_stress-7	DIG1_STE12_SWI4	3	31	11	-4.7	0.41	0.52	0.79	0.53
Centroid_Hypo_osmotic_stress-1	MSN2	16	178	95	-7.9	0.81	0.66	1.24	0.29
Centroid_Hypo_osmotic_stress-1	MSN4	13	178	55	-8.4	0.77	0.66	1.18	0.40
Centroid_Hypo_osmotic_stress-2	BAS1	6	82	30	-5.6	0.68	0.68	1.00	0.44
Centroid_Hypo_osmotic_stress-2	SWI5	6	82	44	-4.6	0.59	0.68	0.87	0.41
Centroid_Hypo_osmotic_stress-3	RCS1	6	62	69	-4.2	0.86	0.62	1.38	0.18
Centroid_Menadione-2	BAS1	10	35	30	-15.5	0.89	0.61	1.46	0.32
Centroid_Menadione-5	CIN5_FKH1_FKH2_MCM1_NDD1_RLM1_SUM1_SWI4_SWI6_YAP6	2	40	2	-4.4	0.73	0.58	1.26	0.28
Centroid_Menadione-5	FKH1_FKH2_SWI4_SWI6	3	40	5	-5.6	0.70	0.58	1.22	0.21
Centroid_Menadione-5	FKH1_SWI4_SWI6	3	40	6	-5.3	0.70	0.58	1.22	0.21
Centroid_Menadione-5	FKH1_FKH2_SWI6	3	40	7	-5.0	0.70	0.58	1.22	0.21
Centroid_Menadione-5	SWI4	7	40	149	-4.3	0.69	0.58	1.20	0.35
Centroid_Menadione-5	SWI4_SWI6	6	40	86	-4.7	0.68	0.58	1.18	0.38
Centroid_Menadione-5	SWI6	7	40	120	-4.9	0.64	0.58	1.11	0.39
Centroid_Menadione-6	HAL9_YAP1_YAP7	2	57	2	-4.1	0.76	0.58	1.31	0.02
Centroid_Menadione-6	RCS1	9	57	69	-7.9	0.71	0.58	1.21	0.46
Centroid_Menadione-6	GCN4	7	57	76	-5.2	0.69	0.58	1.18	0.34
Centroid_Menadione-6	CIN5_YAP1_YAP7	3	57	7	-4.6	0.67	0.58	1.15	0.20
Centroid_Menadione-6	YAP1	8	57	102	-5.4	0.60	0.58	1.02	0.39
Centroid_Menadione-6	YAP1_YAP7	6	57	34	-6.2	0.57	0.58	0.98	0.35
Centroid_Menadione-6	CAD1	5	57	43	-4.3	0.53	0.58	0.91	0.43
Centroid_Menadione-6	YAP7	9	57	67	-8.0	0.53	0.58	0.90	0.32
Centroid_Menadione-7	MSN2_YAP1_YAP7	3	83	7	-4.1	0.99	0.70	1.42	0.01
Centroid_Menadione-7	MSN2_SKN7_YAP7	3	83	4	-5.0	0.95	0.70	1.36	0.10
Centroid_Menadione-7	CAD1_MSN2_YAP7	3	83	4	-5.0	0.95	0.70	1.36	0.10
Centroid_Menadione-7	STB5	5	83	25	-4.7	0.89	0.70	1.28	0.16
Centroid_Menadione-7	CAD1_YAP1	8	83	28	-8.6	0.82	0.70	1.18	0.34
Centroid_Menadione-7	MSN2	18	83	95	-15.6	0.81	0.70	1.17	0.27
Centroid_Menadione-7	MSN2_SKN7	7	83	18	-8.7	0.81	0.70	1.16	0.36
Centroid_Menadione-7	HSF1_MSN2	4	83	13	-4.7	0.80	0.70	1.14	0.27
Centroid_Menadione-7	YAP1	13	83	102	-9.1	0.79	0.70	1.14	0.40
Centroid_Menadione-7	CAD1	11	83	43	-11.1	0.79	0.70	1.13	0.33
Centroid_Menadione-7	CAD1_YAP1_YAP7	6	83	20	-6.7	0.78	0.70	1.12	0.38
Centroid_Menadione-7	YAP1_YAP7	9	83	34	-9.3	0.76	0.70	1.10	0.45
Centroid_Menadione-7	MSN2_MSN4	10	83	27	-12.0	0.76	0.70	1.09	0.32
Centroid_Menadione-7	MSN4	13	83	55	-12.6	0.75	0.70	1.07	0.34
Centroid_Menadione-7	CAD1_YAP7	8	83	29	-8.5	0.75	0.70	1.07	0.38

Cluster	Module	# Overla	Cluster	# Targets	p-value	Avg. MB	Avg. MB	Centrality	SD Centr.
Centroid_Menadione-7	YAP7	12	83	67	-10.2	0.72	0.70	1.04	0.42
Centroid_Menadione-7	MSN2_MSN4_SKN7	4	83	7	-5.9	0.70	0.70	1.01	0.43
Centroid_Menadione-7	SKN7	12	83	95	-8.3	0.70	0.70	1.01	0.42
Centroid_Menadione-7	MSN4_SKN7	5	83	10	-7.0	0.63	0.70	0.90	0.44
Centroid_Nitrogen_depl-0	ACE2_FHL1_RAP1	6	351	6	-7.4	0.98	0.72	1.37	0.02
Centroid_Nitrogen_depl-0	FHL1_SFP1_YAP6	9	351	10	-10.2	0.89	0.72	1.25	0.27
Centroid_Nitrogen_depl-0	FHL1_SFP1_YAP1	6	351	7	-6.6	0.87	0.72	1.21	0.33
Centroid_Nitrogen_depl-0	FHL1_RAP1_SFP1_YAP6	5	351	5	-6.2	0.85	0.72	1.19	0.36
Centroid_Nitrogen_depl-0	FHL1_SFP1_YAP1_YAP6	5	351	6	-5.4	0.85	0.72	1.18	0.35
Centroid_Nitrogen_depl-0	FHL1_SFP1	43	351	67	-36.7	0.84	0.72	1.17	0.29
Centroid_Nitrogen_depl-0	SFP1	43	351	73	-34.4	0.84	0.72	1.17	0.29
Centroid_Nitrogen_depl-0	FHL1_YAP1_YAP6	10	351	11	-11.4	0.84	0.72	1.17	0.33
Centroid_Nitrogen_depl-0	YAP1_YAP6	10	351	18	-7.9	0.84	0.72	1.17	0.33
Centroid_Nitrogen_depl-0	FHL1_YAP6	19	351	23	-19.8	0.84	0.72	1.17	0.33
Centroid_Nitrogen_depl-0	FHL1_RAP1_SFP1	23	351	30	-22.5	0.82	0.72	1.15	0.35
Centroid_Nitrogen_depl-0	RAP1_SFP1	23	351	32	-21.4	0.82	0.72	1.15	0.35
Centroid_Nitrogen_depl-0	YAP6	21	351	48	-13.4	0.82	0.72	1.15	0.36
Centroid_Nitrogen_depl-0	FHL1_RAP1_SFP1_YAP1	4	351	4	-4.9	0.81	0.72	1.13	0.39
Centroid_Nitrogen_depl-0	FHL1_RAP1	43	351	66	-37.1	0.81	0.72	1.13	0.34
Centroid_Nitrogen_depl-0	RAP1	43	351	109	-25.0	0.81	0.72	1.13	0.34
Centroid_Nitrogen_depl-0	YAP1	23	351	102	-7.9	0.81	0.72	1.12	0.36
Centroid_Nitrogen_depl-0	FHL1	138	351	252	-110.9	0.81	0.72	1.12	0.31
Centroid_Nitrogen_depl-0	ACE2	19	351	93	-5.9	0.80	0.72	1.11	0.36
Centroid_Nitrogen_depl-0	CIN5_FHL1_YAP1_YAP6	4	351	4	-4.9	0.80	0.72	1.11	0.38
Centroid_Nitrogen_depl-0	FHL1_RAP1_YAP6	7	351	8	-7.8	0.79	0.72	1.10	0.43
Centroid_Nitrogen_depl-0	CIN5_FHL1_YAP6	7	351	8	-7.8	0.77	0.72	1.08	0.42
Centroid_Nitrogen_depl-0	CIN5_YAP6	7	351	14	-5.3	0.77	0.72	1.08	0.42
Centroid_Nitrogen_depl-0	RME1	8	351	18	-5.5	0.65	0.72	0.91	0.25
Centroid_Nitrogen_depl-1	YAP6	8	167	48	-4.4	0.47	0.58	0.82	0.49
Centroid_Nitrogen_depl-1	RAP1	14	167	109	-5.8	0.43	0.58	0.74	0.28
Centroid_Nitrogen_depl-1	FHL1_RAP1	12	167	66	-6.7	0.41	0.58	0.70	0.28
Centroid_Nitrogen_depl-1	FHL1	28	167	252	-9.7	0.41	0.58	0.70	0.26
Centroid_Nitrogen_depl-1	FHL1_YAP6_YAP7	3	167	4	-4.1	0.24	0.58	0.42	0.09
Centroid_Nitrogen_depl-13	MSN4	5	43	55	-4.4	0.50	0.41	1.24	0.60
Centroid_Nitrogen_depl-13	MSN2	7	43	95	-5.4	0.41	0.41	1.01	0.61
Centroid_Nitrogen_depl-14	MSN2	6	30	95	-5.2	0.53	0.44	1.20	0.51
Centroid_Nitrogen_depl-17	HIR1_HIR2_MBP1_SWI4	2	9	2	-5.7	0.64	0.49	1.29	0.98
Centroid_Nitrogen_depl-17	HIR1_HIR2_SWI4	4	9	6	-10.5	0.63	0.49	1.28	0.73
Centroid_Nitrogen_depl-17	SWI4	4	9	149	-4.4	0.63	0.49	1.28	0.73
Centroid_Nitrogen_depl-17	HIR1_HIR2_HIR3_SWI4	2	9	4	-4.9	0.63	0.49	1.28	0.80
Centroid_Nitrogen_depl-17	HIR1_HIR2_SWI4_YAP1	2	9	3	-5.2	0.60	0.49	1.22	0.88
Centroid_Nitrogen_depl-17	HIR1_HIR2	5	9	8	-13.0	0.56	0.49	1.14	0.71
Centroid_Nitrogen_depl-17	HIR2	5	9	9	-12.6	0.56	0.49	1.14	0.71
Centroid_Nitrogen_depl-17	HIR1	5	9	12	-11.8	0.56	0.49	1.14	0.71
Centroid_Nitrogen_depl-17	HIR1_HIR2_MBP1	3	9	3	-8.6	0.52	0.49	1.05	0.81
Centroid_Nitrogen_depl-17	HIR1_HIR2_HIR3	3	9	5	-7.6	0.51	0.49	1.04	0.70
Centroid_Nitrogen_depl-17	HIR3	3	9	6	-7.3	0.51	0.49	1.04	0.70
Centroid_Nitrogen_depl-17	FKH2_HIR1_HIR2_HIR3	2	9	3	-5.2	0.32	0.49	0.64	0.10
Centroid_Nitrogen_depl-19	LEU3	4	32	33	-4.6	0.78	0.42	1.86	0.55
Centroid_Nitrogen_depl-19	GCN4_LEU3	3	32	9	-4.9	0.72	0.42	1.72	0.58
Centroid_Nitrogen_depl-19	GCN4	5	32	76	-4.4	0.64	0.42	1.52	0.50
Centroid_Nitrogen_depl-2	NDD1_STE12_SWI4_SWI6	7	598	10	-5.1	0.90	0.73	1.24	0.13
Centroid_Nitrogen_depl-2	DIG1_STE12_SWI4_SWI6	8	598	9	-7.1	0.89	0.73	1.22	0.13
Centroid_Nitrogen_depl-2	DIG1_SWI4_SWI6	8	598	10	-6.5	0.89	0.73	1.22	0.13
Centroid_Nitrogen_depl-2	DIG1_STE12_SWI6	8	598	11	-5.9	0.89	0.73	1.22	0.13
Centroid_Nitrogen_depl-2	NDD1_STE12_SWI4	8	598	12	-5.5	0.89	0.73	1.22	0.13
Centroid_Nitrogen_depl-2	STE12_SWI4_SWI6	15	598	25	-9.0	0.88	0.73	1.22	0.17
Centroid_Nitrogen_depl-2	STE12_SWI6	15	598	29	-7.8	0.88	0.73	1.22	0.17
Centroid_Nitrogen_depl-2	FKH2_NDD1_STE12	6	598	9	-4.2	0.88	0.73	1.21	0.13
Centroid_Nitrogen_depl-2	SWI4_SWI6	26	598	86	-6.9	0.87	0.73	1.21	0.22
Centroid_Nitrogen_depl-2	STE12_SWI4	18	598	31	-10.4	0.87	0.73	1.20	0.18
Centroid_Nitrogen_depl-2	NDD1_STE12	13	598	23	-7.4	0.86	0.73	1.19	0.22
Centroid_Nitrogen_depl-2	DIG1_STE12_SWI4	9	598	11	-7.4	0.86	0.73	1.19	0.16
Centroid_Nitrogen_depl-2	MCM1_NDD1_STE12	7	598	13	-4.0	0.83	0.73	1.14	0.28
Centroid_Nitrogen_depl-2	DIG1_NDD1_STE12	6	598	8	-4.7	0.81	0.73	1.12	0.30
Centroid_Nitrogen_depl-2	SWI6	34	598	120	-8.1	0.81	0.73	1.11	0.31
Centroid_Nitrogen_depl-2	NDD1	38	598	143	-8.1	0.81	0.73	1.11	0.29
Centroid_Nitrogen_depl-2	MCM1_STE12	10	598	20	-5.2	0.81	0.73	1.11	0.26
Centroid_Nitrogen_depl-2	DIG1_STE12	32	598	68	-14.5	0.79	0.73	1.08	0.29
Centroid_Nitrogen_depl-2	DIG1	34	598	86	-12.6	0.78	0.73	1.08	0.30
Centroid_Nitrogen_depl-2	STE12	60	598	157	-21.1	0.78	0.73	1.08	0.29
Centroid_Nitrogen_depl-2	SWI4	46	598	149	-12.2	0.78	0.73	1.07	0.32
Centroid_Nitrogen_depl-2	MCM1_NDD1	17	598	51	-5.4	0.77	0.73	1.07	0.32
Centroid_Nitrogen_depl-2	FKH2_NDD1	17	598	48	-5.8	0.76	0.73	1.04	0.30
Centroid_Nitrogen_depl-2	MCM1	27	598	103	-5.8	0.76	0.73	1.04	0.29
Centroid_Nitrogen_depl-2	STB1_SWI4	16	598	44	-5.7	0.75	0.73	1.03	0.33
Centroid_Nitrogen_depl-2	FKH2_MCM1_NDD1	11	598	26	-4.8	0.74	0.73	1.03	0.33
Centroid_Nitrogen_depl-2	STB1	17	598	47	-5.9	0.73	0.73	1.01	0.33
Centroid_Nitrogen_depl-2	FKH2	35	598	145	-6.4	0.72	0.73	1.00	0.35
Centroid_Nitrogen_depl-2	MBP1_SWI4	16	598	49	-5.0	0.63	0.73	0.87	0.38
Centroid_Nitrogen_depl-2	SWI5	15	598	44	-4.9	0.63	0.73	0.87	0.43
Centroid_Nitrogen_depl-2	ACE2_STE12_SWI5	4	598	4	-4.0	0.63	0.73	0.86	0.51
Centroid_Nitrogen_depl-2	MBP1_STB1_SWI4	7	598	13	-4.0	0.62	0.73	0.85	0.42
Centroid_Nitrogen_depl-2	MBP1_STB1	8	598	14	-4.8	0.60	0.73	0.83	0.39
Centroid_Nitrogen_depl-2	ACE2_SWI5	7	598	13	-4.0	0.60	0.73	0.83	0.42
Centroid_Nitrogen_depl-3	FKH1_FKH2_NDD1	5	265	10	-4.5	0.65	0.52	1.24	0.35
Centroid_Nitrogen_depl-3	FKH2_NDD1	17	265	48	-11.2	0.63	0.52	1.20	0.39
Centroid_Nitrogen_depl-3	NDD1	23	265	143	-7.3	0.62	0.52	1.18	0.38
Centroid_Nitrogen_depl-3	FKH2_MCM1_NDD1	12	265	26	-9.6	0.62	0.52	1.18	0.36
Centroid_Nitrogen_depl-3	MCM1_NDD1	13	265	51	-6.7	0.61	0.52	1.16	0.35
Centroid_Nitrogen_depl-3	MCM1_STE12	7	265	20	-4.9	0.60	0.52	1.15	0.50
Centroid_Nitrogen_depl-3	FKH2_NDD1_SWI4	5	265	12	-4.0	0.59	0.52	1.13	0.46
Centroid_Nitrogen_depl-3	FKH2	26	265	145	-9.3	0.57	0.52	1.09	0.40

Cluster	Module	# Overla	Cluster	# Targets	p-value	Avg. MB	Avg. MB	Centrality	SD Centr.
Centroid_Nitrogen_depl-3	FKH1_FKH2	8	265	31	-4.4	0.56	0.52	1.07	0.39
Centroid_Nitrogen_depl-3	MCM1	20	265	103	-7.8	0.55	0.52	1.05	0.40
Centroid_Nitrogen_depl-3	SWI4_SWI6	13	265	86	-4.1	0.49	0.52	0.93	0.41
Centroid_Nitrogen_depl-3	STE12	19	265	157	-4.3	0.47	0.52	0.90	0.44
Centroid_Nitrogen_depl-3	SWI6	19	265	120	-6.0	0.47	0.52	0.90	0.35
Centroid_Nitrogen_depl-3	DIG1	13	265	86	-4.1	0.45	0.52	0.87	0.45
Centroid_Nitrogen_depl-3	DIG1_STE12	12	265	68	-4.5	0.45	0.52	0.85	0.46
Centroid_Nitrogen_depl-3	SWI5	11	265	44	-5.7	0.39	0.52	0.75	0.38
Centroid_Nitrogen_depl-4	PHD1_SKN7	7	848	10	-4.1	0.81	0.77	1.06	0.23
Centroid_Nitrogen_depl-5	CBF1_MET32_MET4	4	108	9	-4.9	0.84	0.58	1.44	0.30
Centroid_Nitrogen_depl-5	CBF1_MET32	7	108	20	-7.5	0.82	0.58	1.42	0.31
Centroid_Nitrogen_depl-5	MET32	8	108	64	-4.8	0.82	0.58	1.41	0.29
Centroid_Nitrogen_depl-5	CBF1_MET32_MET4_TYE7	3	108	4	-4.7	0.78	0.58	1.35	0.30
Centroid_Nitrogen_depl-5	MET32_MET4_TYE7	3	108	5	-4.3	0.78	0.58	1.35	0.30
Centroid_Nitrogen_depl-5	CBF1_MET31_MET32_TYE7	3	108	4	-4.7	0.76	0.58	1.31	0.35
Centroid_Nitrogen_depl-5	MET31_MET32_TYE7	3	108	5	-4.3	0.76	0.58	1.31	0.35
Centroid_Nitrogen_depl-5	CBF1_MET32_TYE7	5	108	7	-7.5	0.76	0.58	1.30	0.29
Centroid_Nitrogen_depl-5	CBF1_TYE7	6	108	40	-4.2	0.72	0.58	1.24	0.30
Centroid_Nitrogen_depl-5	CBF1	14	108	156	-6.2	0.72	0.58	1.24	0.37
Centroid_Nitrogen_depl-5	BAS1	15	108	30	-18.5	0.70	0.58	1.21	0.43
Centroid_Nitrogen_depl-5	GCN4	20	108	76	-18.0	0.61	0.58	1.05	0.45
Centroid_Nitrogen_depl-5	LEU3	7	108	33	-5.8	0.54	0.58	0.93	0.50
Centroid_Nitrogen_depl-5	RTG3	6	108	16	-6.7	0.52	0.58	0.90	0.43
Centroid_Nitrogen_depl-6	HSF1	13	296	74	-4.3	0.53	0.51	1.04	0.50
Centroid_Nitrogen_depl-7	MET31_MET32	11	121	47	-8.9	0.80	0.54	1.48	0.40
Centroid_Nitrogen_depl-7	MET31	12	121	49	-9.9	0.78	0.54	1.46	0.40
Centroid_Nitrogen_depl-7	CBF1_MET31_MET32	5	121	14	-5.3	0.77	0.54	1.43	0.50
Centroid_Nitrogen_depl-7	MET32	14	121	64	-10.8	0.72	0.54	1.34	0.52
Centroid_Nitrogen_depl-7	CBF1_MET32	7	121	20	-7.2	0.71	0.54	1.32	0.54
Centroid_Nitrogen_depl-7	GCN4	12	121	76	-7.6	0.70	0.54	1.30	0.42
Centroid_Nitrogen_depl-7	GLN3	11	121	47	-8.9	0.63	0.54	1.17	0.52
Centroid_Nitrogen_depl-7	CBF1	15	121	156	-6.4	0.60	0.54	1.13	0.58
Centroid_Nitrogen_depl-7	DAL82	6	121	24	-5.2	0.59	0.54	1.11	0.55
Centroid_Nitrogen_depl-7	DAL82_GLN3	5	121	10	-6.1	0.57	0.54	1.06	0.60
Centroid_Nitrogen_depl-9	FHL1	10	47	252	-4.7	0.54	0.42	1.29	0.67
Centroid_Steady_state-1-0	BAS1	9	232	30	-5.9	0.91	0.76	1.20	0.23
Centroid_Steady_state-1-0	UME6	18	232	171	-4.0	0.69	0.76	0.92	0.39
Centroid_Steady_state-1-1	MSN2	17	165	95	-9.3	0.80	0.69	1.17	0.36
Centroid_Steady_state-1-1	SKN7	12	165	95	-5.0	0.71	0.69	1.02	0.38
Centroid_Steady_state-1-1	MSN2_MSN4	7	165	27	-5.2	0.70	0.69	1.02	0.42
Centroid_Steady_state-1-1	MSN2_SKN7	7	165	18	-6.6	0.68	0.69	0.99	0.42
Centroid_Steady_state-1-1	PUT3	6	165	26	-4.2	0.65	0.69	0.94	0.50
Centroid_Steady_state-1-1	MSN4	12	165	55	-7.7	0.64	0.69	0.92	0.42
Centroid_Steady_state-1-1	MSN2_MSN4_SKN7	4	165	7	-4.7	0.60	0.69	0.86	0.46
Centroid_Steady_state-1-1	HSF1	10	165	74	-4.5	0.55	0.69	0.79	0.45
Centroid_Steady_state-1-3	PHO4	7	186	32	-4.4	0.87	0.77	1.13	0.20
Centroid_Steady_state-1-8	RCS1	7	37	69	-6.8	0.69	0.54	1.27	0.67
Centroid_Steady_state-2-1	FHL1	19	112	252	-6.8	0.54	0.57	0.95	0.38
Centroid_Steady_state-2-2	ACE2_SWI5	5	237	13	-4.0	0.68	0.66	1.04	0.27
Centroid_Steady_state-2-2	YAP5	8	237	12	-8.6	0.65	0.66	0.98	0.22
Centroid_Steady_state-2-2	SWI5_YAP5	4	237	5	-4.9	0.60	0.66	0.91	0.25
Centroid_Steady_state-2-3	YAP1_YAP7	7	168	34	-4.5	0.70	0.58	1.21	0.42
Centroid_Steady_state-2-4	MSN2_MSN4	6	106	27	-5.2	0.84	0.51	1.66	0.36
Centroid_Steady_state-2-4	MSN2	12	106	95	-7.1	0.80	0.51	1.57	0.48
Centroid_Steady_state-2-4	PUT3	5	106	26	-4.1	0.70	0.51	1.37	0.37
Centroid_Steady_state-2-4	MSN4	11	106	55	-8.7	0.70	0.51	1.36	0.54
Centroid_Steady_state-2-6	MSN4	6	62	55	-4.7	0.40	0.41	0.97	0.42
Centroid_Steady_state-2-8	ARG81_CIN5_GCN4	2	47	2	-4.2	0.47	0.40	1.18	0.74
Centroid_YPD-2-16	ARG81_CIN5_GCN4	2	40	2	-4.4	0.78	0.39	1.99	0.47
Centroid_YPD-2-3	HAP1_HAP4	8	777	12	-4.6	0.88	0.57	1.54	0.19
Centroid_YPD-2-3	MSN2_MSN4	13	777	27	-5.0	0.86	0.57	1.51	0.30
Centroid_YPD-2-3	MSN2	27	777	95	-4.4	0.78	0.57	1.36	0.40
Centroid_YPD-2-3	MSN4	21	777	55	-5.7	0.77	0.57	1.34	0.46
Centroid_YPD-2-3	HAP4	35	777	62	-15.4	0.73	0.57	1.27	0.40
Centroid_YPD-2-3	MIG1	17	777	39	-5.7	0.64	0.57	1.13	0.50
Centroid_YPD-2-4	FHL1_SFP1_YAP1	7	1393	7	-4.4	0.99	0.72	1.38	0.03
Centroid_YPD-2-4	FHL1_YAP1_YAP6	11	1393	11	-7.0	0.98	0.72	1.37	0.03
Centroid_YPD-2-4	YAP1_YAP6	14	1393	18	-5.8	0.93	0.72	1.30	0.27
Centroid_YPD-2-4	FHL1_YAP6	22	1393	23	-12.8	0.92	0.72	1.29	0.27
Centroid_YPD-2-4	FHL1_SFP1_YAP6	10	1393	10	-6.4	0.92	0.72	1.28	0.30
Centroid_YPD-2-4	FHL1_RAP1	49	1393	66	-17.8	0.91	0.72	1.27	0.23
Centroid_YPD-2-4	FHL1_RAP1_YAP6	8	1393	8	-5.1	0.90	0.72	1.26	0.32
Centroid_YPD-2-4	YAP6	33	1393	48	-10.7	0.89	0.72	1.25	0.31
Centroid_YPD-2-4	FHL1	170	1393	252	-52.2	0.89	0.72	1.24	0.27
Centroid_YPD-2-4	FHL1_SFP1	47	1393	67	-15.5	0.89	0.72	1.24	0.29
Centroid_YPD-2-4	FHL1_RAP1_SFP1	24	1393	30	-10.2	0.89	0.72	1.24	0.25
Centroid_YPD-2-4	SFP1	49	1393	73	-14.9	0.88	0.72	1.23	0.30
Centroid_YPD-2-4	RAP1_SFP1	25	1393	32	-10.2	0.87	0.72	1.21	0.27
Centroid_YPD-2-4	CIN5_YAP6	11	1393	14	-4.7	0.86	0.72	1.20	0.35
Centroid_YPD-2-4	RAP1	62	1393	109	-13.7	0.84	0.72	1.18	0.33
Centroid_YPD-2-4	ACE2	38	1393	93	-4.0	0.83	0.72	1.16	0.35
Centroid_YPD-2-4	YAP1	42	1393	102	-4.5	0.82	0.72	1.14	0.34
Centroid_YPD-2-4	STB1_SWI4	22	1393	44	-4.0	0.72	0.72	1.00	0.36
Centroid_YPD-2-4	STB1	23	1393	47	-4.0	0.71	0.72	1.00	0.36
Centroid_YPD-2-4	BAS1	18	1393	30	-4.8	0.71	0.72	0.99	0.31
Centroid_YPD-2-4	STE12_SWI4_SWI6	17	1393	25	-5.6	0.69	0.72	0.97	0.36
Centroid_YPD-2-4	STE12_SWI4	19	1393	31	-5.2	0.69	0.72	0.96	0.35
Centroid_YPD-2-4	SWI4	71	1393	149	-10.5	0.69	0.72	0.96	0.37
Centroid_YPD-2-4	SWI4_SWI6	47	1393	86	-9.7	0.68	0.72	0.94	0.36
Centroid_YPD-2-4	MBP1	71	1393	174	-6.9	0.68	0.72	0.94	0.36
Centroid_YPD-2-4	SWI6	54	1393	120	-7.1	0.67	0.72	0.94	0.36
Centroid_YPD-2-4	STE12_SWI6	18	1393	29	-5.1	0.67	0.72	0.94	0.38
Centroid_YPD-2-4	STE12	59	1393	157	-4.5	0.66	0.72	0.92	0.35

Cluster	Module	# Overla	Cluster	# Targets	p-value	Avg. MB	Avg. MB	Centrality	SD Centr.
Centroid_YPD-2-4	MBP1_SWI4	24	1393	49	-4.2	0.64	0.72	0.89	0.35
Centroid_YPD-2-4	NDD1	55	1393	143	-4.6	0.63	0.72	0.88	0.40
Centroid_YPD-2-4	FKH2	54	1393	145	-4.1	0.61	0.72	0.85	0.38
Centroid_YPD-2-5	RPN4	29	560	137	-4.8	0.53	0.50	1.05	0.47
Centroid_YPD-2-5	GCR2	8	560	15	-4.7	0.46	0.50	0.91	0.31
Centroid_YPD-2-7	MBP1	11	62	174	-5.9	0.41	0.41	0.99	0.52
