# Supplementary material for: Integrated Assessment and Prediction of Transcription Factor Binding
Source: PLoS Comput Biol. 2006 Jun 16;2(6):e70. doi: 10.1371/journal.pcbi.0020070 (PMC1479087; doi:10.1371/journal.pcbi.0020070)
Supplement: Supplementary file 3 [file pcbi.0020070.st005.pdf]

TF	Target
ABF1	YAL054C
ABF1	YGR059W
ABF1	YHR051W
ABF1	YJL166W
ABF1	YOL086C
ABF1	YOR204W
ACE2	YDL127W
ACE2	YGR044C
ACE2	YJL194W
ACE2	YKL185W
ACE2	YLR079W
ACE2	YLR286C
ACE2	YNL327W
ADR1	YAL054C
ADR1	YDR256C
ADR1	YGL205W
ADR1	YHL032C
ADR1	YIL160C
ADR1	YKR009C
ADR1	YMR303C
ARG80	YPL111W
ARG81	YPL111W
BAS1	YAR015W
BAS1	YBR248C
BAS1	YCL030C
BAS1	YDR408C
BAS1	YGL234W
BAS1	YIL020C
BAS1	YKR080W
BAS1	YLR058C
BAS1	YLR359W
BAS1	YNL220W
BAS1	YOR128C
BAS1	YPR035W
CBF1	YDR007W
CBF1	YIR017C
CBF1	YJL166W
CBF1	YLR081W
CBF1	YOR065W
CBF1	YPR167C
CHA4	YCL064C
CRZ1	YGL006W
CUP9	YKR093W
DAL80	YBR208C
DAL80	YDL210W
DAL80	YEL063C
DAL80	YFL021W
DAL80	YGR019W
DAL80	YHL016C
DAL80	YHR037W
DAL80	YIR027C
DAL80	YIR028W
DAL80	YIR029W
DAL80	YIR031C
DAL80	YIR032C
DAL80	YJR152W
DAL80	YKR039W
DAL80	YLR142W
DAL80	YOR348C
DAL80	YOR375C
DAL81	YBR208C
DAL81	YDL210W
DAL81	YGR019W
DAL81	YHL016C
DAL81	YIR028W
DAL81	YIR029W
DAL81	YIR031C
DAL81	YLR438W
FZF1	YPL092W
GAL3	YMR105C
GAL4	YBR018C
GAL4	YBR019C
GAL4	YBR020W
GAL4	YDR009W
GAL4	YLR081W
GAL4	YML051W
GAL4	YOR120W
GCN4	YAR015W
GCN4	YBR249C
GCN4	YCL030C
GCN4	YDR035W
GCN4	YDR037W
GCN4	YDR158W
GCN4	YDR300C
GCN4	YDR354W
GCN4	YER052C
GCN4	YER086W
GCN4	YGL026C
GCN4	YHR018C
GCN4	YHR025W
GCN4	YJR109C
GCN4	YLR451W
GCN4	YML116W
GCN4	YMR108W
GCN4	YMR300C
GCN4	YOR128C
GCN4	YOR202W
GCN4	YOR303W
GCN4	YPR167C
GCR1	YBR118W
GCR1	YCL040W
GCR1	YCL050C
GCR1	YGR254W
GCR1	YHR174W
GCR1	YKL152C
GCR1	YOL086C
GCR1	YPR080W
GCR2	YGR254W
GLN3	YBR208C
GLN3	YDL210W
GLN3	YDL215C
GLN3	YEL060C
GLN3	YEL063C
GLN3	YFL021W
GLN3	YHL016C
GLN3	YHR037W
GLN3	YIR027C
GLN3	YIR028W
GLN3	YIR029W
GLN3	YIR031C
GLN3	YIR032C
GLN3	YJR152W
GLN3	YKR034W
GLN3	YKR039W
GLN3	YLR142W
GLN3	YNL142W
GLN3	YOR348C
GLN3	YOR375C
GLN3	YPR035W
HAC1	YJL034W
HAP1	YDR044W
HAP1	YEL039C
HAP1	YGR088W
HAP1	YHR007C
HAP1	YJR048W
HAP1	YML054C
HAP1	YOR065W
HAP1	YPR065W
HAP2	YDL205C
HAP2	YDR148C
HAP2	YDR178W
HAP2	YDR232W
HAP2	YFL018C
HAP2	YGL187C
HAP2	YHR008C
HAP2	YHR051W
HAP2	YIL125W
HAP2	YJR048W
HAP2	YKL141W
HAP2	YKL148C
HAP2	YNL052W
HAP2	YNR001C
HAP2	YOR065W
HAP2	YOR375C
HAP2	YPR145W
HAP3	YDL205C
HAP3	YDR148C
HAP3	YDR232W
HAP3	YFL018C
HAP3	YHR051W
HAP3	YIL125W
HAP3	YJR048W
HAP3	YNR001C
HAP3	YOR375C
HAP4	YFL018C
HAP4	YHR008C
HAP4	YHR051W
HAP4	YIL162W
HAP4	YJR048W
HAP4	YNR001C
HAP4	YOR065W
HOG1	YDL022W
HSF1	YAL005C
HSF1	YBL075C
HSF1	YBR072W
HSF1	YER103W
HSF1	YJL034W
HSF1	YLL026W
HSF1	YMR186W
HSF1	YPL240C
IME1	YDR403W
IME1	YDR522C
IME1	YDR523C
IME1	YHL022C
IME1	YHR014W
IME1	YIL072W
IME1	YJL106W
IME1	YMR133W
IME1	YMR139W
IME1	YNL210W
IME1	YOR313C
IME4	YJR094C
INO2	YBR029C
INO2	YDR497C
INO2	YER026C
INO2	YGL077C
INO2	YGR157W
INO2	YJL153C
INO2	YJR073C
INO2	YKL182W
INO2	YNR016C
INO2	YPL231W
INO4	YDR123C
INO4	YDR497C
INO4	YER026C
INO4	YGL077C
INO4	YGR157W
INO4	YJL153C
INO4	YJR073C
INO4	YKL182W
INO4	YNR016C
INO4	YPL231W
IXR1	YIL111W
LEU3	YCL018W
LEU3	YGL009C
LEU3	YLR355C
LEU3	YMR108W
LEU3	YNL104C
LEU3	YOR375C
MAC1	YGR088W
MAC1	YLR214W
MAC1	YLR411W
MAC1	YOL152W
MAC1	YPR124W
MAL33	YBR298C
MBP1	YDL164C
MBP1	YDR353W
MBP1	YER070W
MBP1	YGR109C
MBP1	YML021C
MBP1	YMR199W
MBP1	YPR120C
MCM1	YBR202W
MCM1	YCL067C
MCM1	YDR146C
MCM1	YEL032W
MCM1	YER069W
MCM1	YFL026W
MCM1	YGR108W
MCM1	YJL194W
MCM1	YKL178C
MCM1	YKL209C
MCM1	YLR274W
MCM1	YLR438W
MCM1	YNR044W
MCM1	YPL111W
MCM1	YPR119W
MET31	YJR010W
MET31	YKL001C
MET32	YJR010W
MET32	YKL001C
MET4	YIR017C
MET4	YJR010W
MET4	YPR167C
MIG1	YBR020W
MIG1	YFR053C
MIG1	YHR092C
MIG1	YIL162W
MIG1	YMR011W
MIG1	YMR280C

TF	Target
MIG1	YPL248C
MIG2	YBR020W
MSN1	YIR019C
MSN2	YAL062W
MSN2	YBL075C
MSN2	YBR117C
MSN2	YBR126C
MSN2	YBR149W
MSN2	YCL040W
MSN2	YCR021C
MSN2	YDR074W
MSN2	YER103W
MSN2	YFL014W
MSN2	YFR053C
MSN2	YGR088W
MSN2	YHR008C
MSN2	YLL026W
MSN2	YLL039C
MSN2	YML004C
MSN2	YMR105C
MSN2	YMR169C
MSN2	YOL052C-A
MSN4	YAL062W
MSN4	YBL075C
MSN4	YBR117C
MSN4	YBR126C
MSN4	YBR149W
MSN4	YCL040W
MSN4	YCR021C
MSN4	YDR074W
MSN4	YER103W
MSN4	YFL014W
MSN4	YFR053C
MSN4	YGR088W
MSN4	YHR008C
MSN4	YLL026W
MSN4	YML004C
MSN4	YMR105C
MSN4	YMR169C
MSS11	YIR019C
MTH1	YIL162W
OAF1	YDR256C
OAF1	YNL202W
OAF1	YOR363C
OPI1	YDR123C
OPI1	YDR497C
OPI1	YER026C
OPI1	YGL077C
OPI1	YJL153C
PDC2	YLR134W
PDR1	YBL005W
PDR1	YDR011W
PDR1	YDR406W
PDR1	YGR281W
PDR1	YIL013C
PDR1	YJL219W
PDR1	YOL156W
PDR1	YOR153W
PDR1	YOR328W
PDR3	YDR011W
PDR3	YDR406W
PDR3	YGR281W
PDR3	YJL219W
PDR3	YOL156W
PDR3	YOR153W

TF	Target
PHO2	YBR093C
PHO2	YCL030C
PHO2	YGR233C
PHO4	YAR071W
PHO4	YBR093C
PHO4	YGR233C
PHO4	YML123C
PIP2	YDR256C
PIP2	YNL202W
PPR1	YEL021W
PPR1	YKL216W
PPR1	YLR420W
PPR1	YMR271C
PUT3	YHR037W
PUT3	YLR142W
RAP1	YCL030C
RAP1	YDR064W
RAP1	YGR254W
RAP1	YHR174W
RAP1	YOL086C
RCS1	YBR207W
RCS1	YDR270W
RCS1	YER145C
RCS1	YKL220C
RCS1	YLL051C
RCS1	YLR214W
RCS1	YMR058W
RCS1	YMR319C
RCS1	YNL259C
RCS1	YNR060W
RCS1	YOR381W
RCS1	YOR384W
REB1	YBR020W
REB1	YDR146C
REB1	YER086W
REB1	YGR254W
REB1	YNL216W
REB1	YOL004W
RGT1	YDR345C
RGT1	YHR092C
RGT1	YHR094C
RGT1	YIL162W
RGT1	YMR011W
RLM1	YHR030C
RLM1	YKL161C
RLM1	YLR342W
RME1	YJR094C
ROX1	YBR085W
ROX1	YDR044W
ROX1	YEL039C
ROX1	YGL055W
ROX1	YHR007C
ROX1	YIL111W
ROX1	YJR047C
RPN4	YGL048C
RTG1	YCR005C
RTG1	YEL071W
RTG1	YLR304C
RTG1	YNL037C
RTG1	YNR001C
RTG1	YOR136W
RTG3	YCR005C
RTG3	YEL071W
RTG3	YLR304C
RTG3	YNR001C

TF	Target
RTG3	YOR136W
SFP1	YDR113C
SKN7	YDR353W
SKN7	YGR209C
SKN7	YLR342W
SKN7	YML028W
SKO1	YIL162W
SNF1	YGL035C
SNF1	YHR094C
SNF1	YIL160C
SNF1	YKR009C
SNF1	YMR303C
SNF1	YOR125C
SPT10	YBR009C
SPT10	YMR303C
SPT10	YNL030W
SPT23	YGL055W
STE12	YBL016W
STE12	YBR083W
STE12	YCL027W
STE12	YFL026W
STE12	YIL015W
STE12	YIR019C
STE12	YJL157C
STE12	YMR198W
STE12	YMR199W
STE12	YNR044W
STE12	YPR141C
STP1	YBR068C
SWI4	YCR002C
SWI4	YDL127W
SWI4	YER070W
SWI4	YHR030C
SWI4	YLR286C
SWI4	YMR199W
SWI4	YNL289W
SWI4	YPL256C
SWI5	YDL127W
SWI5	YGR044C
SWI5	YJL194W
SWI5	YKL185W
SWI5	YLR079W
SWI5	YLR286C
SWI5	YNL327W
SWI6	YDL164C
SWI6	YDR052C
SWI6	YDR353W
SWI6	YER070W
SWI6	YER111C
SWI6	YGR109C
SWI6	YHR030C
SWI6	YLR286C
SWI6	YML021C
SWI6	YMR199W
SWI6	YNL102W
SWI6	YPL256C
SWI6	YPR120C
THI2	YBR092C
THI2	YOR143C
THI2	YPL214C
UGA3	YDL210W
UGA3	YGR019W
UME6	YAL054C
UME6	YDR123C
UME6	YDR285W

TF	Target
UME6	YER026C
UME6	YGR157W
UME6	YHL022C
UME6	YHR014W
UME6	YIL072W
UME6	YJL106W
UME6	YJL153C
UME6	YJR073C
UME6	YJR094C
UME6	YNL210W
UME6	YOR386W
UME6	YPL111W
XBP1	YMR199W
YAP1	YAL005C
YAP1	YBR008C
YAP1	YDR074W
YAP1	YDR135C
YAP1	YDR353W
YAP1	YFL014W
YAP1	YGR088W
YAP1	YGR209C
YAP1	YHR008C
YAP1	YJL101C
YAP1	YJR104C
YAP1	YML028W
YAP1	YML116W
YAP1	YPL091W
YRR1	YDR011W
ZAP1	YGL255W
